# Genome-wide association study of major anxiety disorders in 122,341 European-ancestry cases identifies 58 loci and highlights GABAergic signaling

**DOI:** 10.1038/s41588-025-02485-8

**Published:** 2026-02-03

**Authors:** Nora I. Strom, Brad Verhulst, Silviu-Alin Bacanu, Rosa Cheesman, Kirstin L. Purves, Hüseyin Gedik, Brittany L. Mitchell, Alex S. Kwong, Annika B. Faucon, Kritika Singh, Sarah Medland, Lucia Colodro-Conde, Kristi Krebs, Per Hoffmann, Stefan Herms, Jan Gehlen, Stephan Ripke, Swapnil Awasthi, Teemu Palviainen, Elisa M. Tasanko, Roseann E. Peterson, Daniel E. Adkins, Andrey A. Shabalin, Mark J. Adams, Matthew H. Iveson, Archie Campbell, Laurent F. Thomas, Bendik S. Winsvold, Ole Kristian Drange, Sigrid Børte, Abigail R. ter Kuile, Joonas Naamanka, Tan-Hoang Nguyen, Sandra M. Meier, Elizabeth C. Corfield, Laurie Hannigan, Daniel F. Levey, Darina Czamara, Heike Weber, Karmel W. Choi, Giorgio Pistis, Baptiste Couvy-Duchesne, Sandra Van der Auwera, Alexander Teumer, Robert Karlsson, Miguel Garcia-Argibay, Donghyung Lee, Rujia Wang, Ottar Bjerkeset, Eystein Stordal, Julia Bäckman, Giovanni A. Salum, Clement C. Zai, James L. Kennedy, Gwyneth Zai, Arun K. Tiwari, Stefanie Heilmann-Heimbach, Börge Schmidt, Jaakko Kaprio, Martin M. Kennedy, Joseph Boden, Alexandra Havdahl, Christel M. Middeldorp, Fabiana L. Lopes, Nirmala Akula, Francis J. McMahon, Elisabeth B. Binder, Lydia Fehm, Andreas Ströhle, Enrique Castelao, Henning Tiemeier, Dan J. Stein, David Whiteman, Catherine Olsen, Zachary Fuller, Xin Wang, Naomi R. Wray, Enda M. Byrne, Glyn Lewis, Nicholas J. Timpson, Lea K. Davis, Ian B. Hickie, Nathan A. Gillespie, Lili Milani, Johannes Schumacher, David P. Woldbye, Andreas J. Forstner, Markus M. Nöthen, Iiris Hovatta, John Horwood, William E. Copeland, Hermine H. Maes, Andrew M. McIntosh, Ole A. Andreassen, John-Anker Zwart, Ole Mors, Anders D. Børglum, Preben B. Mortensen, Helga Ask, Ted Reichborn-Kjennerud, Jackob M. Najman, Murray B. Stein, Joel Gelernter, Yuri Milaneschi, Brenda W. Penninx, Dorret I. Boomsma, Eduard Maron, Angelika Erhardt-Lehmann, Christian Rück, Tilo T. Kircher, Christiane A. Melzig, Georg W. Alpers, Volker Arolt, Katharina Domschke, Jordan W. Smoller, Martin Preisig, Nicholas G. Martin, Michelle K. Lupton, Annemarie I. Luik, Andreas Reif, Hans J. Grabe, Henrik Larsson, Patrik K. Magnusson, Albertine J. Oldehinkel, Catharina A. Hartman, Gerome Breen, Anna R. Docherty, Hilary Coon, Rupert Conrad, Kelli Lehto, Veterans Affairs Million Veteran Program, Daniel F. Levey, Murray B. Stein, Joel Gelernter, FinnGen, Teemu Palviainen, Elisa M. Tasanko, Joonas Naamanka, Jaakko Kaprio, Iiris Hovatta, 23andMe Research Team, Zachary Fuller, Xin Wang, Jürgen Deckert, Thalia C. Eley, Manuel Mattheisen, John M. Hettema

**Affiliations:** 1 Department of Psychology, Humboldt-Universität zu Berlin, Berlin, Germany; 2 Institute of Psychiatric Phenomics and Genomics (IPPG), University Hospital, LMU Munich, Munich, Germany; 3 Centre for Psychiatry Research, Department of Clinical Neuroscience, Karolinska Institutet & Stockholm Health Care Services, Region Stockholm, Stockholm, Sweden; 4 Department of Biomedicine, Aarhus University, Aarhus, Denmark; 5 Psychiatry and Behavioral Sciences, Texas A&M University, College Station, TX USA; 6 Psychiatry, Virginia Commonwealth University, Richmond, VA USA; 7 PROMENTA Centre, Department of Psychology, University of Oslo, Oslo, Norway; 8 Social, Genetic and Developmental Psychiatry Centre, Institute of Psychiatry, Psychology and Neuroscience, King’s College London, London, UK; 9 Institute for Genomics in Health, Department of Psychiatry and Behavioral Sciences, State University of New York Downstate Health Sciences University, Brooklyn, NY USA; 10 Life Sciences, Integrative Life Sciences Doctoral Program, Virginia Commonwealth University, Richmond, VA USA; 11 Human and Molecular Genetics, Virginia Institute for Psychiatric and Behavioral Genetics, Virginia Commonwealth University, Richmond, VA USA; 12 Brain and Mental Health Program, QIMR Berghofer Medical Research Institute, Brisbane, Queensland Australia; 13 Faculty of Medicine, Queensland University, Brisbane, Queensland Australia; 14 Bristol Medical School, Population Health Sciences, MRC Integrative Epidemiology Unit, University of Bristol, Bristol, UK; 15 Centre for Clinical Brain Sciences, Division of Psychiatry, University of Edinburgh, Edinburgh, UK; 16 Division of Medicine, Human Genetics, Vanderbilt University, Nashville, TN USA; 17 Division of Genetic Medicine, Vanderbilt University Medical Center, Nashville, TN USA; 18 Vanderbilt Genetics Institute, Vanderbilt University Medical Center, Nashville, TN USA; 19 School of Psychology, The University of Queensland, Brisbane, Queensland Australia; 20 Estonian Genome Centre, Institute of Genomics, University of Tartu, Tartu, Estonia; 21 Institute of Human Genetics, University of Bonn, School of Medicine & University Hospital Bonn, Bonn, Germany; 22 Department of Biomedicine, Human Genomics Research Group, University of Basel; University Hospital Basel, Basel, Switzerland; 23 Institute of Medical Genetics and Pathology, Medical Faculty, University Hospital Basel, Basel, Switzerland; 24 Center for Human Genetics, University of Marburg, Marburg, Germany; 25 Department of Psychiatry and Psychotherapy, Charité - Universitätsmedizin, Berlin, Germany; 26 Analytic and Translational Genetics Unit, Massachusetts General Hospital, Boston, MA USA; 27 Helsinki Institute of Life Science, Institute for Molecular Medicine Finland - FIMM, University of Helsinki, Helsinki, Finland; 28 Faculty of Medicine, Department of Psychology and Logopedics, SleepWell Research Program, University of Helsinki, Helsinki, Finland; 29 School of Medicine, Department of Psychiatry, University of Utah, Salt Lake City, UT USA; 30 Centre for Clinical Brain Sciences, University of Edinburgh, Edinburgh, UK; 31 College of Medicine and Veterinary Medicine, Institute of Genetics and Cancer; Centre for Genomic and Experimental Medicine, University of Edinburgh, Edinburgh, UK; 32 Department of Clinical and Molecular Medicine, Norwegian University of Science and Technology, Trondheim, Norway; 33 HUNT Center for Molecular and Clinical Epidemiology, Department of Public Health and Nursing, Faculty of Medicine and Health Sciences, Norwegian University of Science and Technology, Trondheim, Norway; 34 BioCore - Bioinformatics Core Facility, Norwegian University of Science and Technology, Trondheim, Norway; 35 Clinic of Laboratory Medicine, St. Olavs Hospital, Trondheim University Hospital, Trondheim, Norway; 36 Division of Clinical Neuroscience, Department of Research and Innovation, Oslo University Hospital, Oslo, Norway; 37 Department of Public Health and Nursing, HUNT Center for Molecular and Clinical Epidemiology, Norwegian University of Science and Technology, Trondheim, Norway; 38 Department of Neurology, Oslo University Hospital, Oslo, Norway; 39 Department of Mental Health, Norwegian University of Science and Technology, Trondheim, Norway; 40 Division of Mental Health, St. Olavs Hospital, Trondheim University Hospital, Trondheim, Norway; 41 NORMENT Centre, University of Oslo, Oslo, Norway; 42 Centre of Precision Psychiatry, Division of Mental Health and Addiction, Oslo University Hospital and University of Oslo, Oslo, Norway; 43 Department of Psychiatry, Sørlandet Hospital, Kristiansand, Norway; 44 Division of Clinical Neuroscience, Department of Research and Innovation; Musculoskeletal Health, Oslo University Hospital, Oslo, Norway; 45 Faculty of Medicine, Institute of Clinical Medicine, University of Oslo, Oslo, Norway; 46 National Institute for Health and Care Research (NIHR) Maudsley Biomedical Research Centre, South London and Maudsley NHS Foundation Trust, London, UK; 47 Department of Clinical, Educational and Health Psychology, University College London, London, UK; 48 Hector Insitute for Artificial Intelligence in Psychiatry, Central Institute of Mental Health, Mannheim, Germany; 49 SleepWell Research Program, Faculty of Medicine, University of Helsinki, Helsinki, Finland; 50 Psychiatry, Dalhousie University, Halifax, Nova Scotia Canada; 51 PsychGen Centre for Genetic Epidemiology and Mental Health, Norwegian Institute of Public Health, Oslo, Norway; 52 Nic Waals Institute, Lovisenberg Diaconal Hospital, Oslo, Norway; 53 Bristol Medical School, Population Health Sciences, University of Bristol, Bristol, UK; 54 Department of Psychiatry, Division of Human Genetics, Yale University School of Medicine, New Haven, CT USA; 55 Psychiatry, Research, Veterans Affairs Connecticut Healthcare System, West Haven, CT USA; 56 Department of Genes and Environment, Max Planck Institute of Psychiatry, Munich, Germany; 57 Department of Psychiatry, Psychosomatics and Psychotherapy, University Hospital of Würzburg, Würzburg, Germany; 58 Psychiatry, Center for Precision Psychiatry, Massachusetts General Hospital, Boston, MA USA; 59 Psychiatry, Psychiatric and Neurodevelopmental Genetics Unit, Center for Genomic Medicine, Massachusetts General Hospital, Boston, MA USA; 60 Psychiatric Epidemiology and Psychopathology Research Center, Department of Psychiatry, Lausanne University Hospital and University of Lausanne, Prilly, Switzerland; 61 ARAMIS laboratory, Paris Brain Institute, Paris, France; 62 Institute for Molecular Bioscience, University of Queensland, Brisbane, Queensland Australia; 63 Department of Psychiatry and Psychotherapy, University Medicine Greifswald, Greifswald, Germany; 64 Institute for Community Medicine, University Medicine Greifswald, Greifswald, Germany; 65 Department of Medical Epidemiology and Biostatistics, Karolinska Institutet, Stockholm, Sweden; 66 School of Medical Sciences, Faculty of Medicine and Health, Örebro University, Örebro, Sweden; 67 Department of Statistics, Miami University, Oxford, OH USA; 68 Social, Genetic, and Developmental Psychiatry Centre, Institute of Psychiatry, Psychology and Neuroscience, King’s College London, London, UK; 69 Faculty of Nursing and Health Science, Nord University, Levanger, Norway; 70 Department of Psychiatry, Hospital Namsos, Nord-Trøndelag Health Trustt, Namsos, Norway; 71 Department of Psychiatry, Universidade Federal do Rio Grande do Sul, Porto Alegre, Brazil; 72 Child Psychiatry, National Institute of Developmental Psychiatry, São Paulo, Brazil; 73 Tanenbaum Centre for Pharmacogenetics, Molecular Brain Sciences Department, Campbell Family Mental Health Institute, Centre for Addiction and Mental Health, Toronto, Ontario Canada; 74 Department of Psychiatry, Division of Neurosciences and Clinical Translation, University of Toronto, Toronto, Ontario Canada; 75 Institute of Medical Science, University of Toronto, Toronto, Ontario Canada; 76 Laboratory Medicine and Pathobiology, University of Toronto, Toronto, Ontario Canada; 77 Stanley Center for Psychiatric Research, Broad Institute of Harvard and MIT, Cambridge, MA USA; 78 Institute for Medical Informatics, Biometry and Epidemiology, University Hospital of Essen, University of Duisburg-Essen, Essen, Germany; 79 Pathology and Biomedical Science, University of Otago, Christchurch, New Zealand; 80 Psychological Medicine, University of Otago, Christchurch, New Zealand; 81 Child Health Research Centre, University of Queensland, Brisbane, Queensland Australia; 82 Child and Youth Mental Health Service, Children’s Health Queensland Hospital and Health Service, Brisbane, Queensland Australia; 83 National Institute of Mental Health, Human Genetics Branch, National Institutes of Health, Bethesda, MD USA; 84 Department of Psychiatry and Human Behavior, Alpert Medical School of Brown University, Providence, RI USA; 85 National Institute of Mental Health, Genetic Basis of Mood and Anxiety Disorders, National Institutes of Health, Bethesda, MA USA; 86 Psychiatry & Behavioral Sciences, Johns Hopkins University, Baltimore, MA USA; 87 Department of Psychology, Zentrum für Psychotherapie, Humboldt-Universität zu Berlin, Berlin, Germany; 88 Department of Psychiatry and Psychotherapy, Campus Charité Mitte, Charité - Universitätsmedizin Berlin, Corporate member of Freie Universität Berlin and Humboldt-Universität zu Berlin, Berlin, Germany; 89 Social and Behavioral Science, T.H. Chan School of Public Health, Harvard University, Boston, MA USA; 90 Child and Adolescent Psychiatry, Erasmus University Medical Center, Rotterdam, Netherlands; 91 SAMRC Unit on Risk & Resilience in Mental Disorders, Department of Psychiatry & Neuroscience Institute, University of Cape Town, Cape Town, South Africa; 92 Population Health Program, QIMR Berghofer Medical Research Institute, Brisbane, Australia; 93 23andMe, Sunnyvale, CA USA; 94 Department of Psychiatry, University of Oxford, Oxford, UK; 95 UCL Division of Psychiatry, University College London, London, UK; 96 Brain and Mind Centre, University of Sydney, Sydney, New South Wales Australia; 97 Department of Neuroscience, Laboratory of Neural Plasticity, University of Copenhagen, Copenhagen, Denmark; 98 Institute of Neuroscience and Medicine (INM-1), Research Center Jülich, Jülich, Germany; 99 Faculty of Medicine, Department of Psychology and Logopedics and SleepWell Research Program, University of Helsinki, Helsinki, Finland; 100 UVM Medical Center, Department of Psychiatry, University of Vermont, Burlington, VT USA; 101 Massey Cancer Center, Virginia Commonwealth University, Richmond, VA USA; 102 K. G. Jebsen Center for Neurodevelopmental disorders, University of Oslo, Oslo, Norway; 103 Department of Psychiatry, Psychosis Research Unit, Aarhus University Hospital, Aarhus, Denmark; 104 The Lundbeck Foundation Initiative for Integrative Psychiatric Research, iPSYCH, Aarhus University, Aarhus, Denmark; 105 Center for Genomics and Personalised Medicine, Aarhus University, Aarhus, Denmark; 106 The National Centre for Register-based Research, Aarhus University, Aarhus, Denmark; 107 Faculty of Medicine, School of Public Health, University of Queensland, Herston, Queensland Australia; 108 Psychiatry, University of California San Diego, La Jolla, CA USA; 109 School of Public Health, University of California San Diego, La Jolla, CA USA; 110 Psychiatry Research, Veterans Affairs Connecticut Healthcare System, West Haven, CT USA; 111 Departments of Genetics and Neuroscience, Yale University of Medicine, New Haven, CT USA; 112 Amsterdam Neuroscience; Amsterdam Public Health, Amsterdam University Medical Center, Amsterdam, The Netherlands; 113 Twin Register and Department of Complex Trait Genetics, Center for Neurogenomics and Cognitive Research, Vrije Universiteit Amsterdam, Amsterdam, The Netherlands; 114 Amsterdam Public Health, Amsterdam University Medical Center, Amsterdam, The Netherlands; 115 Centre for Digital Health: Department of Health Technologies, Tallinn University of Technology, Tallinn, Estonia; 116 Department of Medicine, Centre for Neuropsychopharmacology, Division of Brain Sciences, Imperial College London, London, UK; 117 Department of Psychiatry, Psychosomatics and Psychotherapy, Center of Mental Health, University Hospital Würzburg, Würzburg, Germany; 118 Department of Psychiatry, University of Marburg, Marburg, Germany; 119 Psychology, Clinical Psychology, Experimental Psychopathology and Psychotherapy, University of Marburg, Marburg, Germany; 120 Psychology, Biological and Clinical Psychology, University of Greifswald, Greifswald, Germany; 121 School of Social Sciences, Department of Psychology, University of Mannheim, Mannheim, Germany; 122 Department of Mental Health, Institute for Translational Psychiatry, University of Muenster, Muenster, Germany; 123 Department of Psychiatry and Psychotherapy, Medical Center, Faculty of Medicine, University of Freiburg, Freiburg, Germany; 124 German Center for Mental Health (DZPG), Partner Site Berlin, Berlin, Germany; 125 Faculty of Health, Queensland University of Technology, Brisbane, Queensland Australia; 126 Epidemiology, Erasmus University Medical Center, Rotterdam, The Netherlands; 127 Department of Psychiatry, Psychosomatic Medicine and Psychotherapy, University Hospital Frankfurt - Goethe University, Frankfurt, Germany; 128 Psychiatry, Interdisciplinary Center Psychopathology and Emotion Regulation, University of Groningen, University Medical Center Groningen, Groningen, The Netherlands; 129 School of Medicine, Psychiatry, University of Utah, Salt Lake City, UT USA; 130 School of Medicine, Psychiatry; Huntsman Mental Health Institute, University of Utah, Salt Lake City, UT USA; 131 Department of Psychosomatic Medicine and Psychotherapy, University Hospital Münster, Münster, Germany; 132 Community Health and Epidemiology, Dalhousie University, Halifax, Nova Scotia Canada; 133 Computer Science, Dalhousie University, Halifax, Nova Scotia Canada

**Keywords:** Psychiatric disorders, Genetic association study

## Abstract

The major anxiety disorders (ANX; including generalized anxiety disorder, panic disorder and phobias) are highly prevalent, often onset early and cause substantial global disability. Although distinct in their clinical presentations, they probably represent differential expressions of a dysregulated threat–response system. Here, we present a genome-wide association meta-analysis comprising 122,341 European ancestry ANX cases and 729,881 controls. We identified 58 independent genome-wide significant risk variants and 66 genes with robust biological support. In an independent sample of 1,175,012 self-report ANX cases and 1,956,379 controls, 51 out of the 58 associations replicated. As predicted by twin studies, we found substantial genetic correlation between ANX and depression, neuroticism and other internalizing phenotypes. Follow-up analyses demonstrated enrichment in all major brain regions and highlighted GABAergic signaling as one potential mechanism implicated in ANX genetic risk. These results advance our understanding of the genetic architecture of ANX and prioritize genes for functional follow-up studies.

## Abstract (web summary)

Genome-wide association meta-analysis identifies 58 independent risk loci for major anxiety disorders among individuals of European ancestry and implicates GABAergic signaling as a potential mechanism underlying genetic risk for these disorders.

## Main

Fear and anxiety are critical survival responses; thus, ANX may result from dysregulation of the brain’s threat–response circuits. Although perturbations in various neurotransmitter systems, such as serotonin or gamma-aminobutyric acid (GABA), have been proposed as a basis of their etiology, no reliable biomarkers have yet been identified^1^. The major ANX, including generalized anxiety disorder (GAD), panic disorder and phobias (specific phobia, social phobia and agoraphobia), represent different clinical presentations of that underlying common diathesis^2–4^. Up to 25% of the population will develop an ANX at some point during their lifetime^5–7^. These disorders tend to onset early in life, are persistent and are highly comorbid with other psychiatric conditions for which they often present as a predisposing risk factor; for example, major depressive disorder (MDD) and substance-use disorders^6,8–10^. ANX are also associated with other medical conditions, such as neurological, cardiovascular and gastrointestinal disorders as well as cancers^11–14^. These features make ANX a leading source of worldwide disability^15,16^.

Each ANX aggregates in families (odds ratio, 4–6) primarily owing to genetic risk factors^17^. Estimates from twin studies indicate that ANX are moderately heritable (*h*^2^ = 30–50%)^2,17^, similar to other common psychiatric disorders like MDD but lower than less prevalent disorders like schizophrenia and bipolar disorder. Different ANX exhibit overlapping clinical features and strong comorbidity, which may be a result of shared genetic susceptibility^17–19^ and environmental risk factors^20–22^. Research implicates mechanisms that affect the structure and functional capacity of brain networks involved in emotion and cognition^23–25^. Twin studies report substantial genetic correlations between ANX and other psychiatric conditions, particularly MDD^26^, helping to explain their high comorbidity. In addition, ANX and depression both share genetic risk with heritable personality traits such as neuroticism^27,28^. Anxiety symptoms often precede suicidal behaviors^29^, with possible causal implications^30^. Therefore, examining the genetic relationship between ANX and related phenotypes on the internalizing spectrum is essential.

The combination of high prevalence, extensive comorbidity and high polygenicity makes it particularly difficult to identify genetic variants underlying risk for ANX. Prior genome-wide association studies (GWAS) have identified a handful of genetic loci with inconsistent results^31–36^. A recent meta-analysis using five publicly available datasets reported ten additional novel associations^37^. Genome-wide single nucleotide polymorphism (SNP)-based heritability estimates range from 10–28%, supporting that ANX have a polygenic basis. Consistent with twin studies, previous psychiatric GWAS have demonstrated that ANX polygenic risk is highly correlated with that of MDD and neuroticism^38–42^. Similar to other complex genetic phenotypes, sufficiently large samples are required to achieve the necessary power to detect the small effects of common variants.

Here, we present a GWAS meta-analysis from the Anxiety Disorders Working Group of the Psychiatric Genomics Consortium (PGC-ANX), consisting of 122,341 individuals diagnosed with any ANX and 729,881 controls, all of European (EUR) ancestry. We analyzed the data at the level of variant, gene, pathway/gene set and tissue by using both functionally informed and functionally agnostic methods. Subsequently, these results were compared with those of other phenotypes and investigated for possible molecular mechanisms and avenues for drug repurposing.

## Results

### GWAS meta-analysis

**Fig. 1:**
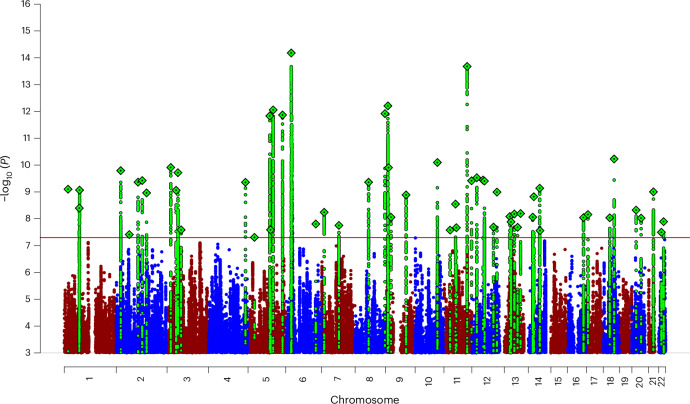
Manhattan plot of the main ANX GWAS showing 58 GWS loci. The *x* axis shows the position in the genome (chromosomes 1 to 22), and the *y* axis represents −log_10_(*P* values) (two-sided, not adjusted for multiple testing) for the association of variants with ANX using an inverse-variance weighted fixed effects model (122,341 ANX cases and 729,881 unaffected controls). The horizontal red line shows the threshold for GWS (*P* = 5 × 10^−8^). Dots represent each SNP that was tested in the GWAS, with a green diamond indicating the lead SNP of a GWS locus and green dots below representing SNPs within the locus with high levels of LD with the lead SNP.

We performed a GWAS meta-analysis of 36 case–control cohorts (122,341 ANX cases and 729,881 controls; Supplementary Table 1). Details about phenotype, quality control and GWAS analysis for each individual cohort are provided in Supplementary Note 2. Among the 7.2 million autosomal SNPs analyzed, we identified 58 independent, genome-wide significant (GWS) SNPs associated with ANX (Fig. 1 and Table 1; further information is provided in Supplementary Table 2, Supplementary Fig. 1 (quantile–quantile plot) and Supplementary Figs. 2–56 (regional association plots of each significant SNP and forest plots indicating each cohort’s effect size)). Estimates of the genomic inflation factor ($\lambda$ = 1.41, ${\lambda }_{1000}=1.00$), linkage disequilibrium (LD) score regression (LDSC) intercept (1.05, standard error (s.e.) = 0.01), and attenuation ratio (0.082, s.e. = 0.014) suggest that inflation was probably caused by polygenicity and not by cryptic population structure. LDSC estimates a SNP-based heritability of 10.1% (s.e. = 0.004), assuming a 20% population prevalence.

**Table 1:** List of the 58 independent GWS SNPs of the main ANX GWAS meta-analysis

Locus	Index SNP	CHR	Position (bp)	*P* value	OR	s.e.	A1/A2	Freq. cases	Freq. controls	Closest genes (distance kb)
1	rs34579341	1	72,745,962	4.01 × 10^−9^	0.964	0.006	G/A	0.18	0.17	*NEGR1*
2	rs11580539	1	73,896,218	8.55 × 10^−10^	0.971	0.005	G/A	0.60	0.61	*LINC01360* (−41.7)
3	rs5015511	2	22,546,852	1.61 × 10^−^^10^	0.032	0.005	A/G	0.52	0.54	–
4	rs79556790	2	63,480,537	3.87 × 10^−^^8^	0.881	0.023	A/C	0.98	0.98	*WDPCP*
5	rs7570682	2	104,983,267	4.31 × 10^−^^10^	0.036	0.006	A/G	0.23	0.23	*LOC100287010*
6	rs2165077	2	124,932,847	3.77 × 10^−^^10^	0.032	0.005	T/C	0.48	0.47	*CNTNAP5*
7	rs17407658	2	145,703,652	1.09 × 10^−^^9^	0.972	0.005	G/A	0.50	0.52	*TEX41*
8	rs9867083	3	18,804,734	1.22 × 10^−^^10^	0.034	0.005	C/T	0.70	0.69	*SATB1-AS1* (−183.1)
9	rs2888367	3	44,242,929	8.67 × 10^−^^10^	0.032	0.005	A/G	0.33	0.34	*TOPAZ1*
10	rs2710323	3	52,815,905	1.91 × 10^−^^10^	0.971	0.005	C/T	0.48	0.49	*NEK4*, *ITIH1*, *ITIH3*, *ITIH4*, *ITIH4-AS1*
11	rs4856929	3	68,030,736	2.59 × 10^−^^8^	0.047	0.008	T/C	0.87	0.88	*SUCLG2-AS1*, *TAFA1*
12	rs72704544	4	176,853,286	4.37 × 10^−^^10^	0.043	0.007	G/A	0.21	0.21	*GPM6A*
13	rs2066928	5	30,843,787	4.92 × 10^−^^8^	0.974	0.005	A/G	0.49	0.51	–
14	rs77960	5	103,964,585	1.47 × 10^−^^12^	0.037	0.005	A/G	0.32	0.31	–
15	rs288160	5	107,364,269	2.58 × 10^−^^8^	0.973	0.005	T/C	0.32	0.33	*FBXL17*
16	rs11241568	5	120,140,556	8.77 × 10^−^^13^	0.037	0.005	C/T	0.33	0.34	*PRR16* (−67.5)
17	rs10476497	5	164,588,817	1.36 × 10^−^^12^	0.034	0.005	A/G	0.54	0.55	–
18	rs58825580	6	26,365,679	6.64 × 10^−^^15^	0.943	0.008	G/T	0.12	0.11	*BTN3A2*, *BTN2A2*, *BTN3A1*
19	rs9373363	6	143,150,043	1.57 × 10^−^^8^	0.969	0.006	G/A	0.26	0.28	*HIVEP2*
20	rs12699332	7	12,269,762	5.75 × 10^−^^9^	0.028	0.005	T/G	0.41	0.39	*TMEM106B*
21	rs2371365	7	82,506,898	1.77 × 10^−^^8^	0.028	0.005	C/T	0.38	0.37	*PCLO*
22	rs4395923	8	65,569,387	4.34 × 10^−^^10^	0.031	0.005	A/G	0.59	0.61	*CYP7B1*
23	rs4976976	8	143,311,653	1.20 × 10^−^^12^	0.965	0.005	A/G	0.40	0.41	*LINC00051*, *TSNARE1*
24	rs10959883	9	11,519,984	6.21 × 10^−^^13^	0.959	0.006	C/T	0.20	0.20	–
25	rs10961649	9	14,670,949	1.24 × 10^−^^10^	0.033	0.005	T/C	0.32	0.31	*ZDHHC21*
26	rs13287777	9	26,719,411	8.74 × 10^−^^9^	0.960	0.007	T/G	0.18	0.18	–
27	rs28474857	9	98,247,204	1.29 × 10^−^^9^	0.048	0.008	T/C	0.10	0.11	*PTCH1*, *LOC100507346*
28	rs11599236	10	106,454,672	7.99 × 10^−^^11^	0.968	0.005	C/T	0.41	0.42	*SORCS3*, *SORCS3-AS1*
29	rs2071754	11	31,812,582	2.65 × 10^−^^8^	0.968	0.006	T/C	0.78	0.79	*ELP4*, *PAX6*, *PAX6-AS1*, *PAUPAR*
30	rs7121169	11	57,452,543	2.84 × 10^−^^9^	0.034	0.006	A/G	0.33	0.34	*MIR130A*, *YPEL4*, *CLP1*, *ZDHHC5*, *MED19*, *TMX2*, *TMX2-CTNND1*
31	rs174560	11	61,581,764	2.15 × 10^−^^8^	0.033	0.006	C/T	0.32	0.34	*TMEM258*, *MIR611*, *FEN1*, *FADS1*, *MIR1908*, *FADS2*
32	rs7110863	11	112,843,138	2.10 × 10^−^^14^	0.039	0.005	G/A	0.44	0.49	*LOC101928847*, *NCAM1*
33	rs73034295	11	133,822,133	3.84 × 10^−^^10^	0.963	0.006	A/G	0.22	0.24	*IGSF9B*
34	rs78120929	12	24,139,063	6.84 × 10^−^^10^	0.955	0.008	C/T	0.11	0.11	*SOX5*
35	rs989657	12	24,166,426	2.95 × 10^−^^10^	0.031	0.005	C/T	0.56	0.56	*SOX5*
36	rs61928096	12	53,780,633	3.60 × 10^−^^10^	0.100	0.015	A/G	0.04	0.03	*SP7*, *SP1*, *AMHR2*
37	rs4382947	12	60,475,057	3.94 × 10^−^^10^	0.969	0.005	A/G	0.42	0.41	–
38	rs6539062	12	103,552,910	2.04 × 10^−^^8^	0.027	0.005	A/C	0.51	0.54	*LOC101929058* (also known as *C12orf42-AS1*)
39	rs3847960	12	120,271,100	1.02 × 10^−^^9^	0.036	0.006	A/T	0.63	0.64	*CIT*
40	rs544271348	12	120,320,793	2.09 × 10^−^^8^	0.930	0.013	T/G	0.96	0.97	*CIT*
41	rs9534593	13	47,879,549	8.23 × 10^−^^9^	0.973	0.005	G/A	0.44	0.44	–
42	rs7997746	13	54,020,455	1.31 × 10^−^^8^	0.973	0.005	A/C	0.46	0.46	–
43	rs36119415	13	69,579,612	6.62 × 10^−^^9^	0.954	0.008	T/G	0.10	0.10	–
44	rs870764	13	84,973,006	2.08 × 10^−^^8^	0.031	0.006	A/G	0.73	0.74	*LINC00333* (non-coding)
45	rs9556979	13	99,241,507	6.38 × 10^−^^9^	0.032	0.005	G/T	0.32	0.32	*STK24*, *STK24-AS1*
46	rs61990288	14	42,074,726	8.70 × 10^−^^9^	0.973	0.005	A/G	0.50	0.49	*LRFN5*
47	rs3007061	14	47,238,606	1.51 × 10^−^^9^	0.031	0.005	C/T	0.62	0.63	–
48	rs12588874	14	75,254,073	7.26 × 10^−^^10^	0.029	0.005	A/G	0.53	0.51	*FCF1*, *YLPM1*
49	rs6574271	14	76,580,655	2.77 × 10^−^^8^	0.973	0.005	C/T	0.45	0.46	*IFT43*, *GPATCH2L*
50	rs616695	16	77,105,587	9.03 × 10^−^^9^	0.973	0.005	T/G	0.43	0.44	–
51	rs2289590	17	8,110,764	6.95 × 10^−^^9^	0.029	0.005	A/C	0.59	0.61	*VAMP2*, *TMEM107*, *SNORD118*, *MIR4521*, *BORCS6*, *AURKB*, *LINC00324*, *CTC1*, *PFAS*
52	rs8091977	18	31,359,414	9.18 × 10^−^^9^	0.029	0.005	C/T	0.46	0.47	*ASXL3*
53	rs4801024	18	52,396,321	5.90 × 10^−^^11^	0.038	0.006	G/T	0.75	0.74	*RAB27B* (49.4)
54	rs6047130	20	20,868,094	4.74 × 10^−^^9^	0.958	0.007	T/C	0.12	0.13	–
55	rs12624433	20	44,680,853	9.43 × 10^−^^9^	0.033	0.006	A/G	0.26	0.25	*MMP9*, *SLC12A5-AS1*, *SLC12A5*, *NCOA5*
56	rs2070865	21	40,715,519	9.93 × 10^−^^10^	0.972	0.005	T/C	0.47	0.50	*BRWD1*, *BRWD1-AS2*, *BRWD1-AS1*, *HMGN1*, *GET1*, *WRB-SH3BGR*
57	rs7290074	22	30,922,642	3.19 × 10^−^^8^	0.095	0.016	A/G	0.02	0.03	*SDC4P*, *SEC14L4*, *SEC14L6*, *GAL3ST1*, *PES1*
58	rs13056300	22	41,408,754	1.28 × 10^−^^8^	0.032	0.006	C/T	0.27	0.28	*RBX1*, *SNORD140* (10.9)

Index SNP, rs number of variant; CHR, chromosome; BP, base pair position (hg19); OR, odds ratio for allele 1; s.e., standard error; A1/A2, allele 1 and allele 2; Freq. cases, frequency of A1 in cases; Freq. controls, frequency of A1 in controls; Closest genes (distance kb), closest genes to the SNP with distance in kilobases in parentheses (if the SNP lies within the gene, no distance is given).

A series of sensitivity analyses, including GWAS Cochran’s *Q* (Supplementary Fig. 57) and *I*² statistics (forest plots in Supplementary Figs. 2–56), revealed no substantial genome-wide heterogeneity across the 36 cohorts. Furthermore, we performed subgroup-specific meta-analyses, subdividing our study cohorts based on (1) their ascertainment strategy (five subgroups: clinical, comorbidity, community, biobanks and self-reported professional diagnosis (SRPD); Manhattan and quantile–quantile plots in Supplementary Figs. 58–62) and (2) their assessment strategy (three subgroups: interview, ICD-10 codes and SRPD; Manhattan and quantile–quantile plots in Supplementary Figs. 63–66). We then used confirmatory factor analysis in GenomicSEM^43^ to test whether these subgroups fit a one-factor model. In both cases, a single latent factor best explained the genetic covariance between the subgroups (ascertainment fit statistics: CFI = 1, SRMR = 0.04; assessment fit statistics: CFI = 1, SRMR = 3.67 × 10^−9^). The factor loadings across both subgroup models were high (0.75–1), with the factor explaining 81.8% and 95.6% of the total genomic variance in the ascertainment and assessment models, respectively (see Supplementary Note 3 for details on the subgrouping and Supplementary Table 5 and Supplementary Fig. 67a,b for GenomicSEM results). Using parallel analysis based on multivariate LDSC (paLDSC^44^), we identified one non-spurious dimension in exploratory genomic factor analysis, including 14 cohorts with more than 10,000 individuals and at least 1,000 cases. This finding supports our hypothesis that the genetic association signals were generally consistent across samples and study designs and tapped into a common underlying ANX genetic vulnerability.

### Replication and validation of GWAS SNPs

We conducted two replication analyses of the 58 significant loci: one in a large independent EUR ANX GWAS from 23andMe, and the other in an African-American (AFR) ancestry ANX GWAS from the Veterans Affairs Million Veteran Program (MVP). The 23andMe sample consisted of 1,175,012 ANX self-report cases and 1,956,379 controls (see Methods for details). Among the 58 SNPs identified in the discovery GWAS, all but one (rs7121169) were available for replication testing in the 23andMe genotype platform. Two additional variants failed quality control procedures (rs72704544 and rs11599236). Considering the remaining 55 loci tested, all showed the same direction of effect as the primary GWAS, and 51 were significant at a Bonferroni-corrected *P* value of *P* = 0.0009 (0.05 / 55) (Supplementary Table 6). At the time of this analysis, only the MVP had published an ANX GWAS in a reasonably sized non-EUR sample (MVP-AFR: military ascertainment, AFR ancestry; 5,664 cases and 26,410 controls)^34^. Analyzing those data, we compared the direction of effect and *P* values of association for our 58 lead SNPs to examine consistency with our EUR results (Supplementary Table 7). Among the 53 SNPs available in MVP-AFR, only 27 (50.9%) showed the same sign. Given differences in LD and allele frequency between EUR and AFR genomes, we also searched for the most significant SNP in a 50-kb window around each lead SNP in the MVP-AFR cohort. A total of 36 of these SNPs were nominally associated, but only two were significantly associated after adjustment for multiple testing.

We further compared our associations with those reported in previous ANX case–control GWAS^31–34,37^ (Supplementary Table 8). A recent GWAS using broader anxiety-related case–control and symptom-based phenotypes reported 40 EUR-ancestry significant SNPs^45^; all but one showed the same direction of effect, while ten were also GWS in our analysis. Importantly, most of the associations in our GWAS are novel discoveries, with only 15 reported in prior ANX GWAS. We note that some of the previously identified SNPs are in LD with each other, and all previously published ANX GWAS partially overlap with our samples. Therefore, these are not independent replications but demonstrate the consistency of results when additional samples are incorporated.

To study the generalizability of our results across different ancestral groups, we tested the extent to which polygenic risk scores (PRS) derived from our GWAS (excluding UK datasets) predicted ANX in the UK Biobank for participants of EUR, AFR and South Asian ancestry (see Supplementary Table 9). The PRS predicted 2.27% of the variance (*P* < 2.0 × 10^−16^) in ANX liability for those of EUR ancestry, assuming a prevalence of 20%. The variance explained for those of South Asian and AFR ancestries was 1.94% (*P* = 6.37 × 10^−5^) and 0.54% (*P* = 0.051), respectively, revealing significant polygenic overlap across EUR and South Asian ancestries.

### Characterization and functional annotation of GWAS SNPs

To identify potential causal variants, we conducted statistical fine mapping of our GWS loci using FINEMAP (v.1.3.1) with stringent inclusion thresholds^46^. This process identified six credible SNP sets defined as having a posterior probability of >0.95 and five or fewer SNPs per credible set to avoid excessive false positive rates (Supplementary Table 10). The lead SNPs of these credible sets were located at the following chromosomal positions: 3:67,895,104 (within *SUCLG2-GT*), 10:104,654,873 (within *SORCS3*), 17:8,187,590 (near *TRI-AAT-5*) and 20:20,876,379 (near *KIZ*); and two within the major histocompatibility complex (MHC) region: 6:28,329,086 (within *ZSCAN31*) and 6:30,170,699 (within *TRIM15*).

To examine the biological relevance of our GWS SNPs, we performed functional annotation in FUMA (v.1.6.1) to link our GWS SNPs with expression quantitative trait loci (eQTL) and brain chromatin interaction (Hi-C) data. The results suggest that most of the identified loci were associated with established gene regulatory mechanisms (circos plots in Supplementary Figs. 68–87). Although these results on their own do not provide enough evidence for involvement of respective genes in the etiology of ANX, they add to a broader picture that includes our summary-data-based Mendelian randomization (SMR) and other analyses (Supplementary Table 20).

We conducted stratified LDSC to partition the heritability into different functional genetic annotations and cell types. As noted in Supplementary Table 11, the association signal is highly conserved across species and significantly enriched for introns, monomethylated and polyacetylated histone marks (H3K4me1 and H3K4ac) and DNase I hypersensitivity sites in both adult and fetal tissues. Similar to other psychiatric GWAS, our findings are enriched for certain non-coding features rather than coding regions. Cell-type-specific enrichment was observed for central nervous system structures, including multiple cortical and subcortical areas, as well as cervical spine.

We also examined whether genetic associations with ANX were enriched among transcriptomic profiles of human tissues and/or individual cell types, using FUMA (v1.6.1)^47^. Tissue-enrichment analyses for general tissue types using data from the GTEx (v.8) consortium suggested that the expression patterns related to brain and pituitary tissues were significantly associated with the genetic risk of ANX (*P* = 1.18 × 10^−13^ and *P* = 6.50 × 10^−5^, respectively; Supplementary Table 12a and Supplementary Fig. 88). All individual brain tissues showed significant enrichment (Supplementary Table 12b and Supplementary Fig. 89), with cortex overall (*P* = 2.62 × 10^−12^) as well as frontal and anterior cingulate cortices and nucleus accumbens as most significant. At the level of individual cell types, we found a consistent association of GABAergic neurons with genetic variation associated with ANX (Supplementary Fig. 90). Our strongest association (*P* = 3.24 × 10^−8^) was found with GABAergic neuroblasts (via GSE76381)^48^.

### Gene-based association and enrichment

Using MAGMA (v.1.08)^49^, we identified 91 significantly associated genes (adjusted *P* < 0.05 / 18,490 = 2.7 × 10^−6^; Supplementary Table 13). Historically interesting candidates include *CLOCK*, *GABBR1*, *PCLO*, *NCAM1* and *DRD2*.

To test whether our loci significantly co-localize with known functional QTLs, we used SMR^50^ to conduct transcriptome-wide, proteome-wide and methylome-wide analyses (T-SMR, P-SMR and M-SMR, respectively). We used the largest available eQTL, protein QTL and methylation QTL reference datasets, respectively, for both brain and blood tissues (Supplementary Table 14). By using the conservative *P* values adjusted for the HEIDI test (see Methods), we detected 27 Bonferroni-corrected significant genes or isoforms in the brain associated with changes in the methylome, 16 in the transcriptome and seven in the proteome (Supplementary Tables 15–17). To improve signal detection in brain transcriptome and methylome data, we used Primo^51^ to jointly analyze blood and brain statistics (see ref. ^52^). We did not jointly analyze proteome data because of the low number of brain probes. These between-tissue concordance analyses yielded 22 significant ANX signals (posterior probability of >0.95) for the transcriptome and 133 for the methylome (Supplementary Tables 18 and 19). *BTN3A2* remains a leading signal in both analyses, and interesting sub-threshold genes from single-tissue analyses become strong findings in the joint T-SMR (*ZDHHC5*, *FURIN* and *NEGR1)*.

**Fig. 2:**
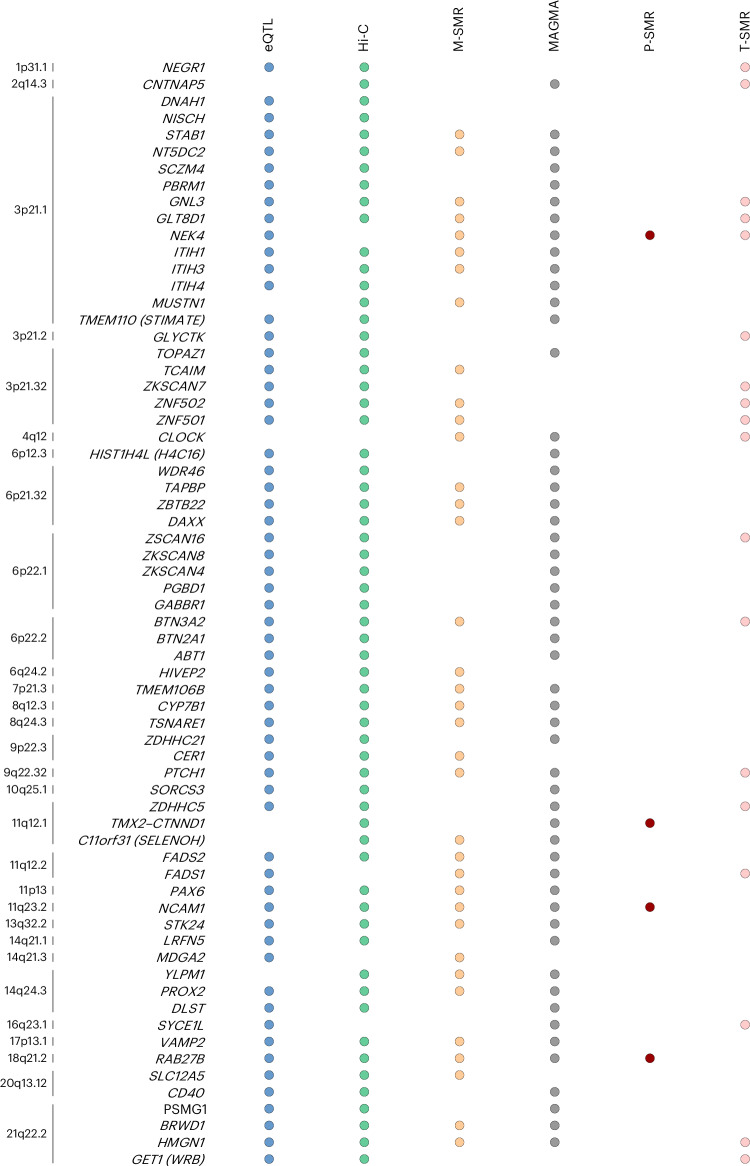
List of 66 most highly supported ANX genes. Genes that were implicated in at least three of the six SNP-based (eQTL, Hi-C) or gene-based (MAGMA, M-SMR, P-SMR, T-SMR) tests. The left side indicates the position of the gene in the genome. Significance is indicated by a colored dot. eQTL (blue dots) compares results from brain-related eQTL studies for overlap in significance between our GWAS and the eQTL studies. Hi-C (green dots) uses brain-related Hi-C information available through FUMA to functionally annotate our results. MAGMA (gray dots) tests genetic associations at the gene level for the combined effect of SNPs in or near protein-coding genes. M-SMR, P-SMR and T-SMR (yellow, red and pink dots, respectively) refer to transcriptome-wide, proteome-wide and methylome-wide analyses that assessed likely causal associations between traits and genes, proteins and genomic regions by inferring the association between the trait and gene expression, protein concentration and methylation, as predicted from genomic data.

To highlight genes for which there was the strongest support, we summarized the findings across multiple (equally weighted) analyses in Supplementary Table 20, which includes an expanded set of 151 genes associated with ANX susceptibility. Starting with the 91 significant associations from MAGMA, we added genes supported by joint T-SMR or joint M-SMR with a posterior probability of >0.95. We annotated these using additional support from P-SMR, eQTL and Hi-C data. Figure 2 lists the 66 genes with three or more sources of support (score of ≥3). Most of these have prior reported associations with one or more psychiatric phenotypes, possibly suggesting gene-based pleiotropy, while a small proportion appear specific to ANX risk (reviewed in the Discussion).

To test whether pre-existing gene sets are enriched for our ANX risk loci, we examined 10,894 gene sets obtained from MsigDB (v.5.2) (curated gene sets, 4,728; Gene Ontology terms, 6,166). Specifically, we used MAGMA to test for enrichment of our ANX signals (see Supplementary Table 21). Overall, one gene set was significant after correction for multiple testing: dawson_methylated_in_lymphoma_tcl1 (*P* = 1.71 × 10^−6^), including 57 genes that are hypermethylated in at least one of the lymphoma tumors in transgenic mice overexpressing *TCL1* in germinal center B lymphocytes; the top three genes were also supported by T-SMR or M-SMR (*NCAM1*, *HMGN1* and *ZDHHC5*). On the surface, it is difficult to appreciate the relevance of this cancer gene pathway for anxiety etiology. We also note that the overlap between this gene set and MAGMA gene signals is small (three out of 54; namely, *NCAM1*, *HMGN1* and *ZDHHC5*). Among the next highly associated sets were genes related to commissural neuron axon guidance (*P* = 5.24 × 10^−5^) and GABAergic synapse (*P* = 9.67 × 10^−5^), the latter with 66 genes, including *GABBR1*, *DRD2*, *CDH13* and *LRFN5*.

### Gene–drug associations

To reveal possible drug repurposing opportunities for ANX, we used DrugTargetor^53^ (v.1.3) with our main ANX summary statistics. Among the 161 drug classes analyzed, several that are already successfully being used for ANX treatment demonstrated significant associations (*q* value_BF_ < 0.05; Supplementary Table 22): psycholeptics (drugs with a calming effect) and psychoanaleptics (mostly antidepressants), as well as other sedating drugs like antihistamines, antipsychotics, general anesthetics and opioids. However, none of the more than 1,500 individual compounds cataloged in ChEMBL^54^ and DgiDB^55^ yielded a significant signal (Supplementary Table 23), possibly because of the moderate power of this GWAS.

### Genetic overlap between ANX and other phenotypes

**Fig. 3:**
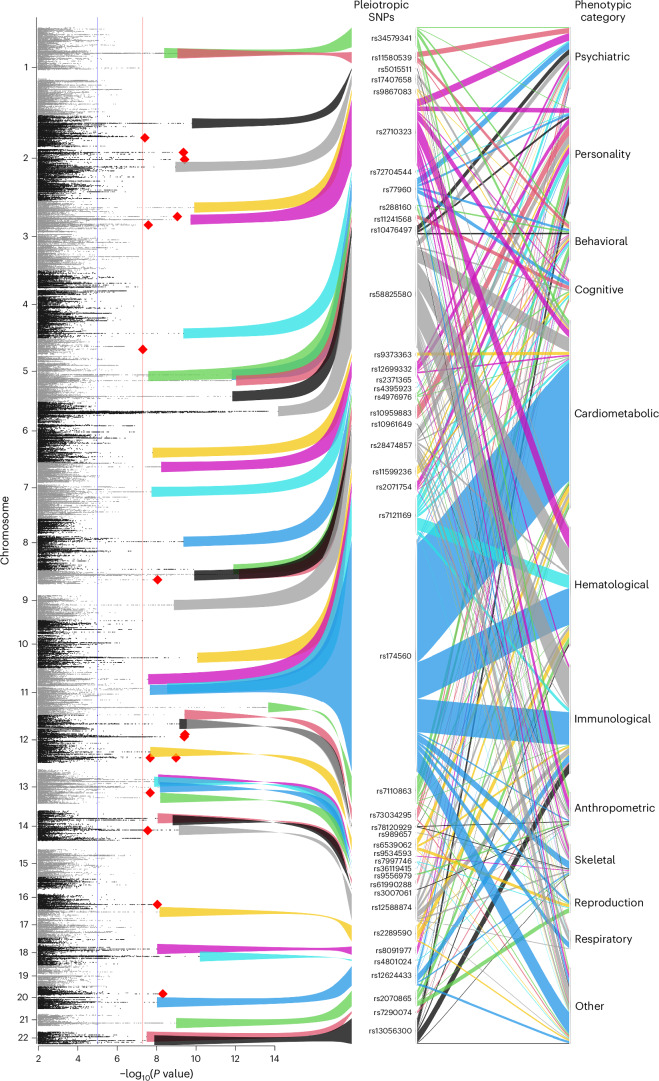
Overview of SNP associations with other phenotypes. The (rotated) Manhattan plot of the −log_10_(*P* values) of the ANX meta-analysis (left; as in Fig. 1) and PheWAS alluvial plot of potentially pleiotropic variants (right). The colored ribbons depict variants that are associated with at least one other published GWAS finding and correspond with the color of the ribbon in the alluvial plot. The red diamonds in the Manhattan plot depict the most significant variant in the region corresponding with potentially ANX-specific SNPs; that is, a variant that reached the GWS threshold for ANX but not in any other published GWAS.

**Fig. 4:**
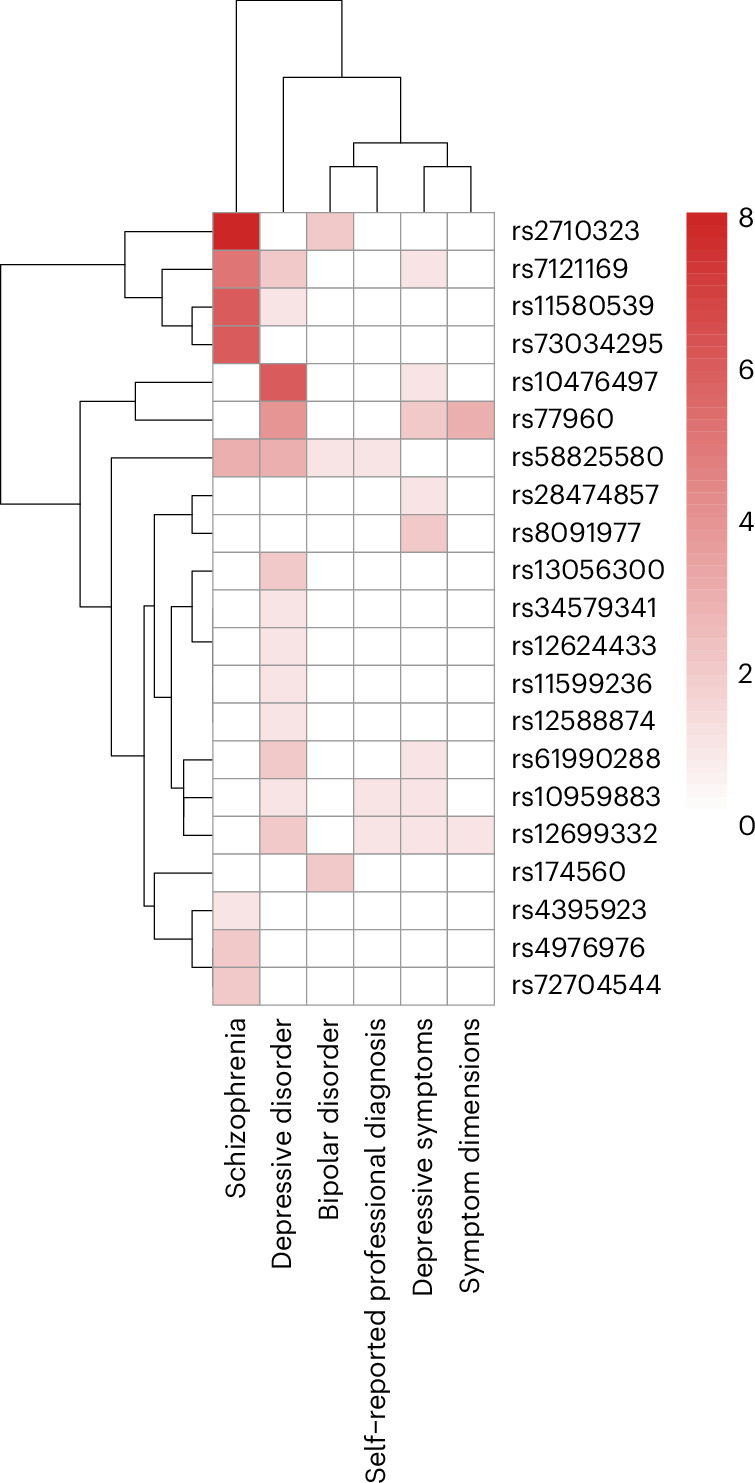
Heatmap of SNP associations with other psychiatric and personality traits. Dendrogram-based heatmap indicating the number of unique GWS associations with psychiatric or personality traits among 24 SNPs that reach significance for multiple such phenotypes. Shading indicates the number of GWAS reporting associations between a specific SNP and the outcomes. Symptom dimensions (mood disturbance, mania, psychosis) and self-reported professional diagnoses (depression, anxiety, distress) are from the UK Biobank.

To examine the overlap between our ANX association signals and other phenotypes, we conducted a phenome-wide association study (PheWAS). Of the 58 SNPs significantly associated with ANX, 15 were deemed ANX-specific (red diamonds in Fig. 3); that is, variants not reported as GWS in other extant GWAS. A total of 43 variants were associated with at least one other phenotype. We note that the higher number of overlapping associations with cardiometabolic, hematological and immunological outcomes reflects both the robust genetic architectures of these phenotypes and the number of GWAS that have been published in these domains. Overlap of ANX-related SNPs with cardiometabolic and hematological traits was heavily skewed towards a subset of variants (rs2710323, rs58825580 and rs174560). Figure 4 depicts a dendrogram-based heatmap showing the association with psychiatric or personality traits among 24 possibly pleiotropic SNPs (other heatmaps for cognitive and behavioral domains are found in Supplementary Figs. 91 and 92). Not surprisingly, more ANX SNPs overlap with internalizing phenotypes (neuroticism, depression) than with psychotic disorders (schizophrenia, bipolar disorder).

**Fig. 5:**
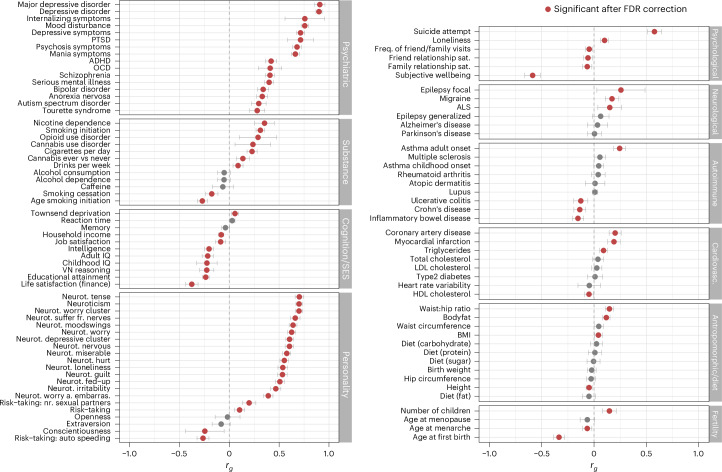
Genetic correlations (*r*_*G*_) between the main ANX GWAS and 112 phenotypes. Genetic correlations (*r*_*g*_) between ANX and psychiatric, substance use, cognition/socioeconomic status (SES), personality, psychological, neurological, autoimmune, cardiovascular, anthropomorphic/diet, fertility and other phenotypes. References and sample sizes of the corresponding summary statistics of the GWAS studies can be found in Supplementary Table 24. The ANX summary statistics are of the main meta-analysis (*n*_cases_ = 122,341; *n*_controls_ = 729,881). Red circles indicate significant associations with a *P* value adjusted for multiple testing with the Benjamini–Hochberg procedure to control the false discovery rate (FDR < 0.05). Black circles indicate associations that are not significant. Error bars represent 95% confidence intervals for the genetic correlation estimates. ADHD, attention-deficit hyperactivity disorder; ALS, amyotrophic lateral sclerosis; BMI, body mass index; embarras., embarrassment; freq., frequency; fr., from; HDL, high-density lipoprotein; LDL, low-density lipoprotein; neurot., neuroticism; nr., number; OCD, obsessive–compulsive disorder; sat., satisfaction.

We used bivariate LDSC to estimate the genetic correlations between ANX and a wide variety of other traits. We included 112 previously published GWAS on various traits, including psychiatric, substance use, cognition or socioeconomic status, personality, psychological, neurological, autoimmune, cardiovascular, anthropomorphic, dietary and fertility phenotypes. After false discovery rate correction, we found that 82 traits showed significant genetic correlation with ANX (Fig. 5 and Supplementary Table 24). Among the psychiatric disorders and traits, ANX showed the strongest correlations with MDD ($${r}_{g}=0.91)$$, followed by childhood internalizing symptoms $${(r}_{g}=0.76)$$, mood disturbance $${(r}_{g}=0.76)$$, symptoms of depression $${(r}_{g}=0.71)$$, post-traumatic stress disorder (PTSD) $${(r}_{g}=0.71)$$, psychosis $${(r}_{g}=0.68)$$, mania $${(r}_{g}=0.66)$$, suicide attempt $${(r}_{g}=0.58)$$ and obsessive–compulsive disorder $${(r}_{g}=0.41)$$. Genetic correlations were also high with total neuroticism score $${(r}_{g}=0.70)$$ and its various clusters and items. We found somewhat lower correlations with other psychiatric and substance-use disorders. ANX genetic risk was also modestly correlated with that of several neurological disorders, as well as adult-onset asthma and heart disease (positive) and inflammatory bowel diseases (negative). As shown in Supplementary Figs. 93 and 94 and Supplementary Table 24, the different ANX data subgroups show a variable but overall similar pattern of correlations.

These results highlight the complex interrelations between the three internalizing phenotypes that also have the highest genetic correlations with ANX: MDD^56^, PTSD^57^ and neuroticism^39^. To examine potential directional effects underlying these correlations, we applied bi-directional generalized SMR (GSMR)^58^ with the latest available GWAS summary statistics. These results (Supplementary Table 25) indicate a highly significant bi-directional effect between ANX and each of these phenotypes. Based on beta-values, the strength of reverse (MDD → ANX = 0.657) and forward (ANX → MDD = 0.545) effects are similar between ANX and MDD. However, both PTSD (PTSD → ANX = 0.891 vs ANX → PTSD = 0.239) and neuroticism (neuroticism → ANX = 1.25 vs ANX → neuroticism = 0.17) effects on ANX are stronger than the reverse.

## Discussion

In this GWAS meta-analysis, we identified 58 independent genome-wide loci associated with anxiety risk by including data from a composite phenotype created from five lifetime anxiety disorders (36 cohorts including 122,341 ANX cases and 729,881 controls; *n*_effective_ = 390,560). Three-quarters of the identified variants are novel, with only 15 reported in prior anxiety GWAS. A total of 51 of these SNPs were replicated in an independent EUR-ancestry sample from 23andMe, strengthening their relevance. These results represent a major advance in identifying validated susceptibility loci for anxiety disorders.

The SNP-based heritability estimated at 10.1% captures approximately one-quarter of the broad-sense heritability from twin studies of adult ANX^17^, similar to other complex traits like MDD^40^. We divided the cohorts into subgroups based on ascertainment and assessment strategies and conducted separate GWAS as a sensitivity test. We observed moderate to high genetic correlations between these subgroups, supporting our decision to combine all samples into a single meta-analysis. SNP-based heritability varied from 23.7% in the clinical subgroup to 6.9% in the community subgroup (ascertainment) and from 7.7% in the interview subgroup to 13.2% in the ICD-10 subgroup (assessment), consistent with the hypothesis that more severe syndromes have higher heritability^59–61^. The overall meta-analytic SNP heritability is probably diminished by the effects of heterogeneity across these subgroups.

Along with replication in an independent EUR cohort from 23andMe (51 loci replicated at a Bonferroni-corrected *P* value), we tested the transferability of our results. First, we examined replication in the MVP-AFR ancestry sample, in which nominally significant proxy loci were identified for 36 lead SNPs, but only two showed significant association after Bonferroni adjustment. This is not surprising given both ancestry and ascertainment differences. Second, we applied PRS to estimate the variance explained in ANX liability. The PRS explained 2.27% of the variance in EUR individuals, which is comparable to PRS reports of MDD^40^. We then tested whether our findings would generalize to non-EUR samples. The EUR-ANX PRS explained 1.94% of the variance in the South Asian subsample of UK Biobank (significant) but only 0.54% for the AFR subsample (non-significant), in line with the low replication in the MVP-AFR ancestry cohort. This shows that for anxiety, as for other phenotypes, genetic liability estimated from EUR samples more closely reflects that of South Asian than AFR ancestry^31^. These findings stress the need for more diverse ancestry inclusion in future ANX GWAS.

Using LDSC, we found that, consistent with prior twin studies and extant GWAS, ANX shares the largest genetic overlap with MDD (*r*_*g*_ = 0.91), with which it has the highest lifetime comorbidity. This is followed by PTSD (*r*_*g*_ = 0.71), which is expected given their high comorbidity and the prior classification of PTSD among anxiety disorders^62^; however, this correlation is over twice that estimated in an early twin study^28^. The genetic correlation with neuroticism was similarly high (*r*_*g*_ = 0.7), reflecting that neuroticism is an important predisposing personality trait for both ANX and MDD. In addition, ANX shows moderate genetic correlations with ADHD (*r*_*g*_ = 0.42), obsessive–compulsive disorder (*r*_*g*_ = 0.41), schizophrenia (*r*_*g*_ = 0.41), bipolar disorder (*r*_*g*_ = 0.34) and anorexia nervosa (*r*_*g*_ = 0.33). ANX also correlates with childhood internalizing symptoms (*r*_*g*_ = 0.76), reflecting genetic continuity across development^63,64^. Noteably, ANX shows a substantial genetic correlation with suicide attempt (*r*_*g*_ = 0.58). This may be partly driven by comorbid depression, although ANX also independently increases suicide risk^65^.

Follow-up Mendelian randomization (MR) analyses suggest bi-directional genetic effects between ANX and its strongest correlates: MDD, PTSD and neuroticism. Although ANX onset tends to precede MDD^66,67^, some studies show mutual prediction over time^68,69^. Our MR analyses support a stronger genetic causation of neuroticism on ANX, reflecting the stability of this personality trait^70^ and its persistent relationship with psychiatric disorders^71^. Unexpectedly, MR suggests that PTSD is more likely to cause ANX, potentially owing to confounding (for example, diagnostic misclassification), ascertainment bias (PTSD presents with more severe symptoms) or because trauma can impact both disorders. These findings align with clinical experience that comorbid internalizing disorders exacerbate each other.

Gene-set and single-cell RNA expression analyses support GABAergic signaling as one potential mechanism underlying ANX genetic risk, supported by the efficacy of drugs like barbiturates and benzodiazepines in enhancing GABA neurotransmission. Indeed, the results of our gene–drug analysis included several classes of drugs that are already successfully used to relieve anxiety.

The PheWAS revealed that 43 SNPs identified in prior GWAS of other phenotypes overlap with ANX, highlighting extensive genetic sharing. The loci clustered into three categories: those affecting multiple medical, physiological and behavioral outcomes; those linked to psychiatric and behavioral phenotypes; and a small set specific to anxiety. Given the high comorbidity and genetic overlap of ANX with phenotypes like MDD or neuroticism, it is unsurprising that many of our loci have been reported in prior GWAS. However, most prior psychiatric GWAS did not exclude ANX, which may have influenced their findings. Notably, several loci—including four genes (*PAX6*, *PROX2*, *VAMP2* and *HMGN1*)*—*show strong evidence of association in our study but have not been reported in prior psychiatric GWAS (further discussed in Supplementary Note 4).

Seven of the 66 protein-coding genes associated with ANX risk (*ZNF502*, *ZNF501*, *STAB1*, *NT5DC2*, *GNL3*, *GLT8D1* and *NEK4*) are located on chromosome 3p21, a region previously linked to depression^56^, schizophrenia^72^, bipolar disorder^73^, suicide^74^, amyotrophic lateral sclerosis^75^ and neuroticism^39^, making it a ‘hot spot’ for overall neuropsychiatric susceptibility. Although little is known about these seven genes in addition to their basic cellular functions, some are implicated in anxiety-like behaviors in rodents^76^. Three genes (*TAPBP*, *ZBTB22* and *DAXX*) of the MHC region (chromosomal band 6p21.32) were also associated with ANX. These findings do not represent a definitive set of anxiety risk genes but instead provide a high-level summary of findings from multiple post-GWAS approaches, serving as a starting point for future studies.

Given similarly high lifetime prevalence, moderate twin-based heritability and extensive comorbidity, our ANX genetic results should be most comparable to those for MDD among all psychiatric diagnoses. Indeed, the authors of a previous publication^40^ describe results from their PGC-MDD2 analyses that are highly similar to ours regarding the number of GWS SNPs identified per effective sample size, SNP-based heritability, enrichment of non-exonic classes of variants and proportion of variance explained by PRS. These highly polygenic internalizing disorders require massive sample sizes to detect association signals from the small effects of many common SNPs. From what we have learned about MDD and other complex psychiatric phenotypes, the 58 loci we report herein are probably ‘the tip of the iceberg’ among the many hundreds of loci presumed to underlie individual differences in ANX risk. Therefore, further genomic discovery efforts for ANX will demand even larger sample sizes.

This study has several potential limitations. First, heterogeneity in ANX case phenotype assessments—from structured psychiatric interviews to ICD clinical assignments to self-report diagnoses—limits the validity and power to detect susceptibility variants. There is often a trade-off between clinical validity and sample size^61,77^, as seen in our largest samples, which had the lowest depth of phenotyping. Second, by collapsing across all five of the adult anxiety diagnoses, we increased phenotypic heterogeneity, making it impossible to pinpoint the genetic signals specific to any particular disorder. Future studies with large, well-phenotyped samples of individual diagnoses are needed to address this limitation. Additionally, genetic contributions to ANX may change over the lifespan, highlighting the importance of longitudinal studies. We allowed comorbid mood disorders in ANX cases but excluded them from controls. Although this was justifiable because of the strong genetic sharing between ANX and depression, it could indirectly inflate their genetic associations and complicate inferences of pleiotropy. Finally, limiting our meta-analysis to EUR data reduces generalizability. We are working to aggregate data across ancestries for future multi-ancestry GWAS.

In summary, this study advances our understanding of the genetic basis of ANX by providing a foundation for future research into the biological mechanisms behind anxiety syndromes. It is our sincere hope that this opens new lines of investigation for expanding the clinical armamentarium of the next generation of clinicians who treat individuals affected by these conditions.

## Methods

### Ethics

All relevant ethics approvals have been obtained by the respective cohort’s institutions, and a list of all respective approvals can be found in Supplementary Note 1.

### Samples

To maximize sample size and power, we assigned the composite Any Anxiety case status if a participant had at least one of five core adult ANX across their lifetime: GAD, panic disorder, social phobia, agoraphobia or specific phobias. This amounts to identifying common genetic effects shared across these disorders. We did not exclude comorbid mood or other anxiety-related disorders in the cases. Controls had no lifetime anxiety disorder. Owing to the genetic overlap between ANX and depression^78,79^, we excluded controls if they had a lifetime comorbid mood disorder like MDD or bipolar disorder. We excluded individuals with a diagnosis of severe mental health conditions such as schizophrenia, autism or intellectual disability. As much as possible, we uniformly applied these criteria across the 36 samples included in this study (Supplementary Table 1). However, like most large-scale psychiatric GWAS, these samples were ascertained and assessed with variable approaches that introduce known and cryptic sources of heterogeneity (see Supplementary Note 2 for details of each study). With the aim to address phenotypic heterogeneity, we classified each of the 36 cohorts into five ascertainment subgroups (clinical, biobank, community, SRPD and comorbid) and three assessment subgroups (interview, ICD-10, biobank); see Supplementary Table 1 and Supplementary Note 3.

Our subsequent analyses fall into six categories, which are described in detail below. These include (1) core GWAS, SNP heritability and sensitivity analyses including differences between ascertainment and assessment groups; (2) replication and validation of the GWAS SNPs; (3) characterization and functional annotation of the significant SNPs, (4) gene-based associations and enrichment; (5) gene–drug associations; and (6) genetic associations and pleiotropy shared with other traits.

### GWAS, SNP-based heritability and sensitivity analyses

#### Genetic data processing and individual GWAS analyses

Each dataset was imputed using either the Haplotype Reference Consortium^80^ or the 1000 Genomes Project Phase 3 (ref. ^81^) reference panels, and a GWAS was conducted for each (Supplementary Note 2 for details). The results from the individual GWAS were then harmonized and transformed to ‘daner’ file format following Rapid Imputation and COmpuational PIpeLIne for GWAS (RICOPILI)^82^ specifications. Details of harmonization, alignment and filtering can be found at the end of Supplementary Note 2. Sumstats further used DENTIST as a quality control measure^83^.

#### GWAS meta-analysis

The GWAS meta-analysis was performed on over 7.2 million autosomal SNPs across the 36 cohorts using inverse-variance weighting in METAL^84^ within RICOPILI. Heterogeneity between the studies was evaluated using Cochran’s *Q* and *I*² statistics (see Supplementary Note 2). To distinguish polygenicity from other causes of genomic inflation, we calculated the LDSC^85^ intercept using the summary statistics for the high-quality common SNPs (INFO score of >0.9) from the meta-analysis. The GWS threshold for association was set at $P < 5\times {10}^{-8}$. Automated LD-based ‘clumping’ of GWS SNPs was conducted in RICOPILI using PLINK to facilitate identification of independently associated loci. We defined LD-independent SNPs as those with low LD (*r*^2^ < 0.1) to a more significantly associated SNP within a 500-kb window. When loci contained several significant SNPs, the SNP with the lowest *P* value in each locus was selected as the lead SNP reported here. In addition to the main meta-analysis, we meta-analyzed similar datasets together according to the subgroup assignments described above.

#### Internal consistency of the ANX phenotype—sensitivity analyses of ANX ascertainment and assessment subgroups

##### SNP-based heritability estimation and genetic correlations

We used LDSC^86^ to calculate the SNP-based heritability of the overall meta-analysis and the subgroup meta-analyses. Additionally, we used cross-trait LDSC to compute pairwise genetic correlations among the subgroups. SNP-based heritability was estimated from the slope of the LDSC on the liability scale, assuming a 20% population prevalence of ANX. To avoid a downward bias in our liability scale heritability estimates, the effective sample size across the contributing cohorts was calculated and used as the input sample size for LDSC^87^. The sample prevalence was then specified as 0.5 for the conversion to the liability scale. Genetic correlation is calculated by estimating the slope from regressing the product of the *Z*-scores from two separate GWAS onto the LD score. It reflects the genetic covariation between two traits that is captured by all SNPs included in the GWAS. For both heritability estimation and genetic correlation analysis, we used pre-calculated LD scores from samples of EUR in the 1000 Genomes Project, which were filtered for SNPs present in the HapMap3 reference panel.

##### paLDSC

The paLDSC function^44^ in GenomicSEM was used to determine the number of non-spurious dimensions in exploratory genomic factor analysis. This is achieved by comparing the eigenvalues obtained from the eigendecomposition of the LDSC genetic correlation matrix to those derived from a Monte Carlo-simulated null correlation matrix, whereby random noise is drawn from the multivariate LDSC sampling distribution. The suggested number of factors to be extracted corresponds with an eigenvalue exceeding a pre-specified percentile from the corresponding distribution of eigenvalues generated under the null.

##### GenomicSEM 1-factor model

To extend the genetic correlation analysis, we used genomic structural equation modeling (GenomicSEM)^43^ to model the genetic architecture of the ascertainment and assessment subgroups. We conducted an exploratory factor analysis first, followed by a confirmatory factor analysis. To conduct these analyses, first, the summary statistics were harmonized and filtered (with the munge-function) using HapMap3 as the reference file, with the effective sample size as the input sample size and SNPs filtered to INFO > 0.9 and MAF > 0.01. Second, multivariable LDSC was run to obtain the genetic covariance matrix and corresponding sampling covariance matrix using pre-computed EUR-ancestry LD scores. Third, we conducted exploratory factor analysis followed by confirmatory factor analysis using the pre-packaged common factor model in GenomicSEM using diagonally weighted least squares estimation.

### Replication and validation of GWAS SNPs

#### Replications

Lead SNPs from the primary GWAS were tested for replication in the 23andMe commercial database using 1,175,012 self-reported ANX cases and 1,956,379 controls. Self-reported ANX cases were individuals who checked ‘anxiety’ in response to either of the following survey questions: “Have you ever been diagnosed with any of the following…” or “What mental health problems have you had? Please check all that apply”. This GWAS excluded close relatives (excluded cases, 13,801; excluded controls, 21,454) and an additional 35,255 samples (1.1%) because of consent restrictions (as of June 9, 2023). We performed logistic regression, assuming an additive model for allelic effects after covarying for age, sex, the first five principal components and genotyping platform. Previous work has demonstrated that the first five principal components in the 23andMe dataset explain more variance than the first ten principal components from the UK BioBank^86^. The *P* values were adjusted using the standard genomic control procedure^88^ in which the chi-squared test statistic is divided by the genome-wide estimated lambda inflation factor, λ = 1.491 (s.e. = 0.024). The estimated SNP heritability was *h*^2^ = 0.088 (s.e. = 0.002), consistent with the estimate from our discovery GWAS.

Furthermore, we conducted a replication analysis of our 58 ANX-associated SNPs in an independent AFR sample from MVP comprising 5,664 ANX cases and 26,410 controls. Initially, we assessed the association results of the same 58 SNPs that reached significance in our main EUR-ancestry GWAS. Recognizing that the lead SNP might not necessarily be the causal SNP in this region and considering the differing LD structures between the EUR and AFR ancestry groups, we anticipated that the same SNP might not exhibit significant association. However, the genomic region might still be associated in AFR samples. Therefore, we performed a second look-up to identify the most significant SNP within a 50-kb window (±25 kb) to accommodate potential differences in LD across EUR and AFR ancestries (proxy loci). LD between AFR and EUR populations was evaluated using *r*² and *D*’ metrics (as reported on https://ldlink.nih.gov). We considered replication significant at a Bonferroni-corrected significance threshold of 8.62 × 10^−4^ (0.05 / 58).

To evaluate the consistency of previously reported ANX-associated SNPs, we performed a look-up of those SNPs in our main GWAS meta-analysis. We restricted the look-up to prior findings from case–control GWAS (as opposed to dimensional, symptom-based GWAS). Of note is that none of the previously published ANX GWAS are completely independent of our sample but are partially overlapping.

#### PRS analyses

We validated our results with PRS analyses in independent UK Biobank samples after removing all UK-based samples (UK Biobank and Generation Scotland) from the primary GWAS. We defined ANX cases as meeting one of the following three criteria: (1) a likely lifetime DSM-IV GAD diagnosis based on the anxiety-related questions from the Composite International Diagnostic Interview short-form questionnaire^89^ and the first UK Biobank Mental Health Questionnaire^90^; (2) SRPD of one of the five core anxiety disorders (GAD, panic disorder, social phobia, agoraphobia, specific phobia; first and second UK Biobank Mental Health Questionnaires); or (3) having a GAD-7 score^91^ of ≥10, reflecting anxiety symptoms over the past 2 weeks (first and second UK Biobank Mental Health Questionnaires). Controls were defined in the same ways as the primary GWAS. We grouped individuals into three ancestry groups: EUR, AFR and South Asian.

We calculated PRS using MegaPRS^92^ within the GenoPred^93^ pipeline, which implements polygenic scoring approaches using the LDAK heritability model, whereby the variance explained by each SNP depends on its allele frequency, LD and functional annotations. Logistic regression was run to estimate the PRS prediction effect for ANX, adjusting for genotyping batch, assessment center and ten genetic principal components.

### Characterization and functional annotation of GWAS SNPs

We conducted variant fine-mapping and functional annotation (described in detail below). Note that although some gene prioritization approaches (for example, MAGMA, eQTL-based analyses, T-SMR) use different underlying statistical algorithms, they rely on overlapping expression datasets such as GTEx and PsychENCODE. Although eQTL uses only significant functional signals, T-SMR also incorporates sub-threshold functional signals that can better inform causal inference. These shared data sources mean that significant findings across methods are not fully independent. Given the challenges and biases associated with weighting schemes^94^, we chose to prioritize genes supported by three or more analyses, acknowledging the varying strengths of evidence but avoiding arbitrary weighting.

#### Variant fine mapping

We conducted statistical fine mapping using FINEMAP (v.1.3.1)^46^. Only variants located in a region of 1 Mb around index variants were included in the analyses. We used the default *k* = 5 maximum number of SNPs in credible sets, and the significant (suggestive) threshold for signals was set at 95% (50%) total posterior probability for the variants in credible sets (see Supplementary Table 10).

#### FUMA: functional annotation (eQTL/Hi-C)

We used FUMA (v.1.6.1) to examine the functional significance of our GWS loci. We compared results from brain-related eQTL studies to identify overlap in significance between our GWAS SNPs and the eQTL results. Furthermore, we used brain-related Hi-C information available through FUMA to functionally annotate our results. Standard settings were applied and results visualized using FUMA’s built-in circos plot routine. More information about the individual third-party datasets (available through the FUMA website) included in the analyses can be found in the Code Availability section or online in FUMA’s tutorial (https://fuma.ctglab.nl/tutorial).

#### Stratified LDSC

Two stratified LDSC analyses were conducted. First, the overall SNP heritability was partitioned into 53 overlapping functional genomic categories^95^. Second, SNP heritability was partitioned into 220 cell-type-specific regulatory elements based on GTEx data and data from the Franke Lab^96^. In both partitioned heritability analyses, we regressed the χ^2^ from the meta-analysis summary statistics onto LD scores downloaded from https://console.cloud.google.com/storage/browser/broad-alkesgroup-public-requester-pays. EUR allele frequencies derived from the 1000 Genome Project data were used as the reference genomes in both analyses. The enrichment of a functional or cell-type-specific category was defined as the proportion of SNP heritability in the category divided by the proportion of SNPs in that category.

#### FUMA: cell-type and tissue enrichment

We used MAGMA (v.1.08)^49^ as implemented in FUMA (v.1.6.1)^47^ to perform tissue-enrichment and cell-type-enrichment analyses. For tissue-enrichment analyses, we considered a set of 30 tissue groupings (average enrichment across all tissues in these groups) and 54 individual tissues (with 13 individual tissues from the ‘Brain’ group). Default settings were applied for all above-mentioned analyses. More information about the individual third-party datasets (available through the FUMA website) included in the analyses can be found in the Code Availability section or online in FUMA’s tutorial (https://fuma.ctglab.nl/tutorial).

### Gene-based associations and enrichment

#### MAGMA: gene-based GWAS and gene-set analysis

We performed gene-based and gene-set analyses using MAGMA^49^ (v.1.08) as implemented in FUMA^47^ (v.1.6.1). To test genetic associations at the gene level for the combined effect of SNPs in or near protein-coding genes, we applied default settings (SNP-wise model for gene-based analysis and competitive model for gene-set analysis). Gene-based *P* values were computed by mapping SNPs to their corresponding gene(s) based on their position in the genome. Positional mapping was based on ANNOVAR annotations, and the maximum distance between SNPs and genes was set to 10 kb (default). A multiple regression model was used while accounting for LD between the markers. The 1000 Genomes phase 3 reference panel^81^, excluding the MHC region, was used to adjust for gene size and LD across SNPs. Using the result of the gene-based analysis (gene-level *P* values), competitive gene-set analysis was performed with default parameters: 15,496 gene sets were tested for association. Gene sets were obtained from MSigDB (v.7.0) (see www.gsea-msigdb.org for details), including ‘Curated gene sets’ consisting of nine data resources, including KEGG, Reactome and BioCarta, and ‘GO terms’ consisting of three categories (biological processes, cellular components and molecular functions).

#### T-SMR, P-SMR and M-SMR

SMR methods are MR tests for assessing (causal) colocalization between significant trait association signals and significantly accurate predictions of molecular mediators or regulators (transcriptomic, proteomic and methylomic) that often use multiple variants, some of which, unlike classical colocalization methods, might possess only suggestive signals. If both trait and molecular mediator QTL signals are statistically significant, the SMR and classical colocalization methods are equivalent. However, the SMR methods accommodate (combinations of) non-significant QTLs that accurately predict molecular mediators, a situation still encountered for many genes owing to the low sample sizes for the reference molecular mediator-genetic data^97^.

We performed T-SMR, P-SMR and M-SMR studies using SMR (v.1.03)^50^ in conjunction with the largest available external blood and brain xQTL reference datasets (Supplementary Table 16). When protein QTL summary statistics from reference data were not available (blood and brain protein QTL) in the SMR-required input binary file format (that is, .besd), we processed them into the required format. One advantage of SMR over competing tools is the inclusion of the HEterogeneity In Dependent Instruments (HEIDI) test, which can be used as a proxy for likely causality.

SMR analyses were based on *cis*-xQTLs (SNPs with *P* < 5 × 10^−8^ within 2 Mb of the probe). We also used the default maximum (20) and minimum (3) number of xQTLs selected for the HEIDI test. We set the significance threshold as *P* < 1.57 × 10^−3^ for xQTL and the mismatch of minimum allele frequency among input files as <15%. For the HEIDI test, SNPs with LD > 0.9 and <0.05 with the top associated xQTL SNP were pruned.

To prioritize genes and perform pathway analyses, we adjusted probe (RNA, protein, CpG) SMR *P* value (${P}_{\mathrm{SMR}}$) for the HEIDI test *P* value (${P}_{\mathrm{HEIDI}}$) by combining the two *P* values into a single one by requiring that ${P}_{\mathrm{SMR}}$ was not penalized when ${P}_{\mathrm{HEIDI}}$ was above 0.01 and ${P}_{\mathrm{SMR}}$ was penalized by the amount ${P}_{\mathrm{HEIDI}}$ fell below 0.01. Consequently, we adjusted ${P}_{\mathrm{SMR}}$ to ${P}_{\mathrm{SMR}}^{{\prime} }=\frac{{P}_{\mathrm{SMR}}}{\min (\frac{{P}_{\mathrm{HEIDI}}}{0.01},1)}$. We used this approach instead of filtering by ${P}_{{H}{E}{I}{D}{I}} < 0.01$ because a misalignment between the GWAS cohort population and the EUR LD reference panel used by SMR might yield very low ${P}_{\mathrm{HEIDI}}$. We previously arrived at this compromise between the two types of SMR *P* values when applying this approach to many psychiatric disorders^52^, for example, the well-known SCZ *C4A* signal yielded a T-SMR ${P}_{\mathrm{HEIDI}}={5.94\,\times \,10}^{-4}$ but a much lower ${P}_{\mathrm{SMR}}$. However, for researchers who prefer to use the more conservative approach based on strict ${P}_{\mathrm{HEIDI}}$ thresholds described in the SMR paper^50^, we also provide gene ${P}_{\mathrm{HEIDI}}$ values for all SMR analyses, as documented in Supplementary Tables 15–17.

### Gene–drug associations

To uncover potential repurposing of existing drugs to ANX, we conducted gene–drug interaction analyses by applying the DrugTargetor^53^ method (v.1.3) to ANX summary statistics. DrugTargetor assesses the association of individual drugs or small-molecule-related gene sets and drug class enrichment. The method used two drug–gene interaction databases: ChEMB^54,98^ and DgiDB^55^. The analysis used the following settings: (1) hypothesized action for the nervous system; (2) both drug class and single drug; and (3) 1,500 maximum number of unique drugs and 200 maximum classes of drugs. Please see Supplementary Tables 22 and 23 and the README tab for the source databases used to accumulate the gene sets. Analyses were run using MAGMA (v.1.10)^49^ using gene flanks of −35 kb 5′ and +10 kb 3′ (ref. ^99^). Drug class enrichment was calculated using the area under the curve defined by the percent of drug class gene sets versus their rank in all the gene sets^100^.

### Genetic overlap between ANX and other phenotypes

#### PheWAS

Using the identified 58 GWS SNPs, we conducted a PheWAS to identify the variants that have been significantly associated with other psychiatric, physiological, medical and behavioral traits in prior GWAS, using the phewas function from the R packages ieugwasr^101^. The R package uses publicly available GWAS data from over 10,000 studies compiled by the IEU Open GWAS Project^101,102^. The PheWAS used the following databases:

- ebi-a: datasets that satisfy minimum requirements imported from the EBI database of complete GWAS summary data;
- finn-b: FinnGen study Data Freeze 5;
- ieu-a: GWAS summary datasets generated by many different consortia that have been manually collected and curated, initially developed for MR-Base;
- ieu-b: GWAS summary datasets generated by many different consortia that have been manually collected and curated, initially developed for MR-Base (round 2);
- ubm-a: complete GWAS summary data on brain region volumes as described by Elliott et al.^103^;
- ukb-d: Neale lab analysis of UK Biobank phenotypes, round 2.

This combination of databases provides the maximum coverage of published GWAS summary statistics that could be used for the PheWAS while minimizing duplication. To increase the accuracy of the PheWAS and consistency of the results across analyses for psychiatric disorders and related behavioral phenotypes, we supplemented the default GWAS summary statistics from the IEU Open GWAS Project for the traits we curated for the genetic correlation analyses. Curating the primary psychiatric and behavioral studies removed duplication from sequential GWAS analyses of the key disorders. We required that a SNP’s *P* value was GWS in both the current ANX GWAS and the alternative GWAS. Figure 2a was constructed using edited combinations of the following packages in R: alluvial^104^, qqman^105^ and pheatmap^106^.

#### Cross-trait genetic correlations

We used cross-trait LDSC to compute genetic correlations between the ANX meta-analysis and 112 selected disorders and traits with publicly available summary statistics. The sources of GWAS summary statistics can be found in Supplementary Table 24. Details of cross-trait LDSC can be found in the section “SNP-based heritability estimation and genetic correlations” (Methods). As a follow-up, we also calculated genetic correlations between the 112 phenotypes and each ascertainment-specific sub-cohort and compared the genetic correlation patterns between the four groups.

#### GSMR

We performed bi-directional GSMR^58^ analyses for trait pairs (ANX with MDD^107^, PTSD^57^ and neuroticism^39^) using GSMR (v.1.1.1), available in the GSMR R package. We used commonly applied parameters: (1) a 5 × 10^−8^ threshold for GWS signals; (2) the original HEIDI outlier method; (3) single-SNP and multi-SNP HEIDI outlier *P* = 0.01; (4) LD threshold for selecting MR SNP instruments of 0.05; and (5) false discovery rate threshold of 0.05. LD between SNPs with significant signals in at least one trait were computed using GCTA^108^ (v.1.94.1) based on the 1000 Genome Project^81^ EUR genetic data.

### Reporting summary

Further information on research design is available in the Nature Portfolio Reporting Summary linked to this article.

## Online content

Any methods, additional references, Nature Portfolio reporting summaries, source data, extended data, supplementary information, acknowledgements, peer review information; details of author contributions and competing interests; and statements of data and code availability are available at 10.1038/s41588-025-02485-8.

## Supplementary information

Supplementary Information Supplementary Notes 1–5 and Supplementary Figs. 1–94

Reporting Summary

Supplementary Tables Supplementary Tables 1–25

## Data Availability

Summary statistics excluding 23andMe are made available on the PGC data-download page (https://pgc.unc.edu/for-researchers/download-results). The replication GWAS summary statistics for the 23andMe data will be made available through 23andMe to qualified researchers under an agreement with 23andMe that protects the privacy of the 23andMe participants. Datasets will be made available at no cost for academic use. Please visit https://research.23andme.com/research-innovation-collaborations for more information and to apply to access the data.

## Code availability

Core analysis code for RICOPILI can be found at https://sites.google.com/a/broadinstitute.org/ricopili. This includes PLINK (https://www.cog-genomics.org/plink2), EIGENSOFT (https://www.hsph.harvard.edu/alkes-price/software), Eagle2 (https://alkesgroup.broadinstitute.org/Eagle), Minimac3 (https://genome.sph.umich.edu/wiki/Minimac3), SHAPEIT3 (https://mathgen.stats.ox.ac.uk/genetics_software/shapeit/shapeit.html), METAL (https://genome.sph.umich.edu/wiki/METAL_Documentation) and LDSC (https://github.com/bulik/ldsc). MAGMA can be found at https://ctg.cncr.nl/software/magma. GenomicSEM, specifically the tutorial ‘Models without Individual SNP effects’, can be found at https://github.com/GenomicSEM/GenomicSEM/wiki/3.-Models-without-Individual-SNP-effects. Additional code for data processing (for example, harmonization of summary statistics) can be found at https://zenodo.org/records/17478061.
