# Supplementary material for: Genome-wide association study of major anxiety disorders in 122,341 European-ancestry cases identifies 58 loci and highlights GABAergic signaling
Source: Nat Genet. 2026 Feb 3;58(2):275–88. doi: 10.1038/s41588-025-02485-8 (PMC12900644; doi:10.1038/s41588-025-02485-8)
Supplement: Supplementary file 1 — Supplementary Notes 1–5 and Supplementary Figs. 1–94 [file 41588_2025_2485_MOESM1_ESM.pdf]

nature genetics

Supplementary information https://doi.org/10.1038/s41588-025-02485-8

Genome-wide association study of major anxiety disorders in 122,341 European-ancestry cases identifies 58 loci and highlights GABAergic signaling

In the format provided by the authors and unedited

Supplementary Material – PGC-ANX1

Supplementary Notes and Supplementary Figures

1. **Supplementary Notes** **3**

Supplementary Note 1: Ethic approvals/consent 3

Supplementary Note 2: Methods: Sample descriptions 4

ALSPAC 4

Anxiety NeuroGenetics Study (ANGST) 5

BioVU 9

ESTBB 9

FinnGen 10

GEDI 11

GSC 13

HUNT 13

iPSYCH 15

MGH 16

MoBa 17

MUSP 18

MVP 19

PANIC 19

QIMR 20

STR 21

UKBB 24

Utah-Suicide 24

Summarystatistics harmonization across cohorts 25

Supplementary Note 3: Subgroup-specific meta-analyses 26

Supplementary Note 4: Results/Discussion: Detailed gene-findings 27

Supplementary Note 5: Consortia 27

MVP 27

23andMe 29

FinnGen 29

2. Supplementary Figures 48
QQ Plot for main ANX GWAS........ 48
Regional association plots and forest plots of the 58 independent significant SNPs........ 49
Heterogeneity test........ 108
Manhattan-plots and QQ-plots of sub-group analyses 109
Number of non-spurious dimensions in exploratory genomic factor analysis (paLDSC)........ 117
GenomicSEM 118
Characterization of GWAS SNPs 119
Tissue enrichment and cell-expression of ANX Genes........ 140
Previous Associations of ANX-SNPs 143
Cross-trait genetic correlations of the ascertainment-specific subgroup analyses........ 145
References 147

1. Supplementary Notes

Supplementary Note 1: Ethic approvals/consent

All relevant ethics approvals have been obtained by the respective cohorts. 23andMe: Participants provided informed consent and participated in the research online under a protocol approved by the external AAHRPP-accredited IRB, Ethical & Independent Review Services (E&I Review). Alspac: Ethical approval was obtained from the ALSPAC Ethics and Law Committee and the Local Research Ethics Committees. ANGST: ANGST-PCL was approved by the local institutional review board. All participants received a detailed description of the goal and funding of the study and signed a written consent. ANGST-MGS was approved by review boards at participating institutions. All subjects gave informed consent to participate in the study after explanation of study protocols and objectives. NESDA and NTR studies were approved by the Central Ethics Committee on Research Involving Human Subjects of the VU University Medical Center, Amsterdam. All subjects provided written informed consent. The Rotterdam Study has been approved by the institutional review board (Medical Ethics Committee) of the Erasmus Medical Center and by the review board of The Netherlands Ministry of Health, Welfare and Sports. The approval has been renewed every 5 years, as well as with the introduction of major new elements in the study (e.g., MRI investigations). The ethics committee of the University of Greifswald approved the ANGST-SHIP-STRAT study. ANGST-TRAILS was approved by the Dutch Central Committee on Research Involving Human Subjects. Participants were treated in compliance with the Declaration of Helsinki, and all measurements were carried out with their adequate understanding and written consent. bioVU: The Vanderbilt University Medical Center Institutional Review Board oversees BioVU and approved this project (IRB201609). EstBB: The EstBB study is approved by the Estonian Committee on Bioethics and Human Research (ref. 1.1-12/624). FinnGen: The Ethical Review Board of the Hospital District of Helsinki and Uusimaa approved the FinnGen study protocol Nr. HUS/990/2017. The FinnGen project was approved by Finnish Institute for Health and Welfare (THL), approval numbers THL/2031/6.02.00/2017, amendments THL/341/6.02.00/2018, THL/2222/6.02.00/2018 and THL/283/6.02.00/2019. Gedi: Gedi-GSMS: The study protocol and consent consent/assent forms were approved by the Duke University Medical Center Institutional Review Board. Gedi-VTSABD: The study protocol and consent/assent forms were approved by the Virginia Commonwealth University Institutional Review Board. Gedi-CHDS: In all cases, data have been gathered on the basis of signed and informed consent from young people and/or their parents. GSC: Written informed consent for linkage was obtained for GS:SFHS and only those individuals that provided informed consent were analyzed. HUNT: The HUNT study is approved by the Regional Committee for Medical and Health Research Ethics (ref. 2015/575). iPSYCH: The iPSYCH study was approved by the Regional Scientific Ethics Committee in Denmark and the Danish Data Protection Agency. MGH: The "International Cohort Collection for Bipolar Disorder" ("ICCBD"; MGH PI Smoller; #2008P002153) and "Partners Biobank" ("PKB"; PI Smoller; #2009P002312) studies receive Human Subject IRB approval from the Massachusetts General Hospital. MoBa: The establishment of MoBa and initial data collection was based on a license from the Norwegian Data Protection Agency and approval from The Regional Committees for Medical and Health Research Ethics. The MoBa cohort is now based on regulations related to the Norwegian Health Registry Act. The current study was approved by The Regional Committees for Medical and Health Research Ethics (20311). MUSP: Informed consent was given, and ethical approval was gained from the University of Queensland Human Research Ethics Committee 2019/HE002492, Mater Health Services Human Research Ethics Committee HREC/13/MHS/79. MVP: Consent is obtained in accordance with all VA policies and under the authority of the VA Central IRB. PANIC: PANIC study protocols were approved by the respective ethics committees and written informed consent obtained from all participants. QIMR: All QIMR studies were approved by the Human Research Ethics Committee of QIMR. STR: All participants gave informed consent, and the ingoing sub-studies of the Swedish Twin Registry have been

approved by Swedish ethical review board (2016/2135-31 for CATSS, 2008/1735-31/3 for SALTY). UKBB: Research on the UK Biobank is conducted under a generic Research Tissue Bank approval from the UK North West Multi-centre Research Ethics Committee (MREC). This research was approved to be conducted under that approval by the governing Research Ethics Committee of the UK Biobank. The analyses in this paper were performed under an approved extension to project 16577. Utah-Suicide: The Utah-suicide study is approved by Institutional Review Boards from the University of Utah, Intermountain Healthcare, and the Utah Department of Health.

We confirm that all necessary patient/participant consent has been obtained, and the appropriate institutional forms have been archived according to each country's and university's regulations, and that any patient/participant/sample identifiers included were not known to anyone (e.g., hospital staff, patients or participants themselves) outside the research group so cannot be used to identify individuals.

Supplementary Note 2: Methods: Sample descriptions

ALSPAC

Sample information

Data were from the Avon Longitudinal Study of Parents and Children (ALSPAC), a longitudinal cohort study that recruited pregnant women residing in the former area of Avon, UK with expected dates of delivery 1st April 1991 to 31st December 1992 (1,2). The initial cohort consisted of 14,062 live births, but has been increased to 14,901 children who were alive after one year with further recruitment (3). The study website contains details of all the data that is available through a fully searchable data dictionary and variable search tool: http://www.bristol.ac.uk/alspac/researchers/our-data.

Any-Anxiety-Disorder phenotypic derivation

An Any-Anxiety-Disorder phenotype was ascertained in ALSPAC using data from two research clinics assessed at age 18 and age 24. The Any-Anxiety-Disorder phenotype was derived using the Clinical Interview Schedule – Revised (CISR) (4), which measures the presence of psychiatric disorders using ICD-10 diagnostic criteria. The CISR was administered via a computer terminal on both occasions. Any-Anxiety-Disorder was defined as having panic, social phobia, specific phobia, and generalized anxiety disorder symptoms within the last 4 weeks at either age 18 and/or age 24. The total sample for the Any-Anxiety-Disorder phenotype was 3,940 with genetic data (controls: 3,404; cases: 536).

Genotype information

ALSPAC children were genotyped using the Illumina HumanHap550 quad chip genotyping platforms by 23andMe subcontracting the Wellcome Trust Sanger Institute, Cambridge, UK and the Laboratory Corporation of America, Burlington, NC, US. The resulting raw genome-wide data were subjected to standard quality control (QC) methods on 9,915 subjects and 550,000 SNPs. Individuals were excluded on the basis of gender mismatches; minimal or excessive heterozygosity; disproportionate levels of individual missingness (> 3%) and insufficient sample replication (IBD < 0.8). Population stratification was assessed by multidimensional scaling analysis and compared with Hapmap II (release 22) European descent (CEU), Han Chinese, Japanese and Yoruba reference populations; all individuals with non-European ancestry were removed by removing samples that clustered outside the CEU HapMap2 population using this multidimensional scaling of genome-wide IBS pairwise distances. SNPs with a minor allele frequency (MAF) of < 1%, a call rate of < 95% or evidence for violations of Hardy-Weinberg equilibrium (HWE, $p < 5x10^{-07}$) were removed. Cryptic relatedness was measured as proportion of identity by descent (IBD > 0.1). Related subjects that passed all other QC thresholds were retained during subsequent phasing and imputation. 9,115 subjects and 500,527 SNPs passed these QC filters.

ALSPAC mothers were genotyped using the Illumina human660W-quad array at Centre National de Genotypage (CNG) and genotypes were called with Illumina GenomeStudio. PLINK (v1.07) (5) was used to carry out QC measures on an initial set of 10,015 subjects and 557,124 directly genotyped SNPs. SNPs were removed if they displayed more than 5% missingness or a HWE p-value of less than $1x10^{-06}$. Additionally, SNPs with a MAF of less than 1% were removed. Samples were excluded if they displayed more than 5% missingness, had indeterminate X chromosome heterozygosity or extreme autosomal heterozygosity. Samples showing evidence of population stratification were identified by multidimensional scaling of genome-wide identity by state pairwise distances using the four HapMap populations as a reference, and then excluded. Cryptic relatedness was assessed using an IBD estimate of more than 0.125 which is expected to correspond to roughly 12.5% alleles shared IBD or a relatedness at the first cousin level. Related subjects that passed all other QC thresholds were retained during subsequent phasing and imputation. 9,048 subjects and 526,688 SNPs passed these QC filters.

We combined 477,482 SNP genotypes in common between the sample of mothers and sample of children. We removed SNPs with genotype missingness above 1% due to poor quality (11,396 SNPs removed) and removed a further 321 subjects due to potential ID mismatches. This resulted in a dataset of 17,842 subjects containing 6,305 duos and 465,740 SNPs (112 were removed during liftover and 234 were out of HWE after combination). We estimated haplotypes using ShapeIT (v2.r644) which utilizes relatedness during phasing. We obtained a phased version of the 1000 genomes reference panel (Phase 1, Version 3) (6) from the Impute2 reference data repository (phased using ShapeIT v2.r644, haplotype release date Dec 2013). Imputation of the target data was performed using Impute V2.2.2 against the reference panel (all polymorphic SNPs excluding singletons), using all 2186 reference haplotypes (including non-Europeans). This resulted in 28,699,419 SNPs, with 8,282,911 SNPs with a MAF > 0.01 and info score of > 0.8.

This gave 8,237 eligible children and 8,196 eligible mothers with available genotype data after exclusion of related subjects using cryptic relatedness measures described previously. Only ALSPAC children were used in the following analyses due to their available phenotypic information, but the data retain information regarding the mothers QC given their combined QC.

Statistical analysis

GWAS were conducted using SNPTEST v2.5.2 (7) adjusting for sex and the first 10 principal components (PCs) of ancestry. Age was not included into the model, as ALSPAC participants show little variation in age since they were all recruited at birth between April 1991 and December 1992.

Anxiety NeuroGenetics Study (ANGST)

We included GWAS summary statistics derived from the following cohorts as originally described in (8). Briefly, we conducted case-control association analyses for an Any Anxiety Disorder phenotype as defined below for each study. These consisted of logistic regressions with imputed SNP dosages under an additive genetic model with sex, age at interview, and ancestry PCs as covariates.

ANGST-PCL

The sample was randomly selected from the residents of the city of Lausanne (Switzerland) in 2003 according to the civil register (CoLaus) (9). 67% of the 35 to 66 year-old subjects who underwent the physical exam between 2003 and 2006 also accepted the psychiatric evaluation, which resulted in a sample of 3,717 individuals (PsyCoLaus-PCL), of whom 92% were of European ancestry (10). GWAS genotyping data from the Affymetrix 500K SNP array were available for 3,419 European ancestry participants of PsyCoLaus, with a final sample of individuals included in the GWAS of 852 cases and 1,103 controls. The GWAS included a final sample of 852 cases and 1,103

controls. As the original publication (8) did not provide specific sample sizes of the GWAS dataset, the final case and control numbers were estimated by maintaining the same case/control ratio as in the overall sample. The psychiatric assessment included the semi-structured Diagnostic Interview for Genetic Studies ((DIGS, French version (11)). The DIGS was completed with a section on GAD using the questions from the Schedule for Affective Disorders and Schizophrenia - Lifetime and Anxiety disorder version (SADS-LA, French version (12)). Similarly, the brief phobia chapter of the DIGS was replaced by the corresponding more extensive chapters from the SADS-LA. Diagnoses were made according to DSM-IV criteria.

ANGST-MGS

Subjects
Data were derived from the "control" sample originally part of the Molecular Genetics of Schizophrenia (MGS) study. The sample consisted of unrelated subjects selected by random digit dialing from approximately 60,000 US households. They were screened for psychotic and bipolar disorders for use as a comparison group for genetic association studies of these more severe psychiatric phenotypes, but they were not excluded for other common psychiatric disorders seen in the general population. The full MGS control sample is described in detail elsewhere (13). The data were obtained with permission from dbGaP (Database of Genotypes and Phenotypes, http://www.ncbi.nlm.nih.gov/gap, Study Accessions: phs000021.v3.p2 ("GAIN") and phs000167.v1.p1 "nonGAIN"). Data for the European American subjects were combined from the GAIN (N = 1,442) and nonGAIN (N = 1,367) datasets. The final sample size for the GWAS included 557 cases and 779 controls. Again, the GWAS sample size was estimated using the same ratio as the overall sample size.

Phenotypic Measures
All MGS control subjects completed an online psychiatric screening interview that included the lifetime version of the Composite International Diagnostic Interview, Short Form (CIDI-SF) (14). For those subjects with requisite response data, we applied DSM-based algorithms to the CIDI-SF responses to obtain the following six lifetime clinical phenotypes: GAD, panic attacks, agoraphobia, social phobia, and specific phobia as well as major depressive disorder. Cases included participants with any of the five primary anxiety diagnoses listed above. Controls had neither an anxiety nor depressive diagnosis.

Genotype Data
As previously described in detail (15), samples were genotyped at the Broad Institute using the Affymetrix 6.0 array. There were 2612 EA subjects and 680K autosomal SNPs available after QC procedures performed in PLINK. After phasing with SHAPEIT, genome-wide imputation with 1000 Genomes Project Phase I integrated reference set v3 (March 2012) was conducted using IMPUTE2 (16) under default parameters. After imputation, SNPs with MAF < 0.01, poor imputation quality < 0.30, and HWE p-value < 10^{-6} were removed. Association analyses were conducted with imputed dosage data in ProbAbel (17).

ANGST-NESDA/NTR

Subjects
The two parent projects that supplied data for this GWAS are large-scale longitudinal studies, the Netherlands Study of Depression and Anxiety (NESDA) and the Netherlands Twin Register (NTR) (18–20). Briefly, NESDA is an ongoing cohort study into the long-term course and consequences of depressive and anxiety disorders. In 2004–2007 2,981 participants aged 18 to 65 years were recruited from the community (19%), general practice (54%) and secondary mental health care (27%) and were followed-up during three biannual assessments. Persons who were not fluent in Dutch and those with a primary diagnosis of a psychotic disorder, obsessive compulsive disorder, bipolar disorder, or severe substance use dependence were excluded.

The NTR study (21) has been collecting longitudinal data on Dutch twin families since 1991 in over 200.000 young and adult participants. All cases were drawn from NESDA. Presence of a DSM-IV lifetime diagnosis of the following DSM-IV anxiety disorders as diagnosed via the Composite International Diagnostic Interview (CIDI) (22) (version 2.1) during one of the NESDA assessments: generalized anxiety disorder, social phobia, panic disorder and/or agoraphobia.

Control subjects were mainly from the NTR. Longitudinal phenotyping includes assessment of depressive symptoms (via multiple instruments), anxiety, neuroticism, and personality measures. Inclusion for controls required a low score on the trait version of the State-Trait Anxiety Inventory (23) or on a composite measure of neuroticism, anxiety and depression. A subsample of the NTR controls was also screened via a CIDI interview. The final GWAS sample contained 1,521 cases and 2,970 controls.

Genotyping, Imputation and Genome-Wide Association analysis
Whole blood and/or buccal DNA samples were collected for various NTR and NESDA projects (see (18,19)). Genotyping was performed on Affymetrix 6.0.

QC was performed within and between the different platforms and all genotypes were lifted over to build HG37 of the human genome. Genotypes that did not properly map to HG37 were removed as well as SNPs with a MAF below 1%, an allele frequency difference with the reference set above 20%, HWE < 0.00001, or a call rate below 95%.

IBD was calculated for all pairs; samples where IBD did not match the expected pedigree were removed. Cross platform concordance was calculated for samples that were genotyped on multiple platforms; samples showing a concordance < 99% were removed. Imputation was conducted in a two-stage approach. First, the genotype platform specific SNPs were imputed using the MaCH (24) software suite. Next, the reference-set SNPs were imputed using Minimach.

The genome-wide association analyses were performed on the dosage genotype data that were transformed into a single additive dosage score per SNP and imported into R. Related subjects or those from non-Western European ancestry were not included in the analyses. SNPs were selected with an R2 > 0.30, MAF > 0.01 and HWE > 10^{-05}.

ANGST-RS (1,2,3)

Subjects
The Rotterdam Study (RS) is an ongoing cohort study since 1989-1990 among the inhabitants of a district in Rotterdam (Ommoord) and aims to investigate the determinants of chronic diseases in the elderly. In 1990-1993, 7,983 people aged 55 years and older were recruited and re-examined every 3-4 years (RS1). In 2000-2001, the cohort was expanded by 3,011 additional people aged 55 and over (RS2). In 2006-2008, in a second expansion wave, an additional 3,932 persons, aged 45 and older, were recruited (RS3) (25). Data on both anxiety and depressive disorders was present for 6,800 people. The final GWAS contained 331 cases and 1,629 controls (RS1), 209 cases and 1,023 controls (RS2), and 370 cases and 1,817 controls (RS3).

Assessment of psychiatric disorders
Anxiety disorders (ADs) were diagnosed as part of the home interview. Trained lay interviewers conducted a slightly adapted version of the Munich version of the Composite International Diagnostic Interview (M-CIDI) to assess the following ADs with a computerized diagnostic algorithm according to DSM-IV criteria: GAD, panic disorder with or without history of agoraphobia, agoraphobia, social phobia, and specific phobia. The assessments of ADs were implemented to measure one-year prevalence, as lifetime prevalence in the elderly cannot be assessed reliably. The M-CIDI was specifically designed to obtain DSM-IV diagnoses of mental disorders, and test–

retest reliability for ADs is good (26). We merged cases with GAD, panic disorder, or any of the phobias in an Any-AD phenotype.

Depressive disorders were diagnosed during a home interview. During a first home interview, participants were screened for symptoms of depression with the Center for Epidemiological Studies Depression (CES-D) scale. Screen-positive persons (CES-D-score ≥ 16) were invited for a semi-structured clinical interview with the Schedules for Clinical Assessment of Neuropsychiatry (SCAN) (27). This interview was conducted by a trained clinician at the participant's home one week to two months (median time interval: two weeks) after the screening procedure and the anxiety interview. We were able to use the SCAN in this population-based setting because depression can be screened for with high sensitivity. With a computerized DSM-IV based diagnostic algorithm, major depression, minor depression, and dysthymia during the past month were diagnosed. We merged cases with any of these disorders into an Any-DEP phenotype.

Genotyping and Imputation
Genotyping was performed on 550K and 610K arrays (Illumina, Inc., San Diego, California) in 11,496 participants of the Rotterdam Study. The genotyped dataset was restricted to persons who reported that they were from European descent. Ethnic outliers were further excluded using identity-by-state distances > 4SD. Duplicates and first-degree or second-degree relatives were excluded using identity-by-state probabilities > 97% as well as samples with gender mismatch and excess autosomal heterozygosity. Variants with call rate < 95.0%, failing missingness test, HWE p value < $1x10^{-06}$, and MAF <1% were also removed. MACH 1.0 software (24) was used to impute to ~30M SNPs based on the 1000 genomes. SNPs included in imputation met the thresholds MAF ≥ 1%, HWE > $1x10^{-06}$, and SNP call rate ≥ 98%. Genotyping and analyses were conducted separately in three batches (RS1, RS2, RS3). For the case-control GWAS, we collapsed all ADs into an Any-AD category and used those as cases. Controls did not have Any-AD or Any-DEP. We conducted genome-wide association testing using GRIMP (28).

ANGST-SHIP-START

Subjects
We analyzed data from the Ship-START cohort of the Study of Health in Pomerania (SHIP-START)(29) comprising adult German residents in northeastern Germany. A two-stage stratified cluster sample of adults aged 20-79 years (baseline) was randomly drawn from local registries. Between 1997 and 2001, 4,308 European-ancestry subjects participated at baseline. From 2008 to 2012, the third phase of data collection (SHIP-START-2, N = 2,333) was carried out. In parallel the SHIP-LEGEND study (Life Events and Gene-Environment Interaction in Depression) was carried out for the detailed assessment of life-events and mental disorders (N = 2,400). The final GWAS sample contained 540 cases and 839 controls.

Interview and psychometric data
Sociodemographic factors and medical history were assessed by a computer-assisted face-to-face interview. The diagnosis of mental disorders was assessed using the Munich-Composite International Diagnostic Interview (M-CIDI) (23) in SHIP-LEGEND. The M-CIDI is a standardized fully structured instrument for assessing psychiatric disorders over the lifespan according to DSM-IV criteria.

Genotyping and Imputation
The SHIP-START sample was genotyped using the Affymetrix Human SNP Array 6.0. The overall genotyping efficiency of the GWA was 98.5%. Standard imputation procedures used the software IMPUTE v2.2.2 and all 1000 Genomes data (phase 1 version 3; March 2012). GWAS was conducted using logistic regression in QUICKTEST version 0.95.

ANGST-TRAILS

TRAILS (TRacking Adolescents' Individual Lives Survey) is a prospective population cohort study of Dutch adolescents with bi- or triennial measurements from age 11 to up until adulthood (30). Five assessment waves have been completed to date, which ran from March 2001 to July 2002 (T1), September 2003 to December 2004 (T2), September 2005 to August 2007 (T3), October 2008 to September 2010 (T4), and January 2012 to December 2013 (T5). Data for the present study were collected during the fourth assessment wave. At T1, 2,230 (pre)adolescents were enrolled in the study (response rate 76%, mean age 11.1, SD 0.6, 51% girls) (31). At T4, 84% (N = 1881, mean age 19.1, SD 0.6, 52% girls) participated again, of whom 1,584 (84%) received a diagnostic interview. Of these, 614 (cases: N = 278, controls: N = 336) were included in the case-control analyses. DSM-IV generalized anxiety disorder, social phobia, specific phobia, panic disorder, agoraphobia, and major depressive disorder were assessed with the Composite International Diagnostic Interview (CIDI), version 3.0. Genome-wide genotyping was performed by the Illumina Cyto SNP12 v2 array. This data was imputed using IMPUTE2 (1000 Genomes, March 2012 release) and association analysis was performed with SNPTEST v2.4.1.

BioVU

The BioVU population consists of individuals who receive care at Vanderbilt University Medical Center and choose to opt-in to the BioVU research study. Detailed description of program operations, ethical considerations, and continuing oversight and patient engagement have been published (32). This study was reviewed by the VUMC IRB and designated as non-human subjects research because of the use of fully de-identified data (IRB# 190418 and IRB# 201609). Individuals in BioVU have an average age of 55.17 years (median = 59, range = 3 to 112) and 56.9% are female (43.1% male). Genotype information and de-identified electronic medical records are available for research purposes for these individuals, including: ICD9/10 (International Classification of Diseases, 9th and 10th editions) billing codes, physician notes, and lab results. Genotype data for the BioVU population was generated using the Illumina Multi-Ethnic Genotype Array (MEGAEX) for 94,474 individuals. The genotype data were imputed into the HRC reference panel using the Michigan imputation server. The set was restricted to the 72,824 individuals who clustered with those of European ancestry. Detailed descriptions of imputation and QC applied to the MEGAEX genotype data are available in (32–34).

The case-control primary analysis was conducted in REGENIE (35) including 7,586 AnyANX cases and 50,545 controls selected according to the following criteria. Exclusion criteria for all individuals were ICD-9 and ICD-10 psychotic disorders, developmental disorders, and intellectual disabilities. Controls also excluded any anxiety or mood disorders, posttraumatic stress disorder, and obsessive compulsive and related disorders due to their genetic relatedness with ANX. AnyANX cases included any ICD-9 and ICD-10 adult anxiety disorder: GAD, panic disorder, social phobia, agoraphobia, and specific phobia including all subtypes as well as anxiety disorder, unspecified.

ESTBB

Estonian Biobank (EstBB) is a population-based cohort (N = 200,000) with a rich variety of phenotypic and health-related information collected for each participant (36,37). At recruitment, participants signed a consent allowing follow-up linkage of their electronic health records (EHR), thereby providing a longitudinal collection of their phenotypic information. The EstBB database includes health records from the National Health Insurance Fund Treatment Bills (from 2004), Tartu University Hospital (from 2008), and North Estonia Medical Center (from 2005), and data from different registries (causes of death, cancer, etc.). For all the participants EstBB provides

information on the diagnoses in ICD-10 coding and information on drug dispensing data, including drug ATC codes, prescription status and purchase date (if available).

Genotyping of DNA samples from the Estonian Biobank was done at the Core Genotyping Lab of the Institute of Genomics, University of Tartu using the Illumina Global Screening Arrays (GSAv1.0, GSAv2.0, and GSAv2.0_EST). Altogether 200,000 samples were genotyped and then PLINK format files were created using Illumina GenomeStudio v2.0.4. During the QC all individuals with call-rate < 95% or mismatching sex that was defined based on the heterozygosity of X chromosome and sex in the phenotype data, were excluded from the analysis. Variants were filtered by call-rate < 95% and HWE p-value < $1x10^{-04}$ (autosomal variants only). Variant positions were updated to Genome Reference Consortium Human Build 37 and all variants were changed to be from TOP strand using reference information provided by Dr. Will Rayner from the University of Oxford (https://www.well.ox.ac.uk/~wrayner/strand/). Before imputation variants with MAF < 1% and Indels were removed. Prephasing was done using the Eagle v2.3 software (38) (number of conditioning haplotypes Eagle2 uses when phasing each sample was set to: --Kpbwt=20000) and imputation was carried out using Beagle v.28Sep18.793 (39,40) with an effective population size N_e = 20,000. As a reference, Estonian population specific imputation reference of 2,297 WGS samples was used (41).

For the current study, we determined 12,523 cases of anxiety based on the participants EHRs as individuals with any of the following ICD-10 codes: F40.0 (agoraphobia), F40.1 (social phobia), F40.2 (specific phobia), F41.0 (GAD), and F41.1 (panic disorder). Controls (N = 134,954) were defined as undiagnosed individuals with records of BIP or MDD were removed. Finally, we further excluded individuals with F20, F25, F84, and F70-F79 ICD10 codes from the entire analysis. We conducted the GWASs using REGENIE (35) adjusting for the first ten PCs of the genotype matrix, as well as for birth year, birth year squared and sex.

FinnGen

The FinnGen (https://www.finngen.fi/en) study combines genotype data with longitudinal health record registry data of Finland. The registry data includes the Causes of Death Register (from 1969), Hospital Discharge information (from 1969), Surgeries and outpatient care (from 1998), Drug purchase register (1995-2018), Primary health care data (2011-2018), Drug reimbursement registry (1964-2017), and Finnish Cancer Registry (1953-2017). In this collaboration data is derived from the FinnGen Data Freeze 5 with a total of 218,792 individuals, mostly collected between August 2017 and August 2019 (collection is further ongoing). The here included data has not been published before but a general description of the FinnGen study can be found elsewhere (42).

FinnGen study participants and donated samples are recruited from epidemiological and disease-based cohorts, as well as from hospital biobanks. Individual data from different registries was connected with national social security number, and this data was connected to genotype data with pseudonym codes. All Finns can take part in the study by giving a biobank consent allowing the use of their samples. All FinnGen data is strictly pseudonymized, and participants cannot be identified from the data. Finnish samples are population-based biobank samples collected between August 2017 and August 2019 (collection further ongoing), including legacy samples collected since the 1980s.

Including disease-based cohorts to FinnGen has resulted in over-representation of certain disorders, which is why individuals with records of mental retardation, psychotic disorders, or pervasive developmental disorders were carefully excluded from all analysis. Controls were not allowed to have any record of bipolar disorder, major depression or any anxiety disorder. Individuals were identified as cases if they had any phobic anxiety, generalized anxiety disorder, panic disorder or unspecified anxiety diagnosis. ICD-8 and ICD-9 diagnoses were matched to ICD-

10 diagnoses to avoid period effects. A total of 9,182 anxiety cases and 160,108 controls were included in the analyses.

The genome-wide association analyses were conducted as follows: genotypes from several Illumina and Affymetrix FinnGen Axiom arrays were pre-phased using Eagle v2.4 and imputed using Beagle v4.1 using The Sequencing Initiative Suomi (SISu v3) reference panel consisting of 3,775 whole-genome sequences (depth up to 30x) and 16,962,023 variants. Before analyses all variants with minor allele count below 5 and imputation quality score below 0.6 were removed. Association analyses were performed using logistic regression with Firth-fallback hybrid algorithm using PLINK v2.0. Age at death or now, sex and 10 genetic PCs were used as covariates.

GEDI

We included three cohorts from the Gene-Environment-Development Initiative (GEDI) collaboration.

GEDI-GSMS

Cohort

The Great Smoky Mountains Study (GSMS) is a longitudinal, representative study of 1,420 children in 11 predominantly rural counties in Southeastern United States (43). Annual assessments on psychopathology and associated factors were completed on the 1,420 children until age 16 (6,674 observations of 1,420 individuals; 1993 to 2000) and then again at ages 19, 21, 25, and 30 (4,556 observations of 1,336 participants; 1999 to 2015) for a total of 11,230 total assessments. The final ANX sample included 244 cases and 217 controls.

Phenotypic assessment

All anxiety constructs were assessed using the structured *Child and Adolescent Psychiatric Assessment* (CAPA) (44,45) until age 16 years; and the upward extension of the CAPA, the Young Adult Psychiatric Assessment (YAPA), at ages 19, 21, 24 to 26 years, and age 30. Both a parent and the participating child were interviewed at ages 9 to 16 years; beginning at age 19, only the participant was interviewed. All interviews were coded by a trained interviewer and then checked by a supervisor. Anxiety disorders assessed included separation anxiety, generalized anxiety, social phobia, specific phobia, agoraphobia, and panic disorder, as well as DSM-III-R overanxious disorder (46). The time frame for determining the presence of psychiatric symptoms was the previous three months. Onset dates for all symptoms were also assessed and were used in diagnostic algorithms when DSM-IV criteria required a symptom duration of three or more months. A detailed glossary provides the operational rules for identifying clinically significant symptoms. Scoring programs for the CAPA and YAPA, written in SAS by the senior authors, combined information about the date of onset, duration, and intensity of each symptom to create diagnoses according to the DSM. A symptom was counted as present if it was reported by either parent or child or both, as is standard in child and adolescent epidemiological studies, approximating the process of combining information from multiple informants in clinical practice. Two-week test–retest reliability of CAPA diagnoses in children and adolescents aged 10 through 18 years is comparable to that of other structured child psychiatric interviews. Construct validity, as judged by 10 different criteria including comparison to other interviews and ability to predict mental health service use, is good to excellent. (All measures, a glossary, and codebooks are provided at http://devepi.duhs.duke.edu/instruments.html.)

Genotyping, QC, and Analysis

GSMS was genotyped using Illumina Human660W-Quad v1 and imputed using the HRC (Version r1.1 2016) reference panel. The following pre-imputation variant filters were used: call rate < 95%, MAF < 1%, HWE < $1x10^{-08}$, and strand ambiguity; and the following pre-imputation sample filters were used: call rate < 90%; heterozygosity > 5 SD; sex mismatch, and relatedness. Additionally, samples were filtered using Mahalanobis

distance applied to the top 10 population stratification PCs, to ensure EA, using 1000 Genomes samples as a population structure reference panel. Imputed SNPs were filtered by AvgCall > 0.9 and R2 > 0.5. Analyses were performed in REGENIE (35), using its standard two-step modeling approach, and specifying the model as logistic regression with Firth correction, with sex and age as covariates. REGENIE applied a MAC > 5 filter in all GWAS tests. Three GWAS were run, one for the full EA sample, one for EA males, and one for EA females.

GEDI-VTSABD

Cohort

The VCU arm of the NIDA-funded GEDI combined existing phenotypic and environmental data from the Virginia Twin Study of Adolescent Behavioral Development (VTSABD) study (47–50), a population-based multi-wave, cohort-sequential twin study of adolescent psychopathology and its risk factors, with genome-wide genotyping, generating a genotyped sample of ~900 subjects among the 1,412 European-ancestry twin families who participated in the study. The final ANX dataset included 224 cases and 406 controls. *Phenotypic assessment*

All anxiety constructs were assessed using the structured *Child and Adolescent Psychiatric Assessment* (CAPA) until age 17 years; and the Structural Clinical Interview for DSM-III-R (SCID) at ages 18 and older. Both a parent and the participating child were interviewed at ages 9 to 17 years; beginning at age 18, only the participant was interviewed. The interviews were conducted in the same way as described for the Gedi-GSMS sample. For Gedi-VTSABD the scoring programs for the used instruments – CAPA and SCID were written in SAS, as for Gedi-GSMS.

Genotyping, QC, and Analysis

VTSABD was genotyped using Illumina Human660W-Quad v1 and imputed using the HRC (Version r1.1 2016) reference panel. The following pre-imputation variant filters were used: call rate < 95%, MAF < 1%, HWE < $1x10^{-08}$, and strand ambiguity; and the following pre-imputation sample filters were used: call rate < 90%; heterozygosity > 5 SD; sex mismatch, and relatedness. Additionally, samples were filtered using Mahalanobis distance applied to the population stratification PCs, to ensure EA ancestry, using 1000 Genomes samples as a population structure reference panel. Imputed SNPs were filtered by AvgCall > 0.9 and R2 > 0.5. Analyses were performed in REGENIE, using its standard two-step modeling approach, and specifying the model as logistic regression with Firth correction, with sex and age as covariates. REGENIE applied a MAC > 5 filter in all GWAS tests. Three GWAS were run, one for the full EA sample, one for EA males, and one for EA females.

GEDI-CHDS

The New Zealand arm of the Gene-Environment-Development Initiative (GEDI) utilized data from the Christchurch Health and Development Study, a longitudinal study of the life course development of a birth cohort of 1,265 children born in the Christchurch (New Zealand) urban region in mid-1977 (51,52). The final ANX dataset included 244 cases and 217 controls.

Phenotypic assessment

At ages 18, 21, 25, 30 and 35, participants completed a structured interview that assessed aspects of their mental health since the previous assessment. Relevant components of the Composite International Diagnostic Interview (CIDI) were used to assess DSM-IV symptom criteria for a range of anxiety disorders including generalized anxiety disorder, panic disorder, agoraphobia, social phobia and specific phobia. For the purposes of this analysis, participants were classified as having an anxiety disorder if they met diagnostic criteria for any of the above disorders at any time during the interval from age 16-35 years. Separate questioning was also conducted to assess DSM-IV symptom criteria for major depressive disorder at each assessment; and at later ages for bipolar disorders, PTSD, and schizophreniform disorders. This information was used to define the comorbidity exclusion criteria used in the present study.

Genotyping, QC, and Analysis

CHDS was genotyped using Illumina Human660W-Quad v1 and imputed using the HRC (Version r1.1 2016) reference panel (53). The following pre-imputation variant filters were used: call rate < 95%, MAF < 1%, HWE < $1x10^{-08}$, and strand ambiguity; and the following pre-imputation sample filters were used: call rate < 90%; heterozygosity > 5 SD; sex mismatch, and relatedness. Additionally, samples were filtered using Mahalanobis distance applied to the population stratification PCs, to ensure EA ancestry, using 1000 Genomes samples as a population structure reference panel. Imputed SNPs were filtered by AvgCall > 0.9 and R2 > 0.5. Analyses were performed in REGENIE, using its standard two-step modeling approach, and specifying the model as logistic regression with Firth correction, with sex as a control variable (age is uniform in the CHDS cohort; thus, it was invariant and not included in the model). REGENIE applied a MAC > 5 filter in the GWAS tests.

GSC

Generation Scotland (GSC) (54): Scottish Family Health Study (GS:SFHS) is a family- and population-based cohort of approximately 24,000 individuals in Scotland. Participants were recruited from general practitioner offices and eligible if they were 18 years of age or older and had at least one first-degree relative who was also willing to participate (55,56). Genotype data was available for 19,994 individuals (57) and the Sanger Imputation Service was used to create the imputed data set (58). Diagnoses were obtained through data linkage administered by National Health Service (NHS) Scotland Information Services Division.

Anxiety status was derived from primary care (GP) diagnoses using CALIBER (phenotype code 267) on data from 18,126 participants. There were 1,341 anxiety cases identified. Controls were identified from the remaining participants after screening for depression using data from GP diagnoses (CALIBER phenotype code 272), from an in-person SCID interview at baseline, and an online follow-up using the CIDI. After screening there were 13,165 control participants. 442 participants who overlapped with the UK Biobank cohort and 83 participants who did not meet genotyping QC were removed.

A GWAS was conducted on a final filtered dataset of 1,306 cases and 12,675 controls using REGENIE (35). Genomic predictions were produced using genotyped SNPs filtered for MAC < 100, MAF < 0.01, sample missingness and variant > 0.1, and HWE < $1x10^{-15}$. Imputed SNPs were tested using Firth logistic regression filtered for MAC < 100 and INFO < 0.4 with four PC covariates.

HUNT

The Nord-Trøndelag Health Study (HUNT) is an ongoing population based cohort study from the county of Nord-Trøndelag in Norway (59,60). All inhabitants aged 20 years or older were invited to participate in the HUNT1 survey (1984-1986), the HUNT2 survey (1995-1997), and the HUNT3 survey (2006-2008). In addition, all inhabitants aged 13 to 19 years were invited to participate in the Young-HUNT1 survey (1995-1997), the Young-HUNT2 survey (2000-2001), and the Young-HUNT3 survey (2006-2008). Approximately 120,000 inhabitants have participated in at least one survey.

All participants have provided questionnaire, interview, and measurement data, which can be found at the HUNT databank [https://hunt-db.medisin.ntnu.no/hunt-db]. In addition, about 80,000 participants have provided biological samples for storage at the HUNT biobank [https://www.ntnu.edu/hunt/hunt-biobank]. The Norwegian Identification Number can be used to link data from local and national registries to data from the HUNT database and the HUNT biobank.

Phenotype definitions

Assessments

The health care system in Norway is publicly funded and the psychiatric departments in Nord-Trøndelag have catchment area responsibilities for the whole county. Diagnostic evaluations are ordinarily made in interdisciplinary teams where at least one medical doctor and one specialist in psychiatry or psychology are present. We obtained data from local hospital registries on ICD-9 and ICD-10 codes from all inpatient and outpatient contacts from 1987 through 2017 for all genotyped participants in the HUNT study. Also, we obtained data on ICPC codes (61) from primary care doctor registries from 2006 through 2018, and data on self-reported psychiatric disorders, self-reported use of medications, and results on psychometric tests from questionnaires in the HUNT database.

Cases

We made three definitions of anxiety disorders. Participants fulfilling the criteria for more than one of the definitions were only included in the highest ranked definition.

1) *Anxiety disorder clinically diagnosed in local hospitals* was defined by at least one inpatient or outpatient local hospital contact due to an ICD-10 diagnosis of F40.0 agoraphobia (n = 467), F40.1 social phobia (n = 909), F40.2 specific isolated phobia (n = 134), F41.0 panic disorder (n = 577), or F41.1 generalized anxiety disorder (n = 1,000). Patients with ICD-9 diagnoses of anxiety disorders were not included as senior clinicians working in Nord-Trøndelag the last decades reported that the relevant diagnostic codes (e.g. ICD-9 300.X) were used for both affective disorders and anxiety disorders. A total of 2,093 unique participants were registered with one or more anxiety disorder diagnoses. The majority of the participants had their diagnosis registered at a psychiatric department (n = 1,922) and from ≥ 2 separate visits (n = 1,639). The mean age at first recorded diagnosis was 46 years, which is higher than the age at onset for most anxiety disorders (62). This discrepancy can reflect a delay in treatment contact (63) and that a proportion of the participants had an established anxiety disorder before the start of data inclusion to the local hospital registries in 1987.

2) *Anxiety disorder clinically diagnosed in general practice* was defined by at least one contact due to an ICPC-2 diagnosis of P74 anxiety disorder (n = 3,514). The majority of the participants were registered with a diagnosis from ≥ 2 separate visits (n = 2,864). Excluding participants who also had a clinical anxiety disorder diagnosed in local hospitals (n = 895) gave a total of 2,619 independent participants. The ICPC-2 diagnosis criterion of P74 is "Clinically meaningful anxiety, which is not limited to specific environmental situations. It manifests by panic disorder (recurrent attacks of severe anxiety, which is not limited to specific situations, with or without physical symptoms), or like a disorder of generalized and persisting anxiety, which is not limited to specific situations, occurring with various accompanying physical symptoms". Of importance, the ICPC-2 also defines a symptom diagnosis of anxiety termed P01 feeling of anxiety/nervousness/tension. The criterion of P01 is "Emotions that the patient conveys as an emotional or psychological experience and that are not attributed to the occurrence of a mental disorder (..)." Only participants with a P74 diagnosis were included in the case definition. The mean age at first recorded diagnosis was 56 years. As for our first case definition, both a delay in treatment contact and a delay in registration of diagnoses in the primary care registry can explain the discrepancy between the age at first registered diagnosis and the expected age at onset of anxiety disorders.

3) *Self-reported anxiety disorder* was defined by a score on the Hospital Anxiety and Depression Scale – Anxiety subscale (HADS-A) > 11 (n = 2,626). Excluding 750 participants diagnosed with anxiety disorders at local hospitals and/or in general practice gave a total of 1,876 independent participants with HADS-A defined anxiety disorder. In a literature review, the HADS-A was found to have an optimal balance between sensitivity and specificity at scores ≥ 8 (64), meaning that our definition favors specificity above sensitivity.

The final dataset included a total of 6,587 ANX cases.

Controls

We defined general criteria for controls for all our GWASs on psychiatric phenotypes (n = 40,742). These are 1) age > 40 years by 2017, 2) no ICD-9 or ICD-10 diagnoses of psychiatric disorders in local hospital registries, 3) no ICPC-2 (https://ehelse.no/icpc-2e-english-version) diagnosis of psychiatric disorders in primary care doctor registries, 4) no self-reported psychiatric disorders in the HUNT questionnaires, 5) no self-reported daily use of antidepressants, relaxants, or sleeping medications in the HUNT questionnaires, and 6) HADS-A and HADS-D $\leq$ 8.

Genotyping, QC, imputation, and association testing

A total of 71,860 participants in the HUNT study have been genotyped with Illumina HumanCoreExome arrays (HumanCoreExome12 v1.0, HumanCoreExome12 v1.1, or UM HUNT Biobank v1.0). We excluded participants whose genotypes had 1) call rates < 99%, 2) contamination > 2.5%, 3) large chromosomal copy number variants, 4) lower call rate of technical duplicate pair or twins, 5) uncommon sex chromosomal constellations (i.e. others than XX or XY), or 6) discrepancies with reported gender. The remaining genotypes were analyzed in a second round of genotype calling following the Genome Studio QC protocol (65) [https://www.illumina.com/techniques/microarrays/array-data-analysis-experimental-design/genomestudio.html]. We used BLAT (66) to determine genomic position, strand orientation and reference allele of all genotyped variants, using the Genome Reference Consortium Human genome build 37 [http://genome.ucsc.edu] and the revised Cambridge Reference Sequence of the Human Mitochondrial DNA [http://mitomap.org] as reference. Variants were excluded if they had 1) call rates < 99%, 2) higher call rates genotyped in another assay, 3) probe sequences not mapping to the reference genome, 4) cluster separation < 0.3, 5) gentrain score < 0.15, or 6) HWE deviation from unrelated samples of European ancestry with p < 0.0001. To harmonize the three arrays, we removed variants with frequency differences >15% between the datasets or were monomorphic in one dataset and had MAF > 1% in one of the others. We inferred ancestry, using PLINK v1.90 (67), to project the genotype samples into the space of the PCs of the Human Genome Diversity Project reference panel (68,69) [http://csg.sph.umich.edu/chaolong/LASER]. Only individuals of European ancestry were included. Eagle2 v2.3 (38) were then used to phase the data. A total of 69,716 samples passed the QC.

Imputation was performed using Minimac3 v2.0.1 software (70) http://genome.sph.umich.edu/wiki/Minimac3]. For the autosomal variants we used a customized merged reference panel of 1) 2,201 low-coverage whole-genome sequenced samples from the HUNT study and 2) the Haplotype Reference consortium release 1.1 (HRC v1.1), excluding 1,023 samples from the HUNT study. For the X-chromosome, we used the HRC v1.1 alone. Variants with estimated squared correlations between imputed and true genotypes (Rsq) < 0.3 were excluded, resulting in a total of ~25 million well-imputed variants.

The final dataset included 6,587 ANX cases and 40,740 controls. We used the REGENIE for testing of associations between binary traits and common genetic variants. The REGENIE method is tailored for GWAS analyses of psychiatric traits in population-based data from a restricted geographic area as it controls for case-control imbalance and relatedness.

iPSYCH

In the scope of The Lundbeck Foundation Initiative for Integrative Psychiatric Research (iPSYCH), Danish nation-wide population-based case-cohort samples were collected and genotyped. All analyses of the samples were performed on the secured national GenomeDK high performance computing cluster in Denmark (https://genome.au.dk). See Pedersen et al., 2018 (71) and Bybjerg-Grauhom et al., 2020 (72) for a detailed description of the overall cohort, array genotyping and QC as well as Meier et al., 2019 (73) for an ANX-related GWAS in iPSYCH. Eligible were singletons that had been a Danish resident on their first birthday. As ANX was not

one of the primary disorders iPSYCH collected data for, all ANX patients that are included are either comorbid with one of the primary disorders in iPSYCH or stem from the population-based pool of controls. Genetic information was obtained by the Statens Serum Institut (SSI) at the Danish Neonatal Screening Biobank (DNSB) from heel prick blood samples that had been collected from all newborn babies in Denmark. Genotyping was performed on the PsychChip v 1.0 array (Illumina, San Diego, CA, USA) at the Broad Institute of MIT and Harvard (Cambridge, MA, USA). Genotyping and data processing was carried out in 25 waves. Genetic information was coupled with other registers via the Danish Civil Registration System (CPR). ANX cases were identified by the Danish Psychiatric Central Research Register (DPCRR) which collects data on all individuals treated in Denmark either in psychiatric hospitals or in outpatient psychiatric clinics that met ICD10 (F40.0-F41.9) criteria. Controls were randomly selected from the same birth cohorts and excluded individuals with a diagnosis of diagnoses of anxiety, stress-related, and mood disorders. Genotypes were processed using the Rapid Imputation and COmputational PIpeLIne for Genome-Wide Association Studies (Ricopili (74)) performing stringent QC of the data. Samples with call rates below 95% and individuals with a mismatch between sex obtained from genotyping and registered sex in the DPCRR were excluded. Related individuals were removed, principal component analysis (PCA) was used to exclude ancestral outliers and the data was imputed using the HRC reference panel. The final dataset included 5,288 individuals with a diagnosis of ANX and 14,422 controls.

MGH

Data were included from two prior studies conducted at the Massachusetts General Hospital (MGH).

MGH-ICCBD

Cases and controls were collected as part of the Inter-national Cohort Collection for Bipolar Disorder (ICCBD), a US, UK, and Swedish consortium established to accelerate genomic studies of BD by applying high throughput phenotyping methods (75–77). The Massachusetts General Hospital (MGH) site of the ICCBD collected DNA bipolar disorder cases and controls by linking discarded blood samples to de-identified electronic health record (EHR) data. Only the MGH data is included herein. Cases and controls were identified by deriving EHR-based phenotyping algorithms trained and validating on the Mass General Brigham Research Patient Data Registry (RPDR) (78), which spans more than 20 years of data from 4.6 million patients. Control patients were selected to be at least 30 years old and had no ICD-9 codes or history of medications related to a psychiatric or neurological condition. Control patients were matched 15:1 to the algorithm-classified case patients on the basis of age, gender, race/ethnicity, and health care utilization (number of facts) by using a standard frequency matching approach.

As described elsewhere (77), bipolar disorder case and control patients identified by the algorithms underwent semistructured diagnostic interviews using the Structured Clinical Interview for DSM-IV (SCID-IV) by an experienced doctoral-level clinician blinded to EHR case control status. We developed and validated algorithms to classify relevant subphenotypes among cases: age at bipolar disorder onset, bipolar disorder subtype, family history of bipolar disorder, and history of: alcohol dependence, drug dependence, suicide attempt, psychosis, or panic disorder/agoraphobia. Two board- certified psychiatrists manually reviewed 620 notes to identify important terms (features) indicative of each subphenotype. Each feature was extracted from the notes by using the HITEx system (79). The gold standard subphenotype classification was based on results of the SCID direct interview and was used to train algorithms using the extracted features. All case patients were used in the training phase regardless of whether they received a SCID diagnosis of bipolar disorder. We trained a separate model for each subphenotype by using the LASSO regression procedure with 10-fold cross-validation. For the panic disorder/agoraphobia phenotype used as cases for the current study, the algorithm area under the receiver operating curve (AUC) = 0.73, positive predictive value (PPV) = 0.83 (95% CI: 0.72–0.91), and negative predictive

value (NPV) = 0.67 (95% CI: 0.56–0.77) based on 143 SCID interviews (77). The final ANX cohort consisted of 1,823 ANX cases and 5,151 controls.

MGH-PBK

Anxiety cases and controls for GWAS were ascertained using EHR data from patients in the Mass General Brigham (MGB; formerly Partners Healthcare) Biobank (80) in the MGB health system. DNA from blood samples were genotyped from MGB Biobank participants on three Illumina arrays (MEGA, MEGAEX, and MEG BeadChip). During QC, SNPs were excluded based on call rate (< 95%) and HWE ($p < 1x10^{-10}$), while individuals were excluded based on missing data (> 2%), sex assignment errors, heterozygosity (±3 SD), and relatedness (randomly removing one individual from any pair with kinship > 0.2). Genotyping array batches were merged then imputed using the Michigan Imputation server (Minimac4 1.2.1, HRC/1KG reference panel). Imputed data were then converted to best-guess genotypes for all markers with high-quality imputation (R2 > 0.8) and common MAF (> 1%) with participants of European ancestry retained for these analyses. EHR data before February 2020 were extracted for these participants including ICD-9/10 diagnostic codes. Anxiety cases were defined based on having two or more anxiety diagnostic codes and excluding those with schizophrenia, psychosis, or autism-related codes), while controls were defined based on having zero anxiety diagnostic codes and excluding those with depression, bipolar, PTSD, schizophrenia, psychosis, or autism-related codes). The final ANX cohort consisted of 1,121 ANX cases and 11,897 controls.

Further processing for association testing was done using the RICOPILI pipeline (74). The GWAS was conducted in each cohort separately using logistic regression with the imputed additive genotype dosages. The first 4 principal components (PCs) were included (along with additional PCs that tested significantly in a Wald test on case-control differences) as covariates to correct for population stratification, and variants with imputation INFO score < 0.8 or minor allele frequency (MAF) < 0.01 were excluded.

MoBa

The Norwegian Mother, Father, and Child Cohort Study (MoBa) is a population-based cohort study conducted by the Norwegian Institute of Public Health (81). Participants were recruited from all over Norway from 1999-2008. The women consented to participation in 41% of the pregnancies. The cohort now includes 114,500 children, 95,200 mothers and 75,200 fathers. The current sample is based on version 12 of the quality-assured data files released for research in January 2019. Blood samples were obtained from the mothers and fathers at 17–18 weeks of gestation and from mothers and children (umbilical cord) at birth (82).

Genotyping and QC

Genotyping of the entire MoBa cohort was still ongoing at the time of analysis. For the current study we used the available genotype data from 17,000 randomly selected trios, genotyped in three batches. The first two batches, comprising 20,664 and 12,874 individuals were genotyped at the NTNU Genomics Core Facility (Trondheim, Oslo) using the Illumina HumanCoreExome (Illumina, San Diego, USA) genotyping array, version 12 1.1. and version24 1.0, respectively. The third batch, comprising 17,949 individuals, was genotyped at ERASMUS MC (the Netherlands) using the Illumina Global Screening Array (Illumina, San Diego, USA) version 24 1. PLINK version 1.90 beta 3.36 was used to conduct the QC (83). After QC, phasing was conducted using Shapeit 2 release 837 and the duoHMM approach was used to account for the pedigree structure. Imputation was conducted using the Haplotype reference consortium release 1-1 as the genetic reference panel. The Sanger Imputation Server was used to perform the imputation with the Positional Burrows-Wheeler Transform. The phasing and imputation were conducted separately for each genotyping batch. Post imputation QC was performed by initially converting the dosages to best-guess genotypes. Individuals were removed if they had a genotyping call rate less than 99% or were of non-European ethnicity. SNPs with an imputation INFO quality score less than 0.8, genotyping call rate

less than 98%, MAF less than 1%, or a HWE p-value less than $1x10^{-06}$ were removed. A core homogeneous sample of European ethnicity (based on PCA of markers overlapping with available HapMap markers) across all batches and arrays were available for use in analysis (totals prior to analysis-specific exclusions for relatedness: N mothers = 14,804; N fathers = 15,198). For this freeze, we analysed unrelated individuals (within generation, defined as accumulated identity-by-descent < 0.015 and overall identity-by-descent PI_HAT < 10%) for the any anxiety (AnyANX) Case-Control study.

Cases
The anxiety cases were obtained from the MoBa parents by linkage to International Classification of Diseases (ICD)-10 diagnostic information from the Norwegian Patient Registry (NPR), available from 2008 to 2018. Cases were defined by at least one contact due to agoraphobia (F40.0), social phobia (F40.1), specific phobias (F40.2), panic disorder (F41.0) or GAD (F41.1). Individuals registered with schizophrenia, schizotypal, and delusional disorders (F20-29) or intellectual disability (F70-79) were excluded from the case group. The total number of AnyANX cases was 715 (280 from batch1, 180 from batch2, and 255 from batch3).

Controls
Our controls were defined as having no contacts due to psychiatric disorders in the NPR (any F-codes in ICD-10) and with no self-reported anxiety, depression, or life-time history of depression in the MoBa questionnaires. We also excluded participants scoring above the 95% percentile on the 5-item version of the Hopkins Symptom Checklist measuring symptoms of anxiety and depression. The total number of controls was 22,168

GWAS
The GWAS analysis was conducted using scalable and accurate implementation of generalized mixed model (SAIGE) (84) adjusting for age, sex, genotyping batch, and population stratification (PC 1 to 5).

MUSP

The Mater-University of Queensland Study of Pregnancy (MUSP) and its outcomes began in 1981 with data collected on 7,223 pregnant woman-child pairs (6,753 mothers of whom 520 had 2 study children less 50 who had multiple births).

At the 30-year follow-up of the offspring, 2,541 individuals participated (85). One of the assessments was the World Health Organization Composite International Diagnostic Interview (WHO-CIDI) version 2.1 which was administered to measure anxiety disorders, major depression and substance use disorders. In addition, eating disorders (anorexia nervosa) and ADHD were assessed.

DNA was collected via blood and analyzed with Illumina Infinium PsychArray-24 BeadChip. After standard QC, genotype data was generated on 1,306 unique individuals. There were 1,031 individuals with genotype and phenotype data that were included in the GWAS. Of these, 316 had a diagnosis of any anxiety disorder including generalized anxiety disorder, panic disorder, agoraphobia, social phobia, or specific phobia, and 715 were controls.

Processing of the GWAS data was done using the RICOPILI pipeline. This included QC, imputation (using HRC reference data) and PCA analysis with standard settings. The GWAS was conducted using logistic regression with the imputed additive genotype dosages. The first 4 principal components (PCs) were included (along with additional PCs that tested significantly in a Wald test on case-control differences) as covariates to correct for population stratification, and variants with imputation INFO score < 0.8 or minor allele frequency (MAF) < 0.01 were excluded.

MVP

The Million Veteran Program (MVP) is a US national research program conducted by the United States Department of Veterans Affairs to learn how genes, lifestyle, and military exposures affect health and illness. This cohort has been previously described in detail (86).

Samples were genotyped using a 723,305-SNP Affymetrix Axiom Biobank array customized for MVP. Imputation was performed with minimac3 using data from the 1000 Genomes Project. For post-imputation QC, SNPs with an imputation INFO score < 0.3 or a MAF < 0.001 were removed from analysis.

The ANX GWAS that produced the summary statistics used in the current meta-analysis was previously reported in detail (87). Self-reported physician diagnosis of anxiety disorder was analyzed based on data collected from the MVP baseline survey. Participants were asked, "Please tell us if you have been diagnosed with the following conditions: anxiety reaction/panic disorder." Answers were recorded as yes/no binary responses, and missing responses were excluded from analysis. A total of 192,256 participants had available phenotype and genotype information and had assignments of European ancestry resulting in 28,525 cases and 163,731 controls. These were analyzed for genome-wide association via logistic regression in PLINK 2.0 on genotype dosage data covarying for age, sex, and the first 10 PCs.

PANIC

We included individual summary statistics from five panic disorder (PD) GWAS cohorts as previously described in detail (88). Their principal features are reiterated briefly below.

Cohorts

PD cases were included from five of the six European cohorts as named in the original manuscript and designated in the Sample Table: Germany I (PANIC-GOM3), Germany II (PANIC-GMDC), Germany III (PANIC-GWC3), Denmark (PANIC-DCA1), and Sweden (PANIC-SWDF). Participants from the Estonian PD cohort were not included herein because they overlapped some individuals from the Estonian Biobank described above. Various validated clinical interview assessments (CIDI, SCID, MINI) were conducted to establish diagnosis of PD.

Genotyping

Genomic DNA was prepared from whole blood using standard procedures. DNA samples of patients with PD were genome-wide genotyped using the Illumina Infinium HumanCoreExome (patients from Denmark, Sweden, and Germany I), 317K/610Q (Germany II) and 660W-Quad (Germany III) BeadChips.

Controls from Germany I were genotyped using the Illumina OmniExpress BeadChip at the Department of Genomics, Life & Brain Center, University of Bonn, Germany. For the remaining controls, genome-wide genotype data were obtained from previous studies: OmniExpress (Denmark, Estonia, and Sweden); 317K/610Q (Germany II); and 550K (Germany III).

QC and imputation

QC and imputation procedures are described in detail elsewhere (88–90). Briefly, the QC parameters used for the exclusion of SNPs and individuals were as follows: SNP missingness > 0.05 (prior to the removal of individual subjects); SNP missingness per individual > 0.02; autosomal heterozygosity deviation (|Fhet| > 0.2); SNP missingness > 0.02; difference in SNP missingness between patients and controls > 0.02; and deviation of an SNP from HWE ($p < 1x10^{-10}$ in patients, $p < 1x10^{-06}$ in controls). The imputation of genotype data in each of the individual case-control cohorts was performed using IMPUTE2/SHAPEIT (91) and the 1,000 Genomes Project reference panel (release v3.macGT1).

Across all case-control cohorts, relatedness testing and population structure was analyzed using a subset of 47,513 SNPs fulfilling stringent QC criteria (imputation INFO score > 0.8; SNP missingness < 0.01; MAF > 0.05) and subjected to LD pruning (r^2 > 0.02). In cryptically related individuals, one member of each pair (π-hat > 0.2) was removed at random with patients being retained in the preference to controls. PCs were estimated from the genotype data, and case-control association was tested using logistic regression in the RICOPILI pipeline (74) controlling for PCs 1–7, 11, 16, and 18 as covariates. Meta-analysis across these samples was performed using METAL (92) with inverse standard error weights. The final analyses had the following sample sizes: Germany I (PANIC-GOM3): 472 cases, 1,803 controls; Germany II (PANIC-GMDC): 247 cases, 537 controls; Germany III (PANIC-GWC3): 280 cases, 855 controls; Denmark (PANIC-DCA1): 248 cases, 970 controls; and Sweden (PANIC-SWDF): 561 cases, 2,591 controls.

QIMR

Summary statistics were provided for three studies conducted at the QIMR Berghofer Medical Research Institute in Brisbane, Australia.

Diagnostic information collection for the first cohort (Adult Twins) was described in detail in Otowa et al., 2016 (8). Briefly, 6,925 individuals, across four studies, completed sections of various structured clinical assessments from which diagnoses of any ANX could be made. Since the publication of Otowa et al, a further study - The Prospective Imaging Study of Aging (PISA) – with overlapping participants from the previous studies, was established. PISA currently consists of over 3,000 genotyped individuals that have completed extensive behavioral, psychological, and medical questionnaires (93). As part of the protocol, participants were administered the GAD-7 in addition to a question about difficulty with functioning. A score of 10 or more on the GAD-7 with significant impact upon functioning were the criteria for assigning anxiety cases for PISA. Furthermore, participants were provided with a list of psychiatric disorders and asked to report any disorder for which they'd been given a diagnosis by a medical professional. Data were amalgamated across all five studies and participants were assigned as a case if they met the diagnostic criteria for any anxiety disorder or self-reported a diagnosis by a medical professional in any of the studies. Individuals who did not meet diagnostic criteria or self-report a diagnosis of any psychiatric disorder in any of the studies were included as controls. All related individuals that met criteria were included, resulting in a final sample size of 2,448 cases and 6,343 controls.

The second family cohort, the 19up (QIMR_NUUP) study, used a structured clinical assessment (Composite International Diagnostic Interview) to collect lifetime prevalence of DSM-IV anxiety diagnoses of social anxiety and panic disorder (94). Individuals were included as cases if they met DSM-IV criteria for one of the disorders (N = 1,131) and controls if they did not meet criteria for either disorder (N = 1,233). Individuals were not excluded based on family-relatedness.

The third cohort, the Australian Genetics of Depression Study (AGDS), comprises approximately 20,000 participants who self-report a diagnosis of depression (~16 000 genotyped). Cohort description of the AGDS has been described previously (95). Cases were defined as individuals that self-reported having a diagnosis of generalized anxiety disorder, social anxiety, or panic disorder (N = 7,434). Controls were individuals from the QSkin study who reported not having been given a diagnosis of any psychiatric disorder (N = 12,729) (96).

Genotyping of all three studies was conducted using one of several Illumina genotyping platforms (Illumina 317K, 370K, 610K or Infinium Global Screening Array). A full description of the QC and imputation procedure is given elsewhere (94). Marker exclusion criteria included: unknown or ambiguous map position and strand alignment in a BLAST search, missingness > 5%, HWE $p < 1x10^{-06}$), MAF < 1%, GenTrain score < 0.6. The Michigan imputation server was used to impute the genotypes using the HRCr1.1 as a reference panel. Individuals were excluded based

on a high missingness (missing rate > 3%), inconsistent (and unresolvable) sex, or if deemed ancestry outliers from the European population (6 SD deviations from the first two genetic principal components from 1000 Genomes). Imputed genotype dosages were used for the analyses.

GWAS analyses for all three cohorts were run with sex, age and 10 PCs as covariates. GWAS was carried out in SAIGE (v0.36.3.3; using the LOCO option) in R 3.6.1 using a generalized linear mixed model to account for population stratification, cryptic relatedness, and unobserved genetic confounding. SAIGE is able to account for relatedness between individuals through fitting a genetic relatedness matrix (GRM) to the model. HRC-imputed genotype data were used to calculate the GRM between participants. Variants with MAF < 1% and imputation accuracy score < 0.6 were excluded from the results.

STR

The Swedish Twin Registry (STR) (97–99) is composed of three primary cohorts from which we utilized data as described below. In order to identify anxiety disorder Cases (ANX) and Controls for the current GWAS, we first linked STR records to the Swedish National Patient Registry (100) and the Prescribed Drug Register (101). We defined ANX cases through several options. First, we identified 268 patients in the NPR with ICD-9 or ICD-10 GAD, panic disorder, agoraphobia, social phobia, and specific phobia or ICD-8 Anxiety Neurosis or Phobic Neurosis. Next, we combined patient and medication data to identify an additional 3,358 patients specifically treated for "anxiety" with an anxiolytic medication (antidepressants, benzodiazepines, buspirone, antihistamines, pregabalin, or propranolol). The remaining N = 15,344 records represent a preliminary set of controls after removing patients with diagnoses of either mood or psychotic disorder. These numbers were further revised to those listed in Supp Table S1 by applying additional psychiatric evaluation criteria from individual cohorts as described below.

STR-CATSS

Study population

Since 2004, the Child and Adolescent Twin Study in Sweden (CATSS) (102) has continuously contacted the parents or other guardians of Swedish-born twin children by the time the twins turn nine years of age. The purpose of the contacts has been dual, both to include new twins into the STR and as a part of a research program focusing on the development of common health and behavioral problems during childhood and adolescence.

By May 2019, 16,476 parental interviews concerning 32,952 twins had been completed with an overall response rate of 69%. All twins not opting out of further contacts are recontacted at age 15 (twins and parents), 18 (twins and parents) and 24 (twins only). The current analyses include data from ages 18 and 24. The age 18 survey included items assessing exaggerated fears, interference, and duration criteria allowing for the creation of diagnoses of agoraphobia, social phobia, and specific phobia. It also included a section assessing symptoms of major depressive disorder (MDD) and a version of the Screen for Child Anxiety Related Emotional Disorders (SCARED) (103). The age 24 survey included the Hospital Anxiety and Depression Scale (HADS) (64) and the Center for Epidemiologic Studies Depression Scale—Revised (CESD-R) (104). Panic and GAD were not assessed in CATSS. Thus, among the five major anxiety disorders, only cases of phobias could be directly assigned in CATSS. We assigned Control status to those participants who had no phobias or MDD or had SCARED total scores below 28, HADS-A or HADS-D scores below 11, or CES-D scores below 16.

Genotyping and Imputation

Genotyping was performed at SNP&SEQ Technology Platform Uppsala, Sweden using the Illumina PsychChip bead chip (PsychChip_15048346_A). Genotyping results for 11,173 subjects passed the initial lab-based QC. Genotypes are encoded on the `+` strand of the GRCh37/hg19 build of the human reference genome. 11,173 genotyped samples were processed using the RICOPILI pipeline (74) for QC. After first removing SNPs with missingness > 0.05

(N = 4,477), 141 samples failed sample QC due to any of the following: per-sample call rate < 0.98; excessive heterozygosity (FHET outside +/- 0.2); sex mismatch. 146,755 out of 588,454 markers failed SNP QC due to any of the following: per-SNP call rate < 0.98; invariant; HWE ($p < 1x10^{-06}$ in controls and $p < 1x10^{-10}$ in cases); difference in call rate between cases and controls > 0.02. Furthermore, 139,072 SNPs with MAF < 1% were excluded, leaving 302,627. By projecting the first two PCs of the study samples to the reference panel of 1000 Genome global population, we identified 236 samples as non-European ancestral outliers with first two PCs exceeding six standard deviations from the mean values of the European samples in the reference population. After excluding ancestral outliers, the same QC procedure as described above was reiterated, leading to a further exclusion of seven samples and 23 SNPs. The data taken forward to imputation thus consisted of 10,789 samples and 302,604 SNPs.

Of the SNPs passing QC, 293,590 were successfully aligned to the forward genomic strand and matched to the reference panel. We then used the Sanger imputation service (https://imputation.sanger.ac.uk/) to impute the post-QC genotype data to the reference panel of Haplotype Reference Consortium data (HRC1.1) (105). EAGLE2 (38) and PBWT (106) were used for prephasing and imputing respectively. 40,359,612 SNPs were available after the HRC imputation (before any post-imputation quality filters). After imputation, genotypes for 2,417 non-genotyped MZ twins with a genotyped co-twin in the sample were added to this dataset by copying the co-twin's genotypes. Finally, imputed data was converted to plink2 pgen format (https://www.cog-genomics.org/plink/2.0/formats#pgen) and filtered to exclude imputed markers with imputation INFO score < 0.1, and MAF < 0.005. 9,140,287 markers passed these filters.

STR-SALTY

Study population

The Screening Across the Lifespan of Twins Younger (SALTY) study was a collaborative effort between STR and researchers in epidemiology, medicine, political science and economics. The target population was the younger part of the SALT cohort born between 1943 and 1958. The data collection began in the fall of 2008 by sending an extensive questionnaire to 24,914 Swedish twins, and it was completed in the summer of 2010. The survey generated a total of 11,647 responses (47%). Out of these, 11,482 (98.6%) respondents gave informed consent to have their responses stored and analyzed. The data collection consisted of three parts: (1) an extensive self-report paper questionnaire; (2) saliva collection for DNA extraction (see under biobanking) and (3) a request to participate in a web-based investigation that included questions on musical experience, tendency to experience psychological flow and creative achievement, as well as tests of cognitive and motor performance. A total of 3,070 twins aged between 50 and 67 (mean 58.9) years chose to participate in that web-based extension of the SALTY study (107). We used diagnoses of GAD and MDD from the original Screening Across the Lifespan Twin (SALT) study (108) to assign additional 47 ANX Cases and remove 1,108 depressed Controls, respectively.

Genotyping and Imputation

Genotyping was performed as for the CATSS sample described above. Genotyping results for 5,546 subjects passed the initial lab-based QC. 5,546 genotyped samples were processed using the RICOPILI pipeline (74) for QC. After first removing SNPs with missingness > 0.05 (N = 4,566), 65 samples failed sample QC due to any of the following: per-sample call rate < 0.98; excessive heterozygosity (FHET outside +/- 0.2); sex mismatch. 183,433 out of 588,454 markers failed SNP QC due to any of the following: per-SNP call rate < 0.98; invariant; HWE ($p < 1x10^{-06}$in controls and $p < 1x10^{-10}$ in cases); difference in call rate between cases and controls > 0.02. Furthermore, 99,534 SNPs with MAF < 1% were excluded, leaving 300,921. By projecting the first two PCs of the study samples to the reference panel of 1000 Genome global population, we identified two samples as non-European ancestral outliers, whose first two PCs exceeded six standard deviations from the mean values of the European samples in the reference population. After excluding ancestral outliers, the same QC procedure as described above was

reiterated, leading to a further exclusion of two samples and one SNP. The data taken forward to imputation thus consisted of 5,477 samples and 300,920 SNPs.
Of the SNPs passing QC, 291,924 were successfully aligned to the forward genomic strand and matched to the reference panel. We applied the same imputation procedure as above. 40,359,612 SNPs were available after the HRC imputation (before any post-imputation quality filters). After imputation, genotypes for 1,065 non-genotyped MZ twins with a genotyped co-twin in the sample were added to this dataset by copying the co-twin's genotypes. Finally, imputed data was converted to plink2 pgen format and filtered to exclude imputed markers with imputation INFO score < 0.1, and MAF < 0.005. 9,111,991 markers passed these filters.

STR-TWINGENE

Study population
The TwinGene project, conducted between 2004 and 2008, is a population-based Swedish study of twins born between 1911 and 1958. The study participants have previously participated in a telephone interview during the SALT study conducted between 1998 and 2002. To be included in TwinGene, both twins within a pair had to be alive. The zygosity of the twins was based on self-reported childhood resemblance or by using DNA markers (for 18% of the total sample). In total, 12,591 individuals participated by donating blood to the study, and by answering questionnaires about lifestyle and health. No additional anxiety or depressive disorders were identified in TwinGene.

Genotyping and Imputation
After excluding subjects in which the DNA concentration in the stock-solution was below 20ng/µl as well as subset of 302 female monozygous twin pairs participating in a previous genome wide effort DNA from 9,896 individual subjects was sent to SNP&SEQ Technology Platform Uppsala, Sweden for genome wide genotyping with Illumina OmniExpress bead chip (HumanOmniExpress-12v1_A) (all available dizygous twins + one twin from each available MZ twin pair). Genotyping results for 9,836 subjects passed the initial lab-based QC. Genotypes are encoded on the + strand of the GRCh37/hg19 build of the human reference genome. 10,947 genotyped samples were processed using the RICOPILI pipeline (74) for QC. After first removing SNPs with missingness > 0.05 (N = 1,709), 29 samples failed sample QC due to any of the following: per-sample call rate < 0.98; excessive heterozygosity (FHET outside +/- 0.2); sex mismatch. 41,972 out of 731,442 markers failed SNP QC due to any of the following: per-SNP call rate < 0.98; invariant; HWE ($p < 1x10^{-06}$in controls and $p < 1x10^{-10}$ in cases); difference in call rate between cases and controls > 0.02. Furthermore, 47,969 SNPs with MAF < 1% were excluded, leaving 641,501. By projecting the first two PCs of the study samples to the reference panel of 1000 Genome global population, we identified six samples as non-European ancestral outliers, whose first two PCs exceeded six standard deviations from the mean values of the European samples in the reference population. After excluding ancestral outliers, the same QC procedure as described above was reiterated, leading to a further exclusion of one sample and four SNPs. The data taken forward to imputation thus consisted of 10,911 samples and 641,497 SNPs.
Of the SNPs passing QC, 638,601 were successfully aligned to the forward genomic strand and matched to the reference panel. We applied the same imputation procedure as above. 40,359,612 SNPs were available after the HRC imputation (before any post-imputation quality filters). After imputation, data was converted to plink2 pgen format and filtered to exclude imputed markers with imputation INFO score < 0.1, and MAF < 0.005. 9,113,633 markers passed these filters.

GWAS Analyses (all cohorts)
GWAS analyses involved converting dosages to hard calls using PLINK defaults, imposing filters for missingness (98%), MAF (1%), and INFO (>0.8). SNPs and individuals with call rates below 98% were excluded. HWE filtering removed SNPs with p≤1.00E-06. Individuals with heterozygosity (F>|0.2|) were removed. PCA was conducted, projecting relatives into the space of unrelated individuals, and clustering was assessed with and without

reference samples (e.g., 1KG). Logistic regression GWAS tested ANX association with SNPs including sex and the first 10 PCs as covariates.

UKBB

Sample and assessment

Participants were recruited from the UK Biobank (UKBB) study Mental Health assessment (109) and consisted of 126,443 adults aged 46-80 years of Western European ancestry.

Cases met lifetime criteria for any anxiety disorder but did not have a self-report diagnosis of schizophrenia, bipolar disorder, autism spectrum disorder, attention deficit hyperactivity disorder or eating disorder. Lifetime anxiety disorder was assessed using two approaches. First were individuals who self-reported via single questionnaire items having received a diagnosis from a mental health professional for one of the core five anxiety disorders (generalized anxiety disorder, social phobia, panic disorder, agoraphobia, or specific phobia; N = 21,108). Second were individuals who met criteria on self-reported lifetime experiences of symptoms for one of the same five core anxiety disorders, reported on the anxiety sections of the Composite International Diagnostic Interview (CIDI) Short Form (CIDI-SF) questionnaire (22). This identified 9,641 cases of whom 4,345 were cases not identified via the single-item reporting. The total number of cases was therefore 25,453 of whom 66% were female.

Genotyping and QC

Genotype data were collected and processed as part of the UKBB extraction and QC pipeline (110). SNPs imputed to the Haplotype Reference Consortium (HRC) reference panel or genotyped SNPs were used for these analyses (hg19 build). SNPs with a MAF > 0.01 and INFO score > 0.4 were retained. We excluded data from related individuals.

Genome-wide association analyses

Analyses were limited to individuals of European ancestry defined by 4-means clustering on the first two ancestry PCs. Covariates (age at time of assessment, sex, genotyping batch, assessment centre, and the first six genetic PCs) were regressed out of each phenotype using logistic regression performed using R (v. 3.5.1). We used the residuals as dependent variables to predict any anxiety disorder in genome-wide association analyses using BGENIE v1.2 software2 (111).

Utah-Suicide

The Utah Suicide Genetic Research Study (USGRS) contributed data from suicide deaths to this study with diagnostic data obtained from electronic health records. The USGRS is an ongoing study of genetic and environmental risk factors leading to suicide death (112,113). It benefits from more than two decades of unprecedented close collaboration with the Utah Department of Health's centralized Office of the Medical Examiner. Suicide status is made by the Medical Examiner following detailed investigation of the scene and circumstances of the death and is given conservatively due to its impact on survivors. High quality DNA is extracted from whole blood using the Qiagen Autopure LS automated DNA extractor at the University of Utah Clinical and Translational Science Center (CTSI, https://ctsi.utah.edu/). Identifiers from cases are used to link each death to data within the Utah Population Database (UPDB; https://uofuhealth.utah.edu/huntsman/utah-population-database/). The UPDB is a state-wide database that contains over 27 million data records on over 12 million individuals, including demographics and health records data that link to ~85% of suicide deaths studied by the USGRS. After linking to health data within the secure UPDB computing environment, identifiers are stripped before data are released to the research team to protect privacy and confidentiality. This ongoing study is

approved by Institutional Review Boards from the University of Utah, Intermountain Healthcare, and the Utah Department of Health.

Overall, the Utah suicide deaths were primarily male (541/832 = 65.02%), and the average age at death was 41.72 years (standard deviation = 15.53 years). Most suicides were reported as White by the Medical Examiner (827/842 = 98.22%). Within the subset with anxiety disorder, relatively fewer were male (175/298 = 58.72%), and age at death was slightly younger (40.27, std dev = 14.30). Genotyping was performed using the Illumina PsychArray platform.

Processing of the GWAS data was done using the RICOPILI pipeline. This included QC, imputation (using HRC reference data) and PCA analysis with standard settings. The GWAS was conducted using logistic regression with the imputed additive genotype dosages. The first 4 principal components (PCs) were included (along with additional PCs that tested significantly in a Wald test on case-control differences) as covariates to correct for population stratification, and variants with imputation INFO score < 0.8 or minor allele frequency (MAF) < 0.01 were excluded. The final GWAS dataset contained 258 cases and 279 controls.

Summary statistics harmonization across cohorts

The results from the individual GWASs were harmonized and transformed to 'daner' file format following RICOPILI (74) specifications. All datasets were transformed to genome-build GRCh37 assembly (b37/Hg19). Variants were removed if they had: minor allele frequency (MAF) of less than 1% in cases or controls, imputation quality score (INFO) of less than 0.8 or out-of-bounds (above 1.2), or if the effect measures, p-value or standard error (SE) was missing or out of bounds. The data were aligned to the HRC reference panel. If necessary, variants were flipped to match the orientation in the reference panel, and marker names were standardized to those present in the HRC reference. Any variant that did not overlap with the HRC reference was removed. Strand-ambiguous A/T and C/G SNPs with MAF over 0.4 were removed. For A/T and C/G SNPs with a MAF below 0.4, their allele frequencies were compared to the frequencies in the HRC reference. If the allele frequency matched, meaning it was also the minor allele in the reference, the same strand orientation was maintained. If the frequency didn't match, meaning it had a frequency greater than 0.5 in the reference, the alleles were assumed to be on different strands and were flipped. As a last step, we applied the software-package DENTIST (Detecting Errors in aNalyses of summary staTISTics) (114) as an additional quality control step and removed SNPs with errors and/or heterogeneity between the summary data and the LD reference by testing the difference between the observed Z-score of each variant and its predicted value from surrounding variants. The removal of SNPs due to DENTIST filters were between 83 and 206,037 SNPs per cohort dataset.

The level of heterogeneity between the studies was evaluated through two measures: Cochran's Q and I^2 statistics. Cochran's Q is a weighted sum of squared differences between the individual study effects and the combined effect across studies with the weights coming from the pooling method. The I^2-statistic measures the proportion of variation among studies that is due to heterogeneity and not due to chance. While Q depends on the number of included studies, I^2 is independent of the number of cohorts. The genomic control factor lambda (λ) was calculated for each GWAS and for the overall meta-analysis to detect residual population stratification or systematic technical issues.

Supplementary Note 3: Subgroup-specific meta-analyses

Like most large-scale psychiatric GWAS, these samples were ascertained and assessed with variable approaches that introduce known and cryptic sources of heterogeneity – see S**upplementary Note 2** for details of each study. In an attempt to address such heterogeneity, we classified each of the 36 cohorts into a) five ascertainment subgroups (clinical, biobank, community, self-reported professional diagnosis, and comorbid) and b) three assessment subgroups (interview, ICD-10, biobank) – see **Supplementary Table 1**. While for both a and b classifications, some cohorts meet criteria for more than one subgroup, cohorts were grouped according to their most defining characteristic (i.e. if a biobank sample relied on self-reported diagnosis, it was categorized into SRPD rather than Biobanks).

With regards to the ascertainment subgroups, cases from the two largest cohorts (MVP and UKBB) were primarily identified by self-reported professional diagnosis (SRPD) (2 cohorts, 53,978 cases and 221,844 controls). UK Biobank also included likely DSM-IV GAD cases assigned from responses to the anxiety section of the Composite International Diagnostic Interview (CIDI) short-form survey included in the Mental Health Questionnaire as described in Purves et al. (115). Community cases were ascertained from either a certain geographic area or from a birth cohort or twin registry (19 cohorts, 14,044 cases and 48,283 controls). Biobank cases were identified in large-scale biobanks or registries using ICD or DSM ANX codes (6 cohorts, 37,714 cases and 420,412 controls). Comorbid cases were primarily recruited for another comorbid psychiatric disorder (3 cohorts, 12,858 cases and 27,514 screened controls). Clinical cases were diagnosed by a healthcare professional in a clinical setting (6 cohorts totaling 3,631 cases and 11,907 screened controls); these were mostly identified for panic disorder.

With regards to the assessment subgroups, the interview-based assessment group contained 21 cohorts (ALSPAC, all ANGST cohorts, all GEDI cohorts, MGH-ICCBD, MUSP, all Panic cohorts, QIMR_Ad, and QIMR_Nuup; 13,396 cases and 35,272 controls), the SRPD assessment group contained three cohorts (MVP, UKBB, and QIMR_AGDS; 61,412 cases and 234,573 controls; note that in the ascertainment subgrouping QIMR_AGDS was assigned to the comorbid-subgroup), and the ICD-10 subgroup contained 12 cohorts (EstBB, FinnGen, GSC, HUNT, MoBa, PBK1, all three Swedish cohorts, Utah, bioVU, and iPSYCH; 47,533 cases and 460,036 controls).

The ascertainment-specific meta-analyses identified four (SRPD), three (Biobanks), one each (Comorbid, Community), and zero (Clinical) independent significant loci. Of those nine identified loci, six were significant in the main meta-analysis (**Supplementary Table 3**). Assuming a population prevalence of 20%, the subgroups provided a range of estimated SNP-based heritabilities from 6.9% (95% CI = [6.87-6.93]; Community) to 23.7% (95% CI = [23.58-23.82]; Clinical; see **Supplementary Table 4A** for all heritability estimates, including for other assumed prevalences). Genetic correlations (rG) among the subgroups ranged from 0.63 (SE = 0.21, between Clinical and Community) to 1.00 (between Community and both Comorbid and SRPD; see **Supplementary Table 4B** for all correlation estimates). The assessment-specific meta-analyses resulted in eight (ICD-10), five (SRPD), and zero (interview) independent significant loci. Of those 13 identified loci, 10 were also significant in the main meta-analysis (though not a lead SNP for the respective loci), while two (overlapping with the main-meta loci) were significant in at least one of the ascertainment-specific meta-analyses (three of the identified SNPs overlapped between both, slightly differing, SRPD diagnoses, while one SNP overlapped between the ICD-10 and biobank meta-analyses). SNP-heritability estimates and genetic correlations across the assessment subgroups were high (see **Supplementary Table 4A and 4C**, respectively).

Supplementary Note 4: Results/Discussion: Detailed gene-findings

Here we discuss details of four genes with strong evidence in the present study and no prior GWAS-based reports: *PAX6*, *PROX2*, *VAMP2*, and *HMGN1*.

PAX6 (11p13) encodes paired box protein Pax-6, one of many human homologs of the Drosophila melanogaster gene *prd* (https://www.ncbi.nlm.nih.gov/gene/5080). The protein contains a homeobox domain in addition to the paired box domain, both of which bind DNA to regulate gene transcription. This protein is critical to the development of neural tissues, particularly the eye. Mutations in *PAX6* cause human ocular disorders like aniridia and Peter's anomaly (116). Pax6-derived progenitors in rodents reportedly generate subpopulations of amygdala neurons important for fear circuitry (117,118). *PAX6* mutant mice exhibit deficits in social interactions and fear-conditioned memory (119). Like *STAB1*, *PAX6* is also associated with neuroticism but no psychiatric disorders to date (120).

PROX2 (14q24.3) is a homeobox gene predicted to enable DNA-binding transcription factor activity (https://www.ncbi.nlm.nih.gov/gene/283571). Except for its role in regulating esophageal motility via its expression in vagal sensory neurons (121), knowledge of its role in human physiology and disease is limited.

VAMP2 (17p13.1) encodes *a* member of the vesicle-associated membrane protein (VAMP) / synaptobrevin family (https://www.ncbi.nlm.nih.gov/gene/6844). *VAMP2* is thought to participate in neurotransmitter release at a step between docking and fusion of synaptic vesicles with the presynaptic membrane. Mutations in this family of genes are associated with a variety of neuropsychiatric conditions including movement disorders, seizures, and neurodevelopmental disorders (122). Two prior studies in rodents support the relevance of *VAMP2* for ANX. The first found increased levels of hippocampal Vamp2 mRNA in rats subjected to a single prolonged stress paradigm (123). Another group studying gene targets of miR-153 reported a reduction in hippocampal expression of *VAMP2* as well as *Pclo*, *Snap25*, and *Trak2* in rats after contextual fear conditioning (124).

The final highly supported gene, *HMGN1* (21q22.2), encodes a protein that binds nucleosomal DNA and associated with transcriptionally active chromatin (https://www.ncbi.nlm.nih.gov/gene/3150). A study in mice found that Hmgn1 modulates the expression of the DNA-binding protein methyl CpG-binding protein 2. The authors linked changes in Hmgn1 levels to abnormalities in activity, anxiety, and social interactions (125).

Supplementary Note 5: Consortia

MVP

MVP Program Office

- Sumitra Muralidhar, Ph.D., Program Director
  US Department of Veterans Affairs, 810 Vermont Avenue NW, Washington, DC 20420
- Jennifer Moser, Ph.D., Associate Director, Scientific Programs
  US Department of Veterans Affairs, 810 Vermont Avenue NW, Washington, DC 20420
- Jennifer E. Deen, B.S., Associate Director, Cohort & Public Relations
  US Department of Veterans Affairs, 810 Vermont Avenue NW, Washington, DC 20420

MVP Executive Committee

- Co-Chair: Philip S. Tsao, Ph.D.
  VA Palo Alto Health Care System, 3801 Miranda Avenue, Palo Alto, CA 94304
- Co-Chair: Sumitra Muralidhar, Ph.D.
  US Department of Veterans Affairs, 810 Vermont Avenue NW, Washington, DC 20420
- J. Michael Gaziano, M.D., M.P.H.
  VA Boston Healthcare System, 150 S. Huntington Avenue, Boston, MA 02130
- Elizabeth Hauser, Ph.D.
  Durham VA Medical Center, 508 Fulton Street, Durham, NC 27705
- Amy Kilbourne, Ph.D., M.P.H.
  VA HSR&D, 2215 Fuller Road, Ann Arbor, MI 48105
- Michael Matheny, M.D., M.S., M.P.H.
  VA Tennessee Valley Healthcare System, 1310 24th Ave. South, Nashville, TN 37212
- Dave Oslin, M.D.
  Philadelphia VA Medical Center, 3900 Woodland Avenue, Philadelphia, PA 19104
- Deepak Voora, MD
  Durham VA Medical Center, 508 Fulton Street, Durham, NC 27705

MVP Co-Principal Investigators

- J. Michael Gaziano, M.D., M.P.H.
  VA Boston Healthcare System, 150 S. Huntington Avenue, Boston, MA 02130
- Philip S. Tsao, Ph.D.
  VA Palo Alto Health Care System, 3801 Miranda Avenue, Palo Alto, CA 94304

MVP Core Operations

- Jessica V. Brewer, M.P.H., Director, MVP Cohort Operations
  VA Boston Healthcare System, 150 S. Huntington Avenue, Boston, MA 02130
- Mary T. Brophy M.D., M.P.H., Director, VA Central Biorepository
  VA Boston Healthcare System, 150 S. Huntington Avenue, Boston, MA 02130
- Kelly Cho, M.P.H, Ph.D., Director, MVP Phenomics
  VA Boston Healthcare System, 150 S. Huntington Avenue, Boston, MA 02130
- Lori Churby, B.S., Director, MVP Regulatory Affairs
  VA Palo Alto Health Care System, 3801 Miranda Avenue, Palo Alto, CA 94304
- Scott L. DuVall, Ph.D., Director, VA Informatics and Computing Infrastructure (VINCI)
  VA Salt Lake City Health Care System, 500 Foothill Drive, Salt Lake City, UT 84148
- Saiju Pyarajan Ph.D., Director, Data and Computational Sciences
  VA Boston Healthcare System, 150 S. Huntington Avenue, Boston, MA 02130
- Robert Ringer, Pharm.D., Director, VA Albuquerque Central Biorepository
  New Mexico VA Health Care System, 1501 San Pedro Drive SE, Albuquerque, NM 87108
- Luis E. Selva, Ph.D., Director, MVP Biorepository Coordination
  VA Boston Healthcare System, 150 S. Huntington Avenue, Boston, MA 02130
- Shahpoor (Alex) Shayan, M.S., Director, MVP PRE Informatics
  VA Boston Healthcare System, 150 S. Huntington Avenue, Boston, MA 02130
- Brady Stephens, M.S., Principal Investigator, MVP Information Center
  Canandaigua VA Medical Center, 400 Fort Hill Avenue, Canandaigua, NY 14424
- Stacey B. Whitbourne, Ph.D., Director, MVP Cohort Development and Management
  VA Boston Healthcare System, 150 S. Huntington Avenue, Boston, MA 02130

MVP Publications and Presentations Committee

- Co-Chair: Themistocles L. Assimes, M.D., Ph. D
  VA Palo Alto Health Care System, 3801 Miranda Avenue, Palo Alto, CA 94304
- Co-Chair: Adriana Hung, M.D.; M.P.H
  VA Tennessee Valley Healthcare System, 1310 24th Ave. South, Nashville, TN 37212
- Co-Chair: Henry Kranzler, M.D.
  Philadelphia VA Medical Center, 3900 Woodland Avenue, Philadelphia, PA 19104

23andMe

The following members of the 23andMe Research Team contributed to this study:

Stella Aslibekyan, Adam Auton, Elizabeth Babalola, Robert K. Bell, Jessica Bielenberg, Ninad S. Chaudhary, Zayn Cochinwala, Sayantan Das, Emily DelloRusso, Payam Dibaeinia, Sarah L. Elson, Nicholas Eriksson, Chris Eijsbouts, Teresa Filshtein, Pierre Fontanillas, Davide Foletti, Will Freyman, Zach Fuller, Julie M. Granka, Chris German, Éadaoin Harney, Alejandro Hernandez, Barry Hicks, David A. Hinds, M. Reza Jabalameli, Ethan M. Jewett, Yunxuan Jiang, Sotiris Karagounis, Lucy Kaufmann, Matt Kmiecik, Katelyn Kukar, Alan Kwong, Keng-Han Lin, Yanyu Liang, Bianca A. Llamas, Aly Khan, Steven J. Micheletti, Matthew H. McIntyre, Meghan E. Moreno, Priyanka Nandakumar, Dominique T. Nguyen, Jared O'Connell, Steve Pitts, G. David Poznik, Alexandra Reynoso, Shubham Saini, Morgan Schumacher, Leah Selcer, Anjali J. Shastri, Jingchunzi Shi, Suyash Shringarpure, Keaton Stagaman, Teague Sterling, Qiaojuan Jane Su, Joyce Y. Tung, Susana A. Tat, Vinh Tran, Xin Wang, Wei Wang, Catherine H. Weldon, Amy L. Williams, Peter Wilton

FinnGen

The following members of the FinnGen Research Team contributed to this study:

Full Name	Affiliation	E-mail	Role 1	Role 2
Aarno Palotie	Institute for Molecular Medicine Finland (FIMM), HiLIFE, University of Helsinki, Helsinki, Finland; Broad Institute of MIT and Harvard; Massachusetts General Hospital, Boston, MA, United States	aarno.palotie@helsinki.fi	Steering Committee	Steering Committee
Mark Daly	Institute for Molecular Medicine Finland (FIMM), HiLIFE, University of Helsinki, Helsinki, Finland; Broad Institute of MIT and Harvard; Massachusetts General Hospital, Boston, MA, United States	mark.daly@helsinki.fi	Steering Committee	Steering Committee
Bridget Riley-Gills	Abbvie, Chicago, IL, United States	bridget.rileygillis@abbvie.com	Steering Committee	**Pharmaceutical companies**
Howard Jacob	Abbvie, Chicago, IL, United States	howard.jacob@abbvie.com	Steering Committee	**Pharmaceutical companies**
Coralie Viollet	Astra Zeneca, Cambridge, United Kingdom	coralie.viollet@astrazeneca.com	Steering Committee	**Pharmaceutical companies**
Slavé Petrovski	Astra Zeneca, Cambridge, United Kingdom	slav.petrovski@astrazeneca.com	Steering Committee	**Pharmaceutical companies**
Alix Berton	Bayer AG, Leverkusen, Germany	alix.berton@bayer.com	Steering Committee	**Pharmaceutical companies**
Santha Ramakrishnan	Bayer AG, Leverkusen, Germany	santha.ramakrishnan@bayer.com	Steering Committee	**Pharmaceutical companies**
Ellen Tsai	Biogen, Cambridge, MA, United States	ellen.tsai@biogen.com	Steering Committee	**Pharmaceutical companies**
Zhihao Ding	Boehringer Ingelheim, Ingelheim am Rhein, Germany	zhihao.ding@boehringer-ingelheim.com	Steering Committee	**Pharmaceutical companies**
Emily Holzinger	Bristol Myers Squibb, New York, NY, United States	emily.holzinger@bms.com	Steering Committee	**Pharmaceutical companies**
Robert Plenge	Bristol Myers Squibb, New York, NY, United States	robert.plenge@bms.com	Steering Committee	**Pharmaceutical companies**
Joseph Maranville	Bristol Myers Squibb, New York, NY, United States	joseph.maranville@bms.com	Steering Committee	**Pharmaceutical companies**
Mark McCarthy	Genentech, San Francisco, CA, United States	mccarthy.mark@gene.com	Steering Committee	**Pharmaceutical companies**
Rion Pendergrass	Genentech, San Francisco, CA, United States	penders2@gene.com	Steering Committee	**Pharmaceutical companies**
Jonathan Davitte	GlaxoSmithKline, Collegeville, PA, United States	jonathan.m.davitte@gsk.com	Steering Committee	**Pharmaceutical companies**

Simonne Longerich	Merck, Kenilworth, NJ, United States	simonne.longerich@merck.com	Steering Committee	**Pharmaceutical companies**
Anders Mälarstig	Pfizer, New York, NY, United States	anders.malarstig@pfizer.com	Steering Committee	**Pharmaceutical companies**
Anna Vlahiotis	Pfizer, New York, NY, United States	anna.vlahiotis@pfizer.com	Steering Committee	**Pharmaceutical companies**
Katherine Klinger	Translational Sciences, Sanofi R&D, Framingham, MA, USA	katherine.klinger@sanofi.com	Steering Committee	**Pharmaceutical companies**
Clement Chatelain	Translational Sciences, Sanofi R&D, Framingham, MA, USA	clement.chatelain@sanofi.com	Steering Committee	**Pharmaceutical companies**
Jorg Blankenstein	Translational Sciences, Sanofi R&D, Framingham, MA, USA	jorg.blankenstein@sanofi.com	Steering Committee	**Pharmaceutical companies**
Karol Estrada	Maze Therapeutics, San Francisco, CA, United States	kestrada@mazetx.com	Steering Committee	**Pharmaceutical companies**
Robert Graham	Maze Therapeutics, San Francisco, CA, United States	rgraham@mazetx.com	Steering Committee	**Pharmaceutical companies**
Dawn Waterworth	Johnson & Johnson Innovative Medicine, Spring House, PA, United States	dwaterwo@its.jnj.com	Steering Committee	**Pharmaceutical companies**
Chris O´Donnell	Novartis Institutes for BioMedical Research, Cambridge, MA, United States	chris.odonnell@novartis.com	Steering Committee	**Pharmaceutical companies**
Nicole Renaud	Novartis Institutes for BioMedical Research, Cambridge, MA, United States	nicole.renaud@novartis.com	Steering Committee	**Pharmaceutical companies**
Tomi P. Mäkelä	HiLIFE, University of Helsinki, Finland	tomi.makela@helsinki.fi	Steering Committee	**University of Helsinki & Biobanks**
Jaakko Kaprio	Institute for Molecular Medicine Finland (FIMM), HiLIFE, University of Helsinki, Helsinki, Finland	jaakko.kaprio@helsinki.fi	Steering Committee	**University of Helsinki & Biobanks**
Minna Ruddock	Arctic biobank / University of Oulu, Oulu, Finland	minna.ruddock@oulu.fi	Steering Committee	**University of Helsinki & Biobanks**
Petri Virolainen	Auria Biobank / University of Turku / Wellbeing Services County of Southwest Finland, Turku, Finland	petri.virolainen@tyks.fi	Steering Committee	**University of Helsinki & Biobanks**
Antti Hakanen	Auria Biobank / University of Turku / Wellbeing Services County of Southwest Finland, Turku, Finland	antti.hakanen@tyks.fi	Steering Committee	**University of Helsinki & Biobanks**
Terhi Kilpi	THL Biobank / Finnish Institute for Health and Welfare (THL), Helsinki, Finland	terhi.kilpi@thl.fi	Steering Committee	**University of Helsinki & Biobanks**
Markus Perola	THL Biobank / Finnish Institute for Health and Welfare (THL), Helsinki, Finland	markus.perola@thl.fi	Steering Committee	**University of Helsinki & Biobanks**
Jukka Partanen	Finnish Red Cross Blood Service / Finnish Hematology Registry and Clinical Biobank, Helsinki, Finland	jukka.partanen@veripalvelu.fi	Steering Committee	**University of Helsinki & Biobanks**
Taneli Raivio	Helsinki Biobank / Helsinki University and Hospital District of Helsinki and Uusimaa, Helsinki	taneli.raivio@hus.fi	Steering Committee	**University of Helsinki & Biobanks**
Raisa Serpi	Northern Finland Biobank Borealis / University of Oulu / Wellbeing services county of North Ostrobothnia, Oulu, Finland	raisa.serpi@pohde.fi	Steering Committee	**University of Helsinki & Biobanks**
Teija Kekonen	Northern Finland Biobank Borealis / University of Oulu / Wellbeing services county of North Ostrobothnia, Oulu, Finland	teija.kekonen@pohde.fi	Steering Committee	**University of Helsinki & Biobanks**
Kati Kristiansson	Finnish Clinical Biobank Tampere / University of Tampere / Wellbeing Services County of Pirkanmaa, Tampere, Finland	kati.kristiansson@pirha.fi	Steering Committee	**University of Helsinki & Biobanks**
Veli-Matti Kosma	Biobank of Eastern Finland / University of Eastern Finland / Wellbeing services county of North Savo, Kuopio, Finland	veli-matti.kosma@uef.fi	Steering Committee	**University of Helsinki & Biobanks**
Jari Laukkanen	Central Finland Biobank / University of Jyväskylä / Wellbeing Services County of Central Finland, Jyväskylä, Finland	jari.laukkanen@hyvaks.fi	Steering Committee	**University of Helsinki & Biobanks**
Tom Southerington	Finnish Biobank Cooperative - FINBB	tom.southerington@finbb.fi	Steering Committee	**University of Helsinki & Biobanks**
Outi Tuovila	Business Finland, Helsinki, Finland	outi.tuovila@businessfinland.fi	Steering Committee	**Other Experts/ Non-Voting Members**
Jeffrey Waring	Abbvie, Chicago, IL, United States	jeff.waring@abbvie.com	Scientific Committee	**Pharmaceutical companies**
Bridget Riley-Gillis	Abbvie, Chicago, IL, United States	bridget.rileygillis@abbvie.com	Scientific Committee	**Pharmaceutical companies**
Fedik Rahimov	Abbvie, Chicago, IL, United States	fedik.rahimov@abbvie.com	Scientific Committee	**Pharmaceutical companies**
Ioanna Tachmazidou	Astra Zeneca, Cambridge, United Kingdom	ioanna.tachmazidou@astrazeneca.com	Scientific Committee	**Pharmaceutical companies**
Alix Berton	Bayer AG, Leverkusen, Germany	alix.berton@bayer.com	Scientific Committee	**Pharmaceutical companies**
Santha Ramakrishnan	Bayer AG, Leverkusen, Germany	santha.ramakrishnan@bayer.com	Scientific Committee	**Pharmaceutical companies**
Ellen Tsai	Biogen, Cambridge, MA, United States	ellen.tsai@biogen.com	Scientific Committee	**Pharmaceutical companies**
Zhihao Ding	Boehringer Ingelheim, Ingelheim am Rhein, Germany	zhihao.ding@boehringer-ingelheim.com	Scientific Committee	**Pharmaceutical companies**
Marc Jung	Boehringer Ingelheim, Ingelheim am Rhein, Germany	marc_oliver.jung@boehringer-ingelheim.com	Scientific Committee	**Pharmaceutical companies**
Hanati Tuoken	Boehringer Ingelheim, Ingelheim am Rhein, Germany	hanati.tuoken@boehringer-ingelheim.com	Scientific Committee	**Pharmaceutical companies**

Shameek Biswas	Bristol Myers Squibb, New York, NY, United States	Shameek.Biswas@bms.com	Scientific Committee	**Pharmaceutical companies**
Benjamin Sun	Bristol Myers Squibb, New York, NY, United States	Benjamin.Sun@bms.com	Scientific Committee	**Pharmaceutical companies**
Rion Pendergrass	Genentech, San Francisco, CA, United States	penders2@gene.com	Scientific Committee	**Pharmaceutical companies**
Jonathan Davitte	GlaxoSmithKline, Collegeville, PA, United States	jonathan.m.davitte@gsk.com	Scientific Committee	**Pharmaceutical companies**
Neha Raghavan	Merck, Kenilworth, NJ, United States	neha.raghavan@merck.com	Scientific Committee	**Pharmaceutical companies**
Adriana Huertas-Vazquez	Merck, Kenilworth, NJ, United States	adriana.huertas.vazquez@merck.co m	Scientific Committee	**Pharmaceutical companies**
Jae-Hoon Sul	Merck, Kenilworth, NJ, United States	jae.hoon.sul@merck.com	Scientific Committee	**Pharmaceutical companies**
Anders Mälarstig	Pfizer, New York, NY, United States	anders.malarstig@pfizer.com	Scientific Committee	**Pharmaceutical companies**
Xinli Hu	Pfizer, New York, NY, United States	xinli.hu@pfizer.com	Scientific Committee	**Pharmaceutical companies**
Åsa Hedman	Pfizer, New York, NY, United States	asa.hedman@pfizer.com	Scientific Committee	**Pharmaceutical companies**
Katherine Klinger	Translational Sciences, Sanofi R&D, Framingham, MA, USA	katherine.klinger@sanofi.com	Scientific Committee	**Pharmaceutical companies**
Robert Graham	Maze Therapeutics, San Francisco, CA, United States	rgraham@mazetx.com	Scientific Committee	**Pharmaceutical companies**
Dawn Waterworth	Johnson & Johnson Innovative Medicine, Spring House, PA, United States	dwaterwo@its.jnj.com	Scientific Committee	**Pharmaceutical companies**
Nicole Renaud	Novartis Institutes for BioMedical Research, Cambridge, MA, United States	nicole.renaud@novartis.com	Scientific Committee	**Pharmaceutical companies**
Ma´en Obeidat	Novartis Institutes for BioMedical Research, Cambridge, MA, United States	maen.obeidat@novartis.com	Scientific Committee	**Pharmaceutical companies**
Jonathan Chung	Novartis Institutes for BioMedical Research, Cambridge, MA, United States	jonathan.chung@novartis.com	Scientific Committee	**Pharmaceutical companies**
Jonas Zierer	Novartis Institutes for BioMedical Research, Cambridge, MA, United States	jonas.zierer@novartis.com	Scientific Committee	**Pharmaceutical companies**
Mari Niemi	Novartis Institutes for BioMedical Research, Cambridge, MA, United States	mari.niemi@novartis.com	Scientific Committee	**Pharmaceutical companies**
Samuli Ripatti	Institute for Molecular Medicine Finland (FIMM), HiLIFE, University of Helsinki, Helsinki, Finland	samuli.ripatti@helsinki.fi	Scientific Committee	**University of Helsinki & Biobanks**
Johanna Schleutker	Auria Biobank / University of Turku / Wellbeing Services County of Southwest Finland, Turku, Finland	johanna.schleutker@utu.fi	Scientific Committee	**University of Helsinki & Biobanks**
Markus Perola	THL Biobank / Finnish Institute for Health and Welfare (THL), Helsinki, Finland	markus.perola@thl.fi	Scientific Committee	**University of Helsinki & Biobanks**
Mikko Arvas	Finnish Red Cross Blood Service / Finnish Hematology Registry and Clinical Biobank, Helsinki, Finland	mikko.arvas@veripalvelu.fi	Scientific Committee	**University of Helsinki & Biobanks**
Olli Carpén	Helsinki Biobank / Helsinki University and Hospital District of Helsinki and Uusimaa, Helsinki	olli.carpen@helsinki.fi	Scientific Committee	**University of Helsinki & Biobanks**
Reetta Hinttala	Northern Finland Biobank Borealis / University of Oulu / Wellbeing services county of North Ostrobothnia, Oulu, Finland	reetta.hinttala@oulu.fi	Scientific Committee	**University of Helsinki & Biobanks**
Johannes Kettunen	Northern Finland Biobank Borealis / University of Oulu / Wellbeing services county of North Ostrobothnia, Oulu, Finland	johannes.kettunen@oulu.fi	Scientific Committee	**University of Helsinki & Biobanks**
Arto Mannermaa	Biobank of Eastern Finland / University of Eastern Finland / Wellbeing services county of North Savo, Kuopio, Finland	arto.mannermaa@uef.fi	Scientific Committee	**University of Helsinki & Biobanks**
Katriina Aalto-Setälä	Faculty of Medicine and Health Technology, Tampere University, Tampere, Finland	katriina.aalto-setala@tuni.fi	Scientific Committee	**University of Helsinki & Biobanks**
Mika Kähönen	Finnish Clinical Biobank Tampere / University of Tampere / Wellbeing Services County of Pirkanmaa, Tampere, Finland	mika.kahonen@uta.fi	Scientific Committee	**University of Helsinki & Biobanks**
Jari Laukkanen	Central Finland Biobank / University of Jyväskylä / Wellbeing Services County of Central Finland, Jyväskylä, Finland	jari.laukkanen@hyvaks.fi	Scientific Committee	**University of Helsinki & Biobanks**
Johanna Mäkelä	FINBB - Finnish biobank cooperative	johanna.makela@finbb.fi	Scientific Committee	**University of Helsinki & Biobanks**
Lila Kallio	Auria Biobank / University of Turku / Wellbeing Services County of Southwest Finland, Turku, Finland	Lila.Kallio@tyks.fi	Biobank directors	Biobank directors
Tiina Wahlfors	THL Biobank / Finnish Institute for Health and Welfare (THL), Helsinki, Finland	tiina.wahlfors@thl.fi	Biobank directors	Biobank directors
Jukka Partanen	Finnish Red Cross Blood Service / Finnish Hematology Registry and Clinical Biobank, Helsinki, Finland	jukka.partanen@veripalvelu.fi	Biobank directors	Biobank directors
Eero Punkka	Helsinki Biobank / Helsinki University and Hospital District of Helsinki and Uusimaa, Helsinki	eero.punkka@hus.fi	Biobank directors	Biobank directors
Raisa Serpi	Northern Finland Biobank Borealis / University of Oulu / Wellbeing services county of North Ostrobothnia, Oulu, Finland	raisa.serpi@pohde.fi	Biobank directors	Biobank directors

Sanna Siltanen	Finnish Clinical Biobank Tampere / University of Tampere / Wellbeing Services County of Pirkanmaa, Tampere, Finland	sanna.siltanen@pirha.fi	**Biobank directors**	**Biobank directors**
Veli-Matti Kosma	Biobank of Eastern Finland / University of Eastern Finland / Wellbeing services county of North Savo, Kuopio, Finland	veli-matti.kosma@uef.fi	**Biobank directors**	**Biobank directors**
Tiina Jokela	Central Finland Biobank / University of Jyväskylä / Wellbeing Services County of Central Finland, Jyväskylä, Finland	tiina.a.jokela@jyu.fi	**Biobank directors**	**Biobank directors**
Anu Jalanko	Institute for Molecular Medicine Finland (FIMM), HiLIFE, University of Helsinki, Helsinki, Finland	anu.jalanko@helsinki.fi	**FinnGen Teams**	**Administration**
Auli Toivola	Institute for Molecular Medicine Finland (FIMM), HiLIFE, University of Helsinki, Helsinki, Finland	auli.toivola@helsinki.fi	**FinnGen Teams**	**Administration**
Denise Öller	Institute for Molecular Medicine Finland (FIMM), HiLIFE, University of Helsinki, Helsinki, Finland	denise.oller@helsinki.fi	**FinnGen Teams**	**Administration**
Helen Cooper	Institute for Molecular Medicine Finland (FIMM), HiLIFE, University of Helsinki, Helsinki, Finland	helen.cooper@helsinki.fi	**FinnGen Teams**	**Administration**
Mervi Aavikko	Institute for Molecular Medicine Finland (FIMM), HiLIFE, University of Helsinki, Helsinki, Finland	mervi.aavikko@helsinki.fi	**FinnGen Teams**	**Administration**
Risto Kajanne	Institute for Molecular Medicine Finland (FIMM), HiLIFE, University of Helsinki, Helsinki, Finland	risto.kajanne@helsinki.fi	**FinnGen Teams**	**Administration**
Rodos Rodosthenous	Institute for Molecular Medicine Finland (FIMM), HiLIFE, University of Helsinki, Helsinki, Finland	rodos.rodosthenous@helsinki.fi	**FinnGen Teams**	**Administration**
Sofia Kuitunen	University of Helsinki, Helsinki, Finland	sofia.kuitunen@helsinki.fi	**FinnGen Teams**	**Administration**
Tarja Laitinen	Institute for Molecular Medicine Finland (FIMM), HiLIFE, University of Helsinki, Helsinki, Finland	tarja.laitinen@helsinki.fi	**FinnGen Teams**	**Administration**
Arto Lehisto	Institute for Molecular Medicine Finland (FIMM), HiLIFE, University of Helsinki, Helsinki, Finland	arto.lehisto@helsinki.fi	**FinnGen Teams**	**Analysis**
Hafiz Sikandar	Institute for Molecular Medicine Finland (FIMM), HiLIFE, University of Helsinki, Helsinki, Finland	hafiz.sikandar@helsinki.fi	**FinnGen Teams**	**Analysis**
Juha Karjalainen	Institute for Molecular Medicine Finland (FIMM), HiLIFE, University of Helsinki, Helsinki, Finland	juha.karjalainen@helsinki.fi	**FinnGen Teams**	**Analysis**
Juha Mehtonen	Institute for Molecular Medicine Finland (FIMM), HiLIFE, University of Helsinki, Helsinki, Finland	juha.mehtonen@helsinki.fi	**FinnGen Teams**	**Analysis**
Masahiro Kanai	Broad Institute, Cambridge, MA, United States	mkanai@broadinstitute.org	**FinnGen Teams**	**Analysis**
Mitja Kurki	Institute for Molecular Medicine Finland (FIMM), HiLIFE, University of Helsinki, Helsinki, Finland; Broad Institute, Cambridge, MA, United States	mkurki@broadinstitute.org	**FinnGen Teams**	**Analysis**
Mutaamba Maasha	Broad Institute, Cambridge, MA, United States	mmaasha@broadinstitute.org	**FinnGen Teams**	**Analysis**
Pietro Della Briotta Parolo	Institute for Molecular Medicine Finland (FIMM), HiLIFE, University of Helsinki, Helsinki, Finland	pietro.dellabriottaparolo@helsinki.f i	**FinnGen Teams**	**Analysis**
Samuel Jones	Institute for Molecular Medicine Finland (FIMM), HiLIFE, University of Helsinki, Helsinki, Finland	samuel.jones@helsinki.fi	**FinnGen Teams**	**Analysis**
Sanni Ruotsalainen	Institute for Molecular Medicine Finland (FIMM), HiLIFE, University of Helsinki, Helsinki, Finland	sanni.ruotsalainen@helsinki.fi	**FinnGen Teams**	**Analysis**
Susanna Lemmelä	Institute for Molecular Medicine Finland (FIMM), HiLIFE, University of Helsinki, Helsinki, Finland	susanna.lemmela@helsinki.fi	**FinnGen Teams**	**Analysis**
Wei Zhou	Broad Institute, Cambridge, MA, United States	wzhou@broadinstitute.org	**FinnGen Teams**	**Analysis**
Aki Havulinna	Finnish Institute for Health and Welfare (THL), Helsinki, Finland	aki.havulinna@thl.fi	**FinnGen Teams**	**Clinical Endpoint Development**
L. Elisa Lahtela	Institute for Molecular Medicine Finland (FIMM), HiLIFE, University of Helsinki, Helsinki, Finland	laura.lahtela@helsinki.fi	**FinnGen Teams**	**Clinical Endpoint Development**
Mari Kaunisto	Institute for Molecular Medicine Finland (FIMM), HiLIFE, University of Helsinki, Helsinki, Finland	mari.kaunisto@helsinki.fi	**FinnGen Teams**	**Communication**
Awaisa Ghazal	Institute for Molecular Medicine Finland (FIMM), HiLIFE, University of Helsinki, Helsinki, Finland	awaisa.ghazal@helsinki.fi	**FinnGen Teams**	**E-Science**
Elina Kilpeläinen	Institute for Molecular Medicine Finland (FIMM), HiLIFE, University of Helsinki, Helsinki, Finland	elina.kilpelainen@helsinki.fi	**FinnGen Teams**	**E-Science**
Jaska Uimonen	Institute for Molecular Medicine Finland (FIMM), HiLIFE, University of Helsinki, Helsinki, Finland	jaska.uimonen@helsinki.fi	**FinnGen Teams**	**E-Science**
Oluwaseun Alexander Dada	Institute for Molecular Medicine Finland (FIMM), HiLIFE, University of Helsinki, Helsinki, Finland	alexander.dada@helsinki.fi	**FinnGen Teams**	**E-Science**
Rigbe Weldatsadik	Institute for Molecular Medicine Finland (FIMM), HiLIFE, University of Helsinki, Helsinki, Finland	rigbe.weldatsadik@helsinki.fi	**FinnGen Teams**	**E-Science**
Sanni Ruotsalainen	Institute for Molecular Medicine Finland (FIMM), HiLIFE, University of Helsinki, Helsinki, Finland	sanni.ruotsalainen@helsinki.fi	**FinnGen Teams**	**E-Science**
Tianduanyi Wang	Institute for Molecular Medicine Finland (FIMM), HiLIFE, University of Helsinki, Helsinki, Finland	tianduanyi.wang@helsinki.fi	**FinnGen Teams**	**E-Science**
Timo P. Sipilä	Institute for Molecular Medicine Finland (FIMM), HiLIFE, University of Helsinki, Helsinki, Finland	timo.p.sipila@helsinki.fi	**FinnGen Teams**	**E-Science**
Kati Donner	Institute for Molecular Medicine Finland (FIMM), HiLIFE, University of Helsinki, Helsinki, Finland	kati.donner@helsinki.fi	**FinnGen Teams**	**Genotyping**
Anu Loukola	Helsinki Biobank / Helsinki University and Hospital District of Helsinki and Uusimaa, Helsinki	anu.loukola@hus.fi	**FinnGen Teams**	**Sample Collection Coordination**

Päivi Ingalsuo	THL Biobank / Finnish Institute for Health and Welfare (THL), Helsinki, Finland	paivi.ingalsuo@thl.fi	FinnGen Teams	**Sample Logistics**
Arto Pietilä	THL Biobank / Finnish Institute for Health and Welfare (THL), Helsinki, Finland	arto.pietila@thl.fi	FinnGen Teams	**Registry Data Operations**
Sami Koskelainen	THL Biobank / Finnish Institute for Health and Welfare (THL), Helsinki, Finland	sami.koskelainen@thl.fi	FinnGen Teams	**Registry Data Operations**
Susanna Lemmelä	Institute for Molecular Medicine Finland (FIMM), HiLIFE, University of Helsinki, Helsinki, Finland	susanna.lemmela@helsinki.fi	FinnGen Teams	**Registry Data Operations**
Teemu Paajanen	THL Biobank / Finnish Institute for Health and Welfare (THL), Helsinki, Finland	teemu.paajanen@thl.fi	FinnGen Teams	**Registry Data Operations**
Tero Hiekkalinna	THL Biobank / Finnish Institute for Health and Welfare (THL), Helsinki, Finland	tero.hiekkalinna@helsinki.fi	FinnGen Teams	**Registry Data Operations**
Priit Palta	Institute for Molecular Medicine Finland (FIMM), HiLIFE, University of Helsinki, Helsinki, Finland	priit.palta@helsinki.fi	FinnGen Teams	**Sequencing Informatics**
Dawit A. Yohannes	Institute for Molecular Medicine Finland (FIMM), HiLIFE, University of Helsinki, Helsinki, Finland	dawit.yohannes@helsinki.fi	FinnGen Teams	**Phenotype team**
Harri Siirtola	University of Tampere, Tampere, Finland	harri.siirtola@tuni.fi	FinnGen Teams	**Phenotype team**
Javier Gracia-Tabuenca	University of Tampere, Tampere, Finland	javier.graciatabuenca@tuni.fi	FinnGen Teams	**Phenotype team**
Marika Kaakinen	Institute for Molecular Medicine Finland (FIMM), HiLIFE, University of Helsinki, Helsinki, Finland	marika.kaakinen@helsinki.fi	FinnGen Teams	**Phenotype team**
Mary Pat Reeve	Institute for Molecular Medicine Finland (FIMM), HiLIFE, University of Helsinki, Helsinki, Finland	mary.reeve@helsinki.fi	FinnGen Teams	**Phenotype team**
Shanmukha Sampath Padmanabhuni	Institute for Molecular Medicine Finland (FIMM), HiLIFE, University of Helsinki, Helsinki, Finland	sam.padmanabhuni@helsinki.fi	FinnGen Teams	**Phenotype team**
Shuang Luo	Institute for Molecular Medicine Finland (FIMM), HiLIFE, University of Helsinki, Helsinki, Finland	shuang.luo@helsinki.fi	FinnGen Teams	**Phenotype team**
Vincent Llorens	Institute for Molecular Medicine Finland (FIMM), HiLIFE, University of Helsinki, Helsinki, Finland	vincent.llorens@helsinki.fi	FinnGen Teams	**Phenotype team**
Iina Laak	Institute for Molecular Medicine Finland (FIMM), HiLIFE, University of Helsinki, Helsinki, Finland	iina.laak@helsinki.fi	FinnGen Teams	**Data protection officer**
Jaakko Tyrmi	University of Oulu, Oulu, Finland / University of Tampere, Tampere, Finland	jaakko.tyrmi@oulu.fi	FinnGen Teams	**FinnGen local support person**
Janne Isojärvi	University of Turku, Turku, Finland	jaheis@utu.fi	FinnGen Teams	**FinnGen local support person**
Tero Sievänen	University of Eastern Finland, Kuopio, Finland	tero.sievanen@uef.fi	FinnGen Teams	**FinnGen local support person**
Timo Pohjonen	University of Jyväskylä, Jyväskylä, Finland	timo.pohjonen@hyvaks.fi	FinnGen Teams	**FinnGen local support person**
Vidal Fey	University of Tampere, Tampere, Finland	vidal.fey@tuni.fi	FinnGen Teams	**FinnGen local support person**
Johanna Mäkelä	Finnish Biobank Cooperative - FINBB	johanna.makela@finbb.fi	FinnGen Teams	**FINBB - Finnish biobank cooperative**
Pauli Wihuri	Finnish Biobank Cooperative - FINBB	pauli.wihuri@finbb.fi	FinnGen Teams	**FINBB - Finnish biobank cooperative**
Tom Southerington	Finnish Biobank Cooperative - FINBB	tom.southerington@finbb.fi	FinnGen Teams	**FINBB - Finnish biobank cooperative**
Meri Lähteenmäki	Finnish Biobank Cooperative - FINBB	meri.lahteenmaki@finbb.fi	FinnGen Teams	**FINBB - Finnish biobank cooperative**
Reetta Kälviäinen	Wellbeing services county of North Savo, Kuopio, Finland	reetta.kalviainen@kuh.fi	Clinical Groups (FinnGen phases 1&2)	**Neurology Group**
Valtteri Julkunen	Wellbeing services county of North Savo, Kuopio, Finland	valtteri.julkunen@kuh.fi	Clinical Groups (FinnGen phases 1&2)	**Neurology Group**
Hilkka Soininen	Wellbeing services county of North Savo, Kuopio, Finland	hilkka.soininen@uef.fi	Clinical Groups (FinnGen phases 1&2)	**Neurology Group**
Anne Remes	Wellbeing services county of North Ostrobothnia, Oulu, Finland	anne.remes@oulu.fi	Clinical Groups (FinnGen phases 1&2)	**Neurology Group**
Mikko Hiltunen	University of Eastern Finland, Kuopio, Finland	mikko.hiltunen@uef.fi	Clinical Groups (FinnGen phases 1&2)	**Neurology Group**
Jukka Peltola	Wellbeing Services County of Pirkanmaa, Tampere, Finland	jukka.peltola@pirha.fi	Clinical Groups (FinnGen phases 1&2)	**Neurology Group**
Minna Raivio	Hospital District of Helsinki and Uusimaa, Helsinki, Finland	minna.raivio@geri.fi	Clinical Groups (FinnGen phases 1&2)	**Neurology Group**
Pentti Tienari	Hospital District of Helsinki and Uusimaa, Helsinki, Finland	pentti.tienari@hus.fi	Clinical Groups (FinnGen phases 1&2)	**Neurology Group**
Juha Rinne	Wellbeing Services County of Southwest Finland, Turku, Finland	juha.rinne@tyks.fi	Clinical Groups (FinnGen phases 1&2)	**Neurology Group**
Roosa Kallionpää	Wellbeing Services County of Southwest Finland, Turku, Finland	roosa.kallionpaa@tyks.fi	Clinical Groups (FinnGen phases 1&2)	**Neurology Group**
Juulia Partanen	Institute for Molecular Medicine Finland, HiLIFE, University of Helsinki, Finland	juulia.partanen@helsinki.fi	Clinical Groups (FinnGen phases 1&2)	**Neurology Group**
Adam Ziemann	Abbvie, Chicago, IL, United States	adam.ziemann@abbvie.com	Clinical Groups (FinnGen phases 1&2)	**Neurology Group**
Nizar Smaoui	Abbvie, Chicago, IL, United States	nizar.smaoui@abbvie.com	Clinical Groups (FinnGen phases 1&2)	**Neurology Group**
Anne Lehtonen	Abbvie, Chicago, IL, United States	anne.lehtonen@abbvie.com	Clinical Groups (FinnGen phases 1&2)	**Neurology Group**

Susan Eaton	Biogen, Cambridge, MA, United States	susan.eaton@biogen.com	Clinical Groups (FinnGen phases 1&2)	**Neurology Group**
Shameek Biswas	Bristol Myers Squibb, New York, NY, United States	shameek.biswas@bms.com	Clinical Groups (FinnGen phases 1&2)	**Neurology Group**
Natalie Bowers	Genentech, San Francisco, CA, United States	bowersn1@gene.com	Clinical Groups (FinnGen phases 1&2)	**Neurology Group**
Edmond Teng	Genentech, San Francisco, CA, United States	teng.edmond@gene.com	Clinical Groups (FinnGen phases 1&2)	**Neurology Group**
Rion Pendergrass	Genentech, San Francisco, CA, United States	penders2@gene.com	Clinical Groups (FinnGen phases 1&2)	**Neurology Group**
Fanli Xu	GlaxoSmithKline, Brentford, United Kingdom	chun-fang.2.xu@gsk.com	Clinical Groups (FinnGen phases 1&2)	**Neurology Group**
Laura Addis	GlaxoSmithKline, Brentford, United Kingdom	laura.x.addis@gsk.com	Clinical Groups (FinnGen phases 1&2)	**Neurology Group**
John Eicher	GlaxoSmithKline, Brentford, United Kingdom	john.d.eicher@gsk.com	Clinical Groups (FinnGen phases 1&2)	**Neurology Group**
Qingqin S Li	Johnson & Johnson Innovative Medicine, Titusville, NJ 08560, United States	QLi2@its.jnj.com	Clinical Groups (FinnGen phases 1&2)	**Neurology Group**
Karen He	Johnson & Johnson Innovative Medicine, Spring House, PA, United States	khe2@its.jnj.com	Clinical Groups (FinnGen phases 1&2)	**Neurology Group**
Ekaterina Khramtsova	Johnson & Johnson Innovative Medicine, Spring House, PA, United States	ekhramts@its.jnj.com	Clinical Groups (FinnGen phases 1&2)	**Neurology Group**
Neha Raghavan	Merck, Kenilworth, NJ, United States	neha.raghavan@merck.com	Clinical Groups (FinnGen phases 1&2)	**Neurology Group**
Martti Färkkilä	Hospital District of Helsinki and Uusimaa, Helsinki, Finland	martti.farkkila@hus.fi	Clinical Groups (FinnGen phases 1&2)	**Gastroenterology Group**
Jukka Koskela	Hospital District of Helsinki and Uusimaa, Helsinki, Finland	jukka.koskela@helsinki.fi	Clinical Groups (FinnGen phases 1&2)	**Gastroenterology Group**
Sampsa Pikkarainen	Hospital District of Helsinki and Uusimaa, Helsinki, Finland	sampsa.pikkarainen@hus.fi	Clinical Groups (FinnGen phases 1&2)	**Gastroenterology Group**
Airi Jussila	Wellbeing Services County of Pirkanmaa, Tampere, Finland	airi.jussila@pirha.fi	Clinical Groups (FinnGen phases 1&2)	**Gastroenterology Group**
Katri Kaukinen	Wellbeing Services County of Pirkanmaa, Tampere, Finland	katri.kaukinen@tuni.fi	Clinical Groups (FinnGen phases 1&2)	**Gastroenterology Group**
Timo Blomster	Wellbeing services county of North Ostrobothnia, Oulu, Finland	timo.blomster@pohde.fi	Clinical Groups (FinnGen phases 1&2)	**Gastroenterology Group**
Mikko Kiviniemi	Wellbeing services county of North Savo, Kuopio, Finland	mikko.kiviniemi@kuh.fi	Clinical Groups (FinnGen phases 1&2)	**Gastroenterology Group**
Markku Voutilainen	Wellbeing Services County of Southwest Finland, Turku, Finland	markku.voutilainen@tyks.fi	Clinical Groups (FinnGen phases 1&2)	**Gastroenterology Group**
Mark Daly	Institute for Molecular Medicine Finland (FIMM), HiLIFE, University of Helsinki, Helsinki, Finland; Broad Institute of MIT and Harvard; Massachusetts General Hospital, Boston, MA, United States	mark.daly@helsinki.fi	Clinical Groups (FinnGen phases 1&2)	**Gastroenterology Group**
Jeffrey Waring	Abbvie, Chicago, IL, United States	jeff.waring@abbvie.com	Clinical Groups (FinnGen phases 1&2)	**Gastroenterology Group**
Nizar Smaoui	Abbvie, Chicago, IL, United States	nizar.smaoui@abbvie.com	Clinical Groups (FinnGen phases 1&2)	**Gastroenterology Group**
Fedik Rahimov	Abbvie, Chicago, IL, United States	fedik.rahimov@abbvie.com	Clinical Groups (FinnGen phases 1&2)	**Gastroenterology Group**
Anne Lehtonen	Abbvie, Chicago, IL, United States	anne.lehtonen@abbvie.com	Clinical Groups (FinnGen phases 1&2)	**Gastroenterology Group**
Tim Lu	Genentech, San Francisco, CA, United States	lut8@gene.com	Clinical Groups (FinnGen phases 1&2)	**Gastroenterology Group**
Natalie Bowers	Genentech, San Francisco, CA, United States	bowersn1@gene.com	Clinical Groups (FinnGen phases 1&2)	**Gastroenterology Group**
Rion Pendergrass	Genentech, San Francisco, CA, United States	penders2@gene.com	Clinical Groups (FinnGen phases 1&2)	**Gastroenterology Group**
Linda McCarthy	GlaxoSmithKline, Brentford, United Kingdom	linda.c.mccarthy@gsk.com	Clinical Groups (FinnGen phases 1&2)	**Gastroenterology Group**
Amy Hart	Johnson & Johnson Innovative Medicine, Spring House, PA, United States	ahart13@its.jnj.com	Clinical Groups (FinnGen phases 1&2)	**Gastroenterology Group**
Meijian Guan	Johnson & Johnson Innovative Medicine, Spring House, PA, United States	mguan4@its.jnj.com	Clinical Groups (FinnGen phases 1&2)	**Gastroenterology Group**
Jason Miller	Merck, Kenilworth, NJ, United States	jason.miller4@merck.com	Clinical Groups (FinnGen phases 1&2)	**Gastroenterology Group**
Kirsi Kalpala	Pfizer, New York, NY, United States	kirsi.kalpala@pfizer.com	Clinical Groups (FinnGen phases 1&2)	**Gastroenterology Group**
Melissa Miller	Pfizer, New York, NY, United States	melissa.r.miller@pfizer.com	Clinical Groups (FinnGen phases 1&2)	**Gastroenterology Group**
Xinli Hu	Pfizer, New York, NY, United States	xinli.hu@pfizer.com	Clinical Groups (FinnGen phases 1&2)	**Gastroenterology Group**
Kari Eklund	Hospital District of Helsinki and Uusimaa, Helsinki, Finland	kari.eklund@hus.fi	Clinical Groups (FinnGen phases 1&2)	**Rheumatology Group**
Antti Palomäki	Wellbeing Services County of Southwest Finland, Turku, Finland	ajpalo@utu.fi	Clinical Groups (FinnGen phases 1&2)	**Rheumatology Group**

Pia Isomäki	Wellbeing Services County of Pirkanmaa, Tampere, Finland	pia.isomaki@pirha.fi	Clinical Groups (FinnGen phases 1&2)	**Rheumatology Group**
Laura Pirilä	Wellbeing Services County of Southwest Finland, Turku, Finland	laura.pirila@fimnet.fi,laura.pirila@tyks.fi	Clinical Groups (FinnGen phases 1&2)	**Rheumatology Group**
Oili Kaipiainen-Seppänen	Wellbeing services county of North Savo, Kuopio, Finland	oili.kaipiainen-seppanen@kuh.fi	Clinical Groups (FinnGen phases 1&2)	**Rheumatology Group**
Johanna Huhtakangas	Wellbeing services county of North Ostrobothnia, Oulu, Finland	johanna.huhtakangas@kuh.fi	Clinical Groups (FinnGen phases 1&2)	**Rheumatology Group**
Nina Mars	Institute for Molecular Medicine Finland (FIMM), HiLIFE, University of Helsinki, Helsinki, Finland	nina.mars@helsinki.fi	Clinical Groups (FinnGen phases 1&2)	**Rheumatology Group**
Jeffrey Waring	Abbvie, Chicago, IL, United States	jeff.waring@abbvie.com	Clinical Groups (FinnGen phases 1&2)	**Rheumatology Group**
Fedik Rahimov	Abbvie, Chicago, IL, United States	fedik.rahimov@abbvie.com	Clinical Groups (FinnGen phases 1&2)	**Rheumatology Group**
Apinya Lertratanakul	Abbvie, Chicago, IL, United States	apinya.lertratanakul@abbvie.com	Clinical Groups (FinnGen phases 1&2)	**Rheumatology Group**
Nizar Smaoui	Abbvie, Chicago, IL, United States	nizar.smaoui@abbvie.com	Clinical Groups (FinnGen phases 1&2)	**Rheumatology Group**
Anne Lehtonen	Abbvie, Chicago, IL, United States	anne.lehtonen@abbvie.com	Clinical Groups (FinnGen phases 1&2)	**Rheumatology Group**
Coralie Viollet	AstraZeneca, Cambridge, United Kingdom	coralie.viollet@astrazeneca.com	Clinical Groups (FinnGen phases 1&2)	**Rheumatology Group**
Marla Hochfeld	Bristol Myers Squibb, New York, NY, United States	mhochfeld@celgene.com	Clinical Groups (FinnGen phases 1&2)	**Rheumatology Group**
Natalie Bowers	Genentech, San Francisco, CA, United States	bowersn1@gene.com	Clinical Groups (FinnGen phases 1&2)	**Rheumatology Group**
Rion Pendergrass	Genentech, San Francisco, CA, United States	penders2@gene.com	Clinical Groups (FinnGen phases 1&2)	**Rheumatology Group**
Jorge Esparza Gordillo	GlaxoSmithKline, Brentford, United Kingdom	jorge.x.esparza-gordillo@gsk.com	Clinical Groups (FinnGen phases 1&2)	**Rheumatology Group**
Dawn Waterworth	Johnson & Johnson Innovative Medicine, Spring House, PA, United States	dwaterwo@its.jnj.com	Clinical Groups (FinnGen phases 1&2)	**Rheumatology Group**
Fabiana Farias	Merck, Kenilworth, NJ, United States	fabiana.farias@merck.com	Clinical Groups (FinnGen phases 1&2)	**Rheumatology Group**
Kirsi Kalpala	Pfizer, New York, NY, United States	kirsi.kalpala@pfizer.com	Clinical Groups (FinnGen phases 1&2)	**Rheumatology Group**
Nan Bing	Pfizer, New York, NY, United States	nan.bing@pfizer.com	Clinical Groups (FinnGen phases 1&2)	**Rheumatology Group**
Xinli Hu	Pfizer, New York, NY, United States	xinli.hu@pfizer.com	Clinical Groups (FinnGen phases 1&2)	**Rheumatology Group**
Tarja Laitinen	Wellbeing Services County of Pirkanmaa, Tampere, Finland	tarja.laitinen@pirha.fi	Clinical Groups (FinnGen phases 1&2)	**Pulmonology Group**
Margit Pelkonen	Wellbeing services county of North Savo, Kuopio, Finland	margit.pelkonen@kuh.fi	Clinical Groups (FinnGen phases 1&2)	**Pulmonology Group**
Paula Kauppi	Hospital District of Helsinki and Uusimaa, Helsinki, Finland	paula.kauppi@hus.fi	Clinical Groups (FinnGen phases 1&2)	**Pulmonology Group**
Hannu Kankaanranta	University of Gothenburg, Gothenburg, Sweden/ Seinäjoki Central Hospital, Seinäjoki, Finland/ Tampere University, Tampere, Finland	hannu.kankaanranta@tuni.fi	Clinical Groups (FinnGen phases 1&2)	**Pulmonology Group**
Terttu Harju	Wellbeing services county of North Ostrobothnia, Oulu, Finland	terttu.harju@oulu.fi	Clinical Groups (FinnGen phases 1&2)	**Pulmonology Group**
Riitta Lahesmaa	Wellbeing Services County of Southwest Finland, Turku, Finland	rilahes@utu.fi	Clinical Groups (FinnGen phases 1&2)	**Pulmonology Group**
Nizar Smaoui	Abbvie, Chicago, IL, United States	nizar.smaoui@abbvie.com	Clinical Groups (FinnGen phases 1&2)	**Pulmonology Group**
Coralie Viollet	AstraZeneca, Cambridge, United Kingdom	coralie.viollet@astrazeneca.com	Clinical Groups (FinnGen phases 1&2)	**Pulmonology Group**
Susan Eaton	Biogen, Cambridge, MA, United States	susan.eaton@biogen.com	Clinical Groups (FinnGen phases 1&2)	**Pulmonology Group**
Hubert Chen	Genentech, San Francisco, CA, United States	chenh37@gene.com	Clinical Groups (FinnGen phases 1&2)	**Pulmonology Group**
Rion Pendergrass	Genentech, San Francisco, CA, United States	penders2@gene.com	Clinical Groups (FinnGen phases 1&2)	**Pulmonology Group**
Natalie Bowers	Genentech, San Francisco, CA, United States	bowersn1@gene.com	Clinical Groups (FinnGen phases 1&2)	**Pulmonology Group**
Joanna Betts	GlaxoSmithKline, Brentford, United Kingdom	joanna.c.betts@gsk.com	Clinical Groups (FinnGen phases 1&2)	**Pulmonology Group**
Kirsi Auro	GlaxoSmithKline, Espoo, Finland	kirsi.m.auro@gsk.com	Clinical Groups (FinnGen phases 1&2)	**Pulmonology Group**
Rajashree Mishra	GlaxoSmithKline, Brentford, United Kingdom	rajashree.x.mishra@gsk.com	Clinical Groups (FinnGen phases 1&2)	**Pulmonology Group**
Majd Mouded	Novartis, Basel, Switzerland	majd.mouded@novartis.com	Clinical Groups (FinnGen phases 1&2)	**Pulmonology Group**
Debby Ngo	Novartis, Basel, Switzerland	debby.ngo@novartis.com	Clinical Groups (FinnGen phases 1&2)	**Pulmonology Group**
Teemu Niiranen	University of Turku, Turku, Finland; Finnish Institute for Health and Welfare (THL), Helsinki, Finland	teemu.niiranen@thl.fi	Clinical Groups (FinnGen phases 1&2)	**Cardiometabolic Diseases Group**

Felix Vaura	Finnish Institute for Health and Welfare (THL), Helsinki, Finland	fechva@utu.fi	Clinical Groups (FinnGen phases 1&2)	**Cardiometabolic Diseases Group**
Veikko Salomaa	Finnish Institute for Health and Welfare (THL), Helsinki, Finland	veikko.salomaa@thl.fi	Clinical Groups (FinnGen phases 1&2)	**Cardiometabolic Diseases Group**
Kaj Metsärinne	Wellbeing Services County of Southwest Finland, Turku, Finland	kaj.metsarinne@tyks.fi	Clinical Groups (FinnGen phases 1&2)	**Cardiometabolic Diseases Group**
Jenni Aittokallio	Wellbeing Services County of Southwest Finland, Turku, Finland	jemato@utu.fi	Clinical Groups (FinnGen phases 1&2)	**Cardiometabolic Diseases Group**
Mika Kähönen	Finnish Clinical Biobank Tampere / University of Tampere / Wellbeing Services County of Pirkanmaa, Tampere, Finland	mika.kahonen@uta.fi	Clinical Groups (FinnGen phases 1&2)	**Cardiometabolic Diseases Group**
Jussi Hernesniemi	Wellbeing Services County of Pirkanmaa, Tampere, Finland	jussi.hernesniemi@tuni.fi	Clinical Groups (FinnGen phases 1&2)	**Cardiometabolic Diseases Group**
Daniel Gordin	Hospital District of Helsinki and Uusimaa, Helsinki, Finland	daniel.gordin@hus.fi	Clinical Groups (FinnGen phases 1&2)	**Cardiometabolic Diseases Group**
Juha Sinisalo	Hospital District of Helsinki and Uusimaa, Helsinki, Finland	juha.sinisalo@hus.fi	Clinical Groups (FinnGen phases 1&2)	**Cardiometabolic Diseases Group**
Marja-Riitta Taskinen	Hospital District of Helsinki and Uusimaa, Helsinki, Finland	marja-riitta.taskinen@helsinki.fi	Clinical Groups (FinnGen phases 1&2)	**Cardiometabolic Diseases Group**
Tiinamaija Tuomi	Institute for Molecular Medicine Finland (FIMM), HiLIFE, University of Helsinki, Helsinki, Finland; Hospital District of Helsinki and Uusimaa, Helsinki, Finland	tiinamaija.tuomi@hus.fi	Clinical Groups (FinnGen phases 1&2)	**Cardiometabolic Diseases Group**
Timo Hiltunen	Hospital District of Helsinki and Uusimaa, Helsinki, Finland	timo.hiltunen@hus.fi	Clinical Groups (FinnGen phases 1&2)	**Cardiometabolic Diseases Group**
Jari Laukkanen	Central Finland Biobank / University of Jyväskylä / Wellbeing Services County of Central Finland, Jyväskylä, Finland	jari.laukkanen@hyvaks.fi	Clinical Groups (FinnGen phases 1&2)	**Cardiometabolic Diseases Group**
Amanda Elliott	Institute for Molecular Medicine Finland (FIMM), HiLIFE, University of Helsinki, Helsinki, Finland; Broad Institute, Cambridge, MA, USA and Massachusetts General Hospital, Boston, MA, USA	aelliott@broadinstitute.org	Clinical Groups (FinnGen phases 1&2)	**Cardiometabolic Diseases Group**
Mary Pat Reeve	Institute for Molecular Medicine Finland (FIMM), HiLIFE, University of Helsinki, Helsinki, Finland	mary.reeve@helsinki.fi	Clinical Groups (FinnGen phases 1&2)	**Cardiometabolic Diseases Group**
Sanni Ruotsalainen	Institute for Molecular Medicine Finland (FIMM), HiLIFE, University of Helsinki, Helsinki, Finland	sanni.ruotsalainen@helsinki.fi	Clinical Groups (FinnGen phases 1&2)	**Cardiometabolic Diseases Group**
Dirk Paul	Astra Zeneca, Cambridge, United Kingdom	dirk.paul@astrazeneca.com	Clinical Groups (FinnGen phases 1&2)	**Cardiometabolic Diseases Group**
Natalie Bowers	Genentech, San Francisco, CA, United States	bowersn1@gene.com	Clinical Groups (FinnGen phases 1&2)	**Cardiometabolic Diseases Group**
Rion Pendergrass	Genentech, San Francisco, CA, United States	penders2@gene.com	Clinical Groups (FinnGen phases 1&2)	**Cardiometabolic Diseases Group**
Audrey Chu	GlaxoSmithKline, Brentford, United Kingdom	audrey.y.chu@gsk.com	Clinical Groups (FinnGen phases 1&2)	**Cardiometabolic Diseases Group**
Dermot Reilly	Johnson & Johnson Innovative Medicine, Boston, MA, United States	dreill11@its.jnj.com	Clinical Groups (FinnGen phases 1&2)	**Cardiometabolic Diseases Group**
Mike Mendelson	Novartis, Boston, MA, United States	mike.mendelson@novartis.com	Clinical Groups (FinnGen phases 1&2)	**Cardiometabolic Diseases Group**
Jaakko Parkkinen	Pfizer, New York, NY, United States	jaakko.parkkinen@pfizer.com	Clinical Groups (FinnGen phases 1&2)	**Cardiometabolic Diseases Group**
Melissa Miller	Pfizer, New York, NY, United States	melissa.r.miller@pfizer.com	Clinical Groups (FinnGen phases 1&2)	**Cardiometabolic Diseases Group**
Tuomo Meretoja	Helsinki University Hospital and University of Helsinki, Helsinki, Finland	tuomo.meretoja@hus.fi	Clinical Groups (FinnGen phases 1&2)	**Oncology Group**
Heikki Joensuu	Helsinki University Hospital and University of Helsinki, Helsinki, Finland	heikki.joensuu@hus.fi	Clinical Groups (FinnGen phases 1&2)	**Oncology Group**
Olli Carpén	Hospital District of Helsinki and Uusimaa, Helsinki, Finland	olli.carpen@helsinki.fi	Clinical Groups (FinnGen phases 1&2)	**Oncology Group**
Johanna Mattson	Hospital District of Helsinki and Uusimaa, Helsinki, Finland	johanna.mattson@hus.fi	Clinical Groups (FinnGen phases 1&2)	**Oncology Group**
Eveliina Salminen	Hospital District of Helsinki and Uusimaa, Helsinki, Finland	eveliina.e.salminen@hus.fi	Clinical Groups (FinnGen phases 1&2)	**Oncology Group**
Annika Auranen	Wellbeing Services County of Pirkanmaa, Tampere, Finland	anaura@utu.fi	Clinical Groups (FinnGen phases 1&2)	**Oncology Group**
Peeter Karihtala	Helsinki University Hospital and University of Helsinki, Helsinki, Finland	peeter.karihtala@hus.fi	Clinical Groups (FinnGen phases 1&2)	**Oncology Group**
Päivi Auvinen	Wellbeing services county of North Savo, Kuopio, Finland	paivi.auvinen@kuh.fi	Clinical Groups (FinnGen phases 1&2)	**Oncology Group**
Klaus Elenius	Wellbeing Services County of Southwest Finland, Turku, Finland	klaus.elenius@utu.fi	Clinical Groups (FinnGen phases 1&2)	**Oncology Group**
Johanna Schleutker	Wellbeing Services County of Southwest Finland, Turku, Finland	johanna.schleutker@utu.fi	Clinical Groups (FinnGen phases 1&2)	**Oncology Group**
Esa Pitkänen	Institute for Molecular Medicine Finland (FIMM), HiLIFE, University of Helsinki, Helsinki, Finland	esa.pitkanen@helsinki.fi	Clinical Groups (FinnGen phases 1&2)	**Oncology Group**
Nina Mars	Institute for Molecular Medicine Finland (FIMM), HiLIFE, University of Helsinki, Helsinki, Finland	nina.mars@helsinki.fi	Clinical Groups (FinnGen phases 1&2)	**Oncology Group**

Mark Daly	Institute for Molecular Medicine Finland (FIMM), HiLIFE, University of Helsinki, Helsinki, Finland; Broad Institute of MIT and Harvard; Massachusetts General Hospital, Boston, MA, United States	mark.daly@helsinki.fi	**Clinical Groups (FinnGen phases 1&2)**	**Oncology Group**
Relja Popovic	Abbvie, Chicago, IL, United States	relja.popovic@abbvie.com	**Clinical Groups (FinnGen phases 1&2)**	**Oncology Group**
Jeffrey Waring	Abbvie, Chicago, IL, United States	jeff.waring@abbvie.com	**Clinical Groups (FinnGen phases 1&2)**	**Oncology Group**
Bridget Riley-Gillis	Abbvie, Chicago, IL, United States	bridget.rileygillis@abbvie.com	**Clinical Groups (FinnGen phases 1&2)**	**Oncology Group**
Anne Lehtonen	Abbvie, Chicago, IL, United States	anne.lehtonen@abbvie.com	**Clinical Groups (FinnGen phases 1&2)**	**Oncology Group**
Margarete Fabre	AstraZeneca, Cambridge, United Kingdom	margarete.fabre@astrazeneca.com	**Clinical Groups (FinnGen phases 1&2)**	**Oncology Group**
Jennifer Schutzman	Genentech, San Francisco, CA, United States	schutzman.jennifer@gene.com	**Clinical Groups (FinnGen phases 1&2)**	**Oncology Group**
Natalie Bowers	Genentech, San Francisco, CA, United States	bowersn1@gene.com	**Clinical Groups (FinnGen phases 1&2)**	**Oncology Group**
Rion Pendergrass	Genentech, San Francisco, CA, United States	penders2@gene.com	**Clinical Groups (FinnGen phases 1&2)**	**Oncology Group**
Diptee Kulkarni	GlaxoSmithKline, Brentford, United Kingdom	diptee.a.kulkarni@gsk.com	**Clinical Groups (FinnGen phases 1&2)**	**Oncology Group**
Alessandro Porello	Johnson & Johnson Innovative Medicine, Spring House, PA, United States	APorrell@ITS.JNJ.com	**Clinical Groups (FinnGen phases 1&2)**	**Oncology Group**
Andrey Loboda	Merck, Kenilworth, NJ, United States	andrey_loboda@merck.com	**Clinical Groups (FinnGen phases 1&2)**	**Oncology Group**
Stefan McDonough	Pfizer, New York, NY, United States	stefan.McDonough@pfizer.com	**Clinical Groups (FinnGen phases 1&2)**	**Oncology Group**
Kai Kaarniranta	Wellbeing services county of North Savo, Kuopio, Finland; University of Lodz, Lodz, Poland	kai.kaarniranta@uef.fi	**Clinical Groups (FinnGen phases 1&2)**	**Opthalmology Group**
Joni A Turunen	Helsinki University Hospital and University of Helsinki, Helsinki, Finland; Folkhälsan Research Center, Helsinki, Finland	joni.turunen@helsinki.fi	**Clinical Groups (FinnGen phases 1&2)**	**Opthalmology Group**
Terhi Ollila	Hospital District of Helsinki and Uusimaa, Helsinki, Finland	terhi.ollila@hus.fi	**Clinical Groups (FinnGen phases 1&2)**	**Opthalmology Group**
Hannu Uusitalo	Wellbeing Services County of Pirkanmaa, Tampere, Finland	hannu.uusitalo@tuni.fi	**Clinical Groups (FinnGen phases 1&2)**	**Opthalmology Group**
Juha Karjalainen	Institute for Molecular Medicine Finland (FIMM), HiLIFE, University of Helsinki, Helsinki, Finland	juha.karjalainen@helsinki.fi	**Clinical Groups (FinnGen phases 1&2)**	**Opthalmology Group**
Esa Pitkänen	Institute for Molecular Medicine Finland (FIMM), HiLIFE, University of Helsinki, Helsinki, Finland	esa.pitkanen@helsinki.fi	**Clinical Groups (FinnGen phases 1&2)**	**Opthalmology Group**
Mengzhen Liu	Abbvie, Chicago, IL, United States	mengzhen.liu@abbvie.com	**Clinical Groups (FinnGen phases 1&2)**	**Opthalmology Group**
Erich Strauss	Genentech, San Francisco, CA, United States	strauss.erich@gene.com	**Clinical Groups (FinnGen phases 1&2)**	**Opthalmology Group**
Natalie Bowers	Genentech, San Francisco, CA, United States	bowersn1@gene.com	**Clinical Groups (FinnGen phases 1&2)**	**Opthalmology Group**
Hao Chen	Genentech, San Francisco, CA, United States	haoc@gene.com	**Clinical Groups (FinnGen phases 1&2)**	**Opthalmology Group**
Rion Pendergrass	Genentech, San Francisco, CA, United States	penders2@gene.com	**Clinical Groups (FinnGen phases 1&2)**	**Opthalmology Group**
Kaisa Tasanen	Wellbeing services county of North Ostrobothnia, Oulu, Finland	kaisa.tasanen-maatta@oulu.fi	**Clinical Groups (FinnGen phases 1&2)**	**Dermatology Group**
Laura Huilaja	Wellbeing services county of North Ostrobothnia, Oulu, Finland	laura.huilaja@oulu.fi	**Clinical Groups (FinnGen phases 1&2)**	**Dermatology Group**
Katariina Hannula-Jouppi	Hospital District of Helsinki and Uusimaa, Helsinki, Finland	katariina.hannula-jouppi@hus.fi	**Clinical Groups (FinnGen phases 1&2)**	**Dermatology Group**
Teea Salmi	Wellbeing Services County of Pirkanmaa, Tampere, Finland	teea.salmi@tuni.fi	**Clinical Groups (FinnGen phases 1&2)**	**Dermatology Group**
Sirkku Peltonen	Wellbeing Services County of Southwest Finland, Turku, Finland	sipelto@utu.fi	**Clinical Groups (FinnGen phases 1&2)**	**Dermatology Group**
Leena Koulu	Wellbeing Services County of Southwest Finland, Turku, Finland	leena.koulu@tyks.fi	**Clinical Groups (FinnGen phases 1&2)**	**Dermatology Group**
Nizar Smaoui	Abbvie, Chicago, IL, United States	nizar.smaoui@abbvie.com	**Clinical Groups (FinnGen phases 1&2)**	**Dermatology Group**
Fedik Rahimov	Abbvie, Chicago, IL, United States	fedik.rahimov@abbvie.com	**Clinical Groups (FinnGen phases 1&2)**	**Dermatology Group**
Anne Lehtonen	Abbvie, Chicago, IL, United States	anne.lehtonen@abbvie.com	**Clinical Groups (FinnGen phases 1&2)**	**Dermatology Group**
David Choy	Genentech, San Francisco, CA, United States	choy.david@gene.com	**Clinical Groups (FinnGen phases 1&2)**	**Dermatology Group**
Rion Pendergrass	Genentech, San Francisco, CA, United States	penders2@gene.com	**Clinical Groups (FinnGen phases 1&2)**	**Dermatology Group**
Dawn Waterworth	Johnson & Johnson Innovative Medicine, Spring House, PA, United States	dwaterwo@its.jnj.com	**Clinical Groups (FinnGen phases 1&2)**	**Dermatology Group**
Kirsi Kalpala	Pfizer, New York, NY, United States	kirsi.kalpala@pfizer.com	**Clinical Groups (FinnGen phases 1&2)**	**Dermatology Group**

Ying Wu	Pfizer, New York, NY, United States	ying.wu3@pfizer.com	Clinical Groups (FinnGen phases 1&2)	**Dermatology Group**
Pirkko Pussinen	Hospital District of Helsinki and Uusimaa, Helsinki, Finland	pirkko.pussinen@helsinki.fi	Clinical Groups (FinnGen phases 1&2)	**Odontology Group**
Aino Salminen	Hospital District of Helsinki and Uusimaa, Helsinki, Finland	aino.m.salminen@helsinki.fi	Clinical Groups (FinnGen phases 1&2)	**Odontology Group**
Tuula Salo	Hospital District of Helsinki and Uusimaa, Helsinki, Finland	tuula.salo@helsinki.fi	Clinical Groups (FinnGen phases 1&2)	**Odontology Group**
David Rice	Hospital District of Helsinki and Uusimaa, Helsinki, Finland	david.rice@helsinki.fi	Clinical Groups (FinnGen phases 1&2)	**Odontology Group**
Pekka Nieminen	Hospital District of Helsinki and Uusimaa, Helsinki, Finland	pekka.nieminen@helsinki.fi	Clinical Groups (FinnGen phases 1&2)	**Odontology Group**
Ulla Palotie	Hospital District of Helsinki and Uusimaa, Helsinki, Finland	ulla.palotie@helsinki.fi	Clinical Groups (FinnGen phases 1&2)	**Odontology Group**
Maria Siponen	Wellbeing services county of North Savo, Kuopio, Finland	maria.siponen@uef.fi	Clinical Groups (FinnGen phases 1&2)	**Odontology Group**
Liisa Suominen	Wellbeing services county of North Savo, Kuopio, Finland	liisa.suominen@uef.fi	Clinical Groups (FinnGen phases 1&2)	**Odontology Group**
Päivi Mäntylä	Wellbeing services county of North Savo, Kuopio, Finland	paivi.mantyla@uef.fi	Clinical Groups (FinnGen phases 1&2)	**Odontology Group**
Ulvi Gursoy	Wellbeing Services County of Southwest Finland, Turku, Finland	ulvi.gursoy@utu.fi	Clinical Groups (FinnGen phases 1&2)	**Odontology Group**
Vuokko Anttonen	Wellbeing services county of North Ostrobothnia, Oulu, Finland	vuokko.anttonen@oulu.fi	Clinical Groups (FinnGen phases 1&2)	**Odontology Group**
Kirsi Sipilä	Oulu University Hospital and University of Oulu, Oulu, Finland	kirsi.sipila@oulu.fi	Clinical Groups (FinnGen phases 1&2)	**Odontology Group**
Rion Pendergrass	Genentech, San Francisco, CA, United States	pendergrass.sarah@gene.com	Clinical Groups (FinnGen phases 1&2)	**Odontology Group**
Hannele Laivuori	Institute for Molecular Medicine Finland (FIMM), HiLIFE, University of Helsinki, Helsinki, Finland	hannele.laivuori@helsinki.fi	Clinical Groups (FinnGen phases 1&2)	**Women's Health and Reproduction Group**
Venla Kurra	Wellbeing Services County of Pirkanmaa, Tampere, Finland	venla.kurra@tuni.fi	Clinical Groups (FinnGen phases 1&2)	**Women's Health and Reproduction Group**
Laura Kotaniemi-Talonen	Wellbeing Services County of Pirkanmaa, Tampere, Finland	laura.kotaniemi-talonen@tuni.fi	Clinical Groups (FinnGen phases 1&2)	**Women's Health and Reproduction Group**
Oskari Heikinheimo	Hospital District of Helsinki and Uusimaa, Helsinki, Finland	oskari.heikinheimo@helsinki.fi	Clinical Groups (FinnGen phases 1&2)	**Women's Health and Reproduction Group**
Ilkka Kalliala	Hospital District of Helsinki and Uusimaa, Helsinki, Finland	ilkka.kalliala@hus.fi	Clinical Groups (FinnGen phases 1&2)	**Women's Health and Reproduction Group**
Lauri Aaltonen	Hospital District of Helsinki and Uusimaa, Helsinki, Finland	lauri.aaltonen@helsinki.fi	Clinical Groups (FinnGen phases 1&2)	**Women's Health and Reproduction Group**
Varpu Jokimaa	Wellbeing Services County of Southwest Finland, Turku, Finland	varpu.jokimaa@utu.fi	Clinical Groups (FinnGen phases 1&2)	**Women's Health and Reproduction Group**
Johannes Kettunen	Northern Finland Biobank Borealis / University of Oulu / Wellbeing services county of North Ostrobothnia, Oulu, Finland	Johannes.Kettunen@oulu.fi	Clinical Groups (FinnGen phases 1&2)	**Women's Health and Reproduction Group**
Marja Vääräsmäki	Wellbeing services county of North Ostrobothnia, Oulu, Finland	marja.vaarasmaki@oulu.fi	Clinical Groups (FinnGen phases 1&2)	**Women's Health and Reproduction Group**
Outi Uimari	Wellbeing services county of North Ostrobothnia, Oulu, Finland	outi.uimari@oulu.fi	Clinical Groups (FinnGen phases 1&2)	**Women's Health and Reproduction Group**
Laure Morin-Papunen	Wellbeing services county of North Ostrobothnia, Oulu, Finland	lmp@cc.oulu.fi	Clinical Groups (FinnGen phases 1&2)	**Women's Health and Reproduction Group**
Maarit Niinimäki	Wellbeing services county of North Ostrobothnia, Oulu, Finland	maarit.niinimaki@oulu.fi	Clinical Groups (FinnGen phases 1&2)	**Women's Health and Reproduction Group**
Terhi Piltonen	Wellbeing services county of North Ostrobothnia, Oulu, Finland	terhi.piltonen@oulu.fi	Clinical Groups (FinnGen phases 1&2)	**Women's Health and Reproduction Group**
Katja Kivinen	Institute for Molecular Medicine Finland (FIMM), HiLIFE, University of Helsinki, Helsinki, Finland	katja.kivinen@helsinki.fi	Clinical Groups (FinnGen phases 1&2)	**Women's Health and Reproduction Group**
Elisabeth Widen	Institute for Molecular Medicine Finland (FIMM), HiLIFE, University of Helsinki, Helsinki, Finland	elisabeth.widen@helsinki.fi	Clinical Groups (FinnGen phases 1&2)	**Women's Health and Reproduction Group**
Taru Tukiainen	Institute for Molecular Medicine Finland (FIMM), HiLIFE, University of Helsinki, Helsinki, Finland	taru.tukiainen@helsinki.fi	Clinical Groups (FinnGen phases 1&2)	**Women's Health and Reproduction Group**
Mary Pat Reeve	Institute for Molecular Medicine Finland (FIMM), HiLIFE, University of Helsinki, Helsinki, Finland	mary.reeve@helsinki.fi	Clinical Groups (FinnGen phases 1&2)	**Women's Health and Reproduction Group**
Mark Daly	Institute for Molecular Medicine Finland (FIMM), HiLIFE, University of Helsinki, Helsinki, Finland; Broad Institute of MIT and Harvard; Massachusetts General Hospital, Boston, MA, United States	mark.daly@helsinki.fi	Clinical Groups (FinnGen phases 1&2)	**Women's Health and Reproduction Group**
Niko Välimäki	University of Helsinki, Helsinki, Finland	niko.valimaki@helsinki.fi	Clinical Groups (FinnGen phases 1&2)	**Women's Health and Reproduction Group**
Eija Laakkonen	University of Jyväskylä, Jyväskylä, Finland	eija.k.laakkonen@jyu.fi	Clinical Groups (FinnGen phases 1&2)	**Women's Health and Reproduction Group**
Jaakko Tyrmi	University of Oulu, Oulu, Finland / University of Tampere, Tampere, Finland	jaakko.tyrmi@oulu.fi	Clinical Groups (FinnGen phases 1&2)	**Women's Health and Reproduction Group**
Heidi Silven	University of Oulu, Oulu, Finland	heidi.silven@student.oulu.fi	Clinical Groups (FinnGen phases 1&2)	**Women's Health and Reproduction Group**
Eeva Sliz	University of Oulu, Oulu, Finland	eeva.sliz@oulu.fi	Clinical Groups (FinnGen phases 1&2)	**Women's Health and Reproduction Group**

Riikka Arffman	University of Oulu, Oulu, Finland	riikka.arffman@oulu.fi	Clinical Groups (FinnGen phases 1&2)	**Women's Health and Reproduction Group**
Susanna Savukoski	University of Oulu, Oulu, Finland	susanna.savukoski@oulu.fi	Clinical Groups (FinnGen phases 1&2)	**Women's Health and Reproduction Group**
Triin Laisk	Estonian biobank, Tartu, Estonia	triin.laisk@ut.ee	Clinical Groups (FinnGen phases 1&2)	**Women's Health and Reproduction Group**
Natalia Pujol	Estonian biobank, Tartu, Estonia	natalia.pujolgualdo@oulu.fi	Clinical Groups (FinnGen phases 1&2)	**Women's Health and Reproduction Group**
Mengzhen Liu	AbbVie, Chicago, IL, United States	mengzhen.liu@abbvie.com	Clinical Groups (FinnGen phases 1&2)	**Women's Health and Reproduction Group**
Bridget Riley-Gillis	AbbVie, Chicago, IL, United States	bridget.rileygillis@abbvie.com	Clinical Groups (FinnGen phases 1&2)	**Women's Health and Reproduction Group**
Rion Pendergrass	Genentech, San Francisco, CA, United States	penders2@gene.com	Clinical Groups (FinnGen phases 1&2)	**Women's Health and Reproduction Group**
Janet Kumar	GlaxoSmithKline, Collegeville, PA, United States	janet.x.kumar@gsk.com	Clinical Groups (FinnGen phases 1&2)	**Women's Health and Reproduction Group**
Iiris Hovatta	University of Helsinki, Finland	iiris.hovatta@helsinki.fi	Clinical Groups (FinnGen phases 1&2)	**Depression group**
Erkki Isometsä	Hospital District of Helsinki and Uusimaa, Helsinki, Finland	erkki.isometsa@hus.fi	Clinical Groups (FinnGen phases 1&2)	**Depression group**
Hanna Ollila	Institute for Molecular Medicine Finland (FIMM), HiLIFE, University of Helsinki, Helsinki, Finland	hanna.m.ollila@helsinki.fi	Clinical Groups (FinnGen phases 1&2)	**Depression group**
Jaana Suvisaari	Finnish Institute for Health and Welfare (THL), Helsinki, Finland	jaana.suvisaari@thl.fi	Clinical Groups (FinnGen phases 1&2)	**Depression group**
Antti Mäkitie	University of Helsinki and Helsinki University Hospital, Helsinki, Finland	antti.makitie@helsinki.fi	Clinical Groups (FinnGen phases 1&2)	**ENT (ear, nose and throath) Group**
Argyro Bizaki-Vallaskangas	Wellbeing Services County of Pirkanmaa, Tampere, Finland	argyro.bizaki-vallaskangas@tuni.fi	Clinical Groups (FinnGen phases 1&2)	**ENT (ear, nose and throath) Group**
Sanna Toppila-Salmi	University of Eastern Finland and Kuopio University Hospital, Kuopio, Finland; Helsinki University Hospital and University of Helsinki, Finland	sanna.salmi@helsinki.fi	Clinical Groups (FinnGen phases 1&2)	**ENT (ear, nose and throath) Group**
Tytti Willberg	Wellbeing Services County of Southwest Finland, Turku, Finland	tytti.willberg@tyks.fi	Clinical Groups (FinnGen phases 1&2)	**ENT (ear, nose and throath) Group**
Elmo Saarentaus	Institute for Molecular Medicine Finland (FIMM), HiLIFE, University of Helsinki, Helsinki, Finland	elmo.saarentaus@helsinki.fi	Clinical Groups (FinnGen phases 1&2)	**ENT (ear, nose and throath) Group**
Antti Aarnisalo	Hospital District of Helsinki and Uusimaa, Helsinki, Finland	antti.aarnisalo@hus.fi	Clinical Groups (FinnGen phases 1&2)	**ENT (ear, nose and throath) Group**
Eveliina Salminen	Hospital District of Helsinki and Uusimaa, Helsinki, Finland	eveliina.e.salminen@hus.fi	Clinical Groups (FinnGen phases 1&2)	**ENT (ear, nose and throath) Group**
Elisa Rahikkala	Northern Ostrobothnia Hospital District, Oulu, Finland	elisa.rahikkala@oulu.fi	Clinical Groups (FinnGen phases 1&2)	**ENT (ear, nose and throath) Group**
Johannes Kettunen	Northern Finland Biobank Borealis / University of Oulu / Wellbeing services county of North Ostrobothnia, Oulu, Finland	johannes.kettunen@oulu.fi	Clinical Groups (FinnGen phases 1&2)	**ENT (ear, nose and throath) Group**
Kristiina Aittomäki	Helsinki University Central Hospital, Helsinki, Finland	kristiina.aittomaki@helsinki.fi	Clinical Groups (FinnGen phases 1&2)	**POI (premature ovarian failure) Group**
Fredrik Åberg	Helsinki University Hospital and University of Helsinki, Helsinki, Finland	fredrik.aberg@helsinki.fi	Clinical Groups (FinnGen phases 1&2)	**LiverScore Group**
Joel Rämö	Institute for Molecular Medicine Finland (FIMM), HiLIFE, University of Helsinki, Helsinki, Finland; Broad Institute, Cambridge, MA, United States	joel.ramo@helsinki.fi	Clinical Task Forces (FinnGen phase 3)	**Eye diseases Task Force**
Mark Daly	Institute for Molecular Medicine Finland (FIMM), HiLIFE, University of Helsinki, Helsinki, Finland; Broad Institute of MIT and Harvard; Massachusetts General Hospital, Boston, MA, United States	mjdaly@broadinstitute.org	Clinical Task Forces (FinnGen phase 3)	**Eye diseases Task Force**
Mary Pat Reeve	Institute for Molecular Medicine Finland (FIMM), HiLIFE, University of Helsinki, Helsinki, Finland; Broad Institute, Cambridge, MA, United States	mary.reeve@helsinki.fi	Clinical Task Forces (FinnGen phase 3)	**Eye diseases Task Force**
Muhammad Adnan Khan	Institute for Molecular Medicine Finland (FIMM), HiLIFE, University of Helsinki, Helsinki, Finland	adnan.khan@helsinki.fi	Clinical Task Forces (FinnGen phase 3)	**Eye diseases Task Force**
Johanna Mäkelä	Finnish Biobank Cooperative - FINBB	johanna.makela@finbb.fi	Clinical Task Forces (FinnGen phase 3)	**Eye diseases Task Force**
Ilkka Immonen	Hospital District of Helsinki and Uusimaa, Helsinki, Finland	ilkka.immonen@hus.fi	Clinical Task Forces (FinnGen phase 3)	**Eye diseases Task Force**
Kai Kaarniranta	Wellbeing services county of North Savo, Kuopio, Finland; University of Lodz, Lodz, Poland	kai.kaarniranta@uef.fi	Clinical Task Forces (FinnGen phase 3)	**Eye diseases Task Force**
Joni A Turunen	Helsinki University Hospital and University of Helsinki, Helsinki, Finland; Folkhälsan Research Center, Helsinki, Finland	Joni.Turunen@hus.fi	Clinical Task Forces (FinnGen phase 3)	**Eye diseases Task Force**
Anneke Den Hollander	AbbVie, Chicago, IL, United States	anneke.denhollander@abbvie.com	Clinical Task Forces (FinnGen phase 3)	**Eye diseases Task Force**
Bridget Riley-Gillis	AbbVie, Chicago, IL, United States	bridget.rileygillis@abbvie.com	Clinical Task Forces (FinnGen phase 3)	**Eye diseases Task Force**
Mengzhen Liu	AbbVie, Chicago, IL, United States	mengzhen.liu@abbvie.com	Clinical Task Forces (FinnGen phase 3)	**Eye diseases Task Force**
Nizar Smaoui	AbbVie, Chicago, IL, United States	nizar.smaoui@abbvie.com	Clinical Task Forces (FinnGen phase 3)	**Eye diseases Task Force**

Fabio Baschiera	Bayer AG, Leverkusen, Germany	fabio.baschiera@bayer.com	Clinical Task Forces (FinnGen phase 3)	**Eye diseases Task Force**
Hans van Leeuwen	Bayer AG, Leverkusen, Germany	hans.vanleeuwen@bayer.com	Clinical Task Forces (FinnGen phase 3)	**Eye diseases Task Force**
Elke Markert	Boehringer Ingelheim, Ingelheim am Rhein, Germany	elke.markert@boehringer-ingelheim.com	Clinical Task Forces (FinnGen phase 3)	**Eye diseases Task Force**
Brian Yaspan	Genentech, San Francisco, CA, United States	yaspan.brian@gene.com	Clinical Task Forces (FinnGen phase 3)	**Eye diseases Task Force**
Charli Harlow	GlaxoSmithKline, Collegeville, PA, United States	charli.e.harlow@gsk.com	Clinical Task Forces (FinnGen phase 3)	**Eye diseases Task Force**
Lea Sarow-Blat	GlaxoSmithKline, Collegeville, PA, United States	lea.2.sarov-blat@gsk.com	Clinical Task Forces (FinnGen phase 3)	**Eye diseases Task Force**
Dermont Reilly	Johnson & Johnson Innovative Medicine, Spring House, PA, United States	DReill11@its.jnj.com	Clinical Task Forces (FinnGen phase 3)	**Eye diseases Task Force**
P. Dunnmon	Johnson & Johnson Innovative Medicine, Spring House, PA, United States	PDunnmon@ITS.JNJ.com	Clinical Task Forces (FinnGen phase 3)	**Eye diseases Task Force**
Sara Gale	Johnson & Johnson Innovative Medicine, Spring House, PA, United States	sgale1@its.jnj.com	Clinical Task Forces (FinnGen phase 3)	**Eye diseases Task Force**
Fabiana Farias	Merck, Kenilworth, NJ, United States	fabiana.farias@merck.com	Clinical Task Forces (FinnGen phase 3)	**Eye diseases Task Force**
Jorge Del-aguila	Merck, Kenilworth, NJ, United States	jorge.del-aguila@merck.com	Clinical Task Forces (FinnGen phase 3)	**Eye diseases Task Force**
Catherine O'Riordan	Translational Sciences, Sanofi R&D, Framingham, MA, USA	Catherine.O'Riordan@sanofi.com	Clinical Task Forces (FinnGen phase 3)	**Eye diseases Task Force**
Samuel Lessard	Translational Sciences, Sanofi R&D, Framingham, MA, USA	samuel.lessard@sanofi.com	Clinical Task Forces (FinnGen phase 3)	**Eye diseases Task Force**
Suzanne Jacobs	Translational Sciences, Sanofi R&D, Framingham, MA, USA	Suzanne.Jacobs@sanofi.com	Clinical Task Forces (FinnGen phase 3)	**Eye diseases Task Force**
Satu Koskela	Finnish Red Cross Blood Service / Finnish Hematology Registry and Clinical Biobank, Helsinki, Finland	satu.koskela@veripalvelu.fi	Clinical Task Forces (FinnGen phase 3)	**Immune mediated diseases Task Force**
Anne Kerola	Institute for Molecular Medicine Finland (FIMM), HiLIFE, University of Helsinki, Helsinki, Finland	anne.kerola@helsinki.fi	Clinical Task Forces (FinnGen phase 3)	**Immune mediated diseases Task Force**
Elisa Lahtela	Institute for Molecular Medicine Finland (FIMM), HiLIFE, University of Helsinki, Helsinki, Finland	laura.lahtela@helsinki.fi	Clinical Task Forces (FinnGen phase 3)	**Immune mediated diseases Task Force**
Helen Cooper	Institute for Molecular Medicine Finland (FIMM), HiLIFE, University of Helsinki, Helsinki, Finland	helen.cooper@helsinki.fi	Clinical Task Forces (FinnGen phase 3)	**Immune mediated diseases Task Force**
Johanna Paltta	Institute for Molecular Medicine Finland (FIMM), HiLIFE, University of Helsinki, Helsinki, Finland; University of Turku, Turku, Finland	johanna.paltta@varha.fi	Clinical Task Forces (FinnGen phase 3)	**Immune mediated diseases Task Force**
Jukka Koskela	Institute for Molecular Medicine Finland (FIMM), HiLIFE, University of Helsinki, Helsinki, Finland	jukka.koskela@helsinki.fi	Clinical Task Forces (FinnGen phase 3)	**Immune mediated diseases Task Force**
Mark Daly	Institute for Molecular Medicine Finland (FIMM), HiLIFE, University of Helsinki, Helsinki, Finland; Broad Institute of MIT and Harvard; Massachusetts General Hospital, Boston, MA, United States	mjdaly@broadinstitute.com	Clinical Task Forces (FinnGen phase 3)	**Immune mediated diseases Task Force**
Mary Pat Reeve	Institute for Molecular Medicine Finland (FIMM), HiLIFE, University of Helsinki, Helsinki, Finland; Broad Institute, Cambridge, MA, United States	mary.reeve@helsinki.fi	Clinical Task Forces (FinnGen phase 3)	**Immune mediated diseases Task Force**
Vincent Llorens	Institute for Molecular Medicine Finland (FIMM), HiLIFE, University of Helsinki, Helsinki, Finland	vincent.llorens@helsinki.fi	Clinical Task Forces (FinnGen phase 3)	**Immune mediated diseases Task Force**
Martti Färkkilä	Hospital District of Helsinki and Uusimaa, Helsinki, Finland	martti.farkkila@hus.fi	Clinical Task Forces (FinnGen phase 3)	**Immune mediated diseases Task Force**
Johannes Kettunen	Northern Finland Biobank Borealis / University of Oulu / Wellbeing services county of North Ostrobothnia, Oulu, Finland	johannes.Kettunen@oulu.fi	Clinical Task Forces (FinnGen phase 3)	**Immune mediated diseases Task Force**
Kaisa Tasanen-Maatta	Wellbeing services county of North Ostrobothnia, Oulu, Finland	kaisa.tasanen-maatta@oulu.fi	Clinical Task Forces (FinnGen phase 3)	**Immune mediated diseases Task Force**
Laura Huilaja	Wellbeing services county of North Ostrobothnia, Oulu, Finland	laura.huilaja@oulu.fi	Clinical Task Forces (FinnGen phase 3)	**Immune mediated diseases Task Force**
Minna Ruddock	Arctic biobank / University of Oulu, Oulu, Finland	minna.ruddock@oulu.fi	Clinical Task Forces (FinnGen phase 3)	**Immune mediated diseases Task Force**
Aki Havulinna	Finnish Institute for Health and Welfare (THL), Helsinki, Finland	aki.havulinna@thl.fi	Clinical Task Forces (FinnGen phase 3)	**Immune mediated diseases Task Force**
Antti Palomäki	University of Turku, Turku, Finland	ajpalo@utu.fi	Clinical Task Forces (FinnGen phase 3)	**Immune mediated diseases Task Force**
Laura Kuusalo	University of Turku, Turku, Finland	laanku@utu.fi	Clinical Task Forces (FinnGen phase 3)	**Immune mediated diseases Task Force**
Laura Pirilä	University of Turku, Turku, Finland	laura.pirila@fimnet.fi	Clinical Task Forces (FinnGen phase 3)	**Immune mediated diseases Task Force**
Fedik Rahimov	AbbVie, Chicago, IL, United States	fedik.rahimov@abbvie.com	Clinical Task Forces (FinnGen phase 3)	**Immune mediated diseases Task Force**
Jan Freudenberg	AbbVie, Chicago, IL, United States	jan.freudenberg@abbvie.com	Clinical Task Forces (FinnGen phase 3)	**Immune mediated diseases Task Force**
Nizar Smaoui	AbbVie, Chicago, IL, United States	nizar.smaoui@abbvie.com	Clinical Task Forces (FinnGen phase 3)	**Immune mediated diseases Task Force**

Bram Prins	Astra Zeneca, Cambridge, United Kingdom	bram.prins@astrazeneca.com	Clinical Task Forces (FinnGen phase 3)	**Immune mediated diseases Task Force**
Coralie Viollet	Astra Zeneca, Cambridge, United Kingdom	coralie.viollet@astrazeneca.com	Clinical Task Forces (FinnGen phase 3)	**Immune mediated diseases Task Force**
Eleanor Wheeler	Astra Zeneca, Cambridge, United Kingdom	eleanor.wheeler@astrazeneca.com	Clinical Task Forces (FinnGen phase 3)	**Immune mediated diseases Task Force**
Kousik Kundu	Astra Zeneca, Cambridge, United Kingdom	kousik.kundu@astrazeneca.com	Clinical Task Forces (FinnGen phase 3)	**Immune mediated diseases Task Force**
Santosh Atanur	Astra Zeneca, Cambridge, United Kingdom	santosh.atanur@astrazeneca.com	Clinical Task Forces (FinnGen phase 3)	**Immune mediated diseases Task Force**
Hans van Leeuwen	Bayer AG, Leverkusen, Germany	hans.vanleeuwen@bayer.com	Clinical Task Forces (FinnGen phase 3)	**Immune mediated diseases Task Force**
Himanshu Manchanda	Bayer AG, Leverkusen, Germany	himanshu.manchanda@bayer.com	Clinical Task Forces (FinnGen phase 3)	**Immune mediated diseases Task Force**
Karl Heilbron	Bayer AG, Leverkusen, Germany	karl.heilbron@bayer.com	Clinical Task Forces (FinnGen phase 3)	**Immune mediated diseases Task Force**
Martin Rao	Bayer AG, Leverkusen, Germany	martin.rao@bayer.com	Clinical Task Forces (FinnGen phase 3)	**Immune mediated diseases Task Force**
Nicole Schmidt	Bayer AG, Leverkusen, Germany	nicole.schmidt1@bayer.com	Clinical Task Forces (FinnGen phase 3)	**Immune mediated diseases Task Force**
Samu Kurki	Bayer AG, Leverkusen, Germany	samu.kurki@bayer.com	Clinical Task Forces (FinnGen phase 3)	**Immune mediated diseases Task Force**
Ellen Tsai	Biogen, Cambridge, MA, United States	ellen.tsai@biogen.com	Clinical Task Forces (FinnGen phase 3)	**Immune mediated diseases Task Force**
Ketian Yu	Biogen, Cambridge, MA, United States	ketian.yu@biogen.com	Clinical Task Forces (FinnGen phase 3)	**Immune mediated diseases Task Force**
Stephanie Loomis	Biogen, Cambridge, MA, United States	stephanie.loomis@biogen.com	Clinical Task Forces (FinnGen phase 3)	**Immune mediated diseases Task Force**
Benjamin Sun	Bristol Myers Squibb, New York, NY, United States	benjamin.sun@bms.com	Clinical Task Forces (FinnGen phase 3)	**Immune mediated diseases Task Force**
Cara Carty	Bristol Myers Squibb, New York, NY, United States	cara.carty@bms.com	Clinical Task Forces (FinnGen phase 3)	**Immune mediated diseases Task Force**
Emily Holzinger	Bristol Myers Squibb, New York, NY, United States	Emily.Holzinger@bms.com	Clinical Task Forces (FinnGen phase 3)	**Immune mediated diseases Task Force**
Michael Turchin	Bristol Myers Squibb, New York, NY, United States	Michael.turchin@bms.com	Clinical Task Forces (FinnGen phase 3)	**Immune mediated diseases Task Force**
Neelakshi Jog	Bristol Myers Squibb, New York, NY, United States	neelakshi.jog@bms.com	Clinical Task Forces (FinnGen phase 3)	**Immune mediated diseases Task Force**
Frank Li	Boehringer Ingelheim, Ingelheim am Rhein, Germany	frank.li@boehringer-ingelheim.com	Clinical Task Forces (FinnGen phase 3)	**Immune mediated diseases Task Force**
Zhihao Ding	Boehringer Ingelheim, Ingelheim am Rhein, Germany	zhihao.ding@boehringer-ingelheim.com	Clinical Task Forces (FinnGen phase 3)	**Immune mediated diseases Task Force**
Cameron Adams	Genentech, San Francisco, CA, United States	adams.cameron@gene.com	Clinical Task Forces (FinnGen phase 3)	**Immune mediated diseases Task Force**
Mark McCarthy	Genentech, San Francisco, CA, United States	mccarm10@gene.com	Clinical Task Forces (FinnGen phase 3)	**Immune mediated diseases Task Force**
Michael Rothenberg	Genentech, San Francisco, CA, United States	rothenm1@gene.com	Clinical Task Forces (FinnGen phase 3)	**Immune mediated diseases Task Force**
Rion Pendergrass	Genentech, San Francisco, CA, United States	pendergrass.rion@gene.com	Clinical Task Forces (FinnGen phase 3)	**Immune mediated diseases Task Force**
Diana L.Cousminer	GlaxoSmithKline, Collegeville, PA, United States	diana.l.cousminer@gsk.com	Clinical Task Forces (FinnGen phase 3)	**Immune mediated diseases Task Force**
Jagtar Nijjar	GlaxoSmithKline, Collegeville, PA, United States	jagtar.s.nijjar@gsk.com	Clinical Task Forces (FinnGen phase 3)	**Immune mediated diseases Task Force**
Jessica Chao	GlaxoSmithKline, Collegeville, PA, United States	jessica.x.chao@gsk.com	Clinical Task Forces (FinnGen phase 3)	**Immune mediated diseases Task Force**
Joanna C.Betts	GlaxoSmithKline, Collegeville, PA, United States	joanna.c.betts@gsk.com	Clinical Task Forces (FinnGen phase 3)	**Immune mediated diseases Task Force**
Jonathan M.Davitte	GlaxoSmithKline, Collegeville, PA, United States	jonathan.m.davitte@gsk.com	Clinical Task Forces (FinnGen phase 3)	**Immune mediated diseases Task Force**
Linda McGarthy	GlaxoSmithKline, Collegeville, PA, United States	linda.c.mccarthy@gsk.com	Clinical Task Forces (FinnGen phase 3)	**Immune mediated diseases Task Force**
Michal Magid	GlaxoSmithKline, Collegeville, PA, United States	michal.m.magid-slav@gsk.com	Clinical Task Forces (FinnGen phase 3)	**Immune mediated diseases Task Force**
Shashank Jariwala	GlaxoSmithKline, Collegeville, PA, United States	shashank.x.jariwala@gsk.com	Clinical Task Forces (FinnGen phase 3)	**Immune mediated diseases Task Force**
Dawn Waterworth	Johnson & Johnson Innovative Medicine, Spring House, PA, United States	dwaterwo@its.jnj.com	Clinical Task Forces (FinnGen phase 3)	**Immune mediated diseases Task Force**
Amy Hart	Johnson & Johnson Innovative Medicine, Spring House, PA, United States	ahart13@its.jnj.com	Clinical Task Forces (FinnGen phase 3)	**Immune mediated diseases Task Force**
Brice Keyes	Johnson & Johnson Innovative Medicine, Spring House, PA, United States	bkeyes1@its.jnj.com	Clinical Task Forces (FinnGen phase 3)	**Immune mediated diseases Task Force**
John Kwon	Johnson & Johnson Innovative Medicine, Spring House, PA, United States	JKwon10@ITS.JNJ.com	Clinical Task Forces (FinnGen phase 3)	**Immune mediated diseases Task Force**
Jonathan Sherlock	Johnson & Johnson Innovative Medicine, Spring House, PA, United States	JSherlo1@its.jnj.com	Clinical Task Forces (FinnGen phase 3)	**Immune mediated diseases Task Force**

Matt Loza	Johnson & Johnson Innovative Medicine, Spring House, PA, United States	MLoza@its.jnj.com	Clinical Task Forces (FinnGen phase 3)	**Immune mediated diseases Task Force**
Elisabeth Vollmann	Merck, Kenilworth, NJ, United States	elisabeth.vollmann@merck.com	Clinical Task Forces (FinnGen phase 3)	**Immune mediated diseases Task Force**
Jozsef Karman	Merck, Kenilworth, NJ, United States	jozsef.karman@merck.com	Clinical Task Forces (FinnGen phase 3)	**Immune mediated diseases Task Force**
Julie Fiore	Merck, Kenilworth, NJ, United States	julie.fiore1@merck.com	Clinical Task Forces (FinnGen phase 3)	**Immune mediated diseases Task Force**
Rajesh Kamath	Merck, Kenilworth, NJ, United States	rajesh.kamath@merck.com	Clinical Task Forces (FinnGen phase 3)	**Immune mediated diseases Task Force**
Enrico Ferrero	Novartis Institutes for BioMedical Research, Cambridge, MA, United States	enrico.ferrero@novartis.com	Clinical Task Forces (FinnGen phase 3)	**Immune mediated diseases Task Force**
Jonas Zierer	Novartis Institutes for BioMedical Research, Cambridge, MA, United States	jonas.zierer@novartis.com	Clinical Task Forces (FinnGen phase 3)	**Immune mediated diseases Task Force**
Nikos Patsopoulos	Novartis Institutes for BioMedical Research, Cambridge, MA, United States	nikos.patsopoulos@novartis.com	Clinical Task Forces (FinnGen phase 3)	**Immune mediated diseases Task Force**
Erin Macdonald-Dunlop	Pfizer, New York, NY, United States	erin.macdonald-dunlop@pfizer.com	Clinical Task Forces (FinnGen phase 3)	**Immune mediated diseases Task Force**
Jessica Chung	Pfizer, New York, NY, United States	jessica.chung@pfizer.com	Clinical Task Forces (FinnGen phase 3)	**Immune mediated diseases Task Force**
Michael McLean	Pfizer, New York, NY, United States	Michael.McLean@pfizer.com	Clinical Task Forces (FinnGen phase 3)	**Immune mediated diseases Task Force**
Hamid Mattoo	Translational Sciences, Sanofi R&D, Framingham, MA, USA	Hamid.Mattoo@sanofi.com	Clinical Task Forces (FinnGen phase 3)	**Immune mediated diseases Task Force**
Aarno Palotie	Institute for Molecular Medicine Finland (FIMM), HiLIFE, University of Helsinki, Helsinki, Finland; Broad Institute of MIT and Harvard; Massachusetts General Hospital, Boston, MA, United States	aarno.palotie@helsinki.fi	Clinical Task Forces (FinnGen phase 3)	**Kidney diseases Task Force**
Elisa Lahtela	Institute for Molecular Medicine Finland (FIMM), HiLIFE, University of Helsinki, Helsinki, Finland	laura.lahtela@helsinki.fi	Clinical Task Forces (FinnGen phase 3)	**Kidney diseases Task Force**
Helen Cooper	Institute for Molecular Medicine Finland (FIMM), HiLIFE, University of Helsinki, Helsinki, Finland	helen.cooper@helsinki.fi	Clinical Task Forces (FinnGen phase 3)	**Kidney diseases Task Force**
Jukka Koskela	Institute for Molecular Medicine Finland (FIMM), HiLIFE, University of Helsinki, Helsinki, Finland	jukka.koskela@helsinki.fi	Clinical Task Forces (FinnGen phase 3)	**Kidney diseases Task Force**
Mark Daly	Institute for Molecular Medicine Finland (FIMM), HiLIFE, University of Helsinki, Helsinki, Finland; Broad Institute of MIT and Harvard; Massachusetts General Hospital, Boston, MA, United States	mjdaly@broadinstitute.com	Clinical Task Forces (FinnGen phase 3)	**Kidney diseases Task Force**
Mary Pat Reeve	Institute for Molecular Medicine Finland (FIMM), HiLIFE, University of Helsinki, Helsinki, Finland; Broad Institute, Cambridge, MA, United States	mary.reeve@helsinki.fi	Clinical Task Forces (FinnGen phase 3)	**Kidney diseases Task Force**
Raymond Walters	Institute for Molecular Medicine Finland (FIMM), HiLIFE, University of Helsinki, Helsinki, Finland; Broad Institute, Cambridge, MA, United States	rwalters@broadinstitute.org	Clinical Task Forces (FinnGen phase 3)	**Kidney diseases Task Force**
Rodos Rodosthenous	Institute for Molecular Medicine Finland (FIMM), HiLIFE, University of Helsinki, Helsinki, Finland	rodos.rodosthenous@helsinki.fi	Clinical Task Forces (FinnGen phase 3)	**Kidney diseases Task Force**
Jouni Lauronen	Finnish Red Cross Blood Service / Finnish Hematology Registry and Clinical Biobank, Helsinki, Finland	jouni.lauronen@veripalvelu.fi	Clinical Task Forces (FinnGen phase 3)	**Kidney diseases Task Force**
Adrian Banerji	Institute for Molecular Medicine Finland (FIMM), HiLIFE, University of Helsinki, Helsinki, Finland; Broad Institute & Harvard Medical School, Cambridge, United States	adrian.banerji@childrens.harvard.edu	Clinical Task Forces (FinnGen phase 3)	**Kidney diseases Task Force**
Matthew Sampson	Broad Institute, Cambridge, MA, United States; Harvard Medical School, Cambridge, United States	matthew.sampson@childrens.harvard.edu	Clinical Task Forces (FinnGen phase 3)	**Kidney diseases Task Force**
Michelle McNulty	Institute for Molecular Medicine Finland (FIMM), HiLIFE, University of Helsinki, Helsinki, Finland; Broad Institute & Harvard Medical School, Cambridge, United States	michelle.mcnulty@childrens.harvard.edu	Clinical Task Forces (FinnGen phase 3)	**Kidney diseases Task Force**
Daniel Gordin	Helsinki University Hospital and University of Helsinki, Helsinki, Finland	daniel.gordin@helsinki.fi	Clinical Task Forces (FinnGen phase 3)	**Kidney diseases Task Force**
Patrik Finne	Helsinki University Hospital and University of Helsinki, Helsinki, Finland	patrik.finne@helsinki.fi	Clinical Task Forces (FinnGen phase 3)	**Kidney diseases Task Force**
Mika Kähönen	Finnish Clinical Biobank Tampere / University of Tampere / Wellbeing Services County of Pirkanmaa, Tampere, Finland	mika.kahonen@tuni.fi	Clinical Task Forces (FinnGen phase 3)	**Kidney diseases Task Force**
Tapio Hellman	University of Turku, Turku, Finland	tapio.hellman@tyks.fi	Clinical Task Forces (FinnGen phase 3)	**Kidney diseases Task Force**
Teemu Niiranen	University of Turku, Turku, Finland; Finnish Institute for Health and Welfare (THL), Helsinki, Finland	tejuni@utu.fi	Clinical Task Forces (FinnGen phase 3)	**Kidney diseases Task Force**
Dirk Paul	Astra Zeneca, Cambridge, United Kingdom	dirk.paul@astrazeneca.com	Clinical Task Forces (FinnGen phase 3)	**Kidney diseases Task Force**
Ioanna Tachmazidou	Astra Zeneca, Cambridge, United Kingdom	ioanna.tachmazidou@astrazeneca.com	Clinical Task Forces (FinnGen phase 3)	**Kidney diseases Task Force**
Hans van Leeuwen	Bayer AG, Leverkusen, Germany	hans.vanleeuwen@bayer.com	Clinical Task Forces (FinnGen phase 3)	**Kidney diseases Task Force**
Johanna Mielke	Bayer AG, Leverkusen, Germany	johanna.mielke@bayer.com	Clinical Task Forces (FinnGen phase 3)	**Kidney diseases Task Force**

Juho Immonen	Bayer AG, Leverkusen, Germany	juho.immonen@bayer.com	Clinical Task Forces (FinnGen phase 3)	**Kidney diseases Task Force**
Thomas Battram	Bayer AG, Leverkusen, Germany	thomas.battram@bayer.com	Clinical Task Forces (FinnGen phase 3)	**Kidney diseases Task Force**
Tobias Hogrebe	Bayer AG, Leverkusen, Germany	tobias.hogrebe@bayer.com	Clinical Task Forces (FinnGen phase 3)	**Kidney diseases Task Force**
Ketian Yu	Biogen, Cambridge, MA, United States	ketian.yu@biogen.com	Clinical Task Forces (FinnGen phase 3)	**Kidney diseases Task Force**
Benjamin Sun	Bristol Myers Squibb, New York, NY, United States	Benjamin.Sun@bms.com	Clinical Task Forces (FinnGen phase 3)	**Kidney diseases Task Force**
Janie Shelton	Bristol Myers Squibb, New York, NY, United States	janie.shelton@bms.com	Clinical Task Forces (FinnGen phase 3)	**Kidney diseases Task Force**
Yao Hu	Boehringer Ingelheim, Ingelheim am Rhein, Germany	yao.hu@boehringer-ingelheim.com	Clinical Task Forces (FinnGen phase 3)	**Kidney diseases Task Force**
Zhihao Ding	Boehringer Ingelheim, Ingelheim am Rhein, Germany	zhihao.ding@boehringer-ingelheim.com	Clinical Task Forces (FinnGen phase 3)	**Kidney diseases Task Force**
Rion Pendergrass	Genentech, San Francisco, CA, United States	pendergrass.rion@gene.com	Clinical Task Forces (FinnGen phase 3)	**Kidney diseases Task Force**
Sergio Dellepiane	Genentech, San Francisco, CA, United States	dellepis@gene.com	Clinical Task Forces (FinnGen phase 3)	**Kidney diseases Task Force**
Audrey Chu	GlaxoSmithKline, Collegeville, PA, United States	audrey.y.chu@gsk.com	Clinical Task Forces (FinnGen phase 3)	**Kidney diseases Task Force**
Chris Floyd	GlaxoSmithKline, Collegeville, PA, United States	christopher.n.floyd@gsk.com	Clinical Task Forces (FinnGen phase 3)	**Kidney diseases Task Force**
Dan Swerdlow	GlaxoSmithKline, Collegeville, PA, United States	daniel.8.swerdlow@gsk.com	Clinical Task Forces (FinnGen phase 3)	**Kidney diseases Task Force**
Erding Hu	GlaxoSmithKline, Collegeville, PA, United States	erding.hu@gsk.com	Clinical Task Forces (FinnGen phase 3)	**Kidney diseases Task Force**
Jonathan Davitte	GlaxoSmithKline, Collegeville, PA, United States	jonathan.m.davitte@gsk.com	Clinical Task Forces (FinnGen phase 3)	**Kidney diseases Task Force**
Prerak Desai	GlaxoSmithKline, Collegeville, PA, United States	prerak.x.desai@gsk.com	Clinical Task Forces (FinnGen phase 3)	**Kidney diseases Task Force**
Stephen Haddad	GlaxoSmithKline, Collegeville, PA, United States	stephen.a.haddad@gsk.com	Clinical Task Forces (FinnGen phase 3)	**Kidney diseases Task Force**
Dermot Reilly	Johnson & Johnson Innovative Medicine, Spring House, PA, United States	DReill11@its.jnj.com	Clinical Task Forces (FinnGen phase 3)	**Kidney diseases Task Force**
P. Dunnmon	Johnson & Johnson Innovative Medicine, Spring House, PA, United States	PDunnmon@ITS.JNJ.com	Clinical Task Forces (FinnGen phase 3)	**Kidney diseases Task Force**
Karol Estrada	Maze Therapeutics, San Francisco, CA, United States	kestrada@mazetx.com	Clinical Task Forces (FinnGen phase 3)	**Kidney diseases Task Force**
Rob Graham	Maze Therapeutics, San Francisco, CA, United States	rgraham@mazetx.com	Clinical Task Forces (FinnGen phase 3)	**Kidney diseases Task Force**
Sahar Mozzafari	Maze Therapeutics, San Francisco, CA, United States	smozaffari@mazetx.com	Clinical Task Forces (FinnGen phase 3)	**Kidney diseases Task Force**
Nancy Finkel	Novartis Institutes for BioMedical Research, Cambridge, MA, United States	nancy.finkel@novartis.com	Clinical Task Forces (FinnGen phase 3)	**Kidney diseases Task Force**
Sabina Pfister	Novartis Institutes for BioMedical Research, Cambridge, MA, United States	sabina.pfister@novartis.com	Clinical Task Forces (FinnGen phase 3)	**Kidney diseases Task Force**
Shola Richards	Novartis Institutes for BioMedical Research, Cambridge, MA, United States	shola.richards@novartis.com	Clinical Task Forces (FinnGen phase 3)	**Kidney diseases Task Force**
Joshua Chiou	Pfizer, New York, NY, United States	joshua.chiou@pfizer.com	Clinical Task Forces (FinnGen phase 3)	**Kidney diseases Task Force**
Ying Wu	Pfizer, New York, NY, United States	ying.wu3@pfizer.com	Clinical Task Forces (FinnGen phase 3)	**Kidney diseases Task Force**
Katherine Klinger	Translational Sciences, Sanofi R&D, Framingham, MA, USA	katherine.klinger@sanofi.com	Clinical Task Forces (FinnGen phase 3)	**Kidney diseases Task Force**
Matti Vuori	University of Turku, Turku, Finland	makvuo@utu.fi	Clinical Task Forces (FinnGen phase 3)	**Metabolic diseases Task Force**
Teemu Niiranen	University of Turku, Turku, Finland; Finnish Institute for Health and Welfare (THL), Helsinki, Finland	tejuni@utu.fi	Clinical Task Forces (FinnGen phase 3)	**Metabolic diseases Task Force**
Bridget Riley-Gillis	AbbVie, Chicago, IL, United States	bridget.rileygillis@abbvie.com	Clinical Task Forces (FinnGen phase 3)	**Metabolic diseases Task Force**
Nizar Smaoui	AbbVie, Chicago, IL, United States	nizar.smaoui@abbvie.com	Clinical Task Forces (FinnGen phase 3)	**Metabolic diseases Task Force**
Alix Berton	Bayer AG, Leverkusen, Germany	alix.berton@bayer.com	Clinical Task Forces (FinnGen phase 3)	**Metabolic diseases Task Force**
Hans van Leeuwen	Bayer AG, Leverkusen, Germany	hans.vanleeuwen@bayer.com	Clinical Task Forces (FinnGen phase 3)	**Metabolic diseases Task Force**
Chen Li	Bristol Myers Squibb, New York, NY, United States	Chen.Li@bms.com	Clinical Task Forces (FinnGen phase 3)	**Metabolic diseases Task Force**
Emily Holzinger	Bristol Myers Squibb, New York, NY, United States	Emily.Holzinger@bms.com	Clinical Task Forces (FinnGen phase 3)	**Metabolic diseases Task Force**
Anubha Mahajan	Genentech, San Francisco, CA, United States	mahajan.anubha@gene.com	Clinical Task Forces (FinnGen phase 3)	**Metabolic diseases Task Force**
Mark Mccarthy	Genentech, San Francisco, CA, United States	mccarthy.mark@gene.com	Clinical Task Forces (FinnGen phase 3)	**Metabolic diseases Task Force**

Christopher Deboever	Maze Therapeutics, San Francisco, CA, United States	cdeboever@mazetx.com	Clinical Task Forces (FinnGen phase 3)	**Metabolic diseases Task Force**
Karol Estrada	Maze Therapeutics, San Francisco, CA, United States	kestrada@mazetx.com	Clinical Task Forces (FinnGen phase 3)	**Metabolic diseases Task Force**
Robert Graham	Maze Therapeutics, San Francisco, CA, United States	rgraham@mazetx.com	Clinical Task Forces (FinnGen phase 3)	**Metabolic diseases Task Force**
Hye In Kim	Pfizer, New York, NY, United States	HyeIn.Kim@pfizer.com	Clinical Task Forces (FinnGen phase 3)	**Metabolic diseases Task Force**
Sivakumar Pitchumani	Pfizer, New York, NY, United States	Pitchumani.Sivakumar@pfizer.com	Clinical Task Forces (FinnGen phase 3)	**Metabolic diseases Task Force**
Sumedha Jassal	Pfizer, New York, NY, United States	Sumedha.Jassal@pfizer.com	Clinical Task Forces (FinnGen phase 3)	**Metabolic diseases Task Force**
Åsa Hedman	Pfizer, New York, NY, United States	asa.Hedman@pfizer.com	Clinical Task Forces (FinnGen phase 3)	**Metabolic diseases Task Force**
Aarno Palotie	Institute for Molecular Medicine Finland (FIMM), HiLIFE, University of Helsinki, Helsinki, Finland; Broad Institute of MIT and Harvard; Massachusetts General Hospital, Boston, MA, United States	aarno.palotie@helsinki.fi	Clinical Task Forces (FinnGen phase 3)	**Neurodegenerative diseases Task Force**
Austin Argentieri	Institute for Molecular Medicine Finland (FIMM), HiLIFE, University of Helsinki, Helsinki, Finland; Broad Institute, Cambridge, MA, United States	aargentieri@mgh.harvard.edu	Clinical Task Forces (FinnGen phase 3)	**Neurodegenerative diseases Task Force**
Aoxing Liu	Institute for Molecular Medicine Finland (FIMM), HiLIFE, University of Helsinki, Helsinki, Finland	aoxing.liu@helsinki.fi	Clinical Task Forces (FinnGen phase 3)	**Neurodegenerative diseases Task Force**
Eero Vuoksimaa	Institute for Molecular Medicine Finland (FIMM), HiLIFE, University of Helsinki, Helsinki, Finland	eero.vuoksimaa@helsinki.fi	Clinical Task Forces (FinnGen phase 3)	**Neurodegenerative diseases Task Force**
Elisa Lahtela	Institute for Molecular Medicine Finland (FIMM), HiLIFE, University of Helsinki, Helsinki, Finland	laura.lahtela@helsinki.fi	Clinical Task Forces (FinnGen phase 3)	**Neurodegenerative diseases Task Force**
Joni Lindbohm	Institute for Molecular Medicine Finland (FIMM), HiLIFE, University of Helsinki, Helsinki, Finland	joni.lindbohm@helsinki.fi	Clinical Task Forces (FinnGen phase 3)	**Neurodegenerative diseases Task Force**
Mark Daly	Institute for Molecular Medicine Finland (FIMM), HiLIFE, University of Helsinki, Helsinki, Finland; Broad Institute of MIT and Harvard; Massachusetts General Hospital, Boston, MA, United States	mjdaly@broadinstitute.com	Clinical Task Forces (FinnGen phase 3)	**Neurodegenerative diseases Task Force**
Mary Pat Reeve	Institute for Molecular Medicine Finland (FIMM), HiLIFE, University of Helsinki, Helsinki, Finland; Broad Institute, Cambridge, MA, United States	mary.reeve@helsinki.fi	Clinical Task Forces (FinnGen phase 3)	**Neurodegenerative diseases Task Force**
Paavo Häppölä	Institute for Molecular Medicine Finland (FIMM), HiLIFE, University of Helsinki, Helsinki, Finland	paavo.happola@helsinki.fi	Clinical Task Forces (FinnGen phase 3)	**Neurodegenerative diseases Task Force**
Zhiyu Yang	Institute for Molecular Medicine Finland (FIMM), HiLIFE, University of Helsinki, Helsinki, Finland	zhiyu.yang@helsinki.fi	Clinical Task Forces (FinnGen phase 3)	**Neurodegenerative diseases Task Force**
Eino Solje	University of Eastern Finland, Kuopio, Finland	eino.solje@uef.fi	Clinical Task Forces (FinnGen phase 3)	**Neurodegenerative diseases Task Force**
Mikko Hiltunen	University of Eastern Finland, Kuopio, Finland	mikko.hiltunen@uef.fi	Clinical Task Forces (FinnGen phase 3)	**Neurodegenerative diseases Task Force**
Valtteri Julkunen	University of Eastern Finland and Kuopio University Hospital, Kuopio, Finland	Valtteri.Julkunen@pshyvinvointialue.fi	Clinical Task Forces (FinnGen phase 3)	**Neurodegenerative diseases Task Force**
Ville Leinonen	University of Eastern Finland and Kuopio University Hospital, Kuopio, Finland	ville.leinonen@pshyvinvointialue.fi	Clinical Task Forces (FinnGen phase 3)	**Neurodegenerative diseases Task Force**
Hanna Kujala	Biobank of Eastern Finland / University of Eastern Finland / Wellbeing services county of North Savo, Kuopio, Finland	Hanna.Kujala@pshyvinvointialue.fi	Clinical Task Forces (FinnGen phase 3)	**Neurodegenerative diseases Task Force**
Aki Havulinna	Finnish Institute for Health and Welfare (THL), Helsinki, Finland	aki.havulinna@thl.fi	Clinical Task Forces (FinnGen phase 3)	**Neurodegenerative diseases Task Force**
Roosa Kallionpää	University of Turku, Turku, Finland	roosa.kallionpaa@tyks.fi	Clinical Task Forces (FinnGen phase 3)	**Neurodegenerative diseases Task Force**
Minttu Marttila	University of Helsinki, Helsinki, Finland	minttu.marttila@helsinki.fi	Clinical Task Forces (FinnGen phase 3)	**Neurodegenerative diseases Task Force**
Britney Milkovich	AbbVie, Chicago, IL, United States	britney.milkovich@abbvie.com	Clinical Task Forces (FinnGen phase 3)	**Neurodegenerative diseases Task Force**
Jan Freudenberg	AbbVie, Chicago, IL, United States	jan.freudenberg@abbvie.com	Clinical Task Forces (FinnGen phase 3)	**Neurodegenerative diseases Task Force**
Andrew Lowe	Astra Zeneca, Cambridge, United Kingdom	andrew.lowe@astrazeneca.com	Clinical Task Forces (FinnGen phase 3)	**Neurodegenerative diseases Task Force**
Ioanna Tachmazidou	Astra Zeneca, Cambridge, United Kingdom	ioanna.tachmazidou@astrazeneca.com	Clinical Task Forces (FinnGen phase 3)	**Neurodegenerative diseases Task Force**
Thomas Spargo	Astra Zeneca, Cambridge, United Kingdom	thomas.spargo@astrazeneca.com	Clinical Task Forces (FinnGen phase 3)	**Neurodegenerative diseases Task Force**
Kritika Singh	Bristol Myers Squibb, New York, NY, United States	Kritika.Singh3@bms.com	Clinical Task Forces (FinnGen phase 3)	**Neurodegenerative diseases Task Force**
Peng Jiang	Bristol Myers Squibb, New York, NY, United States	Peng.Jiang@bms.com	Clinical Task Forces (FinnGen phase 3)	**Neurodegenerative diseases Task Force**
Stephanie Loomis	Bristol Myers Squibb, New York, NY, United States	stephanie.loomis@bms.com	Clinical Task Forces (FinnGen phase 3)	**Neurodegenerative diseases Task Force**
Anubha Mahajan	Genentech, San Francisco, CA, United States	mahaja11@gene.com	Clinical Task Forces (FinnGen phase 3)	**Neurodegenerative diseases Task Force**
Rion Pendergrass	Genentech, San Francisco, CA, United States	penders2@gene.com	Clinical Task Forces (FinnGen phase 3)	**Neurodegenerative diseases Task Force**

Damien Croteau-Chonka	GlaxoSmithKline, Collegeville, PA, United States	damien.c.croteau-chonka@gsk.com	**Clinical Task Forces (FinnGen phase 3)**	**Neurodegenerative diseases Task Force**
John Eicher	GlaxoSmithKline, Collegeville, PA, United States	john.d.eicher@gsk.com	**Clinical Task Forces (FinnGen phase 3)**	**Neurodegenerative diseases Task Force**
Prerak Desai	GlaxoSmithKline, Collegeville, PA, United States	prerak.x.desai@gsk.com	**Clinical Task Forces (FinnGen phase 3)**	**Neurodegenerative diseases Task Force**
Chris Whelan	Johnson & Johnson Innovative Medicine, Spring House, PA, United States	CWhelan4@ITS.JNJ.com	**Clinical Task Forces (FinnGen phase 3)**	**Neurodegenerative diseases Task Force**
Karen He	Johnson & Johnson Innovative Medicine, Spring House, PA, United States	KHe2@ITS.JNJ.com	**Clinical Task Forces (FinnGen phase 3)**	**Neurodegenerative diseases Task Force**
Qingqin Li	Johnson & Johnson Innovative Medicine, Spring House, PA, United States	qli2@its.jnj.com	**Clinical Task Forces (FinnGen phase 3)**	**Neurodegenerative diseases Task Force**
W Galpern	Johnson & Johnson Innovative Medicine, Spring House, PA, United States	wgalpern@ITS.JNJ.com	**Clinical Task Forces (FinnGen phase 3)**	**Neurodegenerative diseases Task Force**
Yanfei Zhang	Johnson & Johnson Innovative Medicine, Spring House, PA, United States	yzhan378@its.jnj.com	**Clinical Task Forces (FinnGen phase 3)**	**Neurodegenerative diseases Task Force**
Andrei Popescu	Merck, Kenilworth, NJ, United States	andrei.popescu@merck.com	**Clinical Task Forces (FinnGen phase 3)**	**Neurodegenerative diseases Task Force**
Delphine Fagegaltier	Merck, Kenilworth, NJ, United States	delphine.fagegaltier@merck.com	**Clinical Task Forces (FinnGen phase 3)**	**Neurodegenerative diseases Task Force**
Mari Niemi	Novartis Institutes for BioMedical Research, Cambridge, MA, United States	Mari.niemi@novartis.com	**Clinical Task Forces (FinnGen phase 3)**	**Neurodegenerative diseases Task Force**
Nikos Patsopoulos	Novartis Institutes for BioMedical Research, Cambridge, MA, United States	nikos.patsopoulos@novartis.com	**Clinical Task Forces (FinnGen phase 3)**	**Neurodegenerative diseases Task Force**
Katherine Klinger	Translational Sciences, Sanofi R&D, Framingham, MA, USA	katherine.klinger@sanofi.com	**Clinical Task Forces (FinnGen phase 3)**	**Neurodegenerative diseases Task Force**
Aarno Palotie	Institute for Molecular Medicine Finland (FIMM), HiLIFE, University of Helsinki, Helsinki, Finland; Broad Institute of MIT and Harvard; Massachusetts General Hospital, Boston, MA, United States	aarno.palotie@helsinki.fi	**Clinical Task Forces (FinnGen phase 3)**	**Pulmonology Task Force and fibrotic diseases interest group**
Elisa Lahtela	Institute for Molecular Medicine Finland (FIMM), HiLIFE, University of Helsinki, Helsinki, Finland	laura.lahtela@helsinki.fi	**Clinical Task Forces (FinnGen phase 3)**	**Pulmonology Task Force and fibrotic diseases interest group**
Jukka Koskela	Institute for Molecular Medicine Finland (FIMM), HiLIFE, University of Helsinki, Helsinki, Finland	jukka.koskela@helsinki.fi	**Clinical Task Forces (FinnGen phase 3)**	**Pulmonology Task Force and fibrotic diseases interest group**
Mark Daly	Institute for Molecular Medicine Finland (FIMM), HiLIFE, University of Helsinki, Helsinki, Finland; Broad Institute of MIT and Harvard; Massachusetts General Hospital, Boston, MA, United States	mjdaly@broadinstitute.org	**Clinical Task Forces (FinnGen phase 3)**	**Pulmonology Task Force and fibrotic diseases interest group**
Sanni Ruotsalainen	Institute for Molecular Medicine Finland (FIMM), HiLIFE, University of Helsinki, Helsinki, Finland	sanni.ruotsalainen@helsinki.fi	**Clinical Task Forces (FinnGen phase 3)**	**Pulmonology Task Force and fibrotic diseases interest group**
Susanna Lemmelä	Institute for Molecular Medicine Finland (FIMM), HiLIFE, University of Helsinki, Helsinki, Finland	susanna.lemmela@helsinki.fi	**Clinical Task Forces (FinnGen phase 3)**	**Pulmonology Task Force and fibrotic diseases interest group**
Tarja Laitinen	Institute for Molecular Medicine Finland (FIMM), HiLIFE, University of Helsinki, Helsinki, Finland	tarja.laitinen@helsinki.fi	**Clinical Task Forces (FinnGen phase 3)**	**Pulmonology Task Force and fibrotic diseases interest group**
Salla Ranta	Hospital District of Helsinki and Uusimaa, Helsinki, Finland	salla.a.ranta@hus.fi	**Clinical Task Forces (FinnGen phase 3)**	**Pulmonology Task Force and fibrotic diseases interest group**
Paavo Häppölä	Institute for Molecular Medicine Finland (FIMM), HiLIFE, University of Helsinki, Helsinki, Finland	paavo.happola@helsinki.fi	**Clinical Task Forces (FinnGen phase 3)**	**Pulmonology Task Force and fibrotic diseases interest group**
Paula Kauppi	Hospital District of Helsinki and Uusimaa, Helsinki, Finland	paula.kauppi@hus.fi	**Clinical Task Forces (FinnGen phase 3)**	**Pulmonology Task Force and fibrotic diseases interest group**
Tiinamaija Tuomi	Institute for Molecular Medicine Finland (FIMM), HiLIFE, University of Helsinki, Helsinki, Finland; Hospital District of Helsinki and Uusimaa, Helsinki, Finland	tiinamaija.tuomi@hus.fi	**Clinical Task Forces (FinnGen phase 3)**	**Pulmonology Task Force and fibrotic diseases interest group**
Raisa Serpi	Northern Finland Biobank Borealis / University of Oulu / Wellbeing services county of North Ostrobothnia, Oulu, Finland	Raisa.Serpi@ppshp.fi	**Clinical Task Forces (FinnGen phase 3)**	**Pulmonology Task Force and fibrotic diseases interest group**
Riitta Kaarteenaho	University of Oulu, Oulu, Finland	riitta.kaarteenaho@oulu.fi	**Clinical Task Forces (FinnGen phase 3)**	**Pulmonology Task Force and fibrotic diseases interest group**
Hannu Kankaanranta	University of Gothenburg, Gothenburg, Sweden/ Seinäjoki Central Hospital, Seinäjoki, Finland/ Tampere University, Tampere, Finland	hannu.kankaanranta@tuni.fi	**Clinical Task Forces (FinnGen phase 3)**	**Pulmonology Task Force and fibrotic diseases interest group**
Coralie Viollet	Astra Zeneca, Cambridge, United Kingdom	coralie.viollet@astrazeneca.com	**Clinical Task Forces (FinnGen phase 3)**	**Pulmonology Task Force and fibrotic diseases interest group**
Eleanor Wheeler	Astra Zeneca, Cambridge, United Kingdom	eleanor.wheeler@astrazeneca.com	**Clinical Task Forces (FinnGen phase 3)**	**Pulmonology Task Force and fibrotic diseases interest group**

Oliver Burren	Astra Zeneca, Cambridge, United Kingdom	oliver.burren@astrazeneca.com	Clinical Task Forces (FinnGen phase 3)	**Pulmonology Task Force and fibrotic diseases interest group**
Christoph Ogris	Boehringer Ingelheim, Ingelheim am Rhein, Germany	christoph.ogris@boehringer-ingelheim.com	Clinical Task Forces (FinnGen phase 3)	**Pulmonology Task Force and fibrotic diseases interest group**
Eric Simon	Boehringer Ingelheim, Ingelheim am Rhein, Germany	eric.simon@boehringer-ingelheim.com	Clinical Task Forces (FinnGen phase 3)	**Pulmonology Task Force and fibrotic diseases interest group**
Frank LI	Boehringer Ingelheim, Ingelheim am Rhein, Germany	frank.li@boehringer-ingelheim.com	Clinical Task Forces (FinnGen phase 3)	**Pulmonology Task Force and fibrotic diseases interest group**
Julio Cesar Bolivar Lopez	Boehringer Ingelheim, Ingelheim am Rhein, Germany	julio_cesar.bolivar_lopez@boehringer-ingelheim.com	Clinical Task Forces (FinnGen phase 3)	**Pulmonology Task Force and fibrotic diseases interest group**
Yao Hu	Boehringer Ingelheim, Ingelheim am Rhein, Germany	yao.hu@boehringer-ingelheim.com	Clinical Task Forces (FinnGen phase 3)	**Pulmonology Task Force and fibrotic diseases interest group**
Zhihao Ding	Boehringer Ingelheim, Ingelheim am Rhein, Germany	zhihao.ding@boehringer-ingelheim.com	Clinical Task Forces (FinnGen phase 3)	**Pulmonology Task Force and fibrotic diseases interest group**
Elena Sanchez	Bristol Myers Squibb, New York, NY, United States	elena.sanchez2@bms.com	Clinical Task Forces (FinnGen phase 3)	**Pulmonology Task Force and fibrotic diseases interest group**
Emily Holzinger	Bristol Myers Squibb, New York, NY, United States	Emily.Holzinger@bms.com	Clinical Task Forces (FinnGen phase 3)	**Pulmonology Task Force and fibrotic diseases interest group**
Joe Maranville	Bristol Myers Squibb, New York, NY, United States	joseph.maranville@bms.com	Clinical Task Forces (FinnGen phase 3)	**Pulmonology Task Force and fibrotic diseases interest group**
Lilith Moss	Bristol Myers Squibb, New York, NY, United States	Lilith.moss@bms.com	Clinical Task Forces (FinnGen phase 3)	**Pulmonology Task Force and fibrotic diseases interest group**
Michael Turchin	Bristol Myers Squibb, New York, NY, United States	michael.turchin@bms.com	Clinical Task Forces (FinnGen phase 3)	**Pulmonology Task Force and fibrotic diseases interest group**
Zijie Zhao	Bristol Myers Squibb, New York, NY, United States	Zijie.Zhao@bms.com	Clinical Task Forces (FinnGen phase 3)	**Pulmonology Task Force and fibrotic diseases interest group**
Diana Chang	Genentech, San Francisco, CA, United States	chang.diana@gene.com	Clinical Task Forces (FinnGen phase 3)	**Pulmonology Task Force and fibrotic diseases interest group**
Audrey Chu	GlaxoSmithKline, Collegeville, PA, United States	audrey.y.chu@gsk.com	Clinical Task Forces (FinnGen phase 3)	**Pulmonology Task Force and fibrotic diseases interest group**
Billy Fahy	GlaxoSmithKline, Collegeville, PA, United States	william.a.fahy@gsk.com	Clinical Task Forces (FinnGen phase 3)	**Pulmonology Task Force and fibrotic diseases interest group**
Jessica Chao	GlaxoSmithKline, Collegeville, PA, United States	jessica.x.chao@gsk.com	Clinical Task Forces (FinnGen phase 3)	**Pulmonology Task Force and fibrotic diseases interest group**
Joanna Betts	GlaxoSmithKline, Collegeville, PA, United States	joanna.c.betts@gsk.com	Clinical Task Forces (FinnGen phase 3)	**Pulmonology Task Force and fibrotic diseases interest group**
Jonathan Davitte	GlaxoSmithKline, Collegeville, PA, United States	jonathan.m.davitte@gsk.com	Clinical Task Forces (FinnGen phase 3)	**Pulmonology Task Force and fibrotic diseases interest group**
Paola Bronson	GlaxoSmithKline, Collegeville, PA, United States	paola.g.bronson@gsk.com	Clinical Task Forces (FinnGen phase 3)	**Pulmonology Task Force and fibrotic diseases interest group**
Prerak Desai	GlaxoSmithKline, Collegeville, PA, United States	prerak.x.desai@gsk.com	Clinical Task Forces (FinnGen phase 3)	**Pulmonology Task Force and fibrotic diseases interest group**
Dermot Reilly	Johnson & Johnson Innovative Medicine, Spring House, PA, United States	DReill11@its.jnj.com	Clinical Task Forces (FinnGen phase 3)	**Pulmonology Task Force and fibrotic diseases interest group**
Mona Selej	Johnson & Johnson Innovative Medicine, Spring House, PA, United States	mselej@its.jnj.com	Clinical Task Forces (FinnGen phase 3)	**Pulmonology Task Force and fibrotic diseases interest group**
P Dunnmon	Johnson & Johnson Innovative Medicine, Spring House, PA, United States	PDunnmon@ITS.JNJ.com	Clinical Task Forces (FinnGen phase 3)	**Pulmonology Task Force and fibrotic diseases interest group**
Jorge Del-aguila	Merck, Kenilworth, NJ, United States	jorge.del-aguila@merck.com	Clinical Task Forces (FinnGen phase 3)	**Pulmonology Task Force and fibrotic diseases interest group**

Jozsef Karman	Merck, Kenilworth, NJ, United States	jozsef.karman@merck.com	Clinical Task Forces (FinnGen phase 3)	**Pulmonology Task Force and fibrotic diseases interest group**
Travis Barr	Merck, Kenilworth, NJ, United States	travis.barr@merck.com	Clinical Task Forces (FinnGen phase 3)	**Pulmonology Task Force and fibrotic diseases interest group**
Katherine Mccauley	Novartis Institutes for BioMedical Research, Cambridge, MA, United States	katherine.mccauley@novartis.com	Clinical Task Forces (FinnGen phase 3)	**Pulmonology Task Force and fibrotic diseases interest group**
Xiaobo Xia	Novartis Institutes for BioMedical Research, Cambridge, MA, United States	xiaobo.xia@novartis.com	Clinical Task Forces (FinnGen phase 3)	**Pulmonology Task Force and fibrotic diseases interest group**
Madhurima Saxena	Pfizer, New York, NY, United States	Madhurima.Saxena@pfizer.com	Clinical Task Forces (FinnGen phase 3)	**Pulmonology Task Force and fibrotic diseases interest group**
Pitchumani Sivakumar	Pfizer, New York, NY, United States	Pitchumani.Sivakumar@pfizer.com	Clinical Task Forces (FinnGen phase 3)	**Pulmonology Task Force and fibrotic diseases interest group**
Sumedha Jassal	Pfizer, New York, NY, United States	sumedha.jassal@pfizer.com	Clinical Task Forces (FinnGen phase 3)	**Pulmonology Task Force and fibrotic diseases interest group**
David Habiel	Translational Sciences, Sanofi R&D, Framingham, MA, USA	david.habiel@sanofi.com	Clinical Task Forces (FinnGen phase 3)	**Pulmonology Task Force and fibrotic diseases interest group**
Guanling Huan	Translational Sciences, Sanofi R&D, Framingham, MA, USA	guanling.huang@sanofi.com	Clinical Task Forces (FinnGen phase 3)	**Pulmonology Task Force and fibrotic diseases interest group**
Marika Kaakinen	Institute for Molecular Medicine Finland (FIMM), HiLIFE, University of Helsinki, Helsinki, Finland	marika.kaakinen@helsinki.fi	Clinical Task Forces (FinnGen phase 3)	**Parkinson´s disease Task Force**
Mary Pat Reeve	Institute for Molecular Medicine Finland (FIMM), HiLIFE, University of Helsinki, Helsinki, Finland; Broad Institute, Cambridge, MA, United States	mary.reeve@helsinki.fi	Clinical Task Forces (FinnGen phase 3)	**Parkinson´s disease Task Force**
Filip Scheperjans	Hospital District of Helsinki and Uusimaa, Helsinki, Finland	Filip.Scheperjans@hus.fi	Clinical Task Forces (FinnGen phase 3)	**Parkinson´s disease Task Force**
Andrew Blumenfeld	AbbVie, Chicago, IL, United States	andrew.blumenfeld@abbvie.com	Clinical Task Forces (FinnGen phase 3)	**Parkinson´s disease Task Force**
Britney Milkovich	AbbVie, Chicago, IL, United States	Britney.Milkovich@abbvie.com	Clinical Task Forces (FinnGen phase 3)	**Parkinson´s disease Task Force**
Jan Freudenberg	AbbVie, Chicago, IL, United States	Jan.Freudenberg@abbvie.com	Clinical Task Forces (FinnGen phase 3)	**Parkinson´s disease Task Force**
Tushar Kumar	AbbVie, Chicago, IL, United States	tushar.kumar@abbvie.com	Clinical Task Forces (FinnGen phase 3)	**Parkinson´s disease Task Force**
Hans van Leeuwen	Bayer AG, Leverkusen, Germany	hans.vanleeuwen@bayer.com	Clinical Task Forces (FinnGen phase 3)	**Parkinson´s disease Task Force**
Juho Immonen	Bayer AG, Leverkusen, Germany	juho.immonen@bayer.com	Clinical Task Forces (FinnGen phase 3)	**Parkinson´s disease Task Force**
Samu Kurki	Bayer AG, Leverkusen, Germany	samu.kurki@bayer.com	Clinical Task Forces (FinnGen phase 3)	**Parkinson´s disease Task Force**
Coro Paisan-Ruiz	Biogen, Cambridge, MA, United States	coro.paisanruiz@biogen.com	Clinical Task Forces (FinnGen phase 3)	**Parkinson´s disease Task Force**
Anna Podgornaia	Bristol Myers Squibb, New York, NY, United States	Anna.Podgornaia@bms.com	Clinical Task Forces (FinnGen phase 3)	**Parkinson´s disease Task Force**
Benjamin Sun	Bristol Myers Squibb, New York, NY, United States	Benjamin.Sun@bms.com	Clinical Task Forces (FinnGen phase 3)	**Parkinson´s disease Task Force**
Janie Shelton	Bristol Myers Squibb, New York, NY, United States	Janie.Shelton@bms.com	Clinical Task Forces (FinnGen phase 3)	**Parkinson´s disease Task Force**
Peng Jiang	Bristol Myers Squibb, New York, NY, United States	Peng.Jiang@bms.com	Clinical Task Forces (FinnGen phase 3)	**Parkinson´s disease Task Force**
Stephanie Loomis	Bristol Myers Squibb, New York, NY, United States	stephanie.loomis@bms.com	Clinical Task Forces (FinnGen phase 3)	**Parkinson´s disease Task Force**
Tushar Bhangale	Genentech, San Francisco, CA, United States	bhangale.tushar@gene.com	Clinical Task Forces (FinnGen phase 3)	**Parkinson´s disease Task Force**
John Eicher	GlaxoSmithKline, Collegeville, PA, United States	john.d.eicher@gsk.com	Clinical Task Forces (FinnGen phase 3)	**Parkinson´s disease Task Force**
Abolfazl Doostparast Torshizi	Johnson & Johnson Innovative Medicine, Spring House, PA, United States	ADoostpa@ITS.JNJ.com	Clinical Task Forces (FinnGen phase 3)	**Parkinson´s disease Task Force**
Aristide Merola	Merck, Kenilworth, NJ, United States	aristide.merola@merck.com	Clinical Task Forces (FinnGen phase 3)	**Parkinson´s disease Task Force**
Oliver Freeman	Merck, Kenilworth, NJ, United States	oliver.freeman@msd.com	Clinical Task Forces (FinnGen phase 3)	**Parkinson´s disease Task Force**
Simonne Longerich	Merck, Kenilworth, NJ, United States	simonne.longerich@merck.com	Clinical Task Forces (FinnGen phase 3)	**Parkinson´s disease Task Force**
Mari Niemi	Novartis Institutes for BioMedical Research, Cambridge, MA, United States	mari.niemi@novartis.com	Clinical Task Forces (FinnGen phase 3)	**Parkinson´s disease Task Force**
Katherine Klinger	Translational Sciences, Sanofi R&D, Framingham, MA, USA	Katherine.Klinger@sanofi.com	Clinical Task Forces (FinnGen phase 3)	**Parkinson´s disease Task Force**

2. Supplementary Figures

QQ Plot for main ANX GWAS

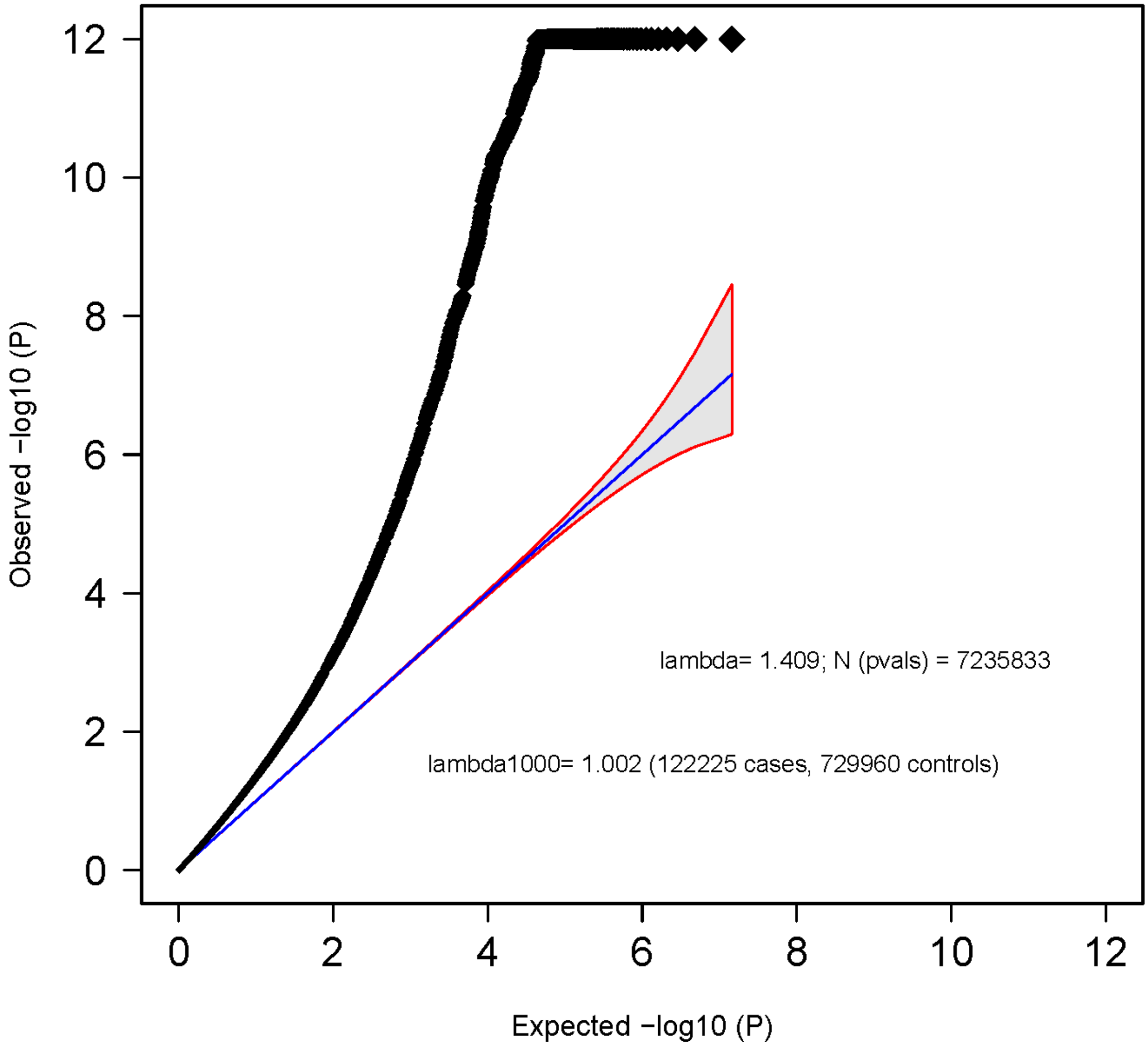

Supplementary Figure 1: Quantile-quantile (QQ) plot of the PGC-ANX1 GWAS. The expected -log10(p) under the null (blue line) is plotted against the observed -log10(p) (black dots). The shading indicates the 95% confidence region under the null. Lambda and Lambda1000 (which is the Lambda if the GWAS contained 1000 cases and 1000 controls) indicate genomic inflation factors. Number of SNPs (N (pvals) = 7,235,833) and Number of cases (n = 122,225) and controls (n = 729,960) are given in parentheses.

Regional association plots and forest plots of the 58 independent significant SNPs

Supplementary Figure 2-56: Shown are regional association plots (A) and forest plots (B) separately for each independently ANX associated SNP (from the main meta-analysis Ncases =122,225 , Ncontrols = 729,960). In the regional association plot (A), the –log10 p-values (from two-sided association tests) are shown on the left y-axis. The recombination rate is expressed in centimorgans (cM) per Mb (Megabase) (blue line) and is shown on the right y-axis. Position in Mb is on the x-axis, with genes shown below the regional association plot. Only SNPs with an association p-value less than 0.1 were plotted. The SNP with the lowest p-value in the region is shown as a diamond and marked with the letter a. The forest plot (B) shows the imputation quality (INFO) score, p-value of the SNP association (two-sided and not corrected for multiple testing), allele frequency in cases with case sample size (f_ca(n)), allele frequency in controls and control sample size (f_co(n)), beta estimates (ln(OR)) and standard error (STDerr) for each study as well as for the combined meta-analysis. In the case of two independent loci in the same region, a second forest plot is shown in C).

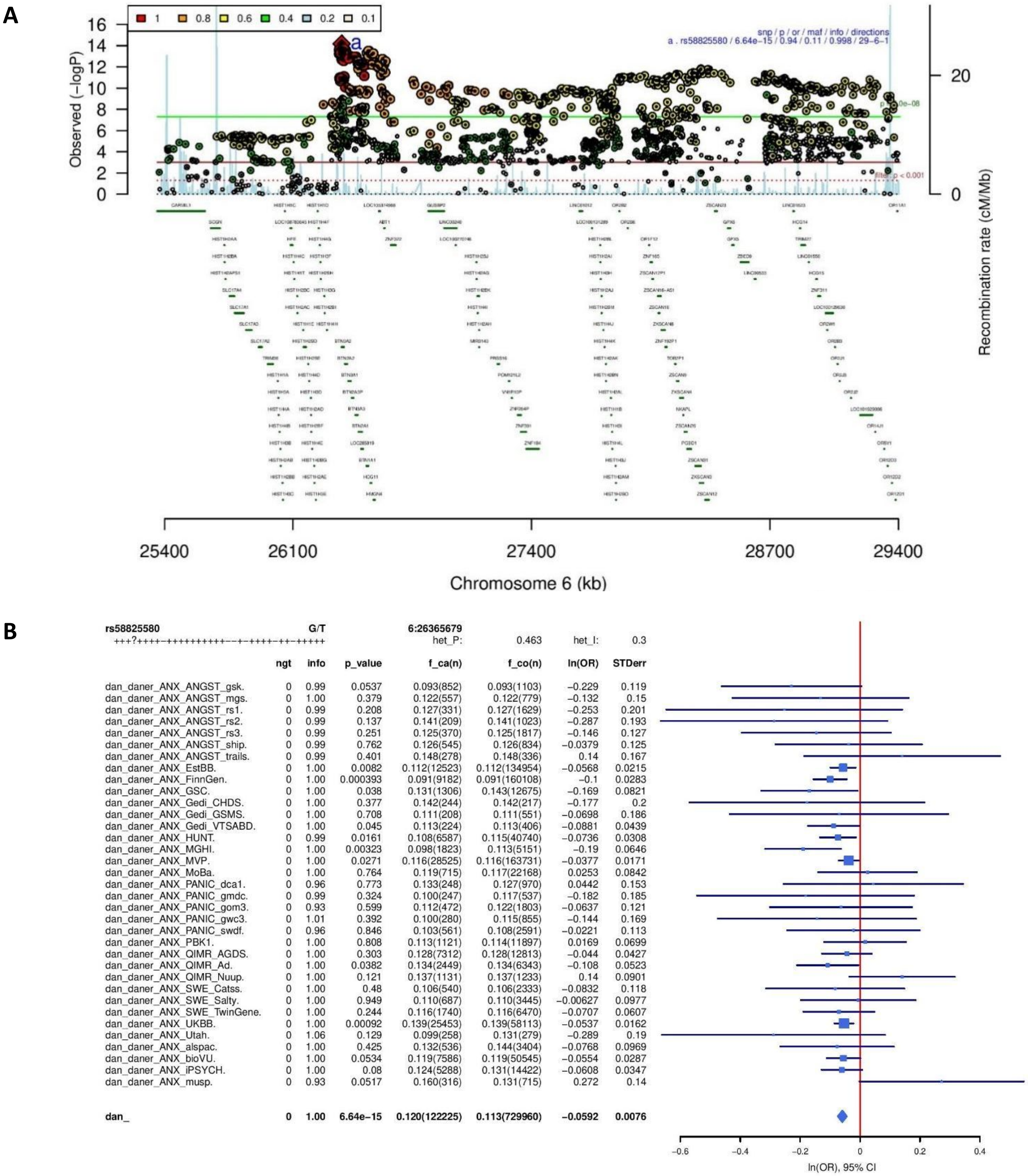

Supplementary Figure 2: Regional association plot (A) and forest plot (B) of SNP rs58825580. See full figure description on page 30.

A

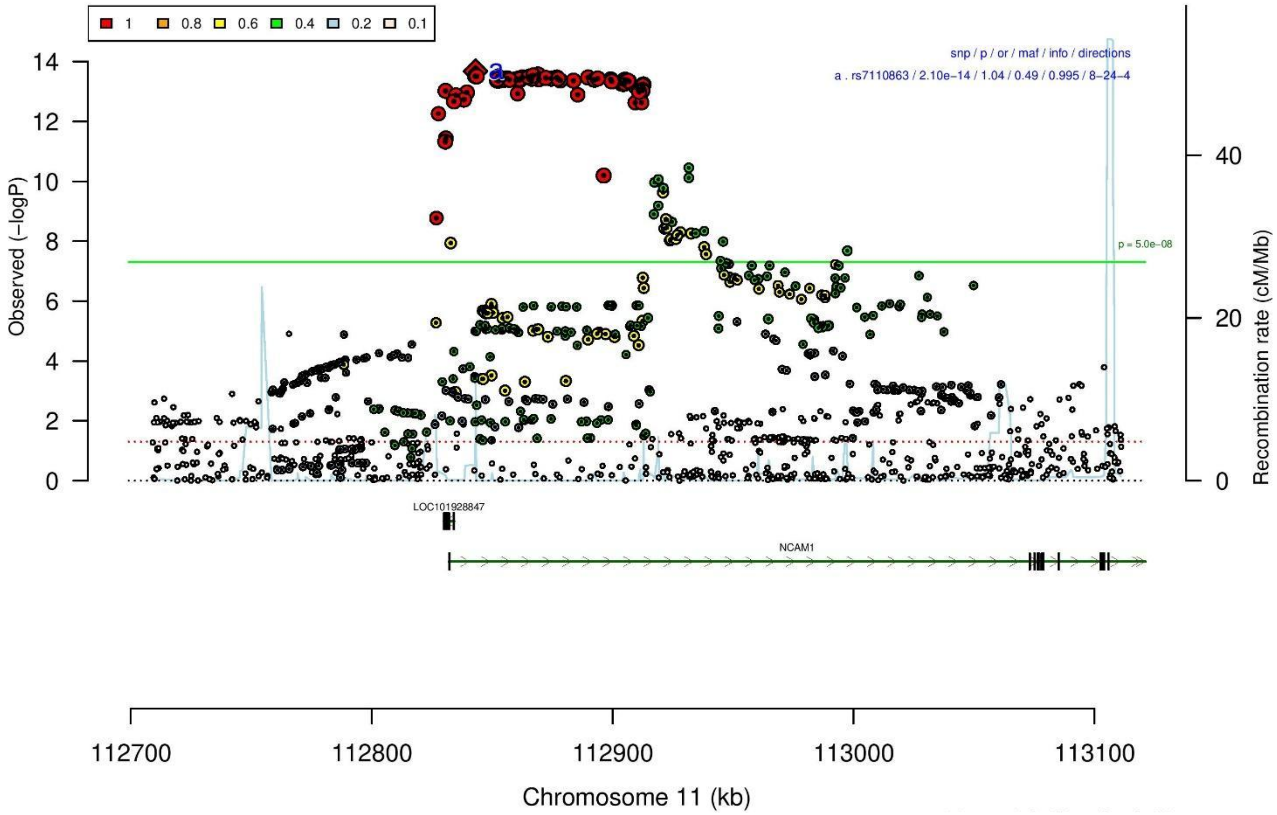

B

rs7110863		G/A		11:112843138			
+?−?+−−+−?−−++−−−−−?+−+−−−+−−−−−−−−−				het_P:	0.99	het_I:	0.0
	ngt	info	p_value	f_ca(n)	f_co(n)	ln(OR)	STDerr
dan_daner_ANX_ANGST_mgs.	0	1.00	0.172	0.391(557)	0.391(779)	0.114	0.0833
dan_daner_ANX_ANGST_rs1.	0	1.00	0.363	0.390(331)	0.390(1629)	−0.092	0.101
dan_daner_ANX_ANGST_rs2.	0	1.00	0.355	0.389(209)	0.389(1023)	0.102	0.111
dan_daner_ANX_ANGST_rs3.	0	1.00	0.451	0.389(370)	0.389(1817)	0.0614	0.0814
dan_daner_ANX_ANGST_ship.	0	1.00	0.833	0.392(545)	0.392(834)	−0.0178	0.0847
dan_daner_ANX_ANGST_trails.	0	1.00	0.374	0.385(278)	0.385(336)	0.108	0.121
dan_daner_ANX_EstBB.	0	0.99	0.0432	0.550(12523)	0.550(134954)	0.0274	0.0135
dan_daner_ANX_FinnGen.	0	1.00	0.0212	0.624(9182)	0.624(160108)	0.037	0.0161
dan_daner_ANX_GSC.	0	1.01	0.45	0.396(1306)	0.391(12675)	0.0325	0.0429
dan_daner_ANX_Gedi_CHDS.	0	1.00	0.985	0.376(244)	0.376(217)	−0.00265	0.137
dan_daner_ANX_Gedi_GSMS.	0	1.00	0.113	0.395(208)	0.395(551)	−0.195	0.123
dan_daner_ANX_Gedi_VTSABD.	0	1.00	0.194	0.399(224)	0.399(406)	0.0381	0.0293
dan_daner_ANX_HUNT.	0	0.99	0.00578	0.416(6587)	0.400(40740)	0.0543	0.0196
dan_daner_ANX_MGHI.	0	0.98	0.679	0.400(1823)	0.397(5151)	0.0165	0.0398
dan_daner_ANX_MVP.	0	1.00	7.68e−05	0.410(28525)	0.410(163731)	0.0385	0.0097
dan_daner_ANX_PANIC_dca1.	0	0.98	0.921	0.401(248)	0.400(970)	−0.0104	0.105
dan_daner_ANX_PANIC_gmdc.	1	0.98	0.202	0.413(247)	0.390(537)	0.148	0.116
dan_daner_ANX_PANIC_gom3.	0	0.96	0.853	0.411(472)	0.403(1803)	0.0146	0.0783
dan_daner_ANX_PANIC_gwc3.	1	1.05	0.48	0.438(280)	0.408(855)	0.0722	0.102
dan_daner_ANX_PANIC_swdf.	0	0.96	0.865	0.439(561)	0.438(2591)	−0.0118	0.0701
dan_daner_ANX_PBK1.	0	1.00	0.161	0.404(1121)	0.390(11897)	0.0639	0.0457
dan_daner_ANX_QIMR_AGDS.	0	0.94	0.523	0.387(7312)	0.387(12813)	0.0194	0.0304
dan_daner_ANX_QIMR_Ad.	0	0.94	0.06	0.394(2449)	0.394(6343)	0.0686	0.0365
dan_daner_ANX_QIMR_Nuup.	0	0.94	0.189	0.393(1131)	0.393(1233)	0.0853	0.0649
dan_daner_ANX_SWE_Catss.	0	1.00	0.867	0.459(540)	0.459(2333)	0.012	0.0715
dan_daner_ANX_SWE_Salty.	0	1.00	0.167	0.444(687)	0.444(3445)	0.0834	0.0604
dan_daner_ANX_SWE_TwinGene.	0	1.00	0.615	0.439(1740)	0.439(6470)	0.0197	0.0392
dan_daner_ANX_UKBB.	0	1.00	4.24e−05	0.384(25453)	0.384(58113)	0.0468	0.0114
dan_daner_ANX_Utah.	0	1.04	0.736	0.406(258)	0.394(279)	0.0413	0.123
dan_daner_ANX_alspac.	0	1.00	0.965	0.381(536)	0.385(3404)	−0.00302	0.0681
dan_daner_ANX_iPSYCH.	0	0.99	0.144	0.401(5288)	0.394(14422)	0.0346	0.0237
dan_daner_ANX_musp.	0	0.99	0.499	0.380(316)	0.397(715)	−0.0671	0.0991
**dan_**	**2**	**0.99**	**2.1e−14**	**0.435(122225)**	**0.487(729960)**	**0.0383**	**0.005**

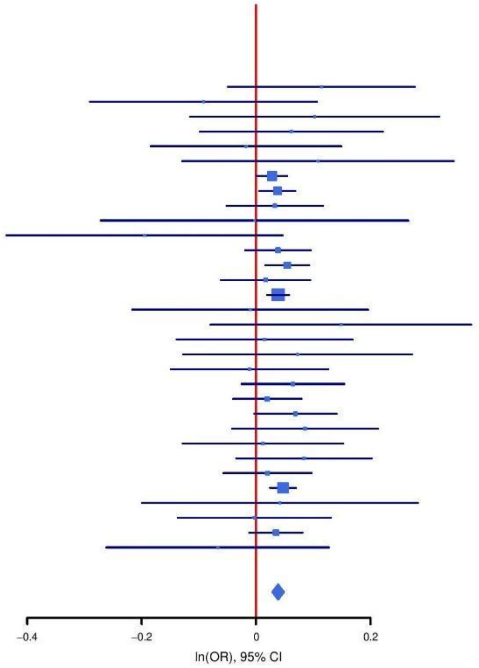

Supplementary Figure 3: Regional association plot (A) and forest plot (B) of SNP rs7110863. See full figure description on page 30.

A

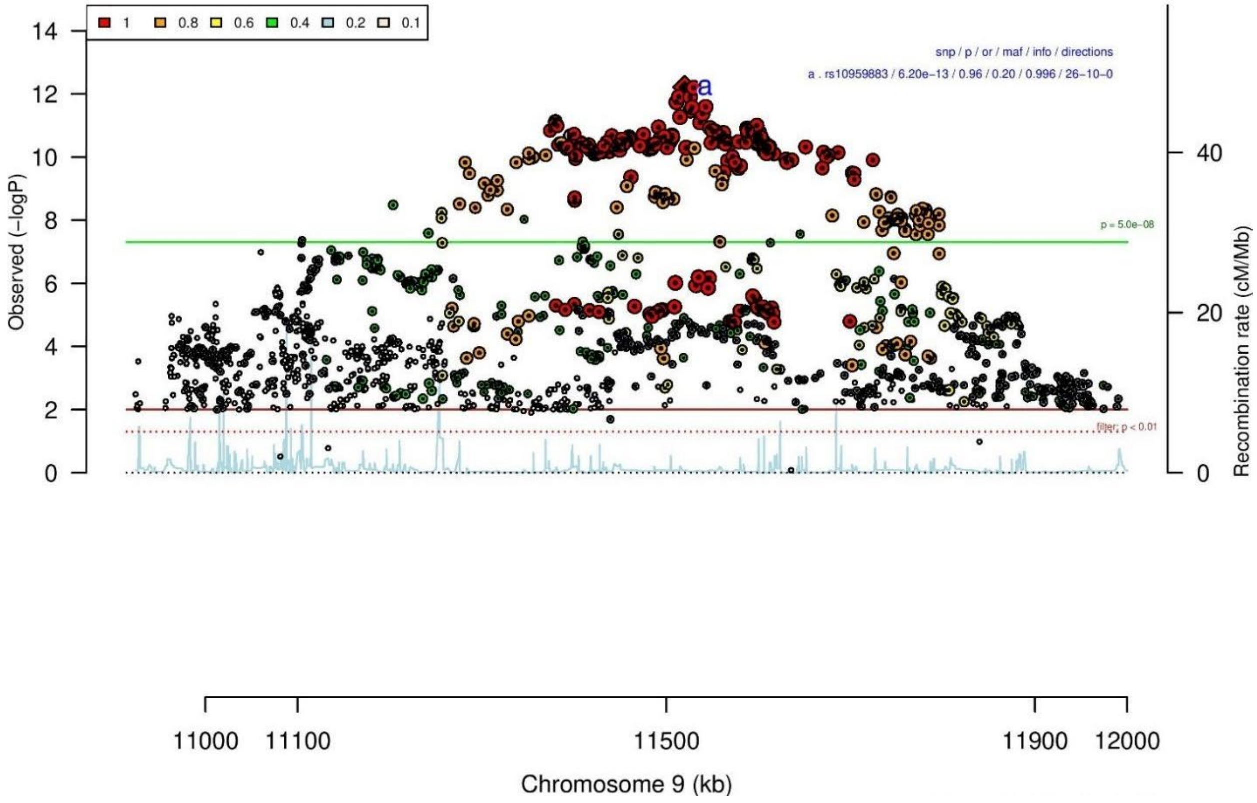

B

rs10959883		C/T		9:11519984			
−−−−−+++++++−++−+++++++−−−++++++++++				het_P:	0.262	het_I:	12.3
	ngt	info	p_value	f_ca(n)	f_co(n)	ln(OR)	STDerr
dan_daner_ANX_ANGST_gsk.	0	1.00	0.205	0.200(852)	0.200(1103)	0.104	0.0823
dan_daner_ANX_ANGST_mgs.	0	1.00	0.936	0.217(557)	0.217(779)	0.00783	0.0968
dan_daner_ANX_ANGST_ntr.	0	0.99	0.932	0.230(1521)	0.230(2970)	0.0063	0.0736
dan_daner_ANX_ANGST_rs1.	0	0.99	0.298	0.230(331)	0.230(1629)	0.119	0.115
dan_daner_ANX_ANGST_rs2.	0	0.99	0.496	0.234(209)	0.234(1023)	−0.088	0.129
dan_daner_ANX_ANGST_rs3.	0	0.99	0.168	0.239(370)	0.239(1817)	−0.133	0.0964
dan_daner_ANX_ANGST_ship.	0	1.00	0.108	0.194(545)	0.194(834)	−0.169	0.105
dan_daner_ANX_ANGST_trails.	0	0.98	0.7	0.239(278)	0.239(336)	−0.0552	0.143
dan_daner_ANX_EstBB.	0	0.99	0.317	0.185(12523)	0.185(134954)	−0.0174	0.0174
dan_daner_ANX_FinnGen.	0	1.00	0.0132	0.168(9182)	0.168(160108)	−0.0517	0.0209
dan_daner_ANX_GSC.	0	1.00	0.359	0.216(1306)	0.210(12675)	0.0474	0.0516
dan_daner_ANX_Gedi_CHDS.	0	1.00	0.837	0.206(244)	0.206(217)	0.0353	0.172
dan_daner_ANX_Gedi_GSMS.	0	1.00	0.213	0.206(208)	0.206(551)	−0.188	0.151
dan_daner_ANX_Gedi_VTSABD.	0	1.00	0.112	0.190(224)	0.190(406)	−0.0549	0.0345
dan_daner_ANX_HUNT.	0	1.00	0.494	0.228(6587)	0.232(40740)	−0.0157	0.0229
dan_daner_ANX_MGHI.	0	1.00	0.799	0.186(1823)	0.187(5151)	−0.0126	0.0498
dan_daner_ANX_MVP.	0	1.00	0.00154	0.203(28525)	0.203(163731)	−0.0379	0.012
dan_daner_ANX_MoBa.	0	1.00	0.84	0.207(715)	0.212(22168)	−0.0134	0.0663
dan_daner_ANX_PANIC_dca1.	0	0.98	0.72	0.232(248)	0.241(970)	−0.0434	0.121
dan_daner_ANX_PANIC_gmdc.	0	1.02	0.324	0.234(247)	0.206(537)	0.132	0.133
dan_daner_ANX_PANIC_gom3.	0	1.01	0.829	0.214(472)	0.212(1803)	0.0195	0.0904
dan_daner_ANX_PANIC_gwc3.	0	1.01	0.563	0.207(280)	0.200(855)	0.0746	0.129
dan_daner_ANX_PANIC_swdf.	0	1.00	0.884	0.203(561)	0.211(2591)	−0.0122	0.0838
dan_daner_ANX_PBK1.	0	1.00	0.513	0.192(1121)	0.199(11897)	−0.037	0.0565
dan_daner_ANX_QIMR_AGDS.	0	0.98	0.0122	0.209(7312)	0.209(12813)	−0.0885	0.0353
dan_daner_ANX_QIMR_Ad.	0	0.98	0.0845	0.207(2449)	0.207(6343)	−0.0768	0.0445
dan_daner_ANX_QIMR_Nuup.	0	0.98	0.593	0.209(1131)	0.209(1233)	−0.0409	0.0766
dan_daner_ANX_SWE_Catss.	0	1.00	0.318	0.195(540)	0.195(2333)	−0.0912	0.0913
dan_daner_ANX_SWE_Salty.	0	1.00	0.0327	0.204(687)	0.204(3445)	−0.165	0.0771
dan_daner_ANX_SWE_TwinGene.	0	1.00	0.312	0.212(1740)	0.212(6470)	−0.0477	0.0472
dan_daner_ANX_UKBB.	0	0.99	2.84e−10	0.213(25453)	0.213(58113)	−0.0867	0.0137
dan_daner_ANX_Utah.	0	1.02	0.215	0.192(258)	0.222(279)	−0.189	0.152
dan_daner_ANX_alspac.	0	1.00	0.617	0.229(536)	0.220(3404)	0.04	0.0799
dan_daner_ANX_bioVU.	0	1.00	0.214	0.205(7586)	0.205(50545)	−0.0289	0.0233
dan_daner_ANX_iPSYCH.	0	0.99	0.896	0.225(5288)	0.227(14422)	−0.0036	0.0277
dan_daner_ANX_musp.	0	0.97	0.85	0.207(316)	0.210(715)	−0.0227	0.12
**dan_**	**0**	**1.00**	**6.2e−13**	**0.204(122225)**	**0.196(729960)**	**−0.0423**	**0.0059**

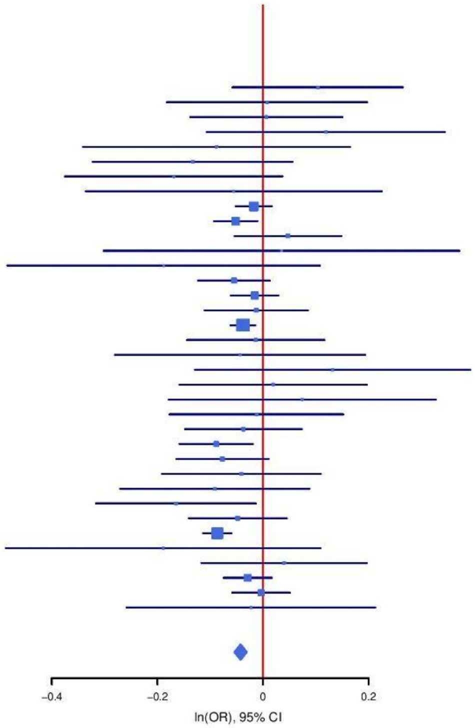

Supplementary Figure 4: Regional association plot (A) and forest plot (B) of SNP rs10959883. See full figure description on page 30.

A

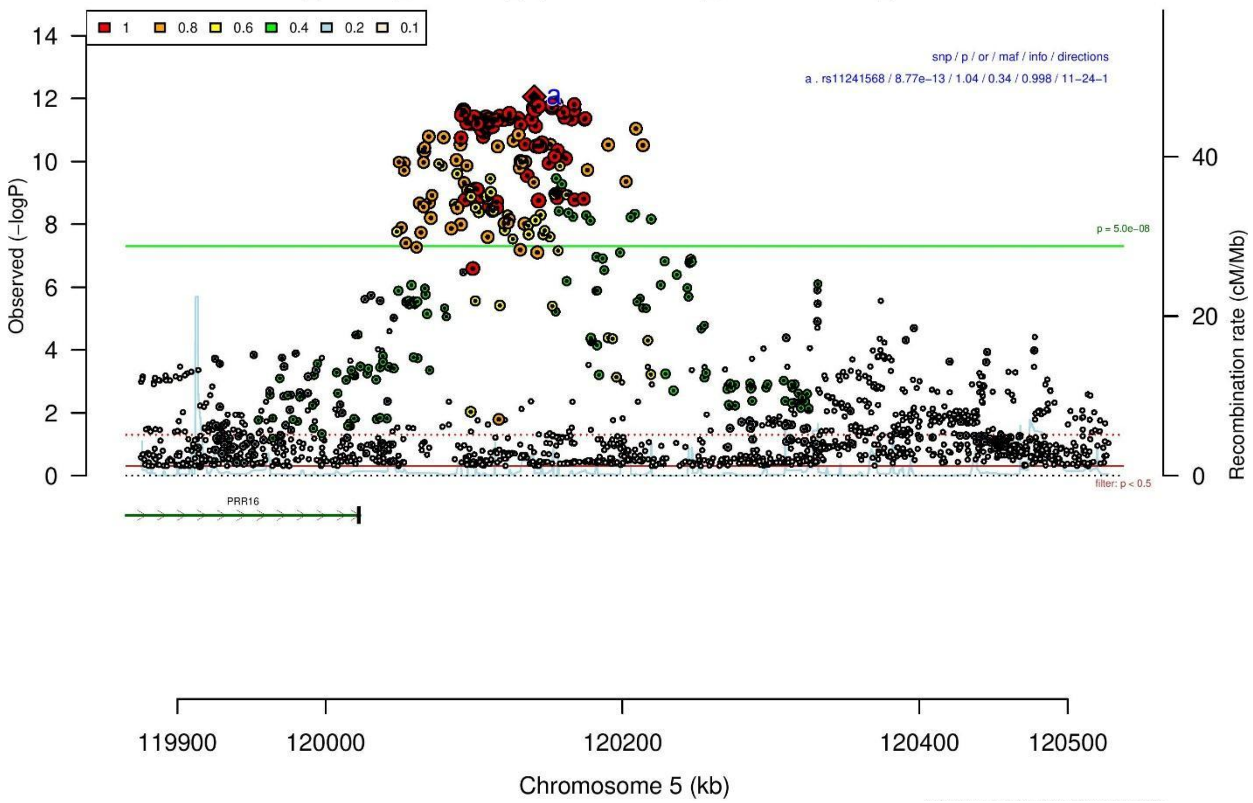

B

rs11241568		C/T		5:120140556			
+?+−++−−−−−−+−−−−−−−+−++−+−−−−−++−−−				het_P:	0.305	het_I:	9.7
	ngt	info	p_value	f_ca(n)	f_co(n)	ln(OR)	STDerr
dan_daner_ANX_ANGST_mgs.	0	1.00	0.427	0.323(557)	0.323(779)	−0.0687	0.0865
dan_daner_ANX_ANGST_ntr.	0	1.00	0.444	0.322(1521)	0.322(2970)	0.0497	0.065
dan_daner_ANX_ANGST_rs1.	0	1.00	0.628	0.329(331)	0.329(1629)	−0.051	0.106
dan_daner_ANX_ANGST_rs2.	0	1.00	0.899	0.335(209)	0.335(1023)	−0.015	0.118
dan_daner_ANX_ANGST_rs3.	0	1.00	0.558	0.329(370)	0.329(1817)	0.0498	0.0849
dan_daner_ANX_ANGST_ship.	0	1.00	0.321	0.334(545)	0.334(834)	0.0833	0.0839
dan_daner_ANX_ANGST_trails.	0	0.97	0.801	0.355(278)	0.355(336)	0.0323	0.129
dan_daner_ANX_EstBB.	0	1.00	0.00093	0.365(12523)	0.365(134954)	0.0458	0.0138
dan_daner_ANX_FinnGen.	0	1.00	0.00126	0.372(9182)	0.372(160108)	0.051	0.0158
dan_daner_ANX_GSC.	0	1.00	0.731	0.315(1306)	0.315(12675)	0.0156	0.0454
dan_daner_ANX_Gedi_CHDS.	0	1.00	0.116	0.321(244)	0.321(217)	−0.245	0.156
dan_daner_ANX_Gedi_GSMS.	0	1.00	0.25	0.329(208)	0.329(551)	0.143	0.124
dan_daner_ANX_Gedi_VTSABD.	0	1.00	0.396	0.310(224)	0.310(406)	0.0255	0.0301
dan_daner_ANX_HUNT.	0	1.00	0.16	0.330(6587)	0.323(40740)	0.0291	0.0207
dan_daner_ANX_MGHI.	0	1.00	0.428	0.303(1823)	0.295(5151)	0.0335	0.0422
dan_daner_ANX_MVP.	0	1.00	0.0178	0.313(28525)	0.313(163731)	0.0255	0.0107
dan_daner_ANX_MoBa.	0	1.00	0.75	0.338(715)	0.335(22168)	0.0182	0.0571
dan_daner_ANX_PANIC_dca1.	0	0.92	0.432	0.309(248)	0.326(970)	−0.0896	0.114
dan_daner_ANX_PANIC_gmdc.	0	0.95	0.444	0.298(247)	0.313(537)	−0.0961	0.126
dan_daner_ANX_PANIC_gom3.	0	0.96	0.268	0.337(472)	0.326(1803)	0.09	0.0812
dan_daner_ANX_PANIC_gwc3.	0	1.02	0.165	0.311(280)	0.347(855)	−0.152	0.11
dan_daner_ANX_PANIC_swdf.	0	1.00	0.653	0.351(561)	0.340(2591)	0.0321	0.0714
dan_daner_ANX_PBK1.	0	1.00	0.539	0.304(1121)	0.300(11897)	0.0296	0.0483
dan_daner_ANX_QIMR_AGDS.	0	0.99	0.151	0.312(7312)	0.312(12813)	0.0442	0.0307
dan_daner_ANX_QIMR_Ad.	0	0.99	0.135	0.320(2449)	0.320(6343)	0.0572	0.0383
dan_daner_ANX_QIMR_Nuup.	0	0.99	0.0586	0.306(1131)	0.306(1233)	0.126	0.0668
dan_daner_ANX_SWE_Catss.	0	1.00	0.062	0.346(540)	0.346(2333)	−0.137	0.0734
dan_daner_ANX_SWE_Salty.	0	1.00	0.0979	0.351(687)	0.351(3445)	−0.106	0.0641
dan_daner_ANX_SWE_TwinGene.	0	1.00	0.619	0.344(1740)	0.344(6470)	0.0202	0.0406
dan_daner_ANX_UKBB.	0	0.99	0.000136	0.319(25453)	0.319(58113)	0.0456	0.012
dan_daner_ANX_Utah.	0	1.01	0.663	0.346(258)	0.332(279)	0.056	0.129
dan_daner_ANX_alspac.	0	1.00	0.917	0.319(536)	0.322(3404)	−0.00742	0.0715
dan_daner_ANX_bioVU.	0	1.00	0.226	0.323(7586)	0.323(50545)	0.0241	0.02
dan_daner_ANX_iPSYCH.	0	0.98	0.000157	0.347(5288)	0.327(14422)	0.0925	0.0245
dan_daner_ANX_musp.	0	0.97	0.183	0.299(316)	0.329(715)	−0.141	0.106
**dan_**	**0**	**1.00**	**8.77e−13**	**0.329(122225)**	**0.339(729960)**	**0.0361**	**0.005**

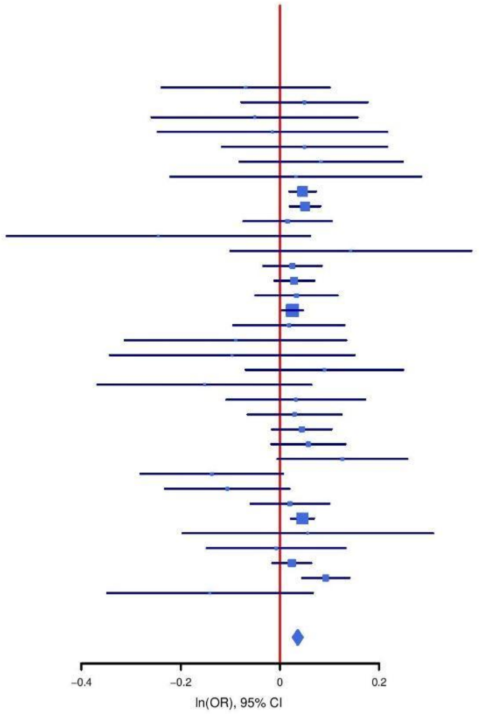

Supplementary Figure 5: Regional association plot (A) and forest plot (B) of SNP rs11241568. See full figure description on page 30.

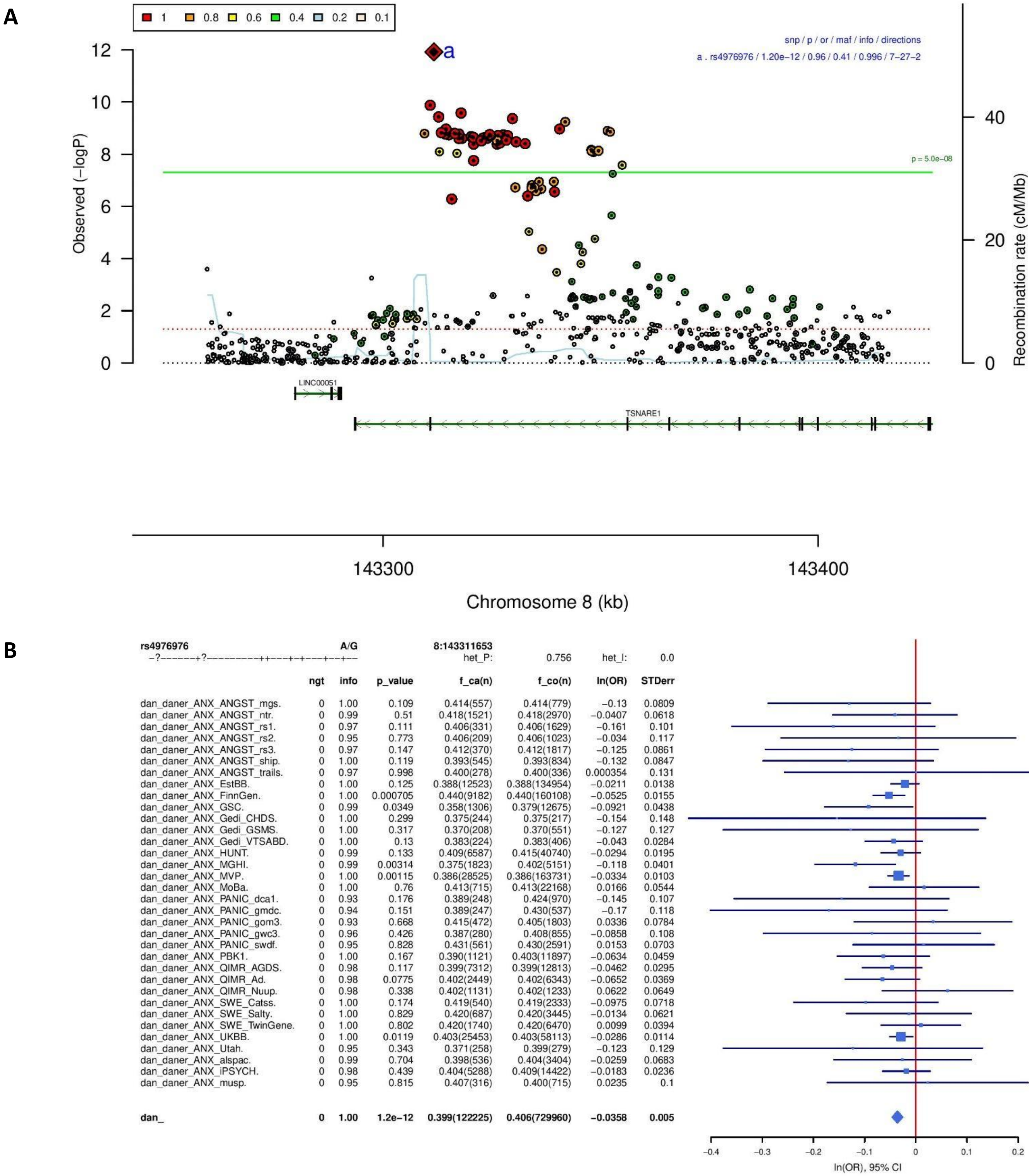

Supplementary Figure 6: Regional association plot (A) and forest plot (B) of SNP rs4976976. See full figure description on page 30.

A

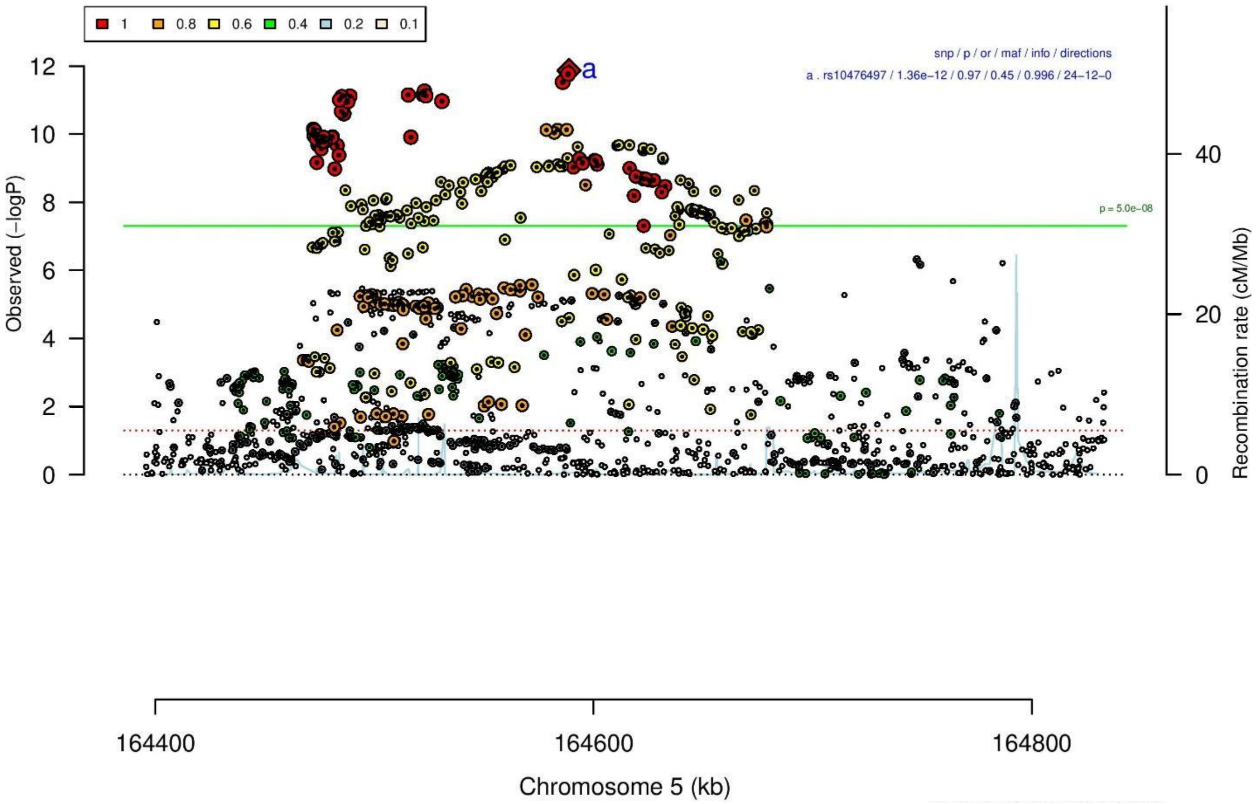

B

rs10476497		A/G		5:164588817			
++++−+−−++++−−−−+++++++−−−++++−−++++				het_P:	0.0682	het_I:	27.4
	ngt	info	p_value	f_ca(n)	f_co(n)	ln(OR)	STDerr
dan_daner_ANX_ANGST_gsk.	0	0.97	0.0432	0.494(852)	0.494(1103)	0.135	0.0666
dan_daner_ANX_ANGST_mgs.	0	1.00	0.631	0.521(557)	0.521(779)	0.0402	0.0836
dan_daner_ANX_ANGST_ntr.	0	0.98	0.122	0.562(1521)	0.562(2970)	0.0966	0.0624
dan_daner_ANX_ANGST_rs1.	0	0.99	0.337	0.565(331)	0.565(1629)	−0.095	0.099
dan_daner_ANX_ANGST_rs2.	0	0.98	0.859	0.572(209)	0.572(1023)	0.02	0.11
dan_daner_ANX_ANGST_rs3.	0	0.98	0.355	0.573(370)	0.573(1817)	−0.077	0.0833
dan_daner_ANX_ANGST_ship.	0	0.98	0.479	0.555(545)	0.555(834)	−0.06	0.0849
dan_daner_ANX_ANGST_trails.	0	0.99	0.0149	0.542(278)	0.542(336)	0.295	0.121
dan_daner_ANX_EstBB.	0	0.99	0.0708	0.536(12523)	0.536(134954)	0.0244	0.0135
dan_daner_ANX_FinnGen.	0	1.00	0.0201	0.609(9182)	0.609(160108)	0.0368	0.0158
dan_daner_ANX_GSC.	0	1.00	0.516	0.533(1306)	0.538(12675)	−0.0274	0.0422
dan_daner_ANX_Gedi_CHDS.	0	1.00	0.666	0.533(244)	0.533(217)	−0.0601	0.139
dan_daner_ANX_Gedi_GSMS.	0	1.00	0.403	0.529(208)	0.529(551)	−0.1	0.12
dan_daner_ANX_Gedi_VTSABD.	0	1.00	0.175	0.530(224)	0.530(406)	−0.0386	0.0284
dan_daner_ANX_HUNT.	0	1.00	0.00353	0.546(6587)	0.532(40740)	0.0568	0.0195
dan_daner_ANX_MGHI.	0	1.00	0.632	0.501(1823)	0.493(5151)	0.0188	0.0392
dan_daner_ANX_MVP.	0	1.00	0.00867	0.533(28525)	0.533(163731)	0.0253	0.0096
dan_daner_ANX_MoBa.	0	1.00	0.0208	0.585(715)	0.552(22168)	0.125	0.054
dan_daner_ANX_PANIC_dca1.	0	0.97	0.325	0.602(248)	0.578(970)	0.103	0.105
dan_daner_ANX_PANIC_gmdc.	0	0.99	0.378	0.546(247)	0.573(537)	−0.1	0.114
dan_daner_ANX_PANIC_gom3.	0	0.99	0.853	0.539(472)	0.546(1803)	−0.014	0.0755
dan_daner_ANX_PANIC_gwc3.	0	0.96	0.393	0.534(280)	0.542(855)	−0.091	0.106
dan_daner_ANX_PANIC_swdf.	0	0.97	0.52	0.599(561)	0.599(2591)	0.0455	0.0708
dan_daner_ANX_PBK1.	0	1.00	0.0719	0.529(1121)	0.517(11897)	0.0814	0.0452
dan_daner_ANX_QIMR_AGDS.	0	0.97	0.655	0.528(7312)	0.528(12813)	0.0129	0.0289
dan_daner_ANX_QIMR_Ad.	0	0.97	0.979	0.529(2449)	0.529(6343)	0.00094	0.0357
dan_daner_ANX_QIMR_Nuup.	0	0.97	0.53	0.539(1131)	0.539(1233)	−0.0391	0.0622
dan_daner_ANX_SWE_Catss.	0	1.00	0.941	0.412(540)	0.412(2333)	−0.00542	0.0738
dan_daner_ANX_SWE_Salty.	0	1.00	0.775	0.419(687)	0.419(3445)	0.0176	0.0617
dan_daner_ANX_SWE_TwinGene.	0	1.00	0.899	0.422(1740)	0.422(6470)	0.005	0.0393
dan_daner_ANX_UKBB.	0	0.99	1.66e−09	0.534(25453)	0.534(58113)	0.0675	0.0112
dan_daner_ANX_Utah.	0	0.91	0.565	0.557(258)	0.538(279)	0.0743	0.129
dan_daner_ANX_alspac.	0	0.99	0.0882	0.566(536)	0.537(3404)	0.115	0.0676
dan_daner_ANX_bioVU.	0	1.00	0.0593	0.528(7586)	0.528(50545)	0.0353	0.0187
dan_daner_ANX_iPSYCH.	0	0.99	0.483	0.589(5288)	0.585(14422)	0.0165	0.0235
dan_daner_ANX_musp.	0	1.01	0.7	0.520(316)	0.514(715)	0.037	0.096
**dan_**	**0**	**1.00**	**1.36e−12**	**0.540(122225)**	**0.550(729960)**	**0.0336**	**0.0047**

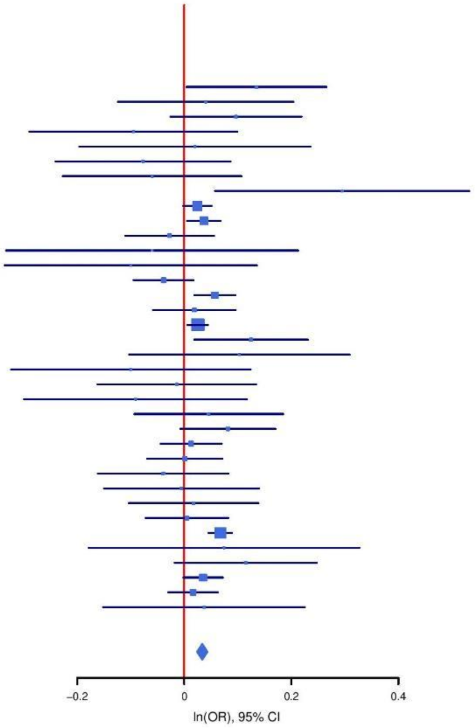

Supplementary Figure 7: Regional association plot (A) and forest plot (B) of SNP rs10476497. See full figure description on page 30.

A

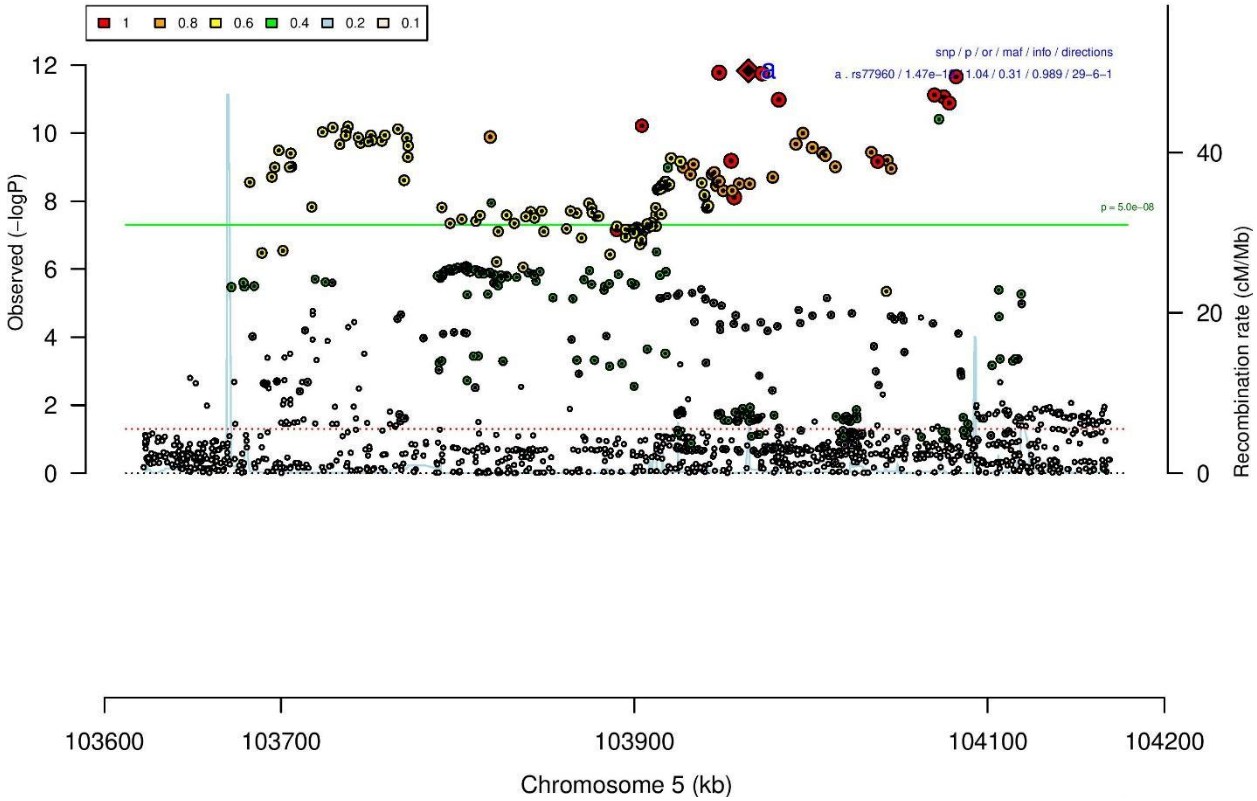

B

rs77960		A/G		5:103964585			
+-+?+++++-+++--+++++++-+-++++++++++				het_P:	0.0421	het_I:	31.3
	ngt	info	p_value	f_ca(n)	f_co(n)	ln(OR)	STDerr
dan_daner_ANX_ANGST_gsk.	0	0.83	0.642	0.310(852)	0.310(1103)	-0.0372	0.0802
dan_daner_ANX_ANGST_mgs.	0	1.00	0.733	0.338(557)	0.338(779)	0.0293	0.086
dan_daner_ANX_ANGST_rs1.	0	0.99	0.217	0.354(331)	0.354(1629)	0.125	0.101
dan_daner_ANX_ANGST_rs2.	0	0.99	0.909	0.350(209)	0.350(1023)	0.014	0.118
dan_daner_ANX_ANGST_rs3.	0	0.99	0.35	0.347(370)	0.347(1817)	0.0797	0.0853
dan_daner_ANX_ANGST_ship.	0	0.98	0.218	0.335(545)	0.335(834)	0.109	0.0887
dan_daner_ANX_ANGST_trails.	0	0.94	0.00666	0.330(278)	0.330(336)	0.362	0.133
dan_daner_ANX_EstBB.	0	0.96	0.0811	0.316(12523)	0.316(134954)	0.0257	0.0147
dan_daner_ANX_FinnGen.	0	1.00	0.0119	0.289(9182)	0.289(160108)	0.0425	0.0169
dan_daner_ANX_GSC.	0	1.01	0.944	0.319(1306)	0.321(12675)	0.00313	0.0447
dan_daner_ANX_Gedi_CHDS.	0	1.00	0.501	0.274(244)	0.274(217)	0.108	0.16
dan_daner_ANX_Gedi_GSMS.	0	1.00	0.5	0.329(208)	0.329(551)	-0.0844	0.125
dan_daner_ANX_Gedi_VTSABD.	0	1.00	0.921	0.343(224)	0.343(406)	-0.00285	0.0286
dan_daner_ANX_HUNT.	0	0.99	0.0354	0.317(6587)	0.307(40740)	0.0437	0.0207
dan_daner_ANX_MGHI.	0	0.97	0.335	0.331(1823)	0.324(5151)	0.0403	0.0417
dan_daner_ANX_MVP.	0	1.00	0.235	0.320(28525)	0.320(163731)	0.0122	0.0103
dan_daner_ANX_MoBa.	0	1.00	0.495	0.321(715)	0.312(22168)	0.0396	0.058
dan_daner_ANX_PANIC_dca1.	0	1.00	0.606	0.315(248)	0.329(970)	-0.056	0.109
dan_daner_ANX_PANIC_gmdc.	0	0.98	0.521	0.351(247)	0.334(537)	0.0759	0.118
dan_daner_ANX_PANIC_gom3.	0	0.97	0.391	0.320(472)	0.337(1803)	-0.0692	0.0807
dan_daner_ANX_PANIC_gwc3.	0	1.00	0.213	0.363(280)	0.340(855)	0.136	0.109
dan_daner_ANX_PANIC_swdf.	0	0.97	0.862	0.314(561)	0.316(2591)	0.0129	0.0742
dan_daner_ANX_PBK1.	0	1.00	0.213	0.335(1121)	0.323(11897)	0.0583	0.0469
dan_daner_ANX_QIMR_AGDS.	0	0.98	0.0148	0.332(7312)	0.332(12813)	0.0746	0.0306
dan_daner_ANX_QIMR_Ad.	0	0.98	0.00945	0.324(2449)	0.324(6343)	0.0977	0.0376
dan_daner_ANX_QIMR_Nuup.	0	0.98	0.0124	0.339(1131)	0.339(1233)	0.167	0.0669
dan_daner_ANX_SWE_Catss.	0	1.00	0.0177	0.311(540)	0.311(2333)	0.18	0.0758
dan_daner_ANX_SWE_Salty.	0	1.00	0.666	0.322(687)	0.322(3445)	0.0277	0.0642
dan_daner_ANX_SWE_TwinGene.	0	1.00	0.0378	0.320(1740)	0.320(6470)	0.0862	0.0415
dan_daner_ANX_UKBB.	0	0.99	1.56e-05	0.327(25453)	0.327(58113)	0.0513	0.0119
dan_daner_ANX_Utah.	0	0.98	0.617	0.357(258)	0.343(279)	0.0653	0.13
dan_daner_ANX_alspac.	0	0.98	0.483	0.334(536)	0.325(3404)	0.05	0.0712
dan_daner_ANX_bioVU.	0	1.00	0.617	0.326(7586)	0.326(50545)	-0.00996	0.0199
dan_daner_ANX_iPSYCH.	0	0.97	0.000245	0.342(5288)	0.322(14422)	0.0906	0.0247
dan_daner_ANX_musp.	0	1.06	0.0557	0.359(316)	0.313(715)	0.189	0.0988
**dan_**	**0**	**0.99**	**1.47e-12**	**0.322(122225)**	**0.313(729960)**	**0.0359**	**0.0051**

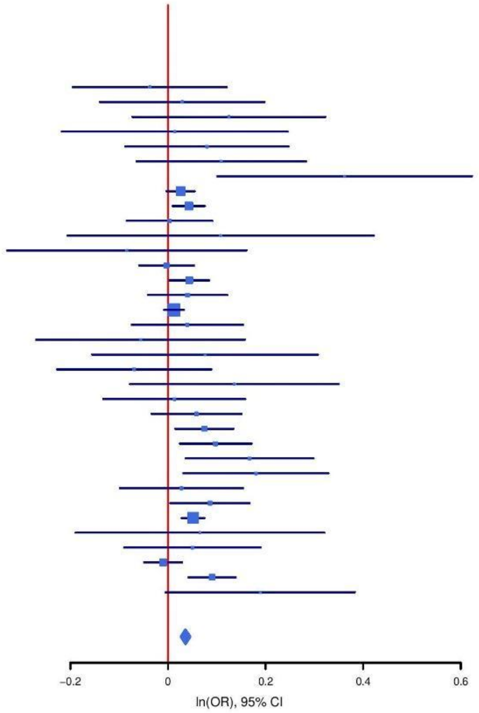

Supplementary Figure 8: Regional association plot (A) and forest plot (B) of SNP rs77960. See full figure description on page 30.

A

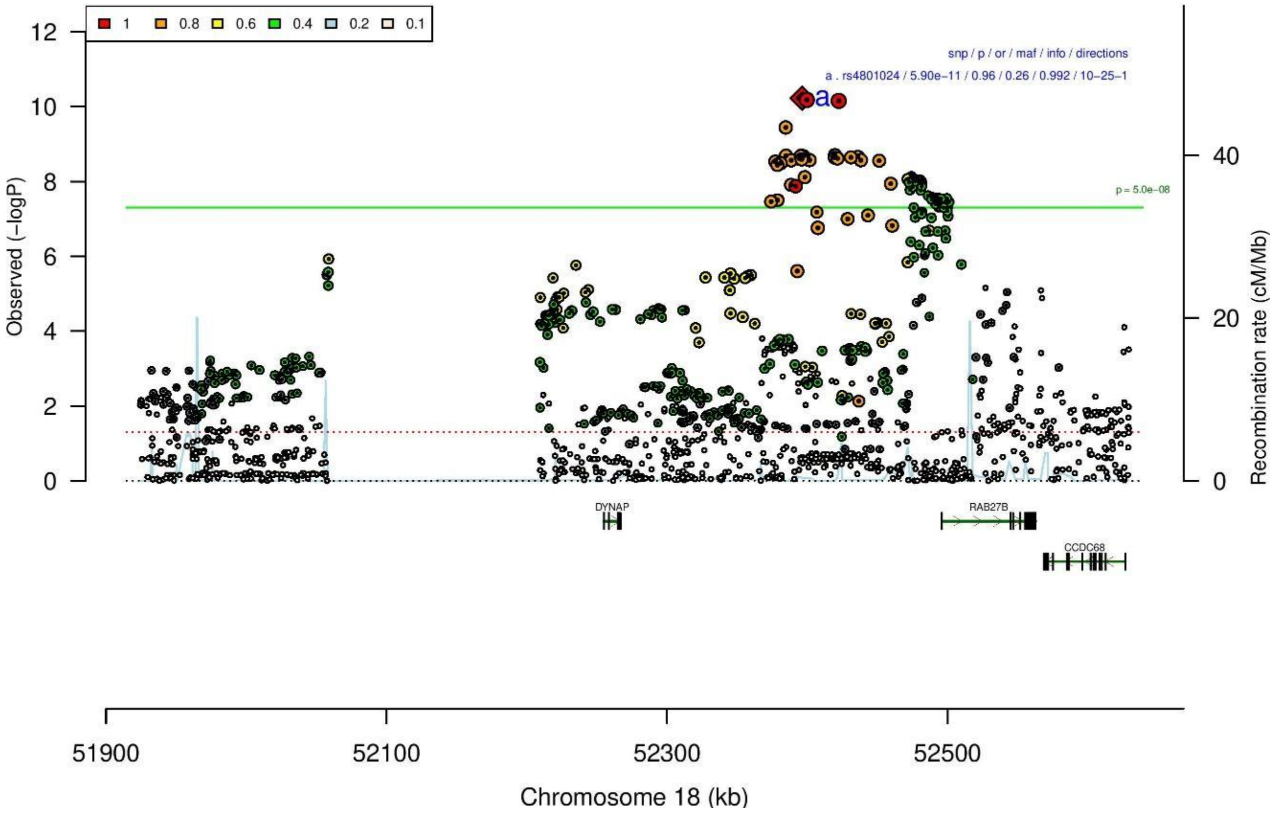

B

rs4801024		G/T		18:52396321			
+---+-++-?-----------+-+-+++--------+				het_P:	0.755	het_I:	0.0
	ngt	info	p_value	f_ca(n)	f_co(n)	ln(OR)	STDerr
dan_daner_ANX_ANGST_gsk.	0	0.99	0.249	0.784(852)	0.784(1103)	0.0936	0.0811
dan_daner_ANX_ANGST_mgs.	0	1.00	0.0959	0.787(557)	0.787(779)	0.169	0.101
dan_daner_ANX_ANGST_ntr.	0	1.00	0.0784	0.791(1521)	0.791(2970)	0.134	0.0761
dan_daner_ANX_ANGST_rs1.	0	1.00	0.474	0.792(331)	0.792(1629)	−0.085	0.118
dan_daner_ANX_ANGST_rs2.	0	0.99	0.205	0.793(209)	0.793(1023)	0.176	0.139
dan_daner_ANX_ANGST_rs3.	0	0.99	0.953	0.795(370)	0.795(1817)	−0.00606	0.102
dan_daner_ANX_ANGST_ship.	0	1.00	0.721	0.763(545)	0.763(834)	−0.0336	0.0941
dan_daner_ANX_ANGST_trails.	0	0.95	0.107	0.799(278)	0.799(336)	0.237	0.147
dan_daner_ANX_EstBB.	0	0.99	0.0411	0.714(12523)	0.714(134954)	0.0305	0.0149
dan_daner_ANX_FinnGen.	0	1.00	0.129	0.723(9182)	0.723(160108)	0.0262	0.0173
dan_daner_ANX_GSC.	0	0.98	0.391	0.775(1306)	0.766(12675)	0.0431	0.0503
dan_daner_ANX_Gedi_CHDS.	0	1.00	0.735	0.770(244)	0.770(217)	0.0543	0.161
dan_daner_ANX_Gedi_GSMS.	0	1.00	0.149	0.769(208)	0.769(551)	0.208	0.144
dan_daner_ANX_Gedi_VTSABD.	0	1.00	0.105	0.771(224)	0.771(406)	0.0544	0.0335
dan_daner_ANX_HUNT.	0	1.00	0.466	0.758(6587)	0.754(40740)	0.0164	0.0225
dan_daner_ANX_MGHI.	0	0.99	0.786	0.783(1823)	0.782(5151)	0.0129	0.0473
dan_daner_ANX_MVP.	0	1.00	0.0011	0.770(28525)	0.770(163731)	0.0371	0.0114
dan_daner_ANX_MoBa.	0	1.00	0.0973	0.771(715)	0.750(22168)	0.103	0.0623
dan_daner_ANX_PANIC_dca1.	0	0.97	0.735	0.759(248)	0.763(970)	−0.0409	0.121
dan_daner_ANX_PANIC_gmdc.	0	0.98	0.208	0.783(247)	0.750(537)	0.17	0.135
dan_daner_ANX_PANIC_gom3.	0	0.98	0.641	0.773(472)	0.779(1803)	−0.0419	0.0901
dan_daner_ANX_PANIC_gwc3.	0	1.02	0.478	0.750(280)	0.769(855)	−0.0856	0.12
dan_daner_ANX_PANIC_swdf.	0	0.95	0.618	0.753(561)	0.756(2591)	−0.0402	0.0804
dan_daner_ANX_PBK1.	0	1.00	0.00737	0.803(1121)	0.777(11897)	0.15	0.0559
dan_daner_ANX_QIMR_AGDS.	0	0.90	0.222	0.757(7312)	0.757(12813)	0.0427	0.035
dan_daner_ANX_QIMR_Ad.	0	0.90	0.18	0.757(2449)	0.757(6343)	0.0567	0.0423
dan_daner_ANX_QIMR_Nuup.	0	0.90	0.804	0.737(1131)	0.737(1233)	0.0182	0.0733
dan_daner_ANX_SWE_Catss.	0	1.00	0.649	0.245(540)	0.245(2333)	0.0376	0.0826
dan_daner_ANX_SWE_Salty.	0	1.00	0.934	0.249(687)	0.249(3445)	0.0058	0.0701
dan_daner_ANX_SWE_TwinGene.	0	1.00	0.0888	0.246(1740)	0.246(6470)	0.0777	0.0456
dan_daner_ANX_UKBB.	0	1.00	0.00056	0.776(25453)	0.776(58113)	0.0464	0.0134
dan_daner_ANX_Utah.	0	1.00	0.519	0.762(258)	0.780(279)	−0.0939	0.146
dan_daner_ANX_alspac.	0	1.00	0.175	0.758(536)	0.777(3404)	−0.106	0.0776
dan_daner_ANX_iPSYCH.	0	0.99	0.164	0.772(5288)	0.765(14422)	0.0383	0.0275
dan_daner_ANX_musp.	0	0.93	0.535	0.761(316)	0.773(715)	−0.0726	0.117
**dan_**	**0**	**0.99**	**5.9e−11**	**0.747(122225)**	**0.737(729960)**	**0.0373**	**0.0057**

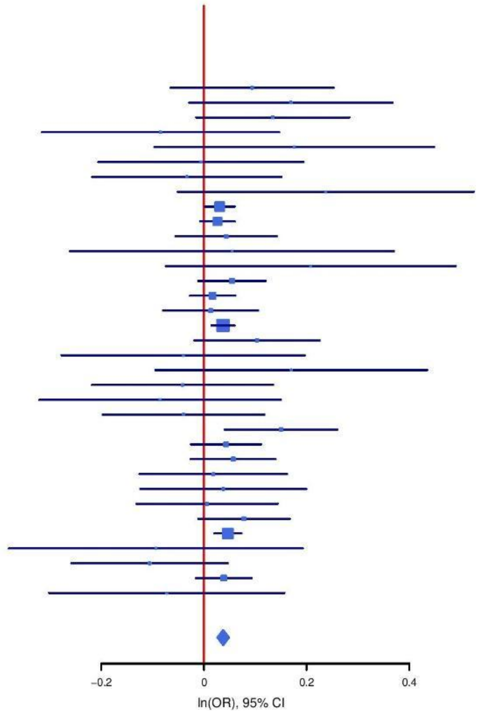

Supplementary Figure 9: Regional association plot (A) and forest plot (B) of SNP rs4801024. See full figure description on page 30.

A

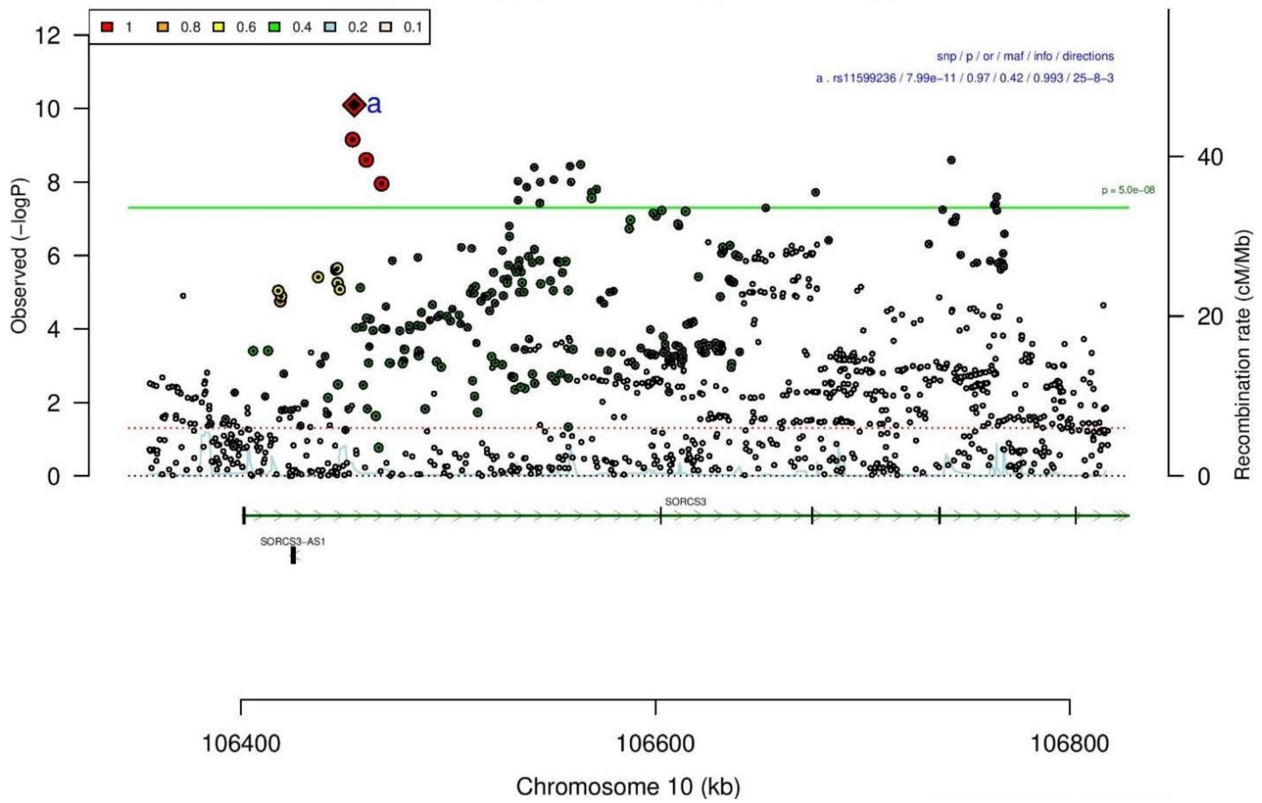

B

rs11599236		C/T		10:106454672			
−?+−+−+++?++−++++++++++−+−−?+++−++++				het_P:	0.773	het_I:	0.0

	ngt	info	p_value	f_ca(n)	f_co(n)	ln(OR)	STDerr
dan_daner_ANX_ANGST_mgs.	0	1.00	0.562	0.407(557)	0.407(779)	−0.0481	0.0828
dan_daner_ANX_ANGST_ntr.	0	1.00	0.546	0.405(1521)	0.405(2970)	0.0376	0.0622
dan_daner_ANX_ANGST_rs1.	0	0.93	0.17	0.407(331)	0.407(1629)	−0.142	0.104
dan_daner_ANX_ANGST_rs2.	0	0.94	0.116	0.407(209)	0.407(1023)	0.179	0.114
dan_daner_ANX_ANGST_rs3.	0	0.93	0.0754	0.399(370)	0.399(1817)	−0.154	0.0867
dan_daner_ANX_ANGST_ship.	0	1.00	0.976	0.417(545)	0.417(834)	−0.00248	0.0816
dan_daner_ANX_ANGST_trails.	0	1.00	0.0792	0.379(278)	0.379(336)	−0.215	0.122
dan_daner_ANX_EstBB.	0	0.99	0.0223	0.380(12523)	0.380(134954)	−0.0317	0.0139
dan_daner_ANX_FinnGen.	0	0.99	0.0597	0.480(9182)	0.480(160108)	−0.029	0.0154
dan_daner_ANX_GSC.	1	1.00	0.166	0.382(1306)	0.396(12675)	−0.0595	0.0429
dan_daner_ANX_Gedi_CHDS.	0	1.00	0.409	0.390(244)	0.390(217)	0.119	0.144
dan_daner_ANX_Gedi_GSMS.	0	1.00	0.854	0.429(208)	0.429(551)	−0.0225	0.122
dan_daner_ANX_Gedi_VTSABD.	0	1.00	0.0841	0.406(224)	0.406(406)	−0.0492	0.0285
dan_daner_ANX_HUNT.	0	0.99	0.0397	0.395(6587)	0.405(40740)	−0.0406	0.0198
dan_daner_ANX_MGHI.	0	1.00	0.333	0.444(1823)	0.455(5151)	−0.0382	0.0394
dan_daner_ANX_MVP.	0	1.00	0.12	0.414(28525)	0.414(163731)	−0.0154	0.0099
dan_daner_ANX_MoBa.	0	1.00	0.111	0.394(715)	0.415(22168)	−0.0874	0.0549
dan_daner_ANX_PANIC_dca1.	0	0.93	0.621	0.391(248)	0.406(970)	−0.0534	0.108
dan_daner_ANX_PANIC_gmdc.	0	0.92	0.831	0.442(247)	0.430(537)	0.0252	0.118
dan_daner_ANX_PANIC_gom3.	0	0.97	0.816	0.413(472)	0.416(1803)	−0.018	0.0774
dan_daner_ANX_PANIC_gwc3.	0	0.96	0.675	0.399(280)	0.411(855)	0.0454	0.108
dan_daner_ANX_PANIC_swdf.	0	0.96	0.845	0.419(561)	0.407(2591)	0.0138	0.0705
dan_daner_ANX_QIMR_AGDS.	0	0.98	0.0744	0.409(7312)	0.409(12813)	−0.0523	0.0293
dan_daner_ANX_QIMR_Ad.	0	0.98	0.932	0.406(2449)	0.406(6343)	−0.00309	0.0364
dan_daner_ANX_QIMR_Nuup.	0	0.98	0.168	0.413(1131)	0.413(1233)	−0.0881	0.064
dan_daner_ANX_SWE_Catss.	0	1.00	0.327	0.414(540)	0.414(2333)	0.0702	0.0716
dan_daner_ANX_SWE_Salty.	0	1.00	0.276	0.408(687)	0.408(3445)	−0.0677	0.0621
dan_daner_ANX_SWE_TwinGene.	0	1.00	0.0818	0.415(1740)	0.415(6470)	−0.0684	0.0393
dan_daner_ANX_UKBB.	0	0.98	7.89e−05	0.414(25453)	0.414(58113)	−0.0452	0.0114
dan_daner_ANX_Utah.	0	1.00	0.48	0.358(258)	0.377(279)	−0.0904	0.128
dan_daner_ANX_alspac.	0	0.99	0.641	0.408(536)	0.403(3404)	0.0323	0.0692
dan_daner_ANX_iPSYCH.	0	0.99	0.0234	0.394(5288)	0.408(14422)	−0.0537	0.0237
dan_daner_ANX_musp.	0	1.01	0.967	0.390(316)	0.392(715)	−0.00409	0.0976
**dan_**	**1**	**0.99**	**7.99e−11**	**0.412(122225)**	**0.422(729960)**	**−0.0327**	**0.005**

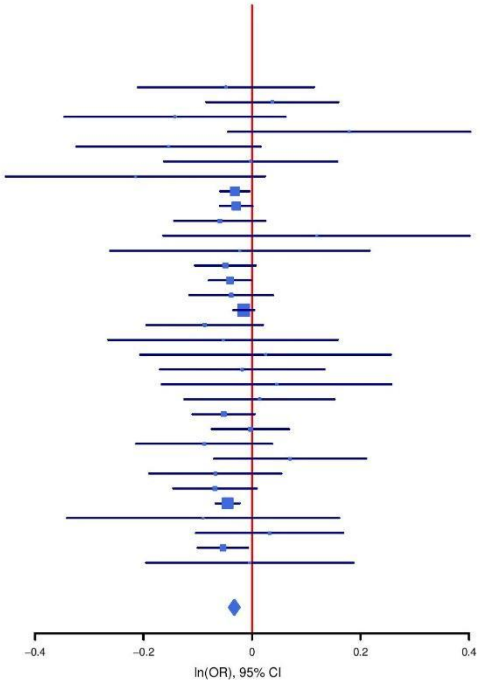

Supplementary Figure 10: Regional association plot (A) and forest plot (B) of SNP rs11599236. See full figure description on page 30.

A

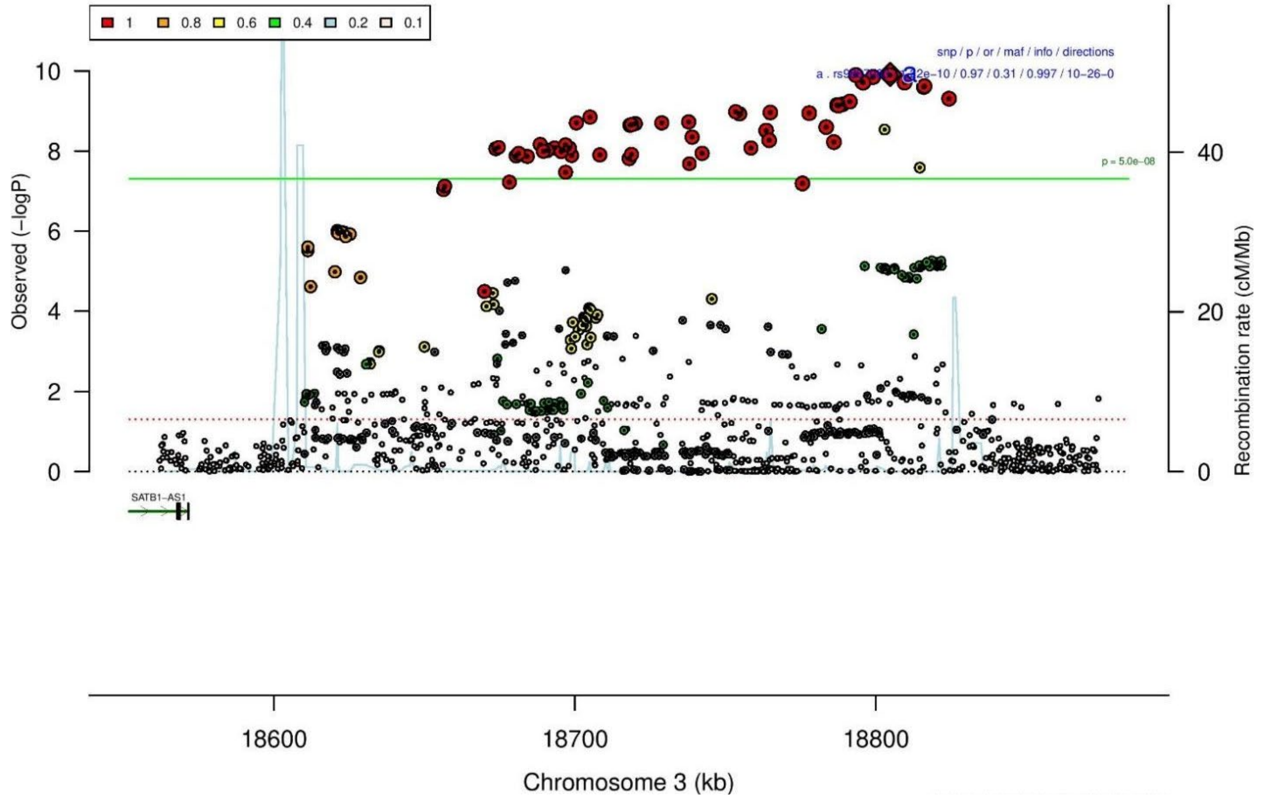

B

rs9867083		C/T		3:18804734			
---+-+-+----+-+-----+-+++----------+				het_P:	0.253	het_I:	12.8
	**ngt**	**info**	**p_value**	**f_ca(n)**	**f_co(n)**	**ln(OR)**	**STDerr**
dan_daner_ANX_ANGST_gsk.	0	0.98	0.275	0.694(852)	0.694(1103)	0.0799	0.0732
dan_daner_ANX_ANGST_mgs.	0	1.00	0.557	0.706(557)	0.706(779)	0.0523	0.089
dan_daner_ANX_ANGST_ntr.	0	0.99	0.601	0.717(1521)	0.717(2970)	−0.0353	0.0675
dan_daner_ANX_ANGST_rs1.	0	0.99	0.265	0.720(331)	0.720(1629)	0.128	0.115
dan_daner_ANX_ANGST_rs2.	0	1.00	0.903	0.718(209)	0.718(1023)	−0.015	0.12
dan_daner_ANX_ANGST_rs3.	0	0.99	0.0222	0.716(370)	0.716(1817)	0.215	0.094
dan_daner_ANX_ANGST_ship.	0	0.99	0.42	0.722(545)	0.722(834)	−0.073	0.0905
dan_daner_ANX_ANGST_trails.	0	0.99	0.499	0.707(278)	0.707(336)	0.0929	0.137
dan_daner_ANX_EstBB.	0	1.00	0.0306	0.718(12523)	0.718(134954)	0.0324	0.015
dan_daner_ANX_FinnGen.	0	0.99	0.0107	0.654(9182)	0.654(160108)	0.0415	0.0163
dan_daner_ANX_GSC.	0	1.00	0.582	0.722(1306)	0.720(12675)	0.0257	0.0466
dan_daner_ANX_Gedi_CHDS.	0	1.00	0.902	0.728(244)	0.728(217)	−0.0197	0.16
dan_daner_ANX_Gedi_GSMS.	0	1.00	0.375	0.713(208)	0.713(551)	0.118	0.133
dan_daner_ANX_Gedi_VTSABD.	0	1.00	0.53	0.737(224)	0.737(406)	−0.0197	0.0313
dan_daner_ANX_HUNT.	0	1.00	0.464	0.739(6587)	0.736(40740)	0.016	0.0219
dan_daner_ANX_MGHI.	0	1.00	0.636	0.705(1823)	0.698(5151)	0.0201	0.0425
dan_daner_ANX_MVP.	0	1.00	7.99e−06	0.709(28525)	0.709(163731)	0.0479	0.0107
dan_daner_ANX_MoBa.	0	1.00	0.0188	0.753(715)	0.725(22168)	0.142	0.0604
dan_daner_ANX_PANIC_dca1.	0	0.98	0.902	0.701(248)	0.706(970)	−0.0138	0.112
dan_daner_ANX_PANIC_gmdc.	0	0.97	0.637	0.673(247)	0.678(537)	−0.0578	0.122
dan_daner_ANX_PANIC_gom3.	0	0.99	0.571	0.705(472)	0.714(1803)	−0.0466	0.0822
dan_daner_ANX_PANIC_gwc3.	0	1.05	0.388	0.716(280)	0.682(855)	0.0949	0.11
dan_daner_ANX_PANIC_swdf.	0	0.99	0.334	0.729(561)	0.717(2591)	0.0741	0.0767
dan_daner_ANX_PBK1.	0	1.00	0.576	0.710(1121)	0.707(11897)	0.0274	0.049
dan_daner_ANX_QIMR_AGDS.	0	0.97	0.893	0.702(7312)	0.702(12813)	0.00427	0.0317
dan_daner_ANX_QIMR_Ad.	0	0.97	0.0248	0.695(2449)	0.695(6343)	0.0893	0.0398
dan_daner_ANX_QIMR_Nuup.	0	0.97	0.321	0.704(1131)	0.704(1233)	0.0691	0.0697
dan_daner_ANX_SWE_Catss.	0	1.00	0.111	0.274(540)	0.274(2333)	0.127	0.0799
dan_daner_ANX_SWE_Salty.	0	1.00	0.00736	0.268(687)	0.268(3445)	0.188	0.07
dan_daner_ANX_SWE_TwinGene.	0	1.00	0.863	0.275(1740)	0.275(6470)	0.00752	0.0436
dan_daner_ANX_UKBB.	0	1.00	0.0788	0.713(25453)	0.713(58113)	0.0217	0.0123
dan_daner_ANX_Utah.	0	1.05	0.0547	0.680(258)	0.736(279)	−0.255	0.133
dan_daner_ANX_alspac.	0	0.99	0.153	0.736(536)	0.717(3404)	0.107	0.0755
dan_daner_ANX_bioVU.	0	1.00	0.0388	0.715(7586)	0.715(50545)	0.0427	0.0207
dan_daner_ANX_iPSYCH.	0	0.99	0.981	0.722(5288)	0.721(14422)	0.0006	0.0259
dan_daner_ANX_musp.	0	1.02	0.185	0.695(316)	0.722(715)	−0.138	0.104
**dan_**	**0**	**1.00**	**1.22e−10**	**0.698(122225)**	**0.694(729960)**	**0.0335**	**0.0052**

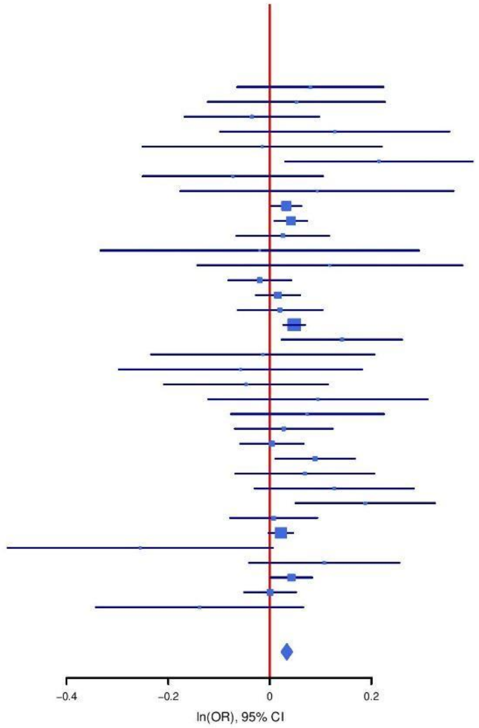

Supplementary Figure 11: Regional association plot (A) and forest plot (B) of SNP rs9867083. See full figure description on page 30.

A

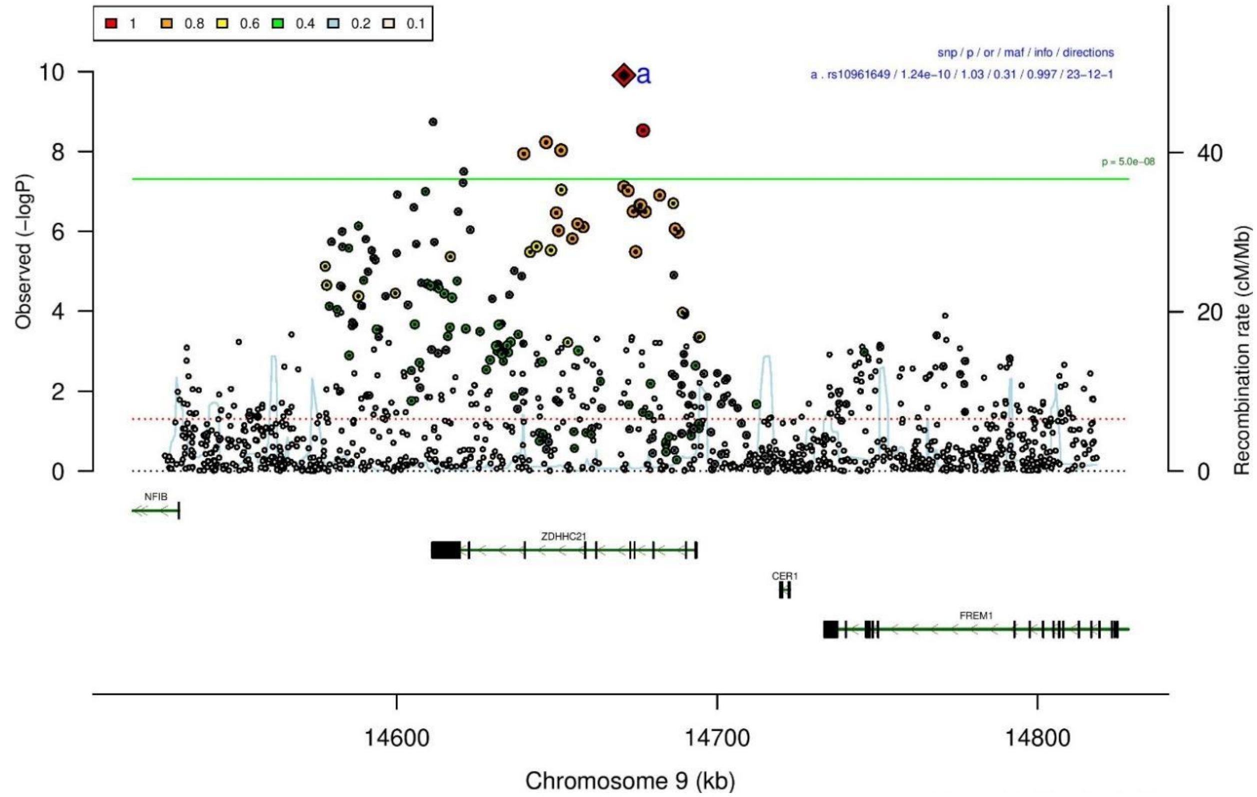

B

rs10961649 **T/C** **9:14670949**

+−−++++−−+++−−+−++++−+−+−++?−++−++++ het_P: 0.387 het_I: 4.8

	ngt	info	p_value	f_ca(n)	f_co(n)	ln(OR)	STDerr
dan_daner_ANX_ANGST_gsk.	0	0.89	0.772	0.339(852)	0.339(1103)	−0.022	0.0758
dan_daner_ANX_ANGST_mgs.	0	1.00	0.545	0.328(557)	0.328(779)	−0.0514	0.0848
dan_daner_ANX_ANGST_ntr.	0	1.00	0.94	0.311(1521)	0.311(2970)	0.005	0.0668
dan_daner_ANX_ANGST_rs1.	0	1.00	0.348	0.321(331)	0.321(1629)	0.1	0.106
dan_daner_ANX_ANGST_rs2.	0	1.00	0.808	0.325(209)	0.325(1023)	0.028	0.115
dan_daner_ANX_ANGST_rs3.	0	1.00	0.19	0.326(370)	0.326(1817)	0.112	0.0854
dan_daner_ANX_ANGST_ship.	0	1.00	0.906	0.322(545)	0.322(834)	−0.0101	0.086
dan_daner_ANX_ANGST_trails.	0	0.96	0.783	0.301(278)	0.301(336)	−0.0367	0.133
dan_daner_ANX_EstBB.	0	1.00	0.0165	0.290(12523)	0.290(134954)	0.0353	0.0147
dan_daner_ANX_FinnGen.	0	1.00	0.0344	0.286(9182)	0.286(160108)	0.0358	0.0169
dan_daner_ANX_GSC.	1	1.00	0.806	0.312(1306)	0.315(12675)	−0.0111	0.0451
dan_daner_ANX_Gedi_CHDS.	0	1.00	0.0971	0.342(244)	0.342(217)	−0.25	0.151
dan_daner_ANX_Gedi_GSMS.	0	1.00	0.775	0.350(208)	0.350(551)	−0.0357	0.125
dan_daner_ANX_Gedi_VTSABD.	0	1.00	0.0138	0.311(224)	0.311(406)	0.0692	0.028
dan_daner_ANX_HUNT.	0	0.99	0.409	0.312(6587)	0.307(40740)	0.0173	0.021
dan_daner_ANX_MGHI.	0	0.98	0.125	0.342(1823)	0.328(5151)	0.0634	0.0413
dan_daner_ANX_MVP.	0	1.00	0.00348	0.320(28525)	0.320(163731)	0.0304	0.0104
dan_daner_ANX_MoBa.	0	1.00	0.0669	0.328(715)	0.304(22168)	0.107	0.0585
dan_daner_ANX_PANIC_dca1.	0	0.98	0.867	0.291(248)	0.298(970)	−0.0189	0.113
dan_daner_ANX_PANIC_gmdc.	0	1.02	0.149	0.366(247)	0.321(537)	0.169	0.117
dan_daner_ANX_PANIC_gom3.	0	0.96	0.938	0.316(472)	0.316(1803)	−0.00632	0.0817
dan_daner_ANX_PANIC_gwc3.	1	0.98	0.155	0.343(280)	0.319(855)	0.16	0.112
dan_daner_ANX_PANIC_swdf.	0	0.98	0.271	0.316(561)	0.296(2591)	0.0818	0.0743
dan_daner_ANX_QIMR_AGDS.	0	1.00	0.0317	0.322(7312)	0.322(12813)	0.066	0.0307
dan_daner_ANX_QIMR_Ad.	0	1.00	0.0196	0.321(2449)	0.321(6343)	−0.0903	0.0387
dan_daner_ANX_QIMR_Nuup.	0	1.00	0.402	0.300(1131)	0.300(1233)	0.0568	0.0678
dan_daner_ANX_SWE_Catss.	0	1.00	0.396	0.283(540)	0.283(2333)	−0.0684	0.0806
dan_daner_ANX_SWE_Salty.	0	1.00	0.0525	0.301(687)	0.301(3445)	0.128	0.0659
dan_daner_ANX_SWE_TwinGene.	0	1.00	0.227	0.296(1740)	0.296(6470)	0.0505	0.0418
dan_daner_ANX_UKBB.	0	0.99	0.0459	0.322(25453)	0.322(58113)	0.0239	0.0119
dan_daner_ANX_Utah.	0	0.91	0.291	0.339(258)	0.310(279)	0.146	0.138
dan_daner_ANX_alspac.	0	1.00	0.937	0.333(536)	0.330(3404)	0.00551	0.0697
dan_daner_ANX_bioVU.	0	1.00	0.000914	0.330(7586)	0.330(50545)	0.0656	0.0198
dan_daner_ANX_iPSYCH.	0	0.97	0.207	0.315(5288)	0.309(14422)	0.0318	0.0252
dan_daner_ANX_musp.	0	0.98	0.993	0.316(316)	0.315(715)	−0.000999	0.104
**dan_**	**2**	**1.00**	**1.24e−10**	**0.315(122225)**	**0.306(729960)**	**0.0328**	**0.0051**

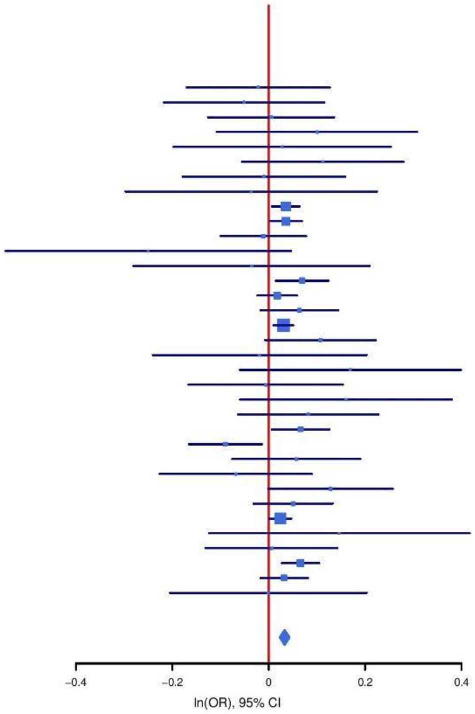

Supplementary Figure 12: Regional association plot (A) and forest plot (B) of SNP rs10961649. See full figure description on page 30.

A

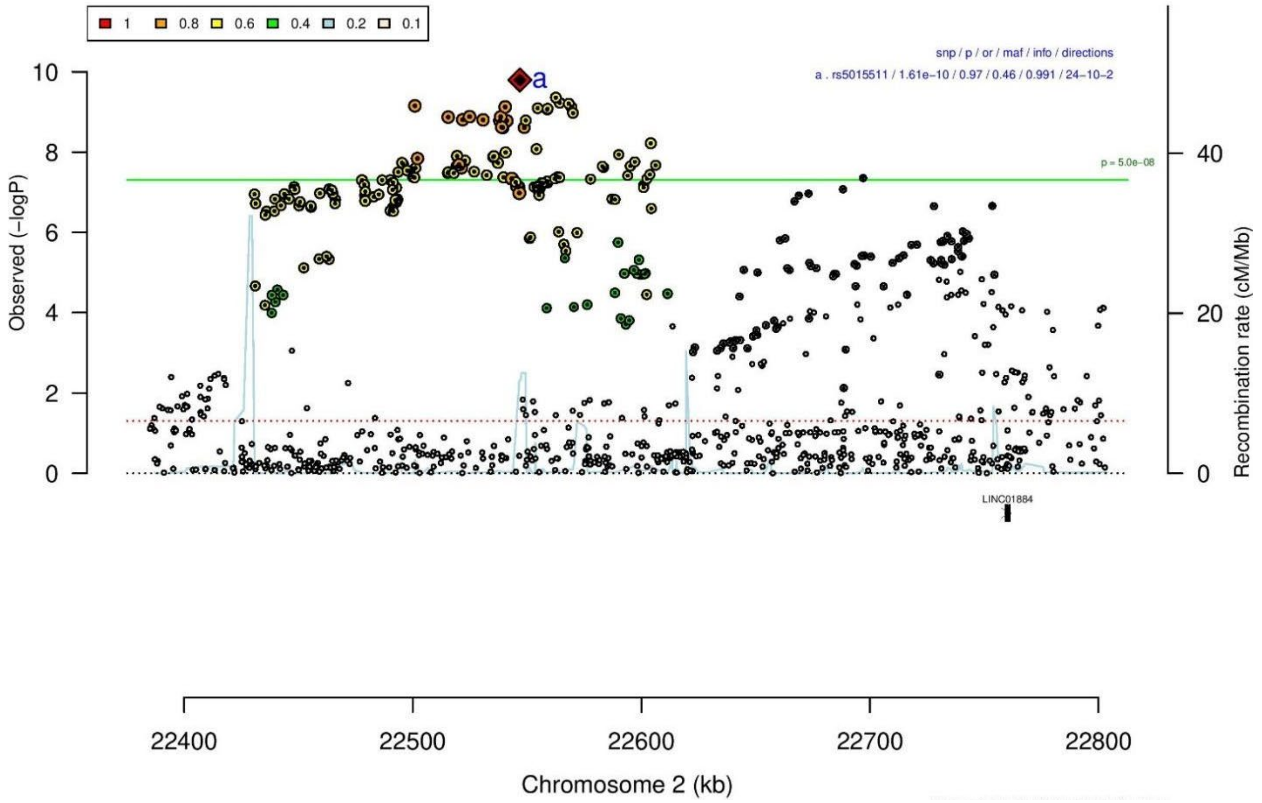

B

rs5015511		A/G		2:22546852			
−+++−+−+−?++++++++++++−−−++?++−−−+++				het_P:	0.0144	het_I:	38.0
	ngt	info	p_value	f_ca(n)	f_co(n)	ln(OR)	STDerr
dan_daner_ANX_ANGST_gsk.	0	0.87	0.512	0.499(852)	0.499(1103)	0.048	0.0732
dan_daner_ANX_ANGST_mgs.	0	1.00	0.0125	0.495(557)	0.495(779)	0.21	0.084
dan_daner_ANX_ANGST_ntr.	0	1.00	0.137	0.508(1521)	0.508(2970)	0.0904	0.0607
dan_daner_ANX_ANGST_rs1.	0	0.93	0.605	0.508(331)	0.508(1629)	−0.053	0.102
dan_daner_ANX_ANGST_rs2.	0	0.92	0.0833	0.519(209)	0.519(1023)	0.201	0.116
dan_daner_ANX_ANGST_rs3.	0	0.91	0.994	0.512(370)	0.512(1817)	−0.000627	0.0858
dan_daner_ANX_ANGST_ship.	0	0.93	0.951	0.520(545)	0.520(834)	0.00518	0.0847
dan_daner_ANX_ANGST_trails.	0	0.91	0.695	0.525(278)	0.525(336)	−0.0497	0.127
dan_daner_ANX_EstBB.	0	0.99	0.028	0.538(12523)	0.538(134954)	0.0296	0.0135
dan_daner_ANX_FinnGen.	0	0.99	0.02	0.627(9182)	0.627(160108)	0.0374	0.0161
dan_daner_ANX_GSC.	1	0.98	0.0956	0.533(1306)	0.514(12675)	0.0707	0.0424
dan_daner_ANX_Gedi_CHDS.	0	1.00	0.903	0.539(244)	0.539(217)	0.0167	0.136
dan_daner_ANX_Gedi_GSMS.	0	1.00	0.26	0.498(208)	0.498(551)	0.133	0.118
dan_daner_ANX_Gedi_VTSABD.	0	1.00	0.559	0.516(224)	0.516(406)	0.016	0.0273
dan_daner_ANX_HUNT.	0	0.99	0.0158	0.491(6587)	0.475(40740)	0.0465	0.0193
dan_daner_ANX_MGHI.	1	1.00	0.138	0.517(1823)	0.500(5151)	0.0575	0.0388
dan_daner_ANX_MVP.	0	1.00	0.475	0.512(28525)	0.512(163731)	0.0069	0.0096
dan_daner_ANX_MoBa.	0	1.00	0.416	0.497(715)	0.485(22168)	0.0436	0.0536
dan_daner_ANX_PANIC_dca1.	0	0.93	0.102	0.479(248)	0.515(970)	−0.172	0.105
dan_daner_ANX_PANIC_gmdc.	0	0.93	0.481	0.479(247)	0.501(537)	−0.082	0.116
dan_daner_ANX_PANIC_gom3.	0	0.94	0.893	0.504(472)	0.506(1803)	−0.0104	0.077
dan_daner_ANX_PANIC_gwc3.	0	0.92	0.0775	0.512(280)	0.490(855)	0.193	0.109
dan_daner_ANX_PANIC_swdf.	0	0.90	0.00743	0.552(561)	0.510(2591)	0.193	0.072
dan_daner_ANX_QIMR_AGDS.	0	0.97	6.44e−05	0.517(7312)	0.517(12813)	0.116	0.0289
dan_daner_ANX_QIMR_Ad.	0	0.97	0.166	0.487(2449)	0.487(6343)	0.0509	0.0367
dan_daner_ANX_QIMR_Nuup.	0	0.97	0.468	0.497(1131)	0.497(1233)	−0.046	0.0634
dan_daner_ANX_SWE_Catss.	0	1.00	0.251	0.489(540)	0.489(2333)	−0.0805	0.0701
dan_daner_ANX_SWE_Salty.	0	1.00	0.992	0.499(687)	0.499(3445)	−0.000585	0.0606
dan_daner_ANX_SWE_TwinGene.	0	1.00	0.672	0.486(1740)	0.486(6470)	0.0164	0.0389
dan_daner_ANX_UKBB.	0	0.98	0.00455	0.502(25453)	0.502(58113)	0.032	0.0113
dan_daner_ANX_Utah.	1	0.98	0.124	0.541(258)	0.493(279)	0.192	0.125
dan_daner_ANX_alspac.	0	0.95	0.987	0.501(536)	0.502(3404)	−0.00108	0.0689
dan_daner_ANX_iPSYCH.	22	1.01	0.00877	0.516(5288)	0.500(14422)	0.06	0.0229
dan_daner_ANX_musp.	1	0.96	0.00705	0.567(316)	0.505(715)	0.266	0.0989
**dan_**	**26**	**0.99**	**1.61e−10**	**0.520(122225)**	**0.540(729960)**	**0.0315**	**0.0049**

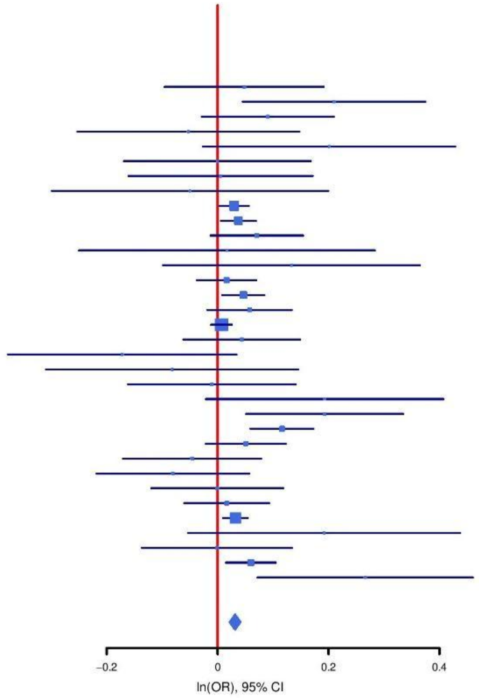

Supplementary Figure 13: Regional association plot (A) and forest plot (B) of SNP rs5015511. See full figure description on page 30.

A

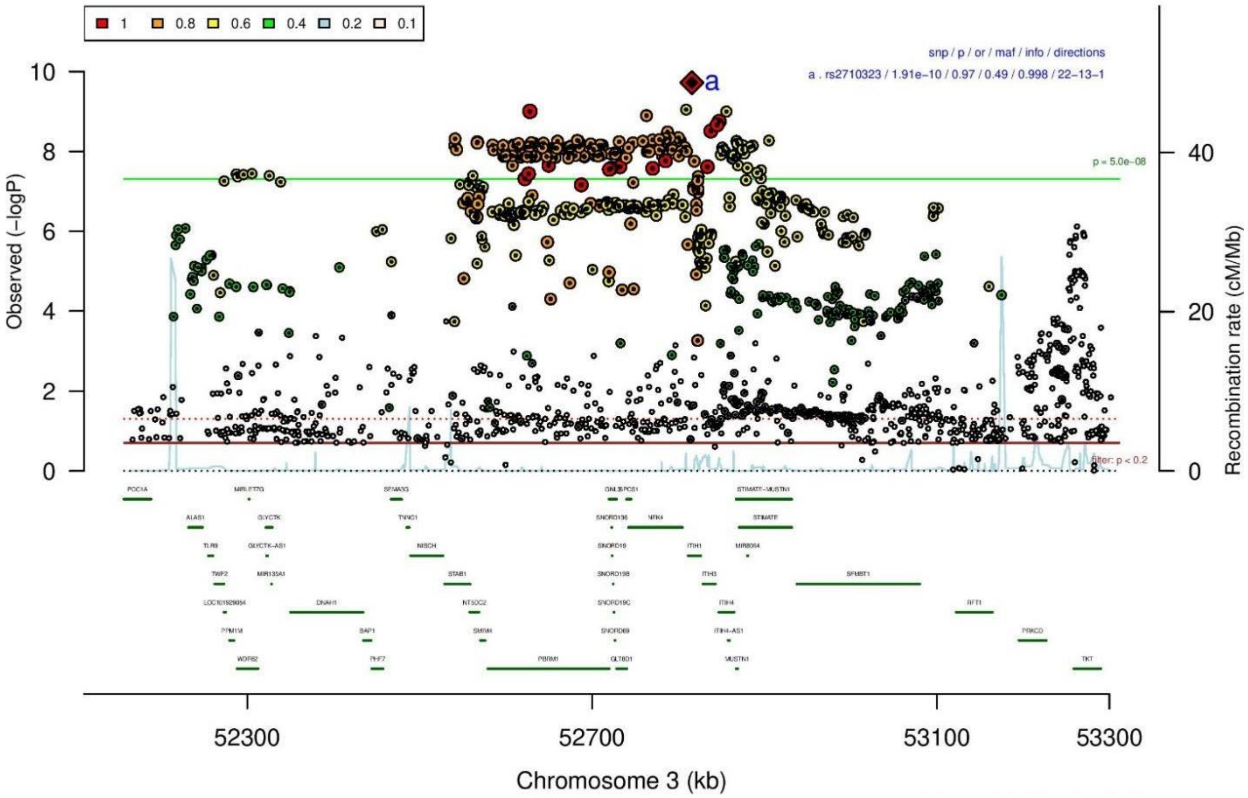

B

rs2710323		C/T		3:52815905			
−?−+−−−−−++++−+++++++++++−++++−+−−+−				het_P:	0.0025	het_I:	44.9
	ngt	info	p_value	f_ca(n)	f_co(n)	ln(OR)	STDerr
dan_daner_ANX_ANGST_mgs.	0	1.00	0.152	0.486(557)	0.486(779)	0.117	0.0815
dan_daner_ANX_ANGST_ntr.	0	1.00	0.0871	0.494(1521)	0.494(2970)	−0.104	0.0606
dan_daner_ANX_ANGST_rs1.	0	1.00	0.00343	0.487(331)	0.487(1629)	0.295	0.101
dan_daner_ANX_ANGST_rs2.	0	1.00	0.223	0.480(209)	0.480(1023)	0.132	0.108
dan_daner_ANX_ANGST_rs3.	0	1.00	0.642	0.494(370)	0.494(1817)	0.0383	0.0823
dan_daner_ANX_ANGST_ship.	0	0.98	0.848	0.501(545)	0.501(834)	0.0159	0.0829
dan_daner_ANX_ANGST_trails.	0	0.98	0.0453	0.501(278)	0.501(336)	0.239	0.119
dan_daner_ANX_EstBB.	0	1.00	6.34e−05	0.512(12523)	0.512(134954)	−0.0536	0.0134
dan_daner_ANX_FinnGen.	0	1.00	0.00784	0.487(9182)	0.487(160108)	−0.0409	0.0154
dan_daner_ANX_GSC.	0	1.00	0.0709	0.447(1306)	0.470(12675)	−0.0759	0.042
dan_daner_ANX_Gedi_CHDS.	0	1.00	0.0936	0.451(244)	0.451(217)	−0.232	0.139
dan_daner_ANX_Gedi_GSMS.	0	1.00	0.709	0.470(208)	0.470(551)	0.0445	0.119
dan_daner_ANX_Gedi_VTSABD.	0	1.00	0.215	0.480(224)	0.480(406)	−0.0332	0.0268
dan_daner_ANX_HUNT.	0	1.00	0.00915	0.481(6587)	0.492(40740)	−0.0503	0.0193
dan_daner_ANX_MGHI.	0	1.00	0.0411	0.455(1823)	0.476(5151)	−0.0796	0.0389
dan_daner_ANX_MVP.	0	1.00	0.00103	0.487(28525)	0.487(163731)	−0.0311	0.0095
dan_daner_ANX_MoBa.	0	1.00	0.145	0.471(715)	0.491(22168)	−0.0788	0.054
dan_daner_ANX_PANIC_dca1.	0	1.00	0.603	0.504(248)	0.519(970)	−0.0527	0.101
dan_daner_ANX_PANIC_gmdc.	1	0.95	0.0391	0.456(247)	0.501(537)	−0.239	0.116
dan_daner_ANX_PANIC_gom3.	0	1.00	0.243	0.480(472)	0.494(1803)	−0.0877	0.0751
dan_daner_ANX_PANIC_gwc3.	1	0.97	0.143	0.532(280)	0.493(855)	0.155	0.106
dan_daner_ANX_PANIC_swdf.	0	1.01	0.674	0.501(561)	0.497(2591)	−0.0285	0.0677
dan_daner_ANX_PBK1.	0	1.00	0.126	0.460(1121)	0.474(11897)	−0.0689	0.045
dan_daner_ANX_QIMR_AGDS.	0	1.00	0.0258	0.465(7312)	0.465(12813)	−0.064	0.0287
dan_daner_ANX_QIMR_Ad.	0	1.00	0.74	0.462(2449)	0.462(6343)	−0.0118	0.0355
dan_daner_ANX_QIMR_Nuup.	0	1.00	0.21	0.479(1131)	0.479(1233)	0.0794	0.0633
dan_daner_ANX_SWE_Catss.	0	1.00	0.318	0.498(540)	0.498(2333)	−0.0709	0.0709
dan_daner_ANX_SWE_Salty.	0	1.00	0.831	0.497(687)	0.497(3445)	0.013	0.0608
dan_daner_ANX_SWE_TwinGene.	0	1.00	0.497	0.490(1740)	0.490(6470)	0.0264	0.0389
dan_daner_ANX_UKBB.	0	1.00	0.311	0.472(25453)	0.472(58113)	−0.0113	0.0112
dan_daner_ANX_Utah.	0	1.02	0.0183	0.520(258)	0.448(279)	0.289	0.122
dan_daner_ANX_alspac.	0	1.00	0.318	0.473(536)	0.459(3404)	0.0675	0.0675
dan_daner_ANX_bioVU.	0	1.00	0.298	0.481(7586)	0.481(50545)	−0.0194	0.0186
dan_daner_ANX_iPSYCH.	0	0.99	0.362	0.500(5288)	0.506(14422)	−0.021	0.0231
dan_daner_ANX_musp.	0	1.03	0.692	0.466(316)	0.474(715)	−0.0376	0.0949
**dan_**	**2**	**1.00**	**1.91e−10**	**0.484(122225)**	**0.490(729960)**	**−0.0299**	**0.0047**

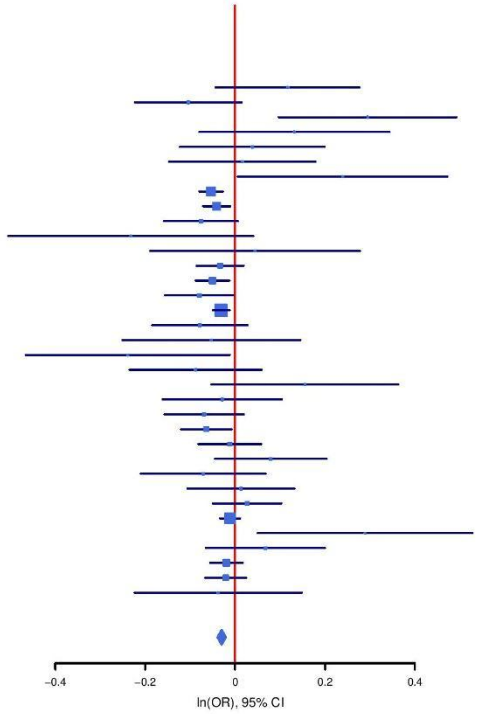

Supplementary Figure 14: Regional association plot (A) and forest plot (B) of SNP rs2710323. See full figure description on page 30.

A

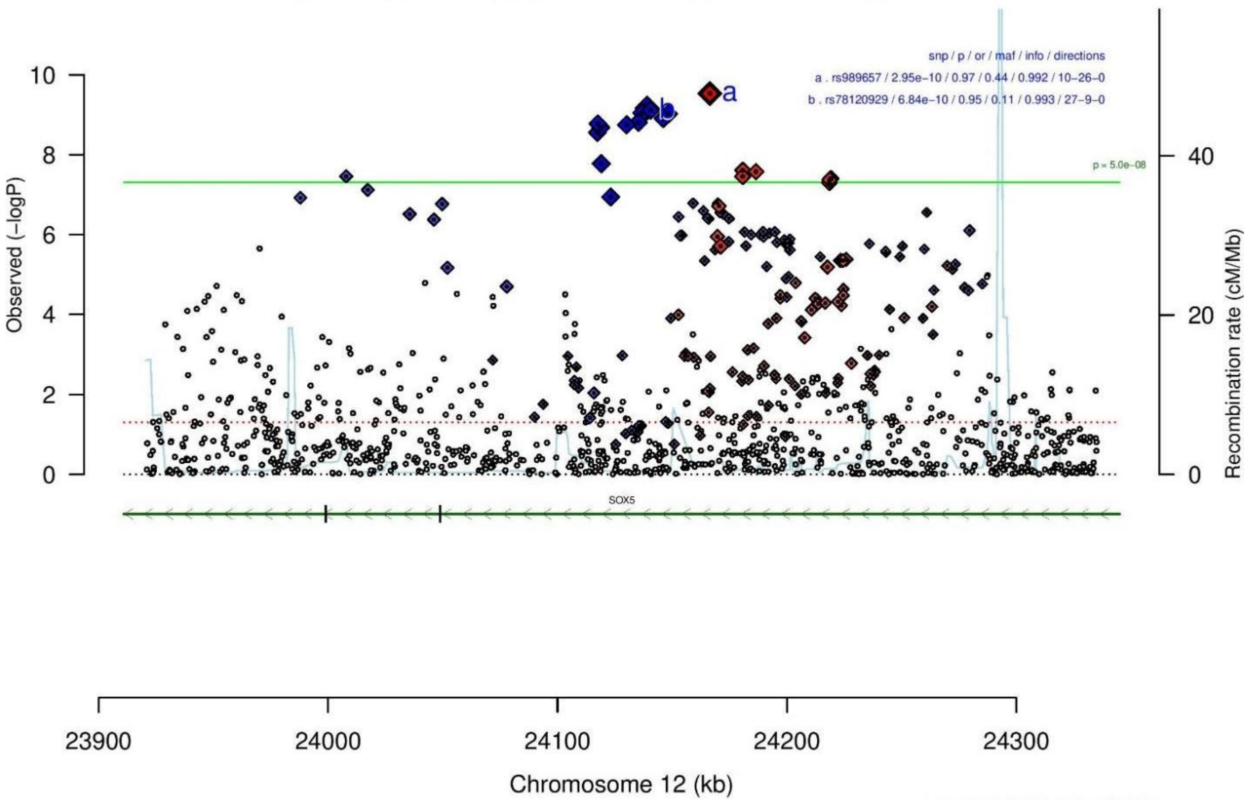

B

rs989657 **C/T** **12:24166426**

−++++−−+−−−−−−+−−−−−−−+++−−+−−−−−−−− het_P: 0.0596 het_I: 28.4

	ngt	info	p_value	f_ca(n)	f_co(n)	ln(OR)	STDerr
dan_daner_ANX_ANGST_gsk.	0	0.95	0.65	0.606(852)	0.606(1103)	−0.0326	0.0718
dan_daner_ANX_ANGST_mgs.	0	1.00	0.693	0.580(557)	0.580(779)	−0.0319	0.0807
dan_daner_ANX_ANGST_ntr.	0	1.00	0.305	0.554(1521)	0.554(2970)	−0.0623	0.0607
dan_daner_ANX_ANGST_rs1.	0	1.00	0.486	0.562(331)	0.562(1629)	−0.068	0.097
dan_daner_ANX_ANGST_rs2.	0	1.00	0.477	0.550(209)	0.550(1023)	0.078	0.109
dan_daner_ANX_ANGST_rs3.	0	1.00	0.452	0.560(370)	0.560(1817)	0.063	0.0838
dan_daner_ANX_ANGST_ship.	0	1.00	0.41	0.541(545)	0.541(834)	−0.066	0.0802
dan_daner_ANX_ANGST_trails.	0	0.93	0.108	0.541(278)	0.541(336)	0.203	0.126
dan_daner_ANX_EstBB.	0	0.99	0.0783	0.527(12523)	0.527(134954)	0.0238	0.0135
dan_daner_ANX_FinnGen.	0	0.99	0.000415	0.560(9182)	0.560(160108)	0.0548	0.0155
dan_daner_ANX_GSC.	1	1.02	0.742	0.548(1306)	0.541(12675)	0.0137	0.0416
dan_daner_ANX_Gedi_CHDS.	0	1.00	0.431	0.542(244)	0.542(217)	0.111	0.141
dan_daner_ANX_Gedi_GSMS.	0	1.00	6.64e−05	0.567(208)	0.567(551)	0.516	0.129
dan_daner_ANX_Gedi_VTSABD.	0	1.00	0.0862	0.566(224)	0.566(406)	−0.0481	0.028
dan_daner_ANX_HUNT.	0	0.99	0.0163	0.601(6587)	0.588(40740)	0.0477	0.0199
dan_daner_ANX_MGHI.	0	0.98	0.812	0.597(1823)	0.598(5151)	0.00955	0.0401
dan_daner_ANX_MVP.	0	1.00	4.01e−05	0.584(28525)	0.584(163731)	0.0411	0.01
dan_daner_ANX_MoBa.	0	1.00	0.237	0.592(715)	0.574(22168)	0.0642	0.0543
dan_daner_ANX_PANIC_dca1.	0	0.93	0.297	0.551(248)	0.577(970)	−0.11	0.106
dan_daner_ANX_PANIC_gmdc.	0	0.98	0.918	0.597(247)	0.589(537)	−0.0118	0.115
dan_daner_ANX_PANIC_gom3.	0	0.95	0.0678	0.544(472)	0.568(1803)	−0.141	0.0774
dan_daner_ANX_PANIC_gwc3.	1	1.02	0.776	0.579(280)	0.584(855)	0.0301	0.106
dan_daner_ANX_PANIC_swdf.	0	0.98	0.737	0.587(561)	0.589(2591)	0.0234	0.0697
dan_daner_ANX_PBK1.	0	1.00	0.899	0.578(1121)	0.574(11897)	−0.00598	0.0454
dan_daner_ANX_QIMR_AGDS.	0	0.98	0.549	0.559(7312)	0.559(12813)	0.0173	0.0288
dan_daner_ANX_QIMR_Ad.	0	0.98	0.423	0.546(2449)	0.546(6343)	0.029	0.0362
dan_daner_ANX_QIMR_Nuup.	0	0.98	0.189	0.562(1131)	0.562(1233)	0.0832	0.0633
dan_daner_ANX_SWE_Catss.	0	1.00	0.375	0.416(540)	0.416(2333)	0.0661	0.0744
dan_daner_ANX_SWE_Salty.	0	1.00	0.298	0.427(687)	0.427(3445)	0.0639	0.0613
dan_daner_ANX_SWE_TwinGene.	0	1.00	0.938	0.423(1740)	0.423(6470)	0.00301	0.0388
dan_daner_ANX_UKBB.	0	0.98	0.0316	0.559(25453)	0.559(58113)	0.0243	0.0113
dan_daner_ANX_Utah.	0	1.00	0.653	0.583(258)	0.564(279)	0.057	0.127
dan_daner_ANX_alspac.	0	1.00	0.029	0.596(536)	0.558(3404)	0.147	0.0675
dan_daner_ANX_bioVU.	0	1.00	0.127	0.579(7586)	0.579(50545)	0.0288	0.0189
dan_daner_ANX_iPSYCH.	0	0.97	0.161	0.577(5288)	0.568(14422)	0.0332	0.0237
dan_daner_ANX_musp.	0	0.99	0.934	0.576(316)	0.578(715)	0.00813	0.0978
**dan_**	**2**	**0.99**	**2.95e−10**	**0.564(122225)**	**0.561(729960)**	**0.0302**	**0.0048**

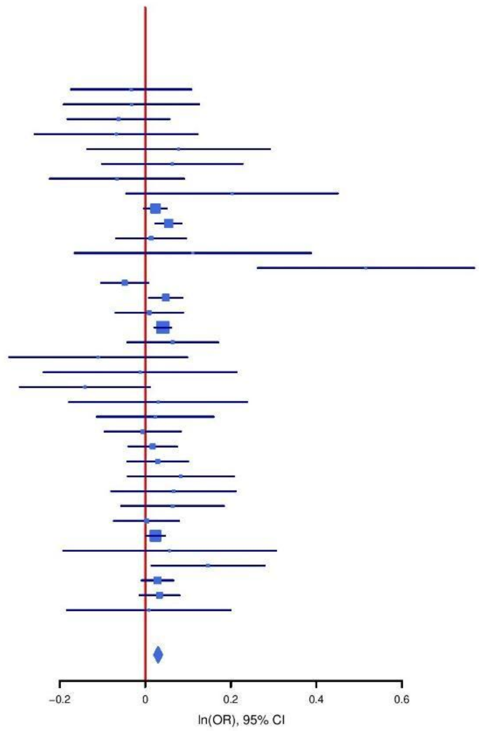

C

rs78120929 C/T 12:24139063

+++−−+++−+++++−+++++++−++−−−++++−+++ het_P: 0.198 het_I: 16.4

	ngt	info	p_value	f_ca(n)	f_co(n)	ln(OR)	STDerr
dan_daner_ANX_ANGST_gsk.	0	0.96	0.149	0.111(852)	0.111(1103)	−0.157	0.109
dan_daner_ANX_ANGST_mgs.	0	1.00	0.753	0.114(557)	0.114(779)	−0.0405	0.129
dan_daner_ANX_ANGST_ntr.	0	0.99	0.22	0.129(1521)	0.129(2970)	0.114	0.0927
dan_daner_ANX_ANGST_rs1.	0	1.00	0.166	0.122(331)	0.122(1629)	0.195	0.141
dan_daner_ANX_ANGST_rs2.	0	0.99	0.538	0.121(209)	0.121(1023)	−0.099	0.161
dan_daner_ANX_ANGST_rs3.	0	1.00	0.798	0.121(370)	0.121(1817)	−0.0326	0.128
dan_daner_ANX_ANGST_ship.	0	0.99	0.554	0.124(545)	0.124(834)	−0.0744	0.126
dan_daner_ANX_ANGST_trails.	0	0.93	0.802	0.132(278)	0.132(336)	0.0477	0.19
dan_daner_ANX_EstBB.	0	0.98	0.0248	0.109(12523)	0.109(134954)	−0.0493	0.022
dan_daner_ANX_FinnGen.	0	1.00	0.0165	0.095(9182)	0.095(160108)	−0.0642	0.0268
dan_daner_ANX_GSC.	0	0.99	0.0066	0.109(1306)	0.127(12675)	−0.179	0.0669
dan_daner_ANX_Gedi_CHDS.	0	1.00	0.207	0.118(244)	0.118(217)	−0.264	0.209
dan_daner_ANX_Gedi_GSMS.	0	1.00	0.31	0.095(208)	0.095(551)	−0.212	0.209
dan_daner_ANX_Gedi_VTSABD.	0	1.00	0.0676	0.115(224)	0.115(406)	0.08	0.0437
dan_daner_ANX_HUNT.	0	0.98	0.144	0.105(6587)	0.112(40740)	−0.0452	0.0309
dan_daner_ANX_MGHI.	0	0.97	0.774	0.110(1823)	0.112(5151)	−0.0179	0.0624
dan_daner_ANX_MVP.	0	1.00	0.00838	0.114(28525)	0.114(163731)	−0.0395	0.015
dan_daner_ANX_MoBa.	0	1.00	0.107	0.094(715)	0.107(22168)	−0.14	0.0867
dan_daner_ANX_PANIC_dca1.	0	0.99	0.387	0.121(248)	0.109(970)	0.137	0.158
dan_daner_ANX_PANIC_gmdc.	0	0.98	0.098	0.086(247)	0.119(537)	−0.32	0.194
dan_daner_ANX_PANIC_gom3.	0	0.96	0.761	0.116(472)	0.119(1803)	−0.0362	0.119
dan_daner_ANX_PANIC_gwc3.	0	1.00	0.251	0.114(280)	0.108(855)	0.192	0.167
dan_daner_ANX_PANIC_swdf.	0	0.90	0.963	0.105(561)	0.101(2591)	0.00541	0.117
dan_daner_ANX_PBK1.	0	1.00	0.382	0.118(1121)	0.113(11897)	0.0602	0.0686
dan_daner_ANX_QIMR_AGDS.	0	0.98	0.62	0.118(7312)	0.118(12813)	−0.0219	0.0442
dan_daner_ANX_QIMR_Ad.	0	0.98	0.38	0.115(2449)	0.115(6343)	−0.0495	0.0564
dan_daner_ANX_QIMR_Nuup.	0	0.98	0.269	0.112(1131)	0.112(1233)	−0.106	0.096
dan_daner_ANX_SWE_Catss.	0	1.00	0.837	0.102(540)	0.102(2333)	−0.0239	0.116
dan_daner_ANX_SWE_Salty.	0	1.00	0.832	0.105(687)	0.105(3445)	0.0208	0.0982
dan_daner_ANX_SWE_TwinGene.	0	1.00	0.752	0.106(1740)	0.106(6470)	−0.0201	0.0636
dan_daner_ANX_UKBB.	0	1.00	0.00751	0.117(25453)	0.117(58113)	−0.0463	0.0173
dan_daner_ANX_Utah.	0	1.06	0.668	0.107(258)	0.116(279)	−0.0815	0.19
dan_daner_ANX_alspac.	0	0.99	0.274	0.106(536)	0.121(3404)	−0.117	0.108
dan_daner_ANX_bioVU.	0	1.00	0.0163	0.114(7586)	0.114(50545)	−0.0717	0.0298
dan_daner_ANX_iPSYCH.	0	0.97	0.00138	0.102(5288)	0.114(14422)	−0.122	0.038
dan_daner_ANX_musp.	0	0.98	0.0226	0.089(316)	0.123(715)	−0.375	0.165
**dan_**	**0**	**0.99**	**6.84e−10**	**0.112(122225)**	**0.109(729960)**	**−0.0463**	**0.0075**

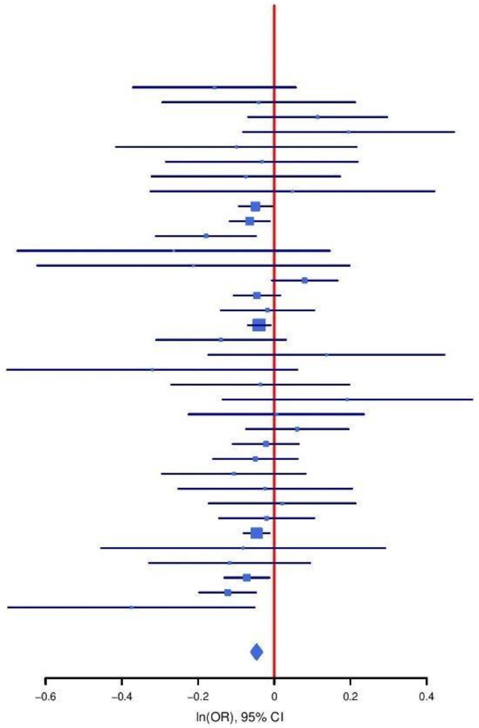

Supplementary Figure 15: Regional association plot (A) and forest plots (B, C) of SNPs rs989657 and rs78120929. See full figure description on page 30.

A

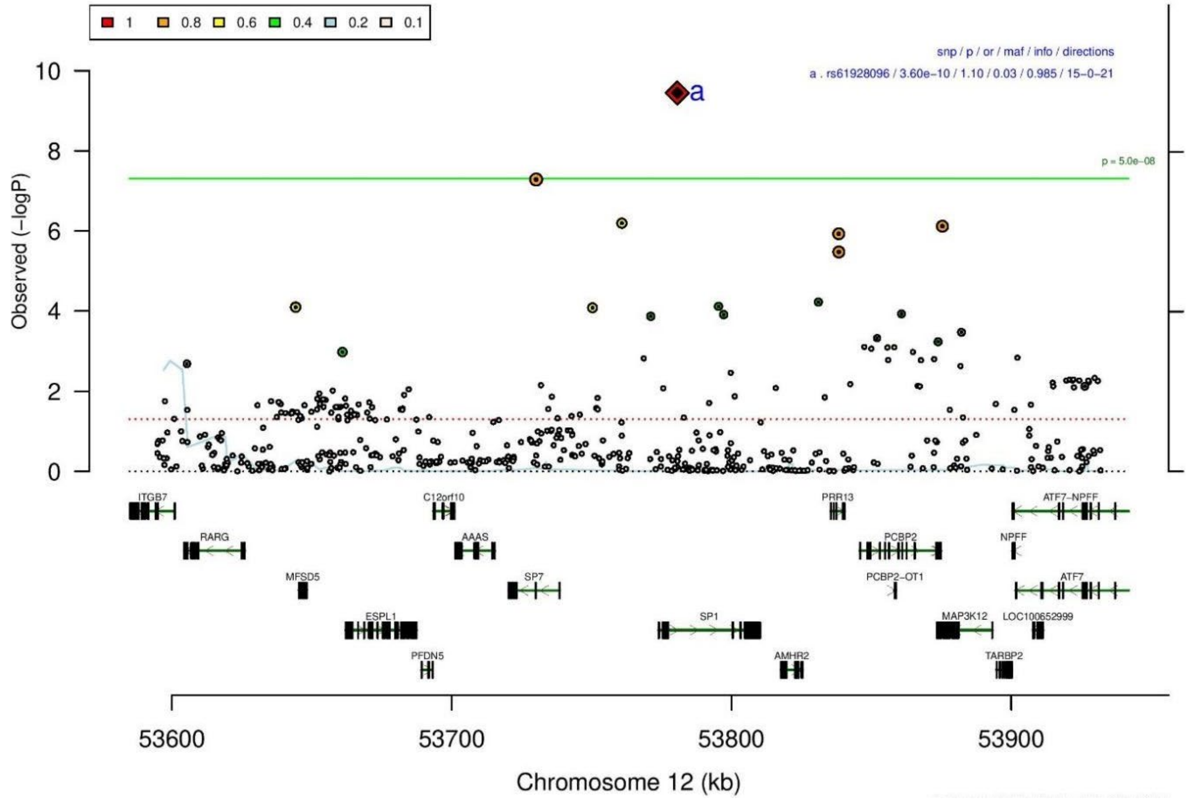

B

rs61928096 **A/G** **12:53780633**

+?????????+?++++++?+?+??????+++??++? het_P: 0.327 het_I: 11.3

	ngt	info	p_value	f_ca(n)	f_co(n)	ln(OR)	STDerr
dan_daner_ANX_EstBB.	0	1.00	0.00526	0.014(12523)	0.014(134954)	0.152	0.0543
dan_daner_ANX_GSC.	0	0.93	0.36	0.060(1306)	0.056(12675)	0.0867	0.0947
dan_daner_ANX_Gedi_CHDS.	0	1.00	0.922	0.044(244)	0.044(217)	0.0317	0.325
dan_daner_ANX_Gedi_GSMS.	0	1.00	0.163	0.040(208)	0.040(551)	0.416	0.298
dan_daner_ANX_Gedi_VTSABD.	0	1.00	0.468	0.029(224)	0.029(406)	0.0598	0.0825
dan_daner_ANX_HUNT.	0	0.92	0.0187	0.043(6587)	0.038(40740)	0.119	0.0498
dan_daner_ANX_MVP.	0	1.00	0.109	0.036(28525)	0.036(163731)	0.0438	0.0273
dan_daner_ANX_MoBa.	0	1.00	0.0153	0.050(715)	0.037(22168)	0.34	0.14
dan_daner_ANX_QIMR_AGDS.	0	1.00	0.0841	0.040(7312)	0.040(12813)	0.127	0.0734
dan_daner_ANX_QIMR_Ad.	0	1.00	0.00866	0.042(2449)	0.042(6343)	0.257	0.098
dan_daner_ANX_QIMR_Nuup.	0	1.00	0.498	0.046(1131)	0.046(1233)	0.11	0.162
dan_daner_ANX_SWE_TwinGene.	0	1.00	0.0271	0.019(1740)	0.019(6470)	0.299	0.135
dan_daner_ANX_UKBB.	0	1.00	0.00219	0.042(25453)	0.042(58113)	0.0846	0.0276
dan_daner_ANX_alspac.	0	0.90	0.122	0.052(536)	0.040(3404)	0.252	0.159
dan_daner_ANX_iPSYCH.	0	0.81	0.0959	0.029(5288)	0.026(14422)	0.129	0.0774
**dan_**	**0**	**0.98**	**3.6e-10**	**0.036(122225)**	**0.031(729960)**	**0.0955**	**0.0152**

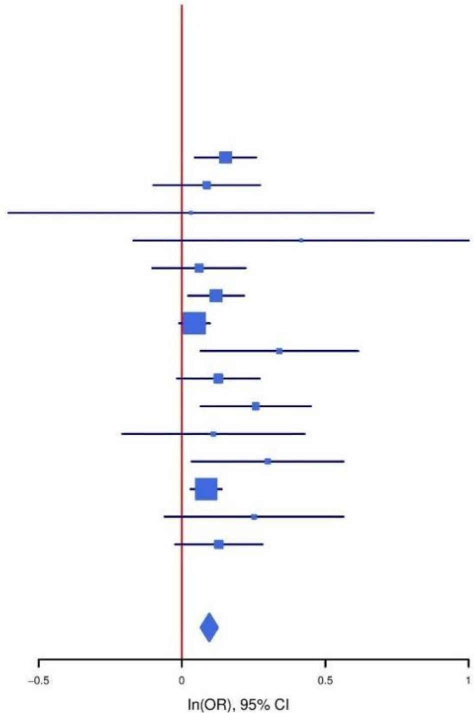

Supplementary Figure 16: Regional association plot (A) and forest plot (B) of SNP rs61928096. See full figure description on page 30.

A

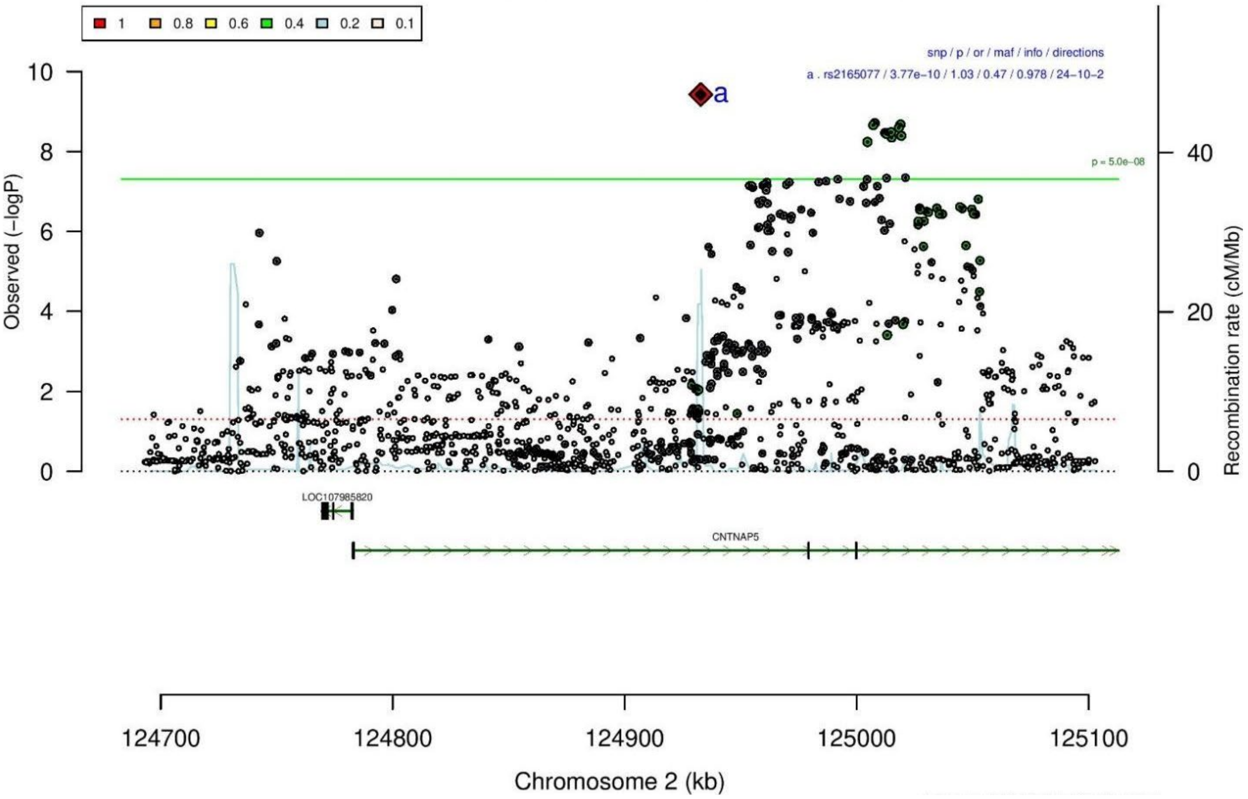

B

rs2165077		T/C		2:124932847			
++−++−−+−?+++−+−+++++++−++−?−++++++−				het_P:	0.862	het_I:	0.0
	ngt	info	p_value	f_ca(n)	f_co(n)	ln(OR)	STDerr
dan_daner_ANX_ANGST_gsk.	0	0.86	0.373	0.473(852)	0.473(1103)	0.0641	0.072
dan_daner_ANX_ANGST_mgs.	0	1.00	0.987	0.505(557)	0.505(779)	−0.00135	0.0856
dan_daner_ANX_ANGST_ntr.	0	0.87	0.323	0.481(1521)	0.481(2970)	0.0652	0.0659
dan_daner_ANX_ANGST_rs1.	0	0.90	0.18	0.492(331)	0.492(1629)	0.141	0.105
dan_daner_ANX_ANGST_rs2.	0	0.90	0.345	0.475(209)	0.475(1023)	−0.107	0.114
dan_daner_ANX_ANGST_rs3.	0	0.90	0.265	0.476(370)	0.476(1817)	−0.0949	0.0851
dan_daner_ANX_ANGST_ship.	0	0.92	0.474	0.470(545)	0.470(834)	0.0605	0.0845
dan_daner_ANX_ANGST_trails.	0	0.89	0.974	0.517(278)	0.517(336)	−0.00409	0.126
dan_daner_ANX_EstBB.	0	0.97	0.00397	0.447(12523)	0.447(134954)	0.0393	0.0136
dan_daner_ANX_FinnGen.	0	0.99	0.00318	0.443(9182)	0.443(160108)	0.0455	0.0154
dan_daner_ANX_GSC.	0	0.95	0.445	0.478(1306)	0.486(12675)	−0.033	0.0432
dan_daner_ANX_Gedi_CHDS.	0	1.00	0.136	0.479(244)	0.479(217)	0.214	0.144
dan_daner_ANX_Gedi_GSMS.	0	1.00	0.997	0.491(208)	0.491(551)	−0.000454	0.117
dan_daner_ANX_Gedi_VTSABD.	0	1.00	0.762	0.502(224)	0.502(406)	0.00808	0.0267
dan_daner_ANX_HUNT.	0	0.98	0.0171	0.511(6587)	0.501(40740)	0.0465	0.0195
dan_daner_ANX_MGHI.	0	0.95	0.0329	0.496(1823)	0.476(5151)	0.0847	0.0397
dan_daner_ANX_MVP.	0	1.00	0.0139	0.482(28525)	0.482(163731)	0.0244	0.0099
dan_daner_ANX_MoBa.	0	1.00	0.238	0.522(715)	0.505(22168)	0.0638	0.0541
dan_daner_ANX_PANIC_dca1.	0	0.87	0.533	0.514(248)	0.500(970)	0.0678	0.109
dan_daner_ANX_PANIC_gmdc.	0	0.90	0.345	0.459(247)	0.482(537)	−0.111	0.118
dan_daner_ANX_PANIC_gom3.	0	0.86	0.556	0.499(472)	0.491(1803)	0.0475	0.0805
dan_daner_ANX_PANIC_gwc3.	0	0.90	0.424	0.480(280)	0.464(855)	0.0889	0.111
dan_daner_ANX_PANIC_swdf.	0	0.92	0.264	0.479(561)	0.505(2591)	−0.0795	0.0711
dan_daner_ANX_QIMR_AGDS.	0	0.95	0.624	0.485(7312)	0.485(12813)	0.0144	0.0294
dan_daner_ANX_QIMR_Ad.	0	0.95	0.989	0.486(2449)	0.486(6343)	−0.000483	0.0364
dan_daner_ANX_QIMR_Nuup.	0	0.95	0.392	0.491(1131)	0.491(1233)	0.0546	0.0638
dan_daner_ANX_SWE_Catss.	0	1.00	0.947	0.485(540)	0.485(2333)	0.005	0.0749
dan_daner_ANX_SWE_Salty.	0	1.00	0.0664	0.490(687)	0.490(3445)	0.119	0.0649
dan_daner_ANX_SWE_TwinGene.	0	1.00	0.291	0.485(1740)	0.485(6470)	0.0423	0.0401
dan_daner_ANX_UKBB.	0	0.96	0.0119	0.488(25453)	0.488(58113)	0.0285	0.0113
dan_daner_ANX_Utah.	0	0.89	0.399	0.486(258)	0.509(279)	−0.109	0.13
dan_daner_ANX_alspac.	0	0.94	0.173	0.511(536)	0.489(3404)	0.0934	0.0686
dan_daner_ANX_iPSYCH.	0	0.94	0.169	0.505(5288)	0.498(14422)	0.0327	0.0238
dan_daner_ANX_musp.	0	0.94	0.883	0.461(316)	0.459(715)	0.0146	0.0994
**dan_**	**0**	**0.98**	**3.77e−10**	**0.480(122225)**	**0.469(729960)**	**0.0312**	**0.005**

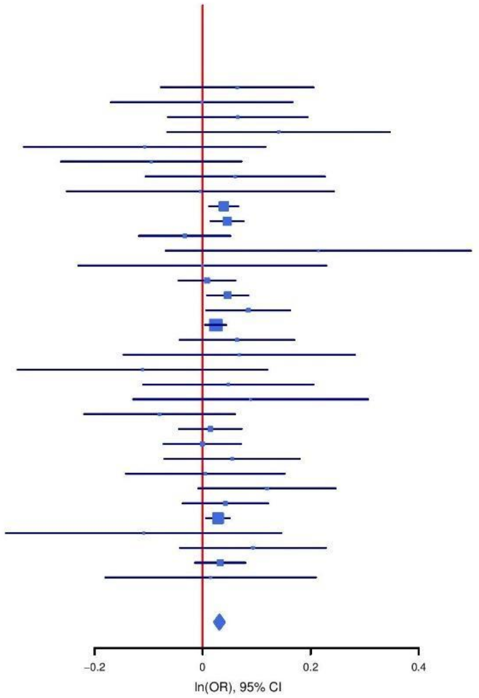

Supplementary Figure 17: Regional association plot (A) and forest plot (B) of SNP rs2165077. See full figure description on page 30.

A

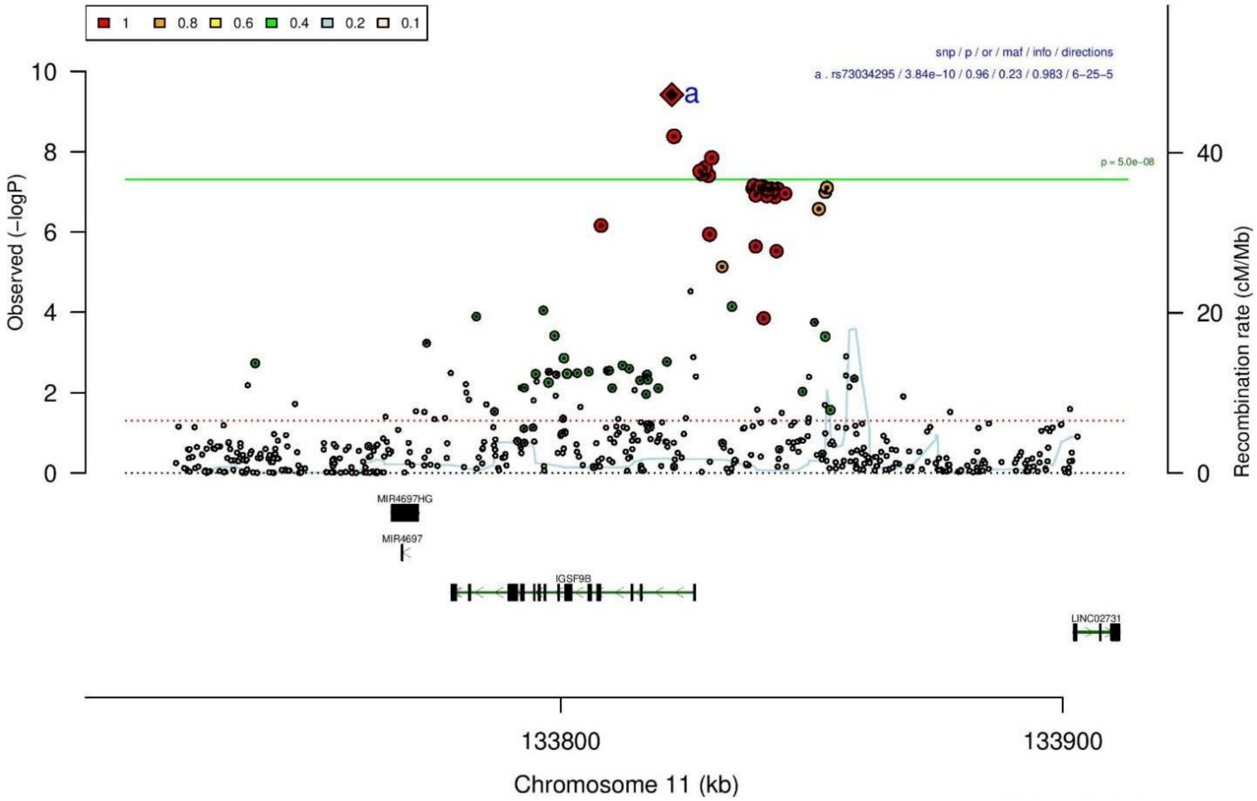

B

rs73034295		A/G		11:133822133			
+−+−−−−+??−−−−+−−−−−−−−?−?−?+−−−+−−−−				het_P:	0.426	het_I:	2.5
	ngt	info	p_value	f_ca(n)	f_co(n)	ln(OR)	STDerr
dan_daner_ANX_ANGST_gsk.	0	0.94	0.407	0.183(852)	0.183(1103)	−0.0745	0.0898
dan_daner_ANX_ANGST_mgs.	0	1.00	0.54	0.198(557)	0.198(779)	0.0619	0.101
dan_daner_ANX_ANGST_ntr.	0	0.95	0.339	0.225(1521)	0.225(2970)	−0.0727	0.076
dan_daner_ANX_ANGST_rs1.	0	0.95	0.598	0.222(331)	0.222(1629)	−0.064	0.122
dan_daner_ANX_ANGST_rs2.	0	0.94	0.0604	0.222(209)	0.222(1023)	−0.269	0.143
dan_daner_ANX_ANGST_rs3.	0	0.96	0.468	0.231(370)	0.231(1817)	−0.0709	0.0978
dan_daner_ANX_ANGST_ship.	0	0.96	0.978	0.231(545)	0.231(834)	0.00266	0.0976
dan_daner_ANX_EstBB.	0	0.99	0.0682	0.265(12523)	0.265(134954)	−0.0278	0.0152
dan_daner_ANX_FinnGen.	0	0.98	0.179	0.279(9182)	0.279(160108)	−0.0231	0.0172
dan_daner_ANX_GSC.	0	0.96	0.347	0.188(1306)	0.195(12675)	−0.0506	0.0538
dan_daner_ANX_Gedi_CHDS.	0	1.00	0.675	0.194(244)	0.194(217)	−0.0749	0.179
dan_daner_ANX_Gedi_GSMS.	0	1.00	0.271	0.187(208)	0.187(551)	−0.174	0.158
dan_daner_ANX_Gedi_VTSABD.	0	1.00	0.73	0.192(224)	0.192(406)	0.0124	0.036
dan_daner_ANX_HUNT.	0	0.91	0.577	0.250(6587)	0.254(40740)	−0.0131	0.0234
dan_daner_ANX_MGHI.	0	0.94	0.398	0.178(1823)	0.184(5151)	−0.0438	0.0518
dan_daner_ANX_MVP.	0	1.00	2.33e−06	0.192(28525)	0.192(163731)	−0.0592	0.0125
dan_daner_ANX_MoBa.	0	1.00	0.826	0.229(715)	0.230(22168)	−0.0141	0.0642
dan_daner_ANX_PANIC_gmdc.	0	0.91	0.164	0.174(247)	0.206(537)	−0.211	0.152
dan_daner_ANX_PANIC_gwc3.	0	0.97	0.0374	0.198(280)	0.230(855)	−0.273	0.131
dan_daner_ANX_PBK1.	0	1.00	0.915	0.182(1121)	0.185(11897)	0.00598	0.0581
dan_daner_ANX_QIMR_AGDS.	0	0.98	0.24	0.193(7312)	0.193(12813)	−0.0427	0.0363
dan_daner_ANX_QIMR_Ad.	0	0.98	0.737	0.193(2449)	0.193(6343)	−0.0163	0.0484
dan_daner_ANX_QIMR_Nuup.	0	0.98	0.69	0.202(1131)	0.202(1233)	−0.0334	0.0837
dan_daner_ANX_SWE_Catss.	0	1.00	0.944	0.231(540)	0.231(2333)	0.00604	0.0854
dan_daner_ANX_SWE_Salty.	0	1.00	0.805	0.236(687)	0.236(3445)	−0.0179	0.0725
dan_daner_ANX_SWE_TwinGene.	0	1.00	0.0187	0.239(1740)	0.239(6470)	−0.11	0.0468
dan_daner_ANX_UKBB.	0	0.98	0.0108	0.194(25453)	0.194(58113)	−0.0365	0.0143
dan_daner_ANX_Utah.	0	0.98	0.0338	0.170(258)	0.222(279)	−0.337	0.159
dan_daner_ANX_alspac.	0	0.97	0.0715	0.213(536)	0.189(3404)	0.152	0.0837
dan_daner_ANX_iPSYCH.	0	0.98	0.124	0.229(5288)	0.236(14422)	−0.0424	0.0276
dan_daner_ANX_musp.	0	0.97	0.12	0.183(316)	0.213(715)	−0.193	0.124
**dan_**	**0**	**0.98**	**3.84e−10**	**0.215(122225)**	**0.235(729960)**	**−0.0378**	**0.006**

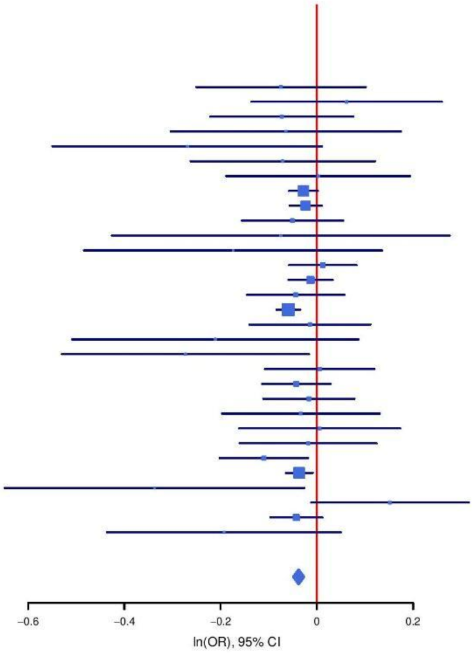

Supplementary Figure 18: Regional association plot (A) and forest plot (B) of SNP rs73034295. See full figure description on page 30.

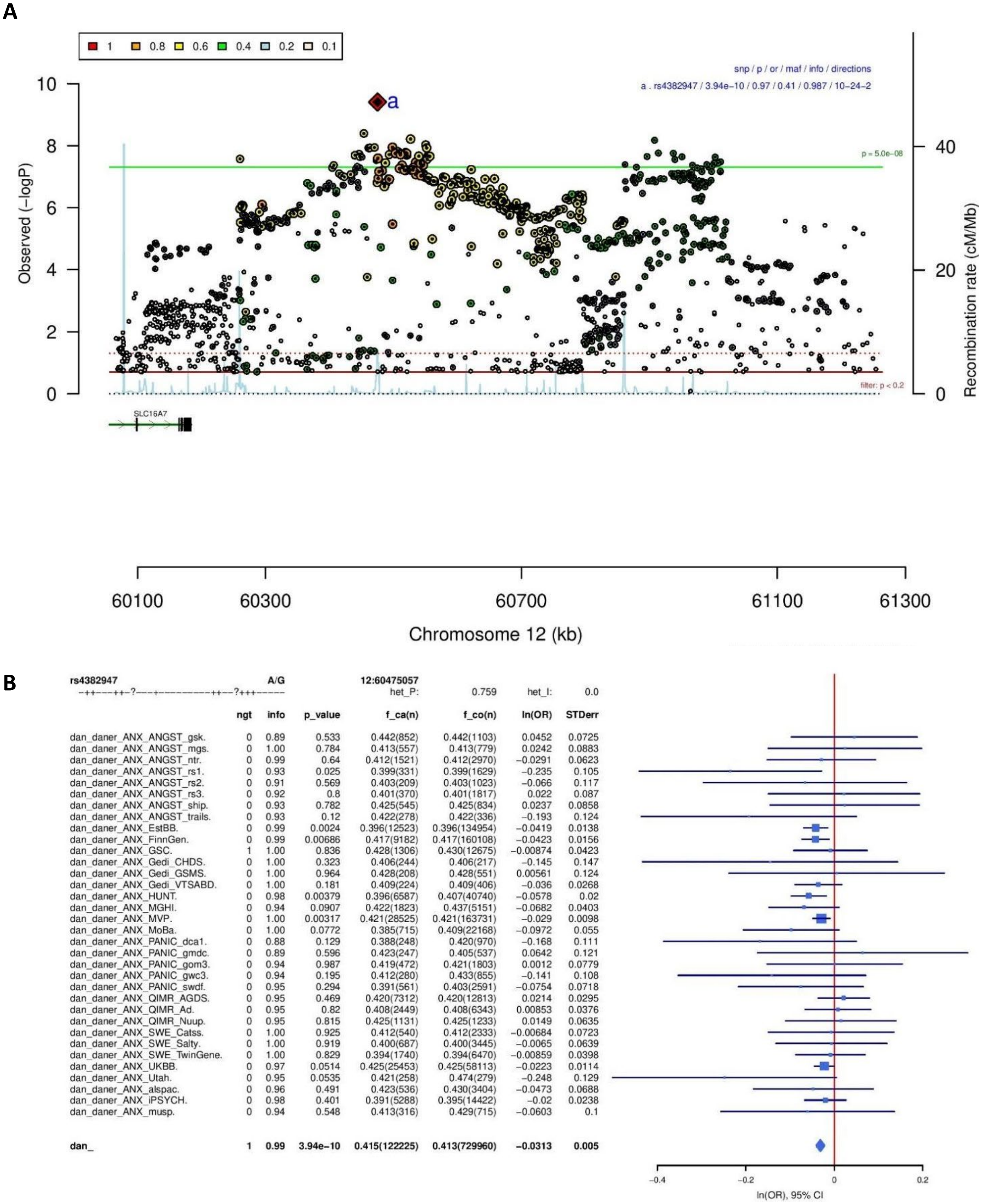

rs4382947			A/G	12:60475057			
−++−−−++−?−−−+−−−−−−−−−−−++−−?+++−−−−−				het_P:	0.759	het_I:	0.0
	ngt	info	p_value	f_ca(n)	f_co(n)	ln(OR)	STDerr
dan_daner_ANX_ANGST_gsk.	0	0.89	0.533	0.442(852)	0.442(1103)	0.0452	0.0725
dan_daner_ANX_ANGST_mgs.	0	1.00	0.784	0.413(557)	0.413(779)	0.0242	0.0883
dan_daner_ANX_ANGST_ntr.	0	0.99	0.64	0.412(1521)	0.412(2970)	−0.0291	0.0623
dan_daner_ANX_ANGST_rs1.	0	0.93	0.025	0.399(331)	0.399(1629)	−0.235	0.105
dan_daner_ANX_ANGST_rs2.	0	0.91	0.569	0.403(209)	0.403(1023)	−0.066	0.117
dan_daner_ANX_ANGST_rs3.	0	0.92	0.8	0.401(370)	0.401(1817)	0.022	0.087
dan_daner_ANX_ANGST_ship.	0	0.93	0.782	0.425(545)	0.425(834)	0.0237	0.0858
dan_daner_ANX_ANGST_trails.	0	0.93	0.12	0.422(278)	0.422(336)	−0.193	0.124
dan_daner_ANX_EstBB.	0	0.99	0.0024	0.396(12523)	0.396(134954)	−0.0419	0.0138
dan_daner_ANX_FinnGen.	0	0.99	0.00686	0.417(9182)	0.417(160108)	−0.0423	0.0156
dan_daner_ANX_GSC.	1	1.00	0.836	0.428(1306)	0.430(12675)	−0.00874	0.0423
dan_daner_ANX_Gedi_CHDS.	0	1.00	0.323	0.406(244)	0.406(217)	−0.145	0.147
dan_daner_ANX_Gedi_GSMS.	0	1.00	0.964	0.428(208)	0.428(551)	0.00561	0.124
dan_daner_ANX_Gedi_VTSABD.	0	1.00	0.181	0.409(224)	0.409(406)	−0.036	0.0268
dan_daner_ANX_HUNT.	0	0.98	0.00379	0.396(6587)	0.407(40740)	−0.0578	0.02
dan_daner_ANX_MGHI.	0	0.94	0.0907	0.422(1823)	0.437(5151)	−0.0682	0.0403
dan_daner_ANX_MVP.	0	1.00	0.00317	0.421(28525)	0.421(163731)	−0.029	0.0098
dan_daner_ANX_MoBa.	0	1.00	0.0772	0.385(715)	0.409(22168)	−0.0972	0.055
dan_daner_ANX_PANIC_dca1.	0	0.88	0.129	0.388(248)	0.420(970)	−0.168	0.111
dan_daner_ANX_PANIC_gmdc.	0	0.89	0.596	0.423(247)	0.405(537)	0.0642	0.121
dan_daner_ANX_PANIC_gom3.	0	0.94	0.987	0.419(472)	0.421(1803)	0.0012	0.0779
dan_daner_ANX_PANIC_gwc3.	0	0.94	0.195	0.412(280)	0.433(855)	−0.141	0.108
dan_daner_ANX_PANIC_swdf.	0	0.95	0.294	0.391(561)	0.403(2591)	−0.0754	0.0718
dan_daner_ANX_QIMR_AGDS.	0	0.95	0.469	0.420(7312)	0.420(12813)	0.0214	0.0295
dan_daner_ANX_QIMR_Ad.	0	0.95	0.82	0.408(2449)	0.408(6343)	0.00853	0.0376
dan_daner_ANX_QIMR_Nuup.	0	0.95	0.815	0.425(1131)	0.425(1233)	0.0149	0.0635
dan_daner_ANX_SWE_Catss.	0	1.00	0.925	0.412(540)	0.412(2333)	−0.00684	0.0723
dan_daner_ANX_SWE_Salty.	0	1.00	0.919	0.400(687)	0.400(3445)	−0.0065	0.0639
dan_daner_ANX_SWE_TwinGene.	0	1.00	0.829	0.394(1740)	0.394(6470)	−0.00859	0.0398
dan_daner_ANX_UKBB.	0	0.97	0.0514	0.425(25453)	0.425(58113)	−0.0223	0.0114
dan_daner_ANX_Utah.	0	0.95	0.0535	0.421(258)	0.474(279)	−0.248	0.129
dan_daner_ANX_alspac.	0	0.96	0.491	0.423(536)	0.430(3404)	−0.0473	0.0688
dan_daner_ANX_iPSYCH.	0	0.98	0.401	0.391(5288)	0.395(14422)	−0.02	0.0238
dan_daner_ANX_musp.	0	0.94	0.548	0.413(316)	0.429(715)	−0.0603	0.1
**dan_**	**1**	**0.99**	**3.94e−10**	**0.415(122225)**	**0.413(729960)**	**−0.0313**	**0.005**

Supplementary Figure 19: Regional association plot (A) and forest plot (B) of SNP rs4382947. See full figure description on page 30.

A

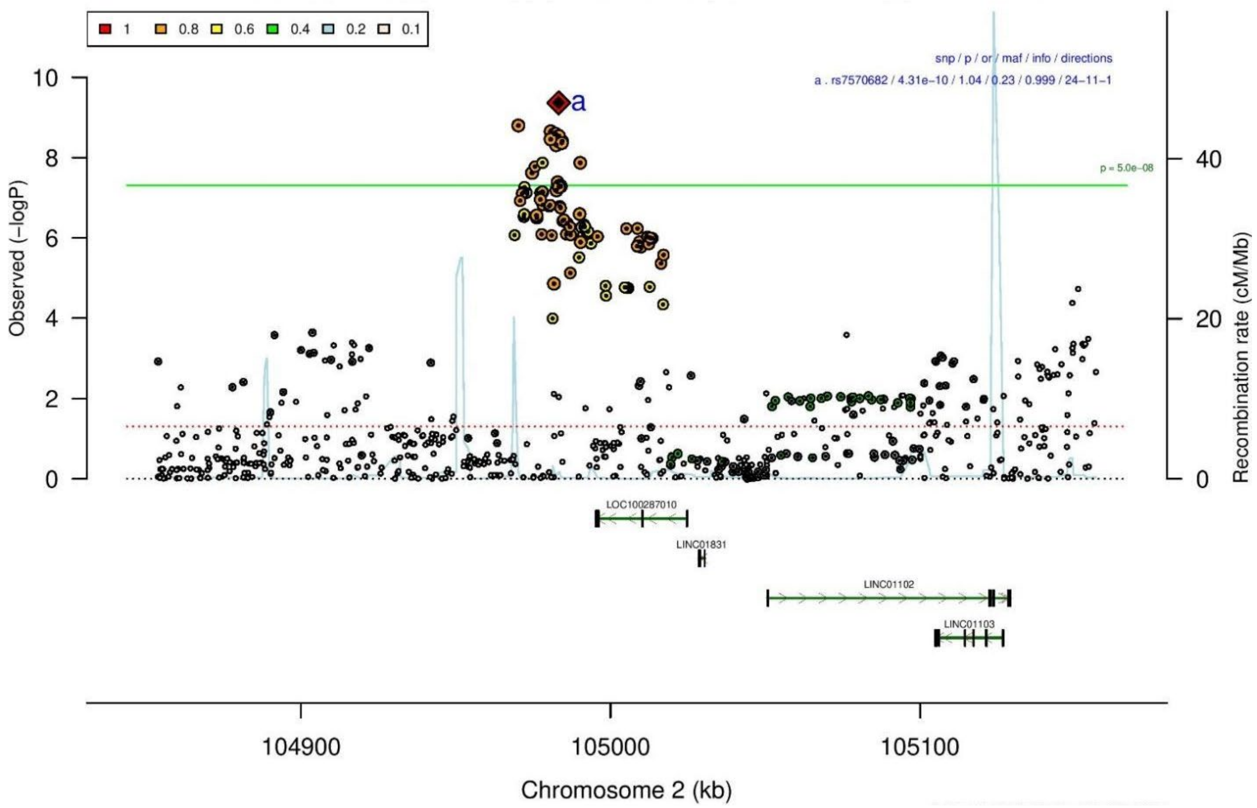

B

rs7570682		A/G		2:104983267			
−?+++−+−−+−++−++++−+−+−+++++−+++−+++				het_P:	0.16	het_I:	19.3
	**ngt**	**info**	**p_value**	**f_ca(n)**	**f_co(n)**	**ln(OR)**	**STDerr**
dan_daner_ANX_ANGST_mgs.	0	1.00	0.255	0.225(557)	0.225(779)	0.111	0.0971
dan_daner_ANX_ANGST_ntr.	0	1.00	0.414	0.218(1521)	0.218(2970)	0.0617	0.0755
dan_daner_ANX_ANGST_rs1.	0	1.00	0.43	0.215(331)	0.215(1629)	0.093	0.117
dan_daner_ANX_ANGST_rs2.	0	1.00	0.491	0.224(209)	0.224(1023)	−0.089	0.13
dan_daner_ANX_ANGST_rs3.	0	1.00	0.345	0.212(370)	0.212(1817)	0.0922	0.0976
dan_daner_ANX_ANGST_ship.	0	1.00	0.148	0.214(545)	0.214(834)	−0.144	0.0994
dan_daner_ANX_ANGST_trails.	0	0.95	0.304	0.230(278)	0.230(336)	−0.152	0.148
dan_daner_ANX_EstBB.	0	1.00	0.832	0.227(12523)	0.227(134954)	−0.0034	0.016
dan_daner_ANX_FinnGen.	0	1.00	0.015	0.237(9182)	0.237(160108)	0.0436	0.0179
dan_daner_ANX_GSC.	1	1.01	0.937	0.238(1306)	0.239(12675)	0.00387	0.049
dan_daner_ANX_Gedi_CHDS.	0	1.00	0.997	0.241(244)	0.241(217)	0.00052	0.156
dan_daner_ANX_Gedi_GSMS.	0	1.00	0.375	0.229(208)	0.229(551)	−0.127	0.143
dan_daner_ANX_Gedi_VTSABD.	0	1.00	0.772	0.225(224)	0.225(406)	0.00982	0.0339
dan_daner_ANX_HUNT.	1	1.00	0.000665	0.252(6587)	0.244(40740)	0.0759	0.0222
dan_daner_ANX_MGHI.	1	1.01	0.721	0.211(1823)	0.213(5151)	−0.0169	0.0471
dan_daner_ANX_MVP.	0	1.00	6.03e−05	0.232(28525)	0.232(163731)	0.0468	0.0117
dan_daner_ANX_MoBa.	0	1.00	0.228	0.237(715)	0.225(22168)	0.0778	0.0645
dan_daner_ANX_PANIC_dca1.	1	0.96	0.923	0.238(248)	0.236(970)	−0.0118	0.123
dan_daner_ANX_PANIC_gmdc.	1	0.99	0.763	0.213(247)	0.210(537)	0.0415	0.138
dan_daner_ANX_PANIC_gom3.	1	1.03	0.398	0.227(472)	0.216(1803)	0.0747	0.0883
dan_daner_ANX_PANIC_gwc3.	1	1.04	0.921	0.209(280)	0.199(855)	0.0124	0.126
dan_daner_ANX_PANIC_swdf.	1	1.01	0.323	0.229(561)	0.220(2591)	0.0797	0.0807
dan_daner_ANX_PBK1.	0	1.00	0.426	0.230(1121)	0.227(11897)	0.0421	0.0526
dan_daner_ANX_QIMR_AGDS.	0	1.00	0.108	0.230(7312)	0.230(12813)	0.055	0.0342
dan_daner_ANX_QIMR_Ad.	0	1.00	0.683	0.227(2449)	0.227(6343)	−0.0173	0.0424
dan_daner_ANX_QIMR_Nuup.	0	1.00	0.0516	0.228(1131)	0.228(1233)	0.144	0.0741
dan_daner_ANX_SWE_Catss.	0	1.00	0.453	0.220(540)	0.220(2333)	0.064	0.0853
dan_daner_ANX_SWE_Salty.	0	1.00	0.183	0.218(687)	0.218(3445)	−0.1	0.0753
dan_daner_ANX_SWE_TwinGene.	0	1.00	0.667	0.217(1740)	0.217(6470)	0.0201	0.0468
dan_daner_ANX_UKBB.	0	1.00	0.00498	0.226(25453)	0.226(58113)	0.0371	0.0132
dan_daner_ANX_Utah.	1	1.04	0.0735	0.240(258)	0.194(279)	0.262	0.147
dan_daner_ANX_alspac.	0	1.00	0.146	0.216(536)	0.239(3404)	−0.115	0.0797
dan_daner_ANX_bioVU.	0	1.00	0.258	0.228(7586)	0.228(50545)	0.0252	0.0223
dan_daner_ANX_iPSYCH.	22	0.99	0.00159	0.240(5288)	0.226(14422)	0.086	0.0272
dan_daner_ANX_musp.	1	1.04	0.167	0.215(316)	0.243(715)	−0.157	0.113
**dan_**	**32**	**1.00**	**4.31e−10**	**0.230(122225)**	**0.231(729960)**	**0.0351**	**0.0056**

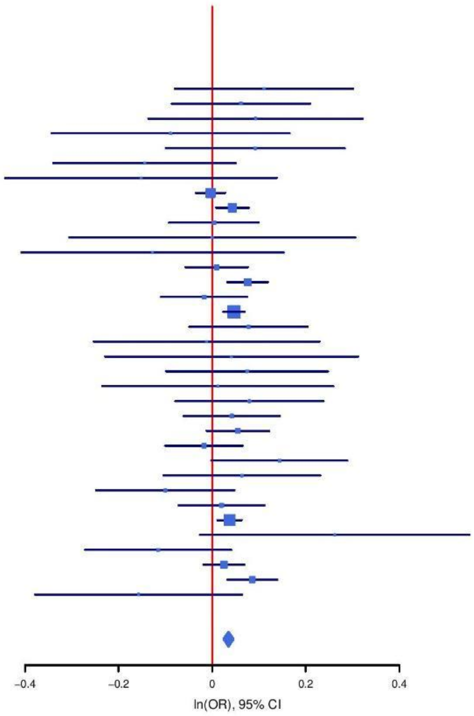

Supplementary Figure 20: Regional association plot (A) and forest plot (B) of SNP rs7570682. See full figure description on page 30.

A

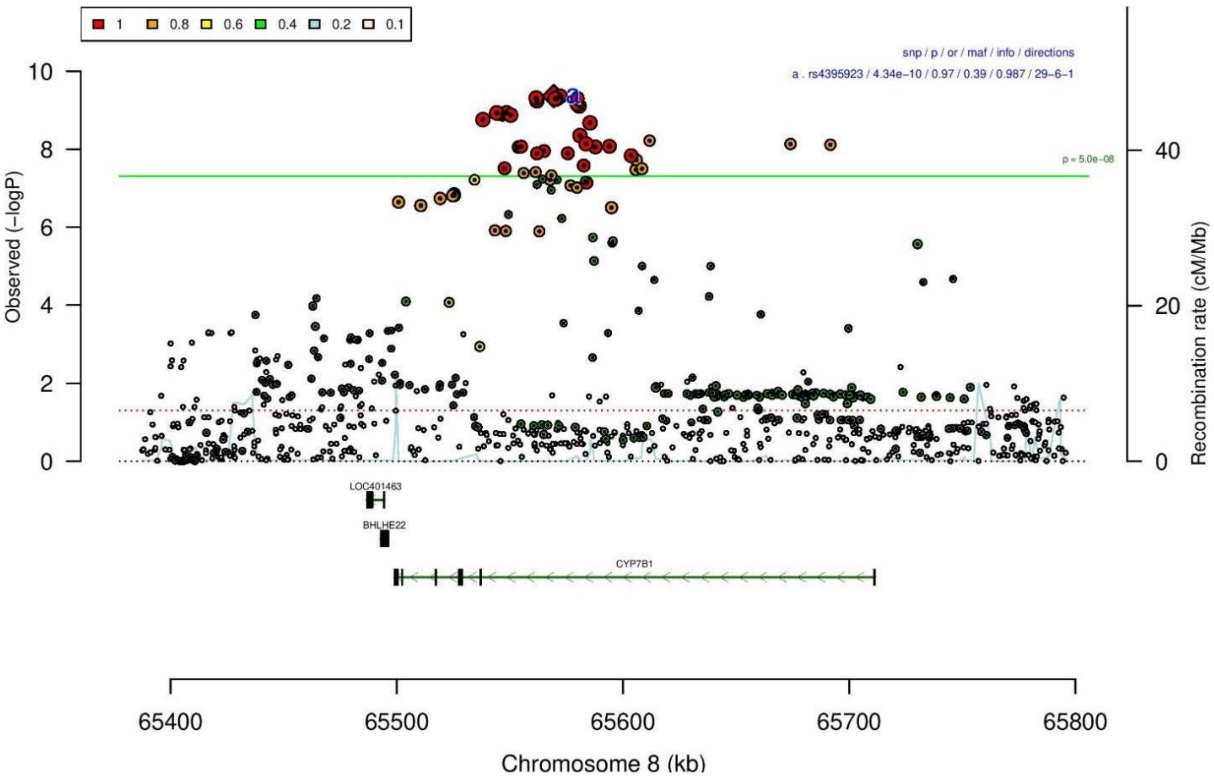

B

rs4395923		A/G		8:65569387			
−?++++++++++−−++++++−++++++++++−+++−				het_P:	0.623	het_I:	0.0
	ngt	info	p_value	f_ca(n)	f_co(n)	ln(OR)	STDerr
dan_daner_ANX_ANGST_mgs.	0	1.00	0.237	0.569(557)	0.569(779)	0.095	0.0802
dan_daner_ANX_ANGST_ntr.	0	1.00	0.556	0.586(1521)	0.586(2970)	0.0365	0.0621
dan_daner_ANX_ANGST_rs1.	0	1.00	0.623	0.590(331)	0.590(1629)	0.049	0.101
dan_daner_ANX_ANGST_rs2.	0	1.00	0.6	0.590(209)	0.590(1023)	0.059	0.112
dan_daner_ANX_ANGST_rs3.	0	1.00	0.79	0.592(370)	0.592(1817)	0.0221	0.0828
dan_daner_ANX_ANGST_ship.	0	1.00	0.913	0.611(545)	0.611(834)	0.00906	0.0828
dan_daner_ANX_ANGST_trails.	0	1.00	0.377	0.574(278)	0.574(336)	0.107	0.121
dan_daner_ANX_EstBB.	0	0.93	0.059	0.653(12523)	0.653(134954)	0.0275	0.0146
dan_daner_ANX_FinnGen.	0	1.00	0.0178	0.675(9182)	0.675(160108)	0.0392	0.0166
dan_daner_ANX_GSC.	1	0.99	0.958	0.570(1306)	0.564(12675)	0.00226	0.0426
dan_daner_ANX_Gedi_CHDS.	0	1.00	0.215	0.585(244)	0.585(217)	−0.175	0.141
dan_daner_ANX_Gedi_GSMS.	0	1.00	0.215	0.592(208)	0.592(551)	−0.15	0.121
dan_daner_ANX_Gedi_VTSABD.	0	1.00	0.745	0.571(224)	0.571(406)	0.00891	0.0274
dan_daner_ANX_HUNT.	0	1.00	0.000109	0.602(6587)	0.584(40740)	0.0763	0.0197
dan_daner_ANX_MGHI.	0	0.99	0.77	0.577(1823)	0.575(5151)	0.0115	0.0394
dan_daner_ANX_MVP.	0	1.00	0.0191	0.574(28525)	0.574(163731)	0.0226	0.0096
dan_daner_ANX_MoBa.	0	1.00	0.853	0.609(715)	0.607(22168)	0.0102	0.0549
dan_daner_ANX_PANIC_dca1.	0	1.02	0.464	0.614(248)	0.597(970)	0.0753	0.103
dan_daner_ANX_PANIC_gmdc.	1	0.98	0.116	0.624(247)	0.578(537)	0.182	0.116
dan_daner_ANX_PANIC_gom3.	0	0.99	0.912	0.596(472)	0.595(1803)	0.00846	0.0763
dan_daner_ANX_PANIC_gwc3.	1	1.00	0.223	0.636(280)	0.576(855)	0.132	0.109
dan_daner_ANX_PANIC_swdf.	0	0.97	0.304	0.629(561)	0.612(2591)	0.0733	0.0713
dan_daner_ANX_PBK1.	0	1.00	0.0842	0.597(1121)	0.581(11897)	0.0776	0.0449
dan_daner_ANX_QIMR_AGDS.	0	1.00	0.000857	0.573(7312)	0.573(12813)	0.0961	0.0288
dan_daner_ANX_QIMR_Ad.	0	1.00	0.21	0.568(2449)	0.568(6343)	0.0447	0.0356
dan_daner_ANX_QIMR_Nuup.	0	1.00	0.576	0.571(1131)	0.571(1233)	0.0345	0.0617
dan_daner_ANX_SWE_Catss.	0	1.00	0.67	0.376(540)	0.376(2333)	−0.031	0.0726
dan_daner_ANX_SWE_Salty.	0	1.00	0.199	0.376(687)	0.376(3445)	0.0806	0.0628
dan_daner_ANX_SWE_TwinGene.	0	1.00	0.855	0.384(1740)	0.384(6470)	0.00724	0.0396
dan_daner_ANX_UKBB.	0	1.00	0.0754	0.574(25453)	0.574(58113)	0.02	0.0112
dan_daner_ANX_Utah.	0	0.99	0.204	0.559(258)	0.593(279)	−0.16	0.126
dan_daner_ANX_alspac.	0	1.00	0.868	0.562(536)	0.563(3404)	−0.0112	0.0674
dan_daner_ANX_bioVU.	0	1.00	0.311	0.576(7586)	0.576(50545)	0.0191	0.0189
dan_daner_ANX_iPSYCH.	0	1.00	0.0688	0.590(5288)	0.579(14422)	0.0426	0.0234
dan_daner_ANX_musp.	0	0.99	0.282	0.562(316)	0.585(715)	−0.105	0.0977
**dan_**	**3**	**0.99**	**4.34e−10**	**0.589(122225)**	**0.609(729960)**	**0.0301**	**0.0048**

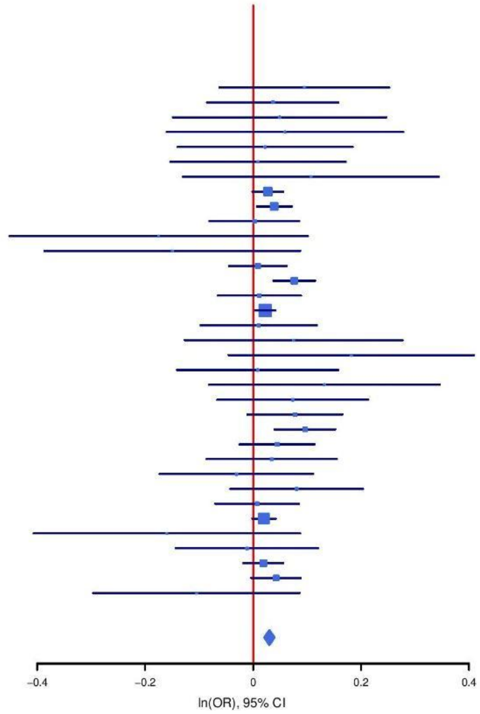

Supplementary Figure 21: Regional association plot (A) and forest plot (B) of SNP rs4395923. See full figure description on page 30.

A

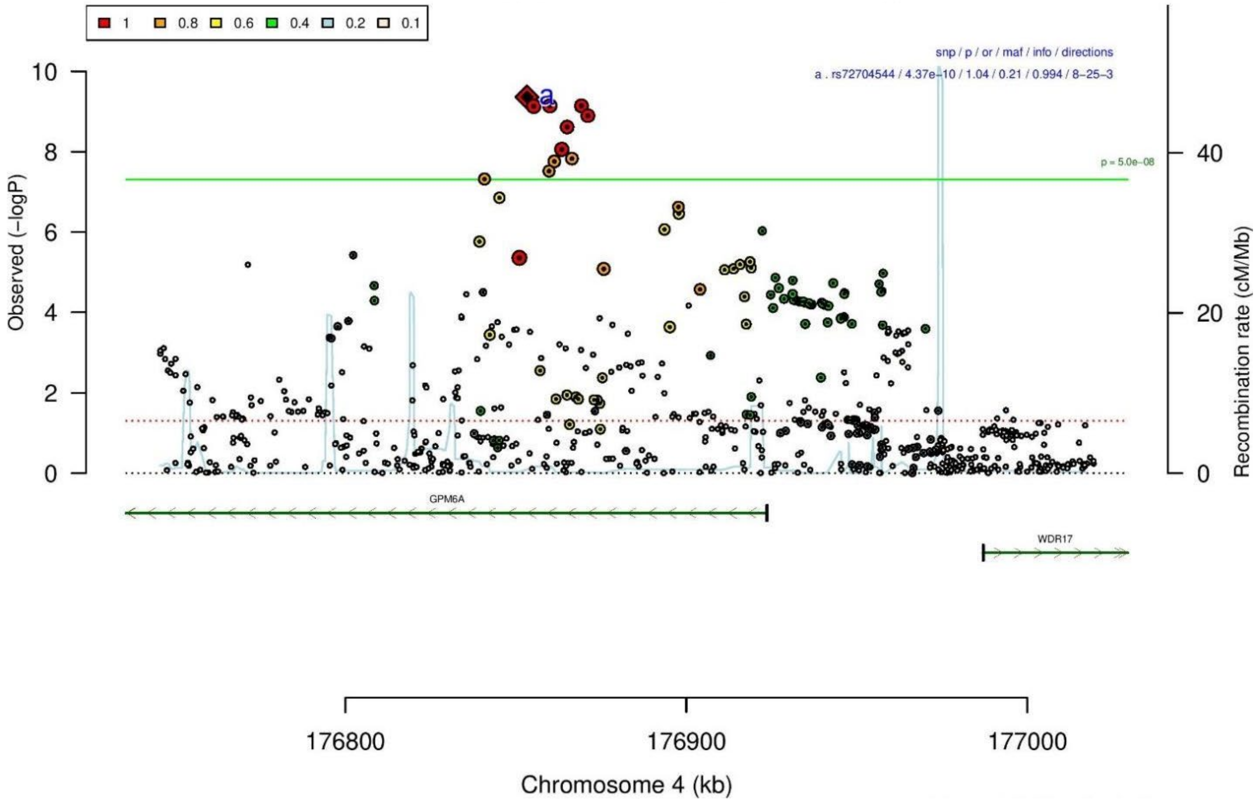

B

rs72704544		G/A		4:176853286			
-----+-+-???+--------+-----+-+--+---+				het_P:	0.104	het_I:	24.5
	ngt	info	p_value	f_ca(n)	f_co(n)	ln(OR)	STDerr
dan_daner_ANX_ANGST_gsk.	0	0.95	0.213	0.199(852)	0.199(1103)	0.108	0.087
dan_daner_ANX_ANGST_mgs.	0	1.00	0.276	0.205(557)	0.205(779)	0.108	0.0992
dan_daner_ANX_ANGST_ntr.	0	0.95	0.254	0.228(1521)	0.228(2970)	0.085	0.0745
dan_daner_ANX_ANGST_rs1.	0	0.95	0.659	0.217(331)	0.217(1629)	0.054	0.122
dan_daner_ANX_ANGST_rs2.	0	0.92	0.38	0.222(209)	0.222(1023)	−0.125	0.142
dan_daner_ANX_ANGST_rs3.	0	0.95	0.721	0.213(370)	0.213(1817)	0.036	0.101
dan_daner_ANX_ANGST_ship.	0	0.98	0.0479	0.214(545)	0.214(834)	−0.2	0.101
dan_daner_ANX_ANGST_trails.	0	0.88	0.724	0.200(278)	0.200(336)	0.0561	0.159
dan_daner_ANX_GSC.	0	0.99	0.278	0.220(1306)	0.212(12675)	0.0558	0.0514
dan_daner_ANX_Gedi_CHDS.	0	1.00	0.932	0.204(244)	0.204(217)	−0.015	0.175
dan_daner_ANX_Gedi_GSMS.	0	1.00	0.561	0.202(208)	0.202(551)	0.0856	0.147
dan_daner_ANX_Gedi_VTSABD.	0	1.00	0.737	0.223(224)	0.223(406)	0.0111	0.0329
dan_daner_ANX_HUNT.	0	0.99	0.0729	0.226(6587)	0.218(40740)	0.0419	0.0233
dan_daner_ANX_MGHI.	1	0.99	0.821	0.195(1823)	0.192(5151)	0.0112	0.0492
dan_daner_ANX_MVP.	0	1.00	0.0135	0.199(28525)	0.199(163731)	0.0293	0.0118
dan_daner_ANX_MoBa.	0	1.00	0.052	0.240(715)	0.219(22168)	0.127	0.0654
dan_daner_ANX_PANIC_dca1.	0	0.94	0.481	0.222(248)	0.208(970)	0.0896	0.127
dan_daner_ANX_PANIC_gmdc.	0	0.96	0.248	0.219(247)	0.197(537)	0.162	0.14
dan_daner_ANX_PANIC_gom3.	0	0.94	0.589	0.218(472)	0.209(1803)	0.0508	0.094
dan_daner_ANX_PANIC_gwc3.	0	1.00	0.00349	0.250(280)	0.200(855)	0.362	0.124
dan_daner_ANX_PANIC_swdf.	0	0.96	0.819	0.223(561)	0.233(2591)	−0.0189	0.0827
dan_daner_ANX_PBK1.	0	1.00	0.115	0.196(1121)	0.186(11897)	0.0889	0.0565
dan_daner_ANX_QIMR_AGDS.	0	0.97	0.328	0.201(7312)	0.201(12813)	0.0347	0.0354
dan_daner_ANX_QIMR_Ad.	0	0.97	0.247	0.202(2449)	0.202(6343)	−0.0518	0.0448
dan_daner_ANX_QIMR_Nuup.	0	0.97	0.0954	0.196(1131)	0.196(1233)	0.132	0.0789
dan_daner_ANX_SWE_Catss.	0	1.00	0.749	0.251(540)	0.251(2333)	−0.0258	0.0805
dan_daner_ANX_SWE_Salty.	0	1.00	0.0297	0.239(687)	0.239(3445)	0.15	0.069
dan_daner_ANX_SWE_TwinGene.	0	1.00	0.00847	0.239(1740)	0.239(6470)	0.119	0.0452
dan_daner_ANX_UKBB.	0	0.99	9.88e−06	0.206(25453)	0.206(58113)	0.0608	0.0138
dan_daner_ANX_Utah.	1	0.98	0.294	0.198(258)	0.224(279)	−0.161	0.153
dan_daner_ANX_alspac.	0	0.98	0.12	0.221(536)	0.202(3404)	0.128	0.0815
dan_daner_ANX_iPSYCH.	22	1.01	0.239	0.236(5288)	0.231(14422)	0.0319	0.0271
dan_daner_ANX_musp.	1	0.94	0.0951	0.199(316)	0.234(715)	−0.204	0.122
**dan_**	**25**	**0.99**	**4.37e−10**	**0.208(122225)**	**0.207(729960)**	**0.0423**	**0.0068**

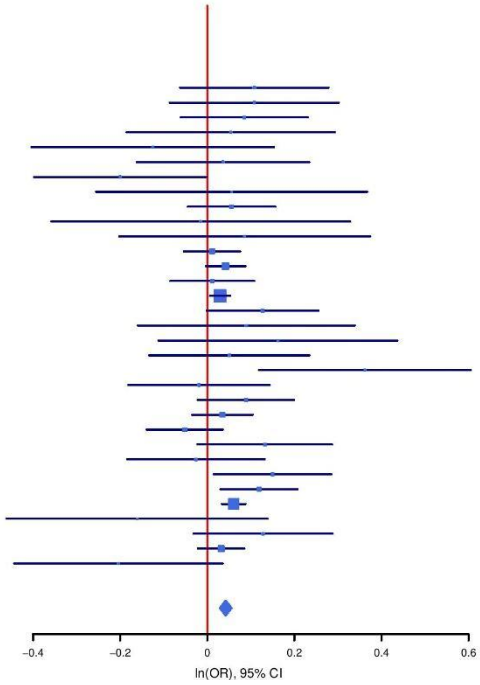

Supplementary Figure 22: Regional association plot (A) and forest plot (B) of SNP rs72704544. See full figure description on page 30.

A

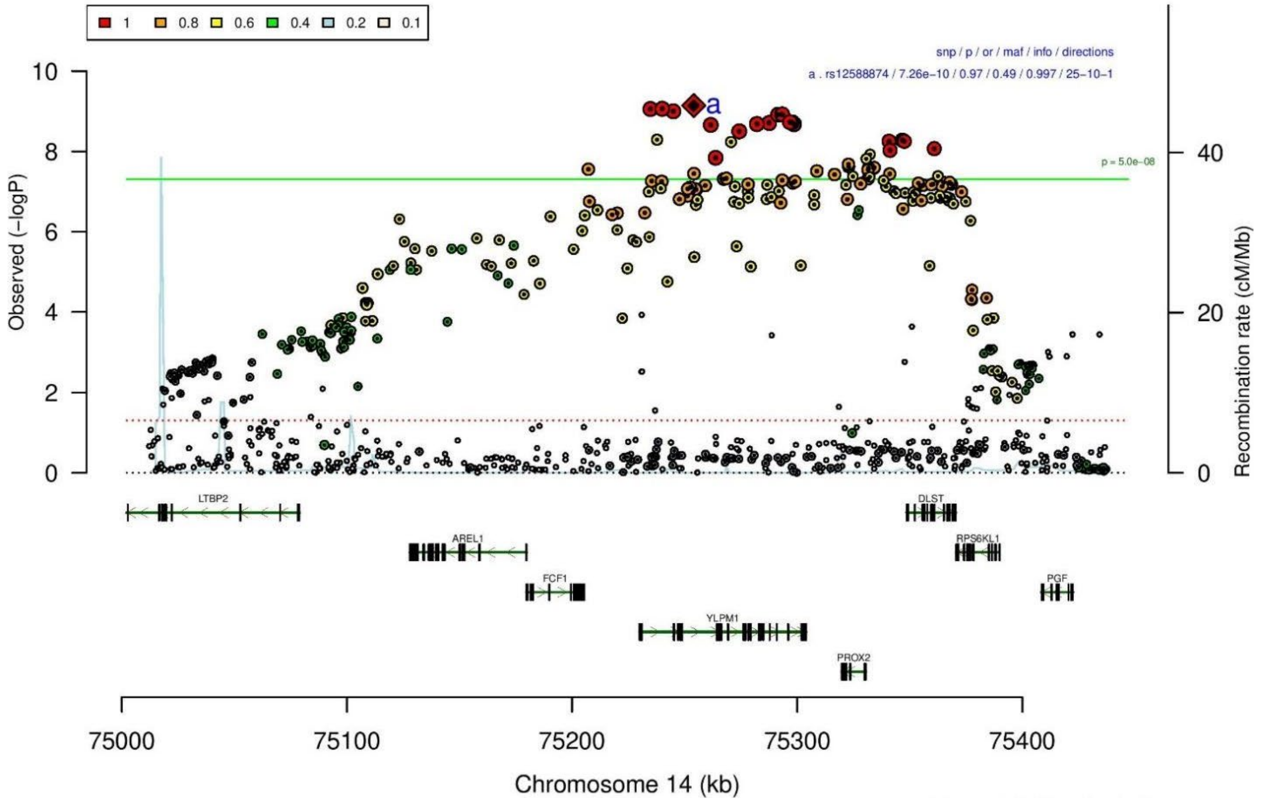

B

rs12588874		A/G		14:75254073			
−?+++−+−++++−−++++++−+++++++++−−+−+−				het_P:	0.0794	het_I:	26.4
	**ngt**	**info**	**p_value**	**f_ca(n)**	**f_co(n)**	**ln(OR)**	**STDerr**
dan_daner_ANX_ANGST_mgs.	0	1.00	0.415	0.559(557)	0.559(779)	0.0659	0.0808
dan_daner_ANX_ANGST_ntr.	0	1.00	0.337	0.514(1521)	0.514(2970)	0.059	0.0614
dan_daner_ANX_ANGST_rs1.	0	0.99	0.307	0.516(331)	0.516(1629)	0.1	0.098
dan_daner_ANX_ANGST_rs2.	0	0.99	0.846	0.519(209)	0.519(1023)	−0.022	0.111
dan_daner_ANX_ANGST_rs3.	0	1.00	0.244	0.517(370)	0.517(1817)	0.0936	0.0804
dan_daner_ANX_ANGST_ship.	0	1.00	0.0913	0.529(545)	0.529(834)	−0.136	0.0805
dan_daner_ANX_ANGST_trails.	0	0.99	0.0632	0.491(278)	0.491(336)	0.214	0.115
dan_daner_ANX_EstBB.	0	0.99	0.0742	0.510(12523)	0.510(134954)	0.0241	0.0135
dan_daner_ANX_FinnGen.	0	1.00	0.109	0.422(9182)	0.422(160108)	0.0249	0.0155
dan_daner_ANX_GSC.	0	1.00	0.24	0.578(1306)	0.561(12675)	0.05	0.0425
dan_daner_ANX_Gedi_CHDS.	0	1.00	0.196	0.534(244)	0.534(217)	−0.181	0.14
dan_daner_ANX_Gedi_GSMS.	0	1.00	0.149	0.563(208)	0.563(551)	−0.166	0.115
dan_daner_ANX_Gedi_VTSABD.	0	1.00	0.371	0.533(224)	0.533(406)	0.0238	0.0265
dan_daner_ANX_HUNT.	0	1.00	0.12	0.518(6587)	0.510(40740)	0.0301	0.0193
dan_daner_ANX_MGHI.	0	1.00	0.218	0.560(1823)	0.545(5151)	0.0479	0.0389
dan_daner_ANX_MVP.	0	1.00	0.197	0.544(28525)	0.544(163731)	0.0124	0.0096
dan_daner_ANX_MoBa.	0	1.00	0.0394	0.550(715)	0.524(22168)	0.111	0.0541
dan_daner_ANX_PANIC_dca1.	0	1.02	0.804	0.527(248)	0.520(970)	0.0251	0.101
dan_daner_ANX_PANIC_gmdc.	0	0.99	0.0365	0.562(247)	0.503(537)	0.238	0.114
dan_daner_ANX_PANIC_gom3.	0	1.01	0.56	0.534(472)	0.522(1803)	0.0433	0.0743
dan_daner_ANX_PANIC_gwc3.	0	1.01	0.0611	0.559(280)	0.527(855)	0.195	0.104
dan_daner_ANX_PANIC_swdf.	0	0.99	0.652	0.516(561)	0.509(2591)	0.0309	0.0686
dan_daner_ANX_PBK1.	0	1.00	0.503	0.563(1121)	0.554(11897)	0.0299	0.0447
dan_daner_ANX_QIMR_AGDS.	0	0.98	0.00198	0.537(7312)	0.537(12813)	0.0891	0.0288
dan_daner_ANX_QIMR_Ad.	0	0.98	0.0322	0.538(2449)	0.538(6343)	0.0778	0.0363
dan_daner_ANX_QIMR_Nuup.	0	0.98	0.199	0.539(1131)	0.539(1233)	−0.0801	0.0623
dan_daner_ANX_SWE_Catss.	0	1.00	0.315	0.495(540)	0.495(2333)	−0.0719	0.0716
dan_daner_ANX_SWE_Salty.	0	1.00	0.368	0.479(687)	0.479(3445)	0.054	0.0601
dan_daner_ANX_SWE_TwinGene.	0	1.00	0.303	0.492(1740)	0.492(6470)	−0.04	0.0388
dan_daner_ANX_UKBB.	0	1.00	4.46e−05	0.537(25453)	0.537(58113)	0.0454	0.0111
dan_daner_ANX_Utah.	0	1.02	0.788	0.549(258)	0.556(279)	−0.033	0.122
dan_daner_ANX_alspac.	0	1.00	0.519	0.532(536)	0.541(3404)	−0.0433	0.0672
dan_daner_ANX_bioVU.	0	1.00	0.324	0.547(7586)	0.547(50545)	0.0185	0.0188
dan_daner_ANX_iPSYCH.	0	0.99	0.0115	0.534(5288)	0.519(14422)	0.0585	0.0231
dan_daner_ANX_musp.	0	0.98	0.74	0.545(316)	0.553(715)	−0.0324	0.0975
**dan_**	**0**	**1.00**	**7.26e−10**	**0.526(122225)**	**0.506(729960)**	**0.029**	**0.0047**

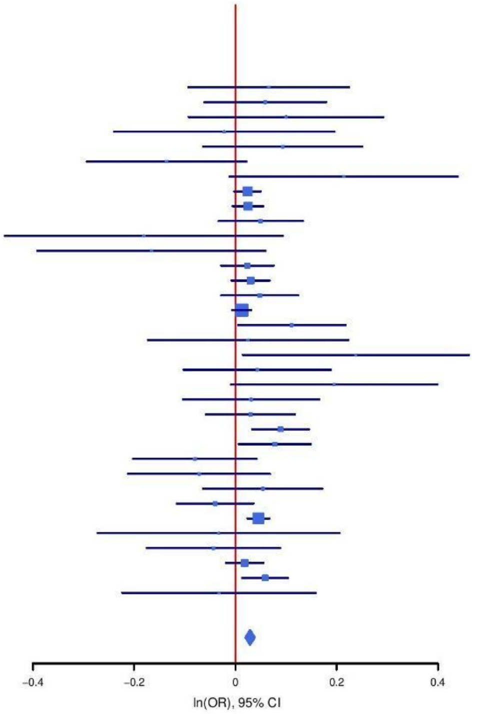

Supplementary Figure 23: Regional association plot (A) and forest plot (B) of SNP rs12588874. See full figure description on page 30.

A

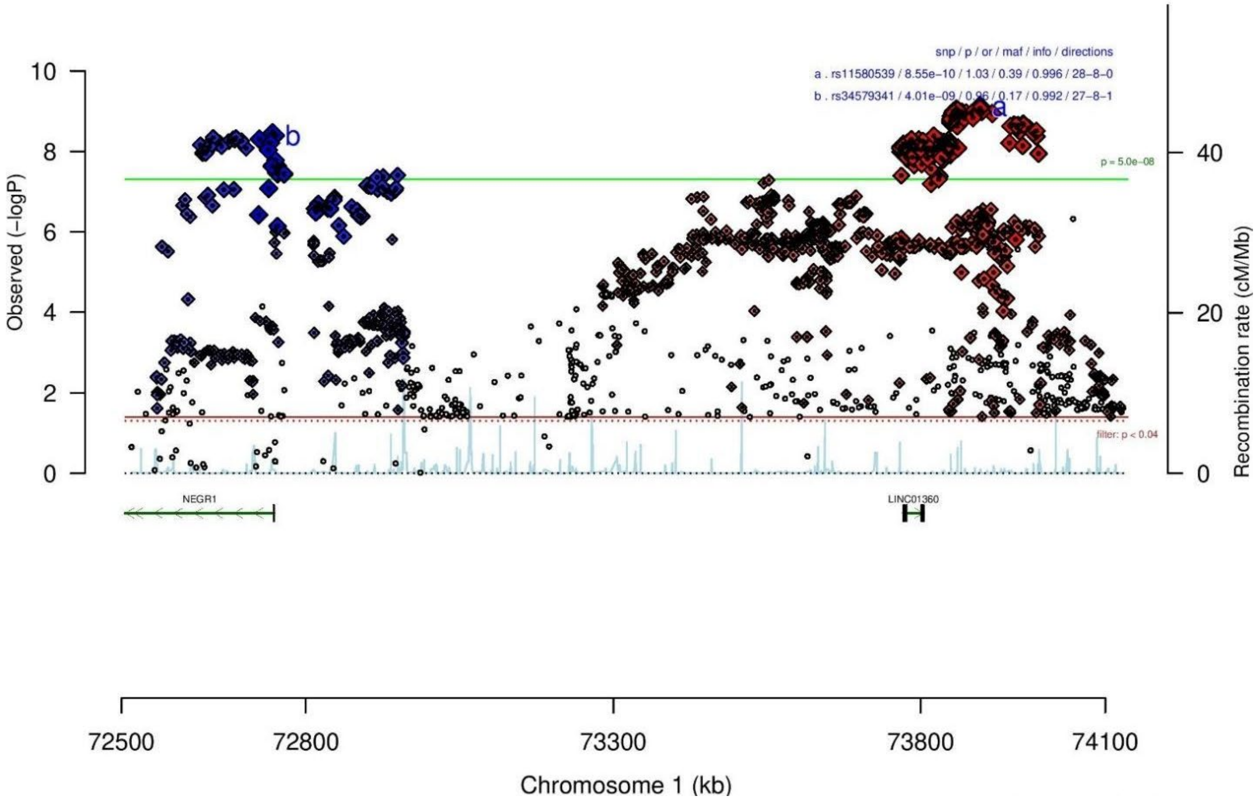

B

rs11580539 **G/A** **1:73896218**

+++++-+++++++--++++--++++---++++++++ het_P: 0.844 het_I: 0.0

	ngt	info	p_value	f_ca(n)	f_co(n)	ln(OR)	STDerr
dan_daner_ANX_ANGST_gsk.	0	1.00	0.864	0.543(852)	0.543(1103)	−0.0115	0.0672
dan_daner_ANX_ANGST_mgs.	0	1.00	0.393	0.597(557)	0.597(779)	−0.0707	0.0827
dan_daner_ANX_ANGST_ntr.	0	0.99	0.394	0.617(1521)	0.617(2970)	−0.0541	0.0634
dan_daner_ANX_ANGST_rs1.	0	0.99	0.877	0.600(331)	0.600(1629)	−0.016	0.103
dan_daner_ANX_ANGST_rs2.	0	0.99	0.918	0.616(209)	0.616(1023)	0.012	0.112
dan_daner_ANX_ANGST_rs3.	0	0.99	0.368	0.606(370)	0.606(1817)	−0.0751	0.0834
dan_daner_ANX_ANGST_ship.	0	1.00	0.0373	0.604(545)	0.604(834)	−0.173	0.0832
dan_daner_ANX_ANGST_trails.	0	0.97	0.113	0.597(278)	0.597(336)	−0.195	0.123
dan_daner_ANX_EstBB.	0	0.99	0.0424	0.635(12523)	0.635(134954)	−0.0283	0.014
dan_daner_ANX_FinnGen.	0	1.00	0.00589	0.629(9182)	0.629(160108)	−0.0435	0.0158
dan_daner_ANX_GSC.	0	0.99	0.0549	0.633(1306)	0.648(12675)	−0.085	0.0443
dan_daner_ANX_Gedi_CHDS.	0	1.00	0.479	0.648(244)	0.648(217)	−0.103	0.146
dan_daner_ANX_Gedi_GSMS.	0	1.00	0.891	0.580(208)	0.580(551)	0.0167	0.122
dan_daner_ANX_Gedi_VTSABD.	0	1.00	0.8	0.597(224)	0.597(406)	0.007	0.0276
dan_daner_ANX_HUNT.	0	1.00	0.00516	0.612(6587)	0.627(40740)	−0.0551	0.0197
dan_daner_ANX_MGHI.	0	0.99	0.118	0.563(1823)	0.575(5151)	−0.0617	0.0394
dan_daner_ANX_MVP.	0	1.00	0.00194	0.588(28525)	0.588(163731)	−0.0302	0.0097
dan_daner_ANX_MoBa.	0	1.00	0.986	0.618(715)	0.621(22168)	0.000948	0.0553
dan_daner_ANX_PANIC_dca1.	0	0.97	0.503	0.619(248)	0.633(970)	−0.0716	0.107
dan_daner_ANX_PANIC_gmdc.	0	1.03	0.324	0.574(247)	0.599(537)	−0.111	0.112
dan_daner_ANX_PANIC_gom3.	0	0.95	0.598	0.588(472)	0.595(1803)	−0.041	0.0778
dan_daner_ANX_PANIC_gwc3.	0	1.00	0.806	0.587(280)	0.578(855)	0.0259	0.105
dan_daner_ANX_PANIC_swdf.	0	0.98	0.673	0.619(561)	0.613(2591)	0.0298	0.0708
dan_daner_ANX_PBK1.	0	1.00	0.649	0.587(1121)	0.589(11897)	0.0206	0.0454
dan_daner_ANX_QIMR_AGDS.	0	0.96	0.0776	0.600(7312)	0.600(12813)	−0.0522	0.0296
dan_daner_ANX_QIMR_Ad.	0	0.96	0.246	0.605(2449)	0.605(6343)	−0.0434	0.0374
dan_daner_ANX_QIMR_Nuup.	0	0.96	0.827	0.599(1131)	0.599(1233)	−0.0144	0.0658
dan_daner_ANX_SWE_Catss.	0	1.00	0.548	0.390(540)	0.390(2333)	−0.0434	0.0723
dan_daner_ANX_SWE_Salty.	0	1.00	0.193	0.397(687)	0.397(3445)	−0.08	0.0614
dan_daner_ANX_SWE_TwinGene.	0	1.00	0.211	0.396(1740)	0.396(6470)	−0.0486	0.0388
dan_daner_ANX_UKBB.	0	1.00	0.458	0.620(25453)	0.620(58113)	−0.00849	0.0114
dan_daner_ANX_Utah.	0	1.02	0.679	0.589(258)	0.598(279)	−0.0513	0.124
dan_daner_ANX_alspac.	0	1.00	0.479	0.606(536)	0.616(3404)	−0.0483	0.068
dan_daner_ANX_bioVU.	0	1.00	0.962	0.586(7586)	0.586(50545)	−0.000896	0.0189
dan_daner_ANX_iPSYCH.	0	1.00	0.0099	0.595(5288)	0.611(14422)	−0.0607	0.0235
dan_daner_ANX_musp.	0	0.95	0.33	0.646(316)	0.623(715)	0.1	0.103
**dan_**	**0**	**1.00**	**8.55e−10**	**0.602(122225)**	**0.610(729960)**	**−0.0295**	**0.0048**

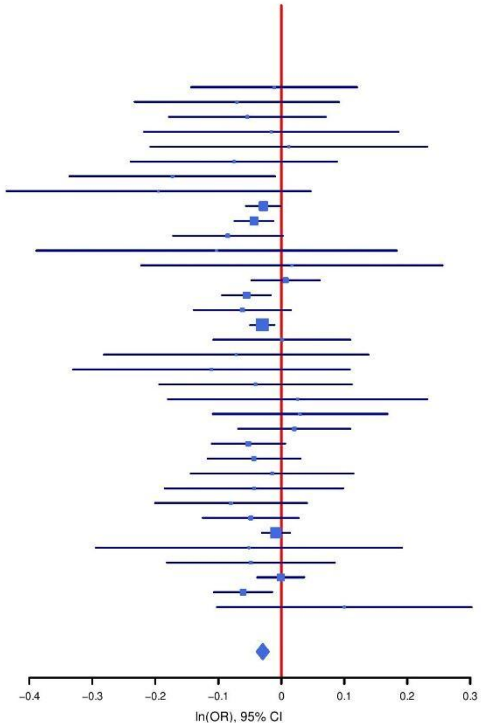

C

rs34579341 G/A 1:72745962

+?++++++-+++-+++++++-++++--+++--+++- het_P: 0.914 het_I: 0.0

	ngt	info	p_value	f_ca(n)	f_co(n)	ln(OR)	STDerr
dan_daner_ANX_ANGST_mgs.	0	1.00	0.27	0.181(557)	0.181(779)	−0.113	0.102
dan_daner_ANX_ANGST_ntr.	0	0.97	0.0888	0.183(1521)	0.183(2970)	−0.14	0.0823
dan_daner_ANX_ANGST_rs1.	0	0.99	0.719	0.179(331)	0.179(1629)	−0.047	0.131
dan_daner_ANX_ANGST_rs2.	0	0.99	0.0228	0.198(209)	0.198(1023)	−0.337	0.148
dan_daner_ANX_ANGST_rs3.	0	0.99	0.0497	0.187(370)	0.187(1817)	−0.212	0.108
dan_daner_ANX_ANGST_ship.	0	0.99	0.978	0.163(545)	0.163(834)	−0.00311	0.113
dan_daner_ANX_ANGST_trails.	0	0.99	0.425	0.189(278)	0.189(336)	0.122	0.153
dan_daner_ANX_EstBB.	0	0.97	0.0954	0.152(12523)	0.152(134954)	−0.0315	0.0189
dan_daner_ANX_FinnGen.	0	1.00	0.205	0.154(9182)	0.154(160108)	−0.0271	0.0214
dan_daner_ANX_GSC.	0	1.00	0.252	0.215(1306)	0.224(12675)	−0.0575	0.0502
dan_daner_ANX_Gedi_CHDS.	0	1.00	0.519	0.205(244)	0.205(217)	0.109	0.169
dan_daner_ANX_Gedi_GSMS.	0	1.00	0.941	0.175(208)	0.175(551)	−0.0115	0.154
dan_daner_ANX_Gedi_VTSABD.	0	1.00	0.973	0.193(224)	0.193(406)	−0.00114	0.0342
dan_daner_ANX_HUNT.	0	1.00	0.0612	0.192(6587)	0.202(40740)	−0.0451	0.0241
dan_daner_ANX_MGHI.	0	1.01	0.761	0.175(1823)	0.174(5151)	−0.0156	0.0512
dan_daner_ANX_MVP.	0	1.00	0.00117	0.173(28525)	0.173(163731)	−0.042	0.0129
dan_daner_ANX_MoBa.	0	1.00	0.297	0.182(715)	0.193(22168)	−0.0713	0.0683
dan_daner_ANX_PANIC_dca1.	0	1.02	0.563	0.177(248)	0.191(970)	−0.0757	0.131
dan_daner_ANX_PANIC_gmdc.	0	0.97	0.114	0.133(247)	0.169(537)	−0.255	0.161
dan_daner_ANX_PANIC_gom3.	0	0.97	0.254	0.148(472)	0.166(1803)	−0.12	0.105
dan_daner_ANX_PANIC_gwc3.	0	1.01	0.857	0.170(280)	0.166(855)	0.0248	0.138
dan_daner_ANX_PANIC_swdf.	0	1.01	0.699	0.188(561)	0.184(2591)	0.0333	0.0863
dan_daner_ANX_PBK1.	0	1.00	0.149	0.169(1121)	0.184(11897)	−0.0859	0.0596
dan_daner_ANX_QIMR_AGDS.	0	0.97	0.41	0.191(7312)	0.191(12813)	−0.0301	0.0365
dan_daner_ANX_QIMR_Ad.	0	0.97	0.127	0.196(2449)	0.196(6343)	−0.0691	0.0452
dan_daner_ANX_QIMR_Nuup.	0	0.97	0.678	0.192(1131)	0.192(1233)	0.0325	0.0781
dan_daner_ANX_SWE_Catss.	0	1.00	0.736	0.188(540)	0.188(2333)	0.0299	0.0887
dan_daner_ANX_SWE_Salty.	0	1.00	0.587	0.187(687)	0.187(3445)	−0.0422	0.0777
dan_daner_ANX_SWE_TwinGene.	0	1.00	0.118	0.188(1740)	0.188(6470)	−0.0788	0.0504
dan_daner_ANX_UKBB.	0	0.99	0.0433	0.199(25453)	0.199(58113)	−0.0283	0.014
dan_daner_ANX_Utah.	0	1.08	0.333	0.184(258)	0.162(279)	0.152	0.157
dan_daner_ANX_alspac.	0	1.00	0.952	0.197(536)	0.196(3404)	−0.00508	0.0845
dan_daner_ANX_bioVU.	0	1.00	0.0996	0.178(7586)	0.178(50545)	−0.0402	0.0244
dan_daner_ANX_iPSYCH.	0	0.98	0.212	0.172(5288)	0.179(14422)	−0.0382	0.0306
dan_daner_ANX_musp.	0	0.99	0.324	0.218(316)	0.198(715)	0.117	0.118
**dan_**	**0**	**0.99**	**4.01e−09**	**0.179(122225)**	**0.172(729960)**	**−0.0365**	**0.0062**

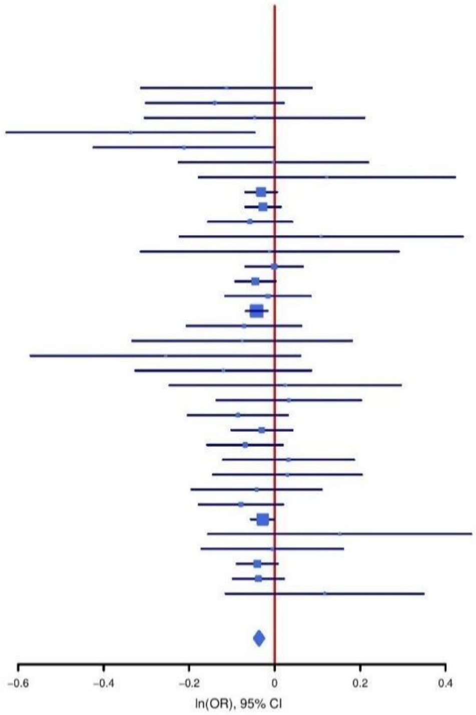

Supplementary Figure 24: Regional association plot (A) and forest plots (B, C) of SNP rs11580539 and rs34579341. See full figure description on page 30.

A

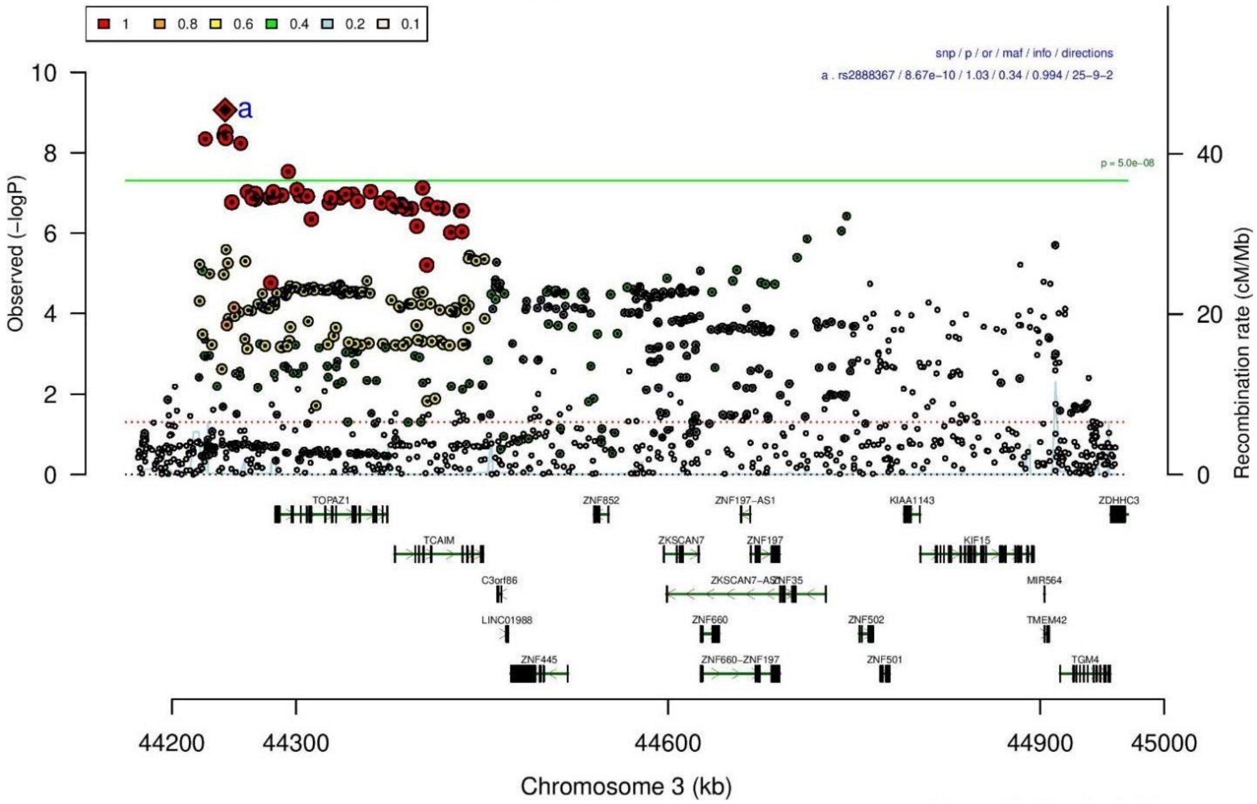

B

rs2888367 A/G 3:44242929

+?−++−−−+?++++−++++−−+++−+−+++++++++ het_P: 0.11 het_I: 23.6

	ngt	info	p_value	f_ca(n)	f_co(n)	ln(OR)	STDerr
dan_daner_ANX_ANGST_mgs.	0	1.00	0.101	0.335(557)	0.335(779)	−0.139	0.0845
dan_daner_ANX_ANGST_ntr.	0	1.00	0.0233	0.337(1521)	0.337(2970)	0.148	0.0651
dan_daner_ANX_ANGST_rs1.	0	0.99	0.232	0.340(331)	0.340(1629)	0.123	0.103
dan_daner_ANX_ANGST_rs2.	0	0.99	0.247	0.338(209)	0.338(1023)	−0.137	0.118
dan_daner_ANX_ANGST_rs3.	0	0.98	0.517	0.335(370)	0.335(1817)	−0.0552	0.0853
dan_daner_ANX_ANGST_ship.	0	1.00	0.892	0.331(545)	0.331(834)	−0.0116	0.0856
dan_daner_ANX_ANGST_trails.	0	0.95	0.279	0.328(278)	0.328(336)	0.144	0.133
dan_daner_ANX_EstBB.	0	1.00	0.0686	0.367(12523)	0.367(134954)	0.0253	0.0139
dan_daner_ANX_FinnGen.	0	0.99	0.00277	0.342(9182)	0.342(160108)	0.0482	0.0161
dan_daner_ANX_GSC.	0	1.01	0.183	0.316(1306)	0.306(12675)	0.0603	0.0453
dan_daner_ANX_Gedi_CHDS.	0	1.00	0.627	0.334(244)	0.334(217)	0.0696	0.143
dan_daner_ANX_Gedi_GSMS.	0	1.00	0.444	0.323(208)	0.323(551)	0.0981	0.128
dan_daner_ANX_Gedi_VTSABD.	0	1.00	0.43	0.333(224)	0.333(406)	−0.023	0.0291
dan_daner_ANX_HUNT.	0	0.98	0.491	0.303(6587)	0.296(40740)	0.0146	0.0212
dan_daner_ANX_MGHI.	0	0.95	0.52	0.334(1823)	0.330(5151)	0.027	0.042
dan_daner_ANX_MVP.	0	1.00	9.91e−06	0.330(28525)	0.330(163731)	0.0443	0.01
dan_daner_ANX_MoBa.	0	1.00	0.537	0.307(715)	0.315(22168)	−0.036	0.0583
dan_daner_ANX_PANIC_dca1.	0	1.01	0.684	0.323(248)	0.311(970)	0.0438	0.108
dan_daner_ANX_PANIC_gmdc.	0	0.97	0.665	0.316(247)	0.315(537)	0.0535	0.123
dan_daner_ANX_PANIC_gom3.	0	0.99	0.277	0.335(472)	0.360(1803)	−0.086	0.0792
dan_daner_ANX_PANIC_gwc3.	0	0.91	0.13	0.379(280)	0.326(855)	0.173	0.114
dan_daner_ANX_PANIC_swdf.	0	0.94	0.64	0.327(561)	0.327(2591)	−0.0348	0.0744
dan_daner_ANX_PBK1.	0	1.00	0.0808	0.341(1121)	0.324(11897)	0.0816	0.0467
dan_daner_ANX_QIMR_AGDS.	0	0.95	0.683	0.313(7312)	0.313(12813)	0.0128	0.0314
dan_daner_ANX_QIMR_Ad.	0	0.95	0.00638	0.310(2449)	0.310(6343)	0.106	0.0388
dan_daner_ANX_QIMR_Nuup.	0	0.95	0.781	0.326(1131)	0.326(1233)	0.0188	0.0678
dan_daner_ANX_SWE_Catss.	0	1.00	0.27	0.323(540)	0.323(2333)	0.0823	0.0746
dan_daner_ANX_SWE_Salty.	0	1.00	0.4	0.332(687)	0.332(3445)	0.0543	0.0644
dan_daner_ANX_SWE_TwinGene.	0	1.00	0.0694	0.333(1740)	0.333(6470)	0.0734	0.0404
dan_daner_ANX_UKBB.	0	1.00	0.367	0.316(25453)	0.316(58113)	0.0108	0.0119
dan_daner_ANX_Utah.	0	1.04	0.701	0.312(258)	0.299(279)	0.0503	0.131
dan_daner_ANX_alspac.	0	0.97	0.00301	0.356(536)	0.310(3404)	0.209	0.0699
dan_daner_ANX_iPSYCH.	0	0.99	0.186	0.329(5288)	0.322(14422)	0.0327	0.0247
dan_daner_ANX_musp.	0	0.88	0.451	0.295(316)	0.310(715)	−0.0841	0.112
**dan_**	**0**	**0.99**	**8.67e−10**	**0.329(122225)**	**0.335(729960)**	**0.0316**	**0.0051**

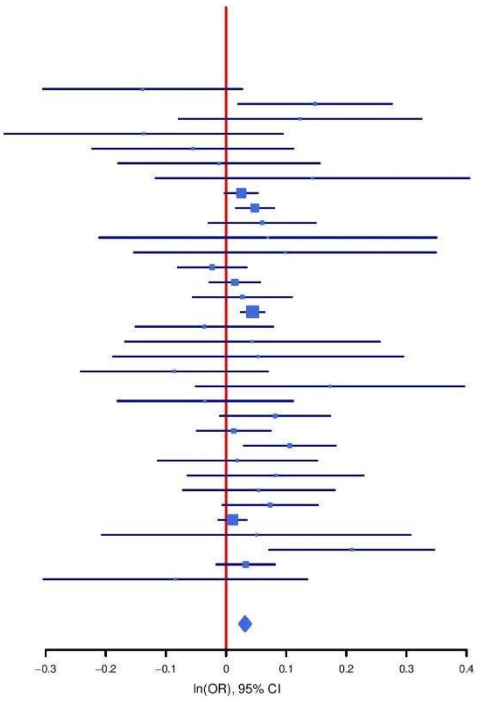

Supplementary Figure 25: Regional association plot (A) and forest plot (B) of SNP rs2888367. See full figure description on page 30.

A

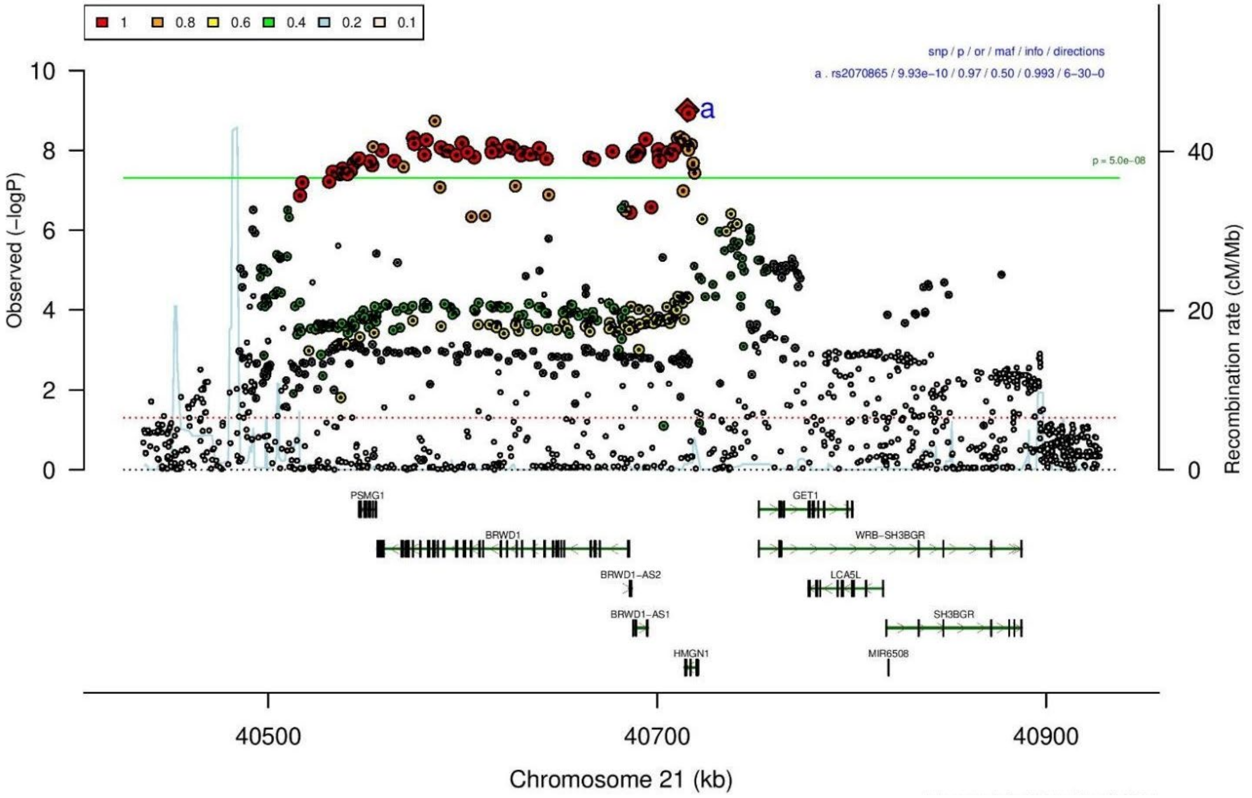

B

rs2070865		T/C		21:40715519			
----+-+------+-+-----------+-----+-----				het_P:	0.643	het_I:	0.0
	ngt	info	p_value	f_ca(n)	f_co(n)	ln(OR)	STDerr
dan_daner_ANX_ANGST_gsk.	0	0.94	0.659	0.452(852)	0.452(1103)	−0.0308	0.0697
dan_daner_ANX_ANGST_mgs.	0	1.00	0.489	0.468(557)	0.468(779)	−0.0572	0.0827
dan_daner_ANX_ANGST_ntr.	0	1.00	0.795	0.479(1521)	0.479(2970)	−0.0158	0.061
dan_daner_ANX_ANGST_rs1.	0	1.00	0.752	0.470(331)	0.470(1629)	0.031	0.098
dan_daner_ANX_ANGST_rs2.	0	1.00	0.59	0.466(209)	0.466(1023)	−0.059	0.109
dan_daner_ANX_ANGST_rs3.	0	1.00	0.093	0.470(370)	0.470(1817)	0.139	0.0829
dan_daner_ANX_ANGST_ship.	0	0.99	0.211	0.475(545)	0.475(834)	−0.102	0.0817
dan_daner_ANX_ANGST_trails.	0	0.94	0.891	0.446(278)	0.446(336)	−0.0181	0.132
dan_daner_ANX_EstBB.	0	0.98	0.0266	0.529(12523)	0.529(134954)	−0.03	0.0135
dan_daner_ANX_FinnGen.	0	1.00	0.0822	0.568(9182)	0.568(160108)	−0.0269	0.0155
dan_daner_ANX_GSC.	1	0.99	0.476	0.444(1306)	0.451(12675)	−0.0302	0.0424
dan_daner_ANX_Gedi_CHDS.	0	1.00	0.608	0.495(244)	0.495(217)	0.0726	0.142
dan_daner_ANX_Gedi_GSMS.	0	1.00	0.912	0.439(208)	0.439(551)	−0.0134	0.12
dan_daner_ANX_Gedi_VTSABD.	0	1.00	0.918	0.450(224)	0.450(406)	0.00278	0.0269
dan_daner_ANX_HUNT.	1	1.00	0.738	0.489(6587)	0.496(40740)	−0.00643	0.0192
dan_daner_ANX_MGHI.	1	1.00	0.729	0.453(1823)	0.456(5151)	−0.0134	0.0389
dan_daner_ANX_MVP.	0	1.00	0.000227	0.457(28525)	0.457(163731)	−0.0359	0.0097
dan_daner_ANX_MoBa.	0	1.00	0.349	0.459(715)	0.468(22168)	−0.0506	0.054
dan_daner_ANX_PANIC_dca1.	1	1.05	0.175	0.454(248)	0.489(970)	−0.135	0.0995
dan_daner_ANX_PANIC_gmdc.	1	1.04	0.565	0.427(247)	0.445(537)	−0.0638	0.111
dan_daner_ANX_PANIC_gom3.	1	1.01	0.875	0.462(472)	0.459(1803)	0.0116	0.0743
dan_daner_ANX_PANIC_gwc3.	1	0.99	0.421	0.445(280)	0.470(855)	−0.0841	0.105
dan_daner_ANX_PANIC_swdf.	1	1.00	0.331	0.470(561)	0.478(2591)	−0.0664	0.0682
dan_daner_ANX_PBK1.	0	1.00	0.309	0.449(1121)	0.459(11897)	−0.0451	0.0444
dan_daner_ANX_QIMR_AGDS.	0	0.92	0.0297	0.436(7312)	0.436(12813)	−0.0653	0.03
dan_daner_ANX_QIMR_Ad.	0	0.92	0.92	0.449(2449)	0.449(6343)	−0.00355	0.0356
dan_daner_ANX_QIMR_Nuup.	0	0.92	0.0117	0.468(1131)	0.468(1233)	0.154	0.0611
dan_daner_ANX_SWE_Catss.	0	1.00	0.125	0.477(540)	0.477(2333)	−0.108	0.0706
dan_daner_ANX_SWE_Salty.	0	1.00	0.611	0.486(687)	0.486(3445)	−0.0308	0.0604
dan_daner_ANX_SWE_TwinGene.	0	1.00	0.848	0.485(1740)	0.485(6470)	−0.00742	0.0387
dan_daner_ANX_UKBB.	0	1.00	0.00718	0.451(25453)	0.451(58113)	−0.0301	0.0112
dan_daner_ANX_Utah.	1	1.00	0.136	0.438(258)	0.484(279)	−0.184	0.123
dan_daner_ANX_alspac.	0	1.00	0.00844	0.418(536)	0.461(3404)	−0.179	0.0682
dan_daner_ANX_bioVU.	0	1.00	0.258	0.457(7586)	0.457(50545)	−0.0211	0.0187
dan_daner_ANX_iPSYCH.	22	1.00	0.0756	0.462(5288)	0.472(14422)	−0.0411	0.0231
dan_daner_ANX_musp.	1	0.98	0.577	0.446(316)	0.462(715)	−0.0545	0.0976
**dan_**	**32**	**0.99**	**9.93e−10**	**0.473(122225)**	**0.497(729960)**	**−0.0289**	**0.0047**

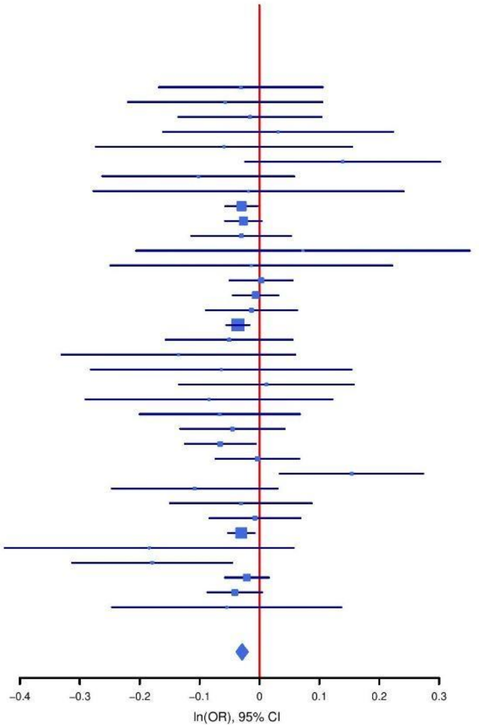

Supplementary Figure 26: Regional association plot (A) and forest plot (B) of SNP rs2070865. See full figure description on page 30.

A

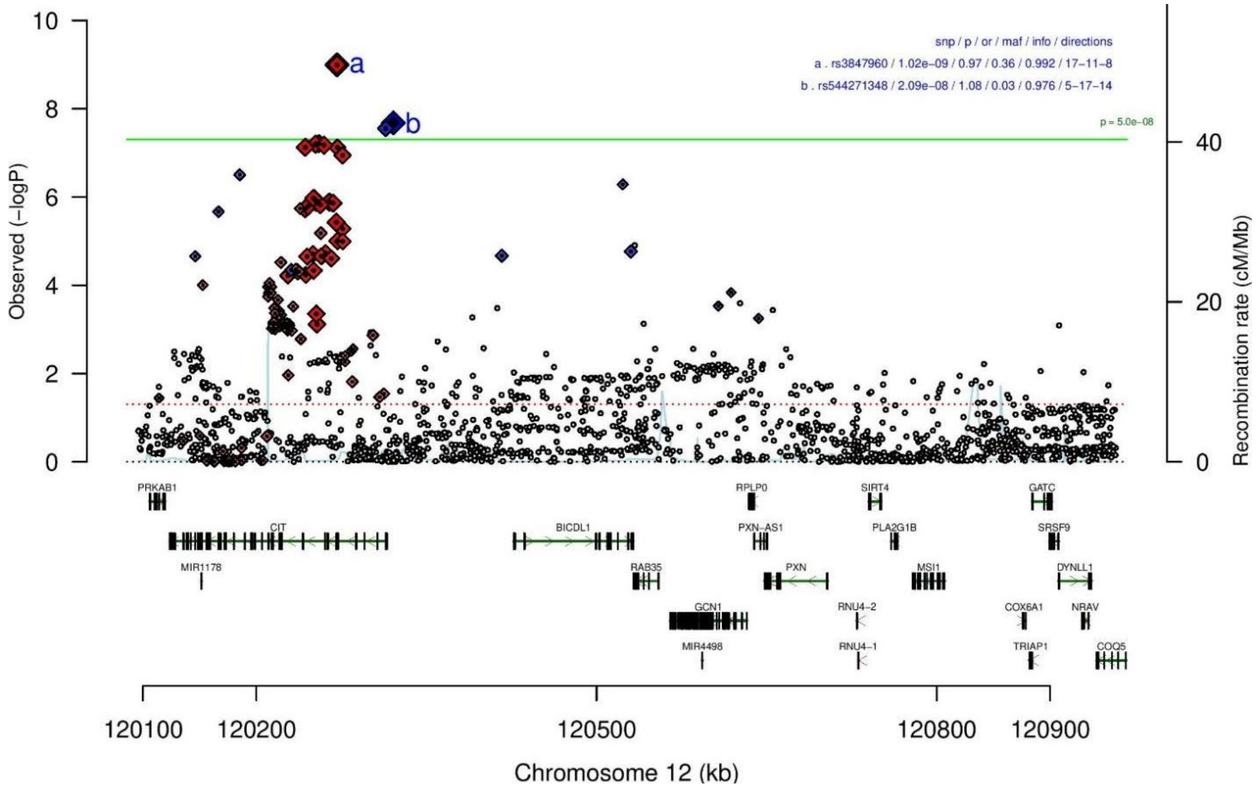

B

rs3847960		A/T		12:120271100			
−?−−−+++−?++−++−++++−+?++−−?+++????−				het_P:	0.335	het_I:	8.6
	ngt	info	p_value	f_ca(n)	f_co(n)	ln(OR)	STDerr
dan_daner_ANX_ANGST_mgs.	0	1.00	0.951	0.624(557)	0.624(779)	−0.0052	0.0848
dan_daner_ANX_ANGST_ntr.	0	0.94	0.826	0.657(1521)	0.657(2970)	−0.0147	0.0668
dan_daner_ANX_ANGST_rs1.	0	0.98	0.993	0.640(331)	0.640(1629)	−0.001	0.102
dan_daner_ANX_ANGST_rs2.	0	0.98	0.358	0.638(209)	0.638(1023)	0.109	0.118
dan_daner_ANX_ANGST_rs3.	0	0.99	0.032	0.638(370)	0.638(1817)	0.184	0.086
dan_daner_ANX_ANGST_ship.	0	1.00	0.615	0.605(545)	0.605(834)	0.042	0.0834
dan_daner_ANX_ANGST_trails.	0	0.99	0.749	0.653(278)	0.653(336)	−0.0401	0.126
dan_daner_ANX_EstBB.	0	0.98	0.00458	0.612(12523)	0.612(134954)	0.0396	0.014
dan_daner_ANX_FinnGen.	0	1.00	0.0466	0.670(9182)	0.670(160108)	0.0327	0.0164
dan_daner_ANX_GSC.	0	0.99	0.362	0.624(1306)	0.631(12675)	−0.0399	0.0437
dan_daner_ANX_Gedi_CHDS.	0	1.00	0.65	0.634(244)	0.634(217)	−0.0637	0.14
dan_daner_ANX_Gedi_GSMS.	0	1.00	0.521	0.647(208)	0.647(551)	0.0815	0.127
dan_daner_ANX_Gedi_VTSABD.	0	1.00	0.804	0.612(224)	0.612(406)	0.00711	0.0287
dan_daner_ANX_HUNT.	0	0.99	0.0881	0.644(6587)	0.635(40740)	0.0342	0.0201
dan_daner_ANX_MGHI.	0	1.00	0.964	0.630(1823)	0.630(5151)	0.0018	0.0402
dan_daner_ANX_MVP.	0	1.00	0.00011	0.627(28525)	0.627(163731)	0.0383	0.0099
dan_daner_ANX_MoBa.	0	1.00	0.128	0.649(715)	0.629(22168)	0.0843	0.0554
dan_daner_ANX_PANIC_gmdc.	0	0.96	0.914	0.605(247)	0.602(537)	0.0125	0.116
dan_daner_ANX_PANIC_gom3.	0	1.02	0.971	0.605(472)	0.604(1803)	0.0028	0.0757
dan_daner_ANX_PANIC_gwc3.	0	0.98	0.807	0.602(280)	0.606(855)	−0.0262	0.107
dan_daner_ANX_PANIC_swdf.	0	0.99	0.158	0.619(561)	0.637(2591)	−0.0996	0.0706
dan_daner_ANX_QIMR_AGDS.	0	0.96	0.0918	0.620(7312)	0.620(12813)	0.0504	0.0299
dan_daner_ANX_QIMR_Ad.	0	0.96	0.00703	0.608(2449)	0.608(6343)	0.0995	0.0369
dan_daner_ANX_QIMR_Nuup.	0	0.96	0.396	0.613(1131)	0.613(1233)	0.0554	0.0654
dan_daner_ANX_Utah.	0	0.98	0.0506	0.608(258)	0.666(279)	−0.254	0.13
dan_daner_ANX_alspac.	0	0.99	0.671	0.604(536)	0.613(3404)	−0.0291	0.0684
dan_daner_ANX_iPSYCH.	0	1.00	0.00404	0.643(5288)	0.627(14422)	0.0687	0.0239
dan_daner_ANX_musp.	0	0.94	0.144	0.616(316)	0.648(715)	−0.149	0.102
**dan_**	**0**	**0.99**	**1.02e−09**	**0.631(122225)**	**0.636(729960)**	**0.0353**	**0.0058**

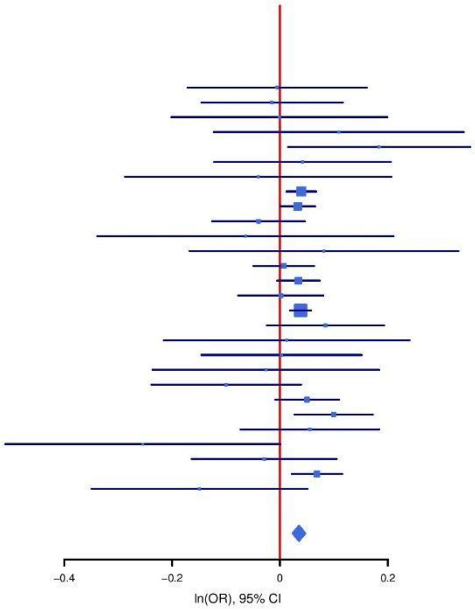

C

rs544271348 G/T 12:120320793

?????????------+-----+-?????-+--++--- het_P: 0.773 het_I: 0.0

	ngt	info	p_value	f_ca(n)	f_co(n)	ln(OR)	STDerr
dan_daner_ANX_EstBB.	0	0.99	0.0157	0.041(12523)	0.041(134954)	0.0799	0.0331
dan_daner_ANX_FinnGen.	0	0.96	0.176	0.021(9182)	0.021(160108)	0.071	0.0524
dan_daner_ANX_GSC.	0	0.89	0.68	0.052(1306)	0.054(12675)	−0.0408	0.0989
dan_daner_ANX_Gedi_CHDS.	0	1.00	0.215	0.046(244)	0.046(217)	0.42	0.339
dan_daner_ANX_Gedi_GSMS.	0	1.00	0.035	0.042(208)	0.042(551)	0.586	0.278
dan_daner_ANX_Gedi_VTSABD.	0	1.00	0.492	0.039(224)	0.039(406)	0.0504	0.0733
dan_daner_ANX_HUNT.	0	0.95	0.282	0.029(6587)	0.027(40740)	0.0646	0.06
dan_daner_ANX_MGHI.	0	0.92	0.573	0.035(1823)	0.033(5151)	0.0622	0.11
dan_daner_ANX_MVP.	0	1.00	0.000158	0.037(28525)	0.037(163731)	0.0967	0.0256
dan_daner_ANX_MoBa.	0	1.00	0.489	0.036(715)	0.032(22168)	0.104	0.151
dan_daner_ANX_PBK1.	0	1.00	0.319	0.038(1121)	0.035(11897)	0.116	0.116
dan_daner_ANX_QIMR_AGDS.	0	0.94	0.609	0.040(7312)	0.040(12813)	0.038	0.0742
dan_daner_ANX_QIMR_Ad.	0	0.94	0.805	0.040(2449)	0.040(6343)	−0.0242	0.0981
dan_daner_ANX_QIMR_Nuup.	0	0.94	0.831	0.037(1131)	0.037(1233)	0.0385	0.18
dan_daner_ANX_SWE_Catss.	0	1.00	0.433	0.016(540)	0.016(2333)	−0.25	0.319
dan_daner_ANX_SWE_Salty.	0	1.00	0.76	0.023(687)	0.023(3445)	−0.0635	0.208
dan_daner_ANX_SWE_TwinGene.	0	1.00	0.0333	0.021(1740)	0.021(6470)	0.271	0.127
dan_daner_ANX_UKBB.	0	0.96	0.118	0.042(25453)	0.042(58113)	0.0443	0.0284
dan_daner_ANX_Utah.	0	0.83	0.376	0.032(258)	0.024(279)	0.363	0.409
dan_daner_ANX_bioVU.	0	1.00	0.286	0.036(7586)	0.036(50545)	0.054	0.0506
dan_daner_ANX_iPSYCH.	0	0.92	0.02	0.042(5288)	0.037(14422)	0.142	0.0608
dan_daner_ANX_musp.	0	0.92	0.505	0.036(316)	0.044(715)	−0.173	0.26
**dan_**	**0**	**0.98**	**2.09e−08**	**0.037(122225)**	**0.034(729960)**	**0.0728**	**0.013**

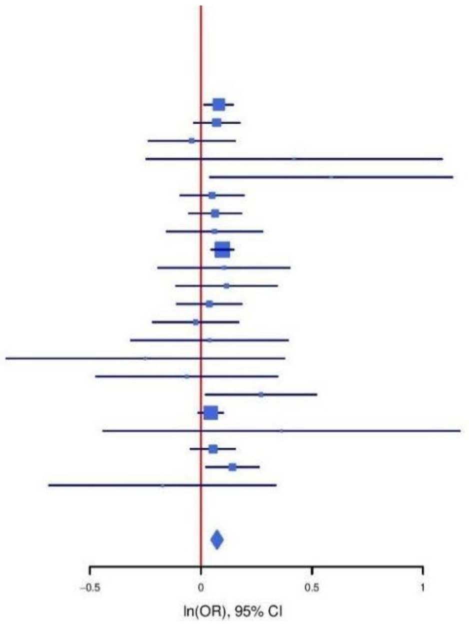

Supplementary Figure 27: Regional association plot (A) and forest plots (B,C) of SNP rs3847960 and rs544271348. See full figure description on page 30.

A

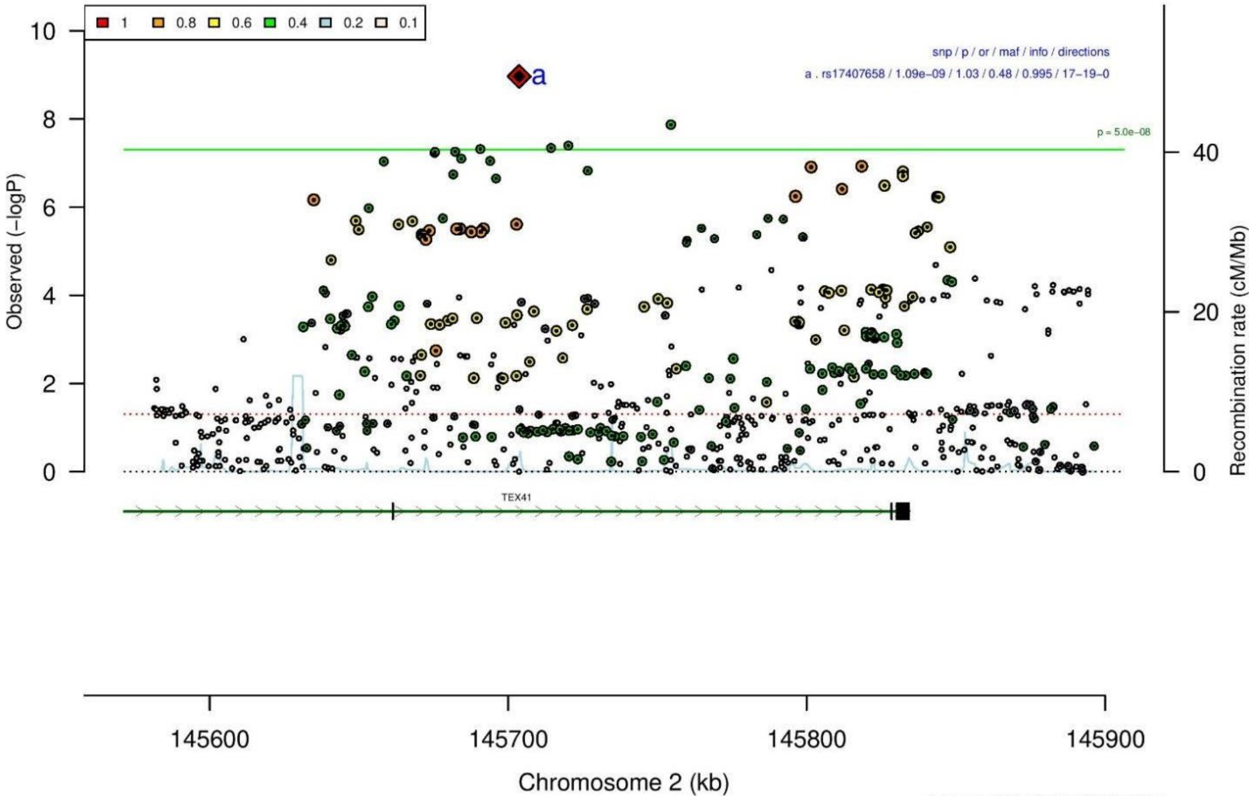

B

rs17407658		G/A		2:145703652			
------+--+++---++--++++---+++++--++-				het_P:	0.129	het_I:	21.5
	ngt	info	p_value	f_ca(n)	f_co(n)	ln(OR)	STDerr
dan_daner_ANX_ANGST_gsk.	0	0.91	0.543	0.480(852)	0.480(1103)	0.0435	0.0716
dan_daner_ANX_ANGST_mgs.	0	1.00	0.402	0.490(557)	0.490(779)	0.0677	0.0808
dan_daner_ANX_ANGST_ntr.	0	1.00	0.725	0.514(1521)	0.514(2970)	0.0217	0.0616
dan_daner_ANX_ANGST_rs1.	0	0.99	0.101	0.503(331)	0.503(1629)	0.162	0.099
dan_daner_ANX_ANGST_rs2.	0	0.99	0.59	0.498(209)	0.498(1023)	0.058	0.108
dan_daner_ANX_ANGST_rs3.	0	0.99	0.582	0.494(370)	0.494(1817)	−0.0457	0.083
dan_daner_ANX_ANGST_ship.	0	0.99	0.554	0.523(545)	0.523(834)	0.0481	0.0814
dan_daner_ANX_ANGST_trails.	0	0.99	0.643	0.536(278)	0.536(336)	0.0555	0.12
dan_daner_ANX_EstBB.	0	0.99	0.164	0.572(12523)	0.572(134954)	−0.019	0.0137
dan_daner_ANX_FinnGen.	0	1.00	0.000224	0.534(9182)	0.534(160108)	−0.0569	0.0154
dan_daner_ANX_GSC.	0	1.01	0.0688	0.474(1306)	0.492(12675)	−0.0757	0.0416
dan_daner_ANX_Gedi_CHDS.	0	1.00	0.622	0.514(244)	0.514(217)	0.0654	0.133
dan_daner_ANX_Gedi_GSMS.	0	1.00	0.619	0.482(208)	0.482(551)	0.0621	0.125
dan_daner_ANX_Gedi_VTSABD.	0	1.00	0.98	0.483(224)	0.483(406)	0.000696	0.0279
dan_daner_ANX_HUNT.	0	1.00	0.161	0.513(6587)	0.518(40740)	−0.0271	0.0193
dan_daner_ANX_MGHI.	0	1.00	0.639	0.498(1823)	0.495(5151)	0.0181	0.0387
dan_daner_ANX_MVP.	0	1.00	0.00367	0.488(28525)	0.488(163731)	−0.028	0.0096
dan_daner_ANX_MoBa.	0	1.00	0.546	0.517(715)	0.524(22168)	−0.0328	0.0543
dan_daner_ANX_PANIC_dca1.	0	0.96	0.795	0.504(248)	0.506(970)	−0.0269	0.104
dan_daner_ANX_PANIC_gmdc.	0	0.87	0.978	0.527(247)	0.517(537)	0.00331	0.12
dan_daner_ANX_PANIC_gom3.	0	0.97	0.915	0.511(472)	0.507(1803)	0.00813	0.0759
dan_daner_ANX_PANIC_gwc3.	0	1.02	0.296	0.536(280)	0.507(855)	0.108	0.104
dan_daner_ANX_PANIC_swdf.	0	0.99	0.00613	0.485(561)	0.520(2591)	−0.187	0.0681
dan_daner_ANX_PBK1.	0	1.00	0.0157	0.469(1121)	0.497(11897)	−0.107	0.0444
dan_daner_ANX_QIMR_AGDS.	0	0.97	0.0342	0.482(7312)	0.482(12813)	−0.0612	0.0289
dan_daner_ANX_QIMR_Ad.	0	0.97	0.137	0.491(2449)	0.491(6343)	−0.0528	0.0355
dan_daner_ANX_QIMR_Nuup.	0	0.97	0.104	0.482(1131)	0.482(1233)	−0.102	0.0625
dan_daner_ANX_SWE_Catss.	0	1.00	0.836	0.474(540)	0.474(2333)	0.0146	0.0706
dan_daner_ANX_SWE_Salty.	0	1.00	0.465	0.466(687)	0.466(3445)	0.0447	0.0612
dan_daner_ANX_SWE_TwinGene.	0	1.00	0.828	0.462(1740)	0.462(6470)	−0.00843	0.0388
dan_daner_ANX_UKBB.	0	0.99	5.09e−05	0.496(25453)	0.496(58113)	−0.0451	0.0111
dan_daner_ANX_Utah.	0	1.05	0.285	0.519(258)	0.486(279)	0.128	0.12
dan_daner_ANX_alspac.	0	1.00	0.373	0.509(536)	0.496(3404)	0.06	0.0673
dan_daner_ANX_bioVU.	0	1.00	0.223	0.489(7586)	0.489(50545)	−0.0226	0.0186
dan_daner_ANX_iPSYCH.	0	0.99	0.318	0.503(5288)	0.498(14422)	0.0231	0.0232
dan_daner_ANX_musp.	0	1.00	0.323	0.481(316)	0.506(715)	−0.0949	0.0961
**dan_**	**0**	**0.99**	**1.09e−09**	**0.504(122225)**	**0.518(729960)**	**−0.0287**	**0.0047**

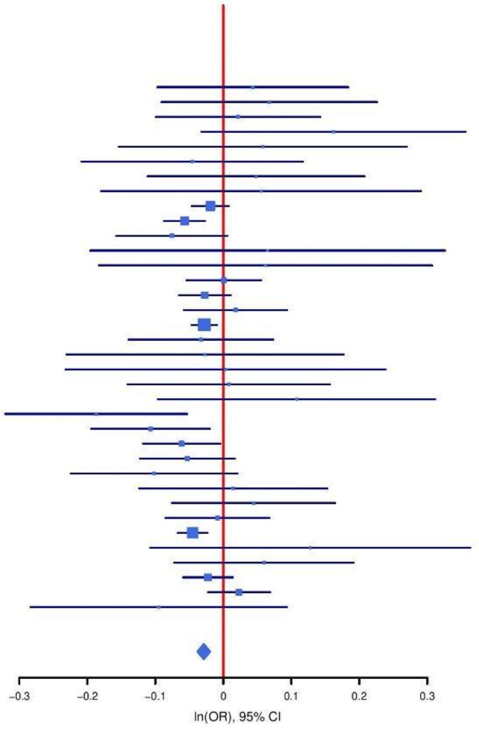

Supplementary Figure 28: Regional association plot (A) and forest plot (B) of SNP rs17407658. See full figure description on page 30.

A

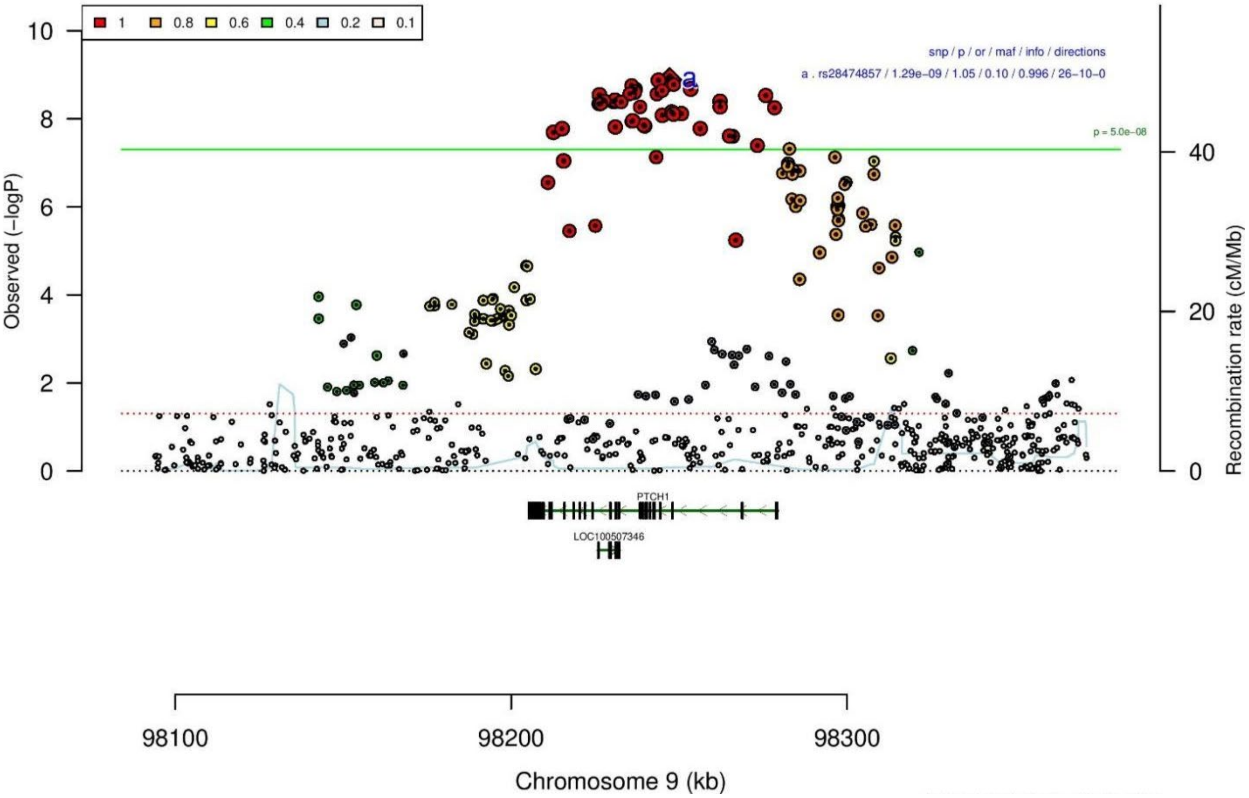

B

rs28474857		T/C		9:98247204			
+−+−+−−++++++−+++−−−+−++++++−+++++				het_P:	0.0171	het_I:	36.3
	**ngt**	**info**	**p_value**	**f_ca(n)**	**f_co(n)**	**ln(OR)**	**STDerr**
dan_daner_ANX_ANGST_gsk.	0	0.85	0.512	0.082(852)	0.082(1103)	−0.0868	0.132
dan_daner_ANX_ANGST_mgs.	0	1.00	0.687	0.093(557)	0.093(779)	0.0566	0.14
dan_daner_ANX_ANGST_ntr.	0	1.00	0.24	0.100(1521)	0.100(2970)	−0.122	0.104
dan_daner_ANX_ANGST_rs1.	0	0.96	0.404	0.094(331)	0.094(1629)	0.137	0.164
dan_daner_ANX_ANGST_rs2.	0	0.97	0.424	0.100(209)	0.100(1023)	−0.149	0.186
dan_daner_ANX_ANGST_rs3.	0	0.95	0.0428	0.092(370)	0.092(1817)	−0.315	0.156
dan_daner_ANX_ANGST_ship.	0	1.00	0.754	0.120(545)	0.120(834)	0.0405	0.13
dan_daner_ANX_ANGST_trails.	0	1.00	0.474	0.085(278)	0.085(336)	0.155	0.216
dan_daner_ANX_EstBB.	0	1.00	0.0504	0.129(12523)	0.129(134954)	0.0393	0.0201
dan_daner_ANX_FinnGen.	0	0.99	0.0563	0.105(9182)	0.105(160108)	0.0471	0.0247
dan_daner_ANX_GSC.	0	0.99	0.195	0.101(1306)	0.094(12675)	0.0934	0.072
dan_daner_ANX_Gedi_CHDS.	0	1.00	0.0254	0.088(244)	0.088(217)	0.588	0.263
dan_daner_ANX_Gedi_GSMS.	0	1.00	0.626	0.091(208)	0.091(551)	0.1	0.206
dan_daner_ANX_Gedi_VTSABD.	0	1.00	0.63	0.097(224)	0.097(406)	−0.0223	0.0463
dan_daner_ANX_HUNT.	0	1.00	0.684	0.123(6587)	0.121(40740)	0.012	0.0295
dan_daner_ANX_MGHI.	0	0.96	0.458	0.082(1823)	0.085(5151)	−0.0533	0.0718
dan_daner_ANX_MVP.	0	1.00	0.0324	0.093(28525)	0.093(163731)	0.0348	0.0163
dan_daner_ANX_MoBa.	0	1.00	0.846	0.118(715)	0.120(22168)	−0.0161	0.0826
dan_daner_ANX_PANIC_dca1.	0	1.01	0.375	0.096(248)	0.108(970)	−0.152	0.171
dan_daner_ANX_PANIC_gmdc.	0	0.94	0.523	0.101(247)	0.093(537)	0.124	0.194
dan_daner_ANX_PANIC_gom3.	0	0.96	0.623	0.112(472)	0.108(1803)	0.0596	0.121
dan_daner_ANX_PANIC_gwc3.	0	0.96	0.00147	0.142(280)	0.090(855)	0.518	0.163
dan_daner_ANX_PANIC_swdf.	0	1.00	0.0376	0.140(561)	0.119(2591)	0.206	0.0992
dan_daner_ANX_PBK1.	0	1.00	0.0014	0.097(1121)	0.081(11897)	0.244	0.0763
dan_daner_ANX_QIMR_AGDS.	0	0.98	0.462	0.090(7312)	0.090(12813)	0.0369	0.0502
dan_daner_ANX_QIMR_Ad.	0	0.98	0.291	0.090(2449)	0.090(6343)	0.0657	0.0622
dan_daner_ANX_QIMR_Nuup.	0	0.98	0.0555	0.089(1131)	0.089(1233)	−0.216	0.113
dan_daner_ANX_SWE_Catss.	0	1.00	0.119	0.120(540)	0.120(2333)	0.164	0.105
dan_daner_ANX_SWE_Salty.	0	1.00	0.826	0.112(687)	0.112(3445)	0.0209	0.0947
dan_daner_ANX_SWE_TwinGene.	0	1.00	0.656	0.111(1740)	0.111(6470)	0.0272	0.061
dan_daner_ANX_UKBB.	0	1.00	1.45e−05	0.090(25453)	0.090(58113)	0.084	0.0194
dan_daner_ANX_Utah.	0	0.98	0.739	0.088(258)	0.083(279)	0.0738	0.221
dan_daner_ANX_alspac.	0	0.98	0.369	0.104(536)	0.093(3404)	0.1	0.111
dan_daner_ANX_bioVU.	0	1.00	0.0372	0.089(7586)	0.089(50545)	0.0679	0.0326
dan_daner_ANX_iPSYCH.	0	1.01	0.0274	0.116(5288)	0.108(14422)	0.0801	0.0363
dan_daner_ANX_musp.	0	1.01	0.373	0.084(316)	0.099(715)	−0.151	0.169
**dan_**	**0**	**1.00**	**1.29e−09**	**0.100(122225)**	**0.105(729960)**	**0.0471**	**0.0078**

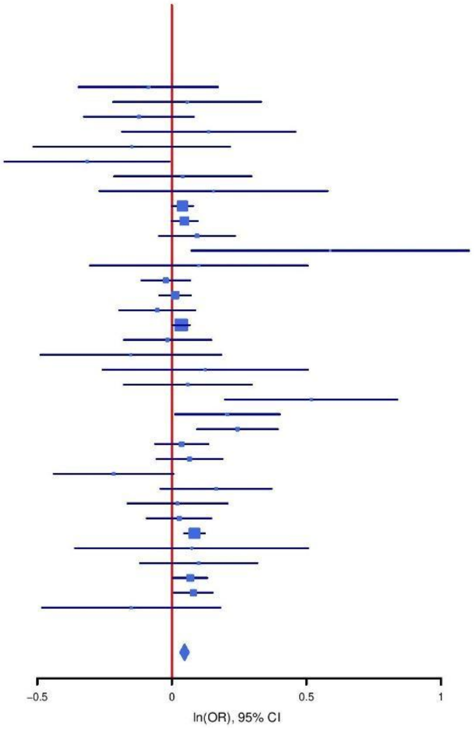

Supplementary Figure 29: Regional association plot (A) and forest plot (B) of SNP rs28474857. See full figure description on page 30.

A

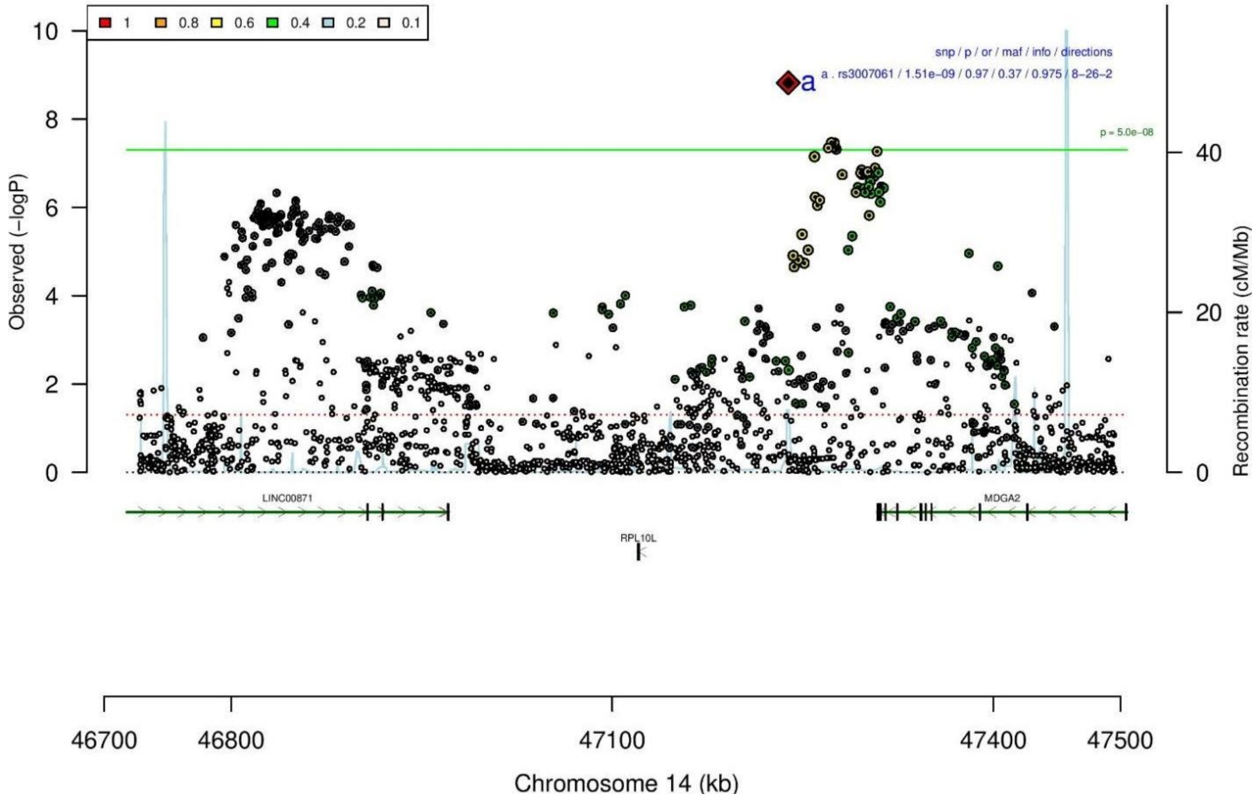

B

rs3007061 C/T 14:47238606

−?−+−−−+−−−−−−−−+−−−−−−−−+−+−?+−+−−−−+

het_P: 0.166 het_I: 19.0

	ngt	info	p_value	f_ca(n)	f_co(n)	ln(OR)	STDerr
dan_daner_ANX_ANGST_mgs.	0	1.00	0.356	0.625(557)	0.625(779)	0.0815	0.0883
dan_daner_ANX_ANGST_ntr.	0	0.99	0.892	0.621(1521)	0.621(2970)	−0.00865	0.0636
dan_daner_ANX_ANGST_rs1.	0	0.88	0.042	0.633(331)	0.633(1629)	0.228	0.112
dan_daner_ANX_ANGST_rs2.	0	0.88	0.704	0.623(209)	0.623(1023)	0.046	0.121
dan_daner_ANX_ANGST_rs3.	0	0.88	0.766	0.641(370)	0.641(1817)	0.0266	0.0893
dan_daner_ANX_ANGST_ship.	0	0.92	0.911	0.652(545)	0.652(834)	−0.0101	0.0904
dan_daner_ANX_ANGST_trails.	0	0.84	0.0184	0.625(278)	0.625(336)	0.319	0.135
dan_daner_ANX_EstBB.	0	0.96	0.864	0.656(12523)	0.656(134954)	0.00247	0.0144
dan_daner_ANX_FinnGen.	0	0.98	0.11	0.616(9182)	0.616(160108)	0.0253	0.0158
dan_daner_ANX_GSC.	1	0.99	0.31	0.608(1306)	0.616(12675)	−0.0441	0.0434
dan_daner_ANX_Gedi_CHDS.	0	1.00	0.619	0.600(244)	0.600(217)	0.0728	0.146
dan_daner_ANX_Gedi_GSMS.	0	1.00	0.049	0.638(208)	0.638(551)	0.25	0.127
dan_daner_ANX_Gedi_VTSABD.	0	1.00	0.82	0.638(224)	0.638(406)	0.00665	0.0293
dan_daner_ANX_HUNT.	0	0.96	0.0668	0.599(6587)	0.589(40740)	0.0368	0.0201
dan_daner_ANX_MGHI.	0	0.89	0.41	0.651(1823)	0.646(5151)	0.0354	0.043
dan_daner_ANX_MVP.	0	1.00	0.00118	0.647(28525)	0.647(163731)	0.0345	0.0106
dan_daner_ANX_MoBa.	0	1.00	0.948	0.607(715)	0.605(22168)	0.00358	0.0552
dan_daner_ANX_PANIC_dca1.	0	0.86	0.0693	0.655(248)	0.613(970)	0.209	0.115
dan_daner_ANX_PANIC_gmdc.	0	0.87	0.683	0.640(247)	0.652(537)	−0.0515	0.126
dan_daner_ANX_PANIC_gom3.	0	0.85	0.93	0.655(472)	0.648(1803)	0.00753	0.0856
dan_daner_ANX_PANIC_gwc3.	0	0.92	0.324	0.632(280)	0.653(855)	−0.111	0.113
dan_daner_ANX_PANIC_swdf.	0	0.88	0.915	0.625(561)	0.624(2591)	0.00793	0.0743
dan_daner_ANX_QIMR_AGDS.	0	0.90	0.639	0.616(7312)	0.616(12813)	0.0143	0.0305
dan_daner_ANX_QIMR_Ad.	0	0.90	0.45	0.608(2449)	0.608(6343)	−0.0285	0.0377
dan_daner_ANX_QIMR_Nuup.	0	0.90	0.153	0.613(1131)	0.613(1233)	−0.0961	0.0673
dan_daner_ANX_SWE_Catss.	0	1.00	0.16	0.362(540)	0.362(2333)	0.113	0.0807
dan_daner_ANX_SWE_Salty.	0	1.00	0.204	0.375(687)	0.375(3445)	0.0872	0.0686
dan_daner_ANX_SWE_TwinGene.	0	1.00	0.881	0.394(1740)	0.394(6470)	0.00589	0.0392
dan_daner_ANX_UKBB.	0	0.96	0.00032	0.615(25453)	0.615(58113)	0.042	0.0117
dan_daner_ANX_Utah.	0	0.85	0.683	0.601(258)	0.613(279)	−0.0554	0.136
dan_daner_ANX_alspac.	0	0.92	0.308	0.633(536)	0.618(3404)	0.0725	0.0712
dan_daner_ANX_bioVU.	0	1.00	0.00699	0.630(7586)	0.630(50545)	0.0524	0.0194
dan_daner_ANX_iPSYCH.	0	0.89	0.00319	0.625(5288)	0.609(14422)	0.0741	0.0251
dan_daner_ANX_musp.	0	0.86	0.0838	0.659(316)	0.621(715)	0.187	0.108
**dan_**	**1**	**0.97**	**1.51e−09**	**0.623(122225)**	**0.626(729960)**	**0.0305**	**0.0051**

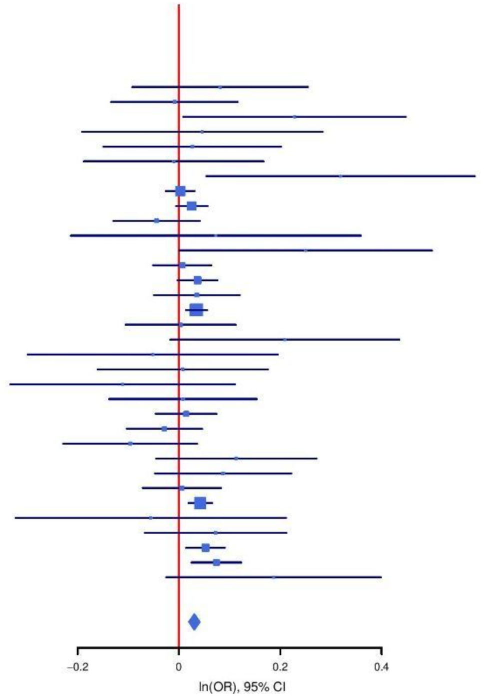

Supplementary Figure 30: Regional association plot (A) and forest plot (B) of SNP rs3007061. See full figure description on page 30.

A

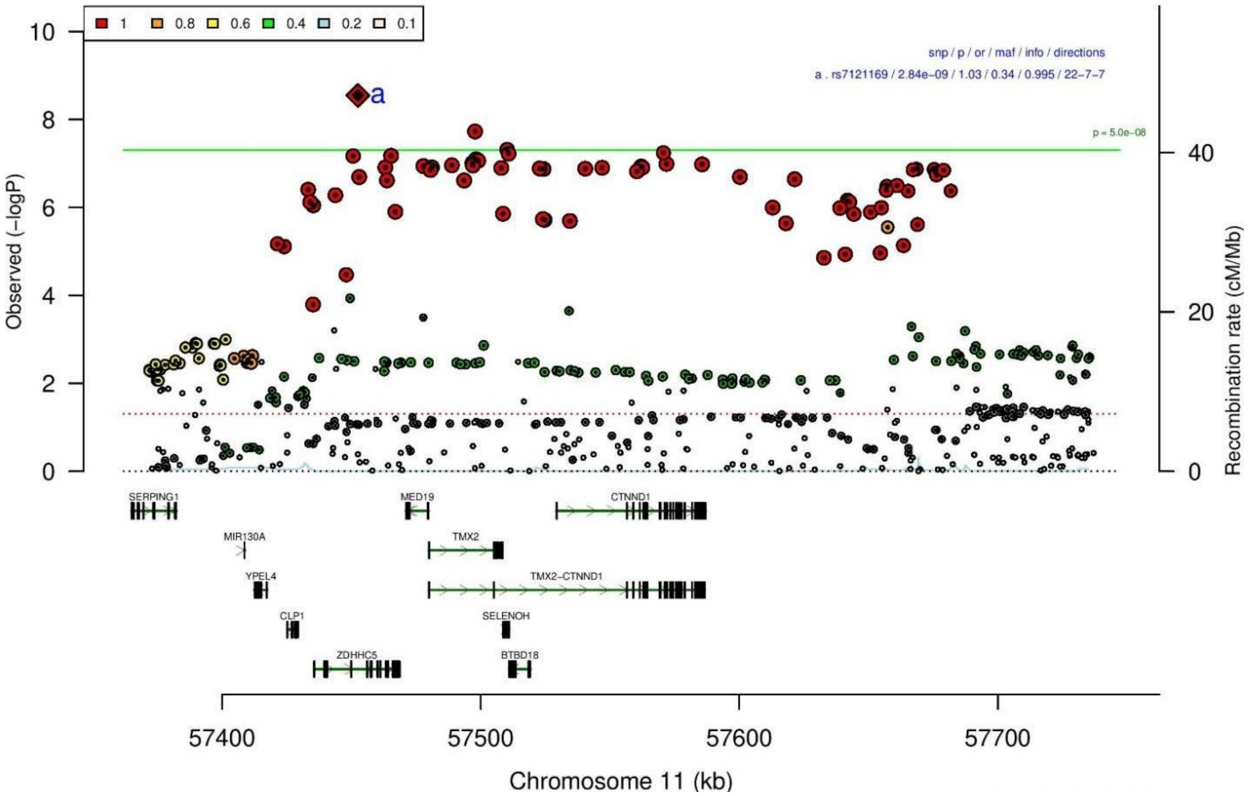

B

rs7121169 **A/G** **11:57452543**

++−++++−+??+++−?+++?++++−−++−++???+− het_P: 0.535 het_I: 0.0

	ngt	info	p_value	f_ca(n)	f_co(n)	ln(OR)	STDerr
dan_daner_ANX_ANGST_gsk.	0	0.98	0.752	0.298(852)	0.298(1103)	0.0235	0.0741
dan_daner_ANX_ANGST_mgs.	0	1.00	0.813	0.337(557)	0.337(779)	−0.02	0.0844
dan_daner_ANX_ANGST_ntr.	0	0.97	0.417	0.303(1521)	0.303(2970)	0.0559	0.0688
dan_daner_ANX_ANGST_rs1.	0	0.99	0.0515	0.316(331)	0.316(1629)	0.204	0.105
dan_daner_ANX_ANGST_rs2.	0	0.99	0.482	0.323(209)	0.323(1023)	0.083	0.118
dan_daner_ANX_ANGST_rs3.	0	0.99	0.012	0.320(370)	0.320(1817)	0.216	0.086
dan_daner_ANX_ANGST_ship.	0	0.99	0.772	0.342(545)	0.342(834)	−0.0251	0.0867
dan_daner_ANX_ANGST_trails.	0	0.99	0.848	0.337(278)	0.337(336)	0.0241	0.126
dan_daner_ANX_FinnGen.	0	0.99	0.0563	0.363(9182)	0.363(160108)	0.0304	0.0159
dan_daner_ANX_Gedi_CHDS.	0	1.00	0.918	0.313(244)	0.313(217)	0.015	0.146
dan_daner_ANX_Gedi_GSMS.	0	1.00	0.816	0.349(208)	0.349(551)	0.0292	0.126
dan_daner_ANX_Gedi_VTSABD.	0	1.00	0.314	0.342(224)	0.342(406)	−0.0292	0.029
dan_daner_ANX_HUNT.	0	1.00	0.117	0.341(6587)	0.333(40740)	0.0323	0.0206
dan_daner_ANX_MGHI.	0	0.99	0.253	0.341(1823)	0.329(5151)	0.0472	0.0413
dan_daner_ANX_MVP.	0	1.00	0.00134	0.319(28525)	0.319(163731)	0.0323	0.0101
dan_daner_ANX_PANIC_dca1.	0	1.03	0.809	0.339(248)	0.334(970)	0.0255	0.105
dan_daner_ANX_PANIC_gmdc.	0	1.01	0.493	0.321(247)	0.303(537)	0.0819	0.119
dan_daner_ANX_PANIC_gom3.	0	1.00	0.632	0.313(472)	0.324(1803)	−0.0384	0.0801
dan_daner_ANX_PANIC_gwc3.	0	1.00	0.975	0.326(280)	0.335(855)	−0.00351	0.111
dan_daner_ANX_PANIC_swdf.	0	0.99	0.134	0.360(561)	0.334(2591)	0.107	0.0711
dan_daner_ANX_PBK1.	0	1.00	0.00105	0.347(1121)	0.317(11897)	0.155	0.0473
dan_daner_ANX_QIMR_AGDS.	0	0.98	0.0197	0.331(7312)	0.331(12813)	0.0709	0.0304
dan_daner_ANX_QIMR_Ad.	0	0.98	0.951	0.327(2449)	0.327(6343)	−0.00229	0.0376
dan_daner_ANX_QIMR_Nuup.	0	0.98	0.598	0.330(1131)	0.330(1233)	0.035	0.0663
dan_daner_ANX_UKBB.	0	0.99	0.0308	0.325(25453)	0.325(58113)	0.0257	0.0119
dan_daner_ANX_Utah.	0	0.98	0.98	0.349(258)	0.350(279)	−0.00329	0.13
dan_daner_ANX_alspac.	0	1.00	0.21	0.340(536)	0.324(3404)	0.0887	0.0705
dan_daner_ANX_iPSYCH.	0	1.02	0.0276	0.339(5288)	0.327(14422)	0.0532	0.0241
dan_daner_ANX_musp.	0	0.96	0.297	0.345(316)	0.322(715)	0.108	0.104
**dan_**	**0**	**0.99**	**2.84e−09**	**0.330(122225)**	**0.336(729960)**	**0.0336**	**0.0057**

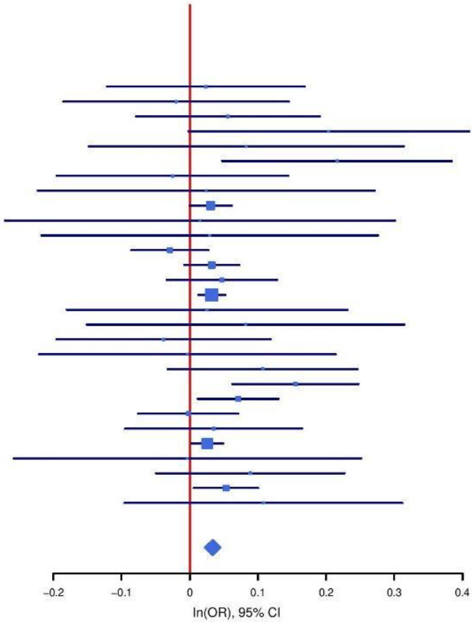

Supplementary Figure 31: Regional association plot (A) and forest plot (B) of SNP rs7121169. See full figure description on page 30.

A

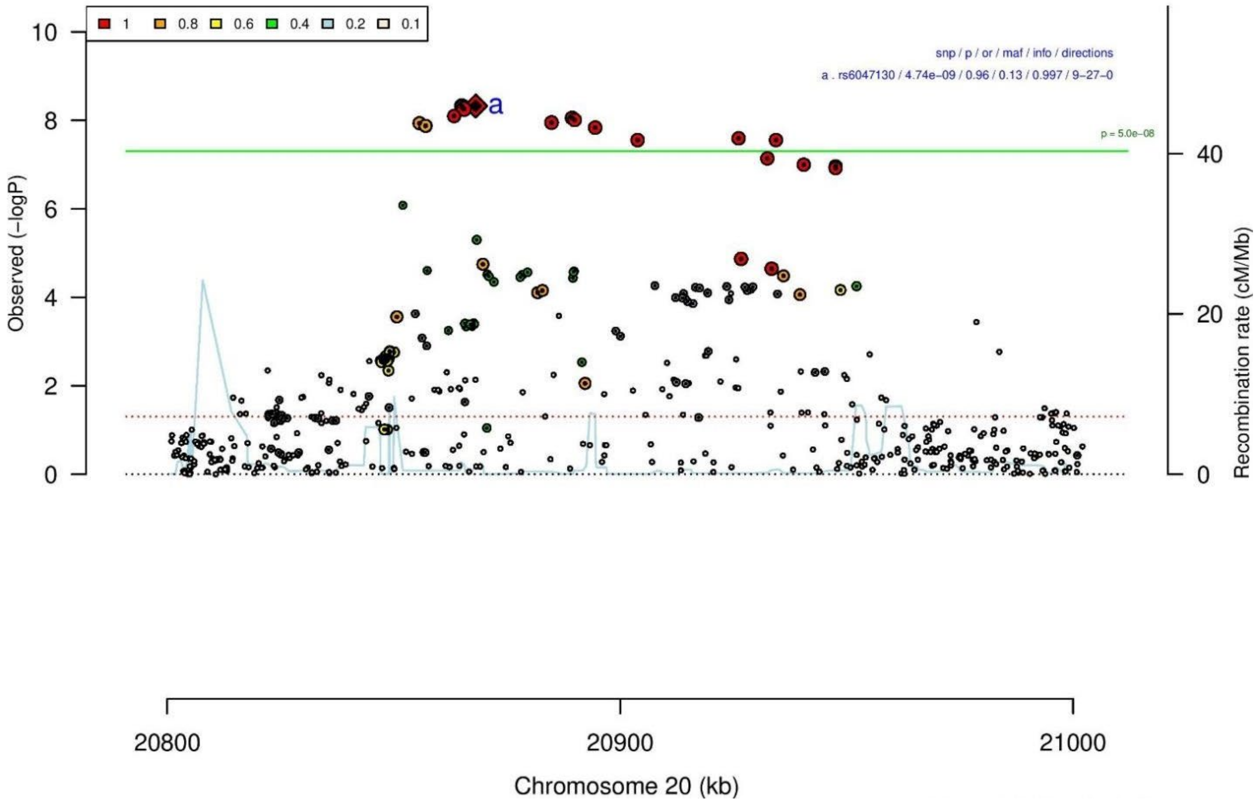

B

rs6047130		T/C		20:20868094			
−−−+++−−+−−−+−−−−−+−−−−−−−−+−−−−−+−+				het_P:	0.137	het_I:	20.8
	ngt	info	p_value	f_ca(n)	f_co(n)	ln(OR)	STDerr
dan_daner_ANX_ANGST_gsk.	0	0.97	0.661	0.116(852)	0.116(1103)	−0.0469	0.107
dan_daner_ANX_ANGST_mgs.	0	1.00	0.538	0.117(557)	0.117(779)	−0.0768	0.125
dan_daner_ANX_ANGST_ntr.	0	1.00	0.175	0.108(1521)	0.108(2970)	0.133	0.0983
dan_daner_ANX_ANGST_rs1.	0	0.99	0.971	0.113(331)	0.113(1629)	0.006	0.153
dan_daner_ANX_ANGST_rs2.	0	1.00	0.471	0.111(209)	0.111(1023)	0.125	0.173
dan_daner_ANX_ANGST_rs3.	0	0.99	0.695	0.118(370)	0.118(1817)	−0.052	0.133
dan_daner_ANX_ANGST_ship.	0	1.00	0.0479	0.116(545)	0.116(834)	−0.262	0.132
dan_daner_ANX_ANGST_trails.	0	0.96	0.132	0.117(278)	0.117(336)	0.279	0.185
dan_daner_ANX_EstBB.	0	1.00	0.195	0.144(12523)	0.144(134954)	−0.0248	0.0191
dan_daner_ANX_FinnGen.	0	1.00	0.0111	0.176(9182)	0.176(160108)	−0.0518	0.0204
dan_daner_ANX_GSC.	0	1.00	0.00553	0.100(1306)	0.119(12675)	−0.188	0.0691
dan_daner_ANX_Gedi_CHDS.	0	1.00	0.541	0.114(244)	0.114(217)	0.133	0.218
dan_daner_ANX_Gedi_GSMS.	0	1.00	0.263	0.113(208)	0.113(551)	−0.221	0.198
dan_daner_ANX_Gedi_VTSABD.	0	1.00	0.447	0.094(224)	0.094(406)	−0.0354	0.0465
dan_daner_ANX_HUNT.	0	1.00	0.405	0.092(6587)	0.093(40740)	−0.0274	0.033
dan_daner_ANX_MGHI.	0	1.00	0.262	0.110(1823)	0.102(5151)	0.0699	0.0623
dan_daner_ANX_MVP.	0	1.00	0.0131	0.114(28525)	0.114(163731)	−0.039	0.0157
dan_daner_ANX_MoBa.	0	1.00	0.401	0.093(715)	0.099(22168)	−0.0754	0.0897
dan_daner_ANX_PANIC_dca1.	0	0.91	0.806	0.109(248)	0.113(970)	−0.0418	0.17
dan_daner_ANX_PANIC_gmdc.	0	1.03	0.847	0.104(247)	0.102(537)	−0.0349	0.181
dan_daner_ANX_PANIC_gom3.	0	1.01	0.215	0.090(472)	0.102(1803)	−0.159	0.128
dan_daner_ANX_PANIC_gwc3.	0	0.98	0.795	0.118(280)	0.114(855)	−0.0427	0.165
dan_daner_ANX_PANIC_swdf.	0	1.00	0.0446	0.095(561)	0.114(2591)	−0.229	0.114
dan_daner_ANX_PBK1.	0	1.00	0.332	0.117(1121)	0.111(11897)	0.0667	0.0692
dan_daner_ANX_QIMR_AGDS.	0	0.99	0.418	0.110(7312)	0.110(12813)	−0.037	0.0457
dan_daner_ANX_QIMR_Ad.	0	0.99	0.0258	0.112(2449)	0.112(6343)	−0.126	0.0564
dan_daner_ANX_QIMR_Nuup.	0	0.99	0.344	0.108(1131)	0.108(1233)	−0.0955	0.101
dan_daner_ANX_SWE_Catss.	0	1.00	0.668	0.116(540)	0.116(2333)	−0.0487	0.114
dan_daner_ANX_SWE_Salty.	0	1.00	0.204	0.114(687)	0.114(3445)	−0.127	0.0997
dan_daner_ANX_SWE_TwinGene.	0	1.00	0.157	0.115(1740)	0.115(6470)	0.0848	0.0599
dan_daner_ANX_UKBB.	0	0.99	4.82e−06	0.112(25453)	0.112(58113)	−0.0817	0.0179
dan_daner_ANX_Utah.	0	1.05	0.169	0.124(258)	0.097(279)	0.266	0.193
dan_daner_ANX_alspac.	0	1.00	0.315	0.109(536)	0.116(3404)	−0.106	0.107
dan_daner_ANX_bioVU.	0	1.00	0.425	0.112(7586)	0.112(50545)	−0.0238	0.0299
dan_daner_ANX_iPSYCH.	0	0.99	0.882	0.111(5288)	0.113(14422)	−0.0055	0.0367
dan_daner_ANX_musp.	0	1.00	0.273	0.112(316)	0.129(715)	−0.164	0.15
**dan_**	**0**	**1.00**	**4.74e−09**	**0.119(122225)**	**0.131(729960)**	**−0.0429**	**0.0073**

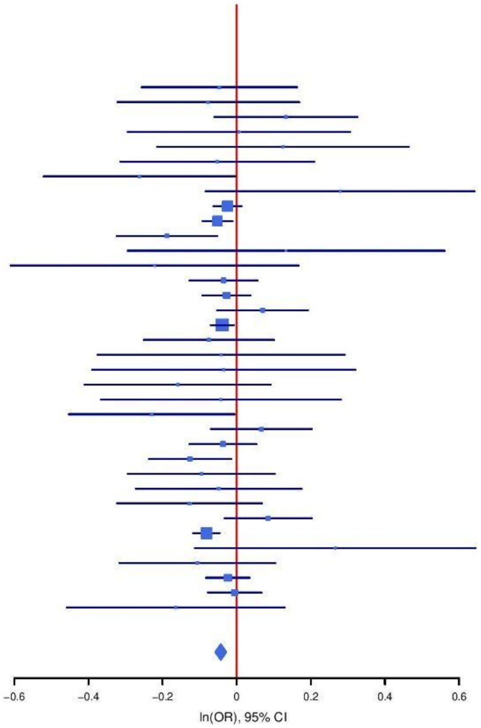

Supplementary Figure 32: Regional association plot (A) and forest plot (B) of SNP rs6047130. See full figure description on page 30.

A

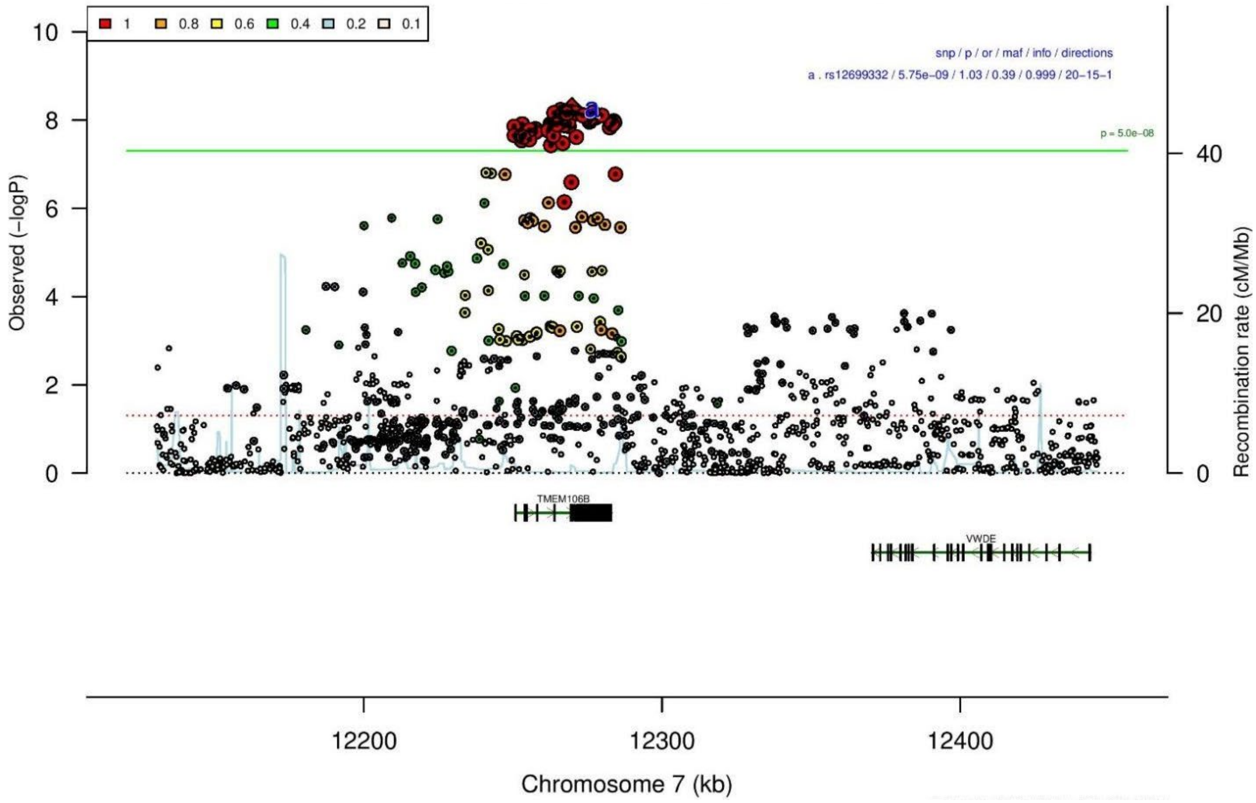

B

rs12699332 T/G 7:12269762

−?+−−−+−−+++−+++++−+−+++−+++−+−−−−++ het_P: 0.0222 het_I: 35.2

	ngt	info	p_value	f_ca(n)	f_co(n)	ln(OR)	STDerr
dan_daner_ANX_ANGST_mgs.	0	1.00	0.0188	0.453(557)	0.453(779)	0.247	0.105
dan_daner_ANX_ANGST_ntr.	0	1.00	0.233	0.424(1521)	0.424(2970)	−0.073	0.0613
dan_daner_ANX_ANGST_rs1.	0	1.00	0.595	0.422(331)	0.422(1629)	−0.053	0.099
dan_daner_ANX_ANGST_rs2.	0	1.00	0.685	0.421(209)	0.421(1023)	−0.045	0.11
dan_daner_ANX_ANGST_rs3.	0	1.00	0.0819	0.414(370)	0.414(1817)	0.14	0.0803
dan_daner_ANX_ANGST_ship.	0	1.00	0.222	0.390(545)	0.390(834)	−0.107	0.0873
dan_daner_ANX_ANGST_trails.	0	1.00	0.0745	0.414(278)	0.414(336)	−0.21	0.118
dan_daner_ANX_EstBB.	0	1.00	0.0137	0.355(12523)	0.355(134954)	0.0344	0.014
dan_daner_ANX_FinnGen.	0	1.00	0.0875	0.343(9182)	0.343(160108)	0.0276	0.0161
dan_daner_ANX_GSC.	0	1.00	0.138	0.439(1306)	0.423(12675)	0.0627	0.0423
dan_daner_ANX_Gedi_CHDS.	0	1.00	0.459	0.418(244)	0.418(217)	−0.103	0.14
dan_daner_ANX_Gedi_GSMS.	0	1.00	0.606	0.425(208)	0.425(551)	0.0619	0.12
dan_daner_ANX_Gedi_VTSABD.	0	1.00	0.197	0.416(224)	0.416(406)	0.0356	0.0276
dan_daner_ANX_HUNT.	0	1.00	0.104	0.426(6587)	0.422(40740)	0.0317	0.0195
dan_daner_ANX_MGHI.	0	0.99	0.54	0.422(1823)	0.426(5151)	−0.0241	0.0394
dan_daner_ANX_MVP.	0	1.00	0.0101	0.417(28525)	0.417(163731)	0.0243	0.0094
dan_daner_ANX_MoBa.	0	1.00	0.0148	0.450(715)	0.417(22168)	0.133	0.0547
dan_daner_ANX_PANIC_dca1.	0	0.99	0.38	0.442(248)	0.419(970)	0.0904	0.103
dan_daner_ANX_PANIC_gmdc.	0	0.98	0.441	0.411(247)	0.390(537)	0.0883	0.115
dan_daner_ANX_PANIC_gom3.	0	0.94	0.0348	0.378(472)	0.419(1803)	−0.166	0.0788
dan_daner_ANX_PANIC_gwc3.	0	0.99	0.0361	0.445(280)	0.401(855)	0.226	0.108
dan_daner_ANX_PANIC_swdf.	0	1.00	0.37	0.411(561)	0.409(2591)	0.062	0.0692
dan_daner_ANX_PBK1.	0	1.00	0.856	0.417(1121)	0.415(11897)	0.00797	0.0449
dan_daner_ANX_QIMR_AGDS.	0	0.99	0.349	0.413(7312)	0.413(12813)	0.0271	0.029
dan_daner_ANX_QIMR_Ad.	0	0.99	0.071	0.416(2449)	0.416(6343)	−0.0655	0.0363
dan_daner_ANX_QIMR_Nuup.	0	0.99	0.691	0.413(1131)	0.413(1233)	−0.0254	0.0639
dan_daner_ANX_SWE_Catss.	0	1.00	0.754	0.399(540)	0.399(2333)	−0.0228	0.0728
dan_daner_ANX_SWE_Salty.	0	1.00	0.871	0.401(687)	0.401(3445)	−0.00992	0.0611
dan_daner_ANX_SWE_TwinGene.	0	1.00	0.307	0.402(1740)	0.402(6470)	−0.0405	0.0396
dan_daner_ANX_UKBB.	0	1.00	3.29e−06	0.413(25453)	0.413(58113)	0.0523	0.0112
dan_daner_ANX_Utah.	0	1.00	0.413	0.428(258)	0.406(279)	0.102	0.125
dan_daner_ANX_alspac.	0	1.00	0.815	0.425(536)	0.431(3404)	−0.0159	0.0681
dan_daner_ANX_bioVU.	0	1.00	0.401	0.427(7586)	0.427(50545)	0.0158	0.0188
dan_daner_ANX_iPSYCH.	0	1.00	0.146	0.426(5288)	0.419(14422)	0.0338	0.0232
dan_daner_ANX_musp.	0	0.99	0.592	0.419(316)	0.431(715)	−0.0525	0.0979
**dan_**	**0**	**1.00**	**5.75e−09**	**0.406(122225)**	**0.390(729960)**	**0.0277**	**0.0048**

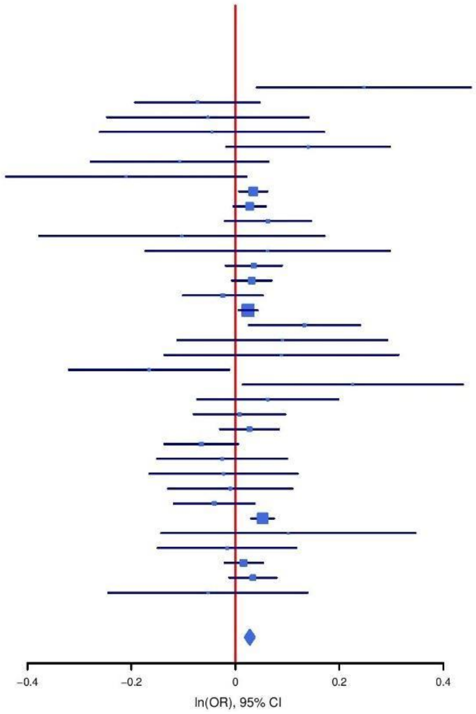

Supplementary Figure 33: Regional association plot (A) and forest plot (B) of SNP rs12699332. See full figure description on page 30.

A

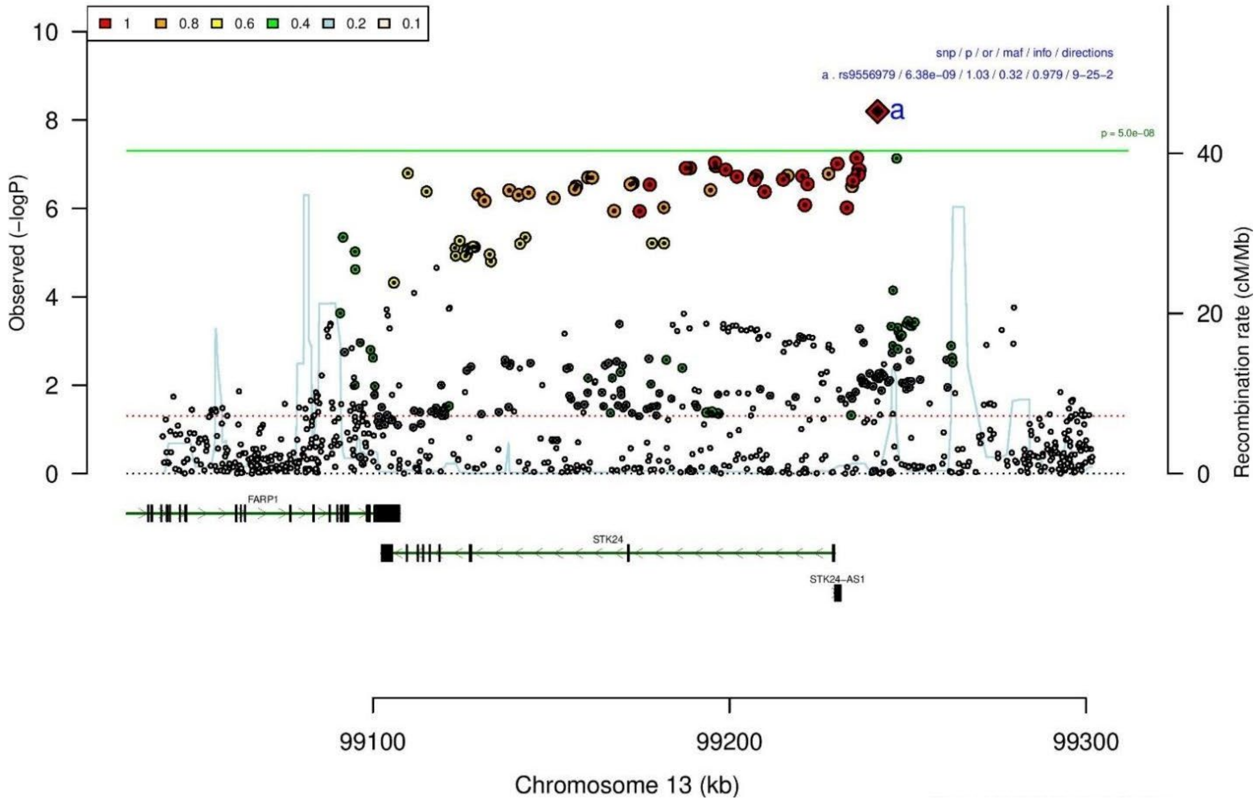

B

rs9556979 **G/T** **13:99241507**

+-----+++?--++-+----+------?+------- het_P: 0.852 het_I: 0.0

	ngt	info	p_value	f_ca(n)	f_co(n)	ln(OR)	STDerr
dan_daner_ANX_ANGST_gsk.	0	0.85	0.202	0.318(852)	0.318(1103)	0.1	0.0788
dan_daner_ANX_ANGST_mgs.	0	1.00	0.0581	0.320(557)	0.320(779)	0.173	0.091
dan_daner_ANX_ANGST_ntr.	0	0.92	0.479	0.342(1521)	0.342(2970)	0.0475	0.0671
dan_daner_ANX_ANGST_rs1.	0	0.93	0.203	0.328(331)	0.328(1629)	0.134	0.105
dan_daner_ANX_ANGST_rs2.	0	0.93	0.224	0.328(209)	0.328(1023)	0.145	0.12
dan_daner_ANX_ANGST_rs3.	0	0.93	0.96	0.340(370)	0.340(1817)	−0.00456	0.0905
dan_daner_ANX_ANGST_ship.	0	0.94	0.872	0.305(545)	0.305(834)	−0.0147	0.0908
dan_daner_ANX_ANGST_trails.	0	0.94	0.401	0.336(278)	0.336(336)	−0.107	0.127
dan_daner_ANX_EstBB.	0	0.96	0.0423	0.290(12523)	0.290(134954)	0.0305	0.015
dan_daner_ANX_FinnGen.	0	0.99	0.0229	0.347(9182)	0.347(160108)	0.0366	0.0161
dan_daner_ANX_GSC.	0	0.96	0.839	0.326(1306)	0.329(12675)	−0.00929	0.0457
dan_daner_ANX_Gedi_CHDS.	0	1.00	0.54	0.344(244)	0.344(217)	−0.0917	0.149
dan_daner_ANX_Gedi_GSMS.	0	1.00	0.638	0.334(208)	0.334(551)	−0.0583	0.124
dan_daner_ANX_Gedi_VTSABD.	0	1.00	0.987	0.333(224)	0.333(406)	0.000498	0.0294
dan_daner_ANX_HUNT.	0	0.96	0.1	0.340(6587)	0.333(40740)	0.0343	0.0208
dan_daner_ANX_MGHI.	0	0.92	0.669	0.310(1823)	0.306(5151)	0.0187	0.0437
dan_daner_ANX_MVP.	0	1.00	0.00105	0.302(28525)	0.302(163731)	0.0367	0.0112
dan_daner_ANX_MoBa.	0	1.00	0.665	0.335(715)	0.328(22168)	0.0247	0.0571
dan_daner_ANX_PANIC_dca1.	0	0.90	0.638	0.323(248)	0.315(970)	0.0538	0.115
dan_daner_ANX_PANIC_gmdc.	0	0.96	0.00923	0.319(247)	0.268(537)	0.328	0.126
dan_daner_ANX_PANIC_gom3.	0	0.90	0.41	0.327(472)	0.314(1803)	0.0692	0.0841
dan_daner_ANX_PANIC_gwc3.	0	1.02	0.783	0.295(280)	0.306(855)	0.0312	0.113
dan_daner_ANX_PANIC_swdf.	0	0.93	0.622	0.334(561)	0.324(2591)	0.0368	0.0745
dan_daner_ANX_QIMR_AGDS.	0	0.93	0.425	0.316(7312)	0.316(12813)	0.0252	0.0316
dan_daner_ANX_QIMR_Ad.	0	0.93	0.623	0.314(2449)	0.314(6343)	−0.0191	0.0389
dan_daner_ANX_QIMR_Nuup.	0	0.93	0.409	0.304(1131)	0.304(1233)	0.0572	0.0693
dan_daner_ANX_SWE_Catss.	0	1.00	0.529	0.328(540)	0.328(2333)	0.0509	0.0808
dan_daner_ANX_SWE_Salty.	0	1.00	0.48	0.335(687)	0.335(3445)	0.0478	0.0676
dan_daner_ANX_SWE_TwinGene.	0	1.00	0.293	0.327(1740)	0.327(6470)	0.0437	0.0416
dan_daner_ANX_UKBB.	0	0.98	0.042	0.323(25453)	0.323(58113)	0.0245	0.012
dan_daner_ANX_Utah.	0	0.91	0.432	0.360(258)	0.341(279)	0.107	0.136
dan_daner_ANX_alspac.	0	0.96	0.221	0.308(536)	0.325(3404)	−0.0903	0.074
dan_daner_ANX_iPSYCH.	0	0.95	0.0121	0.346(5288)	0.333(14422)	0.0627	0.025
dan_daner_ANX_musp.	0	0.95	0.32	0.314(316)	0.333(715)	−0.105	0.106
**dan_**	**0**	**0.98**	**6.38e−09**	**0.317(122225)**	**0.318(729960)**	**0.0314**	**0.0054**

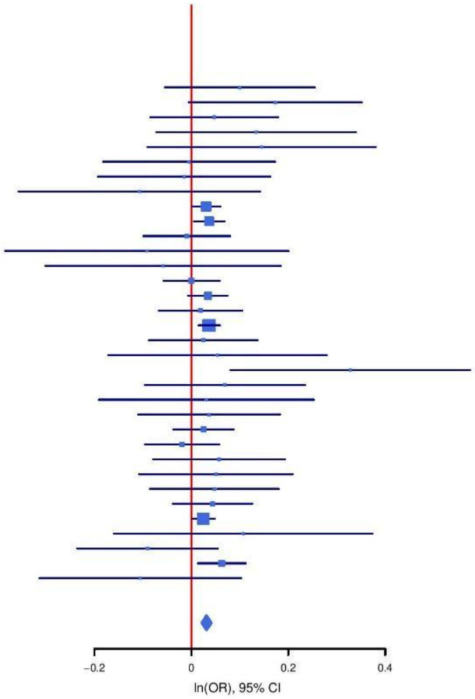

Supplementary Figure 34: Regional association plot (A) and forest plot (B) of SNP rs9556979. See full figure description on page 30.

A

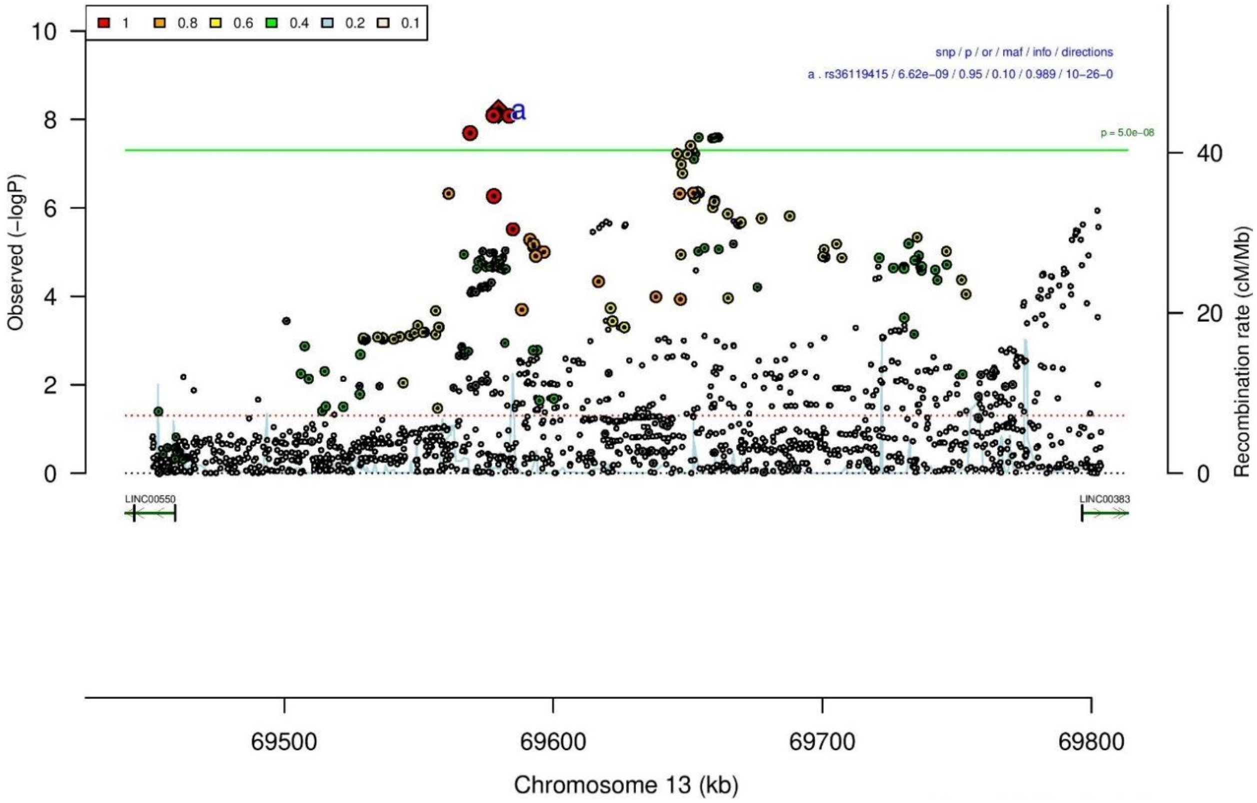

B

rs36119415 **T/G** **13:69579612**

++-++---+-----+----+---------+--------+-+

het_P: 0.0507 het_I: 29.6

	ngt	info	p_value	f_ca(n)	f_co(n)	ln(OR)	STDerr
dan_daner_ANX_ANGST_gsk.	0	0.92	0.734	0.102(852)	0.102(1103)	0.0381	0.112
dan_daner_ANX_ANGST_mgs.	0	1.00	0.3	0.097(557)	0.097(779)	−0.142	0.137
dan_daner_ANX_ANGST_ntr.	0	0.98	0.674	0.089(1521)	0.089(2970)	0.0454	0.108
dan_daner_ANX_ANGST_rs1.	0	0.95	0.593	0.093(331)	0.093(1629)	0.09	0.169
dan_daner_ANX_ANGST_rs2.	0	0.92	0.407	0.083(209)	0.083(1023)	−0.174	0.21
dan_daner_ANX_ANGST_rs3.	0	0.99	0.088	0.095(370)	0.095(1817)	−0.254	0.149
dan_daner_ANX_ANGST_ship.	0	0.95	0.619	0.079(545)	0.079(834)	−0.0789	0.159
dan_daner_ANX_ANGST_trails.	0	0.96	0.257	0.106(278)	0.106(336)	0.228	0.201
dan_daner_ANX_EstBB.	0	0.97	0.654	0.086(12523)	0.086(134954)	−0.0109	0.0244
dan_daner_ANX_FinnGen.	0	0.99	0.00308	0.104(9182)	0.104(160108)	−0.0762	0.0258
dan_daner_ANX_GSC.	0	0.98	0.836	0.107(1306)	0.107(12675)	−0.0141	0.0683
dan_daner_ANX_Gedi_CHDS.	0	1.00	0.0936	0.113(244)	0.113(217)	−0.357	0.213
dan_daner_ANX_Gedi_GSMS.	0	1.00	0.771	0.084(208)	0.084(551)	0.0616	0.211
dan_daner_ANX_Gedi_VTSABD.	0	1.00	0.301	0.081(224)	0.081(406)	−0.0528	0.051
dan_daner_ANX_HUNT.	0	1.00	0.000375	0.089(6587)	0.098(40740)	−0.119	0.0338
dan_daner_ANX_MGHI.	0	0.97	0.362	0.101(1823)	0.106(5151)	−0.0592	0.0649
dan_daner_ANX_MVP.	0	1.00	0.137	0.094(28525)	0.094(163731)	−0.0252	0.0169
dan_daner_ANX_MoBa.	0	1.00	0.557	0.091(715)	0.096(22168)	−0.0539	0.0918
dan_daner_ANX_PANIC_dca1.	0	1.01	0.632	0.067(248)	0.074(970)	−0.096	0.2
dan_daner_ANX_PANIC_gmdc.	0	0.88	0.765	0.089(247)	0.097(537)	−0.0621	0.208
dan_daner_ANX_PANIC_gom3.	0	0.93	0.0667	0.074(472)	0.090(1803)	−0.272	0.148
dan_daner_ANX_PANIC_gwc3.	0	0.91	0.742	0.085(280)	0.081(855)	0.065	0.197
dan_daner_ANX_PANIC_swdf.	0	0.97	0.733	0.082(561)	0.085(2591)	−0.0425	0.125
dan_daner_ANX_PBK1.	0	1.00	0.116	0.089(1121)	0.099(11897)	−0.123	0.0782
dan_daner_ANX_QIMR_AGDS.	0	0.98	0.0117	0.101(7312)	0.101(12813)	−0.12	0.0476
dan_daner_ANX_QIMR_Ad.	0	0.98	0.973	0.102(2449)	0.102(6343)	−0.002	0.0589
dan_daner_ANX_QIMR_Nuup.	0	0.98	0.0495	0.089(1131)	0.089(1233)	−0.216	0.11
dan_daner_ANX_SWE_Catss.	0	1.00	0.000851	0.084(540)	0.084(2333)	−0.482	0.145
dan_daner_ANX_SWE_Salty.	0	1.00	0.454	0.082(687)	0.082(3445)	−0.0867	0.116
dan_daner_ANX_SWE_TwinGene.	0	1.00	0.967	0.082(1740)	0.082(6470)	0.00294	0.0712
dan_daner_ANX_UKBB.	0	0.99	0.00331	0.101(25453)	0.101(58113)	−0.0549	0.0187
dan_daner_ANX_Utah.	0	1.01	0.403	0.093(258)	0.080(279)	0.183	0.218
dan_daner_ANX_alspac.	0	0.96	0.327	0.098(536)	0.089(3404)	0.114	0.115
dan_daner_ANX_bioVU.	0	1.00	0.0726	0.095(7586)	0.095(50545)	−0.0571	0.0318
dan_daner_ANX_iPSYCH.	0	0.97	0.22	0.082(5288)	0.079(14422)	0.0528	0.0431
dan_daner_ANX_musp.	0	1.03	0.0334	0.078(316)	0.110(715)	−0.363	0.171
**dan_**	**0**	**0.99**	**6.62e−09**	**0.095(122225)**	**0.096(729960)**	**−0.0474**	**0.0082**

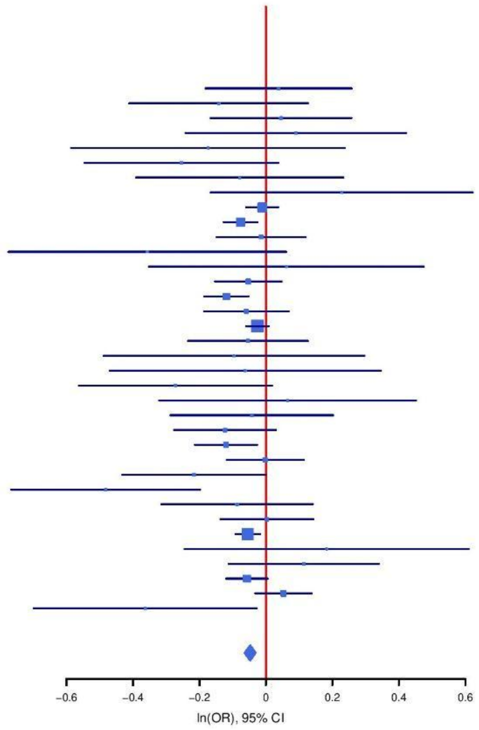

Supplementary Figure 35: Regional association plot (A) and forest plot (B) of SNP rs36119415. See full figure description on page 30.

A

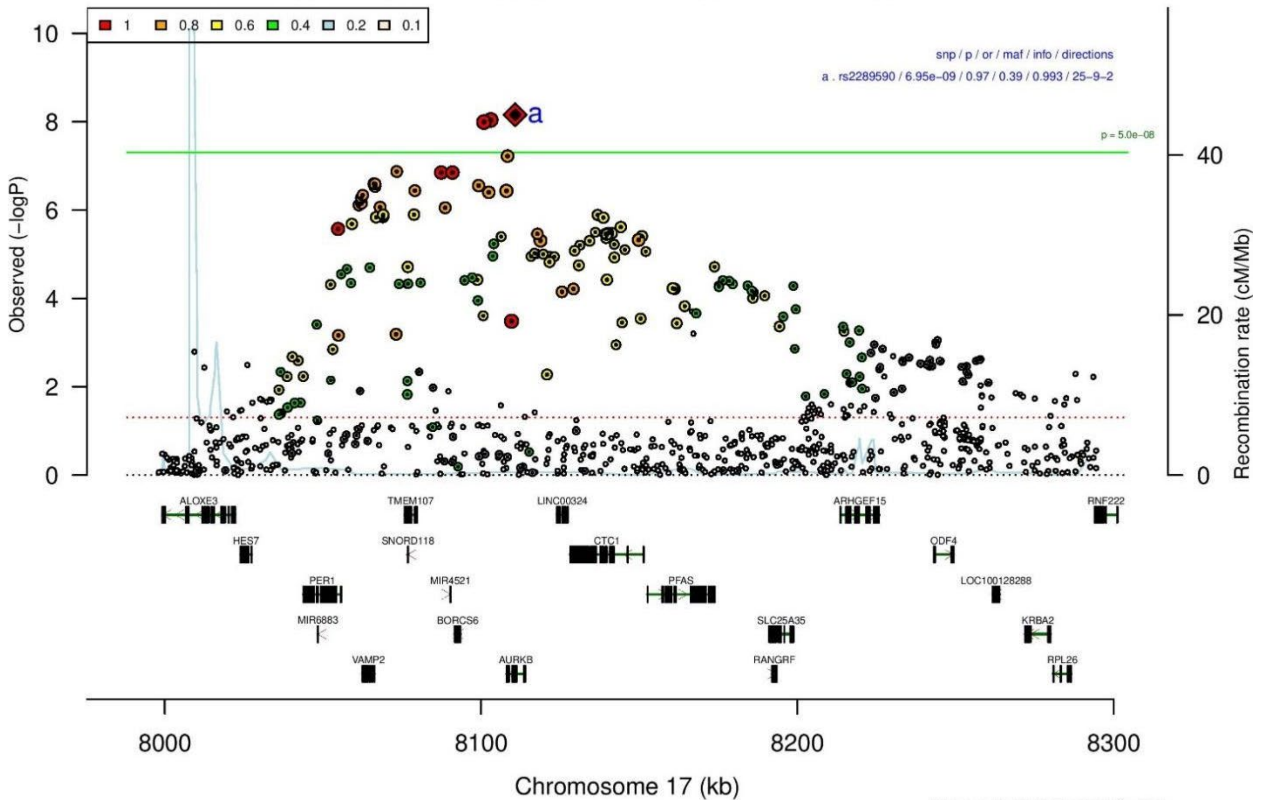

B

rs2289590		A/C		17:8110764			
+?+?+++−−++++++−+++−−+++++−−−++++−++				het_P:	0.67	het_I:	0.0
	**ngt**	**info**	**p_value**	**f_ca(n)**	**f_co(n)**	**ln(OR)**	**STDerr**
dan_daner_ANX_ANGST_mgs.	0	1.00	0.117	0.570(557)	0.570(779)	0.133	0.0847
dan_daner_ANX_ANGST_rs1.	0	0.99	0.809	0.581(331)	0.581(1629)	0.025	0.102
dan_daner_ANX_ANGST_rs2.	0	0.99	0.652	0.581(209)	0.581(1023)	0.049	0.109
dan_daner_ANX_ANGST_rs3.	0	0.99	0.356	0.589(370)	0.589(1817)	0.0762	0.0826
dan_daner_ANX_ANGST_ship.	0	0.98	0.71	0.625(545)	0.625(834)	−0.0319	0.0856
dan_daner_ANX_ANGST_trails.	0	0.98	0.0985	0.606(278)	0.606(336)	−0.201	0.121
dan_daner_ANX_EstBB.	0	0.98	0.0445	0.631(12523)	0.631(134954)	0.0282	0.014
dan_daner_ANX_FinnGen.	0	1.00	0.199	0.657(9182)	0.657(160108)	0.0209	0.0163
dan_daner_ANX_GSC.	0	0.98	0.401	0.596(1306)	0.602(12675)	−0.0363	0.0432
dan_daner_ANX_Gedi_CHDS.	0	1.00	0.743	0.592(244)	0.592(217)	0.0455	0.139
dan_daner_ANX_Gedi_GSMS.	0	1.00	0.392	0.592(208)	0.592(551)	0.106	0.124
dan_daner_ANX_Gedi_VTSABD.	0	1.00	0.866	0.581(224)	0.581(406)	0.00474	0.0282
dan_daner_ANX_HUNT.	0	1.00	0.256	0.623(6587)	0.613(40740)	0.0225	0.0199
dan_daner_ANX_MGHI.	0	1.01	0.739	0.551(1823)	0.545(5151)	0.0131	0.0393
dan_daner_ANX_MVP.	0	1.00	4.15e−08	0.574(28525)	0.574(163731)	0.0543	0.0099
dan_daner_ANX_MoBa.	0	1.00	0.565	0.606(715)	0.611(22168)	−0.0321	0.0557
dan_daner_ANX_PANIC_dca1.	0	1.04	0.366	0.638(248)	0.619(970)	0.0936	0.104
dan_daner_ANX_PANIC_gmdc.	0	0.99	0.899	0.570(247)	0.573(537)	0.0146	0.115
dan_daner_ANX_PANIC_gom3.	0	1.02	0.219	0.623(472)	0.607(1803)	0.0939	0.0763
dan_daner_ANX_PANIC_gwc3.	0	1.01	0.333	0.612(280)	0.577(855)	0.103	0.106
dan_daner_ANX_PANIC_swdf.	0	1.01	0.688	0.629(561)	0.629(2591)	−0.0282	0.0702
dan_daner_ANX_PBK1.	0	1.00	0.726	0.571(1121)	0.582(11897)	−0.0159	0.0452
dan_daner_ANX_QIMR_AGDS.	0	0.94	0.116	0.578(7312)	0.578(12813)	0.0466	0.0297
dan_daner_ANX_QIMR_Ad.	0	0.94	0.854	0.570(2449)	0.570(6343)	−0.0069	0.0375
dan_daner_ANX_QIMR_Nuup.	0	0.94	0.151	0.580(1131)	0.580(1233)	0.0942	0.0657
dan_daner_ANX_SWE_Catss.	0	1.00	0.868	0.366(540)	0.366(2333)	0.0122	0.0736
dan_daner_ANX_SWE_Salty.	0	1.00	0.344	0.366(687)	0.366(3445)	0.0596	0.063
dan_daner_ANX_SWE_TwinGene.	0	1.00	0.467	0.377(1740)	0.377(6470)	−0.0294	0.0404
dan_daner_ANX_UKBB.	0	0.99	0.153	0.586(25453)	0.586(58113)	0.0163	0.0114
dan_daner_ANX_Utah.	0	1.10	0.708	0.603(258)	0.590(279)	0.045	0.12
dan_daner_ANX_alspac.	0	0.99	0.869	0.588(536)	0.584(3404)	0.0114	0.0686
dan_daner_ANX_bioVU.	0	1.00	0.131	0.586(7586)	0.586(50545)	0.0288	0.019
dan_daner_ANX_iPSYCH.	0	0.99	0.212	0.617(5288)	0.609(14422)	0.0297	0.0238
dan_daner_ANX_musp.	0	0.98	0.248	0.573(316)	0.601(715)	−0.113	0.0981
**dan_**	**0**	**0.99**	**6.95e−09**	**0.591(122225)**	**0.606(729960)**	**0.0282**	**0.0049**

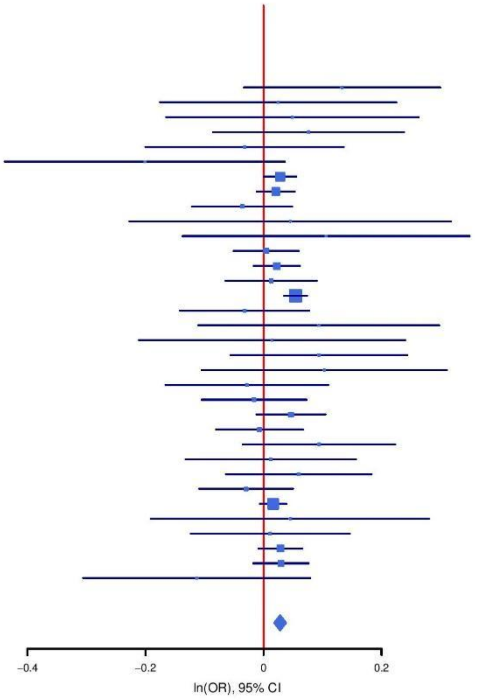

Supplementary Figure 36: Regional association plot (A) and forest plot (B) of SNP rs2289590. See full figure description on page 30.

A

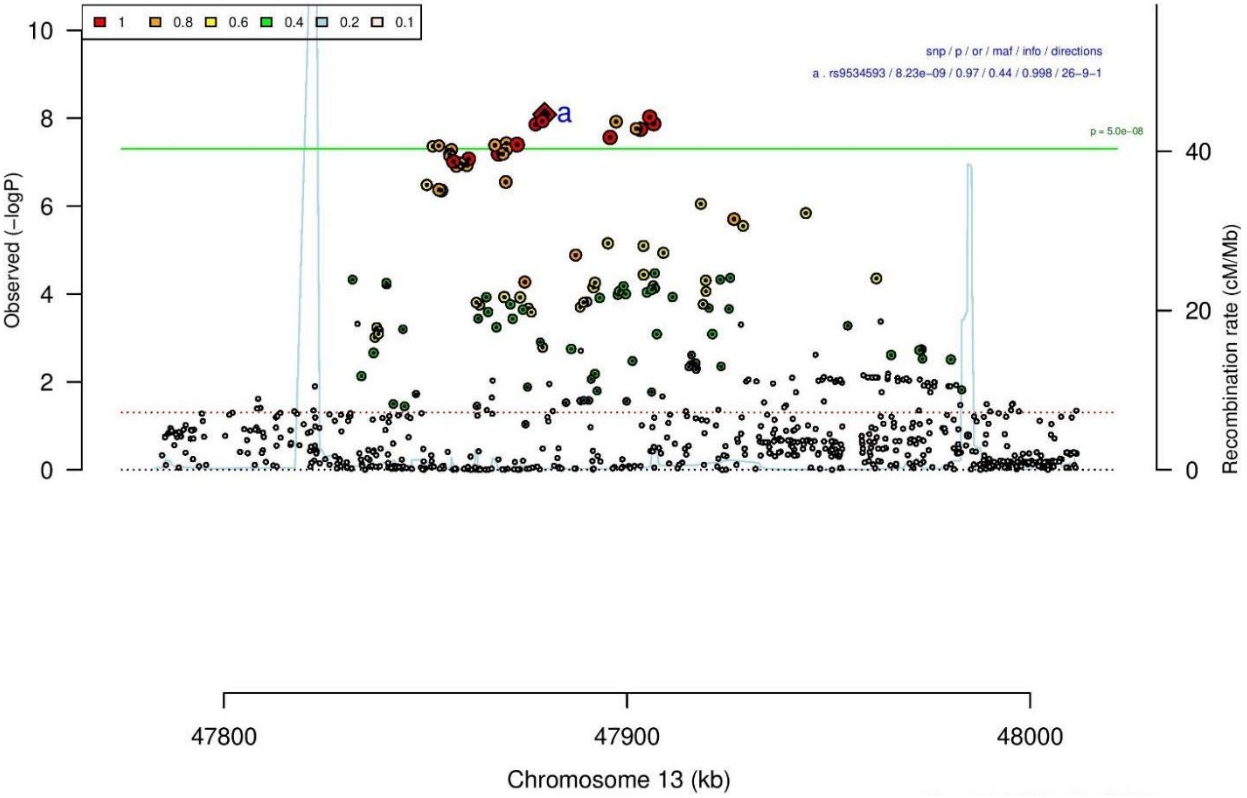

B

rs9534593 G/A 13:47879549

+?−+−−−−+++++++−+++−++++++++++−++++− het_P: 0.477 het_I: 0.0

	ngt	info	p_value	f_ca(n)	f_co(n)	ln(OR)	STDerr
dan_daner_ANX_ANGST_mgs.	0	1.00	0.114	0.432(557)	0.432(779)	0.127	0.0807
dan_daner_ANX_ANGST_ntr.	0	1.00	0.423	0.414(1521)	0.414(2970)	−0.0493	0.0616
dan_daner_ANX_ANGST_rs1.	0	1.00	0.47	0.411(331)	0.411(1629)	0.071	0.098
dan_daner_ANX_ANGST_rs2.	0	1.00	0.00287	0.391(209)	0.391(1023)	0.335	0.112
dan_daner_ANX_ANGST_rs3.	0	1.00	0.567	0.401(370)	0.401(1817)	0.048	0.0837
dan_daner_ANX_ANGST_ship.	0	1.00	0.533	0.410(545)	0.410(834)	0.051	0.0818
dan_daner_ANX_ANGST_trails.	0	0.99	0.83	0.399(278)	0.399(336)	−0.0253	0.118
dan_daner_ANX_EstBB.	0	1.00	0.142	0.414(12523)	0.414(134954)	−0.02	0.0136
dan_daner_ANX_FinnGen.	0	1.00	0.291	0.486(9182)	0.486(160108)	−0.0162	0.0154
dan_daner_ANX_GSC.	0	1.01	0.251	0.484(1306)	0.470(12675)	0.048	0.0418
dan_daner_ANX_Gedi_CHDS.	0	1.00	0.75	0.454(244)	0.454(217)	−0.0438	0.138
dan_daner_ANX_Gedi_GSMS.	0	1.00	0.413	0.421(208)	0.421(551)	−0.101	0.123
dan_daner_ANX_Gedi_VTSABD.	0	1.00	0.902	0.451(224)	0.451(406)	−0.00338	0.0274
dan_daner_ANX_HUNT.	0	1.00	0.0425	0.423(6587)	0.434(40740)	−0.0397	0.0196
dan_daner_ANX_MGHI.	0	0.99	0.397	0.430(1823)	0.436(5151)	−0.0332	0.0392
dan_daner_ANX_MVP.	0	1.00	7.33e−05	0.433(28525)	0.433(163731)	−0.0375	0.0094
dan_daner_ANX_MoBa.	0	1.00	0.945	0.421(715)	0.421(22168)	0.00378	0.0545
dan_daner_ANX_PANIC_dca1.	0	0.96	0.589	0.406(248)	0.418(970)	−0.0572	0.106
dan_daner_ANX_PANIC_gmdc.	0	0.96	0.0881	0.390(247)	0.442(537)	−0.199	0.117
dan_daner_ANX_PANIC_gom3.	0	0.98	0.191	0.391(472)	0.418(1803)	−0.1	0.0767
dan_daner_ANX_PANIC_gwc3.	0	0.99	0.831	0.409(280)	0.419(855)	−0.0231	0.108
dan_daner_ANX_PANIC_swdf.	0	0.99	0.597	0.414(561)	0.419(2591)	−0.0367	0.0694
dan_daner_ANX_PBK1.	0	1.00	0.388	0.427(1121)	0.438(11897)	−0.0391	0.0452
dan_daner_ANX_QIMR_AGDS.	0	0.98	0.00413	0.441(7312)	0.441(12813)	−0.0835	0.0291
dan_daner_ANX_QIMR_Ad.	0	0.98	0.745	0.452(2449)	0.452(6343)	−0.0115	0.0355
dan_daner_ANX_QIMR_Nuup.	0	0.98	0.918	0.447(1131)	0.447(1233)	0.00642	0.0621
dan_daner_ANX_SWE_Catss.	0	1.00	0.465	0.421(540)	0.421(2333)	−0.0525	0.0718
dan_daner_ANX_SWE_Salty.	0	1.00	0.799	0.419(687)	0.419(3445)	−0.0156	0.0612
dan_daner_ANX_SWE_TwinGene.	0	1.00	0.526	0.408(1740)	0.408(6470)	−0.025	0.0394
dan_daner_ANX_UKBB.	0	1.00	0.0324	0.444(25453)	0.444(58113)	−0.024	0.0112
dan_daner_ANX_Utah.	0	0.98	0.705	0.417(258)	0.409(279)	0.0477	0.126
dan_daner_ANX_alspac.	0	1.00	0.882	0.433(536)	0.433(3404)	−0.00989	0.0668
dan_daner_ANX_bioVU.	0	1.00	0.126	0.434(7586)	0.434(50545)	−0.0291	0.019
dan_daner_ANX_iPSYCH.	0	1.00	0.0689	0.403(5288)	0.415(14422)	−0.0426	0.0234
dan_daner_ANX_musp.	0	0.97	0.18	0.429(316)	0.461(715)	−0.132	0.0983
**dan_**	**0**	**1.00**	**8.23e−09**	**0.435(122225)**	**0.442(729960)**	**−0.0272**	**0.0047**

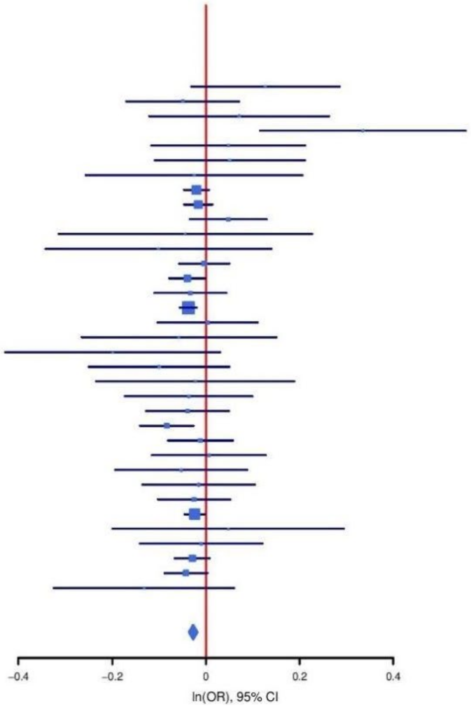

Supplementary Figure 37: Regional association plot (A) and forest plot (B) of SNP rs9534593. See full figure description on page 30.

A

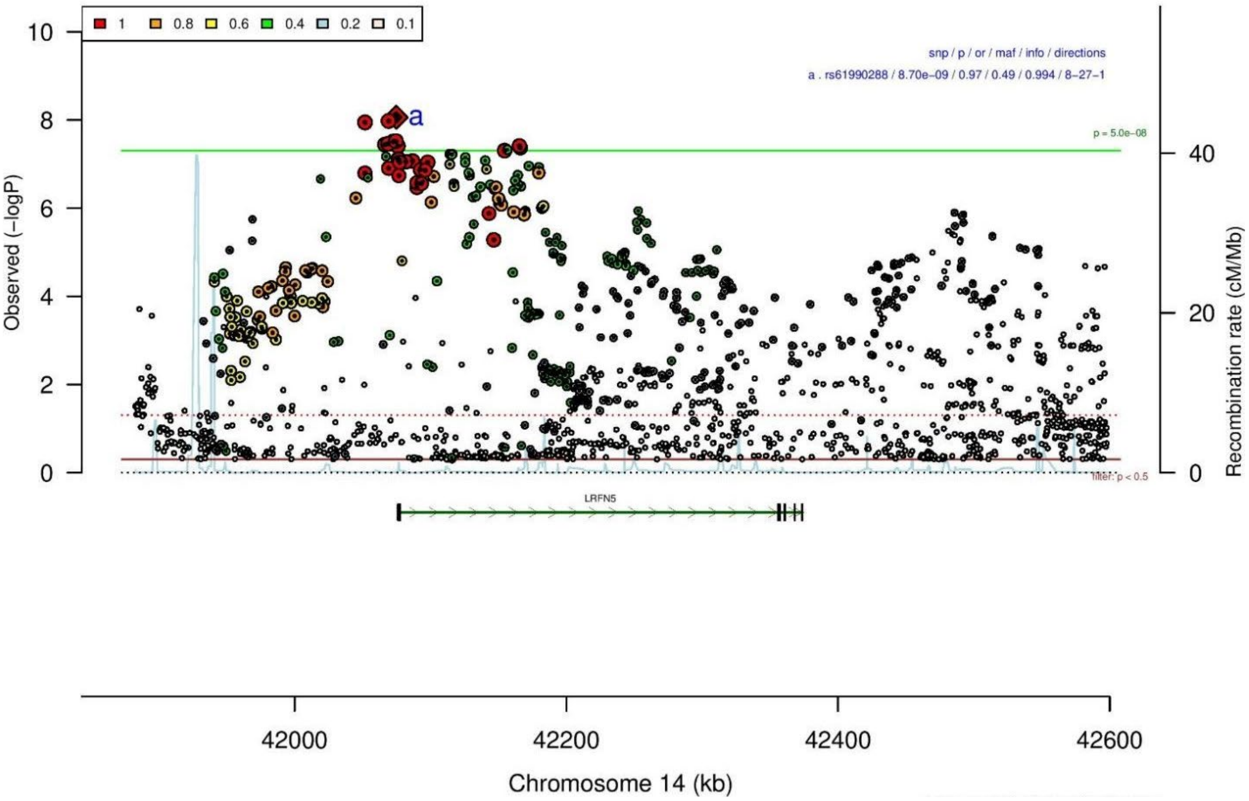

B

rs61990288		A/G		14:42074726			
---?+---+-----+-----+-+-+-----++----				het_P:	0.645	het_I:	0.0
	ngt	info	p_value	f_ca(n)	f_co(n)	ln(OR)	STDerr
dan_daner_ANX_ANGST_gsk.	0	0.97	0.654	0.509(852)	0.509(1103)	−0.0307	0.0686
dan_daner_ANX_ANGST_mgs.	0	1.00	0.589	0.506(557)	0.506(779)	−0.0443	0.082
dan_daner_ANX_ANGST_rs1.	0	1.00	0.899	0.520(331)	0.520(1629)	0.013	0.099
dan_daner_ANX_ANGST_rs2.	0	1.00	0.993	0.514(209)	0.514(1023)	−0.001	0.107
dan_daner_ANX_ANGST_rs3.	0	1.00	0.292	0.511(370)	0.511(1817)	−0.0864	0.082
dan_daner_ANX_ANGST_ship.	0	0.99	0.614	0.498(545)	0.498(834)	−0.0414	0.0821
dan_daner_ANX_ANGST_trails.	0	0.97	0.18	0.535(278)	0.535(336)	0.169	0.126
dan_daner_ANX_EstBB.	0	0.98	0.0541	0.466(12523)	0.466(134954)	−0.0261	0.0136
dan_daner_ANX_FinnGen.	0	1.00	0.00953	0.479(9182)	0.479(160108)	−0.04	0.0154
dan_daner_ANX_GSC.	0	1.00	0.0907	0.492(1306)	0.513(12675)	−0.0712	0.0421
dan_daner_ANX_Gedi_CHDS.	0	1.00	0.0923	0.523(244)	0.523(217)	−0.232	0.138
dan_daner_ANX_Gedi_GSMS.	0	1.00	0.315	0.519(208)	0.519(551)	−0.119	0.118
dan_daner_ANX_Gedi_VTSABD.	0	1.00	0.0804	0.536(224)	0.536(406)	0.0473	0.027
dan_daner_ANX_HUNT.	0	0.99	0.404	0.479(6587)	0.480(40740)	−0.0162	0.0194
dan_daner_ANX_MGHI.	0	0.97	0.617	0.485(1823)	0.489(5151)	−0.0197	0.0393
dan_daner_ANX_MVP.	0	1.00	0.000794	0.495(28525)	0.495(163731)	−0.0317	0.0094
dan_daner_ANX_MoBa.	0	1.00	0.91	0.479(715)	0.479(22168)	−0.00607	0.0537
dan_daner_ANX_PANIC_dca1.	0	0.94	0.867	0.517(248)	0.509(970)	0.0174	0.104
dan_daner_ANX_PANIC_gmdc.	0	1.06	0.733	0.500(247)	0.504(537)	−0.0373	0.109
dan_daner_ANX_PANIC_gom3.	0	0.97	0.516	0.515(472)	0.505(1803)	0.0492	0.0756
dan_daner_ANX_PANIC_gwc3.	0	1.03	0.51	0.463(280)	0.500(855)	−0.0672	0.102
dan_daner_ANX_PANIC_swdf.	0	0.97	0.106	0.465(561)	0.493(2591)	−0.111	0.0688
dan_daner_ANX_PBK1.	0	1.00	0.754	0.488(1121)	0.493(11897)	−0.014	0.0446
dan_daner_ANX_QIMR_AGDS.	0	0.98	0.379	0.497(7312)	0.497(12813)	−0.0255	0.029
dan_daner_ANX_QIMR_Ad.	0	0.98	0.815	0.502(2449)	0.502(6343)	−0.00832	0.0356
dan_daner_ANX_QIMR_Nuup.	0	0.98	0.941	0.481(1131)	0.481(1233)	0.00466	0.0633
dan_daner_ANX_SWE_Catss.	0	1.00	0.0474	0.497(540)	0.497(2333)	0.14	0.0705
dan_daner_ANX_SWE_Salty.	0	1.00	0.296	0.499(687)	0.499(3445)	−0.0644	0.0616
dan_daner_ANX_SWE_TwinGene.	0	1.00	0.683	0.497(1740)	0.497(6470)	−0.0159	0.0389
dan_daner_ANX_UKBB.	0	1.00	0.000907	0.514(25453)	0.514(58113)	−0.0369	0.0111
dan_daner_ANX_Utah.	0	0.96	0.622	0.492(258)	0.509(279)	−0.0618	0.126
dan_daner_ANX_alspac.	0	1.00	0.983	0.508(536)	0.510(3404)	−0.00146	0.0666
dan_daner_ANX_bioVU.	0	1.00	0.282	0.498(7586)	0.498(50545)	−0.02	0.0186
dan_daner_ANX_iPSYCH.	0	0.99	0.0944	0.509(5288)	0.517(14422)	−0.0387	0.0231
dan_daner_ANX_musp.	0	1.00	0.126	0.525(316)	0.483(715)	0.148	0.0966
**dan_**	**0**	**0.99**	**8.7e−09**	**0.495(122225)**	**0.488(729960)**	**−0.027**	**0.0047**

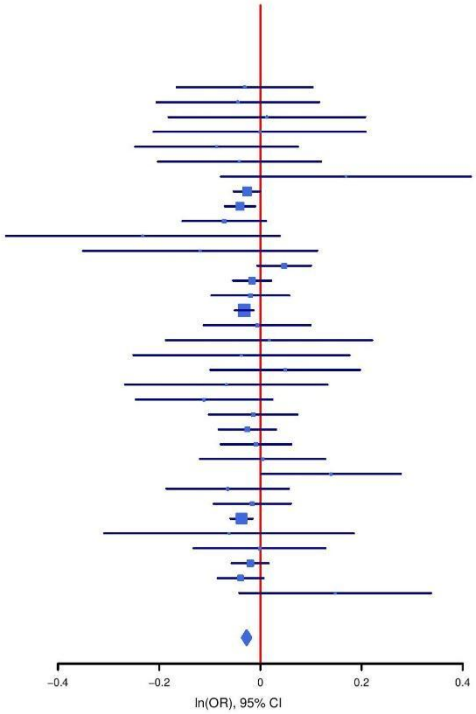

Supplementary Figure 38: Regional association plot (A) and forest plot (B) of SNP rs61990288. See full figure description on page 30.

A

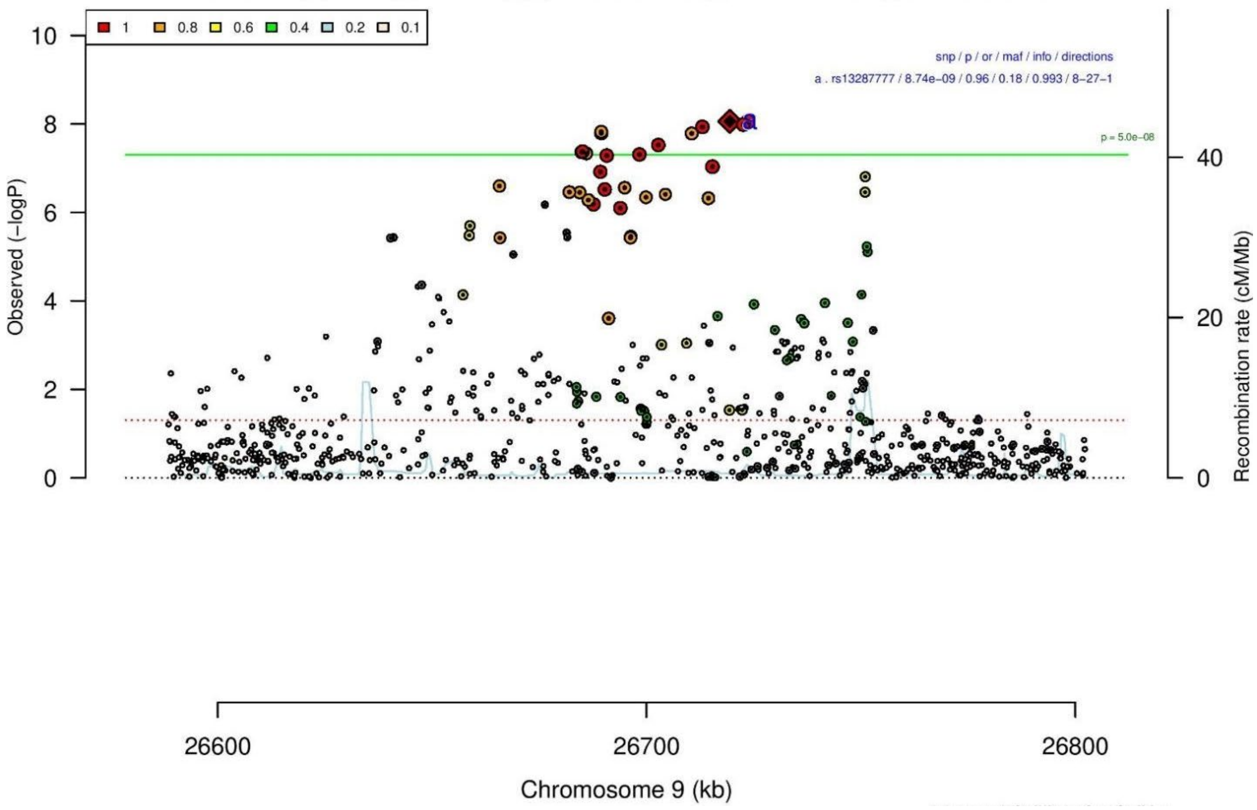

B

rs13287777 **T/G** **9:26719411**

−−+−+−−−−−−−−+−−−−−−+?−+−−−+−−+−−−−+

het_P: 0.36 het_I: 6.4

	ngt	info	p_value	f_ca(n)	f_co(n)	ln(OR)	STDerr
dan_daner_ANX_ANGST_gsk.	0	0.98	0.519	0.200(852)	0.200(1103)	−0.0538	0.0834
dan_daner_ANX_ANGST_mgs.	0	1.00	0.206	0.187(557)	0.187(779)	0.132	0.105
dan_daner_ANX_ANGST_ntr.	0	0.99	0.491	0.187(1521)	0.187(2970)	−0.0541	0.0785
dan_daner_ANX_ANGST_rs1.	0	1.00	0.494	0.185(331)	0.185(1629)	0.084	0.123
dan_daner_ANX_ANGST_rs2.	0	0.99	0.207	0.180(209)	0.180(1023)	−0.191	0.151
dan_daner_ANX_ANGST_rs3.	0	1.00	0.554	0.185(370)	0.185(1817)	−0.0622	0.105
dan_daner_ANX_ANGST_ship.	0	0.99	0.421	0.195(545)	0.195(834)	−0.082	0.102
dan_daner_ANX_ANGST_trails.	0	0.99	0.1	0.187(278)	0.187(336)	−0.247	0.15
dan_daner_ANX_EstBB.	0	0.99	0.0346	0.183(12523)	0.183(134954)	−0.037	0.0175
dan_daner_ANX_FinnGen.	0	0.99	0.00124	0.171(9182)	0.171(160108)	−0.0671	0.0208
dan_daner_ANX_GSC.	0	1.00	0.385	0.171(1306)	0.177(12675)	−0.0477	0.0549
dan_daner_ANX_Gedi_CHDS.	0	1.00	0.00712	0.183(244)	0.183(217)	−0.498	0.185
dan_daner_ANX_Gedi_GSMS.	0	1.00	0.417	0.216(208)	0.216(551)	0.115	0.142
dan_daner_ANX_Gedi_VTSABD.	0	1.00	0.586	0.199(224)	0.199(406)	−0.0194	0.0356
dan_daner_ANX_HUNT.	0	1.00	0.0457	0.176(6587)	0.180(40740)	−0.0504	0.0253
dan_daner_ANX_MGHI.	0	0.96	0.105	0.174(1823)	0.186(5151)	−0.0838	0.0518
dan_daner_ANX_MoBa.	0	1.00	0.697	0.183(715)	0.190(22168)	−0.0269	0.069
dan_daner_ANX_PANIC_dca1.	0	0.96	0.0118	0.164(248)	0.212(970)	−0.346	0.137
dan_daner_ANX_PANIC_gmdc.	0	0.98	0.655	0.221(247)	0.211(537)	0.0614	0.137
dan_daner_ANX_PANIC_gom3.	0	0.99	0.96	0.189(472)	0.190(1803)	−0.00481	0.0952
dan_daner_ANX_PANIC_gwc3.	0	1.03	0.7	0.182(280)	0.192(855)	−0.0512	0.133
dan_daner_ANX_PANIC_swdf.	0	0.97	0.0925	0.156(561)	0.175(2591)	−0.16	0.0951
dan_daner_ANX_PBK1.	0	1.00	0.265	0.195(1121)	0.183(11897)	0.063	0.0561
dan_daner_ANX_QIMR_AGDS.	0	0.98	0.415	0.182(7312)	0.182(12813)	−0.0301	0.0369
dan_daner_ANX_QIMR_Ad.	0	0.98	0.911	0.179(2449)	0.179(6343)	−0.00519	0.0462
dan_daner_ANX_QIMR_Nuup.	0	0.98	0.911	0.180(1131)	0.180(1233)	0.00908	0.0809
dan_daner_ANX_SWE_Catss.	0	1.00	0.0232	0.173(540)	0.173(2333)	−0.219	0.0965
dan_daner_ANX_SWE_Salty.	0	1.00	0.2	0.183(687)	0.183(3445)	−0.103	0.0801
dan_daner_ANX_SWE_TwinGene.	0	1.00	0.73	0.174(1740)	0.174(6470)	−0.0175	0.0508
dan_daner_ANX_UKBB.	0	0.99	0.00177	0.182(25453)	0.182(58113)	−0.0457	0.0146
dan_daner_ANX_Utah.	0	1.00	0.641	0.187(258)	0.178(279)	0.074	0.159
dan_daner_ANX_alspac.	0	1.00	0.292	0.173(536)	0.184(3404)	−0.0907	0.0867
dan_daner_ANX_bioVU.	0	1.00	0.814	0.185(7586)	0.185(50545)	−0.00566	0.0241
dan_daner_ANX_iPSYCH.	0	0.98	0.299	0.192(5288)	0.197(14422)	−0.0306	0.0295
dan_daner_ANX_musp.	0	1.02	0.997	0.188(316)	0.186(715)	0.0005	0.122
**dan_**	**0**	**0.99**	**8.74e−09**	**0.181(122225)**	**0.180(729960)**	**−0.0405**	**0.007**

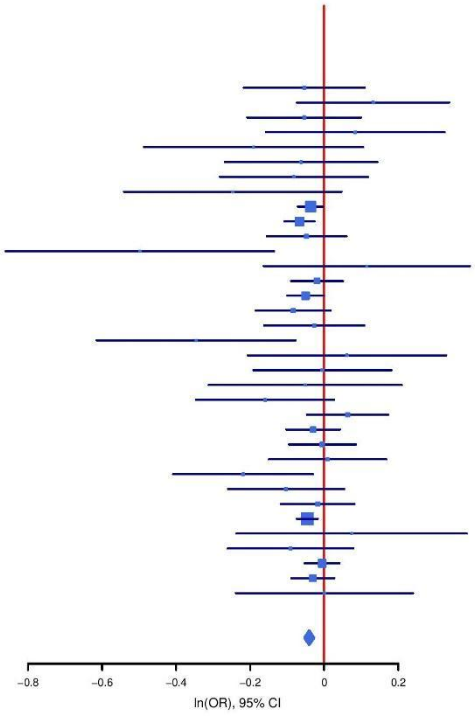

Supplementary Figure 39: Regional association plot (A) and forest plot (B) of SNP rs13287777. See full figure description on page 30.

A

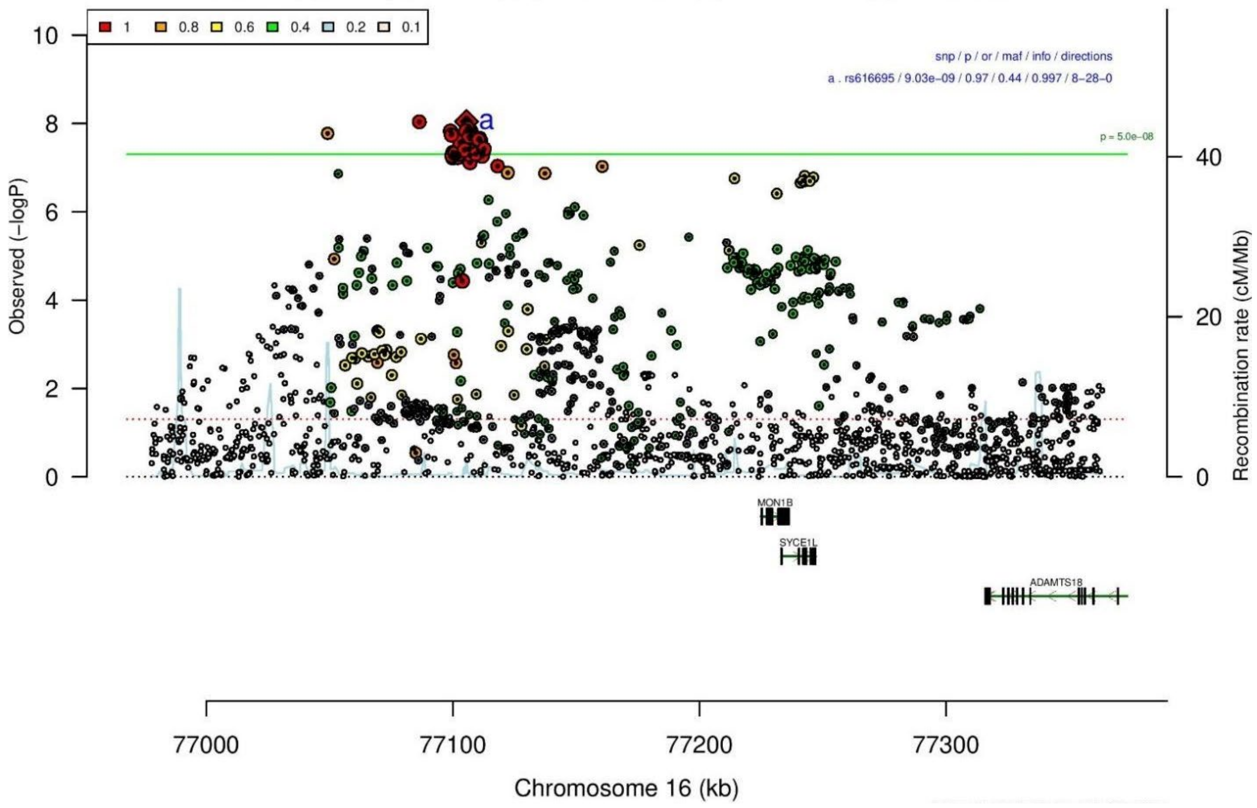

B

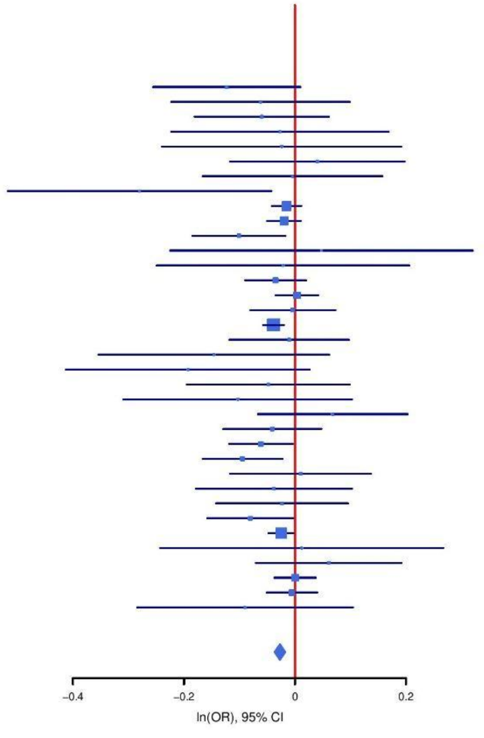

rs616695		T/G		16:77105587			
				het_P:	0.482	het_I:	0.0
	ngt	info	p_value	f_ca(n)	f_co(n)	ln(OR)	STDerr
dan_daner_ANX_ANGST_gsk.	0	0.99	0.0683	0.424(852)	0.424(1103)	−0.123	0.0677
dan_daner_ANX_ANGST_mgs.	0	1.00	0.45	0.421(557)	0.421(779)	−0.0619	0.082
dan_daner_ANX_ANGST_ntr.	0	0.98	0.331	0.431(1521)	0.431(2970)	−0.0599	0.0616
dan_daner_ANX_ANGST_rs1.	0	0.99	0.788	0.422(331)	0.422(1629)	−0.027	0.1
dan_daner_ANX_ANGST_rs2.	0	0.99	0.825	0.416(209)	0.416(1023)	−0.024	0.11
dan_daner_ANX_ANGST_rs3.	0	0.99	0.613	0.421(370)	0.421(1817)	0.0405	0.0801
dan_daner_ANX_ANGST_ship.	0	0.99	0.959	0.428(545)	0.428(834)	−0.00421	0.0825
dan_daner_ANX_ANGST_trails.	0	0.99	0.0203	0.410(278)	0.410(336)	−0.28	0.121
dan_daner_ANX_EstBB.	0	0.99	0.261	0.469(12523)	0.469(134954)	−0.0151	0.0134
dan_daner_ANX_FinnGen.	0	1.00	0.2	0.472(9182)	0.472(160108)	−0.0197	0.0154
dan_daner_ANX_GSC.	0	1.00	0.0177	0.393(1306)	0.419(12675)	−0.101	0.0428
dan_daner_ANX_Gedi_CHDS.	0	1.00	0.732	0.433(244)	0.433(217)	0.0476	0.139
dan_daner_ANX_Gedi_GSMS.	0	1.00	0.855	0.430(208)	0.430(551)	−0.0213	0.116
dan_daner_ANX_Gedi_VTSABD.	0	1.00	0.212	0.434(224)	0.434(406)	−0.0349	0.0279
dan_daner_ANX_HUNT.	0	1.00	0.853	0.435(6587)	0.436(40740)	0.00362	0.0195
dan_daner_ANX_MGHI.	0	1.01	0.923	0.419(1823)	0.419(5151)	−0.00379	0.039
dan_daner_ANX_MVP.	0	1.00	5.29e−05	0.424(28525)	0.424(163731)	−0.0386	0.0095
dan_daner_ANX_MoBa.	0	1.00	0.853	0.429(715)	0.431(22168)	−0.0102	0.0548
dan_daner_ANX_PANIC_dca1.	0	0.94	0.171	0.410(248)	0.442(970)	−0.146	0.106
dan_daner_ANX_PANIC_gmdc.	0	1.02	0.0857	0.422(247)	0.465(537)	−0.193	0.112
dan_daner_ANX_PANIC_gom3.	0	1.02	0.52	0.427(472)	0.437(1803)	−0.048	0.0748
dan_daner_ANX_PANIC_gwc3.	0	1.03	0.328	0.401(280)	0.431(855)	−0.103	0.105
dan_daner_ANX_PANIC_swdf.	0	0.99	0.323	0.452(561)	0.444(2591)	0.0681	0.0689
dan_daner_ANX_PBK1.	0	1.00	0.365	0.405(1121)	0.415(11897)	−0.0408	0.045
dan_daner_ANX_QIMR_AGDS.	0	0.98	0.0349	0.417(7312)	0.417(12813)	−0.0614	0.0291
dan_daner_ANX_QIMR_Ad.	0	0.98	0.00982	0.410(2449)	0.410(6343)	−0.0946	0.0366
dan_daner_ANX_QIMR_Nuup.	0	0.98	0.872	0.415(1131)	0.415(1233)	0.0104	0.0648
dan_daner_ANX_SWE_Catss.	0	1.00	0.597	0.457(540)	0.457(2333)	−0.0378	0.0715
dan_daner_ANX_SWE_Salty.	0	1.00	0.699	0.438(687)	0.438(3445)	−0.0234	0.0605
dan_daner_ANX_SWE_TwinGene.	0	1.00	0.0414	0.438(1740)	0.438(6470)	−0.0805	0.0395
dan_daner_ANX_UKBB.	0	1.00	0.0271	0.419(25453)	0.419(58113)	−0.025	0.0113
dan_daner_ANX_Utah.	0	0.90	0.925	0.436(258)	0.432(279)	0.0123	0.13
dan_daner_ANX_alspac.	0	1.00	0.362	0.441(536)	0.424(3404)	0.0609	0.0668
dan_daner_ANX_bioVU.	0	1.00	0.985	0.421(7586)	0.421(50545)	0.000366	0.0189
dan_daner_ANX_iPSYCH.	0	1.00	0.812	0.429(5288)	0.430(14422)	−0.0055	0.0232
dan_daner_ANX_musp.	0	0.98	0.363	0.404(316)	0.424(715)	−0.0901	0.0991
**dan_**	**0**	**1.00**	**9.03e−09**	**0.431(122225)**	**0.443(729960)**	**−0.0271**	**0.0047**

Supplementary Figure 40: Regional association plot (A) and forest plot (B) of SNP rs616695. See full figure description on page 30.

A

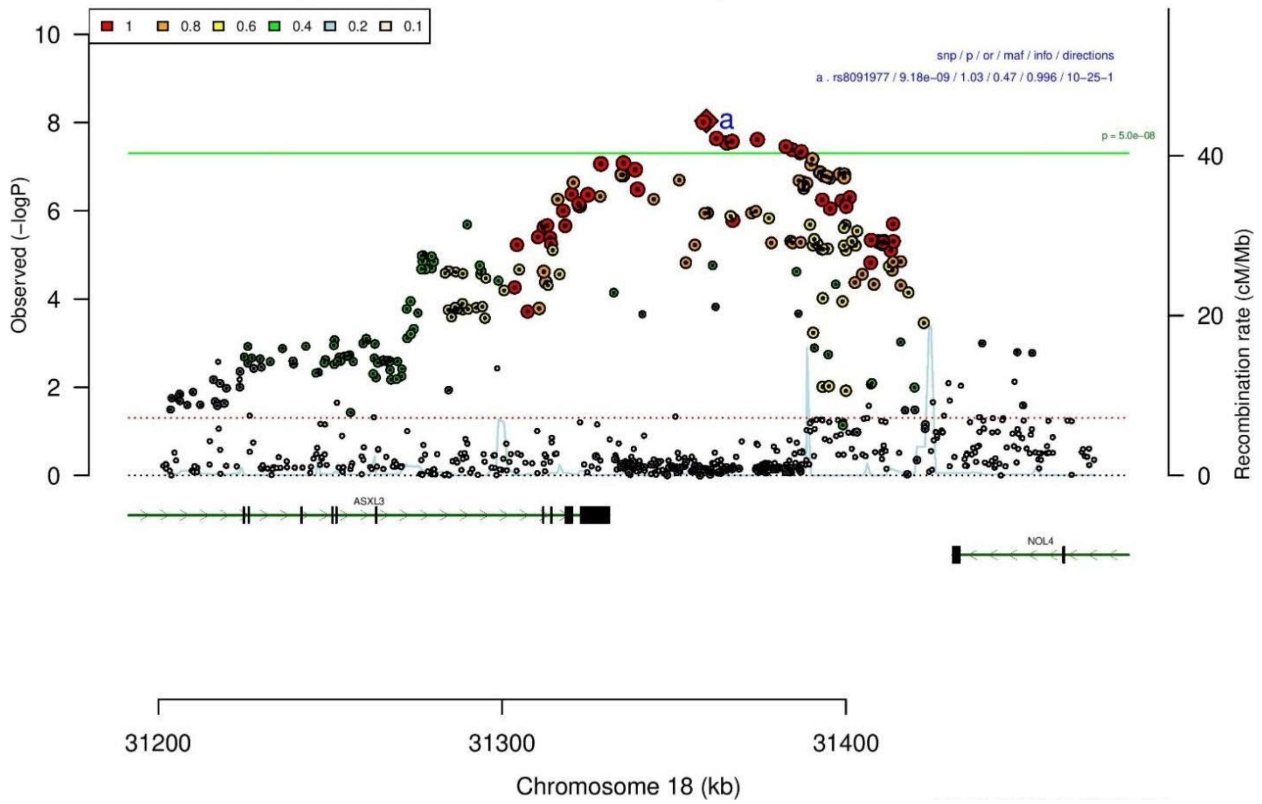

B

rs8091977 +?+---+-------+---+---+---+---+-++----		C/T		18:31359414 het_P:	0.144	het_I:	20.5
	**ngt**	**info**	**p_value**	**f_ca(n)**	**f_co(n)**	**ln(OR)**	**STDerr**
dan_daner_ANX_ANGST_mgs.	0	1.00	0.474	0.495(557)	0.495(779)	−0.0588	0.0822
dan_daner_ANX_ANGST_ntr.	0	0.99	0.082	0.488(1521)	0.488(2970)	0.106	0.0612
dan_daner_ANX_ANGST_rs1.	0	0.99	0.086	0.478(331)	0.478(1629)	0.168	0.098
dan_daner_ANX_ANGST_rs2.	0	0.99	0.177	0.481(209)	0.481(1023)	0.15	0.111
dan_daner_ANX_ANGST_rs3.	0	0.99	0.603	0.485(370)	0.485(1817)	−0.0434	0.0834
dan_daner_ANX_ANGST_ship.	0	1.00	0.36	0.493(545)	0.493(834)	0.0751	0.082
dan_daner_ANX_ANGST_trails.	0	0.99	0.301	0.479(278)	0.479(336)	0.124	0.12
dan_daner_ANX_EstBB.	0	0.98	0.00103	0.501(12523)	0.501(134954)	0.0443	0.0135
dan_daner_ANX_FinnGen.	0	1.00	0.25	0.530(9182)	0.530(160108)	0.0177	0.0154
dan_daner_ANX_GSC.	0	1.01	0.121	0.500(1306)	0.484(12675)	0.0647	0.0417
dan_daner_ANX_Gedi_CHDS.	0	1.00	0.674	0.487(244)	0.487(217)	0.0565	0.134
dan_daner_ANX_Gedi_GSMS.	0	1.00	0.17	0.505(208)	0.505(551)	−0.166	0.121
dan_daner_ANX_Gedi_VTSABD.	0	1.00	0.984	0.504(224)	0.504(406)	0.000542	0.0275
dan_daner_ANX_HUNT.	0	1.00	0.696	0.498(6587)	0.496(40740)	0.00752	0.0193
dan_daner_ANX_MGHI.	0	1.00	0.508	0.511(1823)	0.504(5151)	0.0257	0.0388
dan_daner_ANX_MVP.	0	1.00	0.0163	0.328(28525)	0.328(163731)	0.028	0.0117
dan_daner_ANX_MoBa.	0	1.00	0.336	0.527(715)	0.514(22168)	0.052	0.0541
dan_daner_ANX_PANIC_dca1.	0	0.98	0.495	0.514(248)	0.495(970)	0.07	0.102
dan_daner_ANX_PANIC_gmdc.	0	1.03	0.0556	0.521(247)	0.473(537)	0.212	0.111
dan_daner_ANX_PANIC_gom3.	0	1.00	0.962	0.471(472)	0.469(1803)	−0.00349	0.0748
dan_daner_ANX_PANIC_gwc3.	0	0.99	0.5	0.494(280)	0.468(855)	0.0714	0.106
dan_daner_ANX_PANIC_swdf.	0	0.98	0.00712	0.542(561)	0.502(2591)	0.185	0.0688
dan_daner_ANX_PBK1.	0	1.00	0.0769	0.518(1121)	0.500(11897)	0.079	0.0447
dan_daner_ANX_QIMR_AGDS.	0	0.97	0.732	0.497(7312)	0.497(12813)	0.00991	0.0289
dan_daner_ANX_QIMR_Ad.	0	0.97	0.967	0.500(2449)	0.500(6343)	−0.00149	0.0358
dan_daner_ANX_QIMR_Nuup.	0	0.97	0.346	0.508(1131)	0.508(1233)	−0.059	0.0625
dan_daner_ANX_SWE_Catss.	0	1.00	0.757	0.496(540)	0.496(2333)	−0.0222	0.0716
dan_daner_ANX_SWE_Salty.	0	1.00	0.249	0.479(687)	0.479(3445)	0.0685	0.0593
dan_daner_ANX_SWE_TwinGene.	0	1.00	0.568	0.490(1740)	0.490(6470)	0.0222	0.0388
dan_daner_ANX_UKBB.	0	1.00	1.78e−05	0.498(25453)	0.498(58113)	0.0479	0.0112
dan_daner_ANX_Utah.	0	0.96	0.71	0.521(258)	0.509(279)	0.0465	0.125
dan_daner_ANX_alspac.	0	1.00	0.795	0.493(536)	0.498(3404)	−0.0173	0.0666
dan_daner_ANX_bioVU.	0	1.00	0.223	0.497(7586)	0.497(50545)	0.0228	0.0187
dan_daner_ANX_iPSYCH.	0	0.99	0.0608	0.504(5288)	0.514(14422)	−0.0434	0.0232
dan_daner_ANX_musp.	0	0.96	0.718	0.483(316)	0.490(715)	−0.0353	0.0977
**dan_**	**0**	**1.00**	**9.18e−09**	**0.461(122225)**	**0.467(729960)**	**0.0282**	**0.0049**

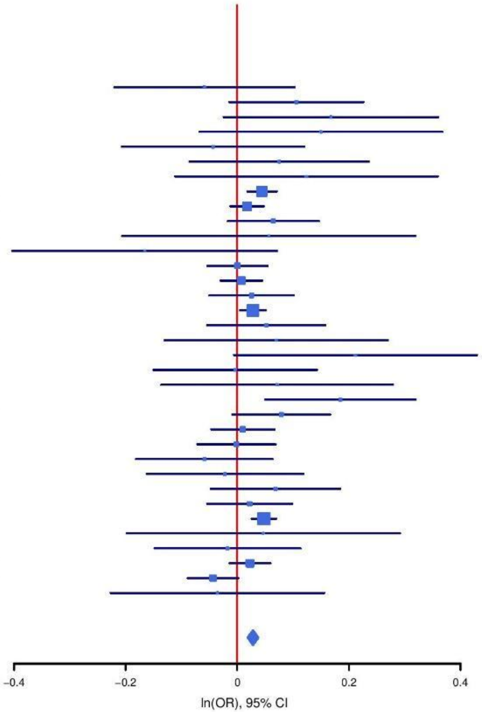

Supplementary Figure 41: Regional association plot (A) and forest plot (B) of SNP rs8091977. See full figure description on page 30.

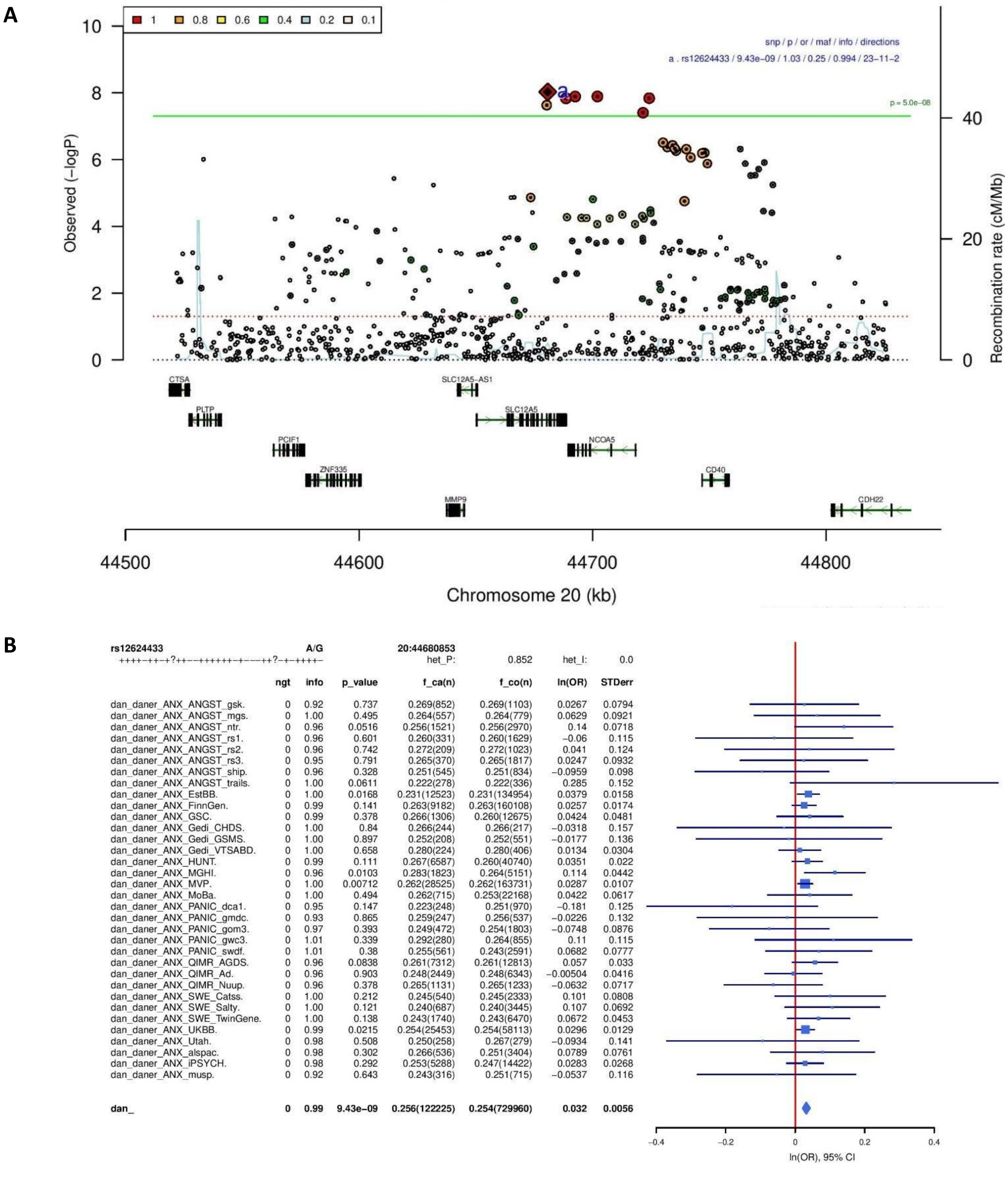

Supplementary Figure 42: Regional association plot (A) and forest plot (B) of SNP rs12624433. See full figure description on page 30.

A

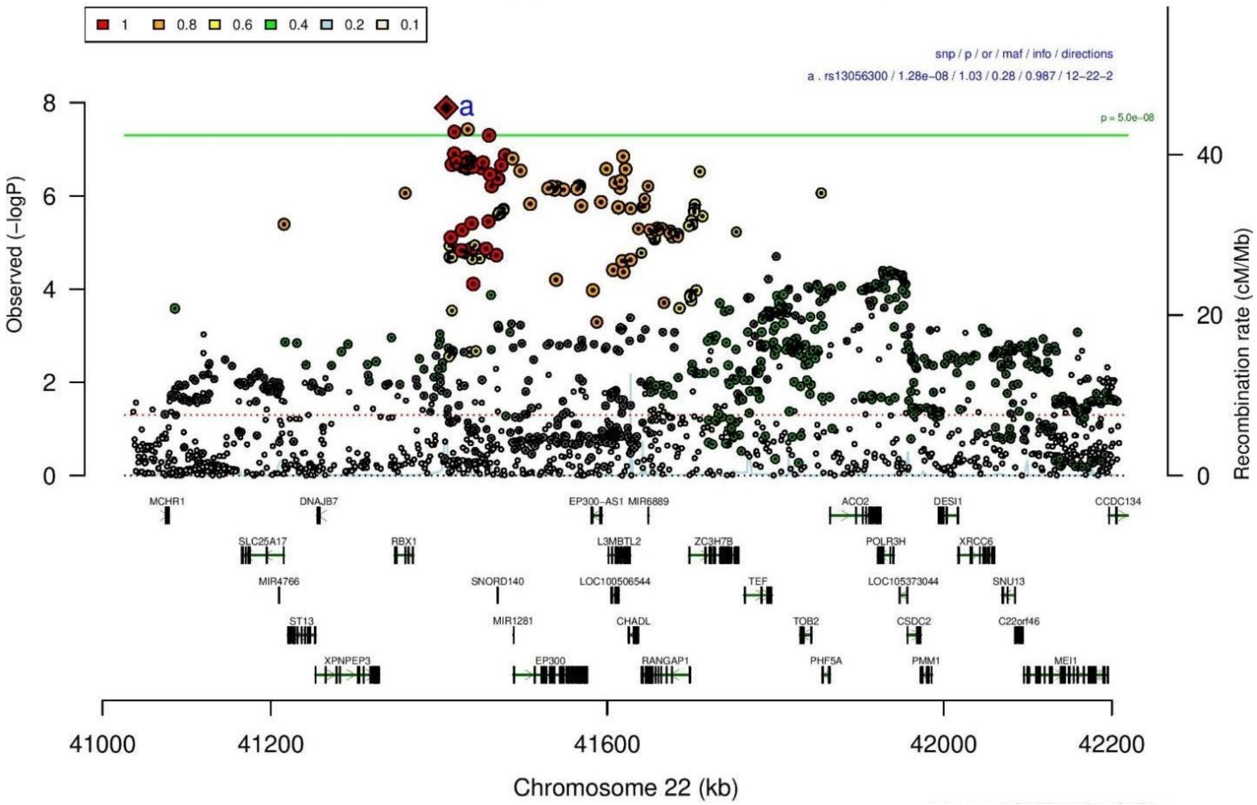

B

rs13056300		C/T		22:41408754			
−−+−−−+−+?−−+−−++−++−−−+−++?−−+−−−−−				het_P:	0.0506	het_I:	30.3
	**ngt**	**info**	**p_value**	**f_ca(n)**	**f_co(n)**	**ln(OR)**	**STDerr**
dan_daner_ANX_ANGST_gsk.	0	0.85	0.649	0.257(852)	0.257(1103)	0.0382	0.0839
dan_daner_ANX_ANGST_mgs.	0	1.00	0.244	0.275(557)	0.275(779)	−0.111	0.095
dan_daner_ANX_ANGST_ntr.	0	0.84	0.0283	0.267(1521)	0.267(2970)	0.165	0.0751
dan_daner_ANX_ANGST_rs1.	0	0.94	0.139	0.264(331)	0.264(1629)	0.166	0.112
dan_daner_ANX_ANGST_rs2.	0	0.93	0.908	0.260(209)	0.260(1023)	0.015	0.126
dan_daner_ANX_ANGST_rs3.	0	0.94	0.582	0.261(370)	0.261(1817)	−0.0521	0.0946
dan_daner_ANX_ANGST_ship.	0	0.95	0.809	0.255(545)	0.255(834)	0.0229	0.0952
dan_daner_ANX_ANGST_trails.	0	0.95	0.585	0.287(278)	0.287(336)	−0.0768	0.141
dan_daner_ANX_EstBB.	0	0.98	0.00861	0.264(12523)	0.264(134954)	0.0401	0.0153
dan_daner_ANX_FinnGen.	0	0.99	0.00104	0.309(9182)	0.309(160108)	0.054	0.0165
dan_daner_ANX_GSC.	0	0.98	0.505	0.279(1306)	0.285(12675)	−0.0313	0.047
dan_daner_ANX_Gedi_CHDS.	0	1.00	0.991	0.254(244)	0.254(217)	−0.00178	0.162
dan_daner_ANX_Gedi_GSMS.	0	1.00	0.117	0.276(208)	0.276(551)	0.208	0.133
dan_daner_ANX_Gedi_VTSABD.	0	1.00	0.98	0.257(224)	0.257(406)	0.000771	0.0301
dan_daner_ANX_HUNT.	0	0.99	0.484	0.285(6587)	0.287(40740)	−0.015	0.0214
dan_daner_ANX_MGHI.	0	0.95	0.587	0.277(1823)	0.282(5151)	−0.0241	0.0443
dan_daner_ANX_MVP.	0	1.00	0.0025	0.263(28525)	0.263(163731)	0.0338	0.0112
dan_daner_ANX_MoBa.	0	1.00	0.786	0.263(715)	0.265(22168)	−0.0165	0.0609
dan_daner_ANX_PANIC_dca1.	0	0.95	0.257	0.267(248)	0.246(970)	0.134	0.118
dan_daner_ANX_PANIC_gmdc.	0	0.93	0.17	0.252(247)	0.283(537)	−0.183	0.133
dan_daner_ANX_PANIC_gom3.	0	0.89	0.788	0.258(472)	0.258(1803)	0.0243	0.0902
dan_daner_ANX_PANIC_gwc3.	0	0.99	0.602	0.259(280)	0.264(855)	−0.0626	0.12
dan_daner_ANX_PANIC_swdf.	0	0.97	0.00344	0.252(561)	0.287(2591)	−0.233	0.0797
dan_daner_ANX_QIMR_AGDS.	0	0.97	0.0102	0.266(7312)	0.266(12813)	0.0844	0.0328
dan_daner_ANX_QIMR_Ad.	0	0.97	0.292	0.272(2449)	0.272(6343)	0.0432	0.041
dan_daner_ANX_QIMR_Nuup.	0	0.97	0.675	0.259(1131)	0.259(1233)	−0.03	0.0717
dan_daner_ANX_SWE_Catss.	0	1.00	0.262	0.270(540)	0.270(2333)	0.089	0.0794
dan_daner_ANX_SWE_Salty.	0	1.00	0.0921	0.274(687)	0.274(3445)	0.116	0.0686
dan_daner_ANX_SWE_TwinGene.	0	1.00	0.57	0.267(1740)	0.267(6470)	0.0247	0.0435
dan_daner_ANX_UKBB.	0	0.98	0.00282	0.273(25453)	0.273(58113)	0.0375	0.0126
dan_daner_ANX_Utah.	0	0.98	0.288	0.329(258)	0.296(279)	0.143	0.135
dan_daner_ANX_alspac.	0	0.98	0.0311	0.294(536)	0.265(3404)	0.161	0.0743
dan_daner_ANX_iPSYCH.	0	0.96	0.713	0.257(5288)	0.255(14422)	0.0099	0.027
dan_daner_ANX_musp.	0	0.95	0.415	0.274(316)	0.257(715)	0.0906	0.111
**dan_**	**0**	**0.99**	**1.28e−08**	**0.271(122225)**	**0.277(729960)**	**0.0315**	**0.0055**

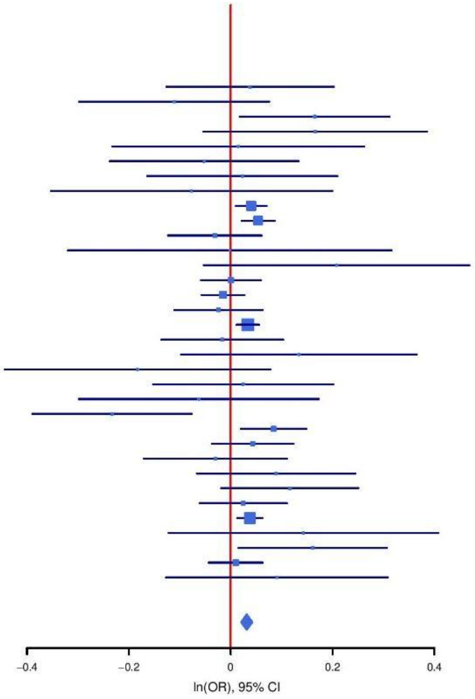

Supplementary Figure 43: Regional association plot (A) and forest plot (B) of SNP rs13056300. See full figure description on page 30.

A

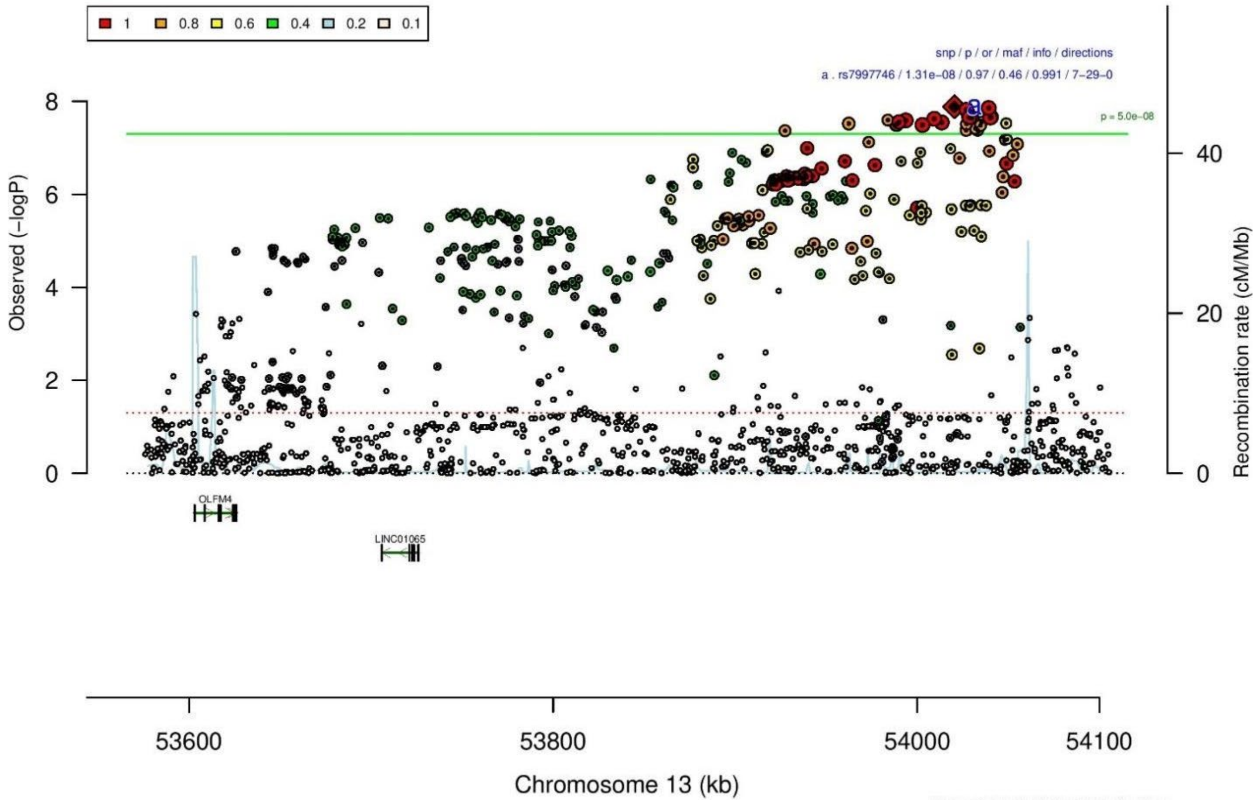

B

rs7997746		A/C		13:54020455			
++-----+-------------------+-----+++---				het_P:	0.674	het_I:	0.0
	ngt	info	p_value	f_ca(n)	f_co(n)	ln(OR)	STDerr
dan_daner_ANX_ANGST_gsk.	0	0.99	0.647	0.447(852)	0.447(1103)	0.0308	0.0673
dan_daner_ANX_ANGST_mgs.	0	1.00	0.4	0.465(557)	0.465(779)	−0.0686	0.0815
dan_daner_ANX_ANGST_ntr.	0	1.00	0.438	0.448(1521)	0.448(2970)	−0.0476	0.0615
dan_daner_ANX_ANGST_rs1.	0	1.00	0.111	0.477(331)	0.477(1629)	−0.153	0.096
dan_daner_ANX_ANGST_rs2.	0	1.00	0.103	0.461(209)	0.461(1023)	−0.178	0.109
dan_daner_ANX_ANGST_rs3.	0	1.00	0.862	0.471(370)	0.471(1817)	−0.0142	0.0817
dan_daner_ANX_ANGST_ship.	0	1.00	0.713	0.432(545)	0.432(834)	0.031	0.0843
dan_daner_ANX_ANGST_trails.	0	0.99	0.81	0.442(278)	0.442(336)	−0.0294	0.123
dan_daner_ANX_EstBB.	0	0.97	0.0058	0.473(12523)	0.473(134954)	−0.0376	0.0136
dan_daner_ANX_FinnGen.	0	1.00	0.907	0.454(9182)	0.454(160108)	−0.00181	0.0155
dan_daner_ANX_GSC.	0	0.99	0.631	0.496(1306)	0.500(12675)	−0.0203	0.0422
dan_daner_ANX_Gedi_CHDS.	0	1.00	0.271	0.467(244)	0.467(217)	−0.151	0.138
dan_daner_ANX_Gedi_GSMS.	0	1.00	0.326	0.456(208)	0.456(551)	−0.122	0.125
dan_daner_ANX_Gedi_VTSABD.	0	1.00	0.0393	0.441(224)	0.441(406)	−0.0589	0.0285
dan_daner_ANX_HUNT.	0	1.00	0.00114	0.436(6587)	0.453(40740)	−0.0637	0.0196
dan_daner_ANX_MGHI.	0	1.00	0.3	0.469(1823)	0.478(5151)	−0.0401	0.0387
dan_daner_ANX_MVP.	0	1.00	0.0684	0.465(28525)	0.465(163731)	−0.0177	0.0097
dan_daner_ANX_MoBa.	0	1.00	0.527	0.444(715)	0.452(22168)	−0.0343	0.0543
dan_daner_ANX_PANIC_dca1.	0	1.02	0.863	0.444(248)	0.450(970)	−0.0175	0.101
dan_daner_ANX_PANIC_gmdc.	0	1.00	0.431	0.467(247)	0.480(537)	−0.0883	0.112
dan_daner_ANX_PANIC_gom3.	0	0.98	0.288	0.449(472)	0.458(1803)	−0.0805	0.0757
dan_daner_ANX_PANIC_gwc3.	0	1.02	0.77	0.420(280)	0.438(855)	0.0303	0.104
dan_daner_ANX_PANIC_swdf.	0	1.02	0.336	0.455(561)	0.467(2591)	−0.0649	0.0675
dan_daner_ANX_PBK1.	0	1.00	0.58	0.472(1121)	0.478(11897)	−0.0247	0.0447
dan_daner_ANX_QIMR_AGDS.	0	0.95	0.244	0.467(7312)	0.467(12813)	−0.034	0.0292
dan_daner_ANX_QIMR_Ad.	0	0.95	0.915	0.466(2449)	0.466(6343)	−0.00385	0.0358
dan_daner_ANX_QIMR_Nuup.	0	0.95	0.564	0.474(1131)	0.474(1233)	0.0361	0.0625
dan_daner_ANX_SWE_Catss.	0	1.00	0.64	0.469(540)	0.469(2333)	0.0329	0.0704
dan_daner_ANX_SWE_Salty.	0	1.00	0.611	0.457(687)	0.457(3445)	0.0306	0.0602
dan_daner_ANX_SWE_TwinGene.	0	1.00	0.0561	0.460(1740)	0.460(6470)	−0.0739	0.0387
dan_daner_ANX_UKBB.	0	0.98	0.00394	0.468(25453)	0.468(58113)	−0.0326	0.0113
dan_daner_ANX_Utah.	0	1.06	0.815	0.451(258)	0.454(279)	−0.0283	0.121
dan_daner_ANX_alspac.	0	1.00	0.0385	0.487(536)	0.455(3404)	0.136	0.0658
dan_daner_ANX_bioVU.	0	1.00	0.85	0.469(7586)	0.469(50545)	−0.00356	0.0188
dan_daner_ANX_iPSYCH.	0	1.00	0.0368	0.447(5288)	0.457(14422)	−0.0482	0.0231
dan_daner_ANX_musp.	0	1.01	0.496	0.464(316)	0.479(715)	−0.0652	0.0957
**dan_**	**0**	**0.99**	**1.31e−08**	**0.463(122225)**	**0.464(729960)**	**−0.027**	**0.0047**

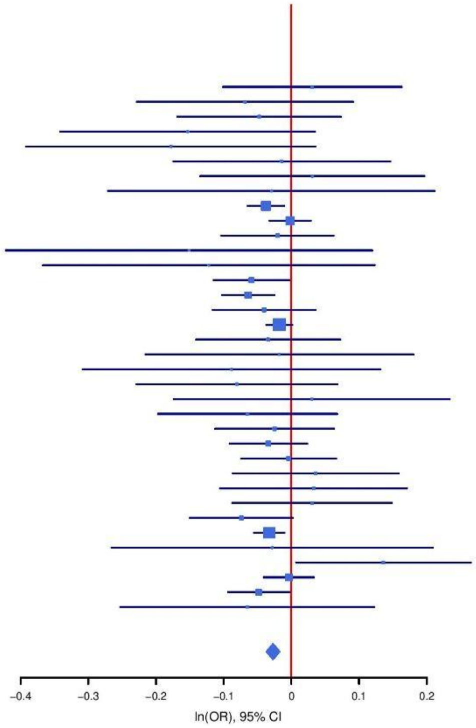

Supplementary Figure 44: Regional association plot (A) and forest plot (B) of SNP rs7997746. See full figure description on page 30.

A

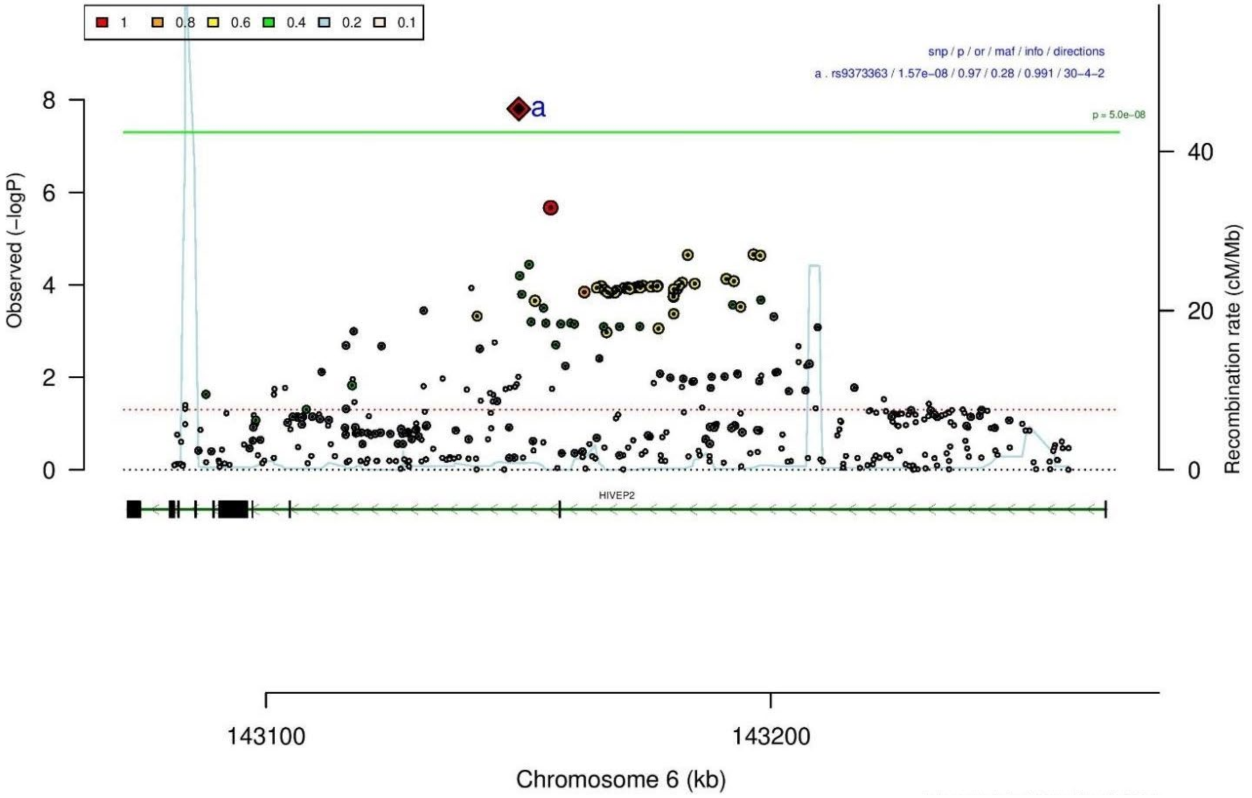

B

rs9373363 G/A 6:143150043

+++++−+++?+++++++++++−−+++?+++++++− het_P: 0.885 het_I: 0.0

	ngt	info	p_value	f_ca(n)	f_co(n)	ln(OR)	STDerr
dan_daner_ANX_ANGST_gsk.	0	0.85	0.79	0.236(852)	0.236(1103)	−0.0223	0.0837
dan_daner_ANX_ANGST_mgs.	0	1.00	0.17	0.247(557)	0.247(779)	−0.131	0.0958
dan_daner_ANX_ANGST_ntr.	0	1.00	0.132	0.271(1521)	0.271(2970)	−0.105	0.0695
dan_daner_ANX_ANGST_rs1.	0	1.00	0.431	0.264(331)	0.264(1629)	−0.09	0.114
dan_daner_ANX_ANGST_rs2.	0	1.00	0.765	0.259(209)	0.259(1023)	0.036	0.122
dan_daner_ANX_ANGST_rs3.	0	1.00	0.265	0.251(370)	0.251(1817)	−0.105	0.0942
dan_daner_ANX_ANGST_ship.	0	0.98	0.425	0.268(545)	0.268(834)	−0.0733	0.0919
dan_daner_ANX_ANGST_trails.	0	0.97	0.0944	0.266(278)	0.266(336)	−0.228	0.136
dan_daner_ANX_EstBB.	0	0.98	0.476	0.278(12523)	0.278(134954)	−0.0108	0.0151
dan_daner_ANX_FinnGen.	0	0.99	0.0389	0.333(9182)	0.333(160108)	−0.0338	0.0164
dan_daner_ANX_GSC.	1	1.01	0.134	0.235(1306)	0.247(12675)	−0.0723	0.0482
dan_daner_ANX_Gedi_CHDS.	0	1.00	0.55	0.261(244)	0.261(217)	−0.0953	0.159
dan_daner_ANX_Gedi_GSMS.	0	1.00	0.675	0.225(208)	0.225(551)	−0.062	0.148
dan_daner_ANX_Gedi_VTSABD.	0	1.00	0.797	0.249(224)	0.249(406)	−0.00814	0.0316
dan_daner_ANX_HUNT.	0	0.99	0.399	0.274(6587)	0.277(40740)	−0.0182	0.0216
dan_daner_ANX_MGHI.	0	0.99	0.331	0.229(1823)	0.237(5151)	−0.0448	0.0461
dan_daner_ANX_MVP.	0	1.00	0.0448	0.246(28525)	0.246(163731)	−0.0236	0.0118
dan_daner_ANX_MoBa.	0	1.00	0.78	0.283(715)	0.287(22168)	−0.0167	0.0597
dan_daner_ANX_PANIC_dca1.	0	1.00	0.587	0.276(248)	0.266(970)	0.0621	0.114
dan_daner_ANX_PANIC_gmdc.	0	0.93	0.219	0.250(247)	0.227(537)	0.168	0.137
dan_daner_ANX_PANIC_gom3.	0	0.98	0.103	0.236(472)	0.263(1803)	−0.144	0.0882
dan_daner_ANX_PANIC_gwc3.	1	1.00	0.256	0.243(280)	0.258(855)	−0.138	0.122
dan_daner_ANX_PANIC_swdf.	0	0.99	0.942	0.301(561)	0.300(2591)	−0.00539	0.0741
dan_daner_ANX_QIMR_AGDS.	0	0.97	0.053	0.250(7312)	0.250(12813)	−0.0644	0.0333
dan_daner_ANX_QIMR_Ad.	0	0.97	0.197	0.250(2449)	0.250(6343)	−0.0547	0.0424
dan_daner_ANX_QIMR_Nuup.	0	0.97	0.443	0.242(1131)	0.242(1233)	−0.0578	0.0753
dan_daner_ANX_SWE_Catss.	0	1.00	0.552	0.271(540)	0.271(2333)	−0.0501	0.0843
dan_daner_ANX_SWE_Salty.	0	1.00	0.0311	0.275(687)	0.275(3445)	−0.159	0.0737
dan_daner_ANX_SWE_TwinGene.	0	1.00	0.352	0.291(1740)	0.291(6470)	−0.0397	0.0426
dan_daner_ANX_UKBB.	0	0.98	0.003	0.255(25453)	0.255(58113)	−0.0385	0.013
dan_daner_ANX_Utah.	0	1.01	0.601	0.252(258)	0.238(279)	0.0743	0.142
dan_daner_ANX_alspac.	0	1.00	0.331	0.235(536)	0.250(3404)	−0.0753	0.0779
dan_daner_ANX_iPSYCH.	0	0.98	0.244	0.265(5288)	0.271(14422)	−0.0307	0.0264
dan_daner_ANX_musp.	0	0.98	0.0563	0.229(316)	0.267(715)	−0.218	0.114
**dan_**	**2**	**0.99**	**1.57e−08**	**0.263(122225)**	**0.279(729960)**	**−0.032**	**0.0057**

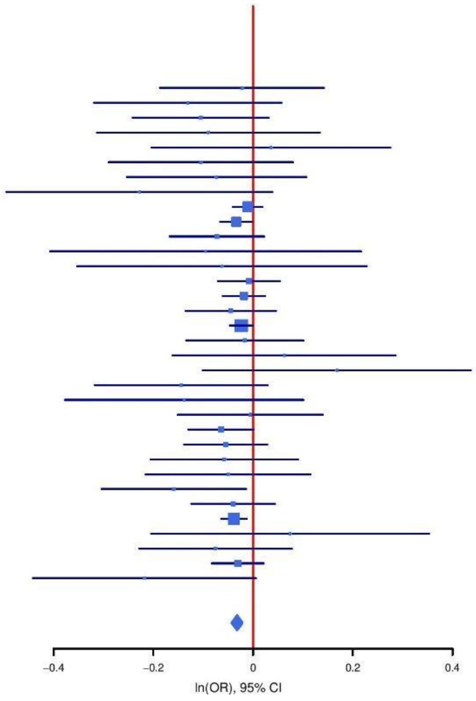

Supplementary Figure 45: Regional association plot (A) and forest plot (B) of SNP rs9373363. See full figure description on page 30.

A

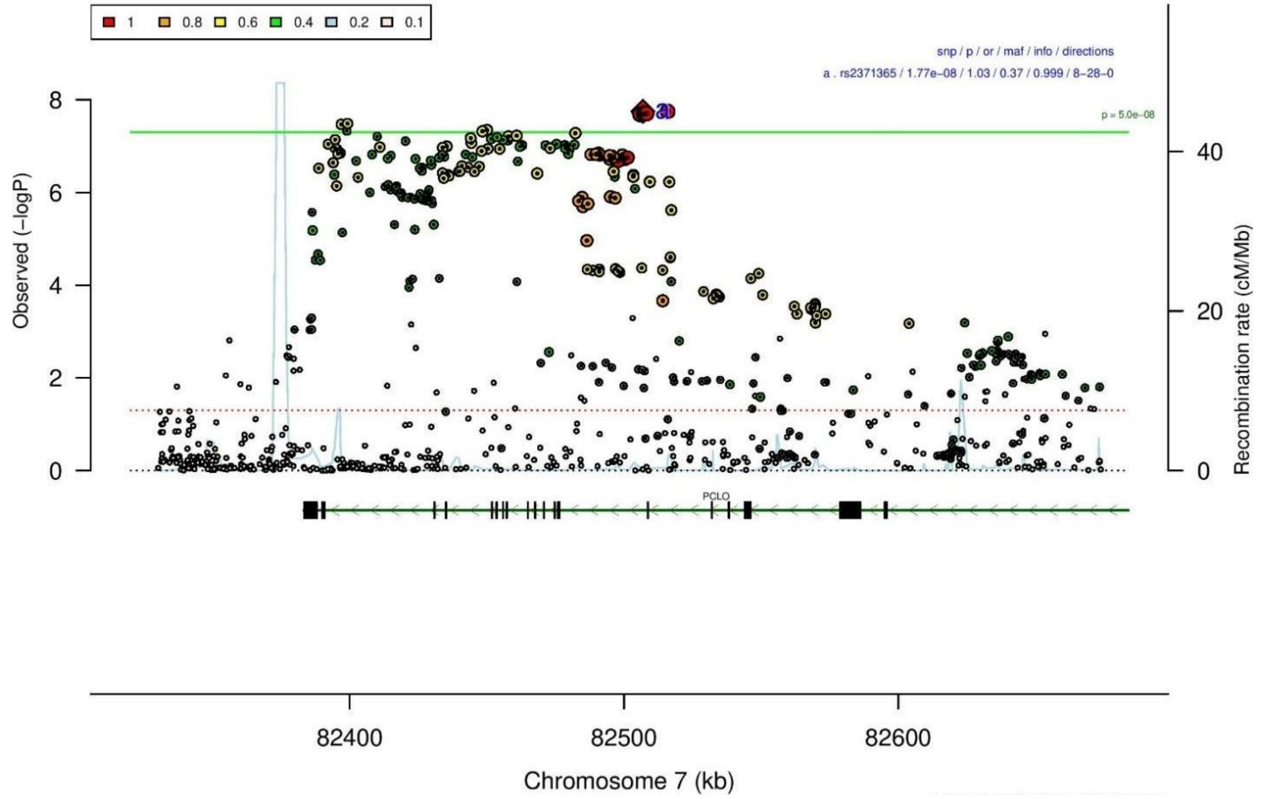

B

rs2371365 **C/T** **7:82506898**

het_P: 0.279 het_I: 11.2

	ngt	info	p_value	f_ca(n)	f_co(n)	ln(OR)	STDerr
dan_daner_ANX_ANGST_gsk.	0	0.94	0.137	0.344(852)	0.344(1103)	0.107	0.0719
dan_daner_ANX_ANGST_mgs.	0	1.00	0.459	0.381(557)	0.381(779)	0.0612	0.0826
dan_daner_ANX_ANGST_ntr.	0	0.99	0.0023	0.394(1521)	0.394(2970)	0.19	0.0625
dan_daner_ANX_ANGST_rs1.	0	1.00	0.202	0.381(331)	0.381(1629)	0.126	0.099
dan_daner_ANX_ANGST_rs2.	0	1.00	0.292	0.382(209)	0.382(1023)	−0.118	0.112
dan_daner_ANX_ANGST_rs3.	0	1.00	0.881	0.386(370)	0.386(1817)	0.0124	0.0827
dan_daner_ANX_ANGST_ship.	0	0.98	0.591	0.377(545)	0.377(834)	−0.0452	0.0843
dan_daner_ANX_ANGST_trails.	0	1.00	0.503	0.402(278)	0.402(336)	−0.0828	0.124
dan_daner_ANX_EstBB.	0	1.00	0.23	0.340(12523)	0.340(134954)	0.0169	0.0141
dan_daner_ANX_FinnGen.	0	1.00	0.0223	0.365(9182)	0.365(160108)	0.0362	0.0159
dan_daner_ANX_GSC.	0	1.00	0.0393	0.401(1306)	0.383(12675)	0.0885	0.0428
dan_daner_ANX_Gedi_CHDS.	0	1.00	0.589	0.377(244)	0.377(217)	0.0803	0.149
dan_daner_ANX_Gedi_GSMS.	0	1.00	0.28	0.391(208)	0.391(551)	−0.129	0.119
dan_daner_ANX_Gedi_VTSABD.	0	1.00	0.206	0.367(224)	0.367(406)	0.0363	0.0287
dan_daner_ANX_HUNT.	0	1.00	0.339	0.397(6587)	0.393(40740)	0.0188	0.0197
dan_daner_ANX_MGHI.	0	0.97	0.0489	0.375(1823)	0.358(5151)	0.0799	0.0406
dan_daner_ANX_MVP.	0	1.00	0.213	0.382(28525)	0.382(163731)	0.0126	0.0101
dan_daner_ANX_MoBa.	0	1.00	0.605	0.398(715)	0.391(22168)	0.0285	0.0551
dan_daner_ANX_PANIC_dca1.	0	0.99	0.0453	0.438(248)	0.389(970)	0.206	0.103
dan_daner_ANX_PANIC_gmdc.	0	0.99	0.789	0.338(247)	0.340(537)	0.0321	0.12
dan_daner_ANX_PANIC_gom3.	0	1.03	0.59	0.371(472)	0.380(1803)	−0.0412	0.0764
dan_daner_ANX_PANIC_gwc3.	0	0.99	0.0181	0.411(280)	0.353(855)	0.256	0.108
dan_daner_ANX_PANIC_swdf.	0	0.99	0.667	0.374(561)	0.372(2591)	0.0303	0.0705
dan_daner_ANX_PBK1.	0	1.00	0.49	0.369(1121)	0.362(11897)	0.0315	0.0459
dan_daner_ANX_QIMR_AGDS.	0	0.99	0.175	0.376(7312)	0.376(12813)	0.0401	0.0296
dan_daner_ANX_QIMR_Ad.	0	0.99	0.764	0.366(2449)	0.366(6343)	−0.0111	0.0369
dan_daner_ANX_QIMR_Nuup.	0	0.99	0.985	0.355(1131)	0.355(1233)	0.00123	0.0636
dan_daner_ANX_SWE_Catss.	0	1.00	0.306	0.394(540)	0.394(2333)	0.0739	0.0722
dan_daner_ANX_SWE_Salty.	0	1.00	0.406	0.372(687)	0.372(3445)	0.0515	0.0619
dan_daner_ANX_SWE_TwinGene.	0	1.00	0.135	0.387(1740)	0.387(6470)	0.0594	0.0398
dan_daner_ANX_UKBB.	0	0.99	0.163	0.376(25453)	0.376(58113)	0.016	0.0115
dan_daner_ANX_Utah.	0	1.00	0.475	0.374(258)	0.394(279)	−0.0902	0.126
dan_daner_ANX_alspac.	0	1.00	0.756	0.365(536)	0.369(3404)	−0.0211	0.068
dan_daner_ANX_bioVU.	0	1.00	0.0106	0.379(7586)	0.379(50545)	0.049	0.0192
dan_daner_ANX_iPSYCH.	0	1.01	0.00224	0.414(5288)	0.397(14422)	0.0713	0.0233
dan_daner_ANX_musp.	0	0.94	0.842	0.372(316)	0.366(715)	0.0203	0.102
**dan_**	**0**	**1.00**	**1.77e−08**	**0.376(122225)**	**0.370(729960)**	**0.0274**	**0.0049**

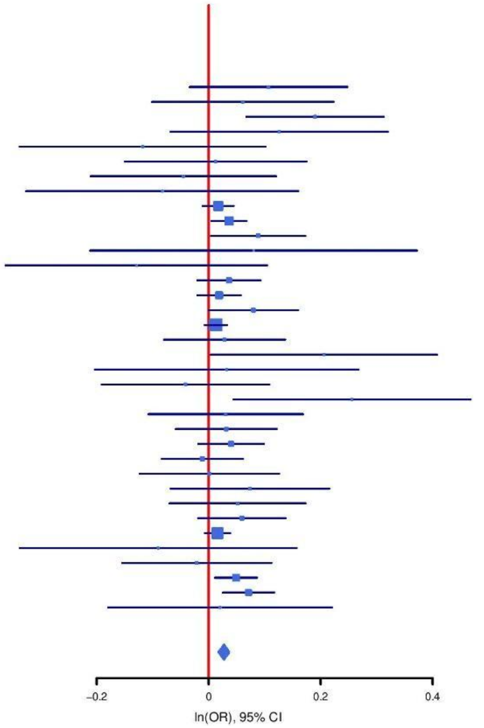

Supplementary Figure 46: Regional association plot (A) and forest plot (B) of SNP rs2371365. See full figure description on page 30.

A

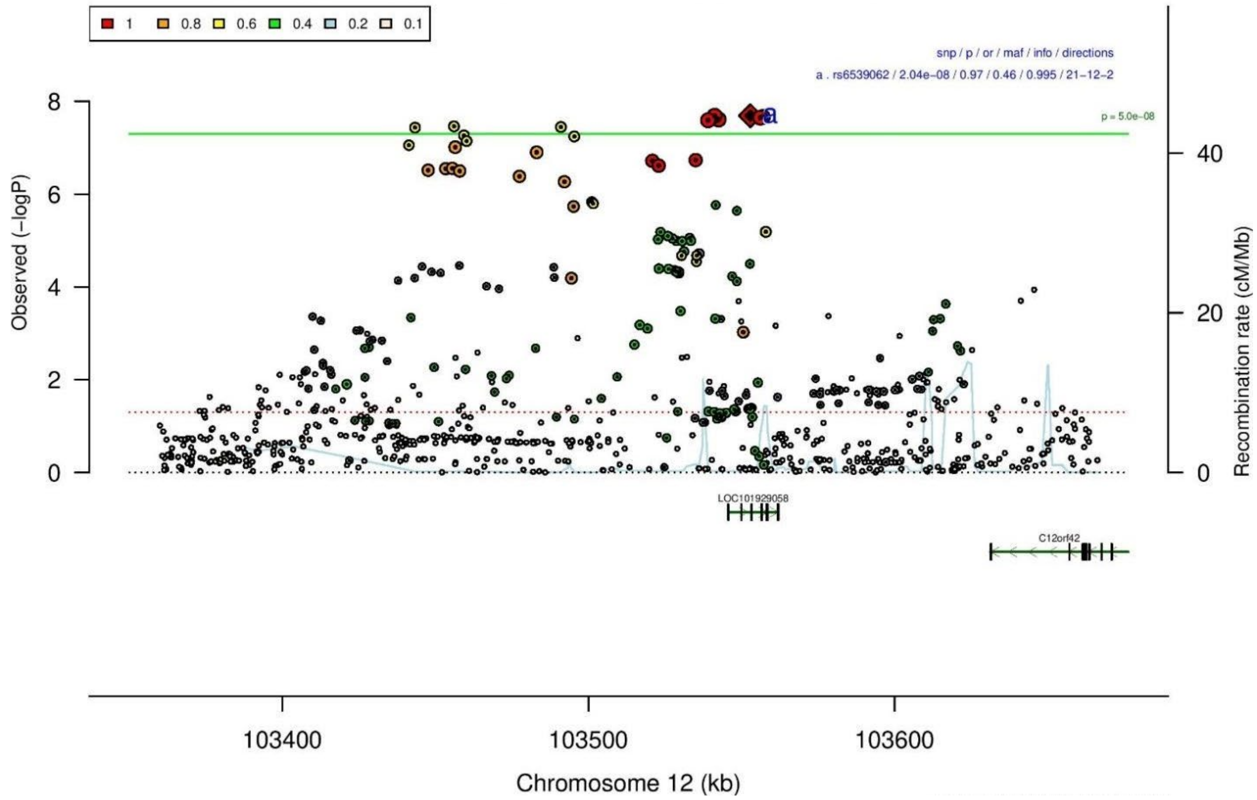

B

rs6539062		A/C		12:103552910			
+?−?−−++−+++−+++++−+−+−+0+++−++−+−+−				het_P:	0.519	het_I:	0.0
	**ngt**	**info**	**p_value**	**f_ca(n)**	**f_co(n)**	**ln(OR)**	**STDerr**
dan_daner_ANX_ANGST_mgs.	0	1.00	0.43	0.481(557)	0.481(779)	−0.0642	0.0814
dan_daner_ANX_ANGST_rs1.	0	0.99	0.563	0.482(331)	0.482(1629)	−0.057	0.098
dan_daner_ANX_ANGST_rs2.	0	0.99	0.754	0.475(209)	0.475(1023)	−0.034	0.108
dan_daner_ANX_ANGST_rs3.	0	0.99	0.148	0.481(370)	0.481(1817)	0.118	0.0814
dan_daner_ANX_ANGST_ship.	0	0.97	0.291	0.490(545)	0.490(834)	0.0865	0.0819
dan_daner_ANX_ANGST_trails.	0	1.00	0.903	0.492(278)	0.492(336)	−0.0143	0.117
dan_daner_ANX_EstBB.	0	0.99	0.892	0.545(12523)	0.545(134954)	0.00183	0.0135
dan_daner_ANX_FinnGen.	0	1.00	0.592	0.658(9182)	0.658(160108)	0.00869	0.0162
dan_daner_ANX_GSC.	0	0.99	0.204	0.484(1306)	0.473(12675)	0.0536	0.0422
dan_daner_ANX_Gedi_CHDS.	0	1.00	0.728	0.498(244)	0.498(217)	−0.0496	0.143
dan_daner_ANX_Gedi_GSMS.	0	1.00	0.785	0.477(208)	0.477(551)	0.0327	0.12
dan_daner_ANX_Gedi_VTSABD.	0	1.00	0.544	0.477(224)	0.477(406)	0.0166	0.0273
dan_daner_ANX_HUNT.	0	0.98	0.0383	0.548(6587)	0.535(40740)	0.0405	0.0196
dan_daner_ANX_MGHI.	0	0.96	0.412	0.465(1823)	0.474(5151)	−0.0325	0.0395
dan_daner_ANX_MVP.	0	1.00	0.00107	0.488(28525)	0.488(163731)	0.0318	0.0097
dan_daner_ANX_MoBa.	0	1.00	0.174	0.557(715)	0.537(22168)	0.0735	0.0541
dan_daner_ANX_PANIC_dca1.	0	1.00	0.353	0.474(248)	0.497(970)	−0.0944	0.102
dan_daner_ANX_PANIC_gmdc.	0	0.99	0.235	0.495(247)	0.465(537)	0.134	0.113
dan_daner_ANX_PANIC_gom3.	0	0.98	1	0.491(472)	0.486(1803)	0	0.0752
dan_daner_ANX_PANIC_gwc3.	0	0.96	0.0938	0.513(280)	0.466(855)	0.18	0.107
dan_daner_ANX_PANIC_swdf.	0	0.99	0.114	0.585(561)	0.553(2591)	0.109	0.069
dan_daner_ANX_PBK1.	0	1.00	0.372	0.474(1121)	0.466(11897)	0.0392	0.0438
dan_daner_ANX_QIMR_AGDS.	0	0.97	0.345	0.478(7312)	0.478(12813)	0.0274	0.029
dan_daner_ANX_QIMR_Ad.	0	0.97	0.46	0.462(2449)	0.462(6343)	−0.0267	0.0362
dan_daner_ANX_QIMR_Nuup.	0	0.97	0.299	0.481(1131)	0.481(1233)	0.0662	0.0637
dan_daner_ANX_SWE_Catss.	0	1.00	0.421	0.458(540)	0.458(2333)	−0.0584	0.0726
dan_daner_ANX_SWE_Salty.	0	1.00	0.526	0.457(687)	0.457(3445)	0.0388	0.0611
dan_daner_ANX_SWE_TwinGene.	0	1.00	0.653	0.438(1740)	0.438(6470)	−0.0176	0.0393
dan_daner_ANX_UKBB.	0	0.99	8.62e−06	0.474(25453)	0.474(58113)	0.0498	0.0112
dan_daner_ANX_Utah.	0	1.04	0.37	0.469(258)	0.496(279)	−0.108	0.12
dan_daner_ANX_alspac.	0	1.00	0.37	0.494(536)	0.480(3404)	0.0599	0.0668
dan_daner_ANX_bioVU.	0	1.00	0.0598	0.486(7586)	0.486(50545)	0.0351	0.0186
dan_daner_ANX_iPSYCH.	0	1.00	0.27	0.509(5288)	0.502(14422)	0.0254	0.023
dan_daner_ANX_musp.	0	0.93	0.375	0.484(316)	0.504(715)	−0.0885	0.0998
**dan_**	**0**	**0.99**	**2.04e−08**	**0.506(122225)**	**0.538(729960)**	**0.0268**	**0.0048**

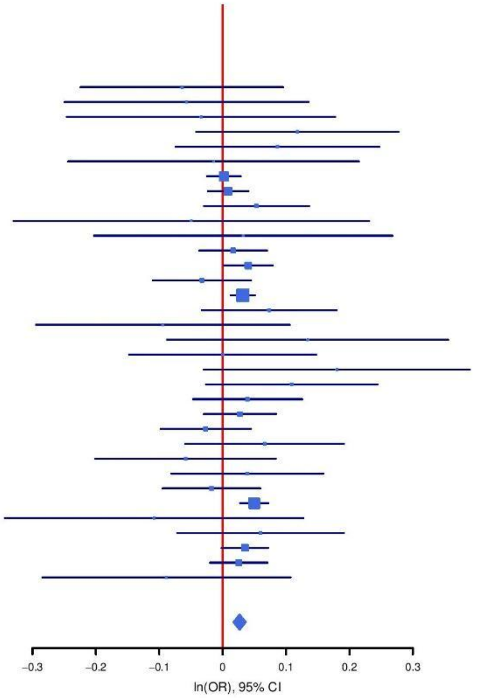

Supplementary Figure 47: Regional association plot (A) and forest plot (B) of SNP rs6539062. See full figure description on page 30.

A

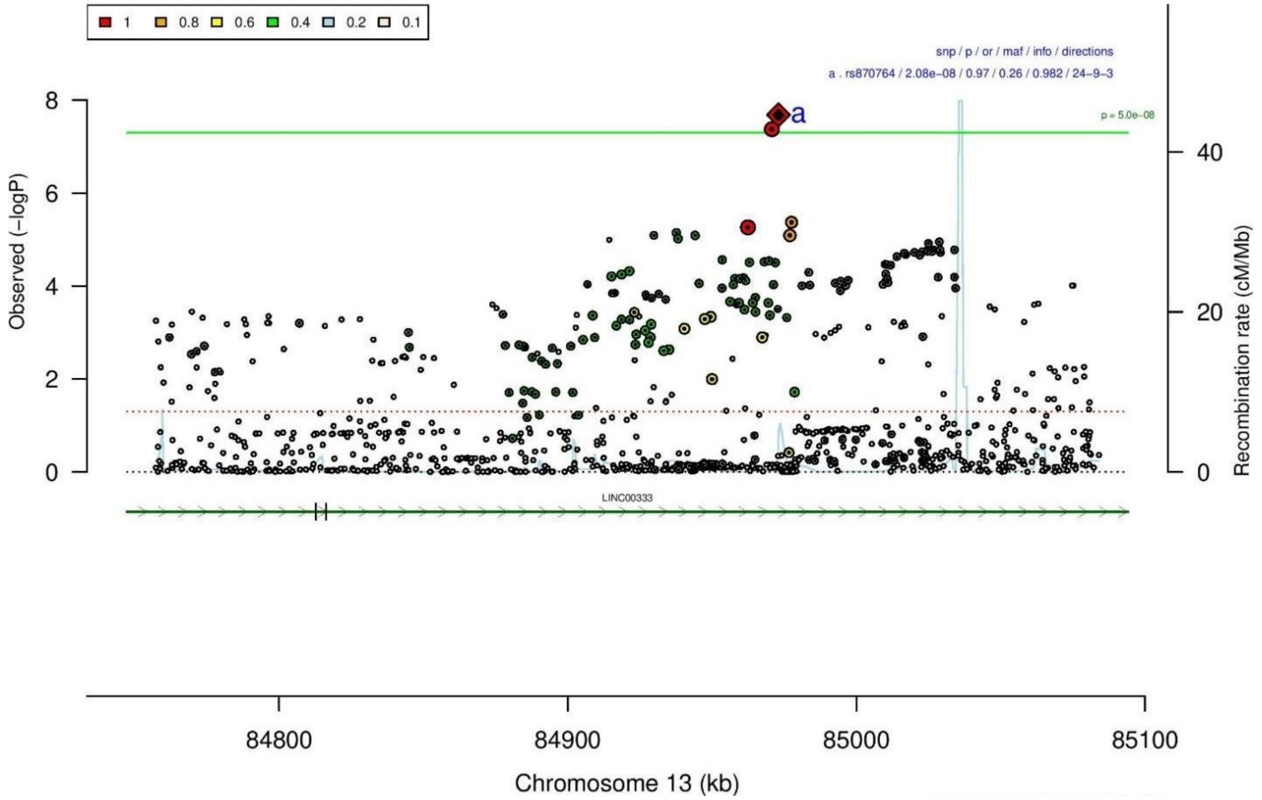

B

rs870764		A/G		13:84973006			
+?+?+--+++++-++-+-++-++-+++?+++-+++-				het_P:	0.366	het_I:	6.2
	ngt	info	p_value	f_ca(n)	f_co(n)	ln(OR)	STDerr
dan_daner_ANX_ANGST_mgs.	0	1.00	0.976	0.727(557)	0.727(779)	0.00274	0.0896
dan_daner_ANX_ANGST_rs1.	0	0.97	0.0213	0.707(331)	0.707(1629)	0.266	0.116
dan_daner_ANX_ANGST_rs2.	0	0.97	0.246	0.725(209)	0.725(1023)	-0.146	0.126
dan_daner_ANX_ANGST_rs3.	0	0.97	0.996	0.714(370)	0.714(1817)	-0.000409	0.0917
dan_daner_ANX_ANGST_ship.	0	0.98	0.747	0.740(545)	0.740(834)	0.0306	0.095
dan_daner_ANX_ANGST_trails.	0	1.00	0.387	0.713(278)	0.713(336)	0.119	0.138
dan_daner_ANX_EstBB.	0	0.96	0.0755	0.796(12523)	0.796(134954)	0.0302	0.017
dan_daner_ANX_FinnGen.	0	0.99	0.204	0.723(9182)	0.723(160108)	0.0219	0.0172
dan_daner_ANX_GSC.	0	0.96	0.0681	0.728(1306)	0.745(12675)	-0.0891	0.0488
dan_daner_ANX_Gedi_CHDS.	0	1.00	0.0432	0.759(244)	0.759(217)	-0.347	0.171
dan_daner_ANX_Gedi_GSMS.	0	1.00	0.533	0.747(208)	0.747(551)	0.0878	0.141
dan_daner_ANX_Gedi_VTSABD.	0	1.00	0.553	0.717(224)	0.717(406)	0.0185	0.0311
dan_daner_ANX_HUNT.	0	0.99	0.133	0.757(6587)	0.748(40740)	0.0337	0.0224
dan_daner_ANX_MGHI.	0	0.95	0.255	0.742(1823)	0.732(5151)	0.0514	0.0451
dan_daner_ANX_MVP.	0	1.00	0.0142	0.734(28525)	0.734(163731)	0.0272	0.0111
dan_daner_ANX_MoBa.	0	1.00	0.0432	0.782(715)	0.759(22168)	0.127	0.0628
dan_daner_ANX_PANIC_dca1.	1	1.02	0.21	0.764(248)	0.739(970)	0.148	0.118
dan_daner_ANX_PANIC_gmdc.	0	0.99	0.357	0.713(247)	0.735(537)	-0.115	0.125
dan_daner_ANX_PANIC_gom3.	1	0.98	0.214	0.743(472)	0.726(1803)	0.108	0.0867
dan_daner_ANX_PANIC_gwc3.	0	0.99	0.85	0.739(280)	0.728(855)	0.0223	0.118
dan_daner_ANX_PANIC_swdf.	0	0.99	0.298	0.743(561)	0.735(2591)	0.0811	0.078
dan_daner_ANX_QIMR_AGDS.	0	0.90	0.0701	0.722(7312)	0.722(12813)	0.0614	0.0339
dan_daner_ANX_QIMR_Ad.	0	0.90	0.0302	0.730(2449)	0.730(6343)	0.0887	0.0409
dan_daner_ANX_QIMR_Nuup.	0	0.90	0.454	0.732(1131)	0.732(1233)	0.0534	0.0713
dan_daner_ANX_SWE_Catss.	0	1.00	0.278	0.268(540)	0.268(2333)	-0.0832	0.0766
dan_daner_ANX_SWE_Salty.	0	1.00	0.964	0.273(687)	0.273(3445)	0.00298	0.0667
dan_daner_ANX_SWE_TwinGene.	0	1.00	0.0573	0.271(1740)	0.271(6470)	0.0835	0.0439
dan_daner_ANX_UKBB.	0	0.97	0.0019	0.737(25453)	0.737(58113)	0.0399	0.0128
dan_daner_ANX_Utah.	0	0.96	0.92	0.752(258)	0.759(279)	-0.0148	0.148
dan_daner_ANX_alspac.	0	0.98	0.507	0.741(536)	0.734(3404)	0.0497	0.0751
dan_daner_ANX_bioVU.	0	1.00	0.0882	0.733(7586)	0.733(50545)	0.036	0.0211
dan_daner_ANX_iPSYCH.	18	0.99	0.767	0.739(5288)	0.740(14422)	-0.0078	0.0264
dan_daner_ANX_musp.	1	1.02	0.954	0.715(316)	0.718(715)	-0.00608	0.106
**dan_**	**21**	**0.98**	**2.08e-08**	**0.730(122225)**	**0.737(729960)**	**0.0309**	**0.0055**

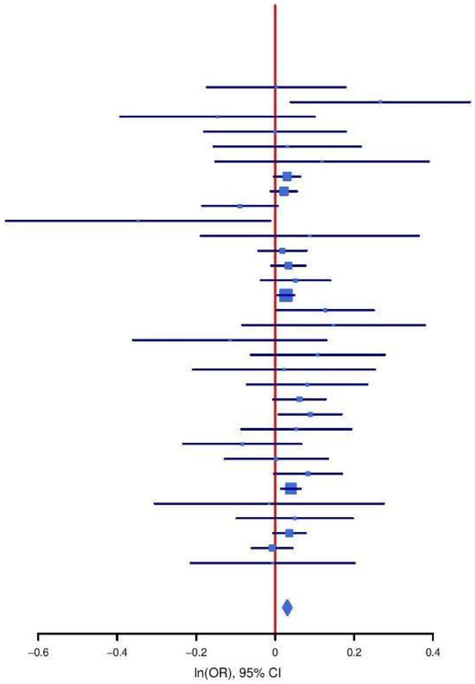

Supplementary Figure 48: Regional association plot (A) and forest plot (B) of SNP rs870764. See full figure description on page 30.

A

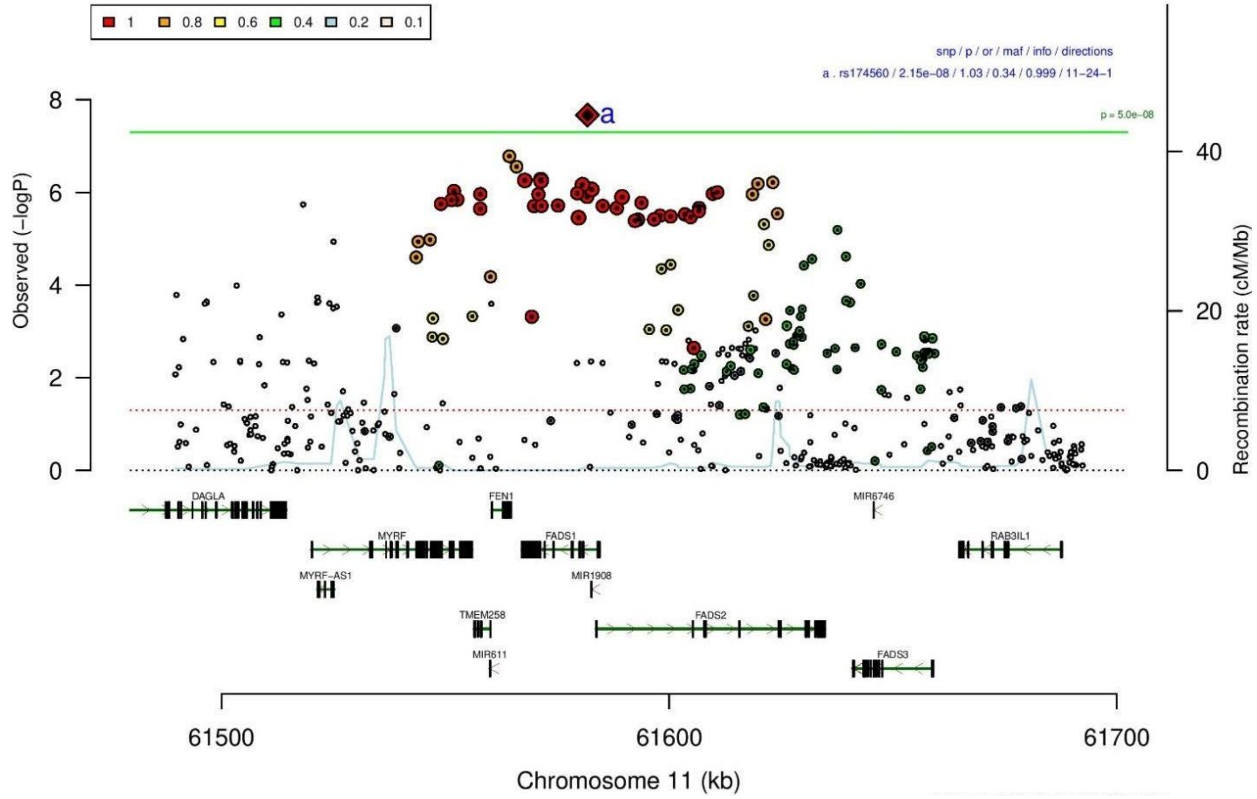

B

rs174560 **C/T** **11:61581764**

−+++−++−−−−−−−−−−−−+−+?−+−+−−+−−+−−−− het_P: 0.268 het_I: 12.0

	ngt	info	p_value	f_ca(n)	f_co(n)	ln(OR)	STDerr
dan_daner_ANX_ANGST_gsk.	0	0.97	0.311	0.267(852)	0.267(1103)	−0.0761	0.0751
dan_daner_ANX_ANGST_mgs.	0	1.00	0.694	0.297(557)	0.297(779)	−0.0354	0.09
dan_daner_ANX_ANGST_ntr.	0	1.00	0.695	0.299(1521)	0.299(2970)	−0.0264	0.0674
dan_daner_ANX_ANGST_rs1.	0	1.00	0.336	0.302(331)	0.302(1629)	0.101	0.105
dan_daner_ANX_ANGST_rs2.	0	1.00	0.15	0.287(209)	0.287(1023)	−0.179	0.124
dan_daner_ANX_ANGST_rs3.	0	1.00	0.507	0.302(370)	0.302(1817)	−0.0573	0.0864
dan_daner_ANX_ANGST_ship.	0	1.00	0.864	0.314(545)	0.314(834)	0.0152	0.0888
dan_daner_ANX_ANGST_trails.	0	0.90	0.154	0.299(278)	0.299(336)	0.196	0.137
dan_daner_ANX_EstBB.	0	1.00	0.112	0.329(12523)	0.329(134954)	0.0227	0.0143
dan_daner_ANX_FinnGen.	0	1.00	0.0166	0.388(9182)	0.388(160108)	0.0377	0.0157
dan_daner_ANX_GSC.	0	1.00	0.967	0.318(1306)	0.320(12675)	0.00187	0.045
dan_daner_ANX_Gedi_CHDS.	0	1.00	0.26	0.309(244)	0.309(217)	0.178	0.158
dan_daner_ANX_Gedi_GSMS.	0	1.00	0.701	0.296(208)	0.296(551)	0.0492	0.128
dan_daner_ANX_Gedi_VTSABD.	0	1.00	0.648	0.275(224)	0.275(406)	0.0144	0.0315
dan_daner_ANX_HUNT.	0	1.00	0.0425	0.337(6587)	0.329(40740)	0.0414	0.0204
dan_daner_ANX_MGHI.	0	1.01	0.435	0.294(1823)	0.299(5151)	−0.033	0.0424
dan_daner_ANX_MoBa.	0	1.00	0.0493	0.358(715)	0.333(22168)	0.113	0.0575
dan_daner_ANX_PANIC_dca1.	0	0.99	0.268	0.329(248)	0.300(970)	0.121	0.109
dan_daner_ANX_PANIC_gmdc.	0	1.02	0.721	0.280(247)	0.291(537)	−0.0438	0.123
dan_daner_ANX_PANIC_gom3.	0	0.96	0.723	0.294(472)	0.288(1803)	0.0294	0.0832
dan_daner_ANX_PANIC_gwc3.	0	1.02	0.864	0.292(280)	0.296(855)	−0.0193	0.113
dan_daner_ANX_PANIC_swdf.	0	0.98	0.0643	0.359(561)	0.325(2591)	0.133	0.0718
dan_daner_ANX_PBK1.	0	1.00	0.171	0.312(1121)	0.300(11897)	0.0658	0.0482
dan_daner_ANX_QIMR_AGDS.	0	1.00	0.0151	0.315(7312)	0.315(12813)	0.0753	0.031
dan_daner_ANX_QIMR_Ad.	0	1.00	0.452	0.312(2449)	0.312(6343)	−0.029	0.0385
dan_daner_ANX_QIMR_Nuup.	0	1.00	0.928	0.296(1131)	0.296(1233)	0.0063	0.0695
dan_daner_ANX_SWE_Catss.	0	1.00	0.654	0.343(540)	0.343(2333)	−0.0333	0.0743
dan_daner_ANX_SWE_Salty.	0	1.00	0.495	0.327(687)	0.327(3445)	0.0437	0.0641
dan_daner_ANX_SWE_TwinGene.	0	1.00	0.00197	0.320(1740)	0.320(6470)	0.128	0.0413
dan_daner_ANX_UKBB.	0	1.00	0.00557	0.313(25453)	0.313(58113)	0.0333	0.012
dan_daner_ANX_Utah.	0	1.02	0.0152	0.363(258)	0.291(279)	0.318	0.131
dan_daner_ANX_alspac.	0	1.00	0.779	0.308(536)	0.304(3404)	0.0204	0.0724
dan_daner_ANX_bioVU.	0	1.00	0.0225	0.303(7586)	0.303(50545)	0.0462	0.0202
dan_daner_ANX_iPSYCH.	0	1.00	0.459	0.304(5288)	0.301(14422)	0.0185	0.025
dan_daner_ANX_musp.	0	0.96	0.191	0.261(316)	0.287(715)	−0.144	0.11
**dan_**	**0**	**1.00**	**2.15e−08**	**0.322(122225)**	**0.338(729960)**	**0.0323**	**0.0058**

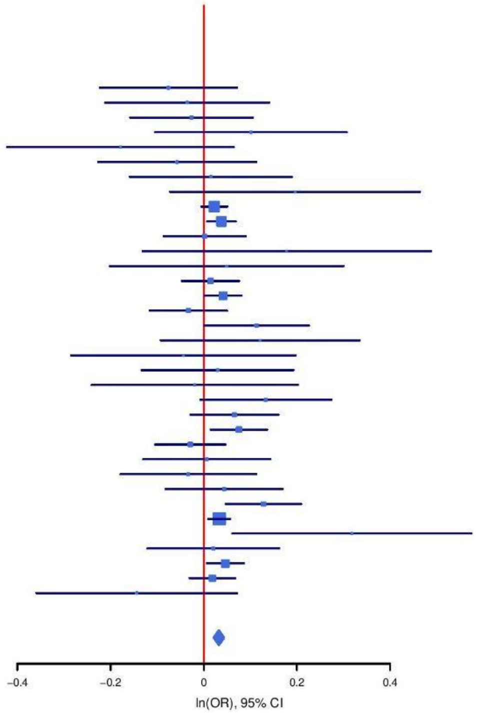

Supplementary Figure 49: Regional association plot (A) and forest plot (B) of SNP rs174560. See full figure description on page 30.

A

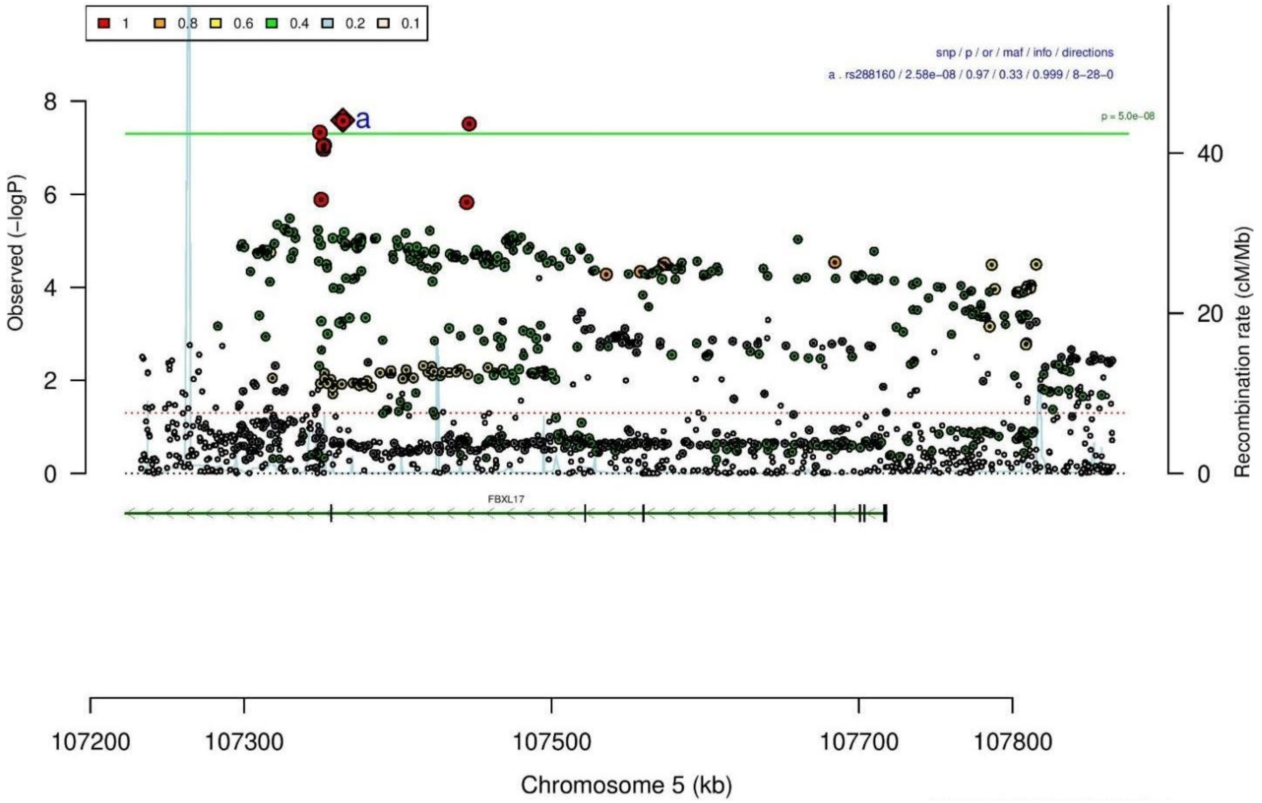

B

rs288160		T/C		5:107364269			
				het_P:	0.327	het_I:	8.4
	**ngt**	**info**	**p_value**	**f_ca(n)**	**f_co(n)**	**ln(OR)**	**STDerr**
dan_daner_ANX_ANGST_gsk.	0	0.99	0.303	0.322(852)	0.322(1103)	0.0742	0.072
dan_daner_ANX_ANGST_mgs.	0	1.00	0.839	0.329(557)	0.329(779)	−0.0174	0.0854
dan_daner_ANX_ANGST_ntr.	0	1.00	0.144	0.344(1521)	0.344(2970)	−0.0955	0.0654
dan_daner_ANX_ANGST_rs1.	0	0.99	0.247	0.332(331)	0.332(1629)	−0.122	0.106
dan_daner_ANX_ANGST_rs2.	0	1.00	0.379	0.339(209)	0.339(1023)	−0.102	0.116
dan_daner_ANX_ANGST_rs3.	0	0.99	0.392	0.343(370)	0.343(1817)	0.074	0.0865
dan_daner_ANX_ANGST_ship.	0	1.00	0.0607	0.310(545)	0.310(834)	0.163	0.0868
dan_daner_ANX_ANGST_trails.	0	1.00	0.154	0.325(278)	0.325(336)	−0.185	0.13
dan_daner_ANX_EstBB.	0	1.00	0.31	0.301(12523)	0.301(134954)	−0.0148	0.0146
dan_daner_ANX_FinnGen.	0	1.00	0.305	0.374(9182)	0.374(160108)	−0.0164	0.0159
dan_daner_ANX_GSC.	0	1.02	0.49	0.317(1306)	0.323(12675)	−0.0308	0.0446
dan_daner_ANX_Gedi_CHDS.	0	1.00	0.442	0.345(244)	0.345(217)	−0.113	0.147
dan_daner_ANX_Gedi_GSMS.	0	1.00	0.747	0.347(208)	0.347(551)	−0.0399	0.124
dan_daner_ANX_Gedi_VTSABD.	0	1.00	0.698	0.366(224)	0.366(406)	0.0116	0.0298
dan_daner_ANX_HUNT.	0	1.00	0.0129	0.314(6587)	0.323(40740)	−0.0514	0.0207
dan_daner_ANX_MGHI.	0	1.01	0.00378	0.277(1823)	0.302(5151)	−0.124	0.0428
dan_daner_ANX_MVP.	0	1.00	0.0305	0.318(28525)	0.318(163731)	−0.0219	0.0101
dan_daner_ANX_MoBa.	0	1.00	0.577	0.311(715)	0.318(22168)	−0.0323	0.0579
dan_daner_ANX_PANIC_dca1.	0	1.00	0.134	0.290(248)	0.327(970)	−0.167	0.111
dan_daner_ANX_PANIC_gmdc.	0	1.05	0.154	0.271(247)	0.306(537)	−0.174	0.122
dan_daner_ANX_PANIC_gom3.	0	0.96	0.898	0.306(472)	0.311(1803)	−0.0106	0.0823
dan_daner_ANX_PANIC_gwc3.	0	0.99	0.0723	0.336(280)	0.308(855)	0.2	0.112
dan_daner_ANX_PANIC_swdf.	0	0.99	0.441	0.315(561)	0.329(2591)	−0.0567	0.0735
dan_daner_ANX_PBK1.	0	1.00	0.369	0.301(1121)	0.311(11897)	−0.0434	0.0484
dan_daner_ANX_QIMR_AGDS.	0	0.99	0.0377	0.326(7312)	0.326(12813)	−0.0637	0.0306
dan_daner_ANX_QIMR_Ad.	0	0.99	0.288	0.314(2449)	0.314(6343)	−0.0408	0.0383
dan_daner_ANX_QIMR_Nuup.	0	0.99	0.88	0.310(1131)	0.310(1233)	0.0103	0.0678
dan_daner_ANX_SWE_Catss.	0	1.00	0.135	0.344(540)	0.344(2333)	−0.115	0.077
dan_daner_ANX_SWE_Salty.	0	1.00	0.617	0.340(687)	0.340(3445)	−0.032	0.064
dan_daner_ANX_SWE_TwinGene.	0	1.00	0.821	0.331(1740)	0.331(6470)	0.00926	0.0409
dan_daner_ANX_UKBB.	0	1.00	0.00465	0.334(25453)	0.334(58113)	−0.0335	0.0119
dan_daner_ANX_Utah.	0	1.07	0.92	0.310(258)	0.315(279)	−0.0129	0.128
dan_daner_ANX_alspac.	0	1.00	0.0791	0.304(536)	0.330(3404)	−0.127	0.0728
dan_daner_ANX_bioVU.	0	1.00	0.102	0.326(7586)	0.326(50545)	−0.0326	0.0199
dan_daner_ANX_iPSYCH.	0	1.00	0.13	0.313(5288)	0.322(14422)	−0.0374	0.0247
dan_daner_ANX_musp.	0	1.03	0.204	0.345(316)	0.316(715)	0.127	0.1
**dan_**	**0**	**1.00**	**2.58e−08**	**0.324(122225)**	**0.330(729960)**	**−0.0278**	**0.005**

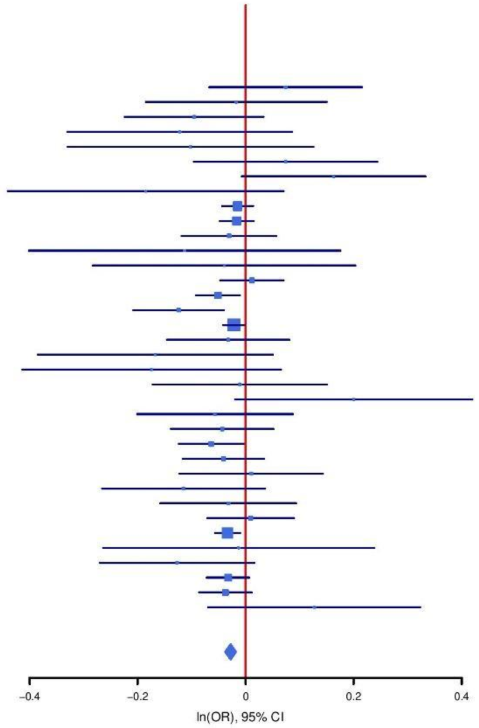

Supplementary Figure 50: Regional association plot (A) and forest plot (B) of SNP rs288160. See full figure description on page 30.

A

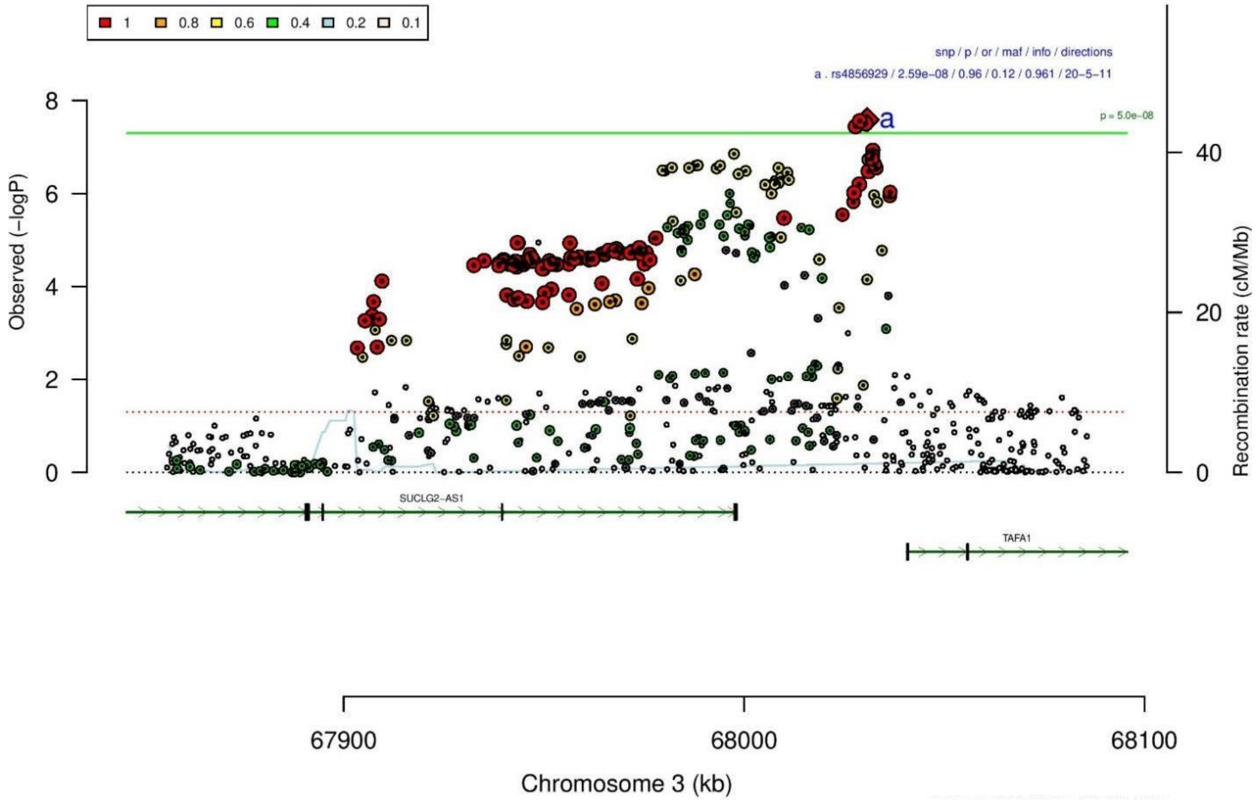

B

rs4856929		T/C		3:68030736			
−+++???−??++−++++++−++??????+++++−++				het_P:	0.0497	het_I:	34.1
	**ngt**	**info**	**p_value**	**f_ca(n)**	**f_co(n)**	**ln(OR)**	**STDerr**
dan_daner_ANX_ANGST_gsk.	0	0.99	0.947	0.893(852)	0.893(1103)	0.00717	0.107
dan_daner_ANX_ANGST_mgs.	0	1.00	0.32	0.887(557)	0.887(779)	0.131	0.132
dan_daner_ANX_ANGST_ntr.	0	0.97	0.115	0.893(1521)	0.893(2970)	0.154	0.0978
dan_daner_ANX_ANGST_ship.	0	0.99	0.22	0.892(545)	0.892(834)	−0.161	0.131
dan_daner_ANX_EstBB.	0	0.90	0.866	0.902(12523)	0.902(134954)	0.00399	0.0236
dan_daner_ANX_FinnGen.	0	0.98	0.00908	0.896(9182)	0.896(160108)	0.067	0.0257
dan_daner_ANX_GSC.	0	0.96	0.521	0.908(1306)	0.905(12675)	0.0465	0.0725
dan_daner_ANX_Gedi_CHDS.	0	1.00	0.00751	0.909(244)	0.909(217)	−0.68	0.254
dan_daner_ANX_Gedi_GSMS.	0	1.00	0.426	0.900(208)	0.900(551)	0.163	0.205
dan_daner_ANX_Gedi_VTSABD.	0	1.00	0.99	0.901(224)	0.901(406)	0.000548	0.0437
dan_daner_ANX_HUNT.	0	0.94	0.00492	0.902(6587)	0.894(40740)	0.0928	0.0333
dan_daner_ANX_MGHI.	0	0.88	0.0869	0.902(1823)	0.892(5151)	0.118	0.0689
dan_daner_ANX_MVP.	0	1.00	0.000471	0.893(28525)	0.893(163731)	0.0535	0.0153
dan_daner_ANX_MoBa.	0	1.00	0.0313	0.877(715)	0.895(22168)	−0.187	0.087
dan_daner_ANX_QIMR_AGDS.	0	0.87	0.0643	0.887(7312)	0.887(12813)	0.089	0.0481
dan_daner_ANX_QIMR_Ad.	0	0.87	0.044	0.877(2449)	0.877(6343)	0.13	0.0647
dan_daner_ANX_QIMR_Nuup.	0	0.87	0.221	0.871(1131)	0.871(1233)	0.133	0.109
dan_daner_ANX_SWE_Catss.	0	1.00	0.353	0.117(540)	0.117(2333)	0.104	0.112
dan_daner_ANX_SWE_Salty.	0	1.00	0.574	0.107(687)	0.107(3445)	0.0567	0.101
dan_daner_ANX_SWE_TwinGene.	0	1.00	0.61	0.113(1740)	0.113(6470)	−0.0315	0.0617
dan_daner_ANX_UKBB.	0	1.00	0.0233	0.893(25453)	0.893(58113)	0.041	0.0181
dan_daner_ANX_Utah.	0	0.86	0.457	0.882(258)	0.868(279)	0.149	0.2
dan_daner_ANX_alspac.	0	0.81	0.925	0.893(536)	0.894(3404)	−0.0113	0.12
dan_daner_ANX_iPSYCH.	0	0.92	0.534	0.891(5288)	0.889(14422)	0.024	0.0385
dan_daner_ANX_musp.	0	0.95	0.0681	0.909(316)	0.883(715)	0.305	0.167
**dan_**	**0**	**0.96**	**2.59e−08**	**0.873(122225)**	**0.881(729960)**	**0.0455**	**0.0082**

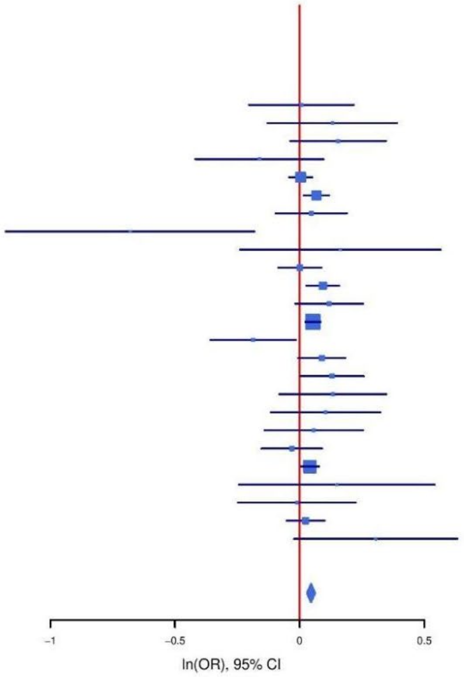

Supplementary Figure 51: Regional association plot (A) and forest plot (B) of SNP rs4856929. See full figure description on page 30.

A

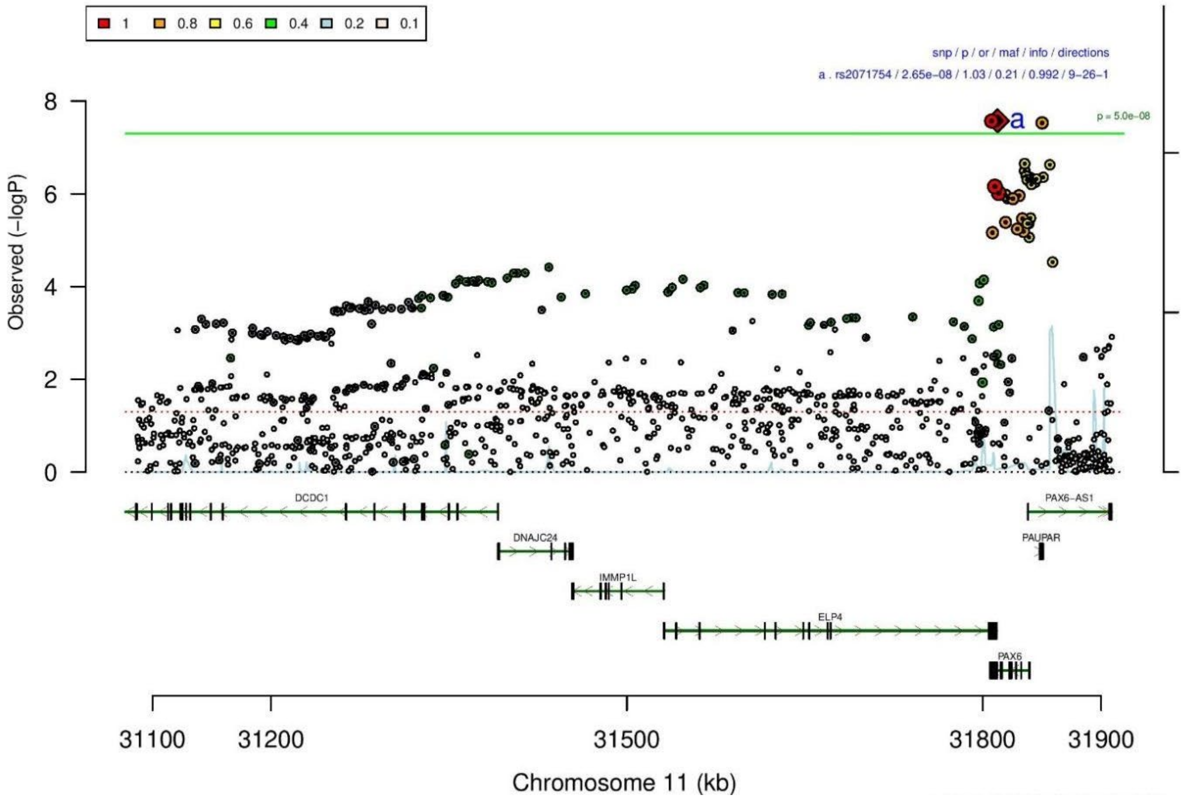

B

rs2071754		T/C		11:31812582			
−+−?+−−−−−−−−−−++−−−−−−−−++−−+−−−−+−+−−				het_P:	0.0181	het_I:	36.4
	**ngt**	**info**	**p_value**	**f_ca(n)**	**f_co(n)**	**ln(OR)**	**STDerr**
dan_daner_ANX_ANGST_gsk.	0	0.93	0.644	0.815(852)	0.815(1103)	0.041	0.0886
dan_daner_ANX_ANGST_mgs.	0	1.00	0.228	0.807(557)	0.807(779)	−0.123	0.102
dan_daner_ANX_ANGST_rs1.	0	1.00	0.813	0.817(331)	0.817(1629)	0.03	0.126
dan_daner_ANX_ANGST_rs2.	0	1.00	0.0042	0.815(209)	0.815(1023)	−0.374	0.131
dan_daner_ANX_ANGST_rs3.	0	1.00	0.566	0.814(370)	0.814(1817)	−0.0596	0.104
dan_daner_ANX_ANGST_ship.	0	1.00	0.0575	0.821(545)	0.821(834)	−0.203	0.107
dan_daner_ANX_ANGST_trails.	0	0.99	0.185	0.802(278)	0.802(336)	−0.202	0.152
dan_daner_ANX_EstBB.	0	0.98	0.118	0.823(12523)	0.823(134954)	−0.0277	0.0177
dan_daner_ANX_FinnGen.	0	1.00	0.0177	0.775(9182)	0.775(160108)	−0.0432	0.0182
dan_daner_ANX_GSC.	1	1.00	0.275	0.805(1306)	0.797(12675)	0.0571	0.0523
dan_daner_ANX_Gedi_CHDS.	0	1.00	0.841	0.811(244)	0.811(217)	−0.0355	0.177
dan_daner_ANX_Gedi_GSMS.	0	1.00	0.294	0.819(208)	0.819(551)	−0.161	0.153
dan_daner_ANX_Gedi_VTSABD.	0	1.00	0.883	0.806(224)	0.806(406)	0.00516	0.035
dan_daner_ANX_HUNT.	0	0.98	0.347	0.791(6587)	0.797(40740)	−0.0225	0.0239
dan_daner_ANX_MGHI.	0	0.98	0.0886	0.782(1823)	0.796(5151)	−0.081	0.0476
dan_daner_ANX_MVP.	0	1.00	0.172	0.796(28525)	0.796(163731)	−0.0158	0.0116
dan_daner_ANX_MoBa.	0	1.00	0.0125	0.764(715)	0.794(22168)	−0.165	0.0661
dan_daner_ANX_PANIC_dca1.	0	1.00	0.391	0.798(248)	0.818(970)	−0.11	0.128
dan_daner_ANX_PANIC_gmdc.	1	0.98	0.123	0.824(247)	0.791(537)	0.227	0.147
dan_daner_ANX_PANIC_gom3.	0	1.01	0.776	0.821(472)	0.818(1803)	0.0275	0.0966
dan_daner_ANX_PANIC_gwc3.	1	0.98	0.364	0.787(280)	0.815(855)	−0.118	0.13
dan_daner_ANX_PANIC_swdf.	0	0.97	0.0986	0.794(561)	0.818(2591)	−0.142	0.086
dan_daner_ANX_PBK1.	0	1.00	0.161	0.810(1121)	0.799(11897)	0.0796	0.0568
dan_daner_ANX_QIMR_AGDS.	0	0.91	0.423	0.781(7312)	0.781(12813)	−0.029	0.0361
dan_daner_ANX_QIMR_Ad.	0	0.91	0.0695	0.792(2449)	0.792(6343)	−0.0806	0.0444
dan_daner_ANX_QIMR_Nuup.	0	0.91	0.362	0.792(1131)	0.792(1233)	−0.0707	0.0775
dan_daner_ANX_SWE_Catss.	0	1.00	0.378	0.194(540)	0.194(2333)	0.0804	0.0912
dan_daner_ANX_SWE_Salty.	0	1.00	0.0863	0.190(687)	0.190(3445)	−0.131	0.0761
dan_daner_ANX_SWE_TwinGene.	0	1.00	0.303	0.183(1740)	0.183(6470)	0.0523	0.0507
dan_daner_ANX_UKBB.	0	1.00	0.0032	0.798(25453)	0.798(58113)	−0.0407	0.0138
dan_daner_ANX_Utah.	0	0.97	0.983	0.806(258)	0.807(279)	−0.00339	0.158
dan_daner_ANX_alspac.	0	1.00	0.00807	0.777(536)	0.814(3404)	−0.217	0.0807
dan_daner_ANX_bioVU.	0	1.00	0.272	0.797(7586)	0.797(50545)	−0.0256	0.0233
dan_daner_ANX_iPSYCH.	0	0.99	0.0107	0.809(5288)	0.820(14422)	−0.0757	0.0297
dan_daner_ANX_musp.	0	0.99	0.0307	0.768(316)	0.814(715)	−0.255	0.118
**dan_**	**3**	**0.99**	**2.65e−08**	**0.782(122225)**	**0.787(729960)**	**−0.0326**	**0.0059**

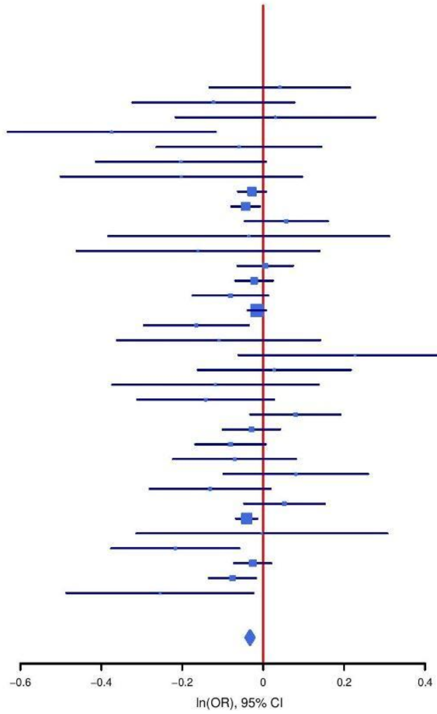

Supplementary Figure 52: Regional association plot (A) and forest plot (B) of SNP rs2071754. See full figure description on page 30.

A

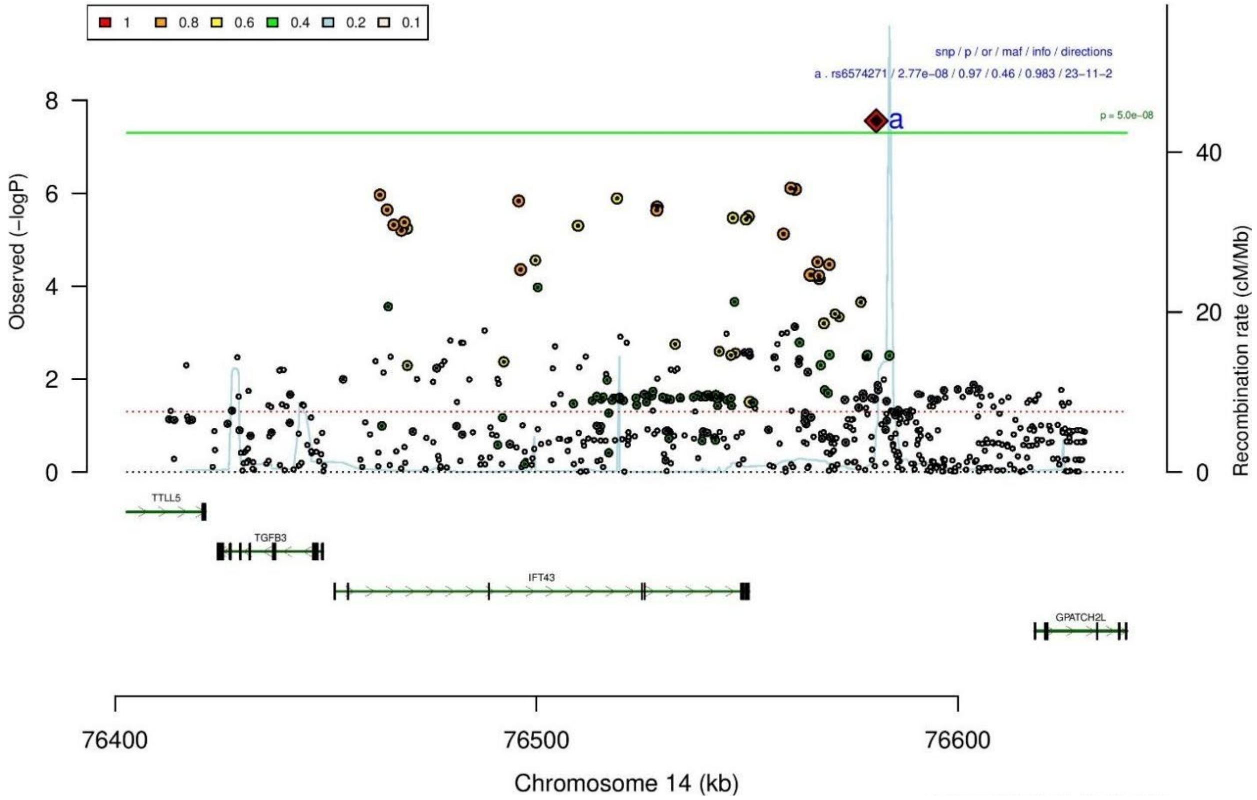

B

rs6574271 **C/T** **14:76580655**

++−+++++−?+++−−+++−+−+−−+−+?+++−+++−

het_P: 0.0814 het_I: 26.5

	ngt	info	p_value	f_ca(n)	f_co(n)	ln(OR)	STDerr
dan_daner_ANX_ANGST_gsk.	0	0.84	0.394	0.448(852)	0.448(1103)	−0.0631	0.0741
dan_daner_ANX_ANGST_mgs.	0	1.00	0.822	0.457(557)	0.457(779)	0.019	0.0843
dan_daner_ANX_ANGST_ntr.	0	0.99	0.125	0.448(1521)	0.448(2970)	−0.0946	0.0616
dan_daner_ANX_ANGST_rs1.	0	0.94	0.65	0.440(331)	0.440(1629)	−0.046	0.102
dan_daner_ANX_ANGST_rs2.	0	1.00	0.478	0.425(209)	0.425(1023)	−0.08	0.112
dan_daner_ANX_ANGST_rs3.	0	0.99	0.34	0.438(370)	0.438(1817)	−0.0789	0.0828
dan_daner_ANX_ANGST_ship.	0	0.94	0.0741	0.458(545)	0.458(834)	−0.153	0.0858
dan_daner_ANX_ANGST_trails.	0	0.92	0.787	0.446(278)	0.446(336)	0.0348	0.129
dan_daner_ANX_EstBB.	0	0.96	0.00645	0.459(12523)	0.459(134954)	−0.0375	0.0138
dan_daner_ANX_FinnGen.	0	0.99	0.0267	0.493(9182)	0.493(160108)	−0.0341	0.0154
dan_daner_ANX_GSC.	1	1.01	0.00483	0.416(1306)	0.442(12675)	−0.118	0.0421
dan_daner_ANX_Gedi_CHDS.	0	1.00	0.817	0.440(244)	0.440(217)	−0.0325	0.14
dan_daner_ANX_Gedi_GSMS.	0	1.00	0.339	0.416(208)	0.416(551)	0.117	0.122
dan_daner_ANX_Gedi_VTSABD.	0	1.00	0.248	0.451(224)	0.451(406)	0.0321	0.0277
dan_daner_ANX_HUNT.	0	0.98	0.00529	0.473(6587)	0.488(40740)	−0.0546	0.0196
dan_daner_ANX_MGHI.	0	0.97	0.935	0.439(1823)	0.440(5151)	0.00321	0.0395
dan_daner_ANX_MVP.	0	1.00	0.013	0.443(28525)	0.443(163731)	−0.0246	0.0099
dan_daner_ANX_MoBa.	0	1.00	0.0546	0.444(715)	0.467(22168)	−0.104	0.0541
dan_daner_ANX_PANIC_dca1.	0	0.90	0.119	0.476(248)	0.435(970)	0.168	0.108
dan_daner_ANX_PANIC_gmdc.	0	0.92	0.22	0.461(247)	0.438(537)	0.144	0.117
dan_daner_ANX_PANIC_gom3.	0	0.92	0.72	0.429(472)	0.433(1803)	−0.0281	0.0784
dan_daner_ANX_PANIC_gwc3.	1	0.96	0.601	0.448(280)	0.433(855)	0.0559	0.107
dan_daner_ANX_PANIC_swdf.	0	0.92	0.011	0.425(561)	0.456(2591)	−0.182	0.0716
dan_daner_ANX_QIMR_AGDS.	0	0.97	0.84	0.440(7312)	0.440(12813)	−0.00586	0.029
dan_daner_ANX_QIMR_Ad.	0	0.97	0.282	0.437(2449)	0.437(6343)	−0.0399	0.0371
dan_daner_ANX_QIMR_Nuup.	0	0.97	0.989	0.412(1131)	0.412(1233)	−0.000892	0.0649
dan_daner_ANX_SWE_Catss.	0	1.00	0.0353	0.440(540)	0.440(2333)	0.158	0.075
dan_daner_ANX_SWE_Salty.	0	1.00	0.129	0.443(687)	0.443(3445)	−0.0983	0.0647
dan_daner_ANX_SWE_TwinGene.	0	1.00	0.963	0.454(1740)	0.454(6470)	−0.00182	0.039
dan_daner_ANX_UKBB.	0	0.98	0.0255	0.436(25453)	0.436(58113)	−0.0252	0.0113
dan_daner_ANX_Utah.	0	0.95	0.0659	0.455(258)	0.401(279)	0.235	0.128
dan_daner_ANX_alspac.	0	1.00	0.746	0.430(536)	0.439(3404)	−0.0218	0.0672
dan_daner_ANX_iPSYCH.	0	0.97	0.604	0.423(5288)	0.427(14422)	−0.0123	0.0237
dan_daner_ANX_musp.	0	0.91	0.863	0.434(316)	0.428(715)	0.0176	0.102
**dan_**	**2**	**0.98**	**2.77e−08**	**0.447(122225)**	**0.461(729960)**	**−0.0277**	**0.005**

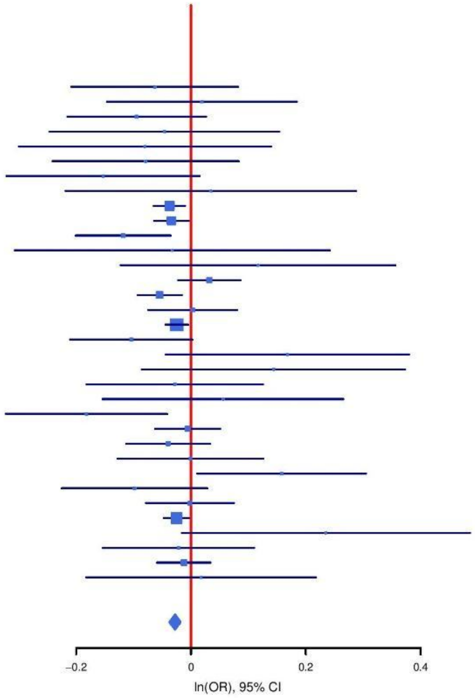

Supplementary Figure 53: Regional association plot (A) and forest plot (B) of SNP rs6574271. See full figure description on page 30.

A

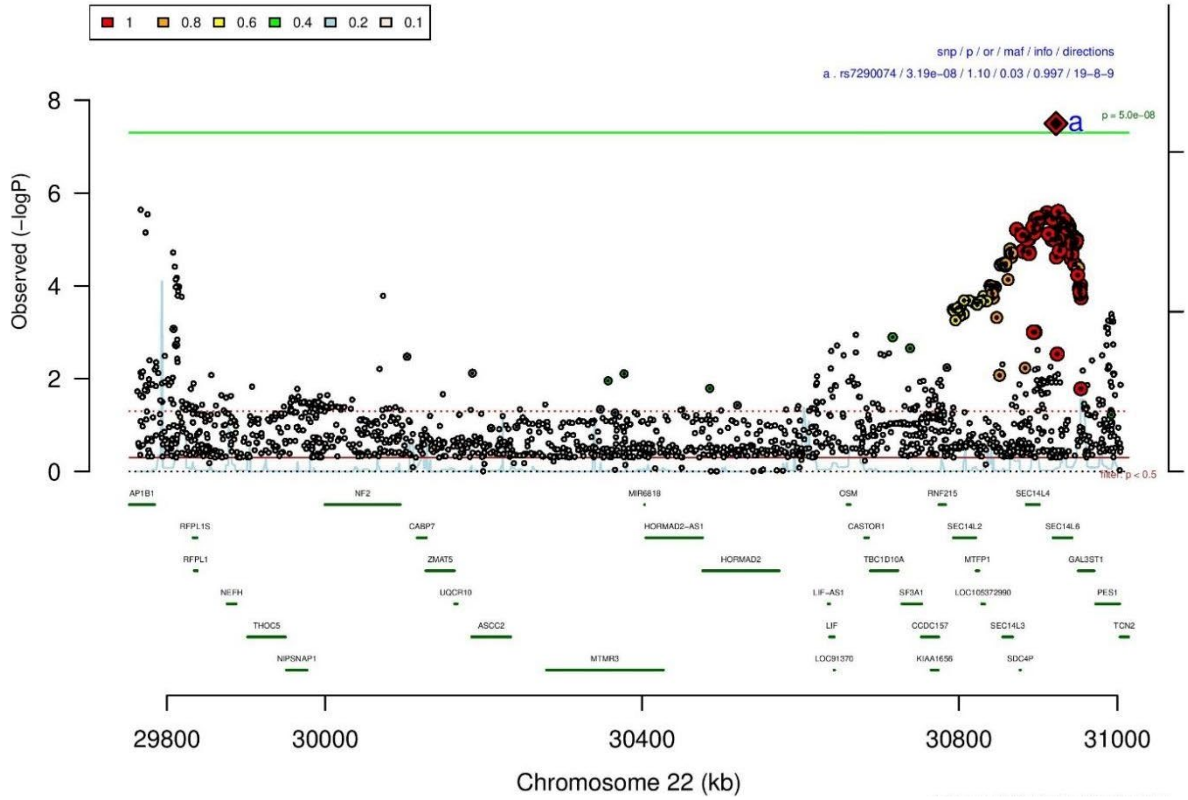

B

rs7290074 A/G 22:30922642

+????????+?++–+++++––+–++–+–++––++++ het_P: 0.095 het_I: 27.4

	ngt	info	p_value	f_ca(n)	f_co(n)	ln(OR)	STDerr
dan_daner_ANX_FinnGen.	0	0.99	0.000871	0.058(9182)	0.058(160108)	0.105	0.0315
dan_daner_ANX_GSC.	0	1.00	0.00334	0.020(1306)	0.013(12675)	0.478	0.155
dan_daner_ANX_Gedi_CHDS.	0	1.00	0.349	0.012(244)	0.012(217)	0.659	0.704
dan_daner_ANX_Gedi_GSMS.	0	1.00	0.76	0.018(208)	0.018(551)	−0.14	0.458
dan_daner_ANX_Gedi_VTSABD.	0	1.00	0.0388	0.024(224)	0.024(406)	0.188	0.0905
dan_daner_ANX_HUNT.	0	1.00	0.955	0.023(6587)	0.023(40740)	0.0036	0.0636
dan_daner_ANX_MGHI.	0	1.00	0.241	0.021(1823)	0.018(5151)	0.163	0.139
dan_daner_ANX_MVP.	0	1.00	0.0403	0.018(28525)	0.018(163731)	0.0702	0.0342
dan_daner_ANX_MoBa.	0	1.00	0.758	0.024(715)	0.026(22168)	−0.0521	0.169
dan_daner_ANX_PANIC_dca1.	0	1.05	0.673	0.022(248)	0.026(970)	−0.14	0.332
dan_daner_ANX_PANIC_gmdc.	0	0.95	0.945	0.019(247)	0.019(537)	0.0288	0.415
dan_daner_ANX_PANIC_gom3.	0	0.96	0.00598	0.028(472)	0.015(1803)	0.702	0.255
dan_daner_ANX_PANIC_gwc3.	0	0.97	0.842	0.019(280)	0.023(855)	−0.075	0.377
dan_daner_ANX_PANIC_swdf.	0	0.99	0.11	0.037(561)	0.026(2591)	0.3	0.187
dan_daner_ANX_PBK1.	0	1.00	0.736	0.018(1121)	0.019(11897)	−0.0557	0.165
dan_daner_ANX_QIMR_AGDS.	0	0.97	0.219	0.018(7312)	0.018(12813)	0.135	0.11
dan_daner_ANX_QIMR_Ad.	0	0.97	0.382	0.016(2449)	0.016(6343)	0.125	0.142
dan_daner_ANX_QIMR_Nuup.	0	0.97	0.0583	0.017(1131)	0.017(1233)	−0.469	0.248
dan_daner_ANX_SWE_Catss.	0	1.00	0.199	0.032(540)	0.032(2333)	−0.275	0.214
dan_daner_ANX_SWE_Salty.	0	1.00	0.142	0.032(687)	0.032(3445)	0.235	0.16
dan_daner_ANX_SWE_TwinGene.	0	1.00	0.271	0.030(1740)	0.030(6470)	0.12	0.109
dan_daner_ANX_UKBB.	0	1.00	0.164	0.016(25453)	0.016(58113)	0.0607	0.0436
dan_daner_ANX_Utah.	0	0.98	0.539	0.023(258)	0.018(279)	0.27	0.44
dan_daner_ANX_alspac.	0	0.99	0.0123	0.027(536)	0.016(3404)	0.572	0.218
dan_daner_ANX_bioVU.	0	1.00	0.0548	0.018(7586)	0.018(50545)	0.137	0.0715
dan_daner_ANX_iPSYCH.	0	1.00	0.477	0.025(5288)	0.024(14422)	0.0529	0.0744
dan_daner_ANX_musp.	0	0.98	0.75	0.014(316)	0.016(715)	−0.127	0.401
**dan_**	**0**	**1.00**	**3.19e−08**	**0.022(122225)**	**0.030(729960)**	**0.091**	**0.0164**

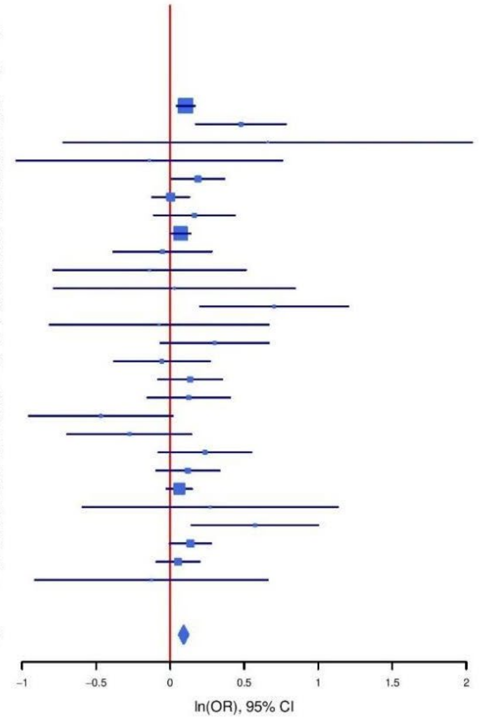

Supplementary Figure 54: Regional association plot (A) and forest plot (B) of SNP rs7290074. See full figure description on page 30.

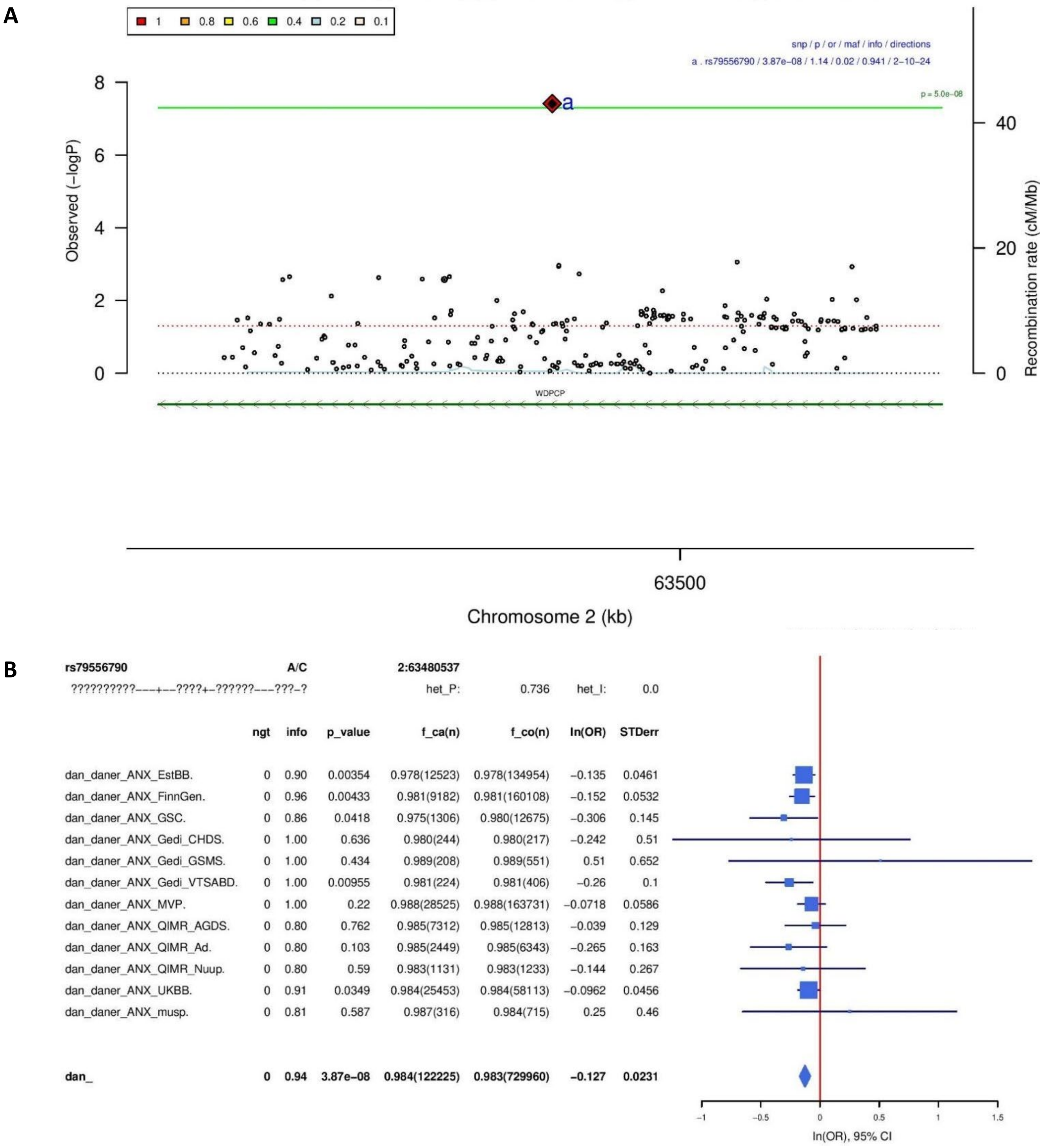

Supplementary Figure 55: Regional association plot (A) and forest plot (B) of SNP rs79556790. See full figure description on page 30.

A

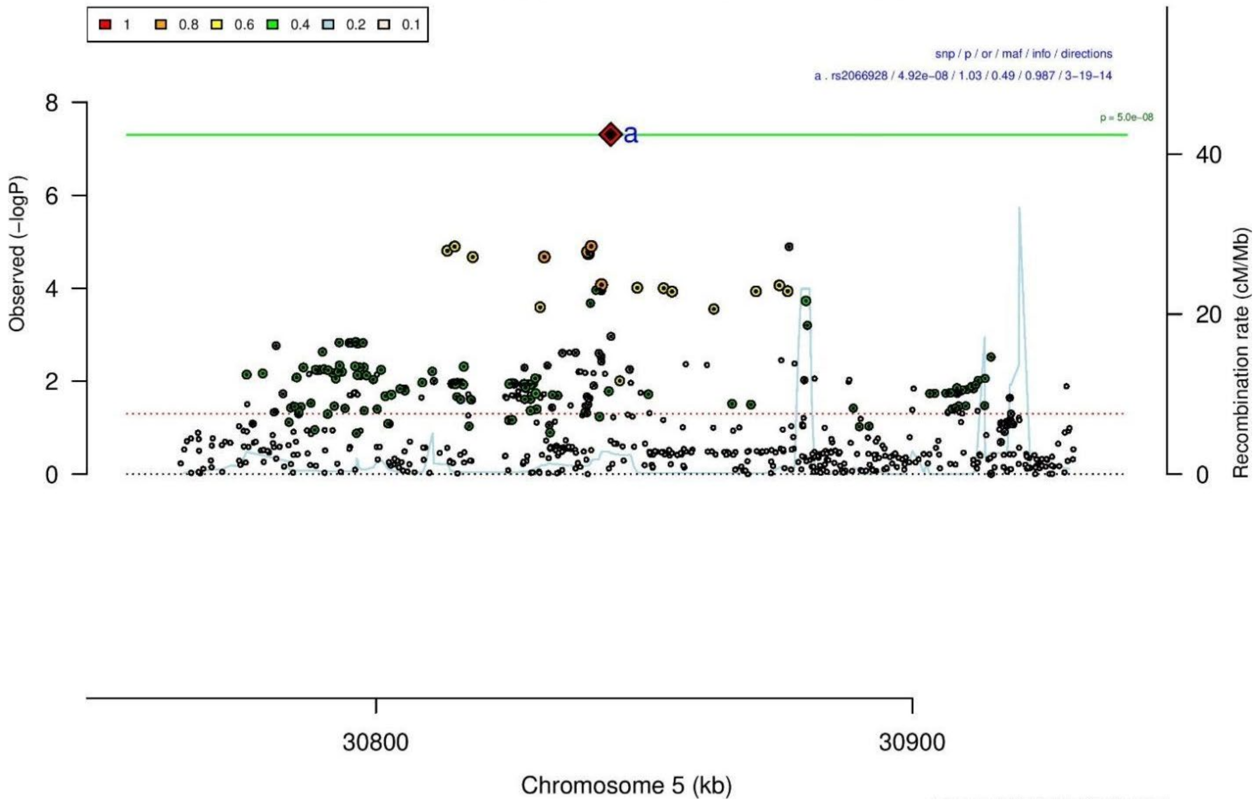

B

rs2066928 A/G 5:30843787

?????????-----++---------?????----------+ het_P: 0.836 het_I: 0.0

	ngt	info	p_value	f_ca(n)	f_co(n)	ln(OR)	STDerr
dan_daner_ANX_EstBB.	0	0.98	0.0469	0.566(12523)	0.566(134954)	−0.0271	0.0136
dan_daner_ANX_FinnGen.	0	0.99	0.0477	0.506(9182)	0.506(160108)	−0.0305	0.0154
dan_daner_ANX_GSC.	0	0.97	0.243	0.460(1306)	0.474(12675)	−0.0498	0.0427
dan_daner_ANX_Gedi_CHDS.	0	1.00	0.753	0.489(244)	0.489(217)	−0.0434	0.138
dan_daner_ANX_Gedi_GSMS.	0	1.00	0.318	0.453(208)	0.453(551)	0.118	0.119
dan_daner_ANX_Gedi_VTSABD.	0	1.00	0.901	0.463(224)	0.463(406)	0.00339	0.0273
dan_daner_ANX_HUNT.	0	0.98	0.0173	0.455(6587)	0.464(40740)	−0.0467	0.0196
dan_daner_ANX_MGHI.	0	0.95	0.142	0.459(1823)	0.474(5151)	−0.0584	0.0397
dan_daner_ANX_MVP.	0	1.00	0.269	0.500(28525)	0.500(163731)	−0.0107	0.0096
dan_daner_ANX_MoBa.	0	1.00	0.181	0.447(715)	0.464(22168)	−0.0728	0.0544
dan_daner_ANX_PBK1.	0	1.00	0.0995	0.474(1121)	0.494(11897)	−0.0727	0.0441
dan_daner_ANX_QIMR_AGDS.	0	0.95	0.0718	0.483(7312)	0.483(12813)	−0.0524	0.0291
dan_daner_ANX_QIMR_Ad.	0	0.95	0.53	0.478(2449)	0.478(6343)	−0.0228	0.0364
dan_daner_ANX_QIMR_Nuup.	0	0.95	0.309	0.474(1131)	0.474(1233)	−0.064	0.0629
dan_daner_ANX_SWE_Catss.	0	1.00	0.213	0.527(540)	0.527(2333)	−0.0902	0.0723
dan_daner_ANX_SWE_Salty.	0	1.00	0.744	0.509(687)	0.509(3445)	−0.0207	0.0634
dan_daner_ANX_SWE_TwinGene.	0	1.00	0.519	0.511(1740)	0.511(6470)	−0.0259	0.0401
dan_daner_ANX_UKBB.	0	0.97	0.00357	0.474(25453)	0.474(58113)	−0.0331	0.0113
dan_daner_ANX_Utah.	0	1.00	0.128	0.476(258)	0.432(279)	0.189	0.124
dan_daner_ANX_bioVU.	0	1.00	0.276	0.483(7586)	0.483(50545)	−0.0205	0.0188
dan_daner_ANX_iPSYCH.	0	0.98	0.195	0.493(5288)	0.499(14422)	−0.0301	0.0232
dan_daner_ANX_musp.	0	0.96	0.705	0.457(316)	0.466(715)	−0.0372	0.0983
**dan_**	**0**	**0.99**	**4.92e−08**	**0.494(122225)**	**0.506(729960)**	**−0.0265**	**0.0049**

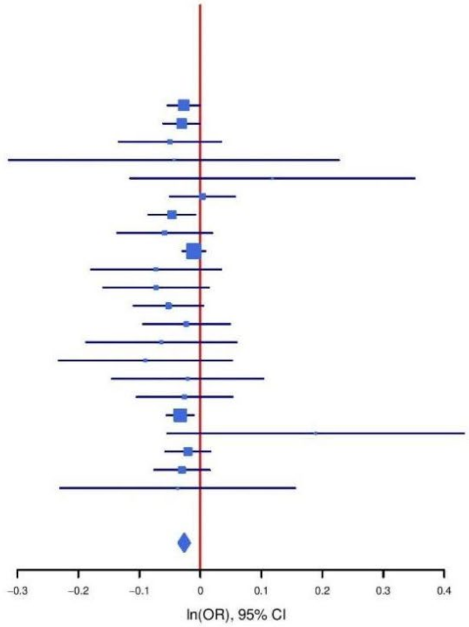

Supplementary Figure 56: Regional association plot (A) and forest plot (B) of SNP rs2066928. See full figure description on page 30.

Heterogeneity test

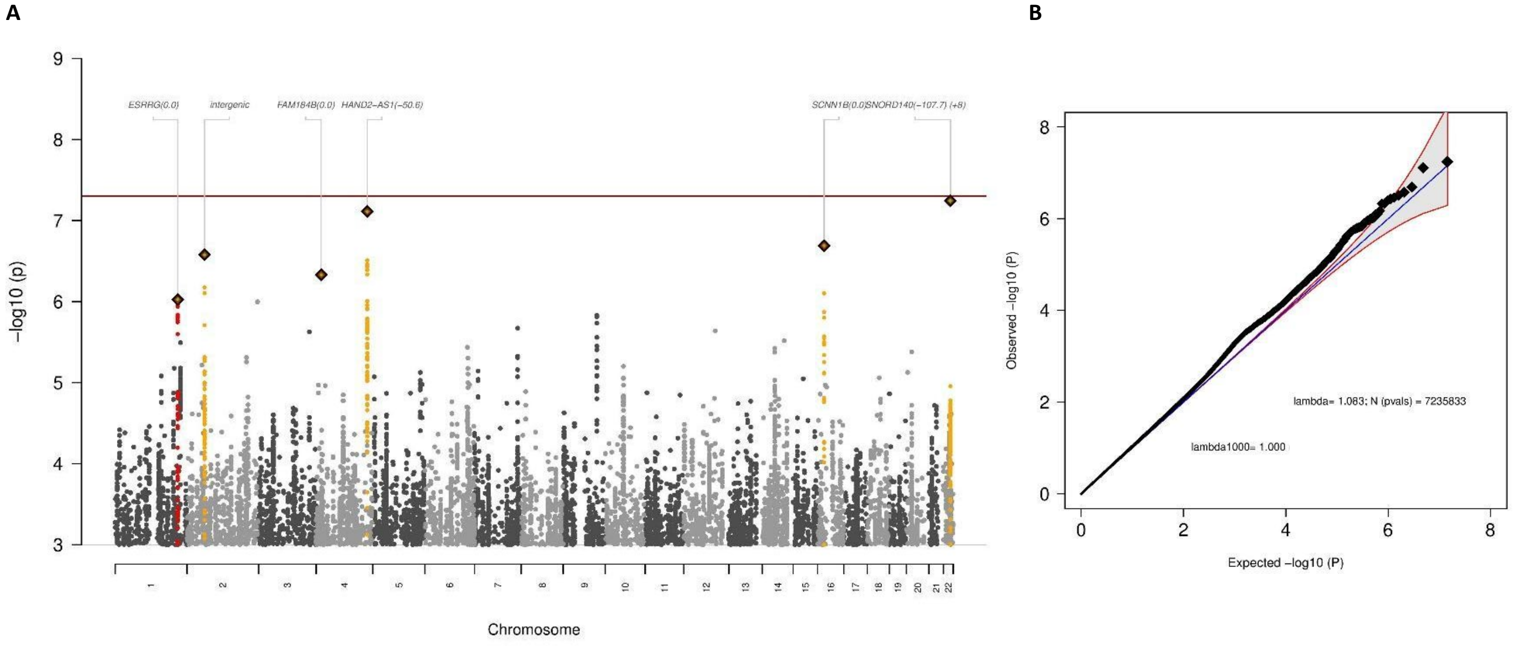

Supplementary Figure 57: Manhattan-plot (A) and QQ-plot (B) of heterogeneity test, indicating whether SNPs across the genome are significantly heterogeneously associated with some cohorts but not others for the entire ANX dataset (122,341 ANX cases and 729,881 unaffected controls). METAL implements Cochran's Q-test for heterogeneity, which follows a chi-square distribution. A higher Q-statistic suggests greater variation in SNP effects across studies, while a low p-value indicates statistically significant heterogeneity. A) The x-axis shows the position in the genome (chromosome 1 to 22), the y-axis represents –log10 p-values (two sided, not adjusted for multiple testing) of the heterogeneity test. The horizontal red line shows the threshold for genome-wide significance (P = 5x10-08). Dots represent each SNP that was tested in the GWAS with a diamond indicating the lead SNP of the genomic loci with the lowest p-value in the heterogeneity test. B) The expected -log10(p) under the null (blue line) is plotted against the observed -log10(p) (black dots). The shading indicates the 95% confidence region under the null. Lambda and Lambda1000 (which is the Lambda if the GWAS contained 1000 cases and 1000 controls) indicate genomic inflation factors. Number of SNPs (N (pvals)) and Number of cases and controls are given in parentheses.

Manhattan-plots and QQ-plots of sub-group analyses

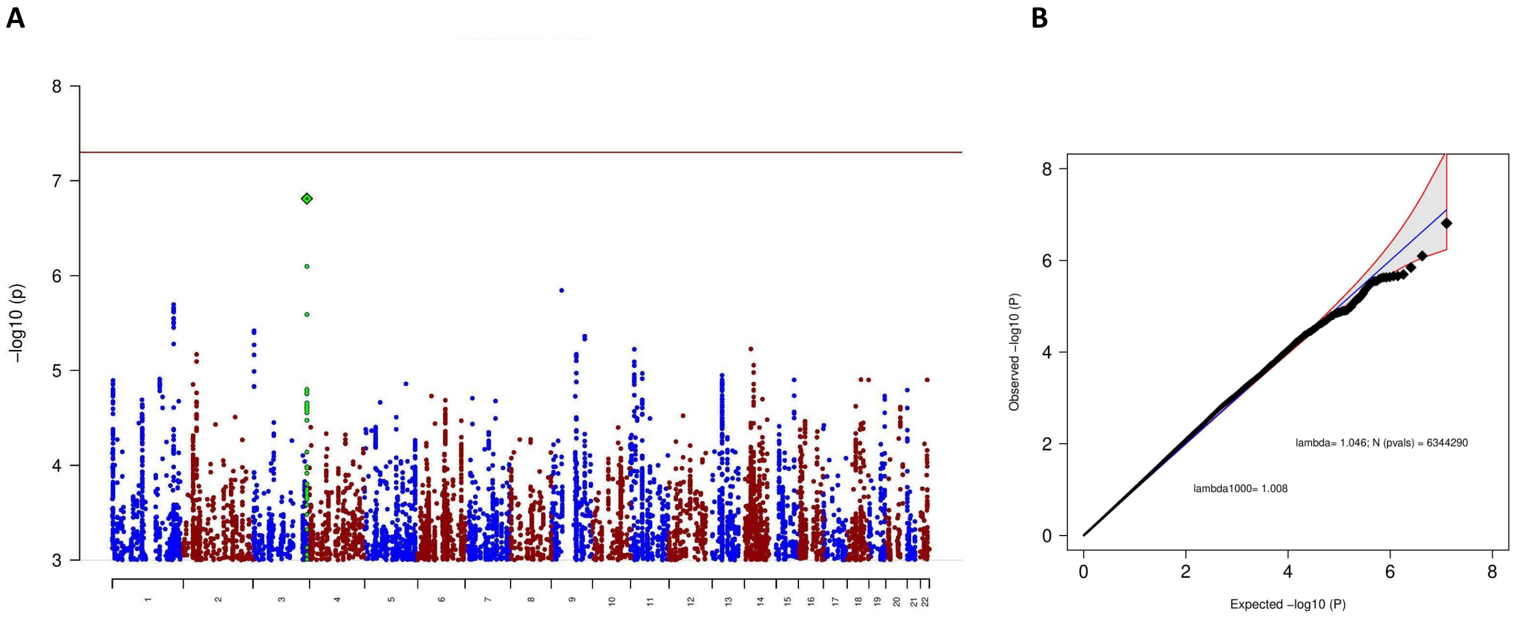

Supplementary Figure 58: Manhattan-plot (A) and QQ-plot (B) of the sub-group *ascertainment*-specific GWAS analysis including only clinical cohorts (Ncases = 3631, Ncontrols = 11907). The analysis aimed to find ascertainment-specific signals associated with ANX specifically in clinical cohorts. (A) The x-axis shows the position in the genome (chromosome 1 to 22), the y-axis represents –log10 p-values (two sided, not adjusted for multiple testing) for the association of variants with ANX from meta-analysis using an inverse-variance weighted fixed effects model. The horizontal red line shows the threshold for genome-wide significance (5x10-08). Each dot represents one SNP that was tested in the GWAS, with a green diamond indicating the lead SNP of a genome-wide significant locus, with green dots below belonging to that locus. (B) the expected -log10(p) under the null is plotted against the observed -log10(p). The shading indicates the 95% confidence region under the null. Lambda and Lambda1000 (which is the Lambda if the GWAS contained 1000 cases and 1000 controls) indicate genomic inflation factors. Number of SNPs (N (pvals)) and Number of cases and controls are given in parentheses.

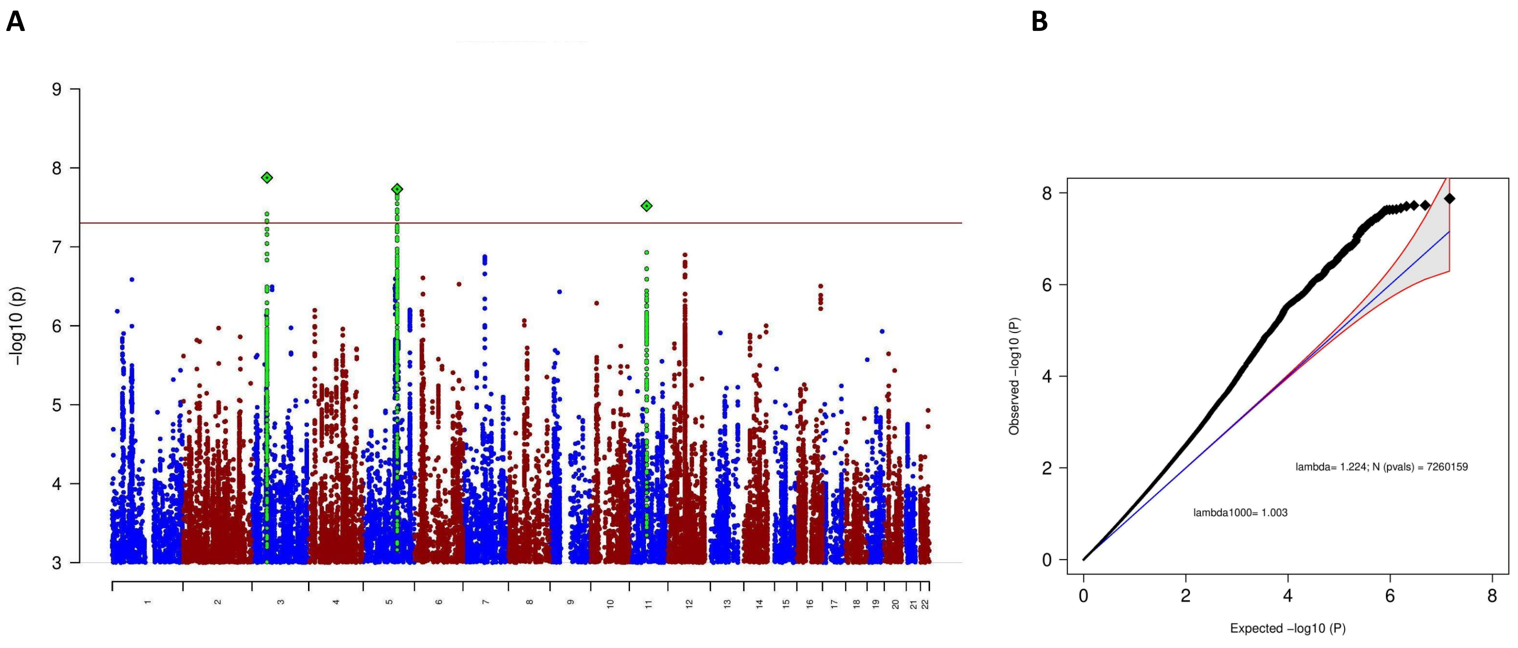

Supplementary Figure 59: Manhattan-plot (A) and QQ-plot (B) of the sub-group *ascertainment*-specific GWAS analysis including only biobank cohorts (Ncases = 37,714, Ncontrols = 420,412). The analysis aimed to find ascertainment-specific signals associated with ANX specifically in biobank cohorts. (A) The x-axis shows the position in the genome (chromosome 1 to 22), the y-axis represents –log10 p-values (two sided, not adjusted for multiple testing) for the association of variants with ANX from meta-analysis using an inverse-variance weighted fixed effects model. The horizontal red line shows the threshold for genome-wide significance (5x10-08). Each dot represents one SNP that was tested in the GWAS, with a green diamond indicating the lead SNP of a genome-wide significant locus, with green dots below belonging to that locus. (B) the expected -log10(p) under the null is plotted against the observed -log10(p). The shading indicates the 95% confidence region under the null. Lambda and Lambda1000 (which is the Lambda if the GWAS contained 1000 cases and 1000 controls) indicate genomic inflation factors. Number of SNPs (N (pvals)) and Number of cases and controls are given in parentheses.

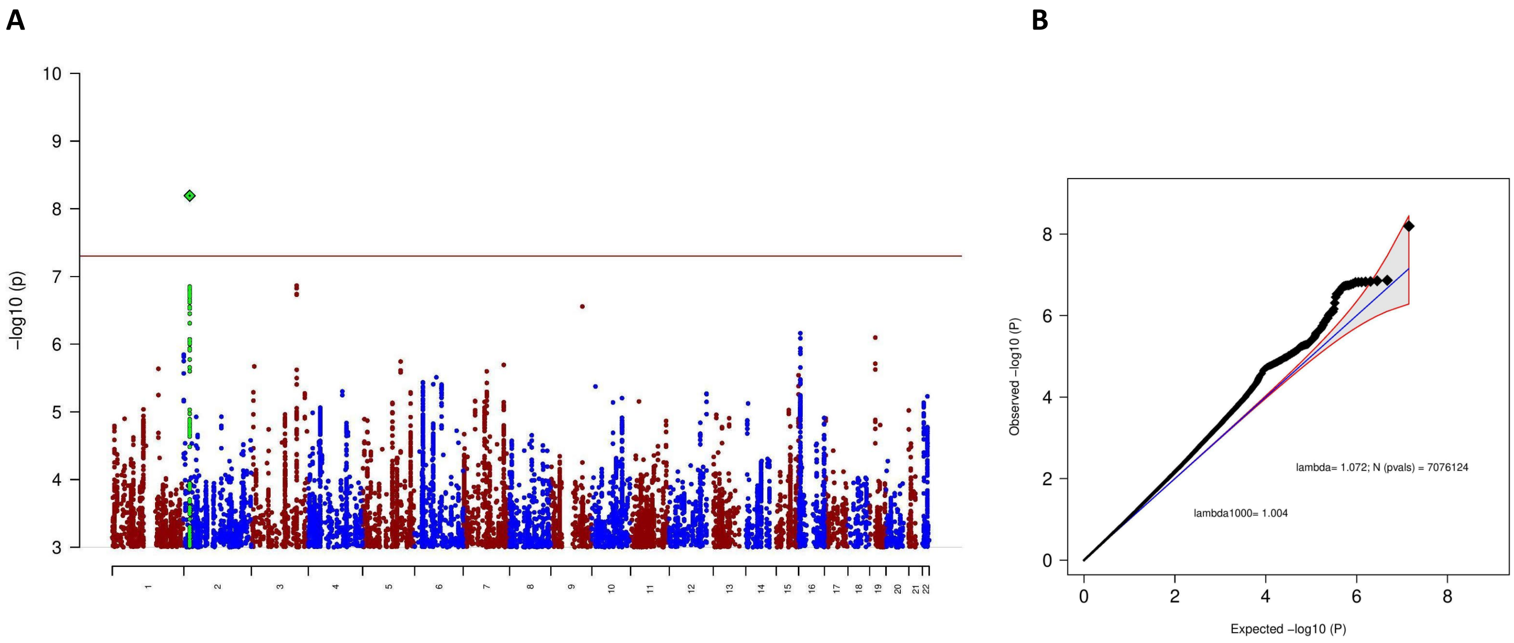

Supplementary Figure 60: Manhattan-plot (A) and QQ-plot (B) of the sub-group *ascertainment*-specific GWAS analysis including only comorbid cohorts (Ncases = 12,980, Ncontrols = 27,430). The analysis aimed to find ascertainment-specific signals associated with ANX specifically in comorbid cohorts. (A) The x-axis shows the position in the genome (chromosome 1 to 22), the y-axis represents –log10 p-values (two sided, not adjusted for multiple testing) for the association of variants with ANX from meta-analysis using an inverse-variance weighted fixed effects model. The horizontal red line shows the threshold for genome-wide significance (5x10-08). Each dot represents one SNP that was tested in the GWAS, with a green diamond indicating the lead SNP of a genome-wide significant locus, with green dots below belonging to that locus. (B) the expected -log10(p) under the null is plotted against the observed -log10(p). The shading indicates the 95% confidence region under the null. Lambda and Lambda1000 (which is the Lambda if the GWAS contained 1000 cases and 1000 controls) indicate genomic inflation factors. Number of SNPs (N (pvals)) and Number of cases and controls are given in parentheses.

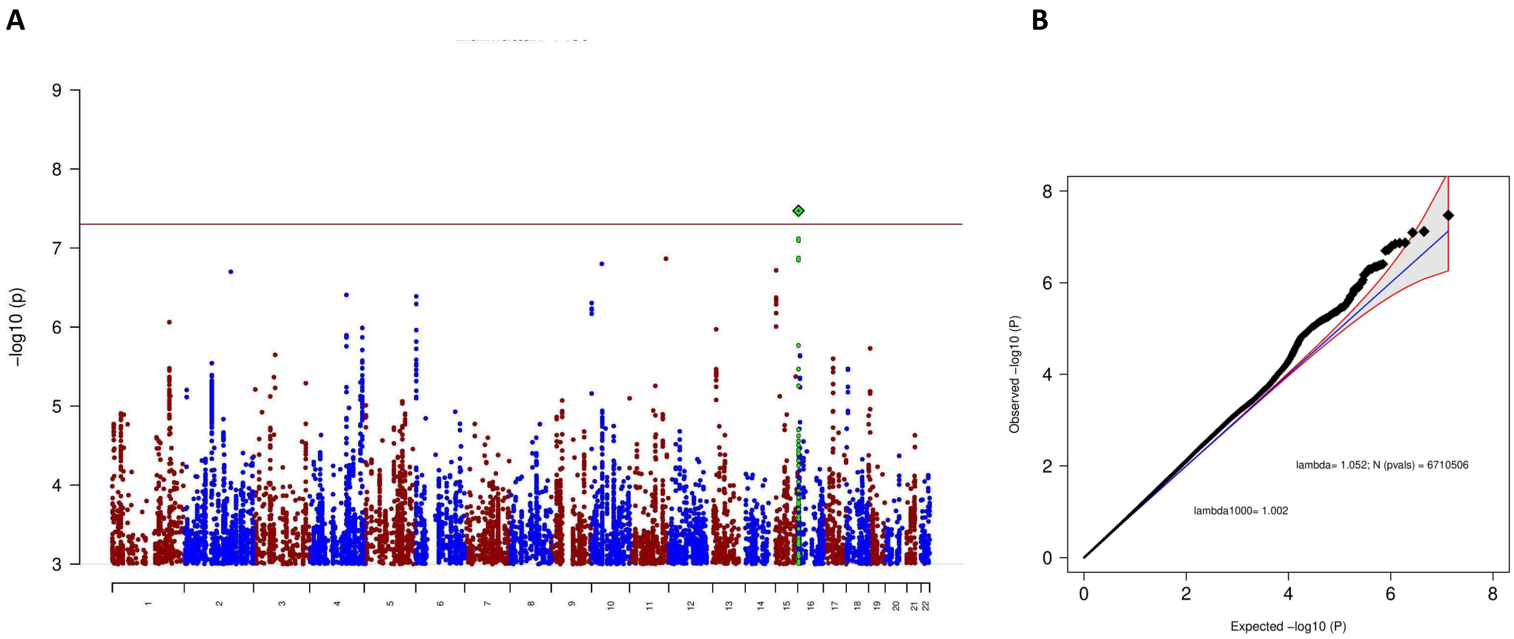

Supplementary Figure 61: Manhattan-plot (A) and QQ-plot (B) of the sub-group *ascertainment*-specific GWAS analysis including only community cohorts (Ncases = 14,038, Ncontrols = 48,288). The analysis aimed to find ascertainment-specific signals associated with ANX specifically in community cohorts. (A) The x-axis shows the position in the genome (chromosome 1 to 22), the y-axis represents –log10 p-values (two sided, not adjusted for multiple testing) for the association of variants with ANX from meta-analysis using an inverse-variance weighted fixed effects model. The horizontal red line shows the threshold for genome-wide significance (5x10-08). Each dot represents one SNP that was tested in the GWAS, with a green diamond indicating the lead SNP of a genome-wide significant locus, with green dots below belonging to that locus. (B) the expected -log10(p) under the null is plotted against the observed -log10(p). The shading indicates the 95% confidence region under the null. Lambda and Lambda1000 (which is the Lambda if the GWAS contained 1000 cases and 1000 controls) indicate genomic inflation factors. Number of SNPs (N (pvals)) and Number of cases and controls are given in parentheses.

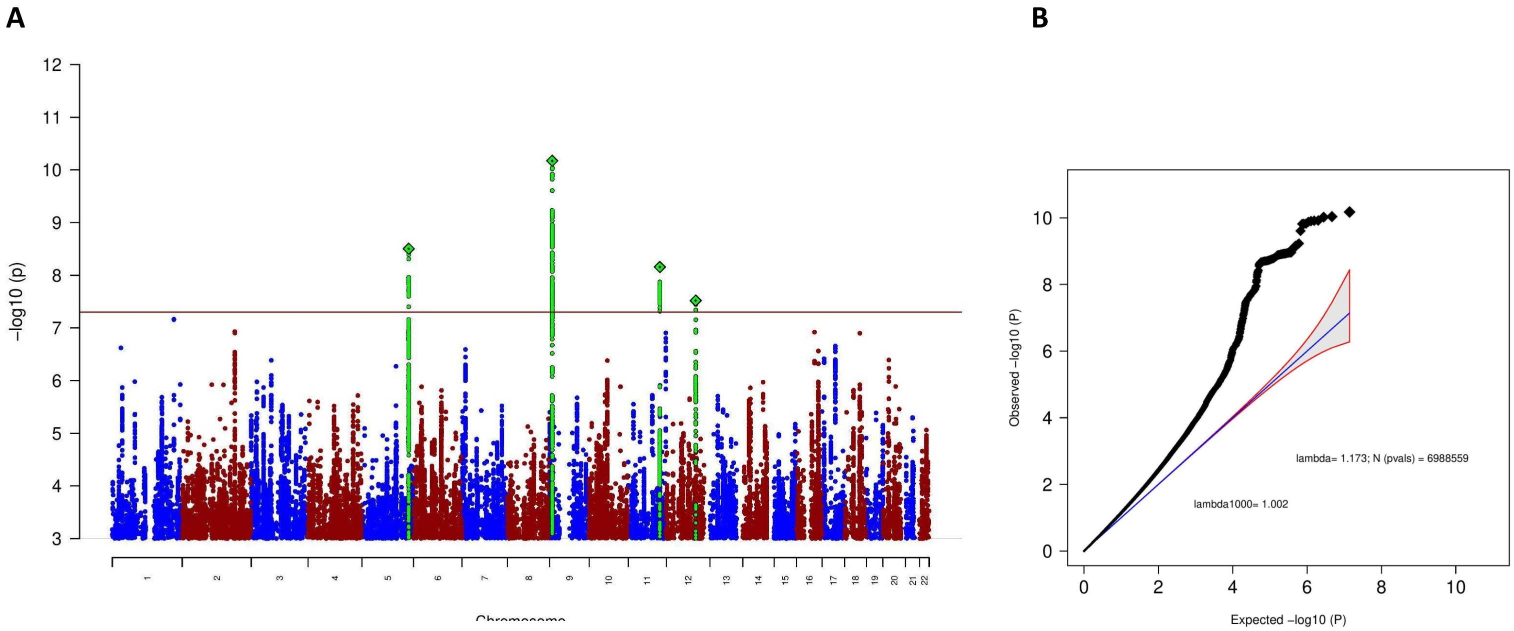

Supplementary Figure 62: Manhattan-plot (A) and QQ-plot (B) of the sub-group *ascertainment*-specific GWAS analysis including only self-reported diagnosis (SRPD) cohorts (Ncases = 53,978, Ncontrols = 221,844). The analysis aimed to find ascertainment-specific signals associated with ANX specifically in SRPD cohorts. (A) The x-axis shows the position in the genome (chromosome 1 to 22), the y-axis represents –log10 p-values (two sided, not adjusted for multiple testing) for the association of variants with ANX from meta-analysis using an inverse-variance weighted fixed effects model. The horizontal red line shows the threshold for genome-wide significance (5x10-08). Each dot represents one SNP that was tested in the GWAS, with a green diamond indicating the lead SNP of a genome-wide significant locus, with green dots below belonging to that locus. (B) the expected -log10(p) under the null is plotted against the observed -log10(p). The shading indicates the 95% confidence region under the null. Lambda and Lambda1000 (which is the Lambda if the GWAS contained 1000 cases and 1000 controls) indicate genomic inflation factors. Number of SNPs (N (pvals)) and Number of cases and controls are given in parentheses.

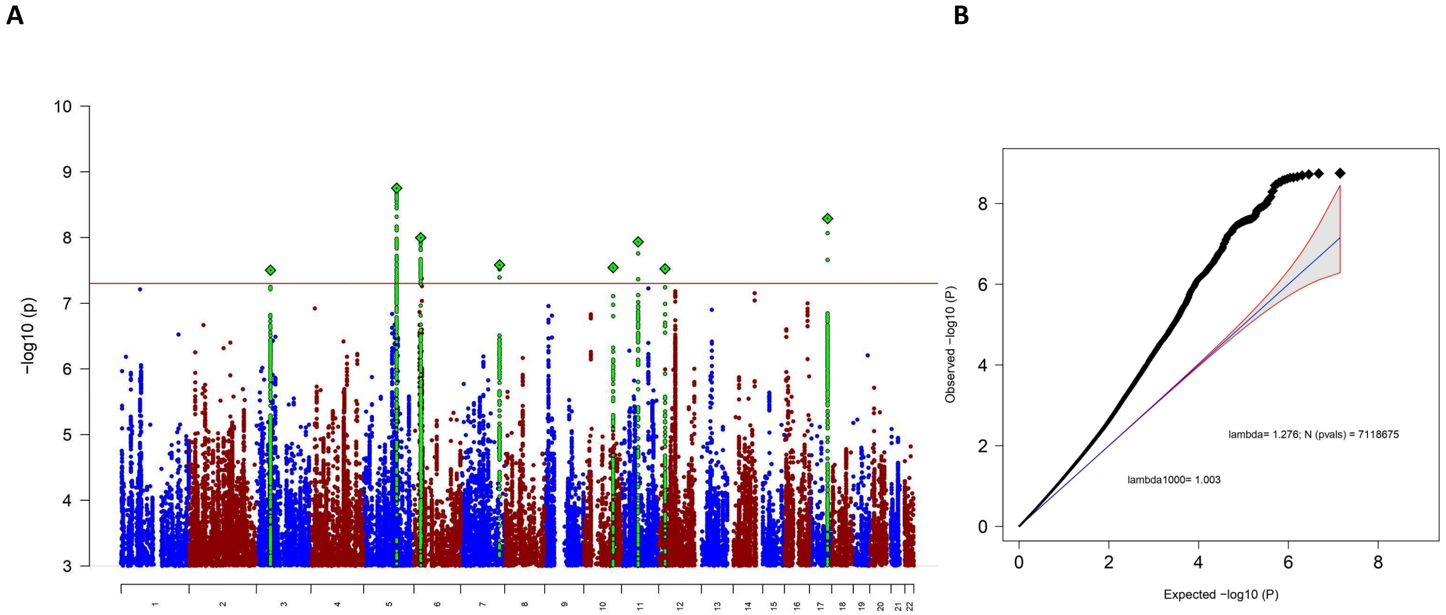

Supplementary Figure 63: Manhattan-plot (A) and QQ-plot (B) of the sub-group *assessment*-specific GWAS analysis including only ICD-10 cohorts (Ncases = 47,533, Ncontrols = 460,036). The analysis aimed to find ascertainment-specific signals associated with ANX specifically in SRPD cohorts. (A) The x-axis shows the position in the genome (chromosome 1 to 22), the y-axis represents –log10 p-values (two sided, not adjusted for multiple testing) for the association of variants with ANX from meta-analysis using an inverse-variance weighted fixed effects model. The horizontal red line shows the threshold for genome-wide significance (5x10-08). Each dot represents one SNP that was tested in the GWAS, with a green diamond indicating the lead SNP of a genome-wide significant locus, with green dots below belonging to that locus. (B) the expected -log10(p) under the null is plotted against the observed -log10(p). The shading indicates the 95% confidence region under the null. Lambda and Lambda1000 (which is the Lambda if the GWAS contained 1000 cases and 1000 controls) indicate genomic inflation factors. Number of SNPs (N (pvals)) and Number of cases and controls are given in parentheses.

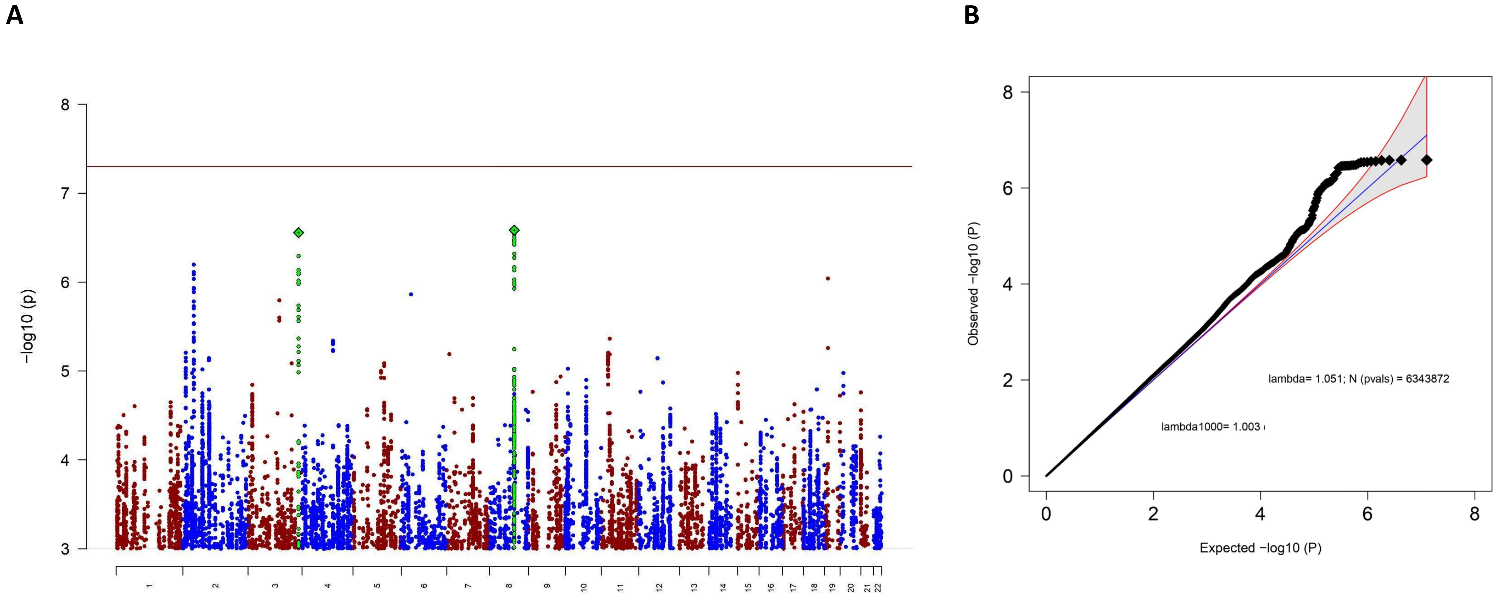

Supplementary Figure 64: Manhattan-plot (A) and QQ-plot (B) of the sub-group *assessment*-specific GWAS analysis including only Interview-based cohorts (Ncases = 13,396, Ncontrols = 35,272). The analysis aimed to find ascertainment-specific signals associated with ANX specifically in SRPD cohorts. (A) The x-axis shows the position in the genome (chromosome 1 to 22), the y-axis represents –log10 p-values (two sided, not adjusted for multiple testing) for the association of variants with ANX from meta-analysis using an inverse-variance weighted fixed effects model. The horizontal red line shows the threshold for genome-wide significance (5x10-08). Each dot represents one SNP that was tested in the GWAS, with a green diamond indicating the lead SNP of a genome-wide significant locus, with green dots below belonging to that locus. (B) the expected -log10(p) under the null is plotted against the observed -log10(p). The shading indicates the 95% confidence region under the null. Lambda and Lambda1000 (which is the Lambda if the GWAS contained 1000 cases and 1000 controls) indicate genomic inflation factors. Number of SNPs (N (pvals)) and Number of cases and controls are given in parentheses.

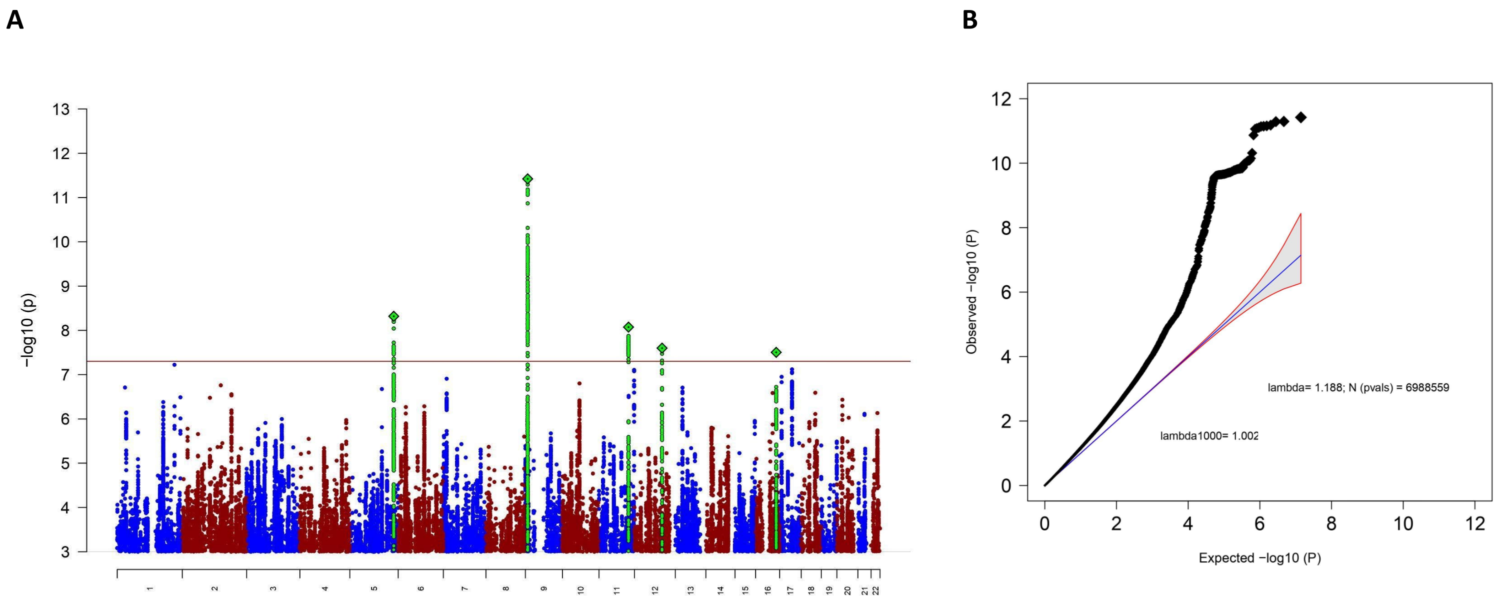

Supplementary Figure 65: Manhattan-plot (A) and QQ-plot (B) of the sub-group *assessment*-specific GWAS analysis including only self-reported diagnosis (SRPD) cohorts (Ncases = 61,412, Ncontrols = 234,573). The analysis aimed to find ascertainment-specific signals associated with ANX specifically in SRPD cohorts. (A) The x-axis shows the position in the genome (chromosome 1 to 22), the y-axis represents –log10 p-values (two sided, not adjusted for multiple testing) for the association of variants with ANX from meta-analysis using an inverse-variance weighted fixed effects model. The horizontal red line shows the threshold for genome-wide significance (5x10-08). Each dot represents one SNP that was tested in the GWAS, with a green diamond indicating the lead SNP of a genome-wide significant locus, with green dots below belonging to that locus. (B) the expected -log10(p) under the null is plotted against the observed -log10(p). The shading indicates the 95% confidence region under the null. Lambda and Lambda1000 (which is the Lambda if the GWAS contained 1000 cases and 1000 controls) indicate genomic inflation factors. Number of SNPs (N (pvals)) and Number of cases and controls are given in parentheses.

Number of non-spurious dimensions in exploratory genomic factor analysis (paLDSC)

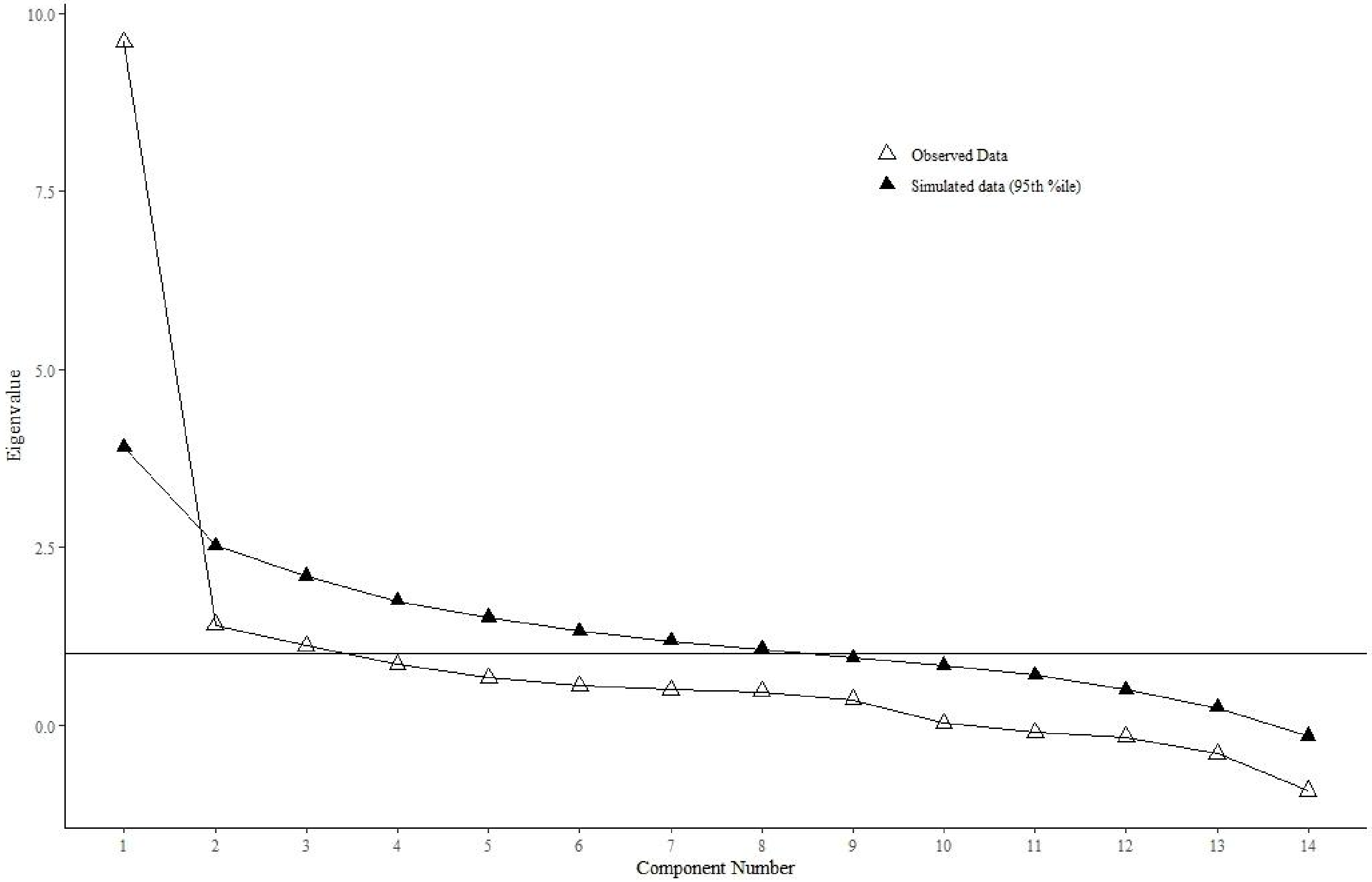

Supplementary Figure 66: results from the parallel analysis based on multivariate LDSC (paLDSC): paLDSC compares the eigenvalues generated from the eigendecomposition of the LDSC genetic correlation matrix (white triangles) to the eigenvalues of a Monte-Carlo simulated null correlation matrix with random noise drawn from the multivariate LDSC sampling distribution (black triangles). The suggested number of factors to be extracted (1) corresponds to an eigenvalue exceeding a pre-specified percentile (95%) from the corresponding distribution of eigenvalues generated under the null. The paLDSC output shows that the first component from the LDSC-derived genetic correlation matrix has a larger eigenvalue than 95% of the eigenvalues observed for the corresponding component from the null correlation matrix. As input we used the 14 largest cohorts from our GWAS meta-analysis (N_{Total} > 10,000 & N_{Cases} > 1000).

GenomicSEM

A

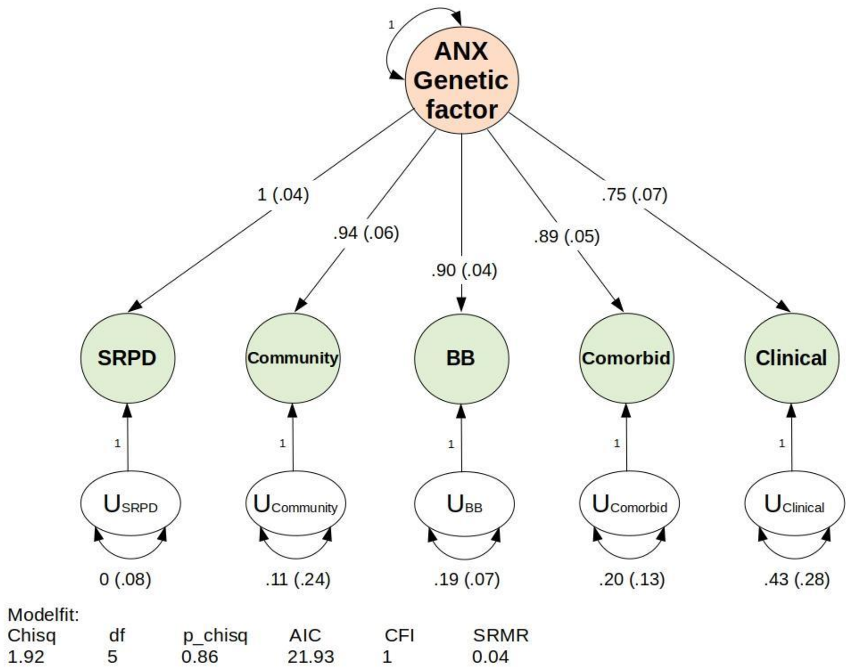

B

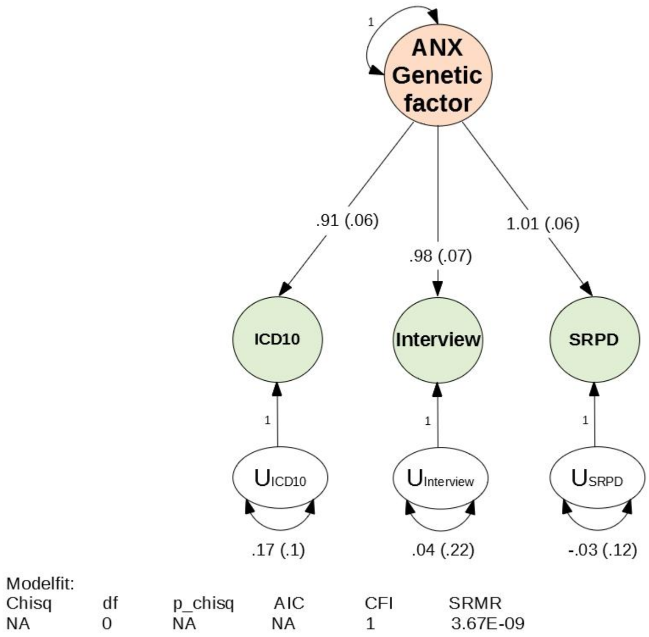

Supplementary Figure 67: Confirmatory Factor Analysis (CFA) of A) the five ANX ascertainment subgroups, and B) the three ANX assessment subgroups, using Genomic Structural Equation Modelling (GenomicSEM). Both plots depict a path diagram of a common-factor GenomicSEM model without SNP effects, specified with unit variance identification, fixing the variance of the common factor F1 to 1. All estimates are standardized. Model-fit indices below are Chi-square statistic (Chisq); degrees of

freedom of the model (df); p-value of the Chi-square (p_chisq); Akaike Information Criterion (AIC), which is a comparative measure of fit with lower values indicating a better fit; comparative fit index (CFI) which assesses the relative improvement in fit compared with the baseline model, ranging between 0 and 1; and standardized root mean square residual (SRMR), which is an absolute measure of fit defined as the standardized difference between observed correlation and the predicted correlation with a value of 0 indicating perfect fit.

Characterization of GWAS SNPs

Supplementary Figures 68-87: FUMA circos plots for the genome-wide significant loci from the main ANX GWAS meta-analsis (Ncases = 122,341, Ncontrols = 729,881). The outermost layer is a Manhattan plot; only SNPs with P < 0.05 are displayed. SNPs in genomic risk loci are color-coded as a function of their maximum r2 to one of the independent significant SNPs in the locus, as follows: red (r2 > 0.8), orange (r2 > 0.6), green (r2 > 0.4) and blue (r2 > 0.2). SNPs that are not in LD with any of the independent significant SNPs (with r2 ≤ 0.2) are grey. The rsID of the top SNPs in each risk locus are displayed in the outermost layer. The Y-axis is between 0 to the maximum -log10(P-value) of the SNPs. The second layer is the chromosome ring. Genomic risk loci are highlighted in blue. Next are mapped genes by chromatin interactions or eQTLs. Only mapped genes by either chromatin interaction and/or eQTLs (conditional on user defined parameters) are displayed. If the gene is mapped only by chromatin interactions or only by eQTLs, it is colored orange or green, respectively. When the gene is mapped by both, it is colored red. The third layer is again a chromosome ring. This is the same as the second layer but without coordinates to make it easy to align the position of genes with genomic coordinates.

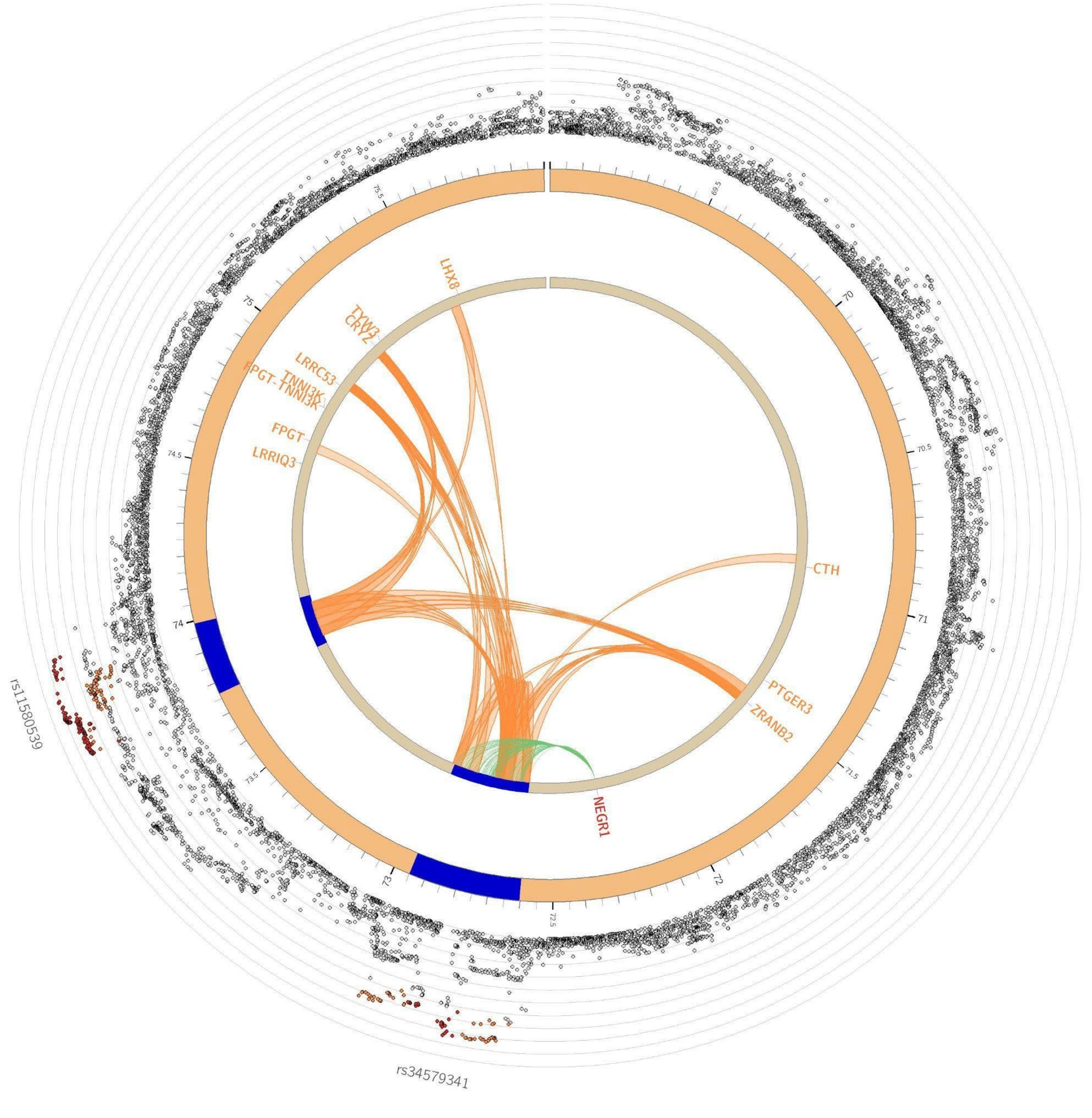

Supplementary Figure 68: Circos plot of chromosome 1

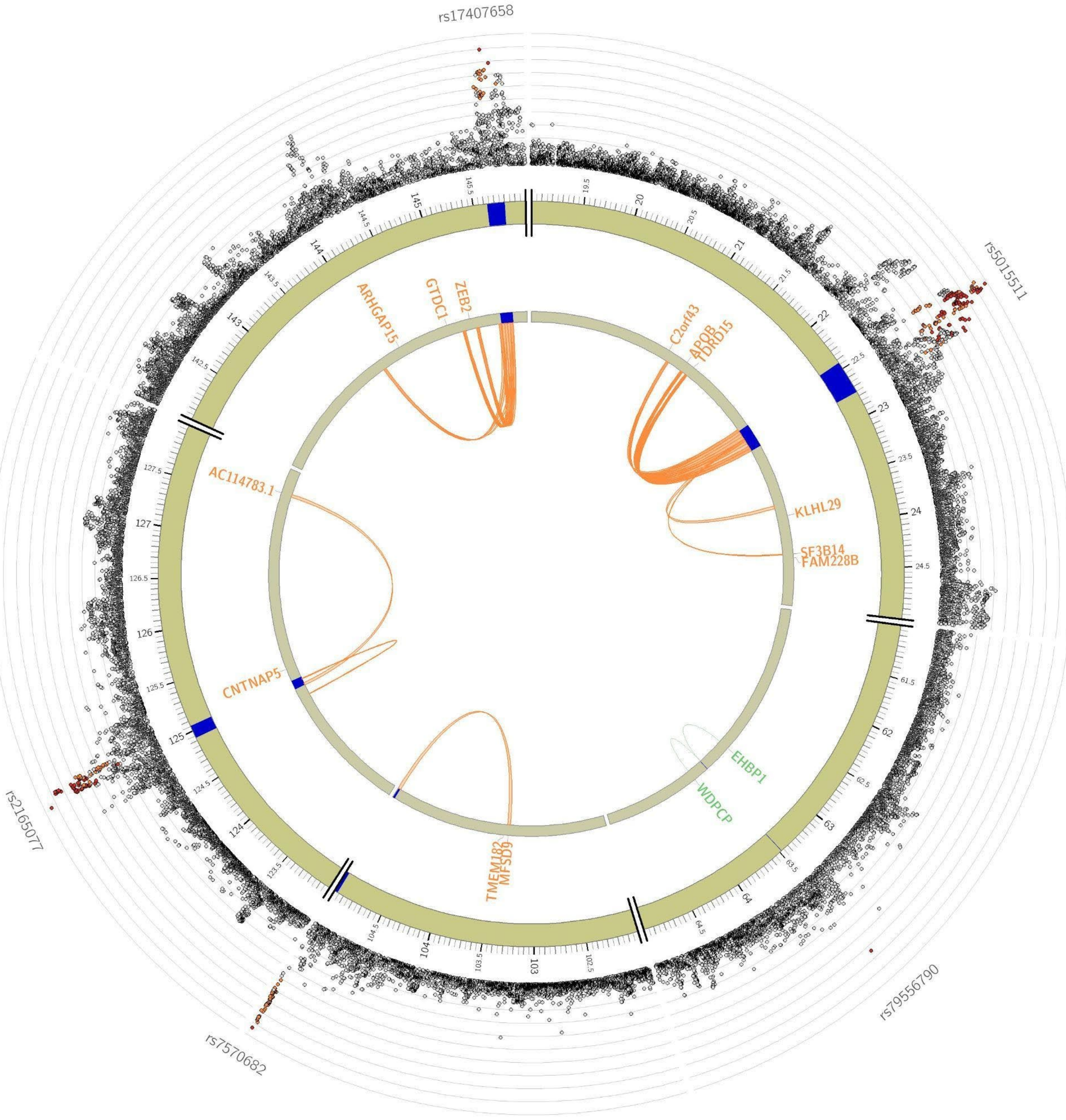

Supplementary Figure 69: Circos plot of chromosome 2

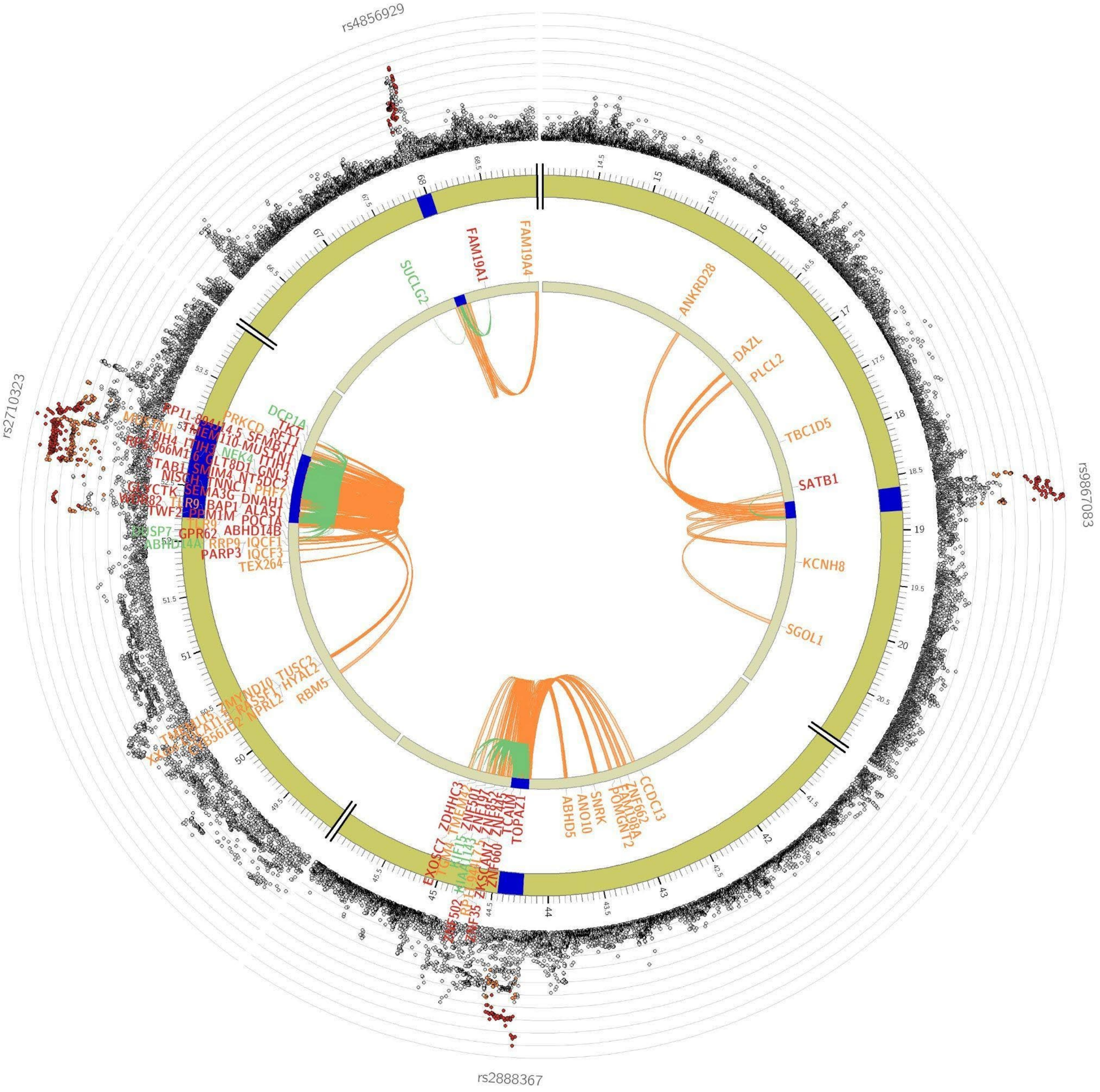

Supplementary Figure 70: Circos plot of chromosome 3

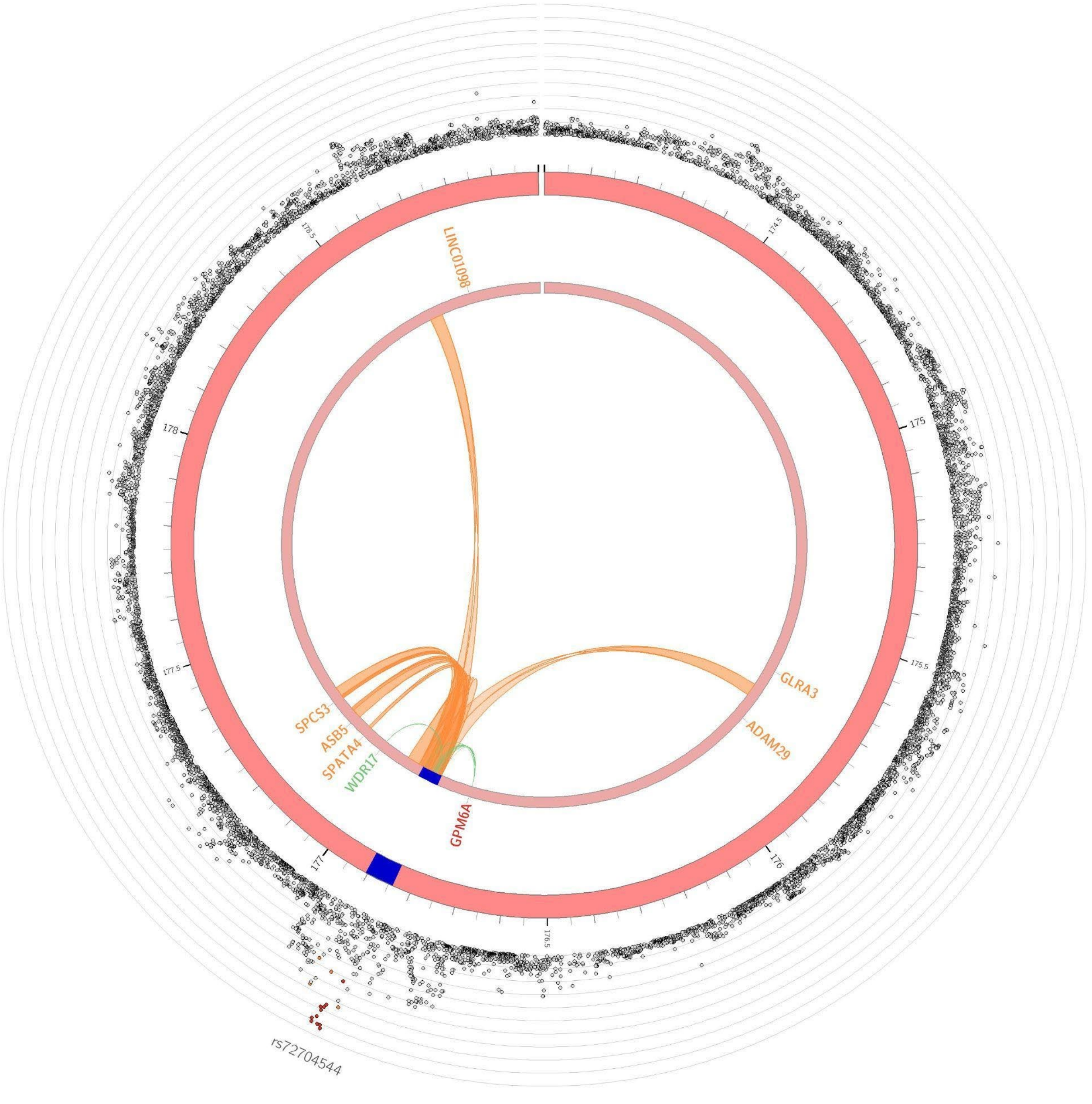

Supplementary Figure 71: Circos plot of chromosome 4

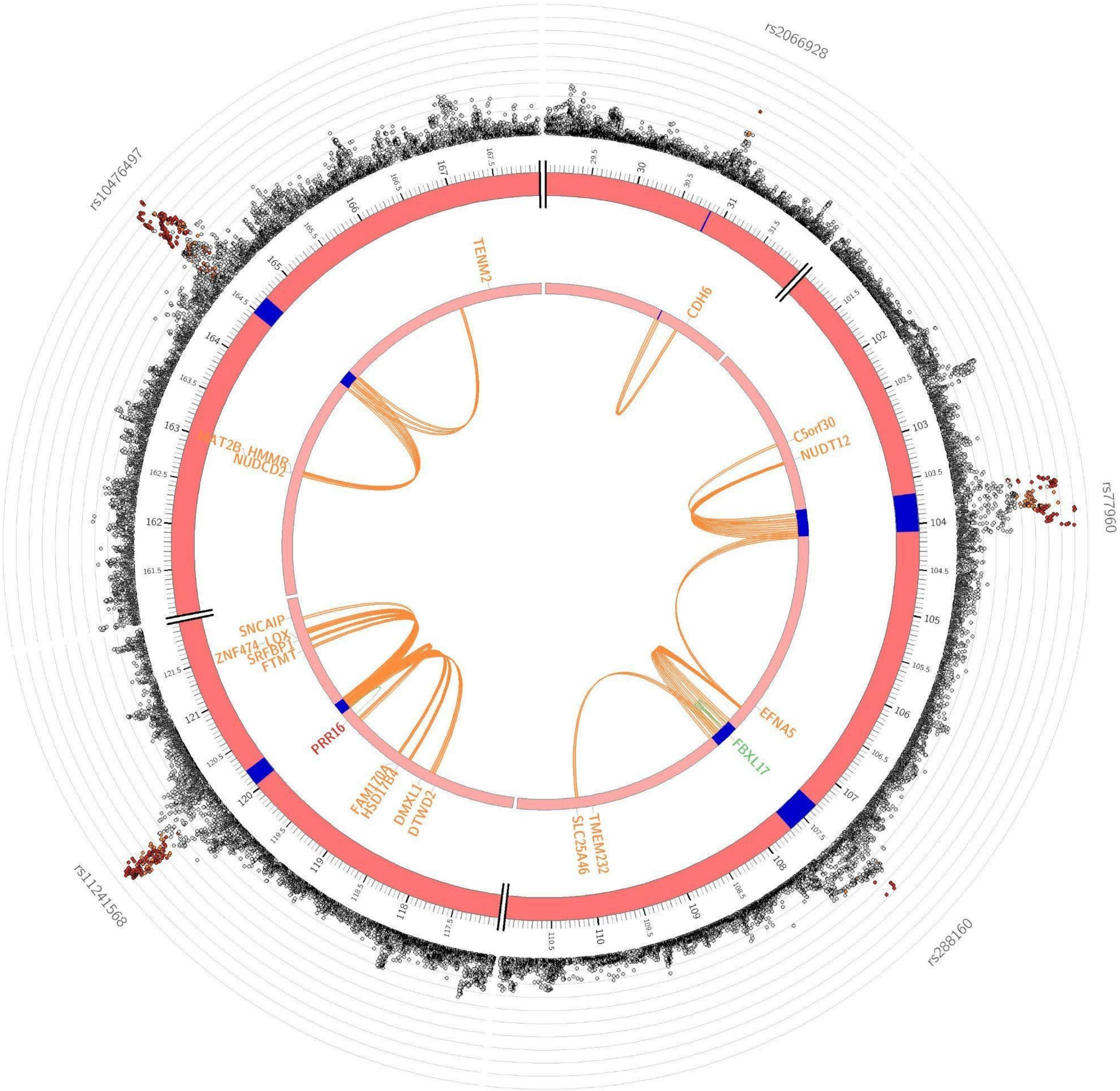

Supplementary Figure 72: Circos plot of chromosome 5

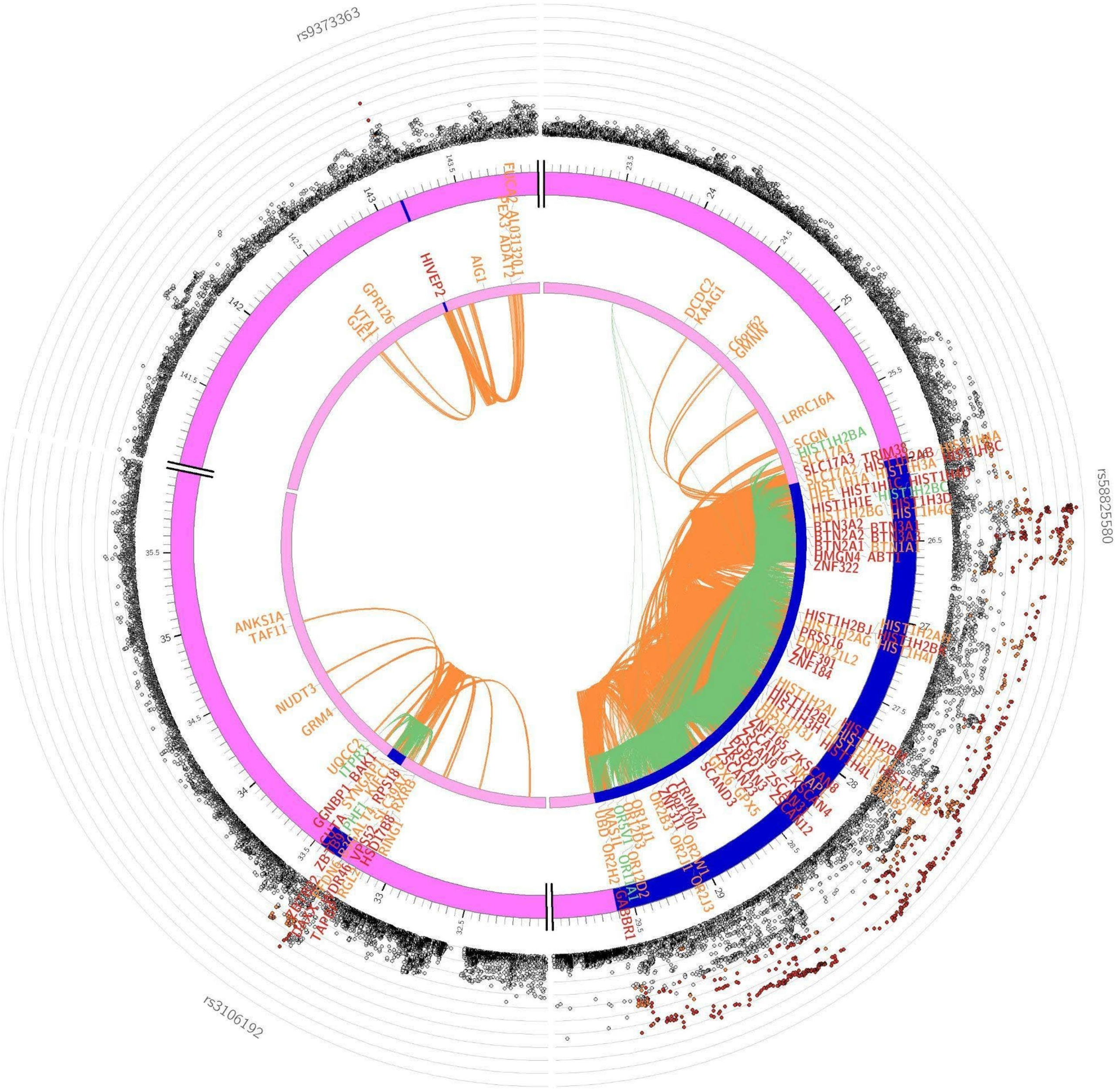

Supplementary Figure 73: Circos plot of chromosome 6

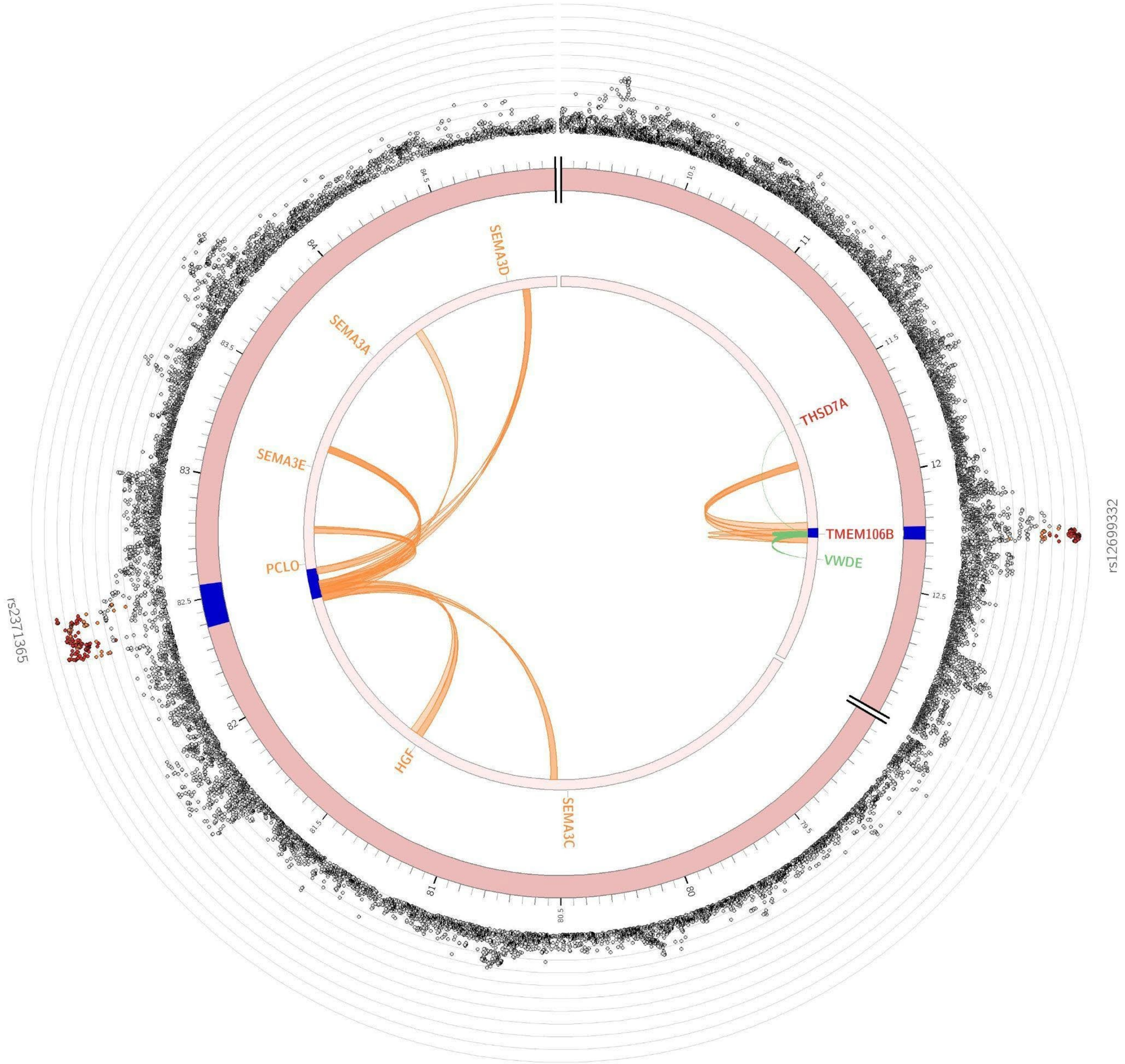

Supplementary Figure 74: Circos plot of chromosome 7

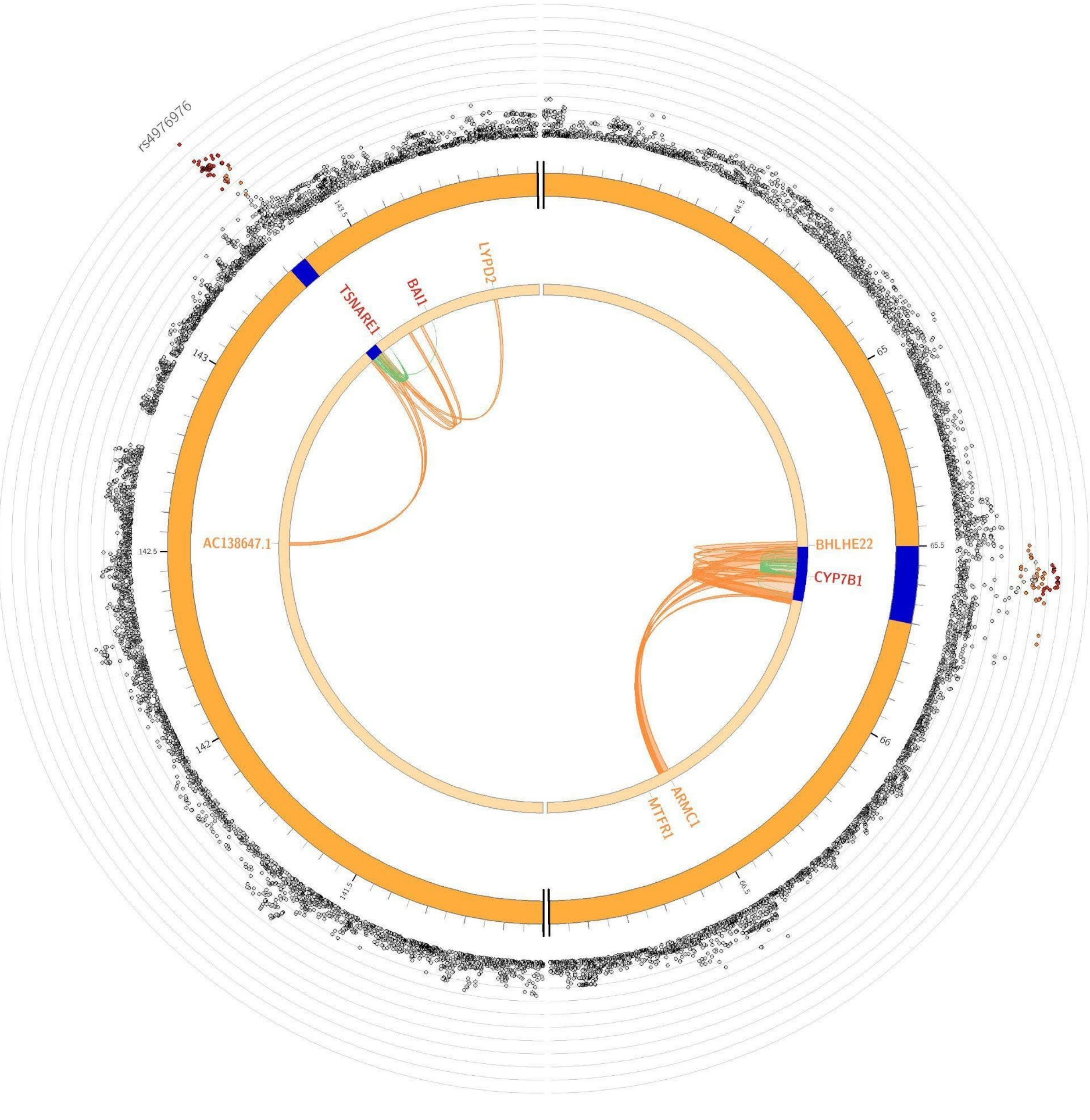

Supplementary Figure 75: Circos plot of chromosome 8

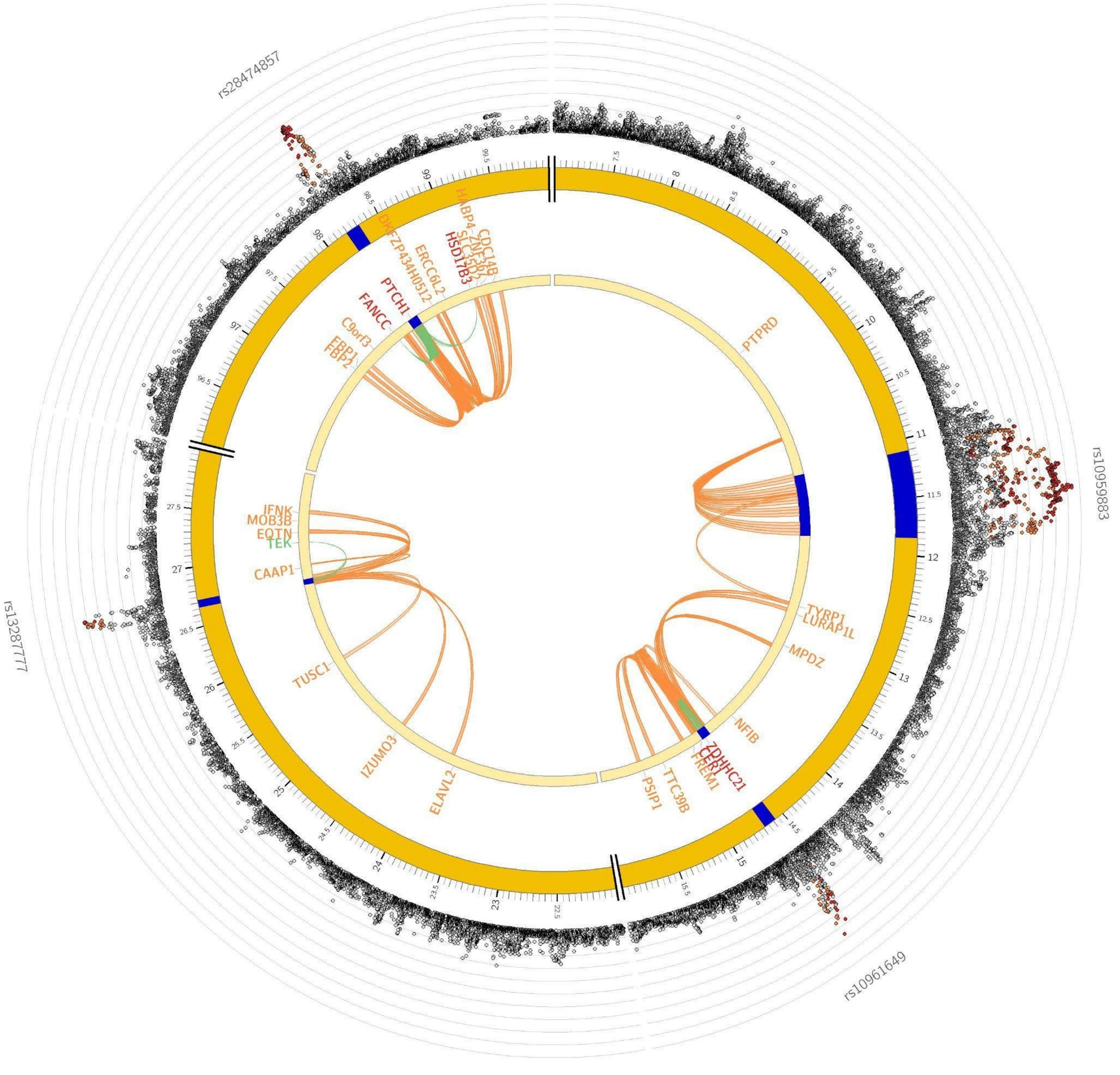

Supplementary Figure 76: Circos plot of chromosome 9

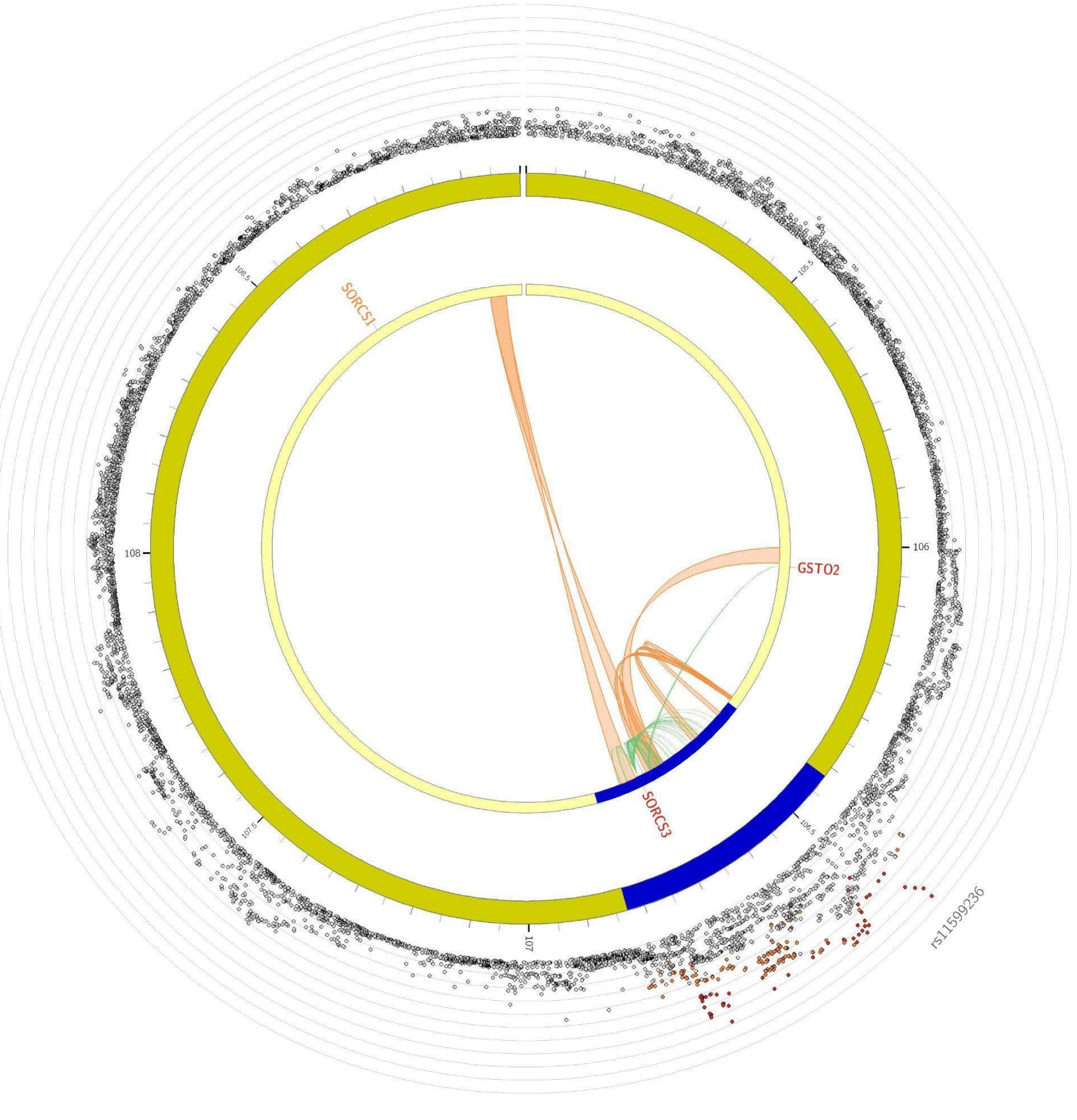

Supplementary Figure 77: Circos plot of chromosome 10

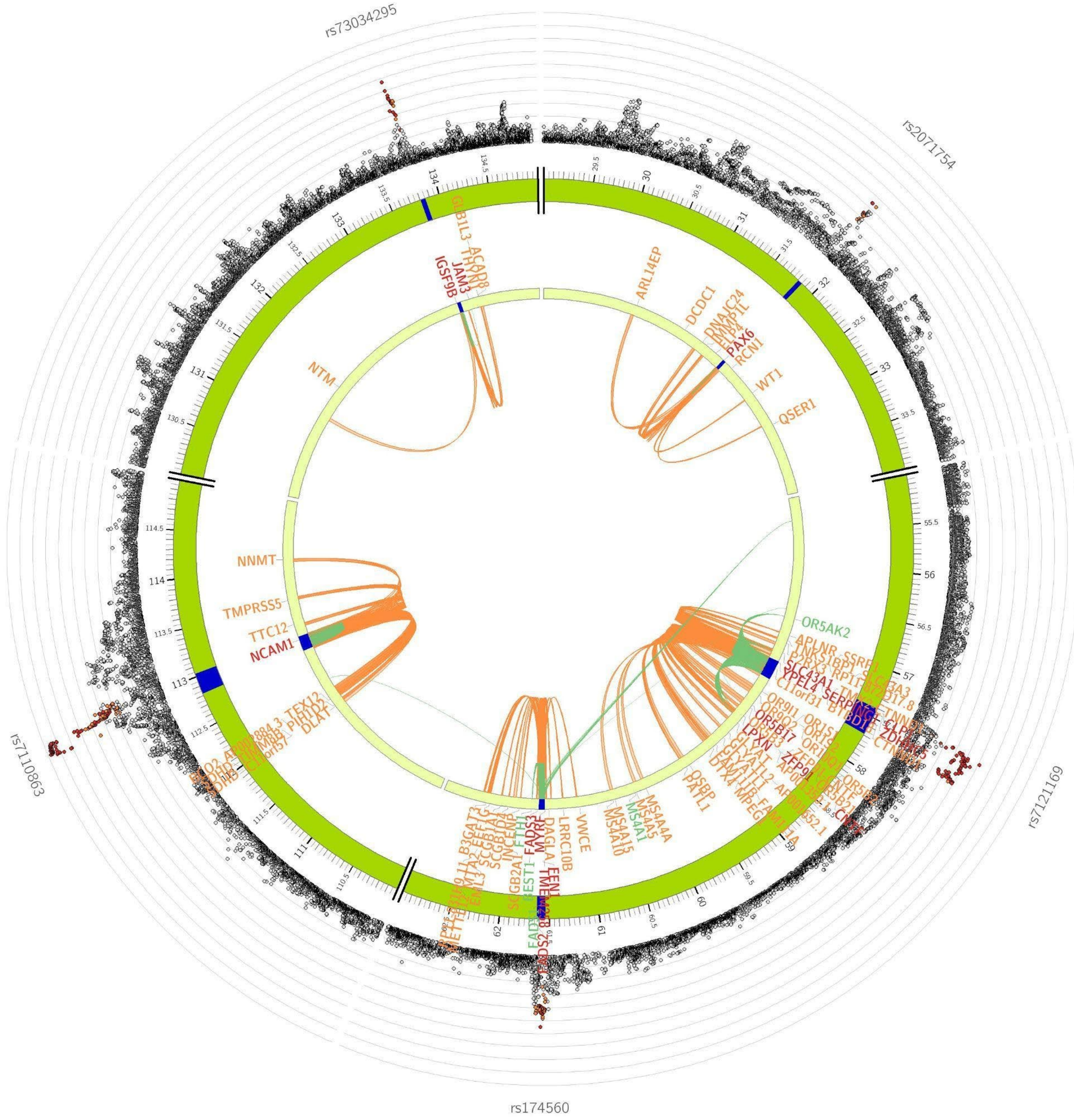

Supplementary Figure 78: Circos plot of chromosome 11

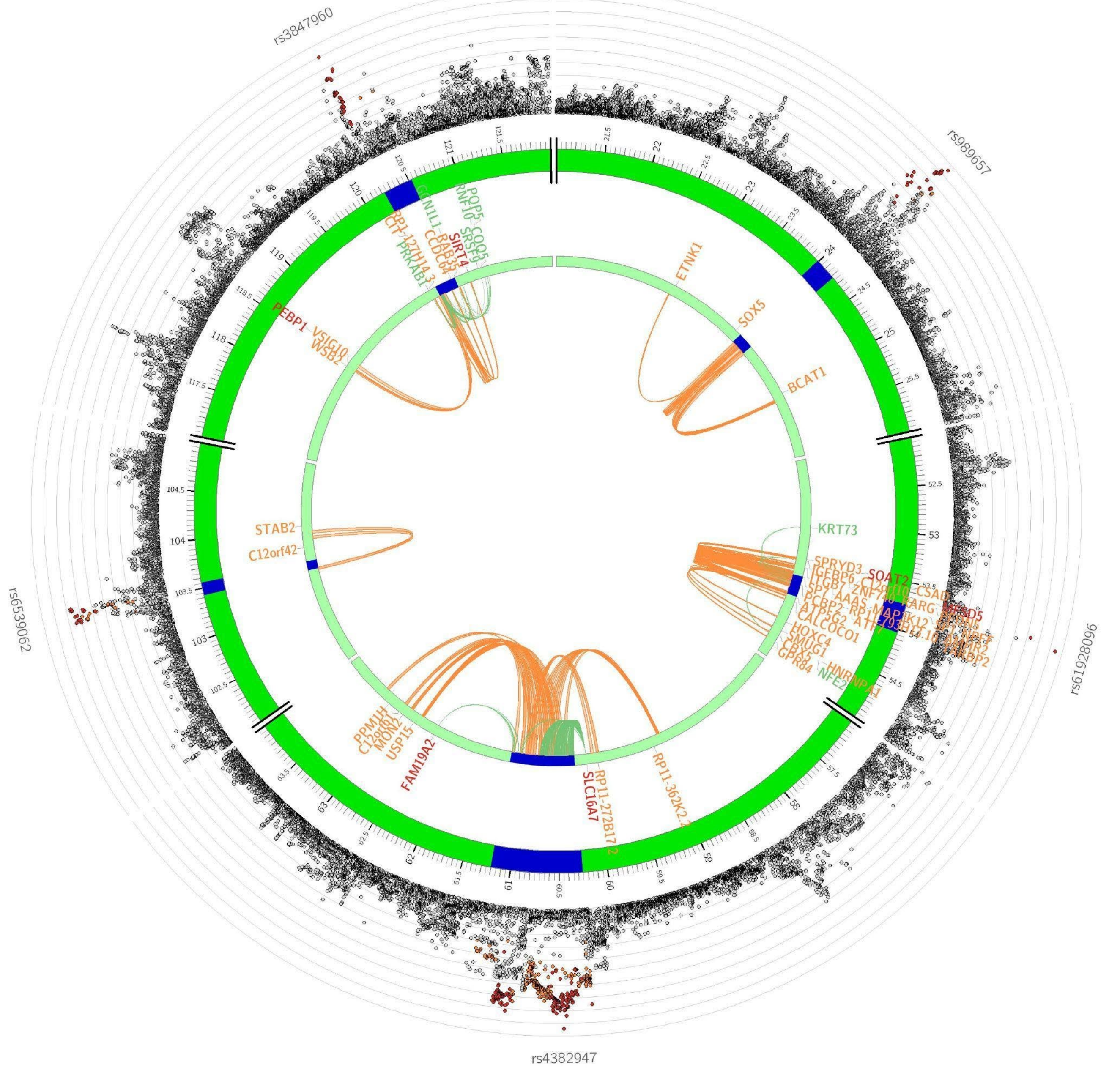

Supplementary Figure 79: Circos plot of chromosome 12

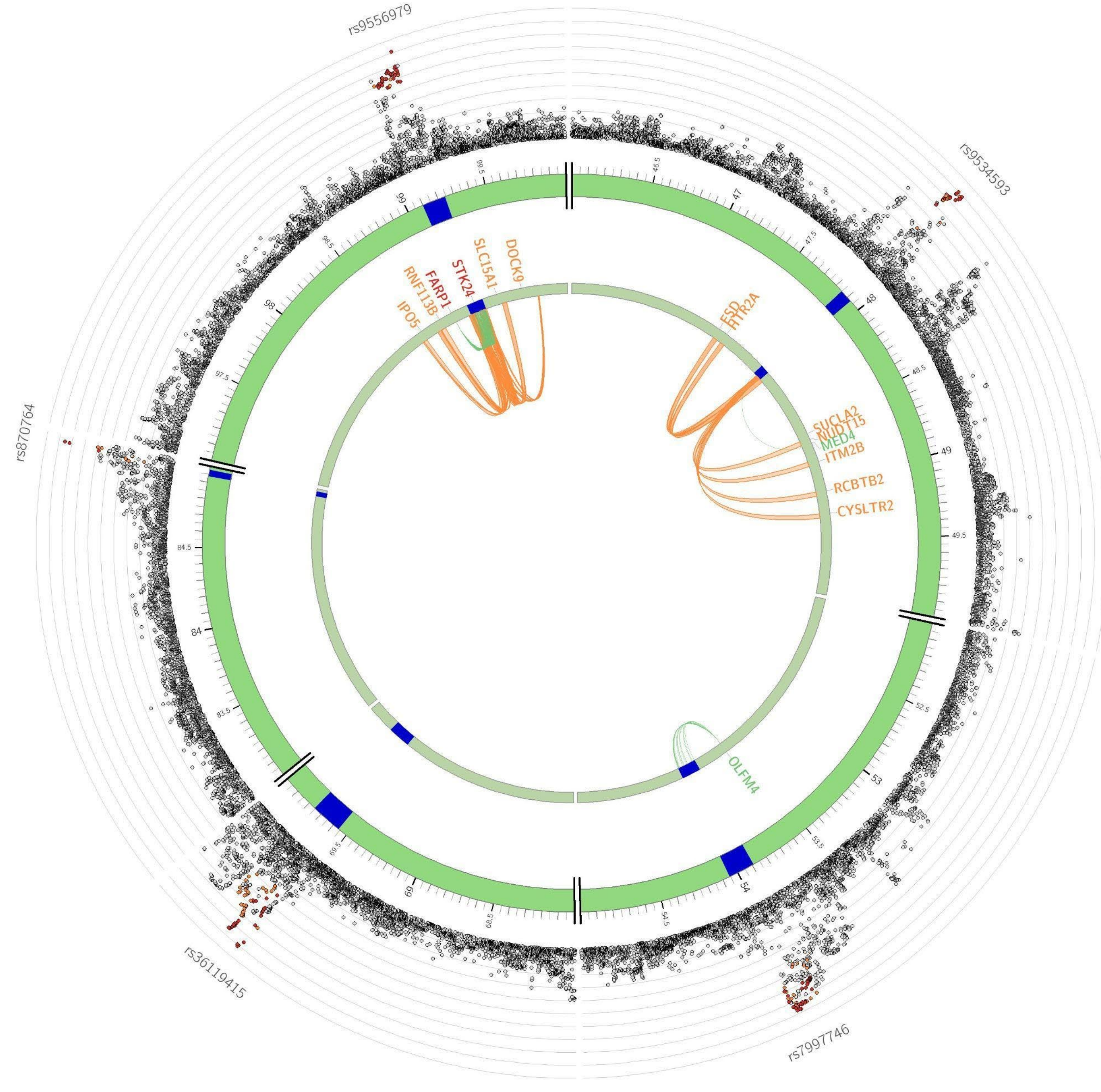

Supplementary Figure 80: Circos plot of chromosome 13

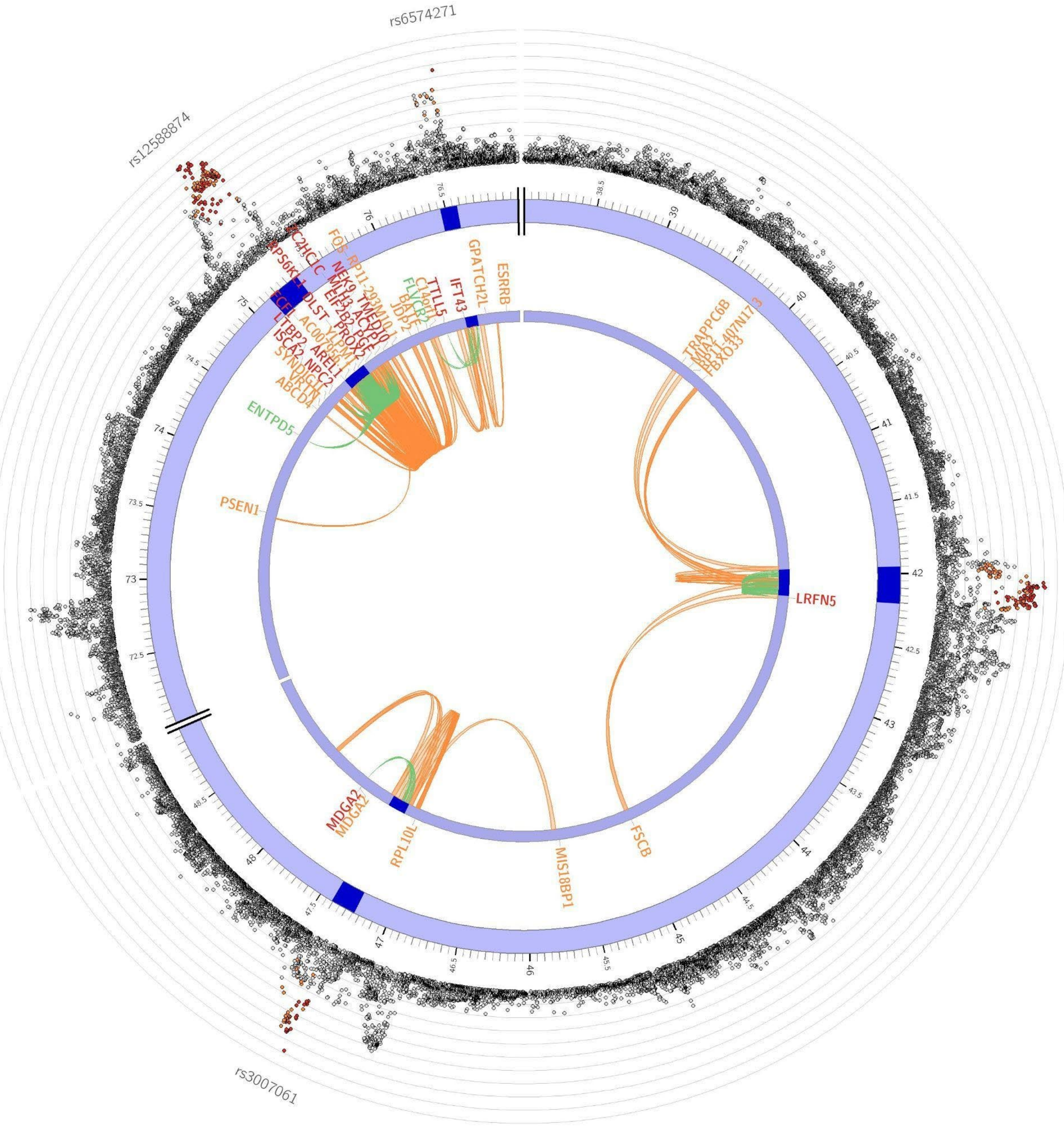

Supplementary Figure 81: Circos plot of chromosome 14

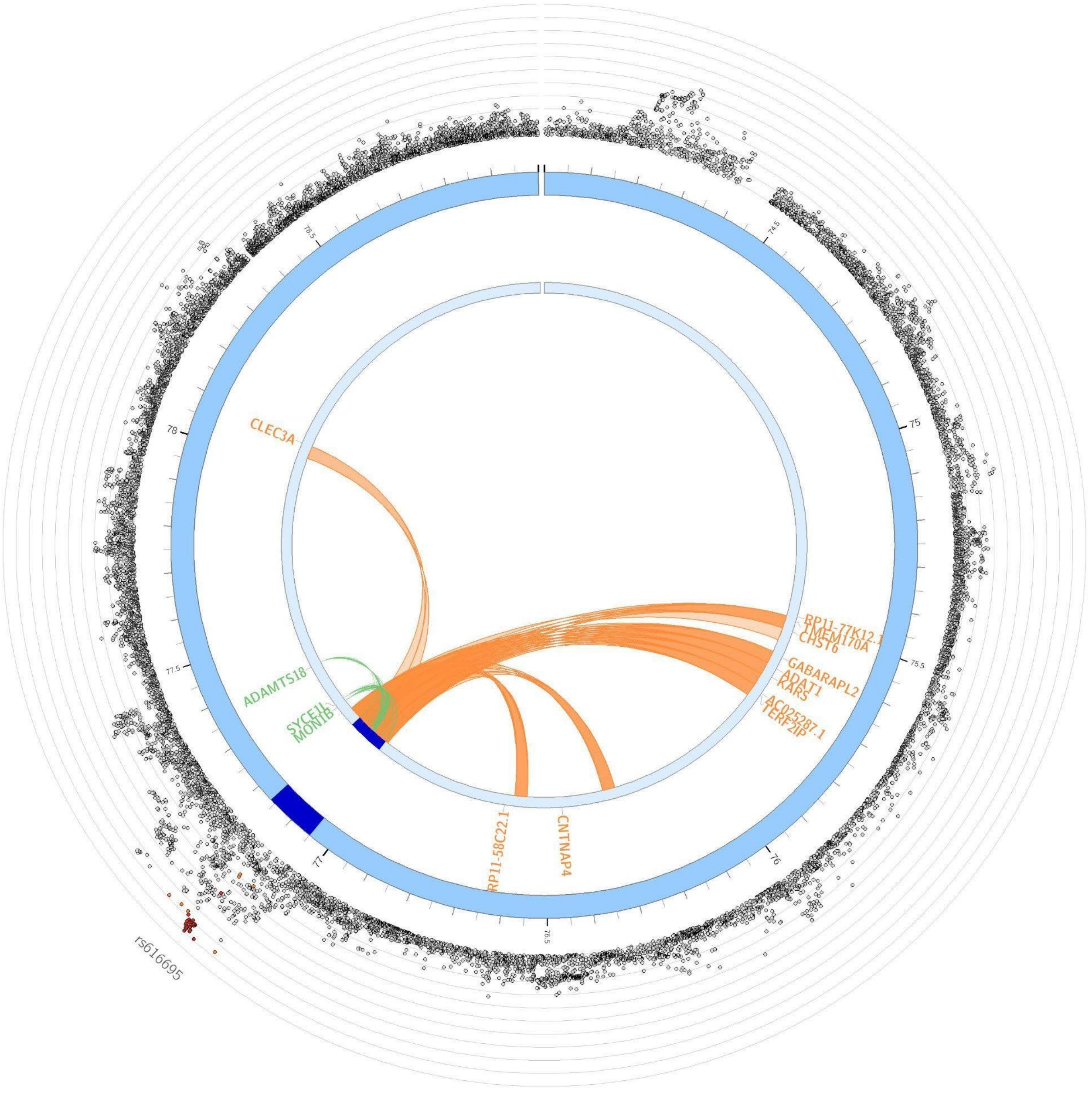

Supplementary Figure 82: Circos plot of chromosome 16

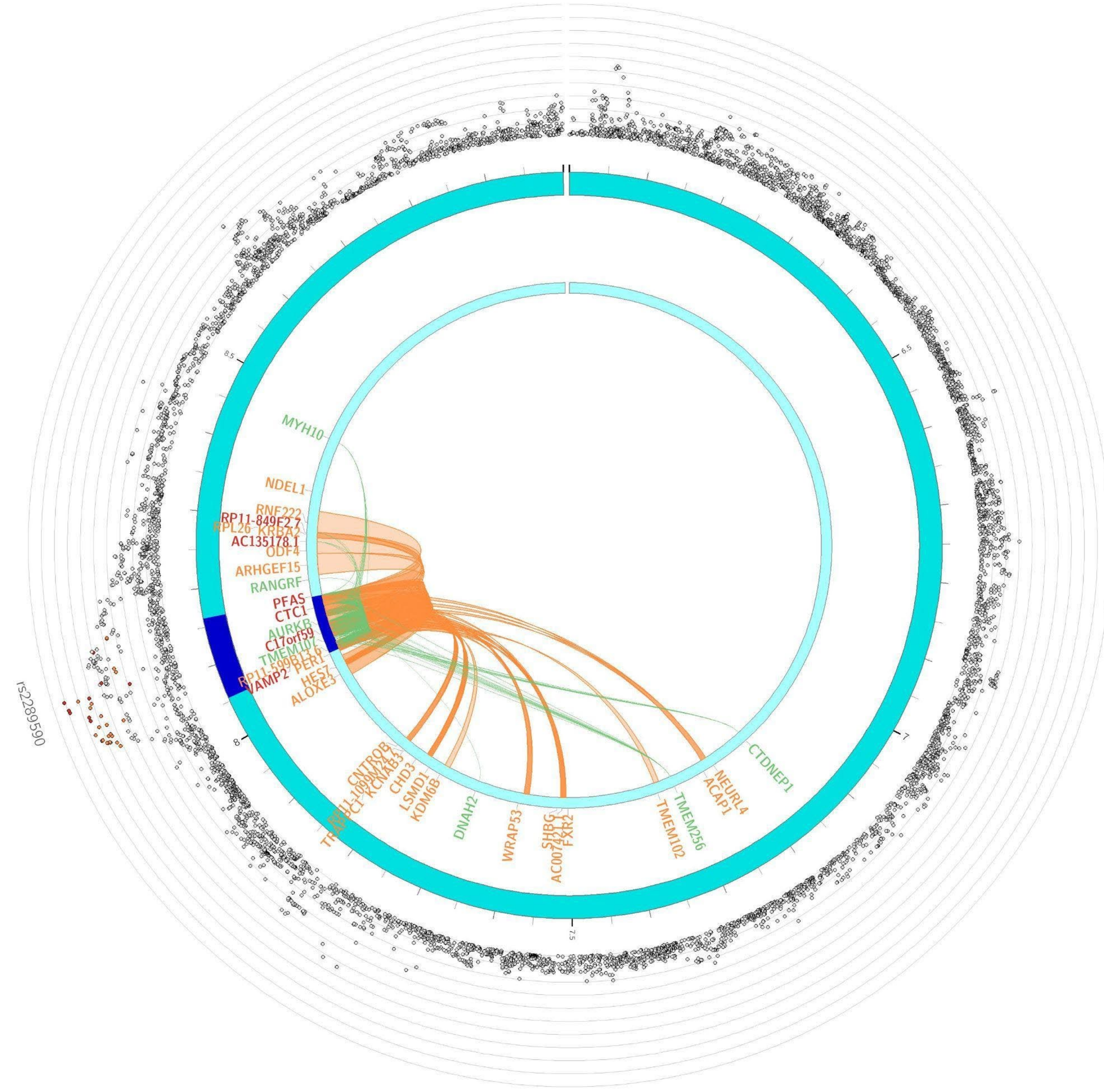

Supplementary Figure 83: Circos plot of chromosome 17

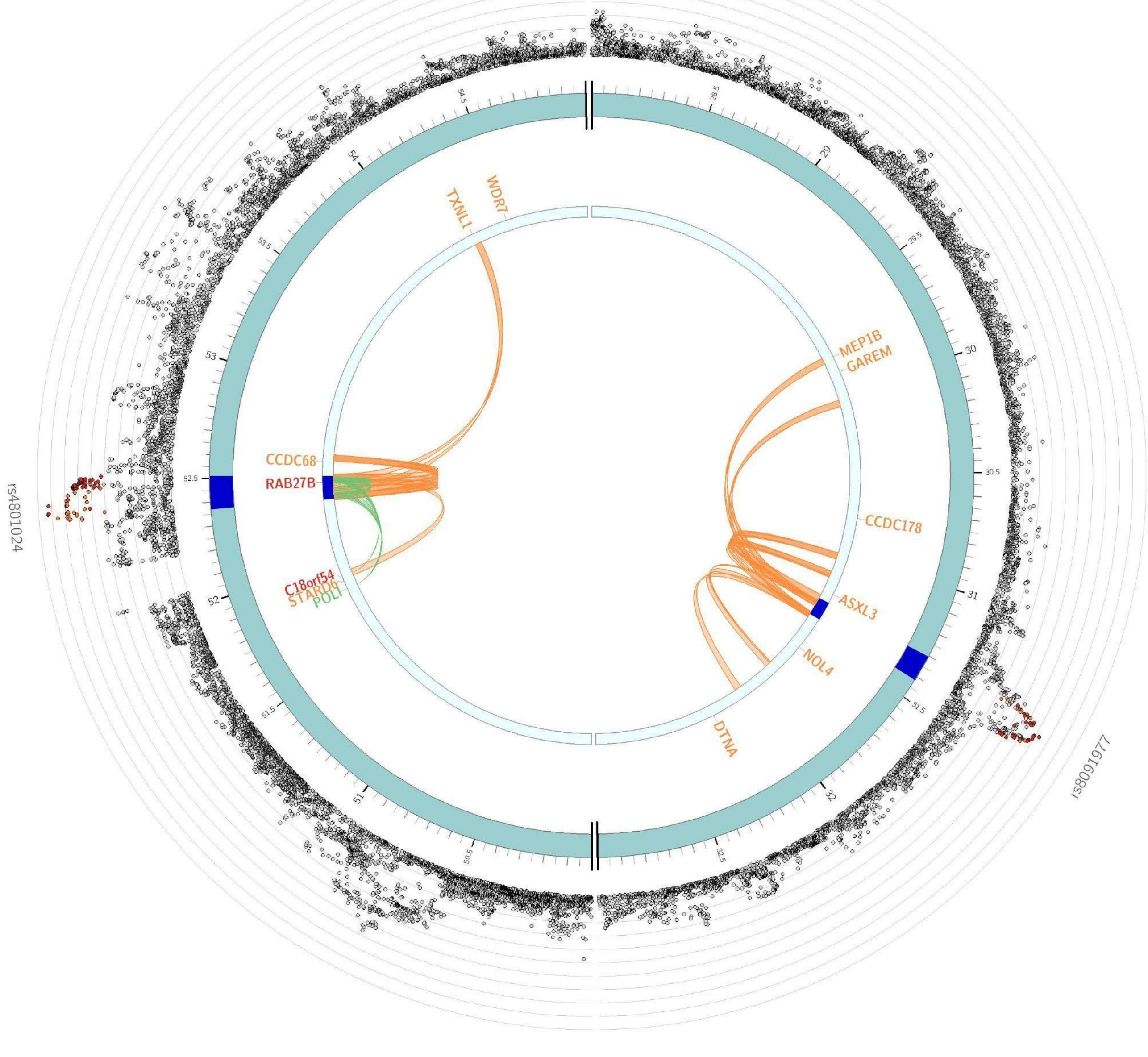

Supplementary Figure 84: Circos plot of chromosome 18

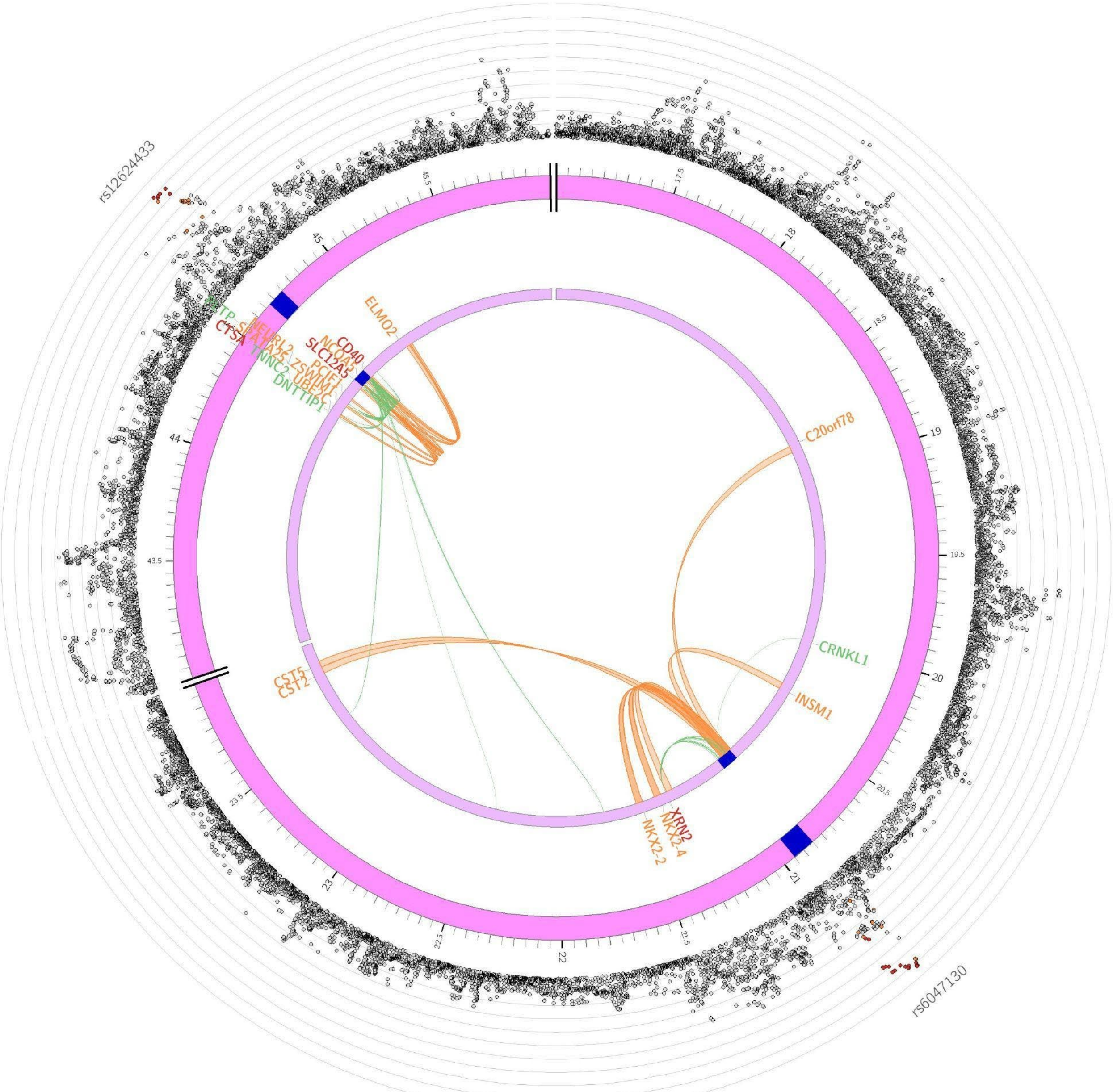

Supplementary Figure 85: Circos plot of chromosome 20

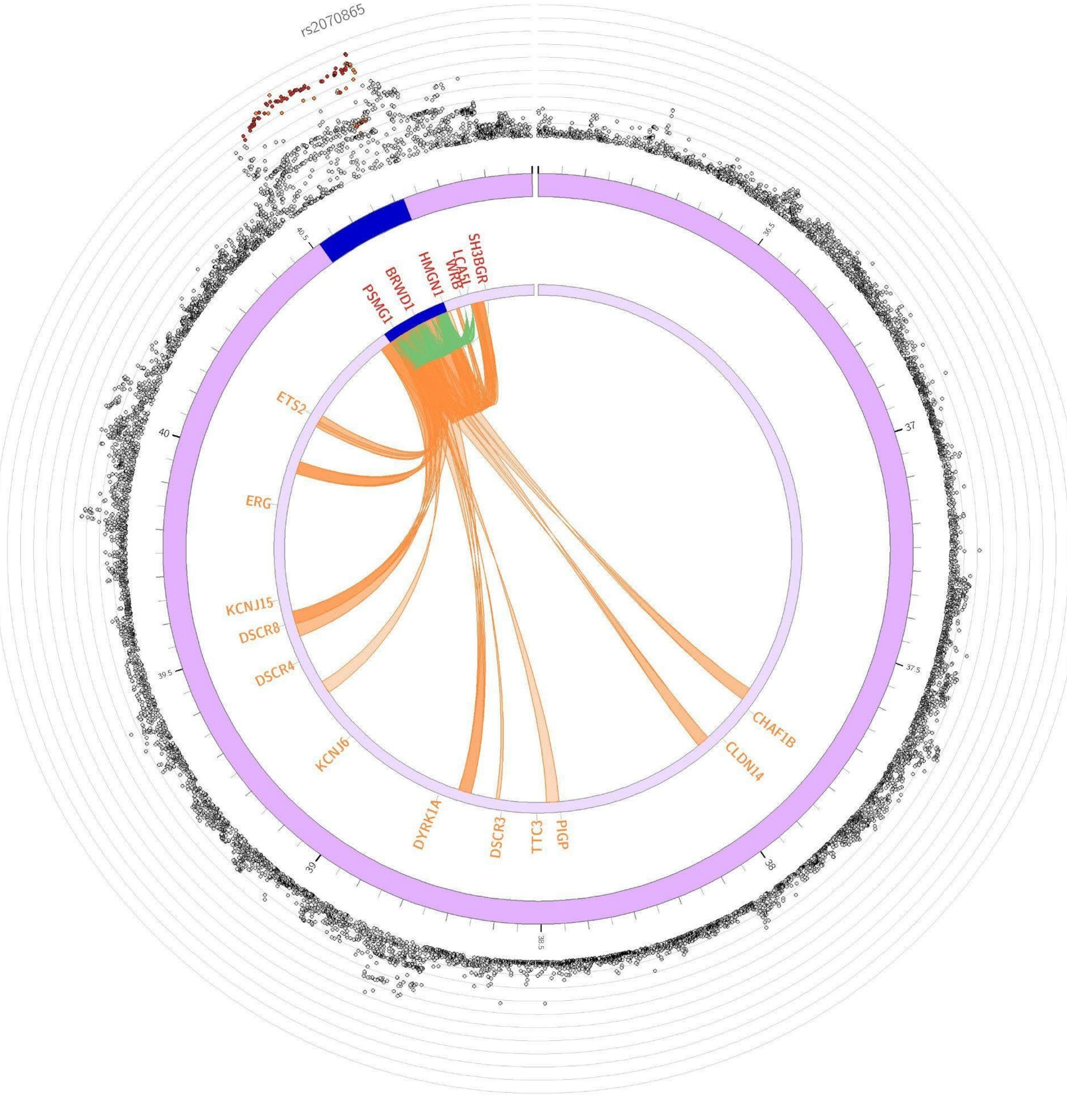

Supplementary Figure 86: Circos plot of chromosome 21

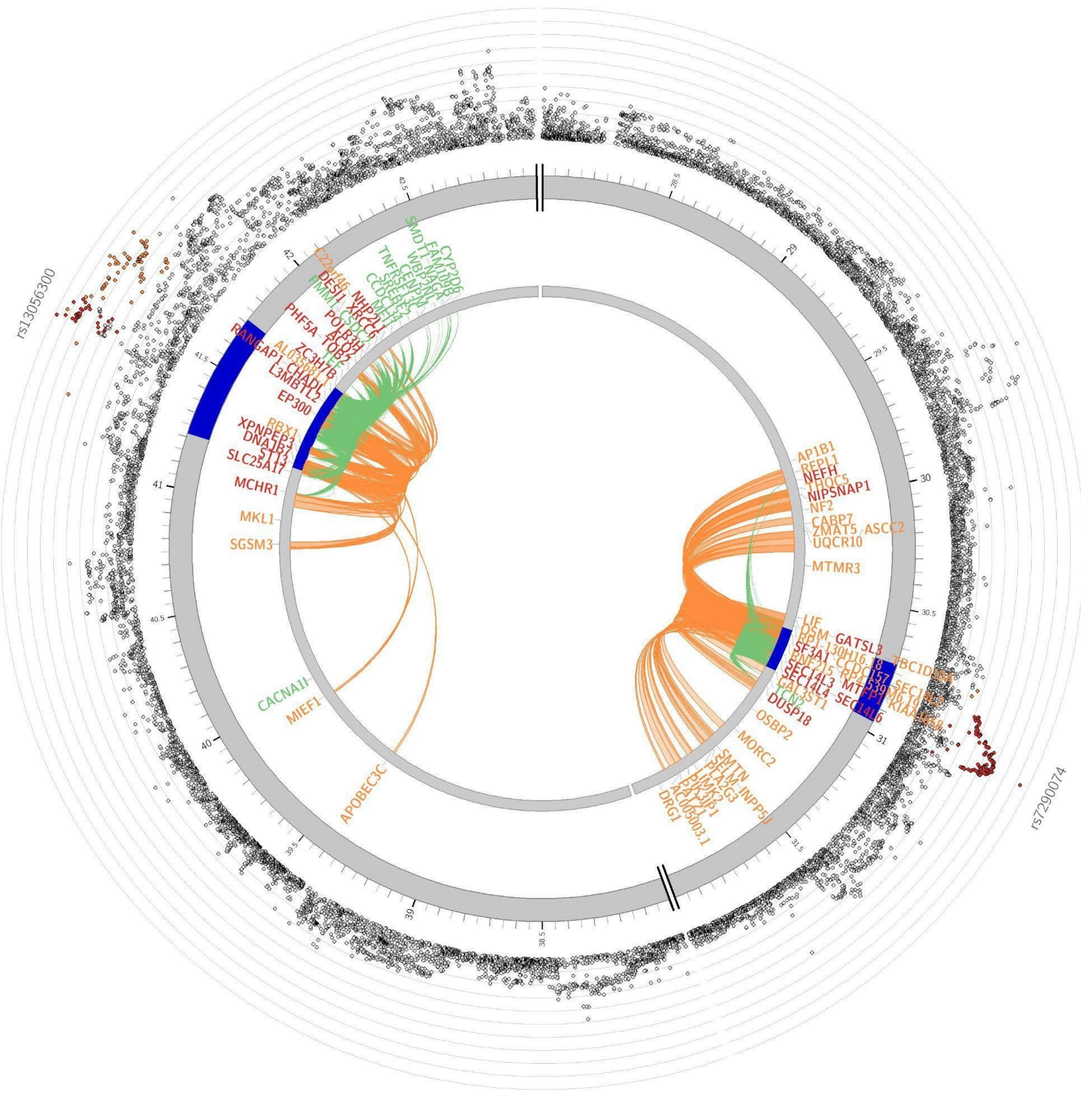

Supplementary Figure 87: Circos plot of chromosome 22

Tissue enrichment and cell-expression of ANX Genes

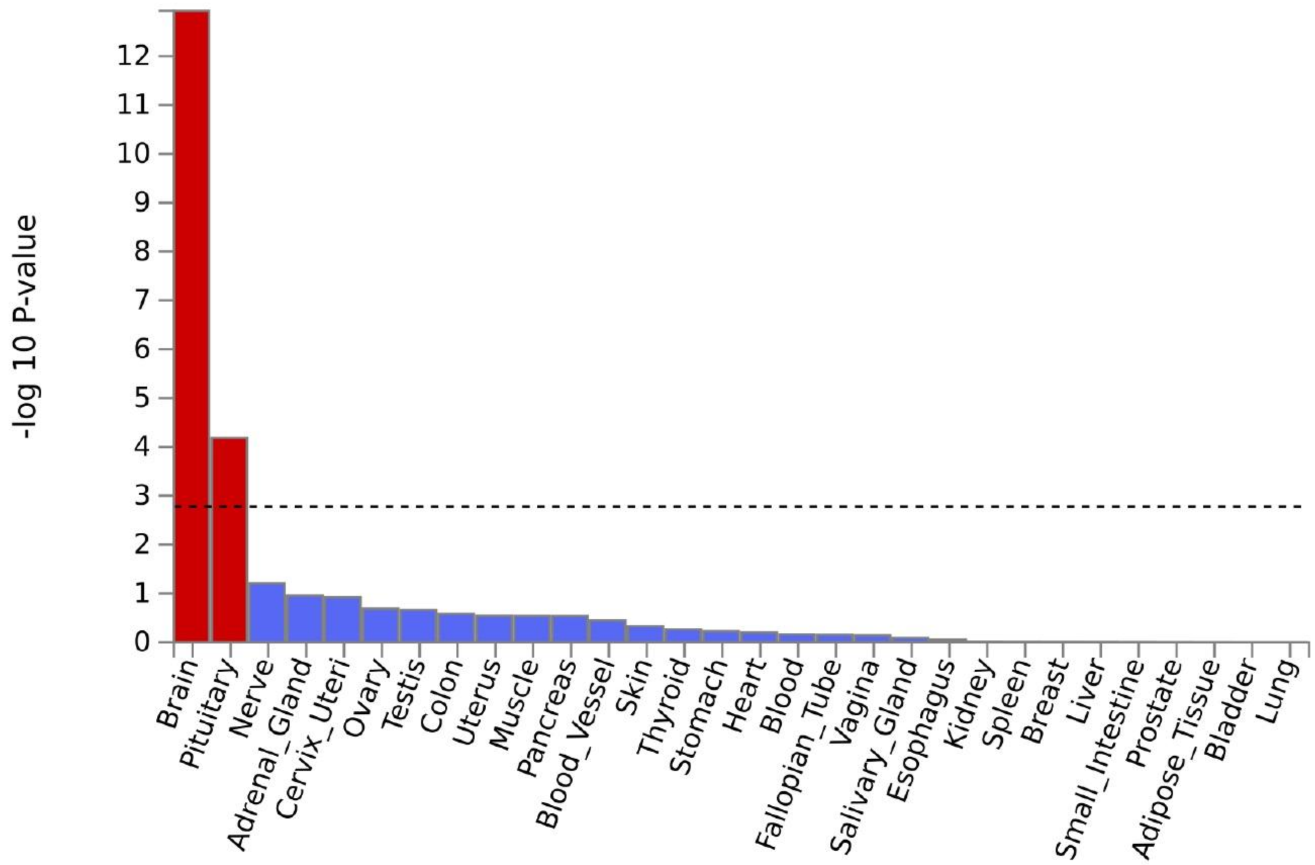

Supplementary Figure 88: MAGMA tissue expression analysis to test tissue enrichment of 30 general tissue types for ANX genes (derived from the main ANX GWAS meta-analsis (Ncases = 122,341, Ncontrols = 729,881)). To test the (positive) relationship between highly expressed genes in a specific tissue and genetic associations, gene-property analysis is performed using average expression of genes per tissue type as a gene covariate. Gene expression values are log2 transformed average RPKM per tissue type after winsorized at 50 based on GTEx RNA-seq data. Tissue expression analysis is performed for 30 general tissue types and 53 specific tissue types separately. MAGMA was performed using the result of gene analysis (gene-based P-value) and tested for one side (greater) with conditioning on average expression across all tissue types. Significant enrichment at Bonferroni corrected P-value 0.05 are coloured in red. Remaining insignificant tissues are cut off to the right for better readability.

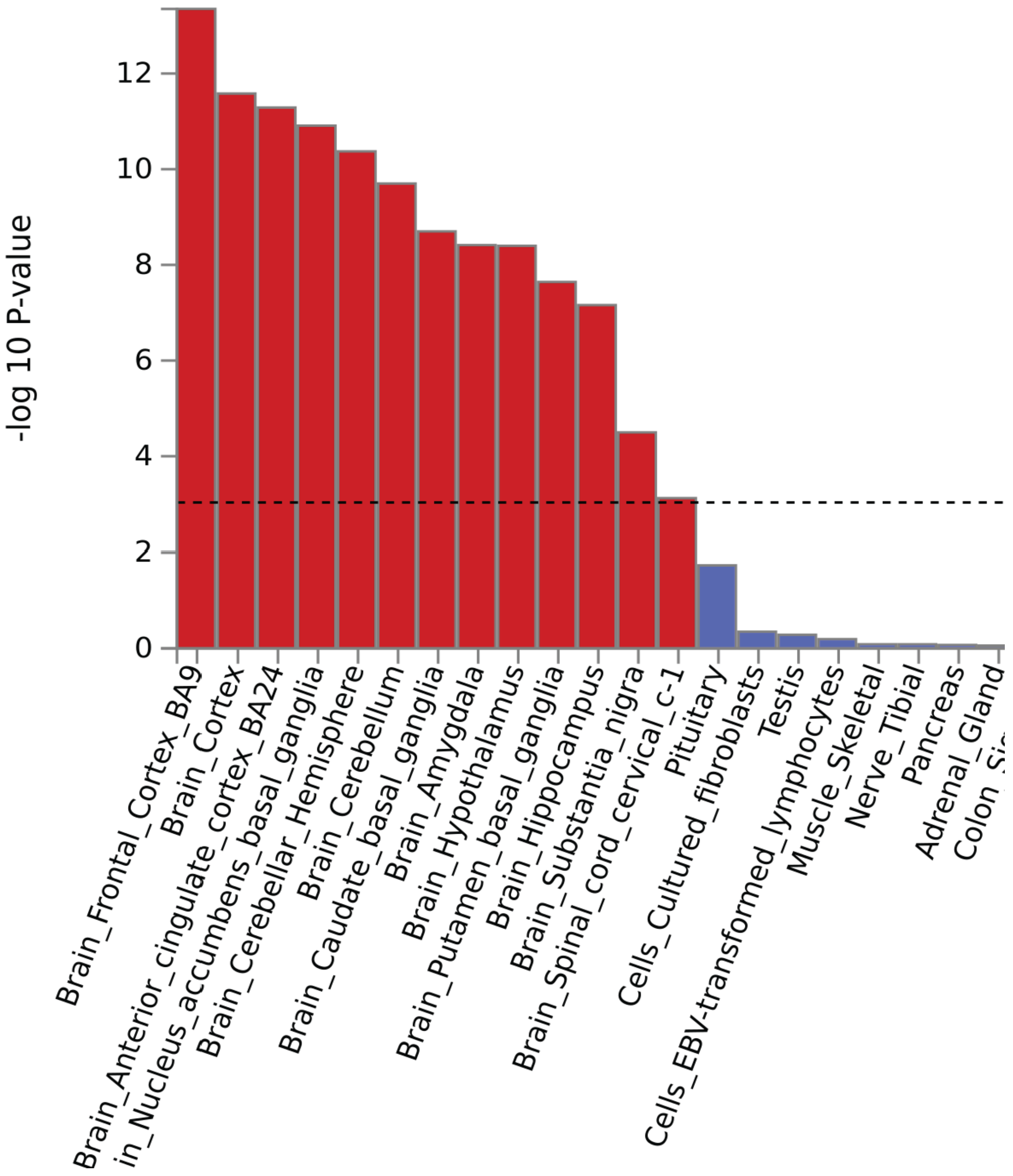

Supplementary Figure 89: MAGMA tissue expression analysis to test tissue enrichment of 53 specific tissue types for ANX genes (derived from the main ANX GWAS meta-analsis (Ncases = 122,341, Ncontrols = 729,881)). To test the (positive) relationship between highly expressed genes in a specific tissue and genetic associations, gene-property analysis is performed using average expression of genes per tissue type as a gene covariate. Gene expression values are log2 transformed average RPKM per tissue type after winsorized at 50 based on GTEx RNA-seq data. Tissue expression analysis is performed for 30 general tissue types and 53 specific tissue types separately. MAGMA was performed using the result of gene analysis (gene-based P-value) and tested for one side (greater) with conditioning on average expression across all tissue types.Significant enrichment at Bonferroni corrected P-value 0.05 are coloured in red. Remaining insignificant tissues are cut off to the right for better readability.

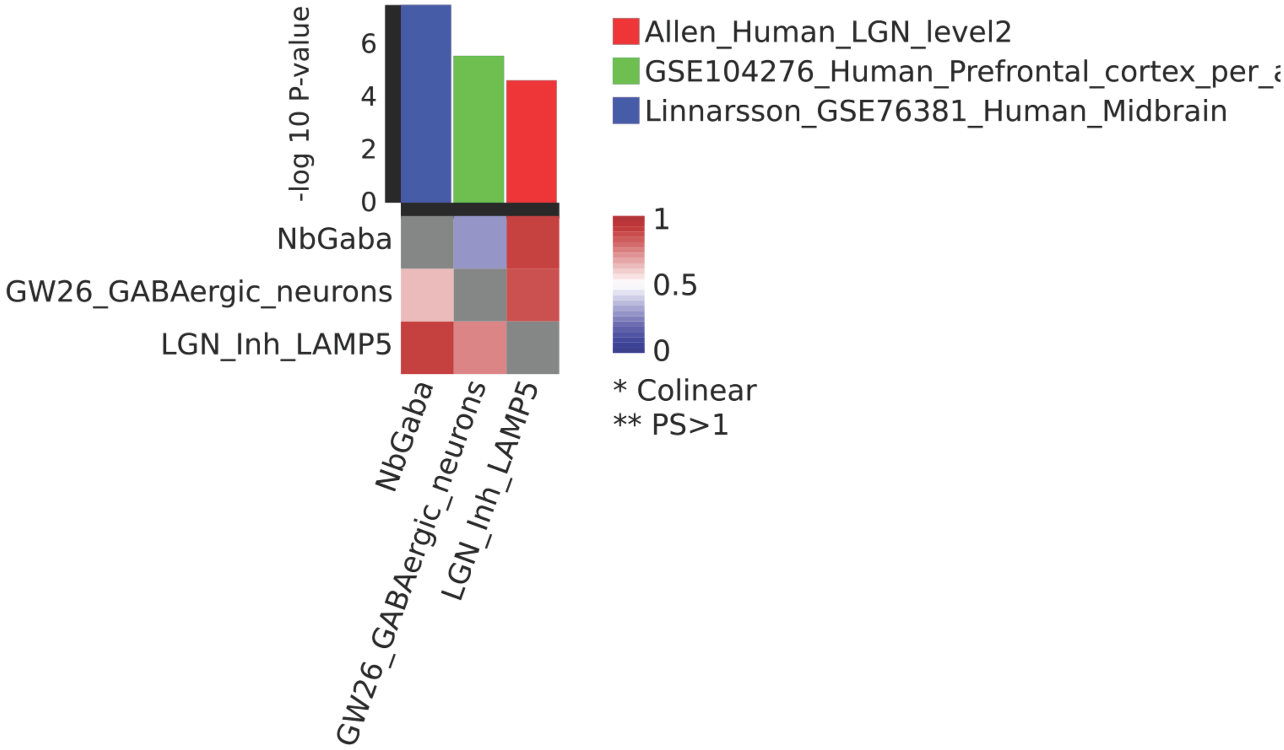

Supplementary Figure 90: **Results of a cross-dataset conditional analysis of the single cell expression association tests**. In brief, all possible cross-dataset pairs of significant cell types retained from the within dataset conditional analysis are analysed in a stepwise conditional analysis per dataset by setting thresholds for proportional significance of the conditional P-value of a cell type relative to the marginal P-value. A full list of all human brain tissue datasets in the analysis and further details are provided in the FUMA tutorial (https://fuma.ctglab.nl/tutorial#celltype). The color in the upper part is based on the original source dataset (**red** for Allen Brain Atlas Human LGN: http://celltypes.brain-map.org/api/v2/well_known_file_download/694416667, **green** for Zhong et al Human cell types: https://www.ncbi.nlm.nih.gov/geo/query/acc.cgi?acc=GSE104276, and **blue** for La Manno et al Human cell types: https://www.ncbi.nlm.nih.gov/geo/query/acc.cgi?acc=GSE76381). Color coding in the lower part is based on value for proportional significance (PS) (a definition of PS can be found at https://fuma.ctglab.nl/tutorial#celltype) with values ranging from 0 (blue) to 1 (red). Interpretation of pairs of PS per dataset comparison can be found at https://fuma.ctglab.nl/tutorial#celltype, PS > 0.8 in both directions suggest an independent association of cell types a and b.

Previous Associations of ANX-SNPs

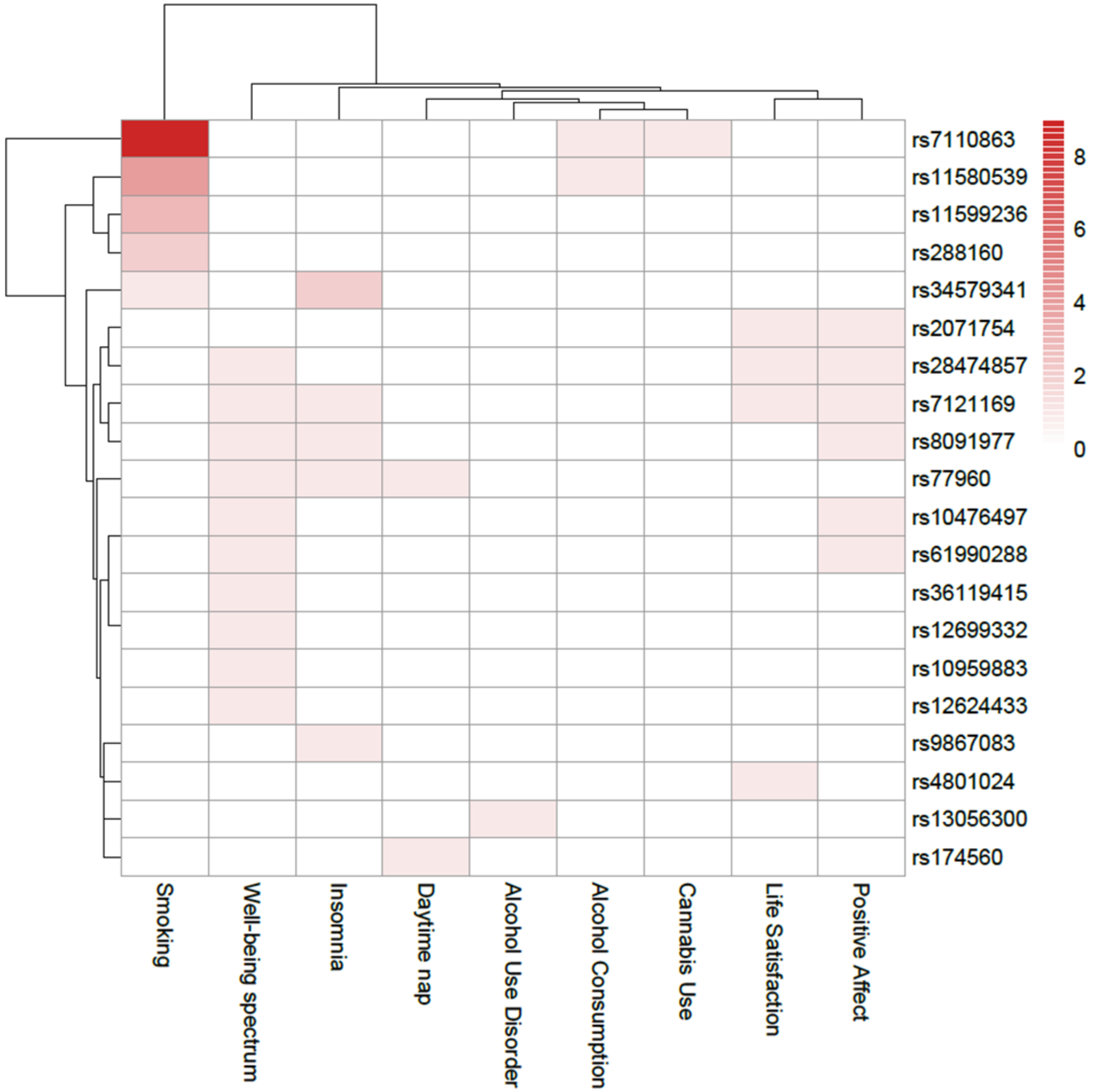

Supplementary Figure 91: Dendrogram-based heatmap indicating the numbers of unduplicated reports of genome-wide behavioural and substance use associations among 20 pleiotropic ANX SNPs. Shading indicates the number of GWAS reporting associations between a specific SNP and the outcomes.

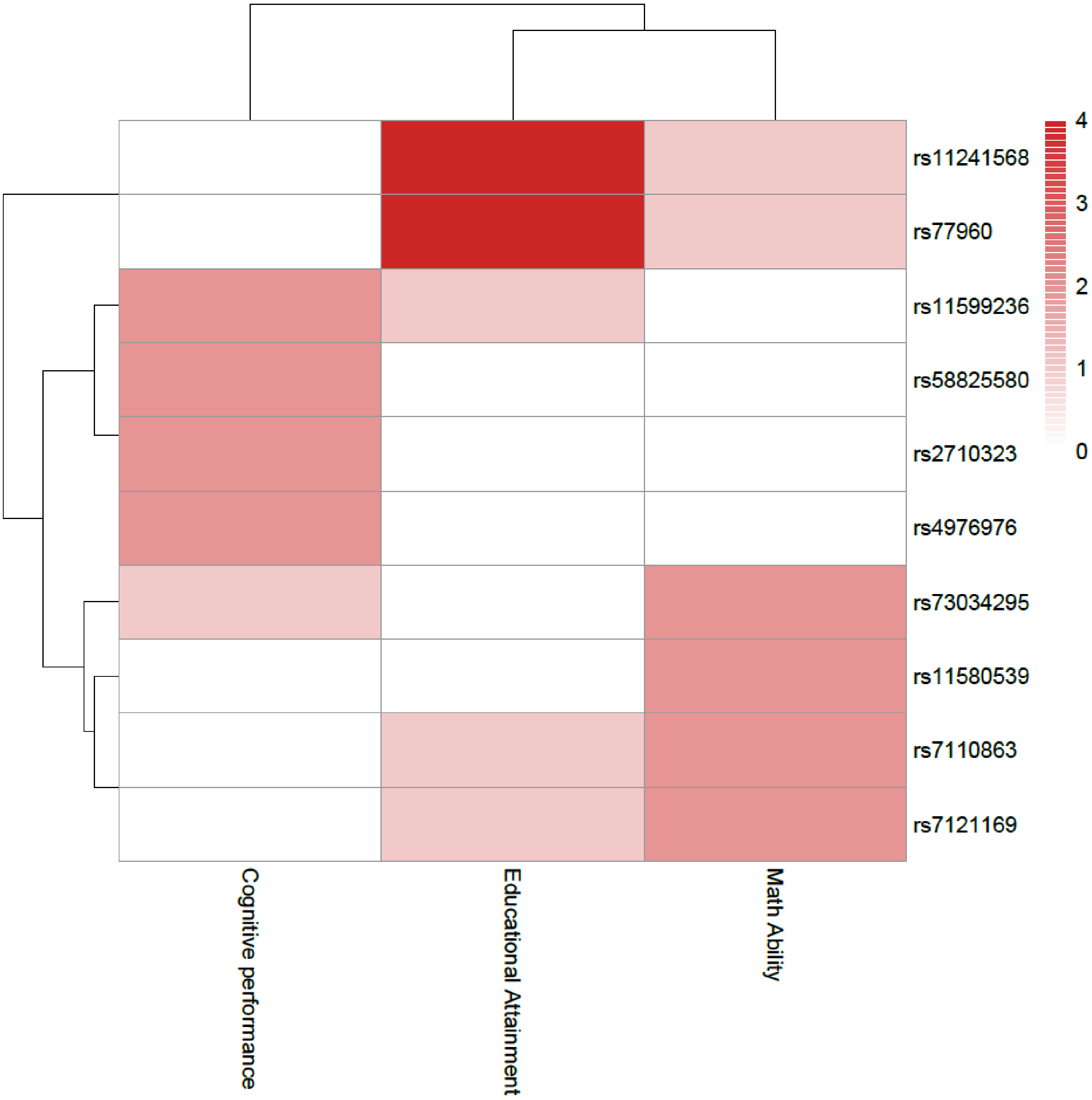

Supplementary Figure 92: Dendrogram-based heatmap indicating the numbers of unduplicated reports of genome-wide associations with three cognitive phenotypes among 10 pleiotropic ANX SNPs. Shading indicates the number of GWAS reporting associations between a specific SNP and the outcomes.

Cross-trait genetic correlations of the ascertainment-specific subgroup analyses

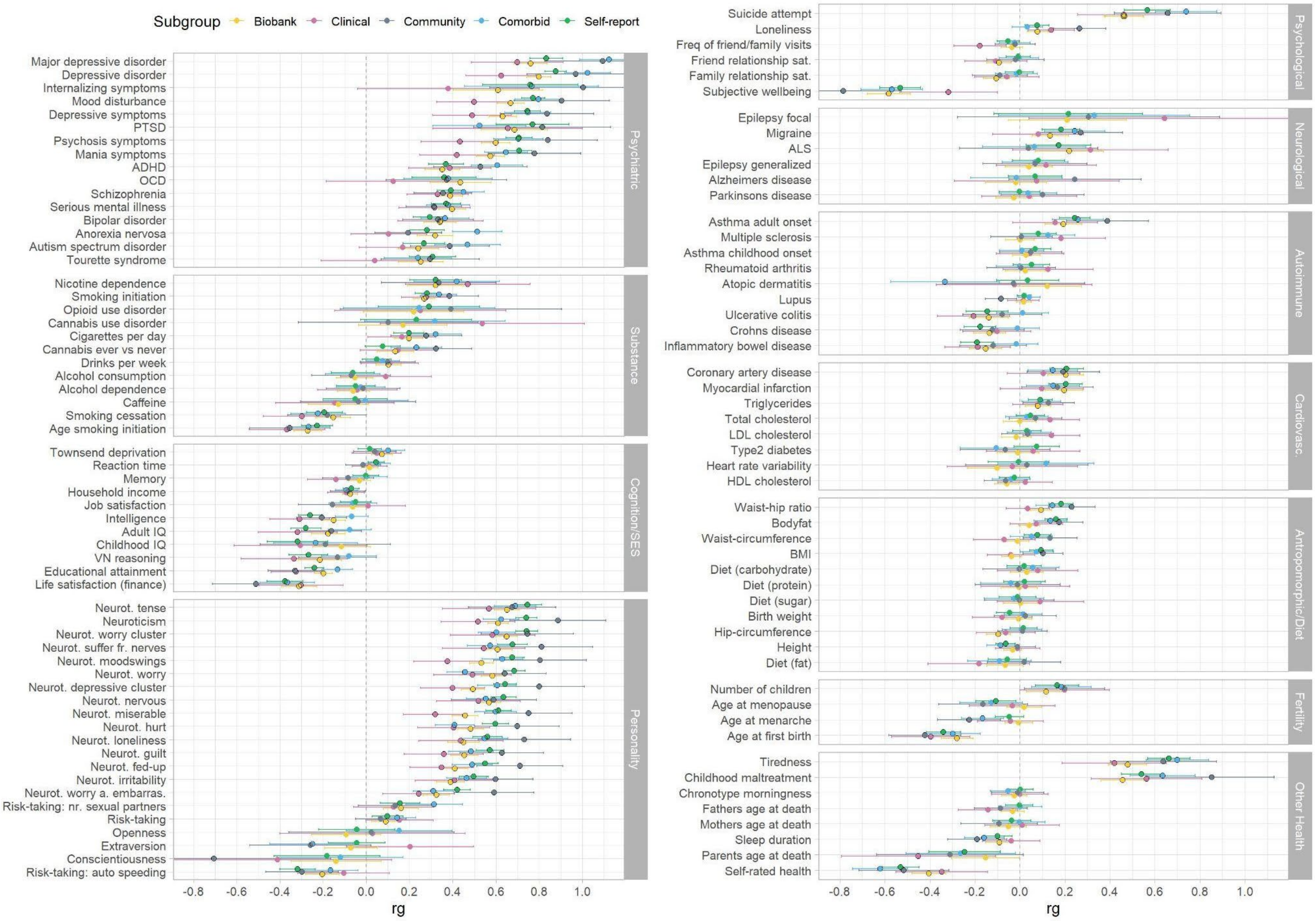

Supplementary Figure 93: Cross-trait genetic correlations between the ANX ascertainment-specific subgroup GWASs (clinical in pink (Ncases = 3,631, Ncontrols = 11,907), biobanks in yellow (Ncases = 37,714, Ncontrols = 420,412), comorbid in blue (Ncases = 12,980, Ncontrols = 27,430), self-reported diagnosis in green (Ncases = 53,978, Ncontrols = 221,844), and community in gray (Ncases = 14,038, Ncontrols = 48,288)) and 112 psychiatric, substance use, cognition/socioeconomic status (SES), personality, psychological, neurological, autoimmune, cardiovascular, anthropomorphic/diet, fertility, and other phenotypes. References and sample sizes of the corresponding summary statistics of the GWAS studies can be found in Supplementary Table 24. Black circles indicate significant associations with a P value adjusted for multiple testing with the Benjamini–Hochberg procedure to control the FDR (<0.05), adjusted separately for each ANX ascertainment-specific subgroup. Error bars represent 95% confidence intervals for the genetic correlation estimates. ADHD, attention-deficit hyperactivity disorder; ALS, amyotrophic lateral sclerosis; BMI, body mass index; embarras., embarrassment; freq, frequency; fr., from; HDL, high-density lipoprotein; LDL, low-density lipoprotein; neurot., neuroticism, nr. number; PTSD post-traumatic stress disorder; sat. satisfaction.

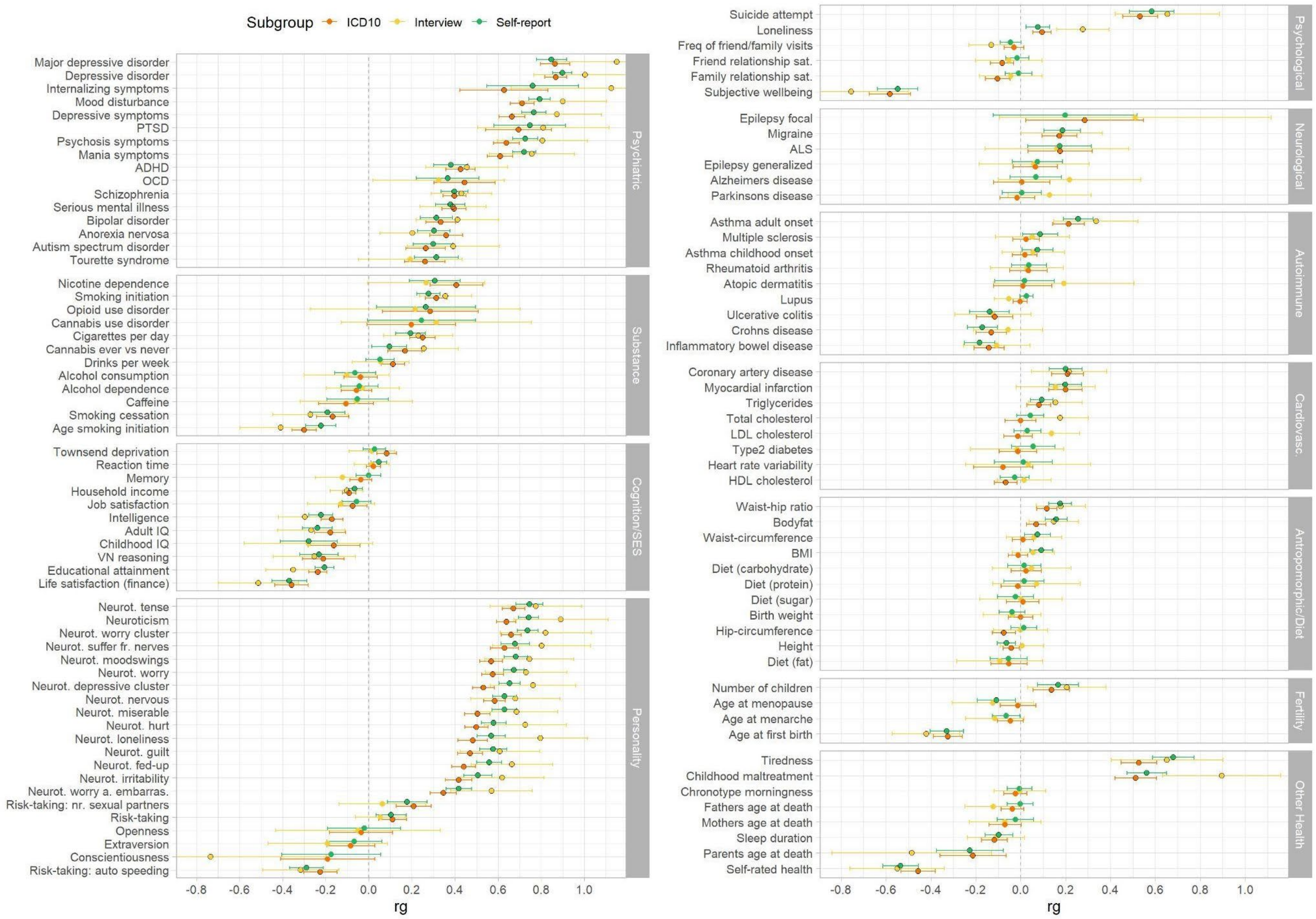

Supplementary Figure 94: Cross-trait genetic correlations between the ANX assessment-specific subgroup GWASs (ICD10 in orange (Ncases = 47,533, Ncontrols = 460,036**)**, Interview-based in yellow (Ncases = 13,396, Ncontrols = 35,272), and self-reported diagnosis in green (Ncases = 61,412, Ncontrols = 234,573) and 112 psychiatric, substance use, cognition/socioeconomic status (SES), personality, psychological, neurological, autoimmune, cardiovascular, anthropomorphic/diet, fertility, and other phenotypes. References and sample sizes of the corresponding summary statistics of the GWAS studies can be found in Supplementary Table 24. Black circles indicate significant associations with a P value adjusted for multiple testing with the Benjamini–Hochberg procedure to control the FDR (<0.05), adjusted separately for each ANX ascertainment-specific subgroup. Error bars represent 95% confidence intervals for the genetic correlation estimates. ADHD, attention-deficit hyperactivity disorder; ALS, amyotrophic lateral sclerosis; BMI, body mass index; embarras., embarrassment; freq, frequency; fr., from; HDL, high-density lipoprotein; LDL, low-density lipoprotein; neurot., neuroticism, nr. number; PTSD post-traumatic stress disorder; sat. satisfaction.

References

1. Boyd A, Golding J, Macleod J, Lawlor DA, Fraser A, Henderson J, *et al.* (2013): Cohort Profile: the 'children of the 90s'--the index offspring of the Avon Longitudinal Study of Parents and Children. *Int J Epidemiol* 42: 111–127.
2. Fraser A, Macdonald-Wallis C, Tilling K, Boyd A, Golding J, Davey Smith G, *et al.* (2013): Cohort Profile: the Avon Longitudinal Study of Parents and Children: ALSPAC mothers cohort. *Int J Epidemiol* 42: 97–110.
3. Northstone K, Lewcock M, Groom A, Boyd A, Macleod J, Timpson N, Wells N (2019): The Avon Longitudinal Study of Parents and Children (ALSPAC): an update on the enrolled sample of index children in 2019. *Wellcome Open Res* 4: 51.
4. Lewis G, Pelosi AJ, Araya R, Dunn G (1992): Measuring psychiatric disorder in the community: a standardized assessment for use by lay interviewers. *Psychol Med* 22: 465–486.
5. Purcell S, Neale B, Todd-Brown K, Thomas L, Ferreira MAR, Bender D, *et al.* (2007): PLINK: a tool set for whole-genome association and population-based linkage analyses. *American journal of human genetics* 81: 559–575.
6. The 1000 Genomes Project Consortium (2015): A global reference for human genetic variation. *Nature* 526: 68–74.
7. Marchini J, Howie B (2010): Genotype imputation for genome-wide association studies [no. 7]. *Nat Rev Genet* 11: 499–511.
8. Otowa T, Hek K, Lee M, Byrne EM, Mirza SS, Nivard MG, *et al.* (2016): Meta-analysis of genome-wide association studies of anxiety disorders. *Mol Psychiatry* 21: 1391–1399.
9. Firmann M, Mayor V, Vidal PM, Bochud M, Pécoud A, Hayoz D, *et al.* (2008): The CoLaus study: a population-based study to investigate the epidemiology and genetic determinants of cardiovascular risk factors and metabolic syndrome. *BMC Cardiovasc Disord* 8: 6.
10. Preisig M, Waeber G, Vollenweider P, Bovet P, Rothen S, Vandeleur C, *et al.* (2009): The PsyCoLaus study: methodology and characteristics of the sample of a population-based survey on psychiatric disorders and their association with genetic and cardiovascular risk factors. *BMC Psychiatry* 9: 9.
11. Preisig M, Fenton BT, Matthey ML, Berney A, Ferrero F (1999): Diagnostic interview for genetic studies (DIGS): inter-rater and test-retest reliability of the French version. *Eur Arch Psychiatry Clin Neurosci* 249: 174–179.
12. Leboyer M, Maier W, Teherani M, Lichtermann D, D'Amato T, Franke P, *et al.* (1991): The reliability of the SADS-LA in a family study setting. *Eur Arch Psychiatry Clin Neurosci* 241: 165–169.
13. Sanders A, Levinson D, Duan J, Dennis J, Li R, Kendler K, *et al.* (2010): The Internet-Based MGS2 Control Sample: Self Report of Mental Illness. *The American journal of psychiatry* 167: 854–65.
14. Gigantesco A, Morosini P (2008): Development, reliability and factor analysis of a self-administered questionnaire which originates from the World Health Organization's Composite International Diagnostic Interview – Short Form (CIDI-SF) for assessing mental disorders. *Clin Pract Epidemiol Ment Health* 4: 8.
15. Shi J, Levinson DF, Duan J, Sanders AR, Zheng Y, Pe'er I, *et al.* (2009): Common variants on chromosome 6p22.1 are associated with schizophrenia. *Nature* 460: 753–757.
16. Howie B, Fuchsberger C, Stephens M, Marchini J, Abecasis GR (2012): Fast and accurate genotype imputation in genome-wide association studies through pre-phasing [no. 8]. *Nat Genet* 44: 955–959.
17. Aulchenko Y, Struchalin M, Duijn C (2010): ProbABEL package for genome-wide association analysis of imputed data. *BMC bioinformatics* 11: 134.

18. Penninx BWJH, Beekman ATF, Smit JH, Zitman FG, Nolen WA, Spinhoven P, *et al.* (2008): The Netherlands Study of Depression and Anxiety (NESDA): rationale, objectives and methods. *Int J Methods Psychiatr Res* 17: 121–140.
19. Willemsen G, Vink JM, Abdellaoui A, den Braber A, van Beek JHDA, Draisma HHM, *et al.* (2013): The Adult Netherlands Twin Register: Twenty-Five Years of Survey and Biological Data Collection. *Twin Res Hum Genet* 16: 271–281.
20. Penninx BWJH, Eikelenboom M, Giltay EJ, van Hemert AM, Riese H, Schoevers RA, Beekman ATF (2021): Cohort profile of the longitudinal Netherlands Study of Depression and Anxiety (NESDA) on etiology, course and consequences of depressive and anxiety disorders. *J Affect Disord* 287: 69–77.
21. Ligthart L, van Beijsterveldt CEM, Kevenaar ST, de Zeeuw E, van Bergen E, Bruins S, *et al.* (2019): The Netherlands Twin Register: Longitudinal Research Based on Twin and Twin-Family Designs. *Twin Res Hum Genet* 22: 623–636.
22. Kessler RC, Ustün TB (2004): The World Mental Health (WMH) Survey Initiative Version of the World Health Organization (WHO) Composite International Diagnostic Interview (CIDI). *Int J Methods Psychiatr Res* 13: 93–121.
23. Spielberger C, Gorsuch R, Lushene R, Vagg P, Jacobs G (1983): Manual for the State-Trait Anxiety Inventory (Form Y1 – Y2). *Palo Alto, CA: Consulting Psychologists Press;*, vol. IV.
24. Li Y, Willer CJ, Ding J, Scheet P, Abecasis GR (2010): MaCH: Using Sequence and Genotype Data to Estimate Haplotypes and Unobserved Genotypes. *Genet Epidemiol* 34: 816–834.
25. Hofman A, Darwish Murad S, van Duijn CM, Franco OH, Goedegebure A, Ikram MA, *et al.* (2013): The Rotterdam Study: 2014 objectives and design update. *Eur J Epidemiol* 28: 889–926.
26. Wittchen HU, Lachner G, Wunderlich U, Pfister H (1998): Test-retest reliability of the computerized DSM-IV version of the Munich-Composite International Diagnostic Interview (M-CIDI). *Soc Psychiatry Psychiatr Epidemiol* 33: 568–578.
27. Wing JK, Babor T, Brugha T, Burke J, Cooper JE, Giel R, *et al.* (1990): SCAN. Schedules for Clinical Assessment in Neuropsychiatry. *Arch Gen Psychiatry* 47: 589–593.
28. Estrada K, Abuseiris A, Grosveld FG, Uitterlinden AG, Knoch TA, Rivadeneira F (2009): GRIMP: a web- and grid-based tool for high-speed analysis of large-scale genome-wide association using imputed data. *Bioinformatics* 25: 2750–2752.
29. Völzke H, Schössow J, Schmidt CO, Jürgens C, Richter A, Werner A, *et al.* (2022): Cohort Profile Update: The Study of Health in Pomerania (SHIP). *Int J Epidemiol* 51: e372–e383.
30. Oldehinkel AJ, Rosmalen JG, Buitelaar JK, Hoek HW, Ormel J, Raven D, *et al.* (2015): Cohort Profile Update: the TRacking Adolescents' Individual Lives Survey (TRAILS). *Int J Epidemiol* 44: 76–76n.
31. de Winter AF, Oldehinkel AJ, Veenstra R, Brunnekreef JA, Verhulst FC, Ormel J (2005): Evaluation of non-response bias in mental health determinants and outcomes in a large sample of pre-adolescents. *Eur J Epidemiol* 20: 173–181.
32. Niarchou M, Gustavson DE, Sathirapongsasuti JF, Anglada-Tort M, Eising E, Bell E, *et al.* (2022): Genome-wide association study of musical beat synchronization demonstrates high polygenicity [no. 9]. *Nat Hum Behav* 6: 1292–1309.
33. Dennis JK, Sealock JM, Straub P, Lee YH, Hucks D, Actkins K, *et al.* (2021): Clinical laboratory test-wide association scan of polygenic scores identifies biomarkers of complex disease. *Genome Medicine* 13: 6.
34. Dennis J, Sealock J, Levinson RT, Farber-Eger E, Franco J, Fong S, *et al.* (2021): Genetic risk for major depressive disorder and loneliness in sex-specific associations with coronary artery disease. *Mol Psychiatry* 26: 4254–4264.
35. Mbatchou J, Barnard L, Backman J, Marcketta A, Kosmicki JA, Ziyatdinov A, *et al.* (2021): Computationally efficient whole-genome regression for quantitative and binary traits [no. 7]. *Nat Genet* 53: 1097–1103.
36. Leitsalu L, Alavere H, Tammesoo ML, Leego E, Metspalu A (2015): Linking a population biobank with national health registries—The Estonian experience. *Journal of Personalized Medicine* 5. https://doi.org/10.3390/jpm5020096

37. Ojalo T, Haan E, Kõiv K, Kariis HM, Krebs K, Uusberg H, *et al.* (2024): Cohort Profile Update: Mental Health Online Survey in the Estonian Biobank (EstBB MHoS). *International Journal of Epidemiology* 53: dyae017.
38. Loh PR, Danecek P, Palamara PF, Fuchsberger C, Reshef YA, Finucane HK, *et al.* (2016): Reference-based phasing using the Haplotype Reference Consortium panel. *Nature Genetics 2016 48:11* 48: 1443–1448.
39. Browning BL, Zhou Y, Browning SR (2018): A One-Penny Imputed Genome from Next-Generation Reference Panels. *Am J Hum Genet* 103: 338–348.
40. Browning SR, Browning BL (2007): Rapid and accurate haplotype phasing and missing-data inference for whole-genome association studies by use of localized haplotype clustering. *American Journal of Human Genetics* 81. https://doi.org/10.1086/521987
41. Mitt M, Kals M, Pärn K, Gabriel SB, Lander ES, Palotie A, *et al.* (2017): Improved imputation accuracy of rare and low-frequency variants using population-specific high-coverage WGS-based imputation reference panel. *European Journal of Human Genetics* 25. https://doi.org/10.1038/ejhg.2017.51
42. Mars N, Koskela JT, Ripatti P, Kiiskinen TTJ, Havulinna AS, Lindbohm JV, *et al.* (2020): Polygenic and clinical risk scores and their impact on age at onset and prediction of cardiometabolic diseases and common cancers. *Nature medicine* 26: 549–557.
43. Costello EJ, Angold A, Burns BJ, Erkanli A, Stangl DK, Tweed DL (1996): The Great Smoky Mountains Study of Youth: Functional Impairment and Serious Emotional Disturbance. *Archives of General Psychiatry* 53: 1137–1143.
44. Angold A, Prendergast M, Cox A, Harrington R, Simonoff E, Rutter M (1995): The Child and Adolescent Psychiatric Assessment (CAPA). *Psychol Med* 25: 739–753.
45. Angold A, Costello EJ (2000): The Child and Adolescent Psychiatric Assessment (CAPA). *J Am Acad Child Adolesc Psychiatry* 39: 39–48.
46. Copeland WE, Angold A, Shanahan L, Costello EJ (2014): Longitudinal patterns of anxiety from childhood to adulthood: the Great Smoky Mountains Study. *J Am Acad Child Adolesc Psychiatry* 53: 21–33.
47. Simonoff E, Pickles A, Meyer JM, Silberg JL, Maes HH, Loeber R, *et al.* (1997): The Virginia Twin Study of Adolescent Behavioral Development. Influences of age, sex, and impairment on rates of disorder. *Arch Gen Psychiatry* 54: 801–808.
48. Hewitt JK, Silberg JL, Rutter M, Simonoff E, Meyer JM, Maes H, *et al.* (1997): Genetics and developmental psychopathology: 1. Phenotypic assessment in the Virginia Twin Study of Adolescent Behavioral Development. *Child Psychology & Psychiatry & Allied Disciplines* 38: 943–963.
49. Eaves LJ, Silberg JL, Meyer JM, Maes HH, Simonoff E, Pickles A, *et al.* (1997): Genetics and developmental psychopathology: 2. The main effects of genes and environment on behavioral problems in the Virginia Twin Study of Adolescent Behavioral Development. *J Child Psychol Psychiatry* 38: 965–980.
50. Maes HH, Silberg JL, Neale MC, Eaves LJ (2007): Genetic and Cultural Transmission of Antisocial Behavior: An Extended Twin Parent Model. *Twin Research and Human Genetics* 10: 136–150.
51. Fergusson DM, Horwood LJ (2001): The Christchurch Health and Development Study: review of findings on child and adolescent mental health. *Aust N Z J Psychiatry* 35: 287–296.
52. Costello EJ, Eaves L, Sullivan P, Kennedy M, Conway K, Adkins DE, *et al.* (2013): Genes, environments, and developmental research: methods for a multi-site study of early substance abuse. *Twin Res Hum Genet* 16: 505–515.
53. Adkins DE, Clark SL, Copeland WE, Kennedy M, Conway K, Angold A, *et al.* (2015): Genome-wide meta-analysis of longitudinal alcohol consumption across youth and early adulthood. *Twin Res Hum Genet* 18: 335–347.
54. Kerr SM, Campbell A, Marten J, Vitart V, McIntosh AM, Porteous DJ, Hayward C (2017): Electronic health record and genome-wide genetic data in Generation Scotland participants. *Wellcome Open Res* 2: 85.
55. Smith BH, Campbell H, Blackwood D, Connell J, Connor M, Deary IJ, *et al.* (2006): Generation Scotland: the Scottish Family Health Study; a new resource for researching genes and heritability. *BMC Med Genet* 7: 74.

56. Smith BH, Campbell A, Linksted P, Fitzpatrick B, Jackson C, Kerr SM, *et al.* (2013): Cohort Profile: Generation Scotland: Scottish Family Health Study (GS:SFHS). The study, its participants and their potential for genetic research on health and illness. *Int J Epidemiol* 42: 689–700.
57. Hall LS, Adams MJ, Arnau-Soler A, Clarke T-K, Howard DM, Zeng Y, *et al.* (2018): Genome-wide meta-analyses of stratified depression in Generation Scotland and UK Biobank [no. 1]. *Transl Psychiatry* 8: 1–12.
58. Nagy R, Boutin TS, Marten J, Huffman JE, Kerr SM, Campbell A, *et al.* (2017): Exploration of haplotype research consortium imputation for genome-wide association studies in 20,032 Generation Scotland participants. *Genome Med* 9: 23.
59. Holmen TL, Bratberg G, Krokstad S, Langhammer A, Hveem K, Midthjell K, *et al.* (2014): Cohort profile of the Young-HUNT Study, Norway: a population-based study of adolescents. *Int J Epidemiol* 43: 536–544.
60. Krokstad S, Langhammer A, Hveem K, Holmen TL, Midthjell K, Stene TR, *et al.* (2013): Cohort profile: The HUNT study, Norway. *International Journal of Epidemiology*. https://doi.org/10.1093/ije/dys095
61. Brage S, Bentsen BG, Bjerkedal T, Nygård JF, Tellnes G (1996): ICPC as a standard classification in Norway. *Fam Pract* 13: 391–396.
62. Kessler RC, Berglund P, Demler O, Jin R, Merikangas KR, Walters EE (2005): Lifetime prevalence and age-of-onset distributions of DSM-IV disorders in the National Comorbidity Survey Replication. *Arch Gen Psychiatry* 62: 593–602.
63. Wang PS, Berglund P, Olfson M, Pincus HA, Wells KB, Kessler RC (2005): Failure and delay in initial treatment contact after first onset of mental disorders in the National Comorbidity Survey Replication. *Arch Gen Psychiatry* 62: 603–613.
64. Bjelland I, Dahl AA, Haug TT, Neckelmann D (2002): The validity of the Hospital Anxiety and Depression Scale. An updated literature review. *J Psychosom Res* 52: 69–77.
65. Guo Y, He J, Zhao S, Wu H, Zhong X, Sheng Q, *et al.* (2014): Illumina human exome genotyping array clustering and quality control. *Nature Protocols* 9: 2643–2662.
66. Kent WJ (2002): BLAT--the BLAST-like alignment tool. *Genome Res* 12: 656–664.
67. Chang CC, Chow CC, Tellier LCAM, Vattikuti S, Purcell SM, Lee JJ (2015): Second-generation PLINK: Rising to the challenge of larger and richer datasets. *GigaScience* 4: 7.
68. Wang C, Zhan X, Bragg-Gresham J, Kang HM, Stambolian D, Chew EY, *et al.* (2014): Ancestry estimation and control of population stratification for sequence-based association studies. *Nature Genetics* 46: 409–415.
69. Li JZ, Absher DM, Tang H, Southwick AM, Casto AM, Ramachandran S, *et al.* (2008): Worldwide human relationships inferred from genome-wide patterns of variation. *Science* 319: 1100–1104.
70. Das S, Forer L, Schönherr S, Sidore C, Locke AE, Kwong A, *et al.* (2016): Next-generation genotype imputation service and methods. *Nature Genetics* 48: 1284–1287.
71. Pedersen CB, Bybjerg-Grauholm J, Pedersen MG, Grove J, Agerbo E, Bækvad-Hansen M, *et al.* (2018): The iPSYCH2012 case-cohort sample: New directions for unravelling genetic and environmental architectures of severe mental disorders. *Mol Psychiatry* 23: 6–14.
72. Bybjerg-Grauholm J, Pedersen CB, Bækvad-Hansen M, Pedersen MG, Adamsen D, Hansen CS, *et al.* (2020, December 2): The iPSYCH2015 Case-Cohort sample: updated directions for unravelling genetic and environmental architectures of severe mental disorders. medRxiv, p 2020.11.30.20237768.
73. Meier, Trontti K, Purves KL, Als TD, Grove J, Laine M, *et al.* (2019): Genetic Variants Associated With Anxiety and Stress-Related Disorders: A Genome-Wide Association Study and Mouse-Model Study. *JAMA Psychiatry* 76: 924–932.
74. Lam M, Awasthi S, Watson HJ, Goldstein J, Panagiotaropoulou G, Trubetskoy V, *et al.* (2020): RICOPILI: Rapid Imputation for COnsortias PIpeLIne. *Bioinformatics* 36: 930–933.
75. Chen C-Y, Lee PH, Castro VM, Minnier J, Charney AW, Stahl EA, *et al.* (2018): Genetic validation of bipolar disorder identified by automated phenotyping using electronic health records. *Transl Psychiatry* 8: 86.
76. Charney AW, Ruderfer DM, Stahl EA, Moran JL, Chambert K, Belliveau RA, *et al.* (2017): Evidence for genetic heterogeneity between clinical subtypes of bipolar disorder. *Transl Psychiatry* 7: e993.

77. Castro VM, Minnier J, Murphy SN, Kohane I, Churchill SE, Gainer V, *et al.* (2015): Validation of electronic health record phenotyping of bipolar disorder cases and controls. *Am J Psychiatry* 172: 363–372.
78. Nalichowski R, Keogh D, Chueh HC, Murphy SN (2006): Calculating the benefits of a Research Patient Data Repository. *AMIA Annu Symp Proc* 2006: 1044.
79. Zeng QT, Goryachev S, Weiss S, Sordo M, Murphy SN, Lazarus R (2006): Extracting principal diagnosis, co-morbidity and smoking status for asthma research: evaluation of a natural language processing system. *BMC Medical Informatics and Decision Making* 6: 30.
80. Karlson EW, Boutin NT, Hoffnagle AG, Allen NL (2016): Building the Partners HealthCare Biobank at Partners Personalized Medicine: Informed Consent, Return of Research Results, Recruitment Lessons and Operational Considerations. *J Pers Med* 6: 2.
81. Magnus P, Birke C, Vejrup K, Haugan A, Alsaker E, Daltveit AK, *et al.* (2016): Cohort Profile Update: The Norwegian Mother and Child Cohort Study (MoBa). *International Journal of Epidemiology* 45: 382–388.
82. Paltiel L, Haugan A, Skjerden T, Harbak K, Bækken S, Stensrud NK, *et al.* (2014): The biobank of the Norwegian mother and child cohort study – Present status. *Norsk Epidemiologi* 24: 29–35.
83. Helgeland Ø, Vaudel M, Juliusson PB, Lingaas Holmen O, Juodakis J, Bacelis J, *et al.* (2019): Genome-wide association study reveals dynamic role of genetic variation in infant and early childhood growth [no. 1]. *Nat Commun* 10: 4448.
84. Zhou W, Nielsen JB, Fritsche LG, Dey R, Gabrielsen ME, Wolford BN, *et al.* (2018): Efficiently controlling for case-control imbalance and sample relatedness in large-scale genetic association studies. *Nature Genetics* 50: 1335–1341.
85. Najman JM, Alati R, Bor W, Clavarino A, Mamun A, McGrath JJ, *et al.* (2015): Cohort Profile Update: The Mater-University of Queensland Study of Pregnancy (MUSP). *Int J Epidemiol* 44: 78–78f.
86. Gaziano JM, Concato J, Brophy M, Fiore L, Pyarajan S, Breeling J, *et al.* (2016): Million Veteran Program: A mega-biobank to study genetic influences on health and disease. *J Clin Epidemiol* 70: 214–223.
87. Levey DF, Gelernter J, Polimanti R, Zhou H, Cheng Z, Aslan M, *et al.* (2020): Reproducible Genetic Risk Loci for Anxiety: Results From ∼200,000 Participants in the Million Veteran Program. *Am J Psychiatry* 177: 223–232.
88. Forstner AJ, Awasthi S, Wolf C, Maron E, Erhardt A, Czamara D, *et al.* (2021): Genome-wide association study of panic disorder reveals genetic overlap with neuroticism and depression. *Mol Psychiatry* 26: 4179–4190.
89. Ripke S, Neale BM, Corvin A, Walters JTR, Farh KH, Holmans PA, *et al.* (2014): Biological insights from 108 schizophrenia-associated genetic loci. *Nature* 511: 421–427.
90. Witt SH, Streit F, Jungkunz M, Frank J, Awasthi S, Reinbold CS, *et al.* (2017): Genome-wide association study of borderline personality disorder reveals genetic overlap with bipolar disorder, major depression and schizophrenia. *Transl Psychiatry* 7: e1155.
91. Delaneau O, Marchini J, Zagury JF (2011): A linear complexity phasing method for thousands of genomes. *Nature Methods 2011 9:2* 9: 179–181.
92. Willer CJ, Li Y, Abecasis GR (2010): METAL: Fast and efficient meta-analysis of genomewide association scans. *Bioinformatics* 26: 2190–2191.
93. Lupton MK, Robinson GA, Adam RJ, Rose S, Byrne GJ, Salvado O, *et al.* (2021): A prospective cohort study of prodromal Alzheimer's disease: Prospective Imaging Study of Ageing: Genes, Brain and Behaviour (PISA). *Neuroimage Clin* 29: 102527.
94. Medland SE, Nyholt DR, Painter JN, McEvoy BP, McRae AF, Zhu G, *et al.* (2009): Common variants in the trichohyalin gene are associated with straight hair in Europeans. *Am J Hum Genet* 85: 750–755.
95. Byrne EM, Kirk KM, Medland SE, McGrath JJ, Colodro-Conde L, Parker R, *et al.* (2020): Cohort profile: the Australian genetics of depression study. *BMJ Open* 10: e032580.
96. Olsen CM, Green AC, Neale RE, Webb PM, Cicero RA, Jackman LM, *et al.* (2012): Cohort profile: the QSkin Sun and Health Study. *Int J Epidemiol* 41: 929–929i.
97. Lichtenstein P, De Faire U, Floderus B, Svartengren M, Svedberg P, Pedersen NL (2002): The Swedish Twin Registry: a unique resource for clinical, epidemiological and genetic studies. *J Intern Med* 252: 184–205.

98. Magnusson PKE, Almqvist C, Rahman I, Ganna A, Viktorin A, Walum H, *et al.* (2013): The swedish twin registry: Establishment of a biobank and other recent developments. *Twin Research and Human Genetics* 16: 317–329.
99. Lichtenstein P, Sullivan PF, Cnattingius S, Gatz M, Johansson S, Carlstrom E, *et al.* (2006): The Swedish Twin Registry in the third millennium: an update. *Twin research and human genetics : the official journal of the International Society for Twin Studies* 9: 875–882.
100. Ludvigsson JF, Andersson E, Ekbom A, Feychting M, Kim J-L, Reuterwall C, *et al.* (2011): External review and validation of the Swedish national inpatient register. *BMC Public Health* 11: 450.
101. Wettermark B, Hammar N, Fored CM, Leimanis A, Otterblad Olausson P, Bergman U, *et al.* (2007): The new Swedish Prescribed Drug Register--opportunities for pharmacoepidemiological research and experience from the first six months. *Pharmacoepidemiol Drug Saf* 16: 726–735.
102. Anckarsäter H, Lundström S, Kollberg L, Kerekes N, Palm C, Carlström E, *et al.* (2011): The Child and Adolescent Twin Study in Sweden (CATSS). *Twin research and human genetics : the official journal of the International Society for Twin Studies* 14: 495–508.
103. Birmaher B, Khetarpal S, Brent D, Cully M, Balach L, Kaufman J, Neer SM (1997): The Screen for Child Anxiety Related Emotional Disorders (SCARED): scale construction and psychometric characteristics. *J Am Acad Child Adolesc Psychiatry* 36: 545–553.
104. Van Dam NT, Earleywine M (2011): Validation of the Center for Epidemiologic Studies Depression Scale—Revised (CESD-R): Pragmatic depression assessment in the general population. *Psychiatry Research* 186: 128–132.
105. McCarthy S, Das S, Kretzschmar W, Delaneau O, Wood AR, Teumer A, *et al.* (2016): A reference panel of 64,976 haplotypes for genotype imputation. *Nature Genetics 2016 48:10* 48: 1279–1283.
106. Durbin R, Barrett J (2014): Efficient haplotype matching and storage using the positional Burrows–Wheeler transform (PBWT). *Bioinformatics* 30: 1266–1272.
107. Mosing MA, Pedersen NL, Cesarini D, Johannesson M, Magnusson PKE, Nakamura J, *et al.* (2012): Genetic and environmental influences on the relationship between flow proneness, locus of control and behavioral inhibition. *PLoS One* 7: e47958.
108. Kendler KS, Gardner CO, Gatz M, Pedersen NL (2007): The sources of co-morbidity between major depression and generalized anxiety disorder in a Swedish national twin sample. *Psychol Med* 37: 453–462.
109. Davis KAS, Coleman JRI, Adams M, Allen N, Breen G, Cullen B, *et al.* (2020): Mental health in UK Biobank - development, implementation and results from an online questionnaire completed by 157 366 participants: a reanalysis. *BJPsych Open* 6: e18.
110. Bycroft C, Freeman C, Petkova D, Band G, Elliott LT, Sharp K, *et al.* (2018): The UK Biobank resource with deep phenotyping and genomic data. *Nature 2018 562:7726* 562: 203–209.
111. Elliott LT, Sharp K, Alfaro-Almagro F, Shi S, Miller KL, Douaud G, *et al.* (2018): Genome-wide association studies of brain imaging phenotypes in UK Biobank [no. 7726]. *Nature* 562: 210–216.
112. Coon H, Shabalin A, Bakian AV, DiBlasi E, Monson ET, Kirby A, *et al.* (2022): Extended familial risk of suicide death is associated with younger age at death and elevated polygenic risk of suicide. *Am J Med Genet B Neuropsychiatr Genet* 189: 60–73.
113. Docherty AR, Mullins N, Ashley-Koch AE, Qin X, Coleman JRI, Shabalin A, *et al.* (2023): GWAS Meta-Analysis of Suicide Attempt: Identification of 12 Genome-Wide Significant Loci and Implication of Genetic Risks for Specific Health Factors. *AJP* 180: 723–738.
114. Chen W, Wu Y, Zheng Z, Qi T, Visscher PM, Zhu Z, Yang J (n.d.): Improved analyses of GWAS summary statistics by reducing data heterogeneity and errors. https://doi.org/10.1101/2020.07.09.196535
115. Purves KL, Coleman JRI, Meier SM, Rayner C, Davis KAS, Cheesman R, *et al.* (2019): A major role for common genetic variation in anxiety disorders. *Molecular Psychiatry*. https://doi.org/10.1038/s41380-019-0559-1
116. Lima Cunha D, Arno G, Corton M, Moosajee M (2019): The Spectrum of PAX6 Mutations and Genotype-Phenotype Correlations in the Eye. *Genes (Basel)* 10: 1050.

117. Cocas LA, Georgala PA, Mangin J-M, Clegg JM, Kessaris N, Haydar TF, *et al.* (2011): Pax6 is required at the telencephalic pallial-subpallial boundary for the generation of neuronal diversity in the postnatal limbic system. *J Neurosci* 31: 5313–5324.
118. Waclaw RR, Ehrman LA, Pierani A, Campbell K (2010): Developmental Origin of the Neuronal Subtypes That Comprise the Amygdalar Fear Circuit in the Mouse. *J Neurosci* 30: 6944–6953.
119. Umeda T, Takashima N, Nakagawa R, Maekawa M, Ikegami S, Yoshikawa T, *et al.* (2010): Evaluation of Pax6 mutant rat as a model for autism. *PLoS One* 5: e15500.
120. Nagel M, Jansen PR, Stringer S, Watanabe K, De Leeuw CA, Bryois J, *et al.* (2018): Meta-analysis of genome-wide association studies for neuroticism in 449,484 individuals identifies novel genetic loci and pathways. *Nature Genetics* 50. https://doi.org/10.1038/s41588-018-0151-7
121. Lowenstein ED, Ruffault P-L, Misios A, Osman KL, Li H, Greenberg RS, *et al.* (2023): Prox2 and Runx3 vagal sensory neurons regulate esophageal motility. *Neuron* 111: 2184-2200.e7.
122. Cali E, Rocca C, Salpietro V, Houlden H (2021): Epileptic Phenotypes Associated With SNAREs and Related Synaptic Vesicle Exocytosis Machinery. *Front Neurol* 12: 806506.
123. Iwamoto Y, Morinobu S, Takahashi T, Yamawaki S (2007): Single prolonged stress increases contextual freezing and the expression of glycine transporter 1 and vesicle-associated membrane protein 2 mRNA in the hippocampus of rats. *Prog Neuropsychopharmacol Biol Psychiatry* 31: 642–651.
124. Mathew RS, Tatarakis A, Rudenko A, Johnson-Venkatesh EM, Yang YJ, Murphy EA, *et al.* (2016): A microRNA negative feedback loop downregulates vesicle transport and inhibits fear memory. *Elife* 5: e22467.
125. Abuhatzira L, Shamir A, Schones DE, Schäffer AA, Bustin M (2011): The Chromatin-binding Protein HMGN1 Regulates the Expression of Methyl CpG-binding Protein 2 (MECP2) and Affects the Behavior of Mice. *J Biol Chem* 286: 42051–42062.
