# Supplementary material for: Genome-wide association study of major anxiety disorders in 122,341 European-ancestry cases identifies 58 loci and highlights GABAergic signaling
Source: Nat Genet. 2026 Feb 3;58(2):275–88. doi: 10.1038/s41588-025-02485-8 (PMC12900644; doi:10.1038/s41588-025-02485-8)
Supplement: Supplementary file 2 — Reporting Summary [file 41588_2025_2485_MOESM2_ESM.pdf]

nature portfolio

Corresponding author(s): John Hettema, Thalia Eley, Jürgen Deckert, Manuel Mattheisen

Last updated by author(s): 11/13/25/2025

Reporting Summary

Nature Portfolio wishes to improve the reproducibility of the work that we publish. This form provides structure for consistency and transparency in reporting. For further information on Nature Portfolio policies, see our Editorial Policies and the Editorial Policy Checklist.

Statistics

For all statistical analyses, confirm that the following items are present in the figure legend, table legend, main text, or Methods section.

n/a	Confirmed	
☐	☒	The exact sample size (n) for each experimental group/condition, given as a discrete number and unit of measurement
☐	☒	A statement on whether measurements were taken from distinct samples or whether the same sample was measured repeatedly
☐	☒	The statistical test(s) used AND whether they are one- or two-sided<br>*Only common tests should be described solely by name; describe more complex techniques in the Methods section.*
☐	☒	A description of all covariates tested
☐	☒	A description of any assumptions or corrections, such as tests of normality and adjustment for multiple comparisons
☐	☒	A full description of the statistical parameters including central tendency (e.g. means) or other basic estimates (e.g. regression coefficient) AND variation (e.g. standard deviation) or associated estimates of uncertainty (e.g. confidence intervals)
☐	☒	For null hypothesis testing, the test statistic (e.g. F, t, r) with confidence intervals, effect sizes, degrees of freedom and P value noted<br>*Give P values as exact values whenever suitable.*
☒	☐	For Bayesian analysis, information on the choice of priors and Markov chain Monte Carlo settings
☒	☐	For hierarchical and complex designs, identification of the appropriate level for tests and full reporting of outcomes
☐	☒	Estimates of effect sizes (e.g. Cohen's d, Pearson's r), indicating how they were calculated

Our web collection on statistics for biologists contains articles on many of the points above.

Software and code

Policy information about availability of computer code

Data collection	This is fully described in the Methods, associated Supplementary Information, and original GWAS publications. In brief, individual level imputed data and summary statistics from previous publications and additional ANX case-control studies were re-evaluated in a meta-analysis for association with ANX.
Data analysis	This is fully described in the Online Methods and associated Supplementary Information. In brief: Quality control and genetic association analyses was performed either using the Ricopili (v 1118b) pipeline: https://github.com/Nealelab/ricopili, which relies on the following software: Eigensoft 6.0.1 (incl. smartPCA), Plink 1.9, METAL 2011-03-25 or in some samples with comparable software tools and approaches (more details can be found in the Supplemental Table S1 and the Supplemental Text). MegaPRS polygenic risk scores were estimated using the GenoPred (92) pipeline. SNP-based finemapping was performed using FINEMAP v.1.3.1. Genomic SEM v 0.04 was used to conduct structural equation modeling. LDSC (v 1.0.1; https://github.com/bulik/ldsc) was used to estimate heritability and bivariate genetic correlations. We used FUMA v1.6.1 to examine the functional significance of loci. Gene-based analyses were performed using MAGMA (38) v1.08 as implemented in FUMA. Tissue and cell-type enrichment of GWAS association signals was conducted using MAGMA (v1.08). We performed transcriptome/proteome/methylome -wide association studies using SMR (v.1.03). We conducted gene-drug interaction analyses by applying the DrugTargetor method. We performed bi-directional generalized summary-data based mendelian randomization using GSMR v1.1.1 available in the GSMR R package. R v3.4 was used in general for statistical analyzes and plotting (https://www.Rproject.org).

For manuscripts utilizing custom algorithms or software that are central to the research but not yet described in published literature, software must be made available to editors and reviewers. We strongly encourage code deposition in a community repository (e.g. GitHub). See the Nature Portfolio guidelines for submitting code & software for further information.

Data

Policy information about availability of data

All manuscripts must include a data availability statement. This statement should provide the following information, where applicable:

- Accession codes, unique identifiers, or web links for publicly available datasets
- A description of any restrictions on data availability
- For clinical datasets or third party data, please ensure that the statement adheres to our policy

Upon manuscript publication, the primary meta-analyzed summary statistics will be made available via the Psychiatric Genomics Consortium Download page (https://www.med.unc.edu/pgc/download- results/).

Research involving human participants, their data, or biological material

Policy information about studies with human participants or human data. See also policy information about sex, gender (identity/presentation), and sexual orientation and race, ethnicity and racism.

Reporting on sex and gender	For most samples, the number of female and male cases or controls was not provided as part of this project and therefore not reported. Sex and/or gender based analyses were not performed
Reporting on race, ethnicity, or other socially relevant groupings	Primary analyses are limited to European ancestry samples, and no race, ethnicity or other socially relevant groupings have been shared for this project. We conducted secondary analyses (replication or polygenic risk scoring) in participants of African descent in the MVP and UK Biobank samples and participants of South Asian descent in the UK Biobank sample as officially designated in those datasets.
Population characteristics	Data from many different samples were included. See above and below and details in the manuscript and Supplement.
Recruitment	Details are provided in the Supplemental material and vary for the different studies that are part of the meta-analysis. In brief, samples were either ascertained in a clinical setting, a community setting, as part of larger biobanking efforts, or as part of studies looking into comorbid phenotypes (such as MDD, ASD, and ADHD). Our replication dataset was collected in 23andMe.
Ethics oversight	The details of the IRB/oversight body that provided approval or exemption for the research described are given below for 23andMe and in the Supplement for all the primary samples.<br><br>23andMe replication dataset: Participants provided informed consent and volunteered to participate in the research online, under a protocol approved by the external AAHRPP-accredited IRB, Ethical & Independent (E&I) Review Services. As of 2022, E&I Review Services is part of Salus IRB (https://www.versiticlinicaltrials.org/salusirb).

Note that full information on the approval of the study protocol must also be provided in the manuscript.

Field-specific reporting

Please select the one below that is the best fit for your research. If you are not sure, read the appropriate sections before making your selection.

☒ Life sciences ☐ Behavioural & social sciences ☐ Ecological, evolutionary & environmental sciences

For a reference copy of the document with all sections, see nature.com/documents/nr-reporting-summary-flat.pdf

Life sciences study design

All studies must disclose on these points even when the disclosure is negative.

Sample size	Sample sizes are provided for each contributing study and summed for the overall meta-analysis. We also estimated an effective sample size.
Data exclusions	Within each analyzed cohort except one we aimed at analyzing genetically homogeneous samples of unrelated individuals. Related individuals were excluded based on Identity by State analyses and genetic outliers were excluded based on principal component analyses. (The exception was the QIMR family-based samples which were analyzed using SAIGE that is able to account for relatedness between individuals through fitting a genetic relatedness matrix to the model.)
Replication	After identifying GWS associations in the primary GWAS, lead SNPs were tested for replication in the commercial database of 23andMe using 1,175,012 ANX self-report cases and 1,956,379 controls of European ancestry.
Randomization	This was not a clinical outcomes study, so randomization was not applied.
Blinding	Due to the different ascertainment strategies, blinding was dealt with differently across the different studies. More information can be found in the Supplemental information.

Reporting for specific materials, systems and methods

We require information from authors about some types of materials, experimental systems and methods used in many studies. Here, indicate whether each material, system or method listed is relevant to your study. If you are not sure if a list item applies to your research, read the appropriate section before selecting a response.

Materials & experimental systems

n/a	Involved in the study
☒	☐ Antibodies
☒	☐ Eukaryotic cell lines
☒	☐ Palaeontology and archaeology
☒	☐ Animals and other organisms
☒	☐ Clinical data
☒	☐ Dual use research of concern
☒	☐ Plants

Methods

n/a	Involved in the study
☒	☐ ChIP-seq
☒	☐ Flow cytometry
☒	☐ MRI-based neuroimaging

Plants

Seed stocks	N/A
Novel plant genotypes	N/A
Authentication	N/A
